# ReproPhylo: An Environment for Reproducible Phylogenomics

**DOI:** 10.1371/journal.pcbi.1004447

**Published:** 2015-09-03

**Authors:** Amir Szitenberg, Max John, Mark L. Blaxter, David H. Lunt

**Affiliations:** 1 Evolutionary Biology Group, School of Biological, Biomedical & Environmental Sciences, The University of Hull, Hull, United Kingdom; 2 Institute of Evolutionary Biology, The University of Edinburgh, Edinburgh, United Kingdom; University of Canterbury, NEW ZEALAND

## Abstract

The reproducibility of experiments is key to the scientific process, and particularly necessary for accurate reporting of analyses in data-rich fields such as phylogenomics. We present ReproPhylo, a phylogenomic analysis environment developed to ensure experimental reproducibility, to facilitate the handling of large-scale data, and to assist methodological experimentation. Reproducibility, and instantaneous repeatability, is built in to the ReproPhylo system and does not require user intervention or configuration because it stores the experimental workflow as a single, serialized Python object containing explicit provenance and environment information. This ‘single file’ approach ensures the persistence of provenance across iterations of the analysis, with changes automatically managed by the version control program Git. This file, along with a Git repository, are the primary reproducibility outputs of the program. In addition, ReproPhylo produces an extensive human-readable report and generates a comprehensive experimental archive file, both of which are suitable for submission with publications. The system facilitates thorough experimental exploration of both parameters and data. ReproPhylo is a platform independent CC0 Python module and is easily installed as a Docker image or a WinPython self-sufficient package, with a Jupyter Notebook GUI, or as a slimmer version in a Galaxy distribution.

This is a *PLOS Computational Biology* Software paper.

## Introduction

Experimental reproducibility has become a widely discussed issue in many areas of science [1,2]. Strict experimental reproducibility is not common in any area of the biological sciences and while the reasons for this may be varied they include the technical challenges in routine and robust implementation. Phylogenetic analyses are very widely used across the biological sciences [3], and, even in studies that are not primarily phylogenetic, the understanding of phylogenetic relationships is almost always required for a meaningful statistical inference [4–6]. Despite this importance, the reproducibility of phylogenetic experiments is low, and Magee et al. [7] estimated that 60% of published phylogenetic analyses are “lost to science” due to the unavailability of the underlying data, an outcome also predicted in other areas of biology [8]. However, even the public archiving of all data does not ensure reproducibility, since complete knowledge of the analytical software, software versions, software parameters, dependencies and operating system versions can be very challenging to both discover and recreate from published manuscripts. The increasing quantity of DNA sequence data available, and the proliferation of analytic toolkits, makes phylogenetics carried out on a genomic scale (“phylogenomics”) both especially powerful, and especially problematic to reproduce. Reproducibility in phylogenomics requires tracking of data provenance of multiple loci from many taxa, and, frequently, deeply nested analyses that explore, sift and partition data to achieve the end goals of biological understanding.

Here we introduce ReproPhylo, a Python package designed to deliver reproducible phylogenomic analyses. ReproPhylo promotes reproducibility on two levels. First, it eases the complex phylogenomic pipeline design process by providing a simple and concise scripting syntax for the execution of complex and forked phylogenetic workflows. Second, it automates reproducibility by employing well trusted containerization, versioning and provenance programs. In ReproPhylo, management of the experiment’s reproducibility and version control is carried out in a ‘frictionless’ manner in the background, without a need for user attention (although users have the option to access and tailor these aspects). Third, it ensures persistence and availability of metadata throughout the workflow, and in all the final products. With these three components of the analysis process considerably simplified, major important practices are addressed [9], and time and effort can be directed towards the core goals of understanding phylogenetic relationships by experimental parameter selection and data exploration, as the examples described here show (See Results section).

ReproPhylo is not the first package to provide phylogenetic workflow or pipeline tools [10–13]. A pipeline approach is a step forward from the point of view of reproducibility, as pipelines can serve as machine-readable records of analyses. Existing solutions [10–13] typically focus on the analysis itself, and do not attempt to provide complete reproducibility solutions. Several phylogenomic pipelines exist as web services [14–16], however, server-based analysis introduces additional complexities and reproducibility challenges, the main one of which is the dependency on a remote software environment. Osiris [17] achieves reproducibility through use of the Galaxy [18–20] reproducible bioinformatics environment, which can easily be used locally. Within the Galaxy framework, Osiris offers tools and format converters for widely used phylogenetic analysis programs, with user friendly and flexible GUI.

ReproPhylo explores an alternative, more generalised, approach to reproducibility, as it avoids dependency on any single high level software environment. It unifies the different components of a flexible, convenient, platform-independent, user friendly and reproducible workflow, drawing on the many advantages of standard data formats and community standard Biopython [21] code classes. ReproPhylo is simply accessed within a Jupyter Notebook (formerly IPython Notebook) [22]. We have also designed several basic ReproPhylo Galaxy tools, which produce self-contained and fully reproducible outputs, even outside the Galaxy system, as a proof of concept.

## Design and Implementation

ReproPhylo interfaces with existing phylogenetic analysis tools *via* standard data structures, such as SeqRecord or MultipleSeqAlignment Biopython objects. In addition, it imports and exports data as text files in all standard formats supported by Biopython [21], and does not itself implement any novel data formats.

ReproPhylo can be run using Jupyter Notebook [22], where it is interacted with using a simple and self-explanatory Python syntax (examples in S1 Methods). We provide a range of notebooks for different types of analysis with the ReproPhylo distribution, including one for the Lepidoptera case analysis presented below. These notebooks are examples of ‘literate programming’ [23] in that they combine instructions, documentation, and code. The user may modify these Notebook pipelines either trivially (e.g. just changing the input data and executing), or more substantially (by altering the nature or sequence of analyses *via* Python code). Our testing with undergraduates, postgraduates, and academics without coding experience indicates that Jupyter Notebook is an effective GUI for scientists lacking a background in programming.

### The ReproPhylo pipeline

**Fig 1:**
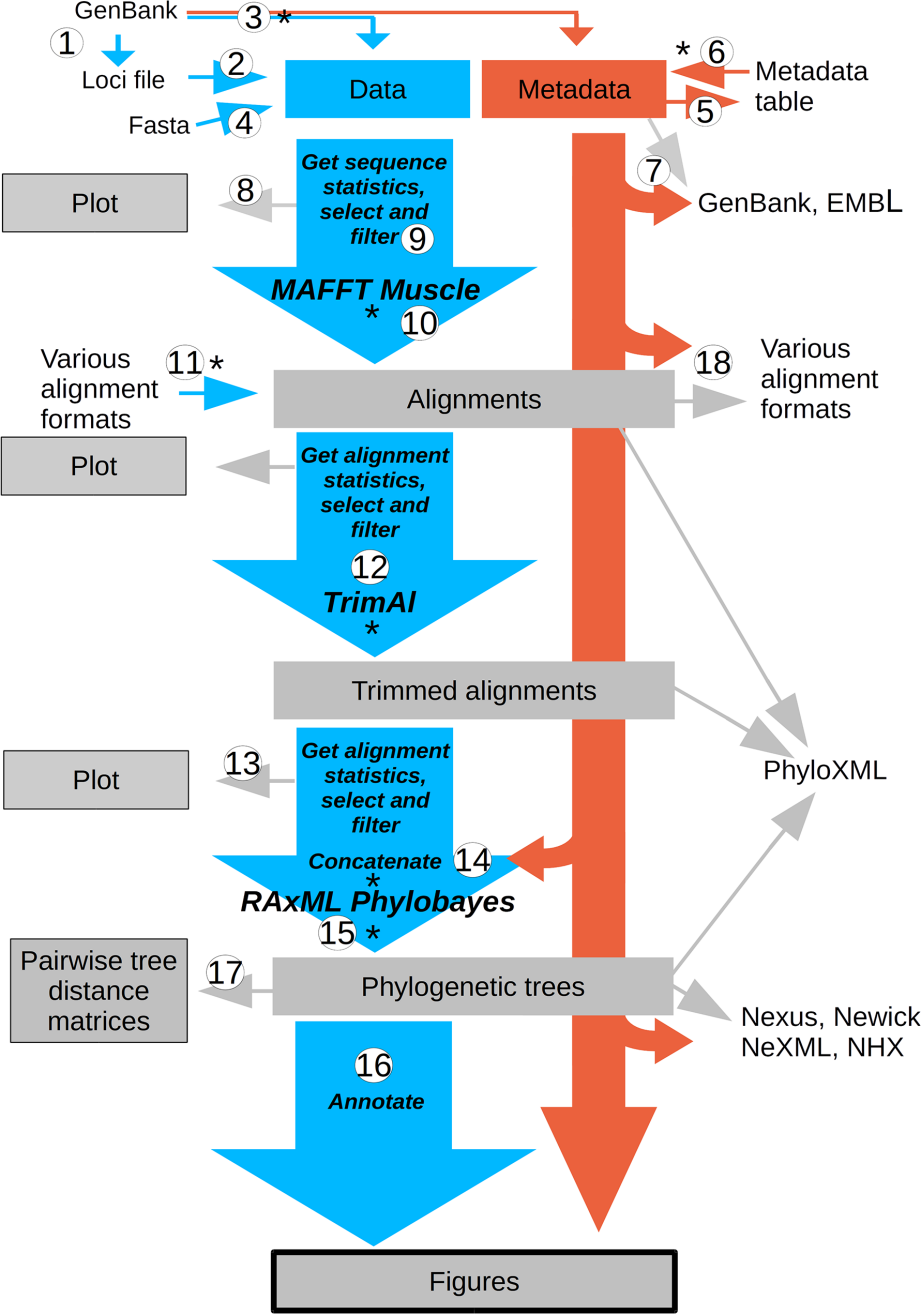
A typical ReproPhylo workflow. This illustration demonstrates the flow of data (blue arrows) and metadata (red arrows) through the phylogenetic analysis. Numbers on arrows correspond with code snippets in S1 Methods. Asterisks indicate an automatic pickle and Git checkpoint. The user can toggle between these checkpoints indefinitely using a built in ReproPhylo function.

ReproPhylo aids processes through the complete arc of a phylogenomics study: dataset collation, data analysis and visualisation/exploration. Table 1 lists the data classes in ReproPhylo and their associated methods and functions. Fig 1 illustrates a typical ReproPhylo workflow, and code snippets associated with each of the workflow steps are demonstrated in S1 Methods. The ReproPhylo module uses a set of Python packages to control the pipeline and report results and quality statistics. The workflow is carried out by Biopython [21] and ETE2 [24], the latter of which also powers tree annotation. The primary output data file format is PhyloXML, although other formats can be produced. Graphics other than phylogenetic trees, such as alignment statistics and sequence statistics box-plots, are produced using Matplotlib [25].

**Table 1:** Summary of the Python module structure.

Module feature	Description
**Class Locus**	Descriptor of the name, aliases, feature type and sequence type of an analysed locus
**Class Project**	Container for the input, intermediate and output datasets, and their metadata. Structured using Locus and Concatenation objects
method categories	
Read	Read data and metadata in any Biopython compatible format or tabular format for metadata
Filter	Filter sequences based on length, GC content or ID
edit_metadata	Programmatically manipulate sequence metadata
Align	Conduct sequence alignment(s) configured by a Conf object
Trim	Conduct alignment trimming configured by a Conf object
Tree	Conduct tree reconstruction(s) configured by a Conf object
Annotate	Annotate and root trees based on metadata stored in the Project
Write	Write files containing sequences, alignments, trees or metadata in any Biopython format
View	View alignments, statistics plots, occupancy tables etc. in the browser
Fetch	Copy a Project attribute (e.g. a tree or alignment object) into an independent variable
**Conf Classes**	A set of classes for configuring the different analytic steps
**Class LociStats**	Contains alignment and sequence parameters of the data in the Project
Methods	
Sort	sort the loci based on one of the available parameters
Plot	plot parameter boxplots
Slice	produce a supermatrix with certain parameter limits
Slide	create supermatrices by a sliding window approach along a gradient of a given parameter
**Class Concatenation**	Descriptor of the locus and OTU composition of a supermatrix
method categories	
Add	Add the concatenation to the analysis
Make	Prepare a supermatrix based on the instructions
**Function categories**	
list_loci	List loci found in a gb file, synonymize and choose from
Report	Write human readable report containing detailed methods and results
Pickle	Serialize/ Unserialize a Project object
Exonerate	Functions to run exonerate yielding metadata rich gb files
Bayestraits	Invokes BayesTraits using a Project object as the input source for both trees and traits

Dataset collation in ReproPhylo has three components: harvesting, selection and filtering. An example of *data harvest* would be importing all GenBank records for a specific taxonomic group from a Genbank format text file, and adding unpublished sequences from a fasta or ab1 format sequence file. Exonerate [26] can be deployed within ReproPhylo to harvest loci of interest from genome or transcript data *via* specialized functions. *Data selection* exploits ReproPhylo’s loci report to automatically include or exclude specific genes and coding sequences present in an input Genbank file. *Data filtering* automatically excludes or includes sequences, or loci, based on user specifications—length, GC content, sequence number or taxonomic coverage—informed by ReproPhylo’s sequence and alignment summary statistics reports.

The analysis workflow in ReproPhylo includes sequence alignment, alignment trimming, and tree reconstruction. These steps can be forked to explore alternative analytic approaches while tracking data provenance in each branch and step. We have included commonly used analysis tools for each step, and additional algorithms can be suggested, or included by modifying the ReproPhylo module code (described in the manual, http://goo.gl/yW6J1J). The first release of ReproPhylo can utilise the sequence aligners MAFFT [27], MUSCLE [28,29] and Pal2Nal [30]. Trimming of alignments to remove poorly aligned ‘gappy’ regions can improve analyses [31], and is carried out based on explicit trimming criteria using TrimAl [32]. Tree reconstruction programs accessible through ReproPhylo include RAxML [33] and PhyloBayes [34].

ReproPhylo facilitates phylogenetic output visualisation and exploration. Tree annotation, and creation of publication quality figures, is powered by ETE2 [24] and informed by metadata from the data harvest step provided to it by ReproPhylo. BayesTraits [35,36] is included for comparative phylogenetic analyses, and is invoked by a function which accepts a ReproPhylo Project object as the source of both the tree and trait information. Pairwise tree distances between trees in the Project can be computed and visualized (see Results section).

### Data provenance and reproducibility

**Fig 2:**
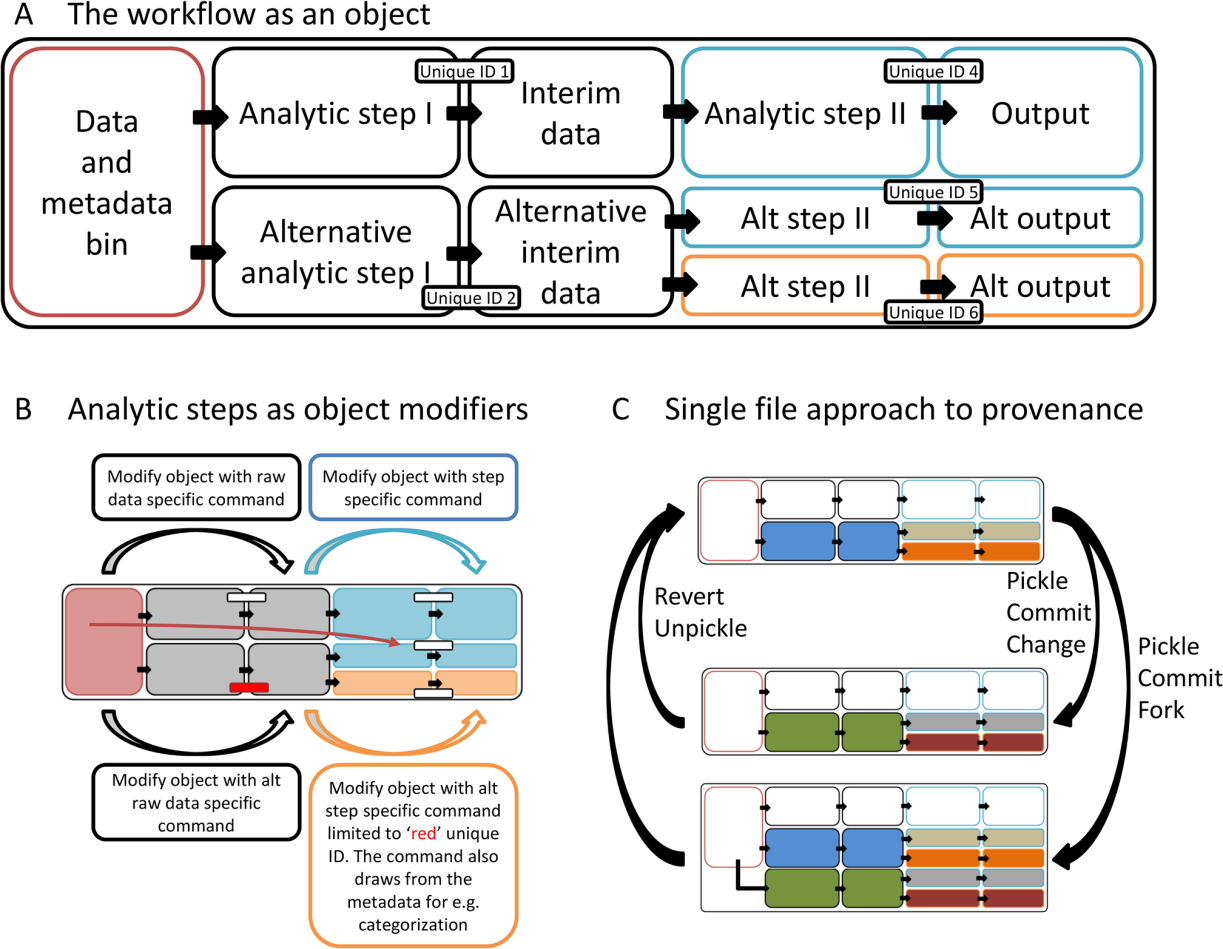
The phylogenetic workflow as a single Python object. (A) The workflow is contained as a single object with bins (attributes) for the raw data and metadata, as well as for the various workflow analyses and forks. These are made provenance-explicit with unique IDs and names. (B) Analyses are invoked via commands that modify the workflow object. A command can invoke batch analysis for all the relevant data in the object. For example, the command ‘align’ will apply for all the unaligned datasets. Commands can be limited to certain datasets using IDs. Commands can be customized using options. (C) Provenance survives version changes. The workflow object can be serialized (pickled) and then committed to a version control repository as a single file. Reverting to previous output version will also revert to the intermediate steps leading to it. Forks can be done post-hoc using the all-inclusive and provenance explicit workflow (pickled) object.

Data provenance, the recording of the input and transformation of information used to generate a result, is a key issue in reproducibility. To maintain phylogenomic data provenance, ReproPhylo keeps the full workflow in a single instance of the Project ReproPhylo class (Fig 2A). This object contains all the analytical steps and their outputs, together with machine and human readable unique process IDs that describe the provenance of each data object for both the programme and the user. In addition, the Project instance contains the metadata associated with each sequence of each locus, with a unique ID, which allows it to associate the metadata with its sequence or tree leaf in any of the existing data objects (the SeqRecord, MultipleSeqAlignment and Tree objects). Analysis is invoked by Project class methods, which modify the data (e.g. align the sequences), place the resulting data object (e.g. MultipleSeqAlignment) in the appropriate Project attribute (e.g. Project.alignments) under a unique ID (Fig 2B), update the binary file storing the Project, and commit it to the Git repository. In each analytical step metadata can be retrieved using unique sequence identifiers, and alternative analytic approaches (forks) can be stored within a single Project through their unique process IDs.

Since the complete workflow is represented as a single Python object, provenance can be maintained across different versions of the analysis (Fig 2C). ReproPhylo serializes (“pickles”) the Project object and maintains it as a binary file that allows the user to pause and resume the analysis seamlessly. ReproPhylo uses the version control program Git (git-scm.com) to record a version of the binary Project file each time it is modified, and thus allows forwards and backwards toggling of file versions. When an older version is restored, the full chain of intermediate results and the records detailing their production are restored throughout the workflow and across forks. ReproPhylo’s version control and reproducibility are implemented passively in the background and are frictionless for the user, requiring neither specialist knowledge nor action to produce a reproducible phylogenomics experiment. The integration of Git in ReproPhylo is demonstrated in S1 Example (also in http://dx.doi.org/10.6084/m9.figshare.1419590 and in nbviewer, http://goo.gl/g3XP5B).

To facilitate publication of the reproducible experiment, ReproPhylo produces a compressed experiment directory (.zip format) suitable for upload to a data repository such as FigShare (http://figshare.com/) or Dryad (http://datadryad.org/). This file contains trees and sequence alignments (in standard phyloXML format [37]), all analysis scripts, tree figure files, and a complete, human-readable report. The report includes a methods section ready for inclusion in a manuscript, which contains program versions, accession numbers, references etc., to which the digital object identifier of the full experimental record can be added. The compressed experiment directory also contains the binary file in which the serialized Project object is stored. This object contains all the data, metadata, method descriptions and results, and includes explicit provenance information. It can be used to revive the entire analysis, either in the ReproPhylo Docker container, in a local ReproPhylo installation or independently of ReproPhylo, and instantly repeat it or extend it. Another product of ReproPhylo is a Git repository, which can be published on websites such as Github (http://github.com/) and Figshare (http://figshare.com/). Both the compressed experiment directory and the Git repository satisfy all the Minimum Information about a Phylogenetic Analysis (MIAPA) goal [38], but the requirement for a description of the research objectives, by providing data files, data objects and human readable reports. They supersede the MIAPA requirements by also providing full software environment details and the machine readable scripts which have produced the intermediate and final files.

Version 1 of ReproPhylo is distributed as a Docker image (See Availability and Future Directions section). Using Docker as a work environment also facilitates reproducibility and reusability, as all relevant files can be committed to the image, generating a single Docker image file containing the computer environment, specific program copies, and data components of the finished analysis. Such containerisation approaches, which deliver both reproducible and easily reusable experiments, are powerful development and delivery tools [39].

### Example use case

Several examples of use of the ReproPhylo phylogenomic analytical pipeline are provided as Jupyter notebooks in the distribution files. We focus here on parameter space exploration using ReproPhylo to demonstrate the advantages of phylogenomic analysis delivered by a fully scripted, reproducible environment. In this use case we demonstrate exploration of the effect of the median residue conservation (gene variability level) in each locus on a resulting species topology, using an existing multigene dataset of lepidopteran species [40]. Loci with different levels of conservation may hold phylogenetic signal of events that occurred in different times in the past, or may be too conserved, or too rapidly evolving and saturated with homoplasies, to provide any signal at all [41]. We utilise Shannon Entropy (SE) [42] as a conservation scoring method [43]. The script generating this analysis is available as S2 Methods. The original Jupyter Notebook, together with the input and output files and figures, has been archived on FigShare (doi:10.6084/m9.figshare.1409423, goo.gl/KzFAvj), and has also been included as one of the tutorials in the current distribution of ReproPhylo (see ReproPhylo documentation at http://goo.gl/aZeRXf). A report with supplementary results generated by ReproPhylo is provided as S1 Results. Instructions on accessing the Project file in order to reproduce this demonstration are provided in the manual.

**Fig 3:**
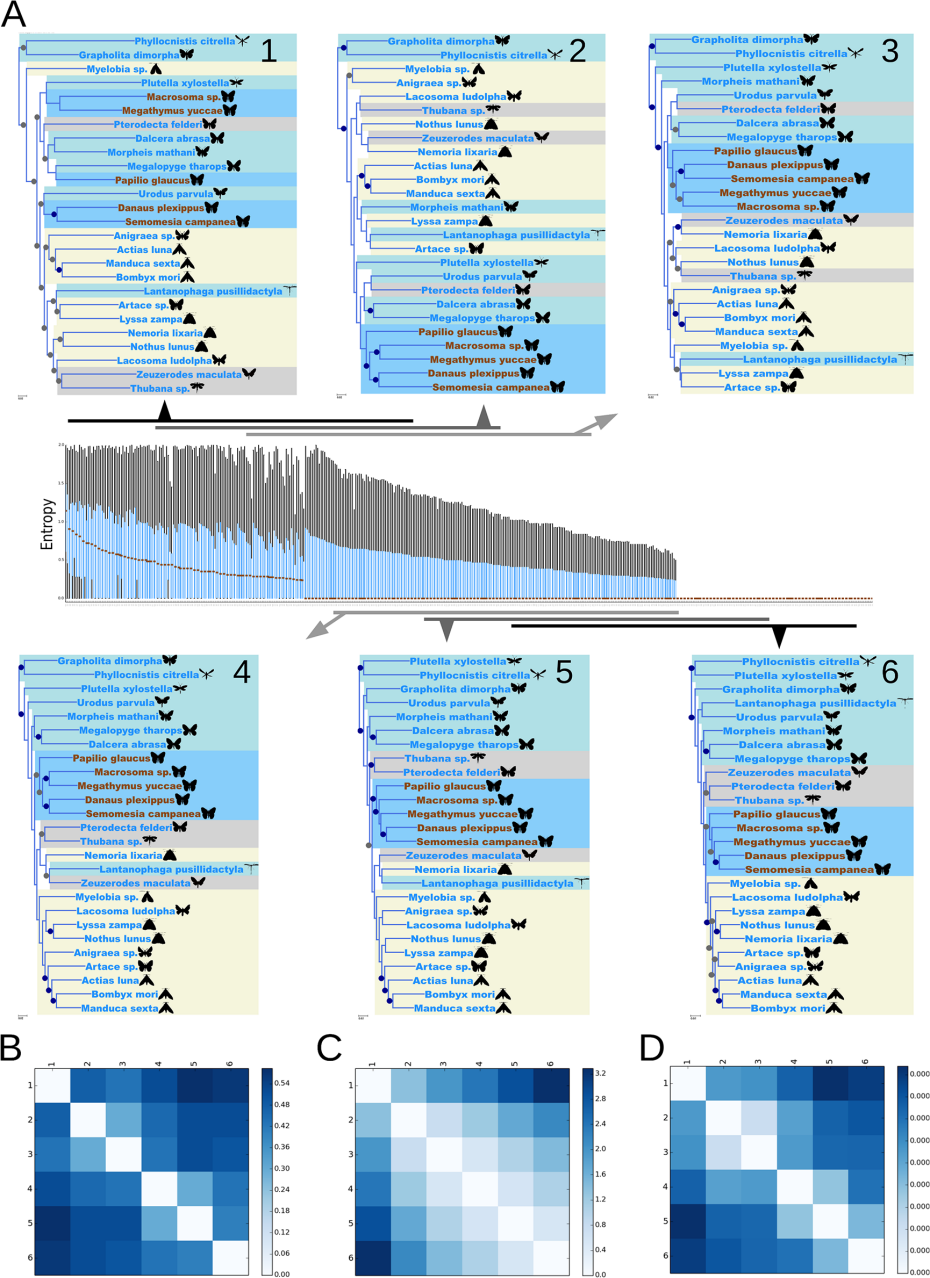
Exploratory phylogenomic analysis of a Lepidoptera dataset. (A) A nucleotide dataset from 26 species from Kawahara and Breinholt [40] was reanalyzed. Loci were sorted by their median, 75 percentile and 25 percentile entropy values (centre panel). For each locus, a box plot was generated. The medians are denoted by brown dots. The boxes (blue) represent the 25–75 percentiles. Whiskers (black) represent values that are found within a range outside the box, 1.5 times as long as the box (which is null, when the box itself has a null range) Trees (insets A 1–6) were reconstructed from 200-locus windows with 50 locus overlap between neighbouring windows. The windows are represented by black and gray horizontal bars, each with an arrow pointing to the tree generated from it. In trees 1–6, dark blue highlights denote Rhopalocera (butterfly) taxa, and light blue, gray and yellow highlights denote clades I, III and IV respectively (*sensu* Kawahara and Breinholt [40]). Bullets on nodes represent Bootstrap percentages (BP). Blue bullets represent maximal support. Other support values above 80% are denoted by gray bullets. (B-D) Three pairwise tree divergence metrics were calculated and presented as heatmaps, with the most divergent tree pairs denoted by dark blue and identical tree pairs by a white box. While the scales are not comparable among the metrics, the relative differences are. The metrics are (B) the Symmetric Distance of Robinson-Foulds [44], (C) the Branch Distance [45] and (D) evolutionary rate corrected Branch Distance [45].

We obtained a nucleotide sequence alignment of 465 loci from 26 Lepidoptera species [40]. Using a built-in function (S2 Methods, section 2.6.1), SE values [42], ignoring gap characters, were calculated for each residue in each locus. An entropy distribution plot (Fig 3A, centre) illustrates the differences in SE among the loci. This plot is typical of alignment statistics and representations produced by the ReproPhylo LociStats class (see Section 2.6.3 of S2 Methods for code generating this plot). Six supermatrices were extracted, each from a sliding window of 200 loci, starting with the highest entropy loci and ending with the lowest entropy loci, and shifting the window by 50 loci between subsets (Fig 3A). Lastly, following the original analysis, all 26 species were included in all of the supermatrices, which contained no missing data (S1 Results, S1 Methods section 2.7). Trees (Fig 2) were reconstructed as described in S2 Methods, sections 2.5–210. Note that data partition information is utilised by ReproPhylo automatically. The trees were formally compared using the Symmetric Distance of Robinson-Foulds [44] (Fig 3B), the Branch Distance [45,46] (Fig 3C), and a modified Branch Distance [45] (Fig 3D), with standardized evolutionary rate (S1 Methods, section 2.11).

### Reproducibility statement

The entire project workflow for our analysis was saved as a pickle file (S1 Results), a Git repository generated by ReproPhylo (doi:10.6084/m9.figshare.1409423), and a publishable archive file (S1 Results). The pickled workflow can most productively be used within the ReproPhylo environment, where it is possible to add data and repeat the analysis or extend the analysis without the need to repeat any previous step. Importantly, the data within the pickled workflow is accessible using Biopython, even in the absence of ReproPhylo. The archive file represents a more traditional approach to reproducibility, as it includes alignment and tree text files, the tree figures (Fig 3A), and a human readable report containing complete methods and results information.

## Results

We explored the partitioned Lepidoptera data for support for the clade Rhopalocera (butterflies) in loci with different SE values. Butterfly taxa are indicated in Fig 3A with dark blue highlight. The resulting topologies depend on the median entropy values in the dataset, with loci possessing low entropy values providing most support for Rhopalocera monophyly (Fig 3A trees 5–6). The result is similar for three other clades identified by Kawahara and Breinholt [40] (their clades I, III and IV; Fig 3A insets, light blue, yellow and gray highlights respectively). The entropy calculations were shown to be unbiased by the GC content or missing data (S1 Fig; generated by section 2.4.6, S2 Methods). Formal tree comparisons (Fig 2B–2D), showing the topological differences (Fig 3B), the branch length differences (Fig 3C), and a combination of both (Fig 3D), also illustrate the effect of entropy on the topology and branch-lengths. This reaffirms the importance of analytic control over confounding effects.

The key novelty in the ReproPhylo environment is the ease and flexibility with which a complex phylogenetic investigation such as this can be set up, and be instantaneously repeatable and reproducible without compromising the user’s control over parameter choice and configuration. ReproPhylo facilitates informed parameter choices and data filtering based on clearly documented and reproducible experimentation. Additional use cases are included with the package and they demonstrate the usage of additional components of the module and their interaction with Git and Docker.

ReproPhylo is an integrated environment for performing fully reproducible, platform independent, phylogenomics analyses that is highly accessible for scientists even without a strong computational background. ReproPhylo, by dealing with input and output formatting of data and results, can improve the accessibility and integration of existing computational tools. Phylogenetic analyses focussing on a single locus are becoming rarer as the power of modern genomics makes the *de novo* generation of large-scale data for multiple species feasible, especially with targeted sequencing approaches [47]. The rapid growth of public databases provides a resource that can be mined for new sets of loci across wide taxonomic spans, offering a second source of very large phylogenomic datasets. To exploit these new data, and at the same time deliver fully reproducible science that can lead to a truly incremental synthesis of evolution of life on earth, toolkits such as ReproPhylo that are large-data-ready, and natively reproducible will be essential.

## Availability and Future Directions

ReproPhylo is open source, using strictly open source dependencies, and is under active development within a publicly accessible Github repository (https://github.com/HullUni-bioinformatics/ReproPhylo). Documentation is provided as a version tracked publicly-editable Google Docs manual at http://goo.gl/yW6J1J, allowing corrections and expansions by the user community. A frozen version of the module (Version 1), utilizing Jupyter Notebook as interface, is available as a self-contained environment in a Docker image (http://goo.gl/JcHMGN). Bioinformatics pipelines may often be challenging to install but the use of a Docker image for distribution eliminates such difficulties, and facilitates installation on any system. The Docker image is accompanied by a shell script that will install and deploy the ReproPhylo image as a Docker container, with a local web browser based GUI. We also provide ReproPhylo as a WinPython version (see manual), and currently develop a Vagrant box solution (https://www.vagrantup.com/) for OSX. These will address any issues with the X11 server within Docker on Windows and Mac OSs. A repository containing the data and script for the analysis presented here is available on FigShare (http://dx.doi.org/10.6084/m9.figshare.1409423), as well as a repository containing the script and data for a demonstration of version control in ReproPhylo (http://dx.doi.org/10.6084/m9.figshare.1419590). The notebook containing the version control demonstration (http://goo.gl/g3XP5B) is also provided here as S1 Example. As a proof of concept, ReproPhylo is also provided as a Galaxy distribution (http://goo.gl/udsS3Q) containing ReproPhylo Galaxy tools. This version utilises the Galaxy framework, while retaining completely reproducible results even outside the Galaxy GUI.

Future development is intended to include an extended suite of quality control indices, allowing better control over large datasets. Specifically, ReproPhylo can benefit from analyses that allow one to detect misleading signal in phylogenies [48]. In addition, we would like to include Resource Description Framework (RDF) outputs and parsers that will allow interactions with online repositories utilizing formal ontology descriptions [49] of phylogenetic experiments (e.g. CDAO-store [50]). Finally, ReproPhylo is intended to be a community tool, and we hope its future development will be guided by input from users, either by pull requests or issue reporting and suggestions in the Github repository.

## Supporting Information

S1 Fig Loci statistics boxplots for data derived from [40]. For each locus, the plots illustrate the distributions of (from top to bottom) per-position entropy, per-position gap score [32], per position conservation score [32], sequence length and GC content. http://dx.doi.org/10.6084/m9.figshare.1409424
(TIFF)

S1 Methods An example code. The code snippets in this supplementary file are those associated with the numbered steps in the workflow illustrated in Fig 1. http://dx.doi.org/10.6084/m9.figshare.1502477.
(PDF)

S2 Methods Scripts used in this research. A static HTML representation of the code that was used to create all the analyses in this study. http://dx.doi.org/10.6084/m9.figshare.1409427
(HTML). Also in nbviewer: http://goo.gl/KzFAvj.

S1 Results ReproPhylo report. A results archive produced by ReproPhylo, containing the serialized Project, input and output files, scripts and an HTML report. http://dx.doi.org/10.6084/m9.figshare.1409488
(ZIP)

S1 Example A Jupyter notebook demonstrating version control in ReproPhylo (also available in FigShare (http://dx.doi.org/10.6084/m9.figshare.1419590) and nbviewer (http://goo.gl/g3XP5B)). (HTML)
