# Supplementary figures and images for: ReproPhylo: An Environment for Reproducible Phylogenomics
Source: PLoS Comput Biol. 2015 Sep 3;11(9):e1004447. doi: 10.1371/journal.pcbi.1004447 (PMC4559436; doi:10.1371/journal.pcbi.1004447)

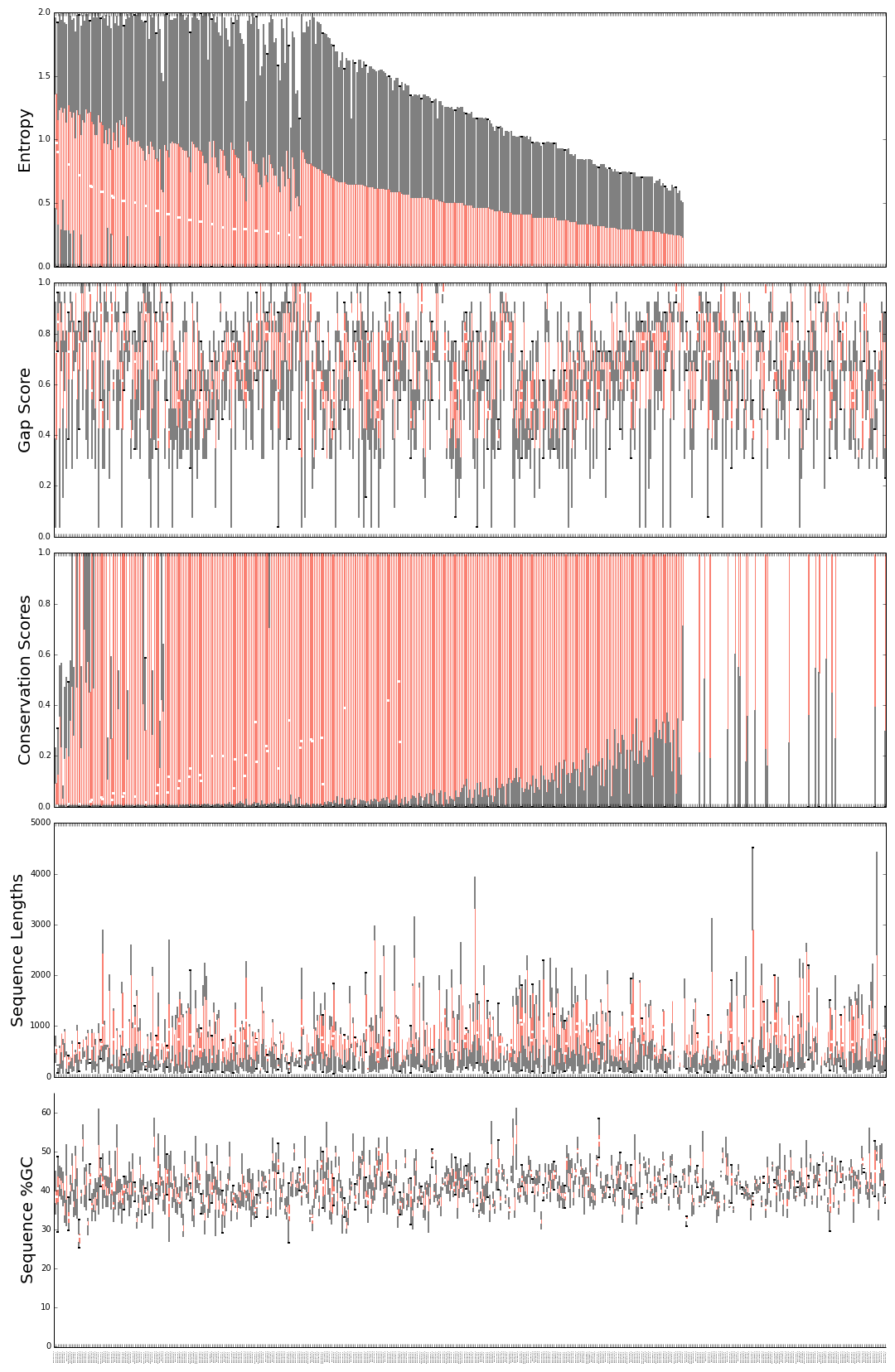

Supplement: S1 Fig — For each locus, the plots illustrate the distributions of (from top to bottom) per-position entropy, per-position gap score [32], per position conservation score [32], sequence length and GC content. http://dx.doi.org/10.6084/m9.figshare.1409424 (TIFF) [file pcbi.1004447.s001.tiff]

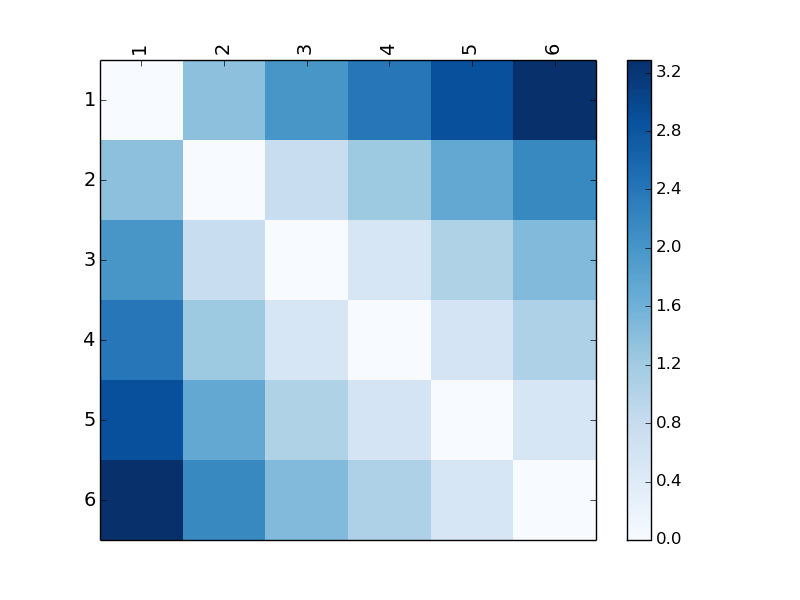

Supplement: S1 Results — A results archive produced by ReproPhylo, containing the serialized Project, input and output files, scripts and an HTML report. http://dx.doi.org/10.6084/m9.figshare.1409488 (ZIP) [file pcbi.1004447.s004.zip › report/files/1077.png]

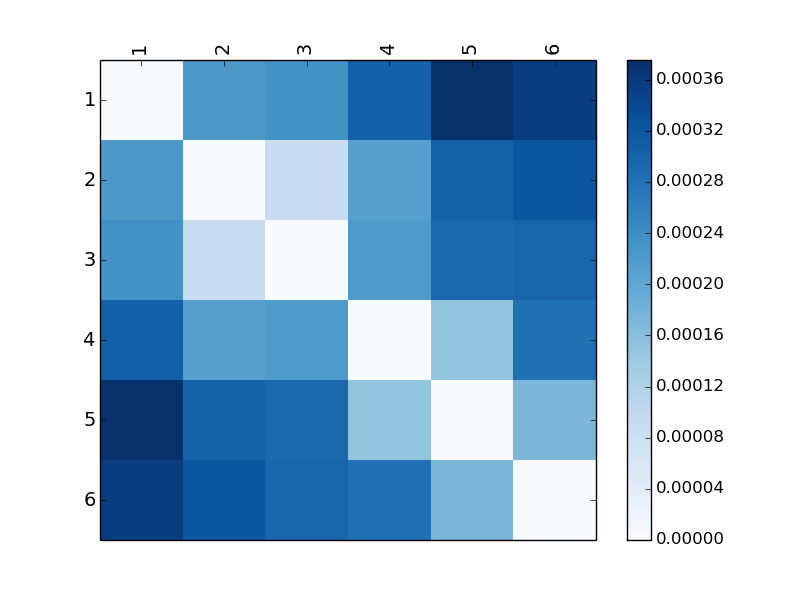

Supplement: S1 Results — A results archive produced by ReproPhylo, containing the serialized Project, input and output files, scripts and an HTML report. http://dx.doi.org/10.6084/m9.figshare.1409488 (ZIP) [file pcbi.1004447.s004.zip › report/files/1383.png]

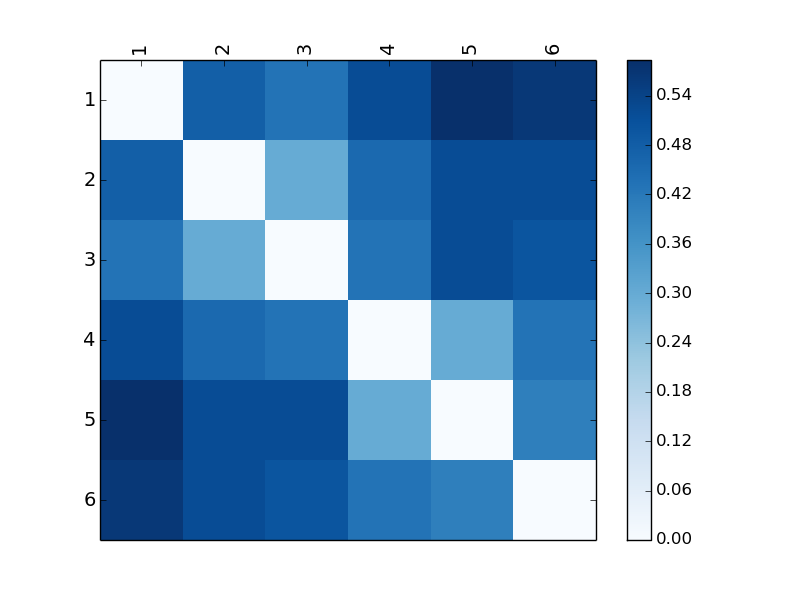

Supplement: S1 Results — A results archive produced by ReproPhylo, containing the serialized Project, input and output files, scripts and an HTML report. http://dx.doi.org/10.6084/m9.figshare.1409488 (ZIP) [file pcbi.1004447.s004.zip › report/files/1810.png]

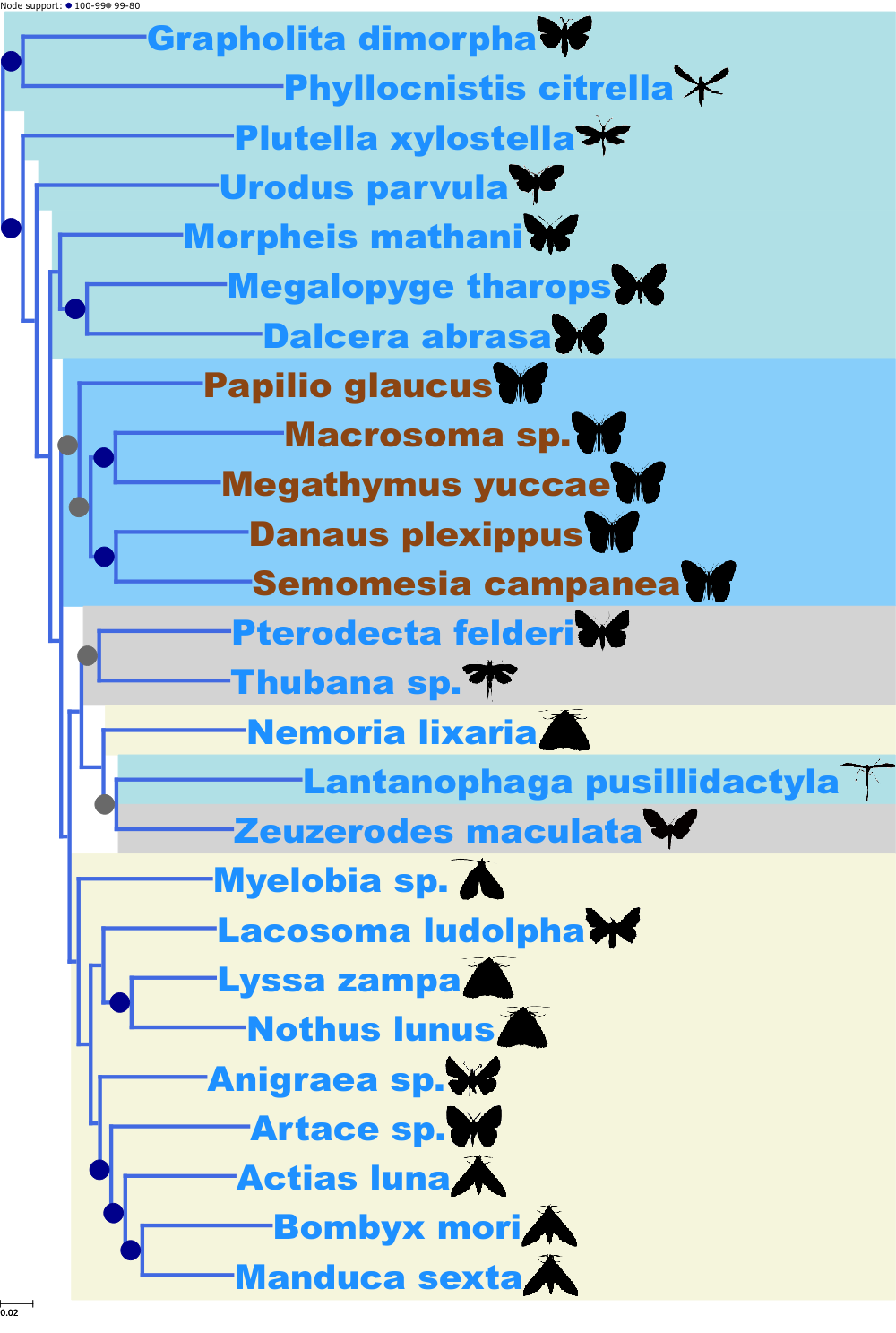

Supplement: S1 Results — A results archive produced by ReproPhylo, containing the serialized Project, input and output files, scripts and an HTML report. http://dx.doi.org/10.6084/m9.figshare.1409488 (ZIP) [file pcbi.1004447.s004.zip › report/files/357421429738848.49_entropy_0.00_0.00_loci_150_to_349.png]

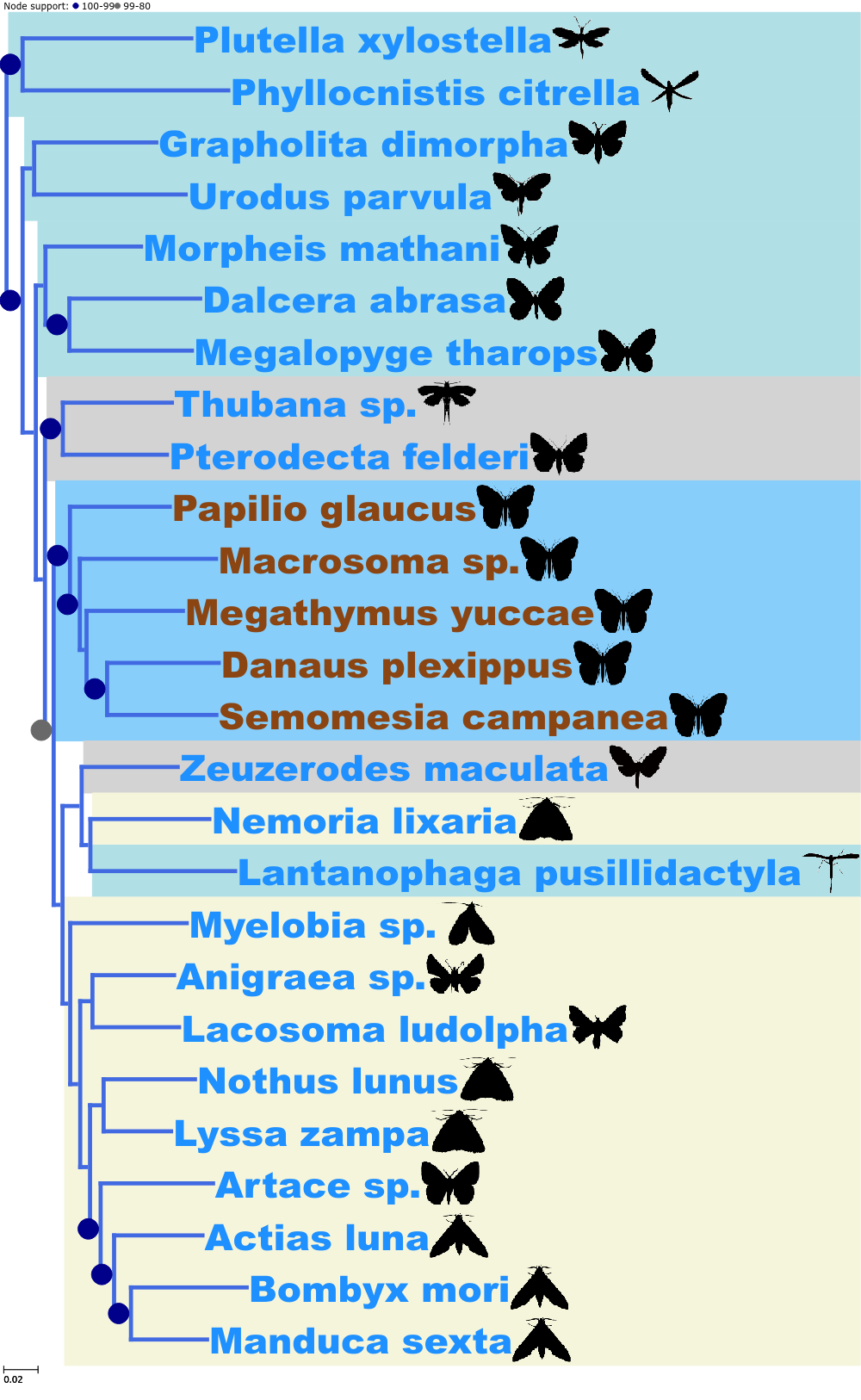

Supplement: S1 Results — A results archive produced by ReproPhylo, containing the serialized Project, input and output files, scripts and an HTML report. http://dx.doi.org/10.6084/m9.figshare.1409488 (ZIP) [file pcbi.1004447.s004.zip › report/files/357421429738848.49_entropy_0.00_0.00_loci_200_to_399.png]

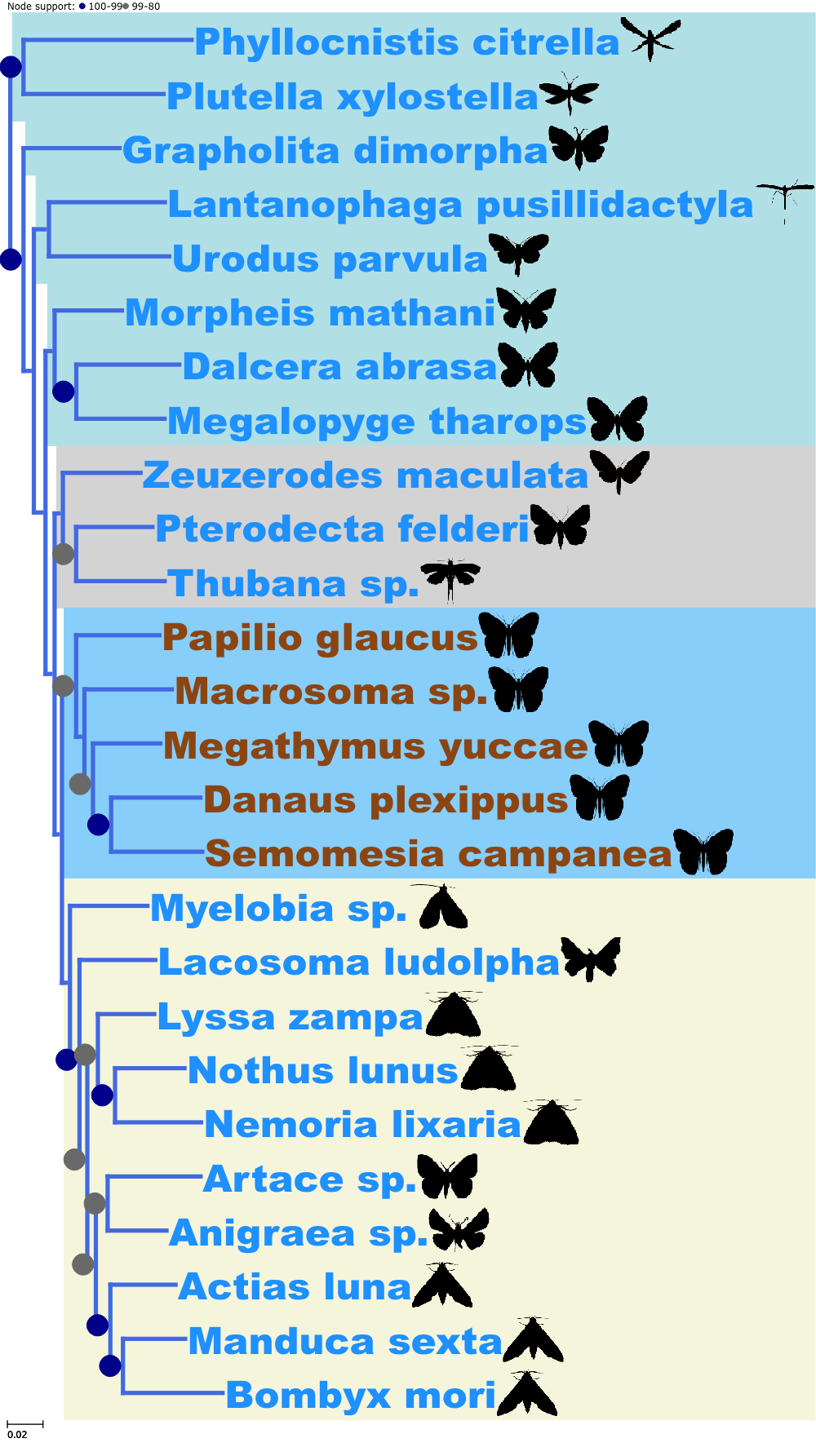

Supplement: S1 Results — A results archive produced by ReproPhylo, containing the serialized Project, input and output files, scripts and an HTML report. http://dx.doi.org/10.6084/m9.figshare.1409488 (ZIP) [file pcbi.1004447.s004.zip › report/files/357421429738848.49_entropy_0.00_0.00_loci_250_to_449.png]

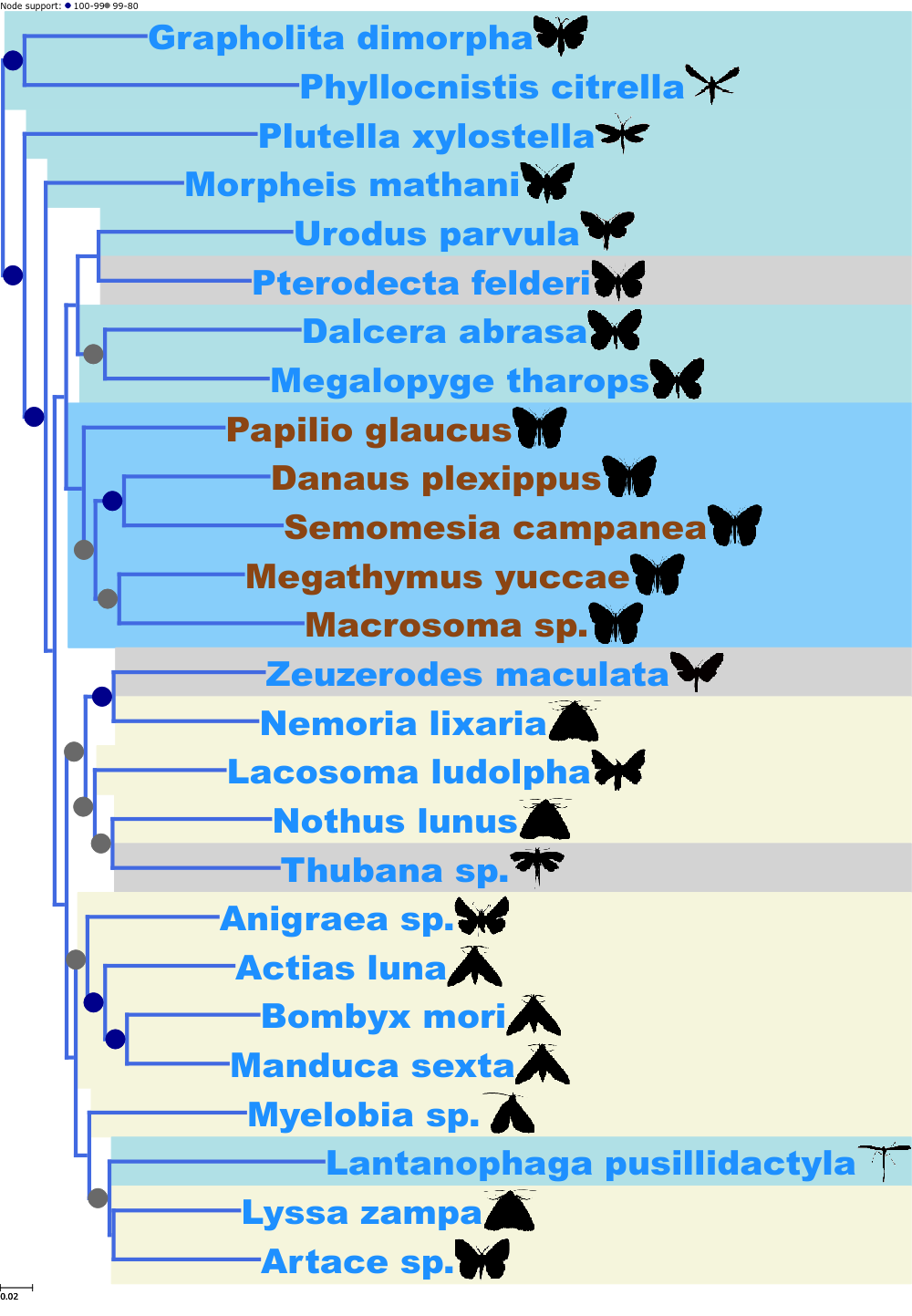

Supplement: S1 Results — A results archive produced by ReproPhylo, containing the serialized Project, input and output files, scripts and an HTML report. http://dx.doi.org/10.6084/m9.figshare.1409488 (ZIP) [file pcbi.1004447.s004.zip › report/files/357421429738848.49_entropy_0.30_0.00_loci_100_to_299.png]

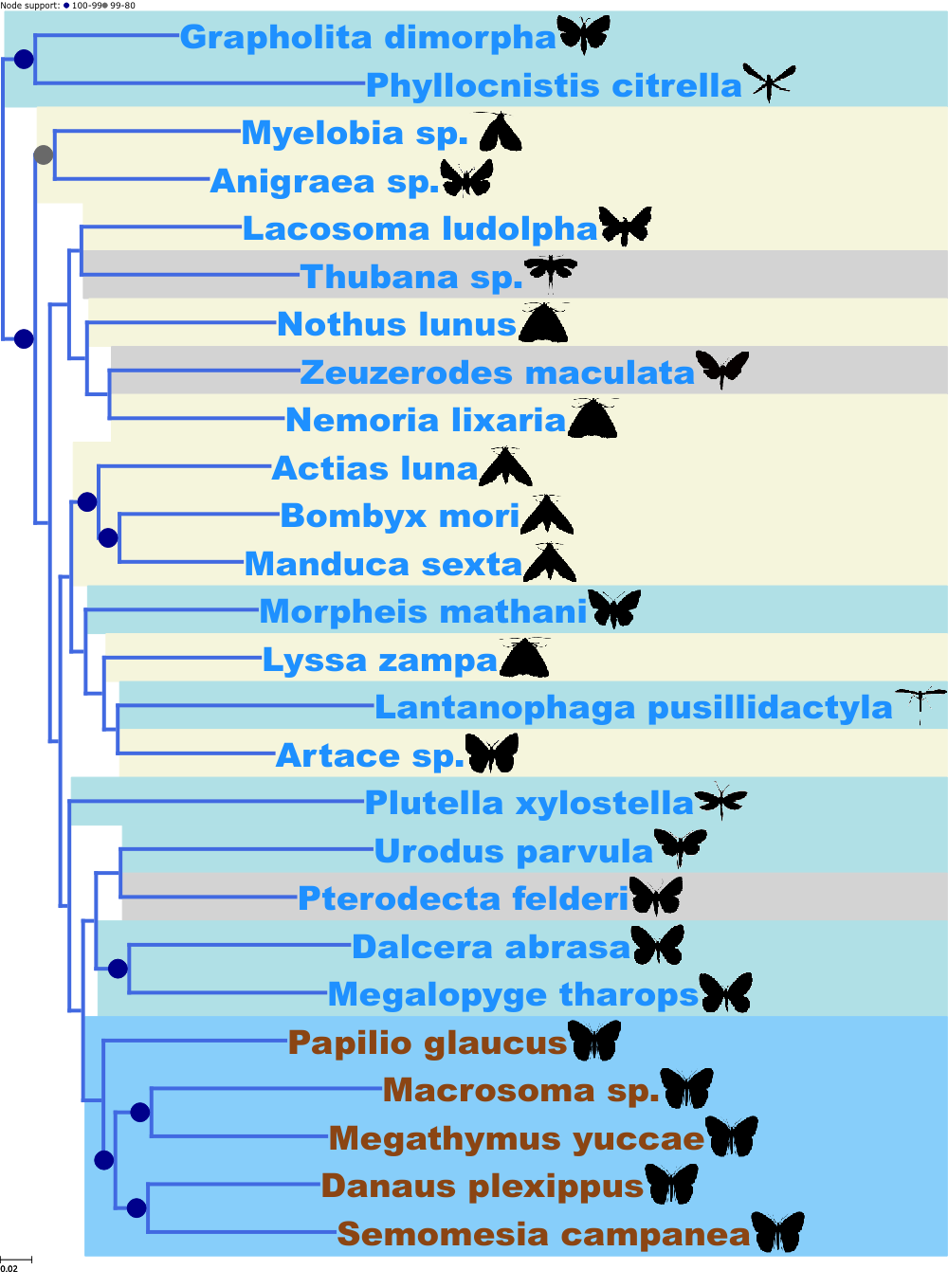

Supplement: S1 Results — A results archive produced by ReproPhylo, containing the serialized Project, input and output files, scripts and an HTML report. http://dx.doi.org/10.6084/m9.figshare.1409488 (ZIP) [file pcbi.1004447.s004.zip › report/files/357421429738848.49_entropy_0.48_0.00_loci_50_to_249.png]

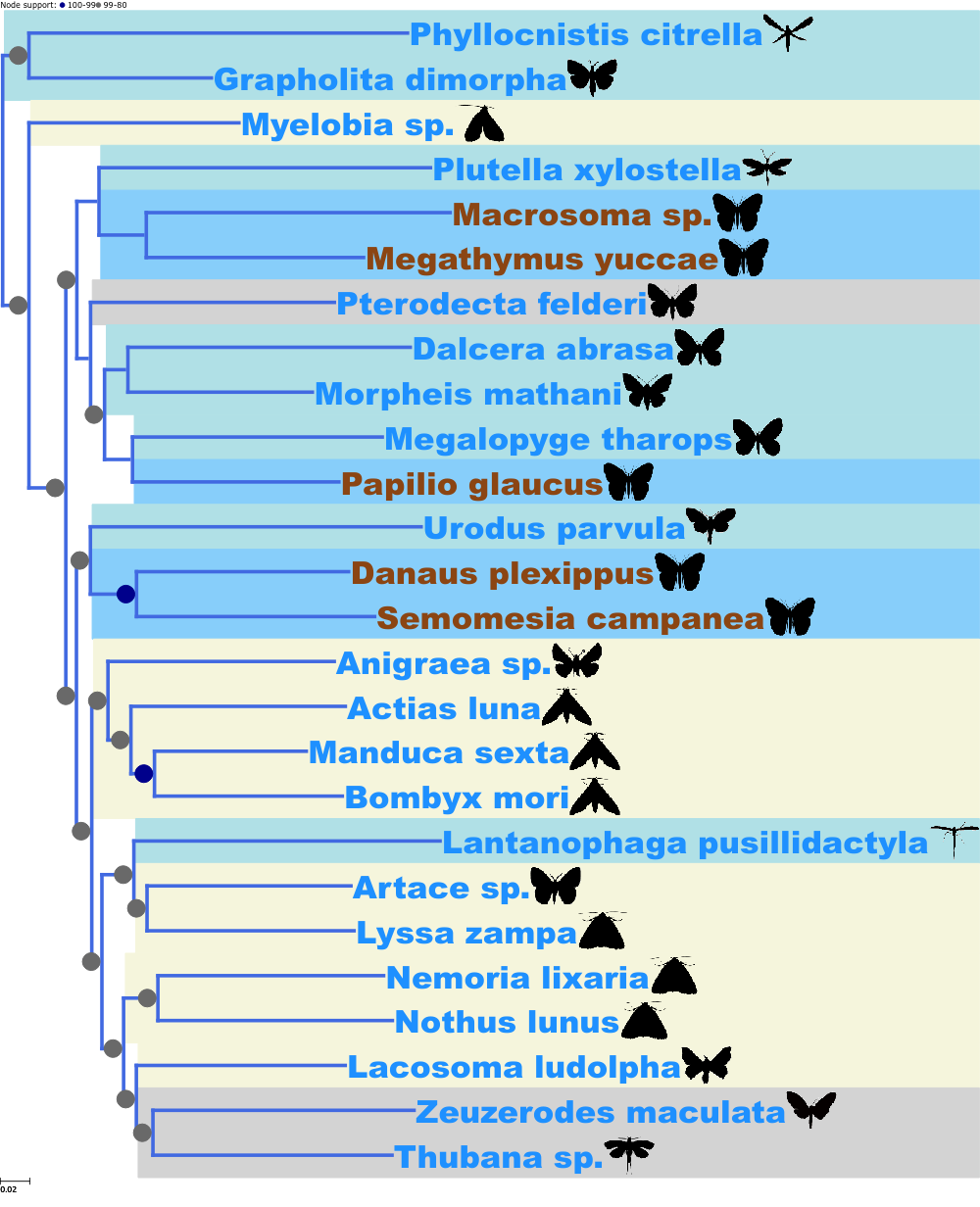

Supplement: S1 Results — A results archive produced by ReproPhylo, containing the serialized Project, input and output files, scripts and an HTML report. http://dx.doi.org/10.6084/m9.figshare.1409488 (ZIP) [file pcbi.1004447.s004.zip › report/files/357421429738848.49_entropy_1.14_0.00_loci_0_to_199.png]
