# Supplementary material for: ReproPhylo: An Environment for Reproducible Phylogenomics
Source: PLoS Comput Biol. 2015 Sep 3;11(9):e1004447. doi: 10.1371/journal.pcbi.1004447 (PMC4559436; doi:10.1371/journal.pcbi.1004447)
Supplement: S1 Methods — The code snippets in this supplementary file are those associated with the numbered steps in the workflow illustrated in Fig 1. http://dx.doi.org/10.6084/m9.figshare.1502477. (PDF) [file pcbi.1004447.s002.pdf]

Basic usage example

June 29, 2015

In [1]:
```
from reprophylo import *

# 1: Report loci in GenBank file
list_loci_in_genbank('genbank_file.gb', 'loci.csv')

# 2: Read the loci file
pj = Project('loci_edited.csv', pickle='project.pkl')

# 3: Read the GenBank file
pj.read_embl_genbank(['genbank_file.gb'])

# 4: Read fasta files
pj.read_denovo(['fasta1.fas', 'fasta2.fas'], 'dna')

# 5: Write a metadata table
pj.write('metadata.csv', format='csv')

# 6: Read edited metadata table
pj.correct_metadata_from_csv('metadata_edited.csv')

# 7: Write an updated GenBank file - NOT a required step
# will only include genes that belong to loci
# in the edited loci file
pj.write('records.gb', format='genbank')

# 8: Print sequence statistics plots
%matplotlib inline
pj.extract_by_locus()
pj.report_seq_stats()

# 9: Exclude and filter sequences
pj.filter_by_seq_length('COI', min_length=500, max_length=1200)
pj.exclude(start_from_max=False, COI=['accession1', 'accession2'])

# 10: Align
mafft = AlnConf(pj, method_name='mafftLinsi',
                CDSAlign=True,
                codontable=1,
                program_name='mafft',
                cmd='mafft',
                loci='all',
                cline_args=dict(localpair=True, maxiterate=1000))
pj.align([mafft])
```

```
# 11: Add external alignments
pj.read_alignment('Another_locus.nex',
                  'dna',
                  'CDS',
                  'ND5',
                  format='nexus')


# 12: Trim the alignments
trimal = TrimalConf(pj,
                    method_name='gappyout',
                    program_name='trimal',
                    cmd='default',
                    alns='all',
                    trimal_commands={'gappyout': True})
pj.trim([trimal])

# 13: Print alignment statistics
stats = LociStats(pj, trimmed=True)
stats.sort(parameter = 'entropy')
stats.plot('all_params.png')

# 14: Concatenate loci.
concat = Concatenation('concat1', pj.loci,
                       'sampleid',
                       otu_must_have_all_of=['COI', 'ND5'])
pj.add_concatenation(concat)
pj.make_concatenation_alignments()

# Or see ways to concatenate using LociStats

# 15: Reconstract a tree
raxml_method_concat = RaxmlConf(pj, method_name='fD_fb_combined',
                                program_name='raxmlHPC-PTHREADS-SSE3',
                                preset = 'fD_fb', alns=['concat1'],
                                model='GAMMA', matrix='JTT', threads=4,
                                cline_args={'-N': 1})
pj.tree([raxml_method_concat])

# 16: Make a figure
pj.annotate('./figures',
            'genus', 'Tetilla', #This is the outgourp
            ['organism', 'sampleid'], # These are the labels
            node_bg_meta = 'genus',
            node_bj_colors = {'Tetilla': 'blue'}
            )

# 17: Compare trees
trees = ['concat1@mixed@mixed@fD_fb', # names are automatic
         'concat2@mixed@mixed@fD_fb']# can be seen in pj.trees.keys()

figfile,legend = calc_rf(pj,
                         './matrices',
```

```
                                    rf_type='proportional',
                                    trees=trees)

# 18: Write alignment files
pj.write_alns(id=['organim','sampleid'], format='nexus')
```
