# Supplementary material for: ReproPhylo: An Environment for Reproducible Phylogenomics
Source: PLoS Comput Biol. 2015 Sep 3;11(9):e1004447. doi: 10.1371/journal.pcbi.1004447 (PMC4559436; doi:10.1371/journal.pcbi.1004447)
Supplement: S2 Methods — A static HTML representation of the code that was used to create all the analyses in this study. http://dx.doi.org/10.6084/m9.figshare.1409427 (HTML). Also in nbviewer: http://goo.gl/KzFAvj. [file pcbi.1004447.s003.html]

IPython\_Notebook\_for\_ReproPhylo\_MS


# Analysis for the ReproPhylo manuscript¶

This notebook containts the analysis conducted for the ReproPhylo manuscript. The repository containig it, as well as related files, is at https://github.com/HullUni-bioinformatics. The ReproPhylo pipeline is needed to run it is provided as a Docker image. The manual and development version is in our Git page GitHub page.

# Table of content¶

1 The full unannotates script  
2 The same script, with annotations  
   2.1 Turn a nexus file with PAUP style charset lines into a ReproPhylo Project instance  
   2.2 Read a supplementary table with pandas  
   2.3 Manually assign species to clades  
   2.4 Export all the records to a text file  
   2.5 Making sure all-gap possitions are removed from the alignments  
   2.6 Preparing supermatrices from 200 loci long sliding windows along the entrophy gradient  
      2.6.1 Calculating the statistics  
      2.6.2 Sort the loci according to statistics values  
      2.6.3 Figure 2A  
      2.6.4 Figure S1, Plot a box plot for each parameter and locus  
   2.7 Prepare the Concatenation objects  
   2.8 Reconstruct the trees  
   2.9 Verify the git commit  
   2.10 Plot the trees, Figure 2A 1-6  
   2.11 Robinson Foulds like metrics  
      2.11.1 Figure 2B - Topological  
      2.11.2 Figure 2C - Branch length  
      2.11.3 Figure 2D - Proportional  
   2.12 Prepare zip folder  
   2.13 Add README and commit  
   2.14 Manual commit of some additional files

# 1 The full, unannotated script:¶

The cell bellow contains the full analysis. Default values are not shown.  
An annotated version follows. Default values are explicitly indicated there.

In [ ]:

```
from reprophylo import *


# Read Kawahara_Breinholt_2014 loci and start a Project 
nexus_filename = 'data/Kawahara_Breinholt_2014/'+\
                 'Kawahara_Breinholt_2014_26taxa_465loci_Degen_nt12.nex'
    
pj = pj_from_nexus_w_charset(nexus_filename, 'data', 'dna', 'CDS', project = True,
                             pickle = 'szitenberg_et_al_15_reprophylo',git = True)     

# manually read metadata with pandas from 
# Kawahara_Breinholt_2014 supplementary file
supp3 = 'data/Kawahara_Breinholt_2014/'+\
        'rspb20140970supp3.csv'
    
metadata = pd.read_table(supp3, skiprows=2)

for r in pj.records:
    source = r.features[0]
    original_id = source.qualifiers['original_id'][0]
    record_metadata = metadata.loc[metadata['Sample ID'] == original_id]
    record_metadata = record_metadata.to_dict()for key in record_metadata:                                          # This will add each value in the table
        source.qualifiers[key] = [record_metadata[key].items()[0][1]] 
    source.qualifiers['organism'] = source.qualifiers['Taxa'] 
    
# Manually assign clades to taxa:
clades =       {'Papilio glaucus': 'butterfilies',
                'Macrosoma sp.*': 'butterfilies',
                'Megathymus yuccae*': 'butterfilies',
                'Semomesia campanea': 'butterfilies',
                'Danaus plexippus*': 'butterfilies',
                'Phyllocnistis citrella*': 'I',
                'Plutella xylostella*': 'I',
                'Grapholita dimorpha*': 'I',
                'Lantanophaga pusillidactyla*': 'I',
                'Urodus parvula*': 'I',
                'Morpheis mathani*': 'I',
                'Megalopyge tharops*': 'I',
                'Dalcera abrasa*': 'I',
                'Thubana sp.*': 'III',
                'Pterodecta felderi*': 'III',
                'Zeuzerodes maculata*': 'III',
                'Lacosoma ludolpha*': 'IV',
                'Lyssa zampa*': 'IV',
                'Nothus lunus*': 'IV',
                'Nemoria lixaria*': 'IV',
                'Anigraea sp.': 'IV',
                'Bombyx mori*': 'IV',
                'Manduca sexta*': 'IV',
                'Artace sp.*': 'IV',
                'Myelobia sp.*': 'IV',
                'Actias luna*': 'IV'}

pj.add_qualifier_from_source('Taxa')

for taxon in clades:               
    pj.if_this_then_that(taxon, 'Taxa', clades[taxon], 'Clade')
    
# Remove all gap positions
trimal = TrimalConf(pj, method_name='no_trim', trimal_commands={'noallgaps': True}) 
pj.trim([trimal])

# Calculate loci statistics
# (entropy, conservation, gap score, GC content and sequence lengths)
stats = LociStats(pj)

# Sort the loci according to entropy
stats.sort(parameter = 'entropy', reverse = True)

# Plot the loci statistics as boxplots
stats.plot('plots.png', figsize=(15,23),  params='all',
           lable_fsize=20, xtick_fsize=2, ytick_fsize=1,
           boxcolor='salmon',
           whiskercolor='gray',
           capcolor='black',
           mediancolor='white',
           medianline_w=3)

# Make concatenations of 200 loci sliding window of entropy
concatenations = stats.slide_loci('source_original_id',
                                  parameter='entropy',
                                  start=0,length=200,step=50)  

# Add the concatenation objects to the project
for concat in concatenations:
    pj.add_concatenation(concat)
    
# Create the supermatrices
pj.make_concatenation_alignments()

# And collect their names
aln_names = [c.name for c in pj.concatenations]

# Build the trees
raxml = RaxmlConf(pj, alns=aln_names, threads=6)

# reconstruct the trees
pj.tree([raxml])

# Remove stars from species names  
from itertools import chain

for f in chain(*[r.features for r in pj.records]):
    if 'Taxa' in f.qualifiers:
        f.qualifiers['Taxa'][0] = f.qualifiers['Taxa'][0].replace('*','')
        
pj.propagate_metadata()


# Plot the trees
pics = {'Bombycoidea': 'silhouettes/Bombycoidea.png',
        'Calliduloidea': 'silhouettes/Calliduloidea.png',
        'Cossoidea': 'silhouettes/Cossoidea.png',
        'Gelechioidea': 'silhouettes/Gelechioidea.png',
        'Geometroidea': 'silhouettes/Geometroidea.png',
        'Hepialoidea': 'silhouettes/Hepialoidea.png',
        'Papilionoidea': 'silhouettes/Papilionoidea.png',
        'Mimallonoidea': 'silhouettes/Mimallonoidea.png',
        'Zygaenoidea': 'silhouettes/Zygaenoidea.png',
        'Lasiocampoidea': 'silhouettes/Lasiocampoidea.png',
        'Thyridoidea': 'silhouettes/Thyridoidea.png',
        'Pterophoroidea': 'silhouettes/Pterophoroidea.png',
        'Pyraloidea': 'silhouettes/Pyraloidea.png',
        'Noctuoidea': 'silhouettes/Noctuoidea.png',
        'Gracillarioidea': 'silhouettes/Gracillarioidea.png',
        'Yponomeutoidea': 'silhouettes/Yponomeutoidea.png',
        'Urodoidea': 'silhouettes/Urodoidea.png',
        'Tortricoidea': 'silhouettes/Tortricoidea.png'
        
       }

colors = {'Bombycoidea': 'DodgerBlue',
          'Calliduloidea': 'DodgerBlue',
          'Cossoidea': 'DodgerBlue',
          'Gelechioidea': 'DodgerBlue',
          'Geometroidea': 'DodgerBlue',
          'Hepialoidea': 'DodgerBlue',
          'Papilionoidea': 'SaddleBrown',
          'Mimallonoidea': 'DodgerBlue',
          'Zygaenoidea': 'DodgerBlue',
          'Lasiocampoidea': 'DodgerBlue',
          'Thyridoidea': 'DodgerBlue',
          'Pterophoroidea': 'DodgerBlue',
          'Pyraloidea': 'DodgerBlue',
          'Noctuoidea': 'DodgerBlue',
         'Gracillarioidea': 'DodgerBlue',
         'Yponomeutoidea': 'DodgerBlue',
         'Urodoidea': 'DodgerBlue',
         'Tortricoidea': 'DodgerBlue'}
            
bg =  {'butterfilies': 'LightSkyBlue',
       'I': 'PowderBlue',
       'III': 'LightGrey',
       'IV': 'Beige'
      }


supports = { 'DarkBlue' : [100,99],
             'DimGray': [99,80]}
dir_name = "butterfly_figs"

html_name = "%s/figures.html"%dir_name

if not os.path.exists(dir_name):
    os.mkdir(dir_name)

pj.clear_tree_annotations()  

pj.annotate(dir_name, 'mid','mid', ['Taxa'], 
            leaf_node_color_meta='source_Superfamily',
            leaf_label_colors=colors, ftype='Arial Black',
            fsize=40, node_support_dict=supports,
            support_bullet_size=15, pic_meta='source_Superfamily', 
            pic_paths=pics, pic_w=85, pic_h=65, node_bg_meta='Clade',     
            node_bg_color=bg, branch_width=6, branch_color='RoyalBlue',
            scale=2500, html=html_name)

# Calculate pairwise tree distances
if not os.path.exists('rf_figs'):
    os.mkdir('rf_figs')
    
# topological
figfile1, legend = calc_rf(pj, 'rf_figs', rf_type='topology', unrooted_trees=True, trees=trees)
# branch-length
figfile2, legend = calc_rf(pj, 'rf_figs', rf_type='branch-length', unrooted_trees=True, trees=trees)
# topological
figfile3,legend = calc_rf(pj, 'rf_figs', rf_type='proportional', unrooted_trees=True, trees=trees)

# Write a zip file
publish(pj, 'report', 'butterfly_figs/',
        compare_trees=['topology','branch-length','proportional'],
        trees_to_compare=trees)
```

# 2 The same script, with annotations¶

In [1]:

```
from reprophylo import *
```

## 2.1 Turn a nexus file with PAUP style charset lines into a ReproPhylo Project instance¶

Any file format can be read, (see manual).  
If GenBank or EMBL formats are read, the metadata is stored and is usable throughout. See example in use case 1 in the manual.

In [2]:

```
nexus_filename = 'data/Kawahara_Breinholt_2014/'+\
                 'Kawahara_Breinholt_2014_26taxa_465loci_Degen_nt12.nex'

pj = pj_from_nexus_w_charset(nexus_filename,
                             
                             'data',             # path to write intermediate fasta file    
                             
                             'dna',              # Character type ('dna' or 'prot')
                             
                             'CDS',              # Feature type (Any)
                             
                             project = True,     # Will return a Project instance instead of a list
                                                 # of fasta files per partition 
                                                 # if project will save it to this file:
                             pickle = 'szitenberg_et_al_15_reprophylo',
                             
                             git = True)         # Will start and manage repository
```

```
/home/amir/Dropbox/python_modules/rpgit.py:74: UserWarning: Thanks to Stack-Overflow users Shane Geiger and Billy Jin for the git wrappers code
  warnings.warn('Thanks to Stack-Overflow users Shane Geiger and Billy Jin for the git wrappers code')
/home/amir/Dropbox/python_modules/rpgit.py:90: UserWarning: A git repository was created in /home/amir/Dropbox/Szitenberg_et_al_2015_ReproPhylo.
  warnings.warn('A git repository was created in %s.'%repoDir)
/home/amir/Dropbox/python_modules/reprophylo.py:255: UserWarning: The new repository is called szitenberg_et_al_15_reprophylo.
  warnings.warn('The new repository is called %s.'%open(cwd + '/.git/description', 'r').read().rstrip())
DEBUG:Cloud:Log file (/home/amir/.picloud/cloud.log) opened
```

```
1/465 reading EOG69CQC1_1
2/465 reading EOG6M65NR_1
3/465 reading EOG69KFV8_1
4/465 reading EOG6SN1SH_1
5/465 reading EOG654933_1
6/465 reading EOG66147B_1
7/465 reading EOG6PRSVJ_1
8/465 reading EOG6PRSVH_1
9/465 reading EOG680J15_1
10/465 reading EOG6S7JTK_1
11/465 reading EOG60ZR1Z_1
12/465 reading EOG6DBTJG_1
13/465 reading EOG6FXRCN_1
14/465 reading EOG69ZXTF_1
15/465 reading EOG60P4BD_1
16/465 reading EOG6BCDR6_1
17/465 reading EOG64QT4T_1
18/465 reading EOG6NCMGZ_1
19/465 reading EOG66147D_1
20/465 reading EOG67SSJD_1
21/465 reading EOG6K3M19_1
22/465 reading EOG670TN2_1
23/465 reading EOG60P4BJ_1
24/465 reading EOG6DBTK0_1
25/465 reading EOG647FZH_1
26/465 reading EOG6D5327_1
27/465 reading EOG63TZZV_1
28/465 reading EOG6HDSZD_1
29/465 reading EOG64MXX7_1
30/465 reading EOG68WC5N_1
31/465 reading EOG6GTKHJ_1
32/465 reading EOG68SGXD_1
33/465 reading EOG6G7C30_1
34/465 reading EOG6K6G7Z_1
35/465 reading EOG6SXNH7_1
36/465 reading EOG65X80D_1
37/465 reading EOG6NVZQC_1
38/465 reading EOG6MPHV2_1
39/465 reading EOG6SJ5J4_1
40/465 reading EOG6C87X0_1
41/465 reading EOG68KRFS_1
42/465 reading EOG6GHZSR_1
43/465 reading EOG65493R_1
44/465 reading EOG6868HF_1
45/465 reading EOG666VQ4_1
46/465 reading EOG6MSD2X_1
47/465 reading EOG63FH1T_1
48/465 reading EOG698V44_1
49/465 reading EOG6F7NPM_1
50/465 reading EOG676K3N_1
51/465 reading EOG651DV5_1
52/465 reading EOG6KWJXS_1
53/465 reading EOG6894QQ_1
54/465 reading EOG6868GW_1
55/465 reading EOG69PB31_1
56/465 reading EOG6SJ5JV_1
57/465 reading EOG65492X_1
58/465 reading EOG6JDHBD_1
59/465 reading EOG63212K_1
60/465 reading EOG67H5VB_1
61/465 reading EOG6C87X4_1
62/465 reading EOG61C717_1
63/465 reading EOG673PVJ_1
64/465 reading EOG68D102_1
65/465 reading EOG60ZR1T_1
66/465 reading EOG68WC5K_1
67/465 reading EOG63JC89_1
68/465 reading EOG6CJVMH_1
69/465 reading EOG60VVTB_1
70/465 reading EOG698V46_1
71/465 reading EOG6GHZSZ_1
72/465 reading EOG6J6RV9_1
73/465 reading EOG6S4PM1_1
74/465 reading EOG68D104_1
75/465 reading EOG68KRG8_1
76/465 reading EOG6N8R81_1
77/465 reading EOG63BMSV_1
78/465 reading EOG69KFV1_1
79/465 reading EOG6H1B0Q_1
80/465 reading EOG6CJVMF_1
81/465 reading EOG6C2HF4_1
82/465 reading EOG6CC44S_1
83/465 reading EOG6FXRCK_1
84/465 reading EOG6M65N3_1
85/465 reading EOG6R2400_1
86/465 reading EOG6BK57N_1
87/465 reading EOG68KRG4_1
88/465 reading EOG61C70T_1
89/465 reading EOG6BK575_1
90/465 reading EOG6R506X_1
91/465 reading EOG6SJ5JS_1
92/465 reading EOG6JT09G_1
93/465 reading EOG6HQDPG_1
94/465 reading EOG6H4677_1
95/465 reading EOG63212W_1
96/465 reading EOG6F7NPJ_1
97/465 reading EOG695ZW4_1
98/465 reading EOG61NTQM_1
99/465 reading EOG6SJ5J3_1
100/465 reading EOG6NZTZ3_1
101/465 reading EOG6J3WM7_1
102/465 reading EOG680J16_1
103/465 reading EOG68SGXM_1
104/465 reading EOG60K843_1
105/465 reading EOG65X811_1
106/465 reading EOG602WWN_1
107/465 reading EOG6RNBCT_1
108/465 reading EOG6M39DN_1
109/465 reading EOG63BMSB_1
110/465 reading EOG6RFKXS_1
111/465 reading EOG6RXZ4G_1
112/465 reading EOG6B8JHB_1
113/465 reading EOG67SSJJ_1
114/465 reading EOG654930_1
115/465 reading EOG6PVP2X_1
116/465 reading EOG69GKKW_1
117/465 reading EOG6RXZ3G_1
118/465 reading EOG6NZTXP_1
119/465 reading EOG602WX4_1
120/465 reading EOG6KH2ZX_1
121/465 reading EOG61JZGH_1
122/465 reading EOG6QFWHZ_1
123/465 reading EOG6SJ5J9_1
124/465 reading EOG6STS8H_1
125/465 reading EOG68PMPX_1
126/465 reading EOG6RFKXB_1
127/465 reading EOG62BXD6_1
128/465 reading EOG647FXX_1
129/465 reading EOG6B8JHP_1
130/465 reading EOG6F7NPN_1
131/465 reading EOG6GHZSG_1
132/465 reading EOG6P8FN9_1
133/465 reading EOG6S1TBX_1
134/465 reading EOG6R506R_1
135/465 reading EOG6DFPRT_1
136/465 reading EOG65TCRW_1
137/465 reading EOG6HX54P_1
138/465 reading EOG60VVST_1
139/465 reading EOG65HS25_1
140/465 reading EOG6FFD5C_1
141/465 reading EOG6PNXM9_1
142/465 reading EOG6R506D_1
143/465 reading EOG670TNG_1
144/465 reading EOG6F4SFZ_1
145/465 reading EOG6Q8524_1
146/465 reading EOG6QVCGP_1
147/465 reading EOG669QZR_1
148/465 reading EOG6JT09B_1
149/465 reading EOG65TCRS_1
150/465 reading EOG6CJVMM_1
151/465 reading EOG6K9BH5_1
152/465 reading EOG63N7HX_1
153/465 reading EOG6QRH88_1
154/465 reading EOG6M65N6_1
155/465 reading EOG6JQ426_1
156/465 reading EOG61G385_1
157/465 reading EOG6QRH81_1
158/465 reading EOG6NCMH9_1
159/465 reading EOG6FN4N0_1
160/465 reading EOG65HS1N_1
161/465 reading EOG6CC44T_1
162/465 reading EOG6RJG4W_1
163/465 reading EOG6K9BGH_1
164/465 reading EOG6FR0VW_1
165/465 reading EOG6N8R82_1
166/465 reading EOG6K0QSG_1
167/465 reading EOG6KKZ6X_1
168/465 reading EOG60VVTG_1
169/465 reading EOG6QC198_1
170/465 reading EOG6D26TG_1
171/465 reading EOG6FFD52_1
172/465 reading EOG6QZ7R6_1
173/465 reading EOG6KKZ71_1
174/465 reading EOG634W9K_1
175/465 reading EOG6DNF7N_1
176/465 reading EOG679FBS_1
177/465 reading EOG6BG909_1
178/465 reading EOG63BMSG_1
179/465 reading EOG61RPZF_1
180/465 reading EOG6BVRZ3_1
181/465 reading EOG6MSD3H_1
182/465 reading EOG60CHM6_1
183/465 reading EOG6PC9WG_1
184/465 reading EOG60GCVD_1
185/465 reading EOG6R506Q_1
186/465 reading EOG66MBNX_1
187/465 reading EOG605S4X_1
188/465 reading EOG66HGF2_1
189/465 reading EOG65TCRN_1
190/465 reading EOG608ND4_1
191/465 reading EOG6640GX_1
192/465 reading EOG6SQX12_1
193/465 reading EOG69GKMS_1
194/465 reading EOG6FR0VX_1
195/465 reading EOG6P2Q5K_1
196/465 reading EOG634W9M_1
197/465 reading EOG6B8JHC_1
198/465 reading EOG6QVCGS_1
199/465 reading EOG6Q2DJZ_1
200/465 reading EOG64TPC4_1
201/465 reading EOG6D26TV_1
202/465 reading EOG605S4Z_1
203/465 reading EOG6N04JN_1
204/465 reading EOG6DNF8K_1
205/465 reading EOG68GW71_1
206/465 reading EOG6CC44M_1
207/465 reading EOG63JC8S_1
208/465 reading EOG67WNS9_1
209/465 reading EOG6NS3G8_1
210/465 reading EOG64TPCQ_1
211/465 reading EOG6PNXM6_1
212/465 reading EOG68GW77_1
213/465 reading EOG6NS3GS_1
214/465 reading EOG6229Q2_1
215/465 reading EOG63TZZW_1
216/465 reading EOG6NP76T_1
217/465 reading EOG63JC90_1
218/465 reading EOG67H5V2_1
219/465 reading EOG63R3QZ_1
220/465 reading EOG65QHJ5_1
221/465 reading EOG6MW89D_1
222/465 reading EOG64F6DH_1
223/465 reading EOG6SJ5K0_1
224/465 reading EOG69GKM6_1
225/465 reading EOG66HGFW_1
226/465 reading EOG6MKNM3_1
227/465 reading EOG63V00H_1
228/465 reading EOG6BG90S_1
229/465 reading EOG6NP77P_1
230/465 reading EOG66DM62_1
231/465 reading EOG6JQ41Z_1
232/465 reading EOG6QFWJH_1
233/465 reading EOG69CQBW_1
234/465 reading EOG6N04K4_1
235/465 reading EOG69KFTV_1
236/465 reading EOG6CJVMQ_1
237/465 reading EOG6SBF24_1
238/465 reading EOG6P8FNB_1
239/465 reading EOG62V8KS_1
240/465 reading EOG6DZ204_1
241/465 reading EOG6C5CPH_1
242/465 reading EOG6FFD51_1
243/465 reading EOG6QNN0J_1
244/465 reading EOG6PC9WN_1
245/465 reading EOG6Q8529_1
246/465 reading EOG6KSPP5_1
247/465 reading EOG6G1MMG_1
248/465 reading EOG63XV6Q_1
249/465 reading EOG6D531X_1
250/465 reading EOG61ZFF6_1
251/465 reading EOG6N5W22_1
252/465 reading EOG63V009_1
253/465 reading EOG6PK2CG_1
254/465 reading EOG6NP779_1
255/465 reading EOG6933N9_1
256/465 reading EOG68GW6V_1
257/465 reading EOG66Q6XP_1
258/465 reading EOG6JQ427_1
259/465 reading EOG6C5CNP_1
260/465 reading EOG6R5066_1
261/465 reading EOG6FTW4C_1
262/465 reading EOG6GXFRF_1
263/465 reading EOG65759X_1
264/465 reading EOG6KWJX8_1
265/465 reading EOG6QNN0N_1
266/465 reading EOG641QFN_1
267/465 reading EOG6S1TBT_1
268/465 reading EOG6GB79M_1
269/465 reading EOG65B1K2_1
270/465 reading EOG66WZD1_1
271/465 reading EOG65TCRK_1
272/465 reading EOG6PNXN7_1
273/465 reading EOG6FR0WR_1
274/465 reading EOG6QRH78_1
275/465 reading EOG6P2Q64_1
276/465 reading EOG666VQC_1
277/465 reading EOG6FJ8F2_1
278/465 reading EOG6HHP6H_1
279/465 reading EOG6DJK1Q_1
280/465 reading EOG63R3R5_1
281/465 reading EOG6PC9WF_1
282/465 reading EOG6Q58T0_1
283/465 reading EOG641QGH_1
284/465 reading EOG6933N7_1
285/465 reading EOG69S6BG_1
286/465 reading EOG64XJM7_1
287/465 reading EOG66Q6XR_1
288/465 reading EOG6PK2CM_1
289/465 reading EOG6NS3FV_1
290/465 reading EOG65MN9B_1
291/465 reading EOG641QFW_1
292/465 reading EOG63R3R9_1
293/465 reading EOG6DZ1ZZ_1
294/465 reading EOG6N04J6_1
295/465 reading EOG6CRM3G_1
296/465 reading EOG6GMV1P_1
297/465 reading EOG6S4PKX_1
298/465 reading EOG65MN9F_1
299/465 reading EOG6NP773_1
300/465 reading EOG6K6G7S_1
301/465 reading EOG6DZ201_1
302/465 reading EOG6BZN62_1
303/465 reading EOG68SGZ1_1
304/465 reading EOG69PB2Z_1
305/465 reading EOG6SXNGX_1
306/465 reading EOG6QZ7Q8_1
307/465 reading EOG6DR9GP_1
308/465 reading EOG6QFWJ6_1
309/465 reading EOG6QC18P_1
310/465 reading EOG6CG0CT_1
311/465 reading EOG6HHP6B_1
312/465 reading EOG6PK2CQ_1
313/465 reading EOG67D9KJ_1
314/465 reading EOG6JWVJQ_1
315/465 reading EOG6F1X6H_1
316/465 reading EOG6PG64S_1
317/465 reading EOG605S4Q_1
318/465 reading EOG6R7VF9_1
319/465 reading EOG6CZBM5_1
320/465 reading EOG6JT092_1
321/465 reading EOG6B5P96_1
322/465 reading EOG63R3RK_1
323/465 reading EOG64F6DK_1
324/465 reading EOG6FJ8D6_1
325/465 reading EOG6QZ7QX_1
326/465 reading EOG6F1X6M_1
327/465 reading EOG6001NN_1
328/465 reading EOG62V8KH_1
329/465 reading EOG695ZWC_1
330/465 reading EOG67PX9B_1
331/465 reading EOG6SBF2V_1
332/465 reading EOG6P8FNH_1
333/465 reading EOG6KSPQ0_1
334/465 reading EOG6GMV1H_1
335/465 reading EOG63R3RC_1
336/465 reading EOG6907D7_1
337/465 reading EOG6JWVHT_1
338/465 reading EOG68SGX8_1
339/465 reading EOG6HDSZ9_1
340/465 reading EOG6BK57S_1
341/465 reading EOG60K83N_1
342/465 reading EOG6SXNHD_1
343/465 reading EOG6KD6Q9_1
344/465 reading EOG68GW6Q_1
345/465 reading EOG6Q852G_1
346/465 reading EOG676K44_1
347/465 reading EOG6FN4NZ_1
348/465 reading EOG6Q58ST_1
349/465 reading EOG6933NR_1
350/465 reading EOG62JNVR_1
351/465 reading EOG64MXWZ_1
352/465 reading EOG6S1TC2_1
353/465 reading EOG6HT8WD_1
354/465 reading EOG63FH1F_1
355/465 reading EOG6PC9X3_1
356/465 reading EOG6Q58SW_1
357/465 reading EOG63N7HF_1
358/465 reading EOG6CRM3H_1
359/465 reading EOG6PRSVP_1
360/465 reading EOG6R5069_1
361/465 reading EOG6CC453_1
362/465 reading EOG6001PC_1
363/465 reading EOG63BMS7_1
364/465 reading EOG676K40_1
365/465 reading EOG64MXWK_1
366/465 reading EOG6N30SN_1
367/465 reading EOG6575BP_1
368/465 reading EOG67D9M5_1
369/465 reading EOG615GJK_1
370/465 reading EOG6RBQP0_1
371/465 reading EOG68KRFQ_1
372/465 reading EOG6DR9GZ_1
373/465 reading EOG6Q2DKD_1
374/465 reading EOG6255XM_1
375/465 reading EOG64BB5X_1
376/465 reading EOG6PG646_1
377/465 reading EOG62FSN7_1
378/465 reading EOG6H72G0_1
379/465 reading EOG6PZJB1_1
380/465 reading EOG64MXWG_1
381/465 reading EOG65QHHP_1
382/465 reading EOG6C87WM_1
383/465 reading EOG698V4R_1
384/465 reading EOG615GHV_1
385/465 reading EOG6FFD5G_1
386/465 reading EOG66WZCQ_1
387/465 reading EOG698V4H_1
388/465 reading EOG6NVZQ8_1
389/465 reading EOG6001NK_1
390/465 reading EOG6JDHB2_1
391/465 reading EOG62BXCX_1
392/465 reading EOG60K833_1
393/465 reading EOG62BXCZ_1
394/465 reading EOG6CG0D0_1
395/465 reading EOG6JM7SX_1
396/465 reading EOG69KFVF_1
397/465 reading EOG615GHP_1
398/465 reading EOG6KH2ZR_1
399/465 reading EOG683D8Q_1
400/465 reading EOG6PRSVM_1
401/465 reading EOG64J2NS_1
402/465 reading EOG66HGF5_1
403/465 reading EOG6DFPSF_1
404/465 reading EOG60P4BZ_1
405/465 reading EOG66MBPR_1
406/465 reading EOG6DFPSD_1
407/465 reading EOG67D9KC_1
408/465 reading EOG6HHP5S_1
409/465 reading EOG64BB5N_1
410/465 reading EOG6STS7W_1
411/465 reading EOG6DBTJX_1
412/465 reading EOG6FXRC9_1
413/465 reading EOG6DBTHZ_1
414/465 reading EOG6KH2ZD_1
415/465 reading EOG6STS87_1
416/465 reading EOG65TCS9_1
417/465 reading EOG67D9KV_1
418/465 reading EOG6SXNHG_1
419/465 reading EOG6255XS_1
420/465 reading EOG65MN8T_1
421/465 reading EOG6GF3JF_1
422/465 reading EOG66Q6X8_1
423/465 reading EOG6KSPPS_1
424/465 reading EOG61RPZ9_1
425/465 reading EOG637RJP_1
426/465 reading EOG66MBP7_1
427/465 reading EOG6J3WMR_1
428/465 reading EOG6B2T20_1
429/465 reading EOG6QJRRQ_1
430/465 reading EOG6GB79H_1
431/465 reading EOG6JDHBG_1
432/465 reading EOG6Q852B_1
433/465 reading EOG676K3Q_1
434/465 reading EOG6RV2WT_1
435/465 reading EOG6DV5QM_1
436/465 reading EOG6GTKHQ_1
437/465 reading EOG6FR0W5_1
438/465 reading EOG6NVZPZ_1
439/465 reading EOG6K0QRT_1
440/465 reading EOG65DWSM_1
441/465 reading EOG6CC452_1
442/465 reading EOG6G4GTX_1
443/465 reading EOG64BB5H_1
444/465 reading EOG64J2NC_1
445/465 reading EOG61G37F_1
446/465 reading EOG6H72G3_1
447/465 reading EOG6SBF2B_1
448/465 reading EOG695ZW1_1
449/465 reading EOG68WC58_1
450/465 reading EOG6CG0CX_1
451/465 reading EOG6FFD5V_1
452/465 reading EOG6G4GTT_1
453/465 reading EOG6K3M11_1
454/465 reading EOG651DTX_1
455/465 reading EOG60VVT3_1
456/465 reading EOG62NJ41_1
457/465 reading EOG66147J_1
458/465 reading EOG63FH1C_1
459/465 reading EOG6GXFR5_1
460/465 reading EOG61C70W_1
461/465 reading EOG6DV5QK_1
462/465 reading EOG6HQDPF_1
463/465 reading EOG6NVZPX_1
464/465 reading EOG6GXFR3_1
465/465 reading EOG6KPTG0_1
```

```
/home/amir/Dropbox/python_modules/reprophylo.py:1216: UserWarning: To get translations, add a feature manually
  warnings.warn("To get translations, add a feature manually")
```

In [6]:

```
pickle_pj(pj, 'szitenberg_et_al_15_reprophylo')
```

Out[6]:

```
'szitenberg_et_al_15_reprophylo'
```

If the keyword `project` is False (default), the function returns a list of fasta file names and a list of Loci objects.  
This is usefull when the loci are not all the same character type or feature type.

If `project` == False, the usage is:

```
filenames, loci_list = make_fasta_files_from_nexus(nexus_filename,
                                                   'data', 'dna', 'CDS')
pj = Project(loci_list)

for f in filenames:
    locus_name = f.split('/')[-1].split('.')[0]
    pj.read_alignment(f, 'dna','CDS',locus_name)
```

This takes a while (for both project=True and False) because a few things are happening at the background.
It is worth while for downstream steps

#### Tip:¶

Next time you run, start here by unpickling the file:

```
pj = unpickle_pj('szitenberg_et_al_15_reprophylo')
```

This is much much faster than reading the data. Git will pick up with the same repository.

In [39]:

```
#pj = unpickle_pj('szitenberg_et_al_15_reprophylo')
```

## 2.2 Read a supplementary table with pandas¶

In [11]:

```
import pandas as pd


# This reads a supplementary table
# form Kawahara and Breinholt 2014
supp3 = 'data/Kawahara_Breinholt_2014/'+\
        'rspb20140970supp3.csv'
    
metadata = pd.read_table(supp3, skiprows=2)

# This places the info from the table
# in the metadta of the sequence records:

# Iterate all the records (each sequence in each locus)
# and update their metadata based on the supplementary
# table

for r in pj.records:
    source = r.features[0]                                               # This is a Biopython (GenBank)
                                                                         # source feature. Since we read a 
                                                                         # Nexus file, it was generated
                                                                         # by ReproPhylo rather than read.
                                                                         # If a GenBank file is read, the source
                                                                         # and other features are read as well.
                
    original_id = source.qualifiers['original_id'][0]                    # This is the nexus sequence header.
                                                                         # To allow abstraction, the actual
                                                                         # records id is generated by the system
                                                                         # when formats other than GenBank or EMBL
                                                                         # are read
                    
    record_metadata = metadata.loc[metadata['Sample ID'] == original_id] # The 'original_id' is the 'Sample ID'
                                                                         #  in the supplementary table.
                                                                         # This will get the table line for 
                                                                         # the current 'original_id'.
                
    record_metadata = record_metadata.to_dict()                          # This will turn the line into a dictionery 
                                                                         # with column names as keys.
                                                                         # A dictionary is the python equivalent
                                                                         # for the perl array.
    
    for key in record_metadata:                                          # This will add each value in the table
        source.qualifiers[key] = [record_metadata[key].items()[0][1]]    # line to the Biopython feature qualifiers
                                                                         # in the source feature
          
        
    source.qualifiers['organism'] = source.qualifiers['Taxa']            # the 'Taxa' column in the 
                                                                         # supplementary contains
                                                                         # the 'organism' sensu GenBank
        


        
pickle_pj(pj, 'szitenberg_et_al_15_reprophylo')
```

```
/usr/local/lib/python2.7/dist-packages/pandas/io/excel.py:626: UserWarning: Installed openpyxl is not supported at this time. Use >=1.6.1 and <2.0.0.
  .format(openpyxl_compat.start_ver, openpyxl_compat.stop_ver))
```

Out[11]:

```
'szitenberg_et_al_15_reprophylo'
```

## 2.3 Manually assign species to clades¶

In [12]:

```
clades =       {'Papilio glaucus': 'butterfilies',    # This is a manual assignment of the 'organism'
                'Macrosoma sp.*': 'butterfilies',     # values to lepidopteran clades sensu
                'Megathymus yuccae*': 'butterfilies', # Kawahara and Breinholt 2014.
                'Semomesia campanea': 'butterfilies',
                'Danaus plexippus*': 'butterfilies',
                'Phyllocnistis citrella*': 'I',
                'Plutella xylostella*': 'I',
                'Grapholita dimorpha*': 'I',
                'Lantanophaga pusillidactyla*': 'I',
                'Urodus parvula*': 'I',
                'Morpheis mathani*': 'I',
                'Megalopyge tharops*': 'I',
                'Dalcera abrasa*': 'I',
                'Thubana sp.*': 'III',
                'Pterodecta felderi*': 'III',
                'Zeuzerodes maculata*': 'III',
                'Lacosoma ludolpha*': 'IV',
                'Lyssa zampa*': 'IV',
                'Nothus lunus*': 'IV',
                'Nemoria lixaria*': 'IV',
                'Anigraea sp.': 'IV',
                'Bombyx mori*': 'IV',
                'Manduca sexta*': 'IV',
                'Artace sp.*': 'IV',
                'Myelobia sp.*': 'IV',
                'Actias luna*': 'IV'}

pj.add_qualifier_from_source('Taxa')  # This copies the 'Taxa' qualifier from the source
                                      # feature to all the other features in the record
                                      # (Biopython SeqFeature. See detailed explanation
                                      # in the ReproPhylo manual). It is not necessary,
                                      # but good practice, because we might want to edit
                                      # it for non-source features and keep the original
                                      # as backup in the source feature.

for taxon in clades:                                            # This will assign a 'Clade' qualifier to each 
    pj.if_this_then_that(taxon, 'Taxa', clades[taxon], 'Clade') # feature in each record based on the 'clades'
                                                                # dictionary, manually created above.


# If we were doing this in a project that contains trees,
# the following line makes sure the trees
# are informed of the changes to the metadata:

pj.propagate_metadata()
```

## 2.4 Export all the records to a text file¶

A text file is not required for downstream process, but can be written for insepction. The default format is GenBank. Other complete representations of the data are the EMBL and CSV formats. All other formats can be chosen as well, but they will not represent the metadta.

In [13]:

```
pj.write('data/sequence_and_metadata.gb') # GenBank
pj.write('data/sequence_and_metadata.csv', format='csv') # spreadsheet
```

### Example GenBank formated record¶

```
LOCUS       denovo0                 1357 bp    DNA              UNK 01-JAN-1980
DEFINITION  .
ACCESSION   denovo0
VERSION     denovo0
KEYWORDS    .
SOURCE      .
  ORGANISM  .
            .
FEATURES             Location/Qualifiers                   # These are annotations and not a part of the output
     source          1..1357                               # ==================================================
                     /Sample ID="Bmoricds"                 # From the supplementary table
                     /Minimum contig size=87               # From the supplementary table
                     /Superfamily="Bombycoidea"            # From the supplementary table
                     /Family="Bombycidae"                  # From the supplementary table
                     /#taxa Per lane="nan"                 # From the supplementary table
                     /Locality="nan"                       # From the supplementary table
                     /Taxa="Bombyx mori"                   # From the supplementary table
                     /Collector="nan"                      # From the supplementary table
                     /Genbank SRA accession number="nan"   # From the supplementary table
                     /Maximum contig size=56289            # From the supplementary table
                     /N50=1698                             # From the supplementary table
                     /Collection date="nan"                # From the supplementary table
                     /feature_id="denovo0_source"          # SYSTEM GENERATED
                     /Collection country/region="nan"      # From the supplementary table
                     /original_id="Bmoricds"               # NEXUS FILE SEQUENCE HEADER
                     /original_desc                        # Only when fasta or stockholm are read
                     /# of contigs=14623                   # From the supplementary table
                     /Mean contig size=1224                # From the supplementary table
                     /Total bp=17891307                    # From the supplementary table
                     /Data type="Genomic gene set"         # From the supplementary table
                     /organism="Bombyx mori"               # CREATED ABOVE
     CDS             1..1357                               #
                     /Taxa="Bombyx mori"                   # CREATED ABOVE     
                     /Clade="IV"                           # CREATED ABOVE
                     /nuc_degen_prop="0.0707442888725"     # SYSTEM GENERATED
                     /feature_id="denovo0_f0"              # SYSTEM GENERATED
                     /GC_content="43.0361090641"           # SYSTEM GENERATED
                     /gene="EOG69CQC1_1"                   # THE SET NAME FROM THE CHARSET COMMAND IN THE NEXUS FILE
ORIGIN
        1 cagtgcaagg gtattaytaa ggccgacagt gtccgcgaag taacacgcat ccggtgcagt
       61 aaytmgatgc ccmgg[...]                            # Truncated for presentation
//
```

## 2.5 Making sure all-gap positions are removed from the read alignments¶

There is no sequence alignment step in this analysis because we read aligned sequences. To learn about sequene alignmnet with MAFFT or MUSCLE in ReproPhylo, see the case studies in the manual. The syntax is similar to that of the trimming step which will be done next.

Alignmnet trimming is done with TrimAl, which allows automated heuristic approaches or manual approaches. Here we use it only to remove all gaps positions because we want to keep the alignmnet as it was used in the original publication. See the manual for more options.

In [14]:

```
# make a TrimAl configuration object

trimal = TrimalConf(pj,                                    # The Project instance
                    method_name='no_trim',                 # Any unique string
                    trimal_commands={'noallgaps': True})   # Any TrimAl command line argument
                                                           # TrimAl Flags receive True or False
    
# Every Conf object gets a unique generated ID, even
# if the method name was used in this Project.
# If an existing method name
# is reused, its results will be overwritten, and
# it will be distinguised from previous versions by
# the system ID.
```

```
trimal -in 861921429737580.9_EOG6PRSVJ_1@ReadDirectly.fasta -noallgaps
trimal -in 861921429737580.9_EOG6FJ8F2_1@ReadDirectly.fasta -noallgaps
trimal -in 861921429737580.9_EOG65MN9F_1@ReadDirectly.fasta -noallgaps
trimal -in 861921429737580.9_EOG6Q852B_1@ReadDirectly.fasta -noallgaps
trimal -in 861921429737580.9_EOG66Q6XR_1@ReadDirectly.fasta -noallgaps
trimal -in 861921429737580.9_EOG6PRSVM_1@ReadDirectly.fasta -noallgaps
trimal -in 861921429737580.9_EOG64MXWK_1@ReadDirectly.fasta -noallgaps
trimal -in 861921429737580.9_EOG60K83N_1@ReadDirectly.fasta -noallgaps
trimal -in 861921429737580.9_EOG6933NR_1@ReadDirectly.fasta -noallgaps
trimal -in 861921429737580.9_EOG6QRH81_1@ReadDirectly.fasta -noallgaps
trimal -in 861921429737580.9_EOG6K6G7Z_1@ReadDirectly.fasta -noallgaps
trimal -in 861921429737580.9_EOG6R5069_1@ReadDirectly.fasta -noallgaps
trimal -in 861921429737580.9_EOG6STS8H_1@ReadDirectly.fasta -noallgaps
trimal -in 861921429737580.9_EOG698V4R_1@ReadDirectly.fasta -noallgaps
trimal -in 861921429737580.9_EOG64TPCQ_1@ReadDirectly.fasta -noallgaps
trimal -in 861921429737580.9_EOG63BMS7_1@ReadDirectly.fasta -noallgaps
trimal -in 861921429737580.9_EOG68GW77_1@ReadDirectly.fasta -noallgaps
trimal -in 861921429737580.9_EOG6BZN62_1@ReadDirectly.fasta -noallgaps
trimal -in 861921429737580.9_EOG6QZ7QX_1@ReadDirectly.fasta -noallgaps
trimal -in 861921429737580.9_EOG6GTKHJ_1@ReadDirectly.fasta -noallgaps
trimal -in 861921429737580.9_EOG6HHP5S_1@ReadDirectly.fasta -noallgaps
trimal -in 861921429737580.9_EOG6GB79H_1@ReadDirectly.fasta -noallgaps
trimal -in 861921429737580.9_EOG68KRG8_1@ReadDirectly.fasta -noallgaps
trimal -in 861921429737580.9_EOG6MKNM3_1@ReadDirectly.fasta -noallgaps
trimal -in 861921429737580.9_EOG6FXRCN_1@ReadDirectly.fasta -noallgaps
trimal -in 861921429737580.9_EOG6K0QSG_1@ReadDirectly.fasta -noallgaps
trimal -in 861921429737580.9_EOG63FH1T_1@ReadDirectly.fasta -noallgaps
trimal -in 861921429737580.9_EOG6DJK1Q_1@ReadDirectly.fasta -noallgaps
trimal -in 861921429737580.9_EOG698V4H_1@ReadDirectly.fasta -noallgaps
trimal -in 861921429737580.9_EOG6JWVHT_1@ReadDirectly.fasta -noallgaps
trimal -in 861921429737580.9_EOG65X80D_1@ReadDirectly.fasta -noallgaps
trimal -in 861921429737580.9_EOG61JZGH_1@ReadDirectly.fasta -noallgaps
trimal -in 861921429737580.9_EOG60VVST_1@ReadDirectly.fasta -noallgaps
trimal -in 861921429737580.9_EOG6QRH78_1@ReadDirectly.fasta -noallgaps
trimal -in 861921429737580.9_EOG6PZJB1_1@ReadDirectly.fasta -noallgaps
trimal -in 861921429737580.9_EOG6NS3G8_1@ReadDirectly.fasta -noallgaps
trimal -in 861921429737580.9_EOG65TCRN_1@ReadDirectly.fasta -noallgaps
trimal -in 861921429737580.9_EOG63R3RC_1@ReadDirectly.fasta -noallgaps
trimal -in 861921429737580.9_EOG6C87X4_1@ReadDirectly.fasta -noallgaps
trimal -in 861921429737580.9_EOG64TPC4_1@ReadDirectly.fasta -noallgaps
trimal -in 861921429737580.9_EOG64MXWG_1@ReadDirectly.fasta -noallgaps
trimal -in 861921429737580.9_EOG61G385_1@ReadDirectly.fasta -noallgaps
trimal -in 861921429737580.9_EOG6NVZQ8_1@ReadDirectly.fasta -noallgaps
trimal -in 861921429737580.9_EOG6N04JN_1@ReadDirectly.fasta -noallgaps
trimal -in 861921429737580.9_EOG67SSJJ_1@ReadDirectly.fasta -noallgaps
trimal -in 861921429737580.9_EOG62NJ41_1@ReadDirectly.fasta -noallgaps
trimal -in 861921429737580.9_EOG68GW71_1@ReadDirectly.fasta -noallgaps
trimal -in 861921429737580.9_EOG6Q58ST_1@ReadDirectly.fasta -noallgaps
trimal -in 861921429737580.9_EOG6FXRCK_1@ReadDirectly.fasta -noallgaps
trimal -in 861921429737580.9_EOG6PVP2X_1@ReadDirectly.fasta -noallgaps
trimal -in 861921429737580.9_EOG676K3Q_1@ReadDirectly.fasta -noallgaps
trimal -in 861921429737580.9_EOG6DBTK0_1@ReadDirectly.fasta -noallgaps
trimal -in 861921429737580.9_EOG6KH2ZR_1@ReadDirectly.fasta -noallgaps
trimal -in 861921429737580.9_EOG66HGF2_1@ReadDirectly.fasta -noallgaps
trimal -in 861921429737580.9_EOG64MXX7_1@ReadDirectly.fasta -noallgaps
trimal -in 861921429737580.9_EOG6F1X6H_1@ReadDirectly.fasta -noallgaps
trimal -in 861921429737580.9_EOG66147D_1@ReadDirectly.fasta -noallgaps
trimal -in 861921429737580.9_EOG6FFD5V_1@ReadDirectly.fasta -noallgaps
trimal -in 861921429737580.9_EOG6RXZ3G_1@ReadDirectly.fasta -noallgaps
trimal -in 861921429737580.9_EOG6C5CPH_1@ReadDirectly.fasta -noallgaps
trimal -in 861921429737580.9_EOG6PC9WG_1@ReadDirectly.fasta -noallgaps
trimal -in 861921429737580.9_EOG6DNF7N_1@ReadDirectly.fasta -noallgaps
trimal -in 861921429737580.9_EOG6GHZSR_1@ReadDirectly.fasta -noallgaps
trimal -in 861921429737580.9_EOG6M65N6_1@ReadDirectly.fasta -noallgaps
trimal -in 861921429737580.9_EOG62V8KH_1@ReadDirectly.fasta -noallgaps
trimal -in 861921429737580.9_EOG6DNF8K_1@ReadDirectly.fasta -noallgaps
trimal -in 861921429737580.9_EOG6QVCGS_1@ReadDirectly.fasta -noallgaps
trimal -in 861921429737580.9_EOG69GKKW_1@ReadDirectly.fasta -noallgaps
trimal -in 861921429737580.9_EOG6FFD5G_1@ReadDirectly.fasta -noallgaps
trimal -in 861921429737580.9_EOG64J2NC_1@ReadDirectly.fasta -noallgaps
trimal -in 861921429737580.9_EOG6R5066_1@ReadDirectly.fasta -noallgaps
trimal -in 861921429737580.9_EOG66WZD1_1@ReadDirectly.fasta -noallgaps
trimal -in 861921429737580.9_EOG6GHZSZ_1@ReadDirectly.fasta -noallgaps
trimal -in 861921429737580.9_EOG69CQBW_1@ReadDirectly.fasta -noallgaps
trimal -in 861921429737580.9_EOG63JC90_1@ReadDirectly.fasta -noallgaps
trimal -in 861921429737580.9_EOG634W9M_1@ReadDirectly.fasta -noallgaps
trimal -in 861921429737580.9_EOG6255XM_1@ReadDirectly.fasta -noallgaps
trimal -in 861921429737580.9_EOG6BK57S_1@ReadDirectly.fasta -noallgaps
trimal -in 861921429737580.9_EOG68WC5N_1@ReadDirectly.fasta -noallgaps
trimal -in 861921429737580.9_EOG68D104_1@ReadDirectly.fasta -noallgaps
trimal -in 861921429737580.9_EOG65B1K2_1@ReadDirectly.fasta -noallgaps
trimal -in 861921429737580.9_EOG6SXNH7_1@ReadDirectly.fasta -noallgaps
trimal -in 861921429737580.9_EOG63JC8S_1@ReadDirectly.fasta -noallgaps
trimal -in 861921429737580.9_EOG6KH2ZX_1@ReadDirectly.fasta -noallgaps
trimal -in 861921429737580.9_EOG6R506R_1@ReadDirectly.fasta -noallgaps
trimal -in 861921429737580.9_EOG6DR9GZ_1@ReadDirectly.fasta -noallgaps
trimal -in 861921429737580.9_EOG6N04K4_1@ReadDirectly.fasta -noallgaps
trimal -in 861921429737580.9_EOG63FH1C_1@ReadDirectly.fasta -noallgaps
trimal -in 861921429737580.9_EOG6001NN_1@ReadDirectly.fasta -noallgaps
trimal -in 861921429737580.9_EOG6FR0WR_1@ReadDirectly.fasta -noallgaps
trimal -in 861921429737580.9_EOG6GXFR3_1@ReadDirectly.fasta -noallgaps
trimal -in 861921429737580.9_EOG63V00H_1@ReadDirectly.fasta -noallgaps
trimal -in 861921429737580.9_EOG6JQ427_1@ReadDirectly.fasta -noallgaps
trimal -in 861921429737580.9_EOG6RBQP0_1@ReadDirectly.fasta -noallgaps
trimal -in 861921429737580.9_EOG6N04J6_1@ReadDirectly.fasta -noallgaps
trimal -in 861921429737580.9_EOG6K3M11_1@ReadDirectly.fasta -noallgaps
trimal -in 861921429737580.9_EOG61C70T_1@ReadDirectly.fasta -noallgaps
trimal -in 861921429737580.9_EOG6H1B0Q_1@ReadDirectly.fasta -noallgaps
trimal -in 861921429737580.9_EOG63JC89_1@ReadDirectly.fasta -noallgaps
trimal -in 861921429737580.9_EOG64BB5N_1@ReadDirectly.fasta -noallgaps
trimal -in 861921429737580.9_EOG6CC44T_1@ReadDirectly.fasta -noallgaps
trimal -in 861921429737580.9_EOG68SGZ1_1@ReadDirectly.fasta -noallgaps
trimal -in 861921429737580.9_EOG69CQC1_1@ReadDirectly.fasta -noallgaps
trimal -in 861921429737580.9_EOG65HS1N_1@ReadDirectly.fasta -noallgaps
trimal -in 861921429737580.9_EOG69PB31_1@ReadDirectly.fasta -noallgaps
trimal -in 861921429737580.9_EOG6CC453_1@ReadDirectly.fasta -noallgaps
trimal -in 861921429737580.9_EOG6MSD3H_1@ReadDirectly.fasta -noallgaps
trimal -in 861921429737580.9_EOG61G37F_1@ReadDirectly.fasta -noallgaps
trimal -in 861921429737580.9_EOG62JNVR_1@ReadDirectly.fasta -noallgaps
trimal -in 861921429737580.9_EOG6HHP6B_1@ReadDirectly.fasta -noallgaps
trimal -in 861921429737580.9_EOG6N30SN_1@ReadDirectly.fasta -noallgaps
trimal -in 861921429737580.9_EOG6KSPP5_1@ReadDirectly.fasta -noallgaps
trimal -in 861921429737580.9_EOG698V46_1@ReadDirectly.fasta -noallgaps
trimal -in 861921429737580.9_EOG68SGXM_1@ReadDirectly.fasta -noallgaps
trimal -in 861921429737580.9_EOG6PK2CQ_1@ReadDirectly.fasta -noallgaps
trimal -in 861921429737580.9_EOG6KH2ZD_1@ReadDirectly.fasta -noallgaps
trimal -in 861921429737580.9_EOG6DBTJG_1@ReadDirectly.fasta -noallgaps
trimal -in 861921429737580.9_EOG6F7NPJ_1@ReadDirectly.fasta -noallgaps
trimal -in 861921429737580.9_EOG6CRM3H_1@ReadDirectly.fasta -noallgaps
trimal -in 861921429737580.9_EOG6FR0VX_1@ReadDirectly.fasta -noallgaps
trimal -in 861921429737580.9_EOG6FR0VW_1@ReadDirectly.fasta -noallgaps
trimal -in 861921429737580.9_EOG605S4Q_1@ReadDirectly.fasta -noallgaps
trimal -in 861921429737580.9_EOG65492X_1@ReadDirectly.fasta -noallgaps
trimal -in 861921429737580.9_EOG676K40_1@ReadDirectly.fasta -noallgaps
trimal -in 861921429737580.9_EOG6S4PM1_1@ReadDirectly.fasta -noallgaps
trimal -in 861921429737580.9_EOG6NS3GS_1@ReadDirectly.fasta -noallgaps
trimal -in 861921429737580.9_EOG65QHJ5_1@ReadDirectly.fasta -noallgaps
trimal -in 861921429737580.9_EOG62BXCZ_1@ReadDirectly.fasta -noallgaps
trimal -in 861921429737580.9_EOG6DFPSF_1@ReadDirectly.fasta -noallgaps
trimal -in 861921429737580.9_EOG6001NK_1@ReadDirectly.fasta -noallgaps
trimal -in 861921429737580.9_EOG6FFD51_1@ReadDirectly.fasta -noallgaps
trimal -in 861921429737580.9_EOG6RV2WT_1@ReadDirectly.fasta -noallgaps
trimal -in 861921429737580.9_EOG6KD6Q9_1@ReadDirectly.fasta -noallgaps
trimal -in 861921429737580.9_EOG67D9KV_1@ReadDirectly.fasta -noallgaps
trimal -in 861921429737580.9_EOG673PVJ_1@ReadDirectly.fasta -noallgaps
trimal -in 861921429737580.9_EOG670TN2_1@ReadDirectly.fasta -noallgaps
trimal -in 861921429737580.9_EOG6Q2DKD_1@ReadDirectly.fasta -noallgaps
trimal -in 861921429737580.9_EOG6KPTG0_1@ReadDirectly.fasta -noallgaps
trimal -in 861921429737580.9_EOG6SBF24_1@ReadDirectly.fasta -noallgaps
trimal -in 861921429737580.9_EOG6JT092_1@ReadDirectly.fasta -noallgaps
trimal -in 861921429737580.9_EOG6DFPSD_1@ReadDirectly.fasta -noallgaps
trimal -in 861921429737580.9_EOG6229Q2_1@ReadDirectly.fasta -noallgaps
trimal -in 861921429737580.9_EOG6DV5QM_1@ReadDirectly.fasta -noallgaps
trimal -in 861921429737580.9_EOG6CJVMF_1@ReadDirectly.fasta -noallgaps
trimal -in 861921429737580.9_EOG6PK2CM_1@ReadDirectly.fasta -noallgaps
trimal -in 861921429737580.9_EOG6R2400_1@ReadDirectly.fasta -noallgaps
trimal -in 861921429737580.9_EOG6NP77P_1@ReadDirectly.fasta -noallgaps
trimal -in 861921429737580.9_EOG63TZZV_1@ReadDirectly.fasta -noallgaps
trimal -in 861921429737580.9_EOG634W9K_1@ReadDirectly.fasta -noallgaps
trimal -in 861921429737580.9_EOG61C70W_1@ReadDirectly.fasta -noallgaps
trimal -in 861921429737580.9_EOG69KFV1_1@ReadDirectly.fasta -noallgaps
trimal -in 861921429737580.9_EOG6JT09G_1@ReadDirectly.fasta -noallgaps
trimal -in 861921429737580.9_EOG60VVTB_1@ReadDirectly.fasta -noallgaps
trimal -in 861921429737580.9_EOG6F4SFZ_1@ReadDirectly.fasta -noallgaps
trimal -in 861921429737580.9_EOG6JQ426_1@ReadDirectly.fasta -noallgaps
trimal -in 861921429737580.9_EOG6QNN0N_1@ReadDirectly.fasta -noallgaps
trimal -in 861921429737580.9_EOG6QNN0J_1@ReadDirectly.fasta -noallgaps
trimal -in 861921429737580.9_EOG65MN9B_1@ReadDirectly.fasta -noallgaps
trimal -in 861921429737580.9_EOG6QVCGP_1@ReadDirectly.fasta -noallgaps
trimal -in 861921429737580.9_EOG6DBTJX_1@ReadDirectly.fasta -noallgaps
trimal -in 861921429737580.9_EOG6Q8529_1@ReadDirectly.fasta -noallgaps
trimal -in 861921429737580.9_EOG69PB2Z_1@ReadDirectly.fasta -noallgaps
trimal -in 861921429737580.9_EOG698V44_1@ReadDirectly.fasta -noallgaps
trimal -in 861921429737580.9_EOG6NCMH9_1@ReadDirectly.fasta -noallgaps
trimal -in 861921429737580.9_EOG64BB5H_1@ReadDirectly.fasta -noallgaps
trimal -in 861921429737580.9_EOG6PC9WF_1@ReadDirectly.fasta -noallgaps
trimal -in 861921429737580.9_EOG6G1MMG_1@ReadDirectly.fasta -noallgaps
trimal -in 861921429737580.9_EOG6N5W22_1@ReadDirectly.fasta -noallgaps
trimal -in 861921429737580.9_EOG6S7JTK_1@ReadDirectly.fasta -noallgaps
trimal -in 861921429737580.9_EOG6JDHB2_1@ReadDirectly.fasta -noallgaps
trimal -in 861921429737580.9_EOG6GHZSG_1@ReadDirectly.fasta -noallgaps
trimal -in 861921429737580.9_EOG6NCMGZ_1@ReadDirectly.fasta -noallgaps
trimal -in 861921429737580.9_EOG6HDSZD_1@ReadDirectly.fasta -noallgaps
trimal -in 861921429737580.9_EOG65TCRK_1@ReadDirectly.fasta -noallgaps
trimal -in 861921429737580.9_EOG6CG0CT_1@ReadDirectly.fasta -noallgaps
trimal -in 861921429737580.9_EOG66147B_1@ReadDirectly.fasta -noallgaps
trimal -in 861921429737580.9_EOG6G7C30_1@ReadDirectly.fasta -noallgaps
trimal -in 861921429737580.9_EOG6GXFRF_1@ReadDirectly.fasta -noallgaps
trimal -in 861921429737580.9_EOG63N7HF_1@ReadDirectly.fasta -noallgaps
trimal -in 861921429737580.9_EOG61C717_1@ReadDirectly.fasta -noallgaps
trimal -in 861921429737580.9_EOG6P2Q64_1@ReadDirectly.fasta -noallgaps
trimal -in 861921429737580.9_EOG65QHHP_1@ReadDirectly.fasta -noallgaps
trimal -in 861921429737580.9_EOG63BMSB_1@ReadDirectly.fasta -noallgaps
trimal -in 861921429737580.9_EOG6GB79M_1@ReadDirectly.fasta -noallgaps
trimal -in 861921429737580.9_EOG61NTQM_1@ReadDirectly.fasta -noallgaps
trimal -in 861921429737580.9_EOG67H5V2_1@ReadDirectly.fasta -noallgaps
trimal -in 861921429737580.9_EOG6RXZ4G_1@ReadDirectly.fasta -noallgaps
trimal -in 861921429737580.9_EOG6SBF2B_1@ReadDirectly.fasta -noallgaps
trimal -in 861921429737580.9_EOG6BK575_1@ReadDirectly.fasta -noallgaps
trimal -in 861921429737580.9_EOG6P8FN9_1@ReadDirectly.fasta -noallgaps
trimal -in 861921429737580.9_EOG66HGF5_1@ReadDirectly.fasta -noallgaps
trimal -in 861921429737580.9_EOG6FN4NZ_1@ReadDirectly.fasta -noallgaps
trimal -in 861921429737580.9_EOG65TCS9_1@ReadDirectly.fasta -noallgaps
trimal -in 861921429737580.9_EOG6255XS_1@ReadDirectly.fasta -noallgaps
trimal -in 861921429737580.9_EOG6G4GTX_1@ReadDirectly.fasta -noallgaps
trimal -in 861921429737580.9_EOG63FH1F_1@ReadDirectly.fasta -noallgaps
trimal -in 861921429737580.9_EOG62BXD6_1@ReadDirectly.fasta -noallgaps
trimal -in 861921429737580.9_EOG6SJ5J9_1@ReadDirectly.fasta -noallgaps
trimal -in 861921429737580.9_EOG62FSN7_1@ReadDirectly.fasta -noallgaps
trimal -in 861921429737580.9_EOG63212W_1@ReadDirectly.fasta -noallgaps
trimal -in 861921429737580.9_EOG68KRG4_1@ReadDirectly.fasta -noallgaps
trimal -in 861921429737580.9_EOG6SJ5JV_1@ReadDirectly.fasta -noallgaps
trimal -in 861921429737580.9_EOG67D9M5_1@ReadDirectly.fasta -noallgaps
trimal -in 861921429737580.9_EOG641QFW_1@ReadDirectly.fasta -noallgaps
trimal -in 861921429737580.9_EOG6PC9WN_1@ReadDirectly.fasta -noallgaps
trimal -in 861921429737580.9_EOG6C5CNP_1@ReadDirectly.fasta -noallgaps
trimal -in 861921429737580.9_EOG641QFN_1@ReadDirectly.fasta -noallgaps
trimal -in 861921429737580.9_EOG679FBS_1@ReadDirectly.fasta -noallgaps
trimal -in 861921429737580.9_EOG654933_1@ReadDirectly.fasta -noallgaps
trimal -in 861921429737580.9_EOG6907D7_1@ReadDirectly.fasta -noallgaps
trimal -in 861921429737580.9_EOG666VQC_1@ReadDirectly.fasta -noallgaps
trimal -in 861921429737580.9_EOG6FFD52_1@ReadDirectly.fasta -noallgaps
trimal -in 861921429737580.9_EOG60GCVD_1@ReadDirectly.fasta -noallgaps
trimal -in 861921429737580.9_EOG6NP76T_1@ReadDirectly.fasta -noallgaps
trimal -in 861921429737580.9_EOG6HDSZ9_1@ReadDirectly.fasta -noallgaps
trimal -in 861921429737580.9_EOG67SSJD_1@ReadDirectly.fasta -noallgaps
trimal -in 861921429737580.9_EOG62BXCX_1@ReadDirectly.fasta -noallgaps
trimal -in 861921429737580.9_EOG65TCRW_1@ReadDirectly.fasta -noallgaps
trimal -in 861921429737580.9_EOG6STS7W_1@ReadDirectly.fasta -noallgaps
trimal -in 861921429737580.9_EOG68GW6Q_1@ReadDirectly.fasta -noallgaps
trimal -in 861921429737580.9_EOG63TZZW_1@ReadDirectly.fasta -noallgaps
trimal -in 861921429737580.9_EOG602WWN_1@ReadDirectly.fasta -noallgaps
trimal -in 861921429737580.9_EOG6CG0D0_1@ReadDirectly.fasta -noallgaps
trimal -in 861921429737580.9_EOG647FXX_1@ReadDirectly.fasta -noallgaps
trimal -in 861921429737580.9_EOG695ZW4_1@ReadDirectly.fasta -noallgaps
trimal -in 861921429737580.9_EOG651DTX_1@ReadDirectly.fasta -noallgaps
trimal -in 861921429737580.9_EOG6RFKXB_1@ReadDirectly.fasta -noallgaps
trimal -in 861921429737580.9_EOG6MSD2X_1@ReadDirectly.fasta -noallgaps
trimal -in 861921429737580.9_EOG6B8JHB_1@ReadDirectly.fasta -noallgaps
trimal -in 861921429737580.9_EOG6BK57N_1@ReadDirectly.fasta -noallgaps
trimal -in 861921429737580.9_EOG6CZBM5_1@ReadDirectly.fasta -noallgaps
trimal -in 861921429737580.9_EOG6M65N3_1@ReadDirectly.fasta -noallgaps
trimal -in 861921429737580.9_EOG695ZW1_1@ReadDirectly.fasta -noallgaps
trimal -in 861921429737580.9_EOG6868GW_1@ReadDirectly.fasta -noallgaps
trimal -in 861921429737580.9_EOG63R3R9_1@ReadDirectly.fasta -noallgaps
trimal -in 861921429737580.9_EOG63BMSV_1@ReadDirectly.fasta -noallgaps
trimal -in 861921429737580.9_EOG60CHM6_1@ReadDirectly.fasta -noallgaps
trimal -in 861921429737580.9_EOG6FXRC9_1@ReadDirectly.fasta -noallgaps
trimal -in 861921429737580.9_EOG6KSPQ0_1@ReadDirectly.fasta -noallgaps
trimal -in 861921429737580.9_EOG6DR9GP_1@ReadDirectly.fasta -noallgaps
trimal -in 861921429737580.9_EOG6D531X_1@ReadDirectly.fasta -noallgaps
trimal -in 861921429737580.9_EOG6933N7_1@ReadDirectly.fasta -noallgaps
trimal -in 861921429737580.9_EOG60P4BZ_1@ReadDirectly.fasta -noallgaps
trimal -in 861921429737580.9_EOG69GKM6_1@ReadDirectly.fasta -noallgaps
trimal -in 861921429737580.9_EOG66MBP7_1@ReadDirectly.fasta -noallgaps
trimal -in 861921429737580.9_EOG6SJ5J3_1@ReadDirectly.fasta -noallgaps
trimal -in 861921429737580.9_EOG6SJ5J4_1@ReadDirectly.fasta -noallgaps
trimal -in 861921429737580.9_EOG6KWJX8_1@ReadDirectly.fasta -noallgaps
trimal -in 861921429737580.9_EOG6H72G3_1@ReadDirectly.fasta -noallgaps
trimal -in 861921429737580.9_EOG61ZFF6_1@ReadDirectly.fasta -noallgaps
trimal -in 861921429737580.9_EOG6SXNGX_1@ReadDirectly.fasta -noallgaps
trimal -in 861921429737580.9_EOG6R506D_1@ReadDirectly.fasta -noallgaps
trimal -in 861921429737580.9_EOG6DFPRT_1@ReadDirectly.fasta -noallgaps
trimal -in 861921429737580.9_EOG6JDHBG_1@ReadDirectly.fasta -noallgaps
trimal -in 861921429737580.9_EOG6MPHV2_1@ReadDirectly.fasta -noallgaps
trimal -in 861921429737580.9_EOG65DWSM_1@ReadDirectly.fasta -noallgaps
trimal -in 861921429737580.9_EOG6F7NPM_1@ReadDirectly.fasta -noallgaps
trimal -in 861921429737580.9_EOG60K833_1@ReadDirectly.fasta -noallgaps
trimal -in 861921429737580.9_EOG6MW89D_1@ReadDirectly.fasta -noallgaps
trimal -in 861921429737580.9_EOG6PG64S_1@ReadDirectly.fasta -noallgaps
trimal -in 861921429737580.9_EOG6868HF_1@ReadDirectly.fasta -noallgaps
trimal -in 861921429737580.9_EOG6DZ204_1@ReadDirectly.fasta -noallgaps
trimal -in 861921429737580.9_EOG6Q58SW_1@ReadDirectly.fasta -noallgaps
trimal -in 861921429737580.9_EOG68WC58_1@ReadDirectly.fasta -noallgaps
trimal -in 861921429737580.9_EOG6DBTHZ_1@ReadDirectly.fasta -noallgaps
trimal -in 861921429737580.9_EOG6K0QRT_1@ReadDirectly.fasta -noallgaps
trimal -in 861921429737580.9_EOG68PMPX_1@ReadDirectly.fasta -noallgaps
trimal -in 861921429737580.9_EOG6K9BH5_1@ReadDirectly.fasta -noallgaps
trimal -in 861921429737580.9_EOG676K3N_1@ReadDirectly.fasta -noallgaps
trimal -in 861921429737580.9_EOG6CRM3G_1@ReadDirectly.fasta -noallgaps
trimal -in 861921429737580.9_EOG6K6G7S_1@ReadDirectly.fasta -noallgaps
trimal -in 861921429737580.9_EOG64J2NS_1@ReadDirectly.fasta -noallgaps
trimal -in 861921429737580.9_EOG65HS25_1@ReadDirectly.fasta -noallgaps
trimal -in 861921429737580.9_EOG64XJM7_1@ReadDirectly.fasta -noallgaps
trimal -in 861921429737580.9_EOG6HHP6H_1@ReadDirectly.fasta -noallgaps
trimal -in 861921429737580.9_EOG6DV5QK_1@ReadDirectly.fasta -noallgaps
trimal -in 861921429737580.9_EOG68GW6V_1@ReadDirectly.fasta -noallgaps
trimal -in 861921429737580.9_EOG6CC452_1@ReadDirectly.fasta -noallgaps
trimal -in 861921429737580.9_EOG66HGFW_1@ReadDirectly.fasta -noallgaps
trimal -in 861921429737580.9_EOG6NVZQC_1@ReadDirectly.fasta -noallgaps
trimal -in 861921429737580.9_EOG647FZH_1@ReadDirectly.fasta -noallgaps
trimal -in 861921429737580.9_EOG68KRFS_1@ReadDirectly.fasta -noallgaps
trimal -in 861921429737580.9_EOG6001PC_1@ReadDirectly.fasta -noallgaps
trimal -in 861921429737580.9_EOG6F1X6M_1@ReadDirectly.fasta -noallgaps
trimal -in 861921429737580.9_EOG615GHP_1@ReadDirectly.fasta -noallgaps
trimal -in 861921429737580.9_EOG64F6DK_1@ReadDirectly.fasta -noallgaps
trimal -in 861921429737580.9_EOG6NZTZ3_1@ReadDirectly.fasta -noallgaps
trimal -in 861921429737580.9_EOG6QJRRQ_1@ReadDirectly.fasta -noallgaps
trimal -in 861921429737580.9_EOG67H5VB_1@ReadDirectly.fasta -noallgaps
trimal -in 861921429737580.9_EOG69KFTV_1@ReadDirectly.fasta -noallgaps
trimal -in 861921429737580.9_EOG6QC18P_1@ReadDirectly.fasta -noallgaps
trimal -in 861921429737580.9_EOG60P4BD_1@ReadDirectly.fasta -noallgaps
trimal -in 861921429737580.9_EOG6N8R82_1@ReadDirectly.fasta -noallgaps
trimal -in 861921429737580.9_EOG6M65NR_1@ReadDirectly.fasta -noallgaps
trimal -in 861921429737580.9_EOG6K9BGH_1@ReadDirectly.fasta -noallgaps
trimal -in 861921429737580.9_EOG67D9KJ_1@ReadDirectly.fasta -noallgaps
trimal -in 861921429737580.9_EOG6BCDR6_1@ReadDirectly.fasta -noallgaps
trimal -in 861921429737580.9_EOG6GTKHQ_1@ReadDirectly.fasta -noallgaps
trimal -in 861921429737580.9_EOG6FN4N0_1@ReadDirectly.fasta -noallgaps
trimal -in 861921429737580.9_EOG6JDHBD_1@ReadDirectly.fasta -noallgaps
trimal -in 861921429737580.9_EOG615GHV_1@ReadDirectly.fasta -noallgaps
trimal -in 861921429737580.9_EOG6CJVMH_1@ReadDirectly.fasta -noallgaps
trimal -in 861921429737580.9_EOG6CC44S_1@ReadDirectly.fasta -noallgaps
trimal -in 861921429737580.9_EOG6B2T20_1@ReadDirectly.fasta -noallgaps
trimal -in 861921429737580.9_EOG6JQ41Z_1@ReadDirectly.fasta -noallgaps
trimal -in 861921429737580.9_EOG637RJP_1@ReadDirectly.fasta -noallgaps
trimal -in 861921429737580.9_EOG6QRH88_1@ReadDirectly.fasta -noallgaps
trimal -in 861921429737580.9_EOG6933N9_1@ReadDirectly.fasta -noallgaps
trimal -in 861921429737580.9_EOG605S4Z_1@ReadDirectly.fasta -noallgaps
trimal -in 861921429737580.9_EOG6J3WMR_1@ReadDirectly.fasta -noallgaps
trimal -in 861921429737580.9_EOG6Q58T0_1@ReadDirectly.fasta -noallgaps
trimal -in 861921429737580.9_EOG6BVRZ3_1@ReadDirectly.fasta -noallgaps
trimal -in 861921429737580.9_EOG6CJVMM_1@ReadDirectly.fasta -noallgaps
trimal -in 861921429737580.9_EOG6Q8524_1@ReadDirectly.fasta -noallgaps
trimal -in 861921429737580.9_EOG6PNXN7_1@ReadDirectly.fasta -noallgaps
trimal -in 861921429737580.9_EOG6QFWHZ_1@ReadDirectly.fasta -noallgaps
trimal -in 861921429737580.9_EOG6SXNHD_1@ReadDirectly.fasta -noallgaps
trimal -in 861921429737580.9_EOG6B5P96_1@ReadDirectly.fasta -noallgaps
trimal -in 861921429737580.9_EOG6C87WM_1@ReadDirectly.fasta -noallgaps
trimal -in 861921429737580.9_EOG63212K_1@ReadDirectly.fasta -noallgaps
trimal -in 861921429737580.9_EOG63XV6Q_1@ReadDirectly.fasta -noallgaps
trimal -in 861921429737580.9_EOG66Q6XP_1@ReadDirectly.fasta -noallgaps
trimal -in 861921429737580.9_EOG6Q852G_1@ReadDirectly.fasta -noallgaps
trimal -in 861921429737580.9_EOG66MBPR_1@ReadDirectly.fasta -noallgaps
trimal -in 861921429737580.9_EOG6RNBCT_1@ReadDirectly.fasta -noallgaps
trimal -in 861921429737580.9_EOG67WNS9_1@ReadDirectly.fasta -noallgaps
trimal -in 861921429737580.9_EOG6HQDPF_1@ReadDirectly.fasta -noallgaps
trimal -in 861921429737580.9_EOG66Q6X8_1@ReadDirectly.fasta -noallgaps
trimal -in 861921429737580.9_EOG63R3QZ_1@ReadDirectly.fasta -noallgaps
trimal -in 861921429737580.9_EOG6B8JHP_1@ReadDirectly.fasta -noallgaps
trimal -in 861921429737580.9_EOG6HT8WD_1@ReadDirectly.fasta -noallgaps
trimal -in 861921429737580.9_EOG641QGH_1@ReadDirectly.fasta -noallgaps
trimal -in 861921429737580.9_EOG669QZR_1@ReadDirectly.fasta -noallgaps
trimal -in 861921429737580.9_EOG6GMV1P_1@ReadDirectly.fasta -noallgaps
trimal -in 861921429737580.9_EOG61RPZ9_1@ReadDirectly.fasta -noallgaps
trimal -in 861921429737580.9_EOG66147J_1@ReadDirectly.fasta -noallgaps
trimal -in 861921429737580.9_EOG60K843_1@ReadDirectly.fasta -noallgaps
trimal -in 861921429737580.9_EOG6BG90S_1@ReadDirectly.fasta -noallgaps
trimal -in 861921429737580.9_EOG602WX4_1@ReadDirectly.fasta -noallgaps
trimal -in 861921429737580.9_EOG6GF3JF_1@ReadDirectly.fasta -noallgaps
trimal -in 861921429737580.9_EOG6PC9X3_1@ReadDirectly.fasta -noallgaps
trimal -in 861921429737580.9_EOG6HQDPG_1@ReadDirectly.fasta -noallgaps
trimal -in 861921429737580.9_EOG64QT4T_1@ReadDirectly.fasta -noallgaps
trimal -in 861921429737580.9_EOG6BG909_1@ReadDirectly.fasta -noallgaps
trimal -in 861921429737580.9_EOG6H4677_1@ReadDirectly.fasta -noallgaps
trimal -in 861921429737580.9_EOG69ZXTF_1@ReadDirectly.fasta -noallgaps
trimal -in 861921429737580.9_EOG6NVZPX_1@ReadDirectly.fasta -noallgaps
trimal -in 861921429737580.9_EOG68KRFQ_1@ReadDirectly.fasta -noallgaps
trimal -in 861921429737580.9_EOG60P4BJ_1@ReadDirectly.fasta -noallgaps
trimal -in 861921429737580.9_EOG6P8FNH_1@ReadDirectly.fasta -noallgaps
trimal -in 861921429737580.9_EOG6N8R81_1@ReadDirectly.fasta -noallgaps
trimal -in 861921429737580.9_EOG60VVTG_1@ReadDirectly.fasta -noallgaps
trimal -in 861921429737580.9_EOG6RJG4W_1@ReadDirectly.fasta -noallgaps
trimal -in 861921429737580.9_EOG6JT09B_1@ReadDirectly.fasta -noallgaps
trimal -in 861921429737580.9_EOG670TNG_1@ReadDirectly.fasta -noallgaps
trimal -in 861921429737580.9_EOG6CG0CX_1@ReadDirectly.fasta -noallgaps
trimal -in 861921429737580.9_EOG6D26TV_1@ReadDirectly.fasta -noallgaps
trimal -in 861921429737580.9_EOG666VQ4_1@ReadDirectly.fasta -noallgaps
trimal -in 861921429737580.9_EOG6FTW4C_1@ReadDirectly.fasta -noallgaps
trimal -in 861921429737580.9_EOG60VVT3_1@ReadDirectly.fasta -noallgaps
trimal -in 861921429737580.9_EOG64BB5X_1@ReadDirectly.fasta -noallgaps
trimal -in 861921429737580.9_EOG654930_1@ReadDirectly.fasta -noallgaps
trimal -in 861921429737580.9_EOG6Q2DJZ_1@ReadDirectly.fasta -noallgaps
trimal -in 861921429737580.9_EOG63R3R5_1@ReadDirectly.fasta -noallgaps
trimal -in 861921429737580.9_EOG6QFWJH_1@ReadDirectly.fasta -noallgaps
trimal -in 861921429737580.9_EOG6SN1SH_1@ReadDirectly.fasta -noallgaps
trimal -in 861921429737580.9_EOG6894QQ_1@ReadDirectly.fasta -noallgaps
trimal -in 861921429737580.9_EOG6HX54P_1@ReadDirectly.fasta -noallgaps
trimal -in 861921429737580.9_EOG6QFWJ6_1@ReadDirectly.fasta -noallgaps
trimal -in 861921429737580.9_EOG6FJ8D6_1@ReadDirectly.fasta -noallgaps
trimal -in 861921429737580.9_EOG6R7VF9_1@ReadDirectly.fasta -noallgaps
trimal -in 861921429737580.9_EOG6S4PKX_1@ReadDirectly.fasta -noallgaps
trimal -in 861921429737580.9_EOG680J16_1@ReadDirectly.fasta -noallgaps
trimal -in 861921429737580.9_EOG6PK2CG_1@ReadDirectly.fasta -noallgaps
trimal -in 861921429737580.9_EOG6H72G0_1@ReadDirectly.fasta -noallgaps
trimal -in 861921429737580.9_EOG6PNXM9_1@ReadDirectly.fasta -noallgaps
trimal -in 861921429737580.9_EOG67PX9B_1@ReadDirectly.fasta -noallgaps
trimal -in 861921429737580.9_EOG64F6DH_1@ReadDirectly.fasta -noallgaps
trimal -in 861921429737580.9_EOG65759X_1@ReadDirectly.fasta -noallgaps
trimal -in 861921429737580.9_EOG6PG646_1@ReadDirectly.fasta -noallgaps
trimal -in 861921429737580.9_EOG6KKZ71_1@ReadDirectly.fasta -noallgaps
trimal -in 861921429737580.9_EOG6JM7SX_1@ReadDirectly.fasta -noallgaps
trimal -in 861921429737580.9_EOG6DZ201_1@ReadDirectly.fasta -noallgaps
trimal -in 861921429737580.9_EOG6SBF2V_1@ReadDirectly.fasta -noallgaps
trimal -in 861921429737580.9_EOG65MN8T_1@ReadDirectly.fasta -noallgaps
trimal -in 861921429737580.9_EOG6NP773_1@ReadDirectly.fasta -noallgaps
trimal -in 861921429737580.9_EOG68SGX8_1@ReadDirectly.fasta -noallgaps
trimal -in 861921429737580.9_EOG6J3WM7_1@ReadDirectly.fasta -noallgaps
trimal -in 861921429737580.9_EOG6STS87_1@ReadDirectly.fasta -noallgaps
trimal -in 861921429737580.9_EOG651DV5_1@ReadDirectly.fasta -noallgaps
trimal -in 861921429737580.9_EOG66DM62_1@ReadDirectly.fasta -noallgaps
trimal -in 861921429737580.9_EOG605S4X_1@ReadDirectly.fasta -noallgaps
trimal -in 861921429737580.9_EOG6K3M19_1@ReadDirectly.fasta -noallgaps
trimal -in 861921429737580.9_EOG6NZTXP_1@ReadDirectly.fasta -noallgaps
trimal -in 861921429737580.9_EOG65493R_1@ReadDirectly.fasta -noallgaps
trimal -in 861921429737580.9_EOG69GKMS_1@ReadDirectly.fasta -noallgaps
trimal -in 861921429737580.9_EOG60ZR1Z_1@ReadDirectly.fasta -noallgaps
trimal -in 861921429737580.9_EOG6D5327_1@ReadDirectly.fasta -noallgaps
trimal -in 861921429737580.9_EOG6PNXM6_1@ReadDirectly.fasta -noallgaps
trimal -in 861921429737580.9_EOG6575BP_1@ReadDirectly.fasta -noallgaps
trimal -in 861921429737580.9_EOG68D102_1@ReadDirectly.fasta -noallgaps
trimal -in 861921429737580.9_EOG60ZR1T_1@ReadDirectly.fasta -noallgaps
trimal -in 861921429737580.9_EOG6GXFR5_1@ReadDirectly.fasta -noallgaps
trimal -in 861921429737580.9_EOG6NS3FV_1@ReadDirectly.fasta -noallgaps
trimal -in 861921429737580.9_EOG62V8KS_1@ReadDirectly.fasta -noallgaps
trimal -in 861921429737580.9_EOG6PRSVP_1@ReadDirectly.fasta -noallgaps
trimal -in 861921429737580.9_EOG6JWVJQ_1@ReadDirectly.fasta -noallgaps
trimal -in 861921429737580.9_EOG6QZ7Q8_1@ReadDirectly.fasta -noallgaps
trimal -in 861921429737580.9_EOG6NVZPZ_1@ReadDirectly.fasta -noallgaps
trimal -in 861921429737580.9_EOG63N7HX_1@ReadDirectly.fasta -noallgaps
trimal -in 861921429737580.9_EOG6QC198_1@ReadDirectly.fasta -noallgaps
trimal -in 861921429737580.9_EOG6C2HF4_1@ReadDirectly.fasta -noallgaps
trimal -in 861921429737580.9_EOG6NP779_1@ReadDirectly.fasta -noallgaps
trimal -in 861921429737580.9_EOG6S1TBX_1@ReadDirectly.fasta -noallgaps
trimal -in 861921429737580.9_EOG6F7NPN_1@ReadDirectly.fasta -noallgaps
trimal -in 861921429737580.9_EOG6D26TG_1@ReadDirectly.fasta -noallgaps
trimal -in 861921429737580.9_EOG6CJVMQ_1@ReadDirectly.fasta -noallgaps
trimal -in 861921429737580.9_EOG63BMSG_1@ReadDirectly.fasta -noallgaps
trimal -in 861921429737580.9_EOG6QZ7R6_1@ReadDirectly.fasta -noallgaps
trimal -in 861921429737580.9_EOG6SXNHG_1@ReadDirectly.fasta -noallgaps
trimal -in 861921429737580.9_EOG69S6BG_1@ReadDirectly.fasta -noallgaps
trimal -in 861921429737580.9_EOG6R506X_1@ReadDirectly.fasta -noallgaps
trimal -in 861921429737580.9_EOG608ND4_1@ReadDirectly.fasta -noallgaps
trimal -in 861921429737580.9_EOG6640GX_1@ReadDirectly.fasta -noallgaps
trimal -in 861921429737580.9_EOG6G4GTT_1@ReadDirectly.fasta -noallgaps
trimal -in 861921429737580.9_EOG695ZWC_1@ReadDirectly.fasta -noallgaps
trimal -in 861921429737580.9_EOG6S1TC2_1@ReadDirectly.fasta -noallgaps
trimal -in 861921429737580.9_EOG69KFVF_1@ReadDirectly.fasta -noallgaps
trimal -in 861921429737580.9_EOG683D8Q_1@ReadDirectly.fasta -noallgaps
trimal -in 861921429737580.9_EOG66MBNX_1@ReadDirectly.fasta -noallgaps
trimal -in 861921429737580.9_EOG680J15_1@ReadDirectly.fasta -noallgaps
trimal -in 861921429737580.9_EOG6GMV1H_1@ReadDirectly.fasta -noallgaps
trimal -in 861921429737580.9_EOG69KFV8_1@ReadDirectly.fasta -noallgaps
trimal -in 861921429737580.9_EOG66WZCQ_1@ReadDirectly.fasta -noallgaps
trimal -in 861921429737580.9_EOG6KSPPS_1@ReadDirectly.fasta -noallgaps
trimal -in 861921429737580.9_EOG6PRSVH_1@ReadDirectly.fasta -noallgaps
trimal -in 861921429737580.9_EOG615GJK_1@ReadDirectly.fasta -noallgaps
trimal -in 861921429737580.9_EOG68SGXD_1@ReadDirectly.fasta -noallgaps
trimal -in 861921429737580.9_EOG6P2Q5K_1@ReadDirectly.fasta -noallgaps
trimal -in 861921429737580.9_EOG67D9KC_1@ReadDirectly.fasta -noallgaps
trimal -in 861921429737580.9_EOG6SJ5JS_1@ReadDirectly.fasta -noallgaps
trimal -in 861921429737580.9_EOG6B8JHC_1@ReadDirectly.fasta -noallgaps
trimal -in 861921429737580.9_EOG61RPZF_1@ReadDirectly.fasta -noallgaps
trimal -in 861921429737580.9_EOG6P8FNB_1@ReadDirectly.fasta -noallgaps
trimal -in 861921429737580.9_EOG6J6RV9_1@ReadDirectly.fasta -noallgaps
trimal -in 861921429737580.9_EOG6SJ5K0_1@ReadDirectly.fasta -noallgaps
trimal -in 861921429737580.9_EOG65X811_1@ReadDirectly.fasta -noallgaps
trimal -in 861921429737580.9_EOG63V009_1@ReadDirectly.fasta -noallgaps
trimal -in 861921429737580.9_EOG6DZ1ZZ_1@ReadDirectly.fasta -noallgaps
trimal -in 861921429737580.9_EOG6KWJXS_1@ReadDirectly.fasta -noallgaps
trimal -in 861921429737580.9_EOG6KKZ6X_1@ReadDirectly.fasta -noallgaps
trimal -in 861921429737580.9_EOG6S1TBT_1@ReadDirectly.fasta -noallgaps
trimal -in 861921429737580.9_EOG6FR0W5_1@ReadDirectly.fasta -noallgaps
trimal -in 861921429737580.9_EOG6SQX12_1@ReadDirectly.fasta -noallgaps
trimal -in 861921429737580.9_EOG6M39DN_1@ReadDirectly.fasta -noallgaps
trimal -in 861921429737580.9_EOG6CC44M_1@ReadDirectly.fasta -noallgaps
trimal -in 861921429737580.9_EOG6FFD5C_1@ReadDirectly.fasta -noallgaps
trimal -in 861921429737580.9_EOG63R3RK_1@ReadDirectly.fasta -noallgaps
trimal -in 861921429737580.9_EOG65TCRS_1@ReadDirectly.fasta -noallgaps
trimal -in 861921429737580.9_EOG6C87X0_1@ReadDirectly.fasta -noallgaps
trimal -in 861921429737580.9_EOG6RFKXS_1@ReadDirectly.fasta -noallgaps
trimal -in 861921429737580.9_EOG6R506Q_1@ReadDirectly.fasta -noallgaps
trimal -in 861921429737580.9_EOG676K44_1@ReadDirectly.fasta -noallgaps
trimal -in 861921429737580.9_EOG64MXWZ_1@ReadDirectly.fasta -noallgaps
trimal -in 861921429737580.9_EOG68WC5K_1@ReadDirectly.fasta -noallgaps
```

### The TrimalConf default values:¶

```
TrimalConf(pj, 
           method_name='gappyout',
           program_name='trimal',
           cmd='default',                      # The defaults cmd is in Project.defaults['trimal']
                                               # You can change the default value or provide it here.

           alns='all',                         # Or a list of specific alignmnet names.
           trimal_commands={'gappyout': True})
```

### Trimming execution¶

The trim Project method excepts a list of one or more `TrimalConf` objects. Different objects can be used to trim different subsets of alignments.

In [15]:

```
pj.trim([trimal])
```

## 2.6 Preparing supermatrices from 200 loci long sliding windows along the entropy gradient¶

### 2.6.1 Calculating the statistics¶

Entropy, conservation, and gap score will be calculated for each position in each alignmnet.  
Sequence length and GC content will be calculated for each untrimmed sequence in each locus.

This is done by passing the `Project` instance (`pj`) into a `LociStats` object:

In [4]:

```
stats = LociStats(pj,                      # The Project instance
                  
                  trimmed=True,            # Only trimmed alignments will be checked
                  
                  alignmnet_method=None,   # If specified, only regard alignments created
                                           # with this method
                                           
                  trimming_method=None)    # If specified, only regard alignments trimmed
                                           # with this method
    
                                           # By default, only regard the first occurance
                                           # of each locus, if it was aligned or trimmed
                                           # using more than one method
```

### 2.6.2 Sort the alignmnets according to statistics values¶

This done by using the `sort` method of the `LociStats` instance:

In [5]:

```
stats.sort(parameter = 'entropy',     # Sort the loci according to entropy values
           
           percentile=50,             # First sort by the specified percentile of each locus,
                                      # typically, the median
                                      
           percentile_range=(25,75),  # Then, sort by the distance between the specified percentiles
                                      # for each locus, typically the 25 and 75 percentiles
                                      
           reverse = True)            # Sort in descending order
```

### 2.6.3 Figure 2 A¶

**The next cell produced Figure 2A**. It plots the entropy distribution as a box-plot for each locus. The brown dot is the median, the blue stretch is the 25-75 percentiles, the black stretches are the > 95 and < 5 percentiles.

In [6]:

```
%matplotlib inline

stats.plot('entropy.png',     # Output file name
           
           figsize=(30,7),    # Figure size in cm
           
           params=['entropy'],# The parameters to include.
                              # can take a list of just one or a few parameters
                              # including 'entropy','conservation', 'gapscore'
                              # 'sequence_length' and 'sequence_gc'
           
           lable_fsize=40,
           
           xtick_fsize=0,
           
           ytick_fsize=1,
          
           boxcolor='DodgerBlue',
           whiskercolor='black',
           capcolor='black',
           mediancolor='SaddleBrown',
           medianline_w=5)
```

### 2.6.4 Figure S1, Plot a box plot for each parameter for each locus¶

In the resulting figure the loci will be sorted in a descending order of the entropy median, as a result of the above sorting step.
As expected, entropy is correlated with the conservation value. It is not correlated with other measures which makes its effect easy to check.

In [5]:

```
stats.plot('plot.png',     # Output file name
           
           figsize=(50,23), # Figure size in cm
           
           params='all',    # The parameters to include.
                            # can take a list of just one
                            # or a few parameters
                            # including 'entropy','conservation',
                            # 'gapscore', 'sequence_length'
                            # and 'sequence_gc'
           
           lable_fsize=20,
           
           xtick_fsize=10,
           
           ytick_fsize=1,
          
           boxcolor='salmon',
           whiskercolor='gray',
           capcolor='black',
           mediancolor='white',
           medianline_w=3)
```

## 2.7 Prepare the Concatenation objects¶

`Concatenation` objects contain the configuration information for building a supermatrix. This will produce six `Concatenation` objects each with a sliding window of 200 loci, along the entropy gradient.

In [21]:

```
concatenations = stats.slide_loci('source_original_id', # OTU metadata value.
                                                        # sequences from different loci
                                                        # ,which share this value,
                                                        # will be considered to belong to
                                                        # the same sample and will be
                                                        # concatenated
                                  
                                  median_range='all',   # Before starting the slide,
                                                        # you can exclude loci
                                  parameter='entropy',  # which median fall outside
                                                        # the specified range
                                                        # of the specified parameter.
                                                        # The remaining loci will still
                                                        # be sorted according to the
                                                        # parameter specified in the
                                                        # sorting step above, and not
                                                        # according to the parameter that
                                                        # specified here, if it is
                                                        # different from above.
                                  
                                  start=0,              # Counting from the first locus
                                                        # that was not excluded,
                                                        # start the first window here.
                                  
                                  length=200,           # the length of the window,
                                                        # counting 200 loci that were
                                                        # not filtered out
                                  
                                  step=50)              # the length of the slide.

# Add the concatenation objects to the project
for concat in concatenations:
    pj.add_concatenation(concat)
    
# Create the supermatrices
pj.make_concatenation_alignments()

# And collect their names
aln_names = [c.name for c in pj.concatenations]
```

```
entropy_1.14_0.00_loci_0_to_199
entropy_0.48_0.00_loci_50_to_249
entropy_0.30_0.00_loci_100_to_299
entropy_0.00_0.00_loci_150_to_349
entropy_0.00_0.00_loci_200_to_399
entropy_0.00_0.00_loci_250_to_449
Concatenation entropy_1.14_0.00_loci_0_to_199 will have the following data
OTU                           EOG6M65NR_1         EOG6SN1SH_1         EOG654933_1         EOG66147B_1         EOG680J15_1         EOG6S7JTK_1         EOG60ZR1Z_1         EOG6DBTJG_1         EOG69ZXTF_1         EOG64QT4T_1         EOG6K3M19_1         EOG60P4BJ_1         EOG6DBTK0_1         EOG64MXX7_1         EOG6GTKHJ_1         EOG6NVZQC_1         EOG6MPHV2_1         EOG68KRFS_1         EOG6GHZSR_1         EOG666VQ4_1         EOG651DV5_1         EOG6JDHBD_1         EOG63212K_1         EOG68D102_1         EOG6CJVMH_1         EOG60VVTB_1         EOG698V46_1         EOG6N8R81_1         EOG6FXRCK_1         EOG6R2400_1         EOG68KRG4_1         EOG61C70T_1         EOG6SJ5JS_1         EOG63212W_1         EOG6F7NPJ_1         EOG695ZW4_1         EOG61NTQM_1         EOG6NZTZ3_1         EOG6J3WM7_1         EOG68SGXM_1         EOG6RNBCT_1         EOG6M39DN_1         EOG6RFKXS_1         EOG6RXZ4G_1         EOG67SSJJ_1         EOG69GKKW_1         EOG6NZTXP_1         EOG6QFWHZ_1         EOG68PMPX_1         EOG6RFKXB_1         EOG62BXD6_1         EOG647FXX_1         EOG6B8JHP_1         EOG6S1TBX_1         EOG6DFPRT_1         EOG65HS25_1         EOG6FFD5C_1         EOG6R506D_1         EOG670TNG_1         EOG6F4SFZ_1         EOG6QVCGP_1         EOG65TCRS_1         EOG6QRH88_1         EOG6M65N6_1         EOG61G385_1         EOG6QRH81_1         EOG6RJG4W_1         EOG6K0QSG_1         EOG6KKZ6X_1         EOG60VVTG_1         EOG6D26TG_1         EOG6QZ7R6_1         EOG679FBS_1         EOG61RPZF_1         EOG6PC9WG_1         EOG60GCVD_1         EOG6R506Q_1         EOG605S4X_1         EOG65TCRN_1         EOG608ND4_1         EOG6640GX_1         EOG6SQX12_1         EOG69GKMS_1         EOG6D26TV_1         EOG605S4Z_1         EOG6N04JN_1         EOG67WNS9_1         EOG64TPCQ_1         EOG6NS3GS_1         EOG6NP76T_1         EOG63JC90_1         EOG67H5V2_1         EOG6NP77P_1         EOG6QFWJH_1         EOG6N04K4_1         EOG6CJVMQ_1         EOG6DZ204_1         EOG6C5CPH_1         EOG6FFD51_1         EOG6NP779_1         EOG6933N9_1         EOG6JQ427_1         EOG6C5CNP_1         EOG6FTW4C_1         EOG65759X_1         EOG6QNN0N_1         EOG66WZD1_1         EOG6FR0WR_1         EOG6P2Q64_1         EOG666VQC_1         EOG63R3R5_1         EOG641QGH_1         EOG69S6BG_1         EOG64XJM7_1         EOG65MN9B_1         EOG641QFW_1         EOG63R3R9_1         EOG6N04J6_1         EOG6GMV1P_1         EOG6S4PKX_1         EOG6NP773_1         EOG6DZ201_1         EOG6QFWJ6_1         EOG6CG0CT_1         EOG6HHP6B_1         EOG605S4Q_1         EOG6R7VF9_1         EOG6CZBM5_1         EOG6JT092_1         EOG6FJ8D6_1         EOG6QZ7QX_1         EOG6F1X6M_1         EOG6SBF2V_1         EOG6KSPQ0_1         EOG6GMV1H_1         EOG63R3RC_1         EOG68SGX8_1         EOG6BK57S_1         EOG60K83N_1         EOG6KD6Q9_1         EOG68GW6Q_1         EOG6Q852G_1         EOG676K44_1         EOG6FN4NZ_1         EOG6S1TC2_1         EOG6HT8WD_1         EOG63FH1F_1         EOG6PRSVP_1         EOG6R5069_1         EOG6CC453_1         EOG6001PC_1         EOG676K40_1         EOG6N30SN_1         EOG67D9M5_1         EOG6RBQP0_1         EOG6Q2DKD_1         EOG6255XM_1         EOG64BB5X_1         EOG6H72G0_1         EOG6PZJB1_1         EOG64MXWG_1         EOG65QHHP_1         EOG698V4R_1         EOG615GHV_1         EOG6FFD5G_1         EOG6NVZQ8_1         EOG60K833_1         EOG62BXCZ_1         EOG6CG0D0_1         EOG69KFVF_1         EOG615GHP_1         EOG683D8Q_1         EOG6PRSVM_1         EOG64J2NS_1         EOG66HGF5_1         EOG60P4BZ_1         EOG66MBPR_1         EOG6DFPSD_1         EOG64BB5N_1         EOG6DBTJX_1         EOG6FXRC9_1         EOG6STS87_1         EOG6SXNHG_1         EOG6255XS_1         EOG6GF3JF_1         EOG6KSPPS_1         EOG637RJP_1         EOG6J3WMR_1         EOG6B2T20_1         EOG6GB79H_1         EOG6JDHBG_1         EOG676K3Q_1         EOG6RV2WT_1         EOG6GTKHQ_1         EOG6FR0W5_1         EOG6CC452_1         EOG61G37F_1         EOG6GXFR5_1         EOG6DV5QK_1         EOG6KPTG0_1         
Dplexcds                      denovo28_f0    denovo80_f0    denovo106_f0   denovo132_f0   denovo210_f0   denovo236_f0   denovo262_f0   denovo288_f0   denovo340_f0   denovo418_f0   denovo522_f0   denovo574_f0   denovo600_f0   denovo730_f0   denovo782_f0   denovo938_f0   denovo964_f0   denovo1042_f0  denovo1068_f0  denovo1146_f0  denovo1302_f0  denovo1484_f0  denovo1510_f0  denovo1640_f0  denovo1744_f0  denovo1770_f0  denovo1796_f0  denovo1952_f0  denovo2134_f0  denovo2186_f0  denovo2238_f0  denovo2264_f0  denovo2342_f0  denovo2446_f0  denovo2472_f0  denovo2498_f0  denovo2524_f0  denovo2576_f0  denovo2602_f0  denovo2654_f0  denovo2758_f0  denovo2784_f0  denovo2836_f0  denovo2862_f0  denovo2914_f0  denovo2992_f0  denovo3044_f0  denovo3148_f0  denovo3226_f0  denovo3252_f0  denovo3278_f0  denovo3304_f0  denovo3330_f0  denovo3434_f0  denovo3486_f0  denovo3590_f0  denovo3616_f0  denovo3668_f0  denovo3694_f0  denovo3720_f0  denovo3772_f0  denovo3850_f0  denovo3954_f0  denovo3980_f0  denovo4032_f0  denovo4058_f0  denovo4188_f0  denovo4292_f0  denovo4318_f0  denovo4344_f0  denovo4396_f0  denovo4448_f0  denovo4552_f0  denovo4630_f0  denovo4734_f0  denovo4760_f0  denovo4786_f0  denovo4838_f0  denovo4890_f0  denovo4916_f0  denovo4942_f0  denovo4968_f0  denovo4994_f0  denovo5202_f0  denovo5228_f0  denovo5254_f0  denovo5384_f0  denovo5436_f0  denovo5514_f0  denovo5592_f0  denovo5618_f0  denovo5644_f0  denovo5930_f0  denovo6008_f0  denovo6060_f0  denovo6112_f0  denovo6216_f0  denovo6242_f0  denovo6268_f0  denovo6580_f0  denovo6606_f0  denovo6684_f0  denovo6710_f0  denovo6762_f0  denovo6814_f0  denovo6866_f0  denovo6996_f0  denovo7074_f0  denovo7126_f0  denovo7152_f0  denovo7256_f0  denovo7334_f0  denovo7386_f0  denovo7412_f0  denovo7516_f0  denovo7542_f0  denovo7568_f0  denovo7620_f0  denovo7672_f0  denovo7698_f0  denovo7750_f0  denovo7802_f0  denovo7984_f0  denovo8036_f0  denovo8062_f0  denovo8218_f0  denovo8244_f0  denovo8270_f0  denovo8296_f0  denovo8400_f0  denovo8426_f0  denovo8452_f0  denovo8582_f0  denovo8634_f0  denovo8660_f0  denovo8686_f0  denovo8764_f0  denovo8816_f0  denovo8842_f0  denovo8894_f0  denovo8920_f0  denovo8946_f0  denovo8972_f0  denovo8998_f0  denovo9128_f0  denovo9154_f0  denovo9180_f0  denovo9310_f0  denovo9336_f0  denovo9362_f0  denovo9388_f0  denovo9440_f0  denovo9492_f0  denovo9544_f0  denovo9596_f0  denovo9674_f0  denovo9700_f0  denovo9726_f0  denovo9804_f0  denovo9830_f0  denovo9856_f0  denovo9882_f0  denovo9934_f0  denovo9960_f0  denovo9986_f0  denovo10064_f0 denovo10168_f0 denovo10194_f0 denovo10220_f0 denovo10272_f0 denovo10298_f0 denovo10350_f0 denovo10376_f0 denovo10402_f0 denovo10428_f0 denovo10480_f0 denovo10506_f0 denovo10532_f0 denovo10610_f0 denovo10662_f0 denovo10688_f0 denovo10766_f0 denovo10844_f0 denovo10870_f0 denovo10922_f0 denovo10974_f0 denovo11026_f0 denovo11078_f0 denovo11104_f0 denovo11156_f0 denovo11182_f0 denovo11234_f0 denovo11260_f0 denovo11312_f0 denovo11338_f0 denovo11442_f0 denovo11546_f0 denovo11910_f0 denovo11962_f0 denovo12066_f0 
FG120077                      denovo36_f0    denovo88_f0    denovo114_f0   denovo140_f0   denovo218_f0   denovo244_f0   denovo270_f0   denovo296_f0   denovo348_f0   denovo426_f0   denovo530_f0   denovo582_f0   denovo608_f0   denovo738_f0   denovo790_f0   denovo946_f0   denovo972_f0   denovo1050_f0  denovo1076_f0  denovo1154_f0  denovo1310_f0  denovo1492_f0  denovo1518_f0  denovo1648_f0  denovo1752_f0  denovo1778_f0  denovo1804_f0  denovo1960_f0  denovo2142_f0  denovo2194_f0  denovo2246_f0  denovo2272_f0  denovo2350_f0  denovo2454_f0  denovo2480_f0  denovo2506_f0  denovo2532_f0  denovo2584_f0  denovo2610_f0  denovo2662_f0  denovo2766_f0  denovo2792_f0  denovo2844_f0  denovo2870_f0  denovo2922_f0  denovo3000_f0  denovo3052_f0  denovo3156_f0  denovo3234_f0  denovo3260_f0  denovo3286_f0  denovo3312_f0  denovo3338_f0  denovo3442_f0  denovo3494_f0  denovo3598_f0  denovo3624_f0  denovo3676_f0  denovo3702_f0  denovo3728_f0  denovo3780_f0  denovo3858_f0  denovo3962_f0  denovo3988_f0  denovo4040_f0  denovo4066_f0  denovo4196_f0  denovo4300_f0  denovo4326_f0  denovo4352_f0  denovo4404_f0  denovo4456_f0  denovo4560_f0  denovo4638_f0  denovo4742_f0  denovo4768_f0  denovo4794_f0  denovo4846_f0  denovo4898_f0  denovo4924_f0  denovo4950_f0  denovo4976_f0  denovo5002_f0  denovo5210_f0  denovo5236_f0  denovo5262_f0  denovo5392_f0  denovo5444_f0  denovo5522_f0  denovo5600_f0  denovo5626_f0  denovo5652_f0  denovo5938_f0  denovo6016_f0  denovo6068_f0  denovo6120_f0  denovo6224_f0  denovo6250_f0  denovo6276_f0  denovo6588_f0  denovo6614_f0  denovo6692_f0  denovo6718_f0  denovo6770_f0  denovo6822_f0  denovo6874_f0  denovo7004_f0  denovo7082_f0  denovo7134_f0  denovo7160_f0  denovo7264_f0  denovo7342_f0  denovo7394_f0  denovo7420_f0  denovo7524_f0  denovo7550_f0  denovo7576_f0  denovo7628_f0  denovo7680_f0  denovo7706_f0  denovo7758_f0  denovo7810_f0  denovo7992_f0  denovo8044_f0  denovo8070_f0  denovo8226_f0  denovo8252_f0  denovo8278_f0  denovo8304_f0  denovo8408_f0  denovo8434_f0  denovo8460_f0  denovo8590_f0  denovo8642_f0  denovo8668_f0  denovo8694_f0  denovo8772_f0  denovo8824_f0  denovo8850_f0  denovo8902_f0  denovo8928_f0  denovo8954_f0  denovo8980_f0  denovo9006_f0  denovo9136_f0  denovo9162_f0  denovo9188_f0  denovo9318_f0  denovo9344_f0  denovo9370_f0  denovo9396_f0  denovo9448_f0  denovo9500_f0  denovo9552_f0  denovo9604_f0  denovo9682_f0  denovo9708_f0  denovo9734_f0  denovo9812_f0  denovo9838_f0  denovo9864_f0  denovo9890_f0  denovo9942_f0  denovo9968_f0  denovo9994_f0  denovo10072_f0 denovo10176_f0 denovo10202_f0 denovo10228_f0 denovo10280_f0 denovo10306_f0 denovo10358_f0 denovo10384_f0 denovo10410_f0 denovo10436_f0 denovo10488_f0 denovo10514_f0 denovo10540_f0 denovo10618_f0 denovo10670_f0 denovo10696_f0 denovo10774_f0 denovo10852_f0 denovo10878_f0 denovo10930_f0 denovo10982_f0 denovo11034_f0 denovo11086_f0 denovo11112_f0 denovo11164_f0 denovo11190_f0 denovo11242_f0 denovo11268_f0 denovo11320_f0 denovo11346_f0 denovo11450_f0 denovo11554_f0 denovo11918_f0 denovo11970_f0 denovo12074_f0 
SRR803483                     denovo46_f0    denovo98_f0    denovo124_f0   denovo150_f0   denovo228_f0   denovo254_f0   denovo280_f0   denovo306_f0   denovo358_f0   denovo436_f0   denovo540_f0   denovo592_f0   denovo618_f0   denovo748_f0   denovo800_f0   denovo956_f0   denovo982_f0   denovo1060_f0  denovo1086_f0  denovo1164_f0  denovo1320_f0  denovo1502_f0  denovo1528_f0  denovo1658_f0  denovo1762_f0  denovo1788_f0  denovo1814_f0  denovo1970_f0  denovo2152_f0  denovo2204_f0  denovo2256_f0  denovo2282_f0  denovo2360_f0  denovo2464_f0  denovo2490_f0  denovo2516_f0  denovo2542_f0  denovo2594_f0  denovo2620_f0  denovo2672_f0  denovo2776_f0  denovo2802_f0  denovo2854_f0  denovo2880_f0  denovo2932_f0  denovo3010_f0  denovo3062_f0  denovo3166_f0  denovo3244_f0  denovo3270_f0  denovo3296_f0  denovo3322_f0  denovo3348_f0  denovo3452_f0  denovo3504_f0  denovo3608_f0  denovo3634_f0  denovo3686_f0  denovo3712_f0  denovo3738_f0  denovo3790_f0  denovo3868_f0  denovo3972_f0  denovo3998_f0  denovo4050_f0  denovo4076_f0  denovo4206_f0  denovo4310_f0  denovo4336_f0  denovo4362_f0  denovo4414_f0  denovo4466_f0  denovo4570_f0  denovo4648_f0  denovo4752_f0  denovo4778_f0  denovo4804_f0  denovo4856_f0  denovo4908_f0  denovo4934_f0  denovo4960_f0  denovo4986_f0  denovo5012_f0  denovo5220_f0  denovo5246_f0  denovo5272_f0  denovo5402_f0  denovo5454_f0  denovo5532_f0  denovo5610_f0  denovo5636_f0  denovo5662_f0  denovo5948_f0  denovo6026_f0  denovo6078_f0  denovo6130_f0  denovo6234_f0  denovo6260_f0  denovo6286_f0  denovo6598_f0  denovo6624_f0  denovo6702_f0  denovo6728_f0  denovo6780_f0  denovo6832_f0  denovo6884_f0  denovo7014_f0  denovo7092_f0  denovo7144_f0  denovo7170_f0  denovo7274_f0  denovo7352_f0  denovo7404_f0  denovo7430_f0  denovo7534_f0  denovo7560_f0  denovo7586_f0  denovo7638_f0  denovo7690_f0  denovo7716_f0  denovo7768_f0  denovo7820_f0  denovo8002_f0  denovo8054_f0  denovo8080_f0  denovo8236_f0  denovo8262_f0  denovo8288_f0  denovo8314_f0  denovo8418_f0  denovo8444_f0  denovo8470_f0  denovo8600_f0  denovo8652_f0  denovo8678_f0  denovo8704_f0  denovo8782_f0  denovo8834_f0  denovo8860_f0  denovo8912_f0  denovo8938_f0  denovo8964_f0  denovo8990_f0  denovo9016_f0  denovo9146_f0  denovo9172_f0  denovo9198_f0  denovo9328_f0  denovo9354_f0  denovo9380_f0  denovo9406_f0  denovo9458_f0  denovo9510_f0  denovo9562_f0  denovo9614_f0  denovo9692_f0  denovo9718_f0  denovo9744_f0  denovo9822_f0  denovo9848_f0  denovo9874_f0  denovo9900_f0  denovo9952_f0  denovo9978_f0  denovo10004_f0 denovo10082_f0 denovo10186_f0 denovo10212_f0 denovo10238_f0 denovo10290_f0 denovo10316_f0 denovo10368_f0 denovo10394_f0 denovo10420_f0 denovo10446_f0 denovo10498_f0 denovo10524_f0 denovo10550_f0 denovo10628_f0 denovo10680_f0 denovo10706_f0 denovo10784_f0 denovo10862_f0 denovo10888_f0 denovo10940_f0 denovo10992_f0 denovo11044_f0 denovo11096_f0 denovo11122_f0 denovo11174_f0 denovo11200_f0 denovo11252_f0 denovo11278_f0 denovo11330_f0 denovo11356_f0 denovo11460_f0 denovo11564_f0 denovo11928_f0 denovo11980_f0 denovo12084_f0 
FG120035                      denovo31_f0    denovo83_f0    denovo109_f0   denovo135_f0   denovo213_f0   denovo239_f0   denovo265_f0   denovo291_f0   denovo343_f0   denovo421_f0   denovo525_f0   denovo577_f0   denovo603_f0   denovo733_f0   denovo785_f0   denovo941_f0   denovo967_f0   denovo1045_f0  denovo1071_f0  denovo1149_f0  denovo1305_f0  denovo1487_f0  denovo1513_f0  denovo1643_f0  denovo1747_f0  denovo1773_f0  denovo1799_f0  denovo1955_f0  denovo2137_f0  denovo2189_f0  denovo2241_f0  denovo2267_f0  denovo2345_f0  denovo2449_f0  denovo2475_f0  denovo2501_f0  denovo2527_f0  denovo2579_f0  denovo2605_f0  denovo2657_f0  denovo2761_f0  denovo2787_f0  denovo2839_f0  denovo2865_f0  denovo2917_f0  denovo2995_f0  denovo3047_f0  denovo3151_f0  denovo3229_f0  denovo3255_f0  denovo3281_f0  denovo3307_f0  denovo3333_f0  denovo3437_f0  denovo3489_f0  denovo3593_f0  denovo3619_f0  denovo3671_f0  denovo3697_f0  denovo3723_f0  denovo3775_f0  denovo3853_f0  denovo3957_f0  denovo3983_f0  denovo4035_f0  denovo4061_f0  denovo4191_f0  denovo4295_f0  denovo4321_f0  denovo4347_f0  denovo4399_f0  denovo4451_f0  denovo4555_f0  denovo4633_f0  denovo4737_f0  denovo4763_f0  denovo4789_f0  denovo4841_f0  denovo4893_f0  denovo4919_f0  denovo4945_f0  denovo4971_f0  denovo4997_f0  denovo5205_f0  denovo5231_f0  denovo5257_f0  denovo5387_f0  denovo5439_f0  denovo5517_f0  denovo5595_f0  denovo5621_f0  denovo5647_f0  denovo5933_f0  denovo6011_f0  denovo6063_f0  denovo6115_f0  denovo6219_f0  denovo6245_f0  denovo6271_f0  denovo6583_f0  denovo6609_f0  denovo6687_f0  denovo6713_f0  denovo6765_f0  denovo6817_f0  denovo6869_f0  denovo6999_f0  denovo7077_f0  denovo7129_f0  denovo7155_f0  denovo7259_f0  denovo7337_f0  denovo7389_f0  denovo7415_f0  denovo7519_f0  denovo7545_f0  denovo7571_f0  denovo7623_f0  denovo7675_f0  denovo7701_f0  denovo7753_f0  denovo7805_f0  denovo7987_f0  denovo8039_f0  denovo8065_f0  denovo8221_f0  denovo8247_f0  denovo8273_f0  denovo8299_f0  denovo8403_f0  denovo8429_f0  denovo8455_f0  denovo8585_f0  denovo8637_f0  denovo8663_f0  denovo8689_f0  denovo8767_f0  denovo8819_f0  denovo8845_f0  denovo8897_f0  denovo8923_f0  denovo8949_f0  denovo8975_f0  denovo9001_f0  denovo9131_f0  denovo9157_f0  denovo9183_f0  denovo9313_f0  denovo9339_f0  denovo9365_f0  denovo9391_f0  denovo9443_f0  denovo9495_f0  denovo9547_f0  denovo9599_f0  denovo9677_f0  denovo9703_f0  denovo9729_f0  denovo9807_f0  denovo9833_f0  denovo9859_f0  denovo9885_f0  denovo9937_f0  denovo9963_f0  denovo9989_f0  denovo10067_f0 denovo10171_f0 denovo10197_f0 denovo10223_f0 denovo10275_f0 denovo10301_f0 denovo10353_f0 denovo10379_f0 denovo10405_f0 denovo10431_f0 denovo10483_f0 denovo10509_f0 denovo10535_f0 denovo10613_f0 denovo10665_f0 denovo10691_f0 denovo10769_f0 denovo10847_f0 denovo10873_f0 denovo10925_f0 denovo10977_f0 denovo11029_f0 denovo11081_f0 denovo11107_f0 denovo11159_f0 denovo11185_f0 denovo11237_f0 denovo11263_f0 denovo11315_f0 denovo11341_f0 denovo11445_f0 denovo11549_f0 denovo11913_f0 denovo11965_f0 denovo12069_f0 
FG120046B                     denovo32_f0    denovo84_f0    denovo110_f0   denovo136_f0   denovo214_f0   denovo240_f0   denovo266_f0   denovo292_f0   denovo344_f0   denovo422_f0   denovo526_f0   denovo578_f0   denovo604_f0   denovo734_f0   denovo786_f0   denovo942_f0   denovo968_f0   denovo1046_f0  denovo1072_f0  denovo1150_f0  denovo1306_f0  denovo1488_f0  denovo1514_f0  denovo1644_f0  denovo1748_f0  denovo1774_f0  denovo1800_f0  denovo1956_f0  denovo2138_f0  denovo2190_f0  denovo2242_f0  denovo2268_f0  denovo2346_f0  denovo2450_f0  denovo2476_f0  denovo2502_f0  denovo2528_f0  denovo2580_f0  denovo2606_f0  denovo2658_f0  denovo2762_f0  denovo2788_f0  denovo2840_f0  denovo2866_f0  denovo2918_f0  denovo2996_f0  denovo3048_f0  denovo3152_f0  denovo3230_f0  denovo3256_f0  denovo3282_f0  denovo3308_f0  denovo3334_f0  denovo3438_f0  denovo3490_f0  denovo3594_f0  denovo3620_f0  denovo3672_f0  denovo3698_f0  denovo3724_f0  denovo3776_f0  denovo3854_f0  denovo3958_f0  denovo3984_f0  denovo4036_f0  denovo4062_f0  denovo4192_f0  denovo4296_f0  denovo4322_f0  denovo4348_f0  denovo4400_f0  denovo4452_f0  denovo4556_f0  denovo4634_f0  denovo4738_f0  denovo4764_f0  denovo4790_f0  denovo4842_f0  denovo4894_f0  denovo4920_f0  denovo4946_f0  denovo4972_f0  denovo4998_f0  denovo5206_f0  denovo5232_f0  denovo5258_f0  denovo5388_f0  denovo5440_f0  denovo5518_f0  denovo5596_f0  denovo5622_f0  denovo5648_f0  denovo5934_f0  denovo6012_f0  denovo6064_f0  denovo6116_f0  denovo6220_f0  denovo6246_f0  denovo6272_f0  denovo6584_f0  denovo6610_f0  denovo6688_f0  denovo6714_f0  denovo6766_f0  denovo6818_f0  denovo6870_f0  denovo7000_f0  denovo7078_f0  denovo7130_f0  denovo7156_f0  denovo7260_f0  denovo7338_f0  denovo7390_f0  denovo7416_f0  denovo7520_f0  denovo7546_f0  denovo7572_f0  denovo7624_f0  denovo7676_f0  denovo7702_f0  denovo7754_f0  denovo7806_f0  denovo7988_f0  denovo8040_f0  denovo8066_f0  denovo8222_f0  denovo8248_f0  denovo8274_f0  denovo8300_f0  denovo8404_f0  denovo8430_f0  denovo8456_f0  denovo8586_f0  denovo8638_f0  denovo8664_f0  denovo8690_f0  denovo8768_f0  denovo8820_f0  denovo8846_f0  denovo8898_f0  denovo8924_f0  denovo8950_f0  denovo8976_f0  denovo9002_f0  denovo9132_f0  denovo9158_f0  denovo9184_f0  denovo9314_f0  denovo9340_f0  denovo9366_f0  denovo9392_f0  denovo9444_f0  denovo9496_f0  denovo9548_f0  denovo9600_f0  denovo9678_f0  denovo9704_f0  denovo9730_f0  denovo9808_f0  denovo9834_f0  denovo9860_f0  denovo9886_f0  denovo9938_f0  denovo9964_f0  denovo9990_f0  denovo10068_f0 denovo10172_f0 denovo10198_f0 denovo10224_f0 denovo10276_f0 denovo10302_f0 denovo10354_f0 denovo10380_f0 denovo10406_f0 denovo10432_f0 denovo10484_f0 denovo10510_f0 denovo10536_f0 denovo10614_f0 denovo10666_f0 denovo10692_f0 denovo10770_f0 denovo10848_f0 denovo10874_f0 denovo10926_f0 denovo10978_f0 denovo11030_f0 denovo11082_f0 denovo11108_f0 denovo11160_f0 denovo11186_f0 denovo11238_f0 denovo11264_f0 denovo11316_f0 denovo11342_f0 denovo11446_f0 denovo11550_f0 denovo11914_f0 denovo11966_f0 denovo12070_f0 
GNV129007                     denovo41_f0    denovo93_f0    denovo119_f0   denovo145_f0   denovo223_f0   denovo249_f0   denovo275_f0   denovo301_f0   denovo353_f0   denovo431_f0   denovo535_f0   denovo587_f0   denovo613_f0   denovo743_f0   denovo795_f0   denovo951_f0   denovo977_f0   denovo1055_f0  denovo1081_f0  denovo1159_f0  denovo1315_f0  denovo1497_f0  denovo1523_f0  denovo1653_f0  denovo1757_f0  denovo1783_f0  denovo1809_f0  denovo1965_f0  denovo2147_f0  denovo2199_f0  denovo2251_f0  denovo2277_f0  denovo2355_f0  denovo2459_f0  denovo2485_f0  denovo2511_f0  denovo2537_f0  denovo2589_f0  denovo2615_f0  denovo2667_f0  denovo2771_f0  denovo2797_f0  denovo2849_f0  denovo2875_f0  denovo2927_f0  denovo3005_f0  denovo3057_f0  denovo3161_f0  denovo3239_f0  denovo3265_f0  denovo3291_f0  denovo3317_f0  denovo3343_f0  denovo3447_f0  denovo3499_f0  denovo3603_f0  denovo3629_f0  denovo3681_f0  denovo3707_f0  denovo3733_f0  denovo3785_f0  denovo3863_f0  denovo3967_f0  denovo3993_f0  denovo4045_f0  denovo4071_f0  denovo4201_f0  denovo4305_f0  denovo4331_f0  denovo4357_f0  denovo4409_f0  denovo4461_f0  denovo4565_f0  denovo4643_f0  denovo4747_f0  denovo4773_f0  denovo4799_f0  denovo4851_f0  denovo4903_f0  denovo4929_f0  denovo4955_f0  denovo4981_f0  denovo5007_f0  denovo5215_f0  denovo5241_f0  denovo5267_f0  denovo5397_f0  denovo5449_f0  denovo5527_f0  denovo5605_f0  denovo5631_f0  denovo5657_f0  denovo5943_f0  denovo6021_f0  denovo6073_f0  denovo6125_f0  denovo6229_f0  denovo6255_f0  denovo6281_f0  denovo6593_f0  denovo6619_f0  denovo6697_f0  denovo6723_f0  denovo6775_f0  denovo6827_f0  denovo6879_f0  denovo7009_f0  denovo7087_f0  denovo7139_f0  denovo7165_f0  denovo7269_f0  denovo7347_f0  denovo7399_f0  denovo7425_f0  denovo7529_f0  denovo7555_f0  denovo7581_f0  denovo7633_f0  denovo7685_f0  denovo7711_f0  denovo7763_f0  denovo7815_f0  denovo7997_f0  denovo8049_f0  denovo8075_f0  denovo8231_f0  denovo8257_f0  denovo8283_f0  denovo8309_f0  denovo8413_f0  denovo8439_f0  denovo8465_f0  denovo8595_f0  denovo8647_f0  denovo8673_f0  denovo8699_f0  denovo8777_f0  denovo8829_f0  denovo8855_f0  denovo8907_f0  denovo8933_f0  denovo8959_f0  denovo8985_f0  denovo9011_f0  denovo9141_f0  denovo9167_f0  denovo9193_f0  denovo9323_f0  denovo9349_f0  denovo9375_f0  denovo9401_f0  denovo9453_f0  denovo9505_f0  denovo9557_f0  denovo9609_f0  denovo9687_f0  denovo9713_f0  denovo9739_f0  denovo9817_f0  denovo9843_f0  denovo9869_f0  denovo9895_f0  denovo9947_f0  denovo9973_f0  denovo9999_f0  denovo10077_f0 denovo10181_f0 denovo10207_f0 denovo10233_f0 denovo10285_f0 denovo10311_f0 denovo10363_f0 denovo10389_f0 denovo10415_f0 denovo10441_f0 denovo10493_f0 denovo10519_f0 denovo10545_f0 denovo10623_f0 denovo10675_f0 denovo10701_f0 denovo10779_f0 denovo10857_f0 denovo10883_f0 denovo10935_f0 denovo10987_f0 denovo11039_f0 denovo11091_f0 denovo11117_f0 denovo11169_f0 denovo11195_f0 denovo11247_f0 denovo11273_f0 denovo11325_f0 denovo11351_f0 denovo11455_f0 denovo11559_f0 denovo11923_f0 denovo11975_f0 denovo12079_f0 
SW130126                      denovo50_f0    denovo102_f0   denovo128_f0   denovo154_f0   denovo232_f0   denovo258_f0   denovo284_f0   denovo310_f0   denovo362_f0   denovo440_f0   denovo544_f0   denovo596_f0   denovo622_f0   denovo752_f0   denovo804_f0   denovo960_f0   denovo986_f0   denovo1064_f0  denovo1090_f0  denovo1168_f0  denovo1324_f0  denovo1506_f0  denovo1532_f0  denovo1662_f0  denovo1766_f0  denovo1792_f0  denovo1818_f0  denovo1974_f0  denovo2156_f0  denovo2208_f0  denovo2260_f0  denovo2286_f0  denovo2364_f0  denovo2468_f0  denovo2494_f0  denovo2520_f0  denovo2546_f0  denovo2598_f0  denovo2624_f0  denovo2676_f0  denovo2780_f0  denovo2806_f0  denovo2858_f0  denovo2884_f0  denovo2936_f0  denovo3014_f0  denovo3066_f0  denovo3170_f0  denovo3248_f0  denovo3274_f0  denovo3300_f0  denovo3326_f0  denovo3352_f0  denovo3456_f0  denovo3508_f0  denovo3612_f0  denovo3638_f0  denovo3690_f0  denovo3716_f0  denovo3742_f0  denovo3794_f0  denovo3872_f0  denovo3976_f0  denovo4002_f0  denovo4054_f0  denovo4080_f0  denovo4210_f0  denovo4314_f0  denovo4340_f0  denovo4366_f0  denovo4418_f0  denovo4470_f0  denovo4574_f0  denovo4652_f0  denovo4756_f0  denovo4782_f0  denovo4808_f0  denovo4860_f0  denovo4912_f0  denovo4938_f0  denovo4964_f0  denovo4990_f0  denovo5016_f0  denovo5224_f0  denovo5250_f0  denovo5276_f0  denovo5406_f0  denovo5458_f0  denovo5536_f0  denovo5614_f0  denovo5640_f0  denovo5666_f0  denovo5952_f0  denovo6030_f0  denovo6082_f0  denovo6134_f0  denovo6238_f0  denovo6264_f0  denovo6290_f0  denovo6602_f0  denovo6628_f0  denovo6706_f0  denovo6732_f0  denovo6784_f0  denovo6836_f0  denovo6888_f0  denovo7018_f0  denovo7096_f0  denovo7148_f0  denovo7174_f0  denovo7278_f0  denovo7356_f0  denovo7408_f0  denovo7434_f0  denovo7538_f0  denovo7564_f0  denovo7590_f0  denovo7642_f0  denovo7694_f0  denovo7720_f0  denovo7772_f0  denovo7824_f0  denovo8006_f0  denovo8058_f0  denovo8084_f0  denovo8240_f0  denovo8266_f0  denovo8292_f0  denovo8318_f0  denovo8422_f0  denovo8448_f0  denovo8474_f0  denovo8604_f0  denovo8656_f0  denovo8682_f0  denovo8708_f0  denovo8786_f0  denovo8838_f0  denovo8864_f0  denovo8916_f0  denovo8942_f0  denovo8968_f0  denovo8994_f0  denovo9020_f0  denovo9150_f0  denovo9176_f0  denovo9202_f0  denovo9332_f0  denovo9358_f0  denovo9384_f0  denovo9410_f0  denovo9462_f0  denovo9514_f0  denovo9566_f0  denovo9618_f0  denovo9696_f0  denovo9722_f0  denovo9748_f0  denovo9826_f0  denovo9852_f0  denovo9878_f0  denovo9904_f0  denovo9956_f0  denovo9982_f0  denovo10008_f0 denovo10086_f0 denovo10190_f0 denovo10216_f0 denovo10242_f0 denovo10294_f0 denovo10320_f0 denovo10372_f0 denovo10398_f0 denovo10424_f0 denovo10450_f0 denovo10502_f0 denovo10528_f0 denovo10554_f0 denovo10632_f0 denovo10684_f0 denovo10710_f0 denovo10788_f0 denovo10866_f0 denovo10892_f0 denovo10944_f0 denovo10996_f0 denovo11048_f0 denovo11100_f0 denovo11126_f0 denovo11178_f0 denovo11204_f0 denovo11256_f0 denovo11282_f0 denovo11334_f0 denovo11360_f0 denovo11464_f0 denovo11568_f0 denovo11932_f0 denovo11984_f0 denovo12088_f0 
SW130103                      denovo49_f0    denovo101_f0   denovo127_f0   denovo153_f0   denovo231_f0   denovo257_f0   denovo283_f0   denovo309_f0   denovo361_f0   denovo439_f0   denovo543_f0   denovo595_f0   denovo621_f0   denovo751_f0   denovo803_f0   denovo959_f0   denovo985_f0   denovo1063_f0  denovo1089_f0  denovo1167_f0  denovo1323_f0  denovo1505_f0  denovo1531_f0  denovo1661_f0  denovo1765_f0  denovo1791_f0  denovo1817_f0  denovo1973_f0  denovo2155_f0  denovo2207_f0  denovo2259_f0  denovo2285_f0  denovo2363_f0  denovo2467_f0  denovo2493_f0  denovo2519_f0  denovo2545_f0  denovo2597_f0  denovo2623_f0  denovo2675_f0  denovo2779_f0  denovo2805_f0  denovo2857_f0  denovo2883_f0  denovo2935_f0  denovo3013_f0  denovo3065_f0  denovo3169_f0  denovo3247_f0  denovo3273_f0  denovo3299_f0  denovo3325_f0  denovo3351_f0  denovo3455_f0  denovo3507_f0  denovo3611_f0  denovo3637_f0  denovo3689_f0  denovo3715_f0  denovo3741_f0  denovo3793_f0  denovo3871_f0  denovo3975_f0  denovo4001_f0  denovo4053_f0  denovo4079_f0  denovo4209_f0  denovo4313_f0  denovo4339_f0  denovo4365_f0  denovo4417_f0  denovo4469_f0  denovo4573_f0  denovo4651_f0  denovo4755_f0  denovo4781_f0  denovo4807_f0  denovo4859_f0  denovo4911_f0  denovo4937_f0  denovo4963_f0  denovo4989_f0  denovo5015_f0  denovo5223_f0  denovo5249_f0  denovo5275_f0  denovo5405_f0  denovo5457_f0  denovo5535_f0  denovo5613_f0  denovo5639_f0  denovo5665_f0  denovo5951_f0  denovo6029_f0  denovo6081_f0  denovo6133_f0  denovo6237_f0  denovo6263_f0  denovo6289_f0  denovo6601_f0  denovo6627_f0  denovo6705_f0  denovo6731_f0  denovo6783_f0  denovo6835_f0  denovo6887_f0  denovo7017_f0  denovo7095_f0  denovo7147_f0  denovo7173_f0  denovo7277_f0  denovo7355_f0  denovo7407_f0  denovo7433_f0  denovo7537_f0  denovo7563_f0  denovo7589_f0  denovo7641_f0  denovo7693_f0  denovo7719_f0  denovo7771_f0  denovo7823_f0  denovo8005_f0  denovo8057_f0  denovo8083_f0  denovo8239_f0  denovo8265_f0  denovo8291_f0  denovo8317_f0  denovo8421_f0  denovo8447_f0  denovo8473_f0  denovo8603_f0  denovo8655_f0  denovo8681_f0  denovo8707_f0  denovo8785_f0  denovo8837_f0  denovo8863_f0  denovo8915_f0  denovo8941_f0  denovo8967_f0  denovo8993_f0  denovo9019_f0  denovo9149_f0  denovo9175_f0  denovo9201_f0  denovo9331_f0  denovo9357_f0  denovo9383_f0  denovo9409_f0  denovo9461_f0  denovo9513_f0  denovo9565_f0  denovo9617_f0  denovo9695_f0  denovo9721_f0  denovo9747_f0  denovo9825_f0  denovo9851_f0  denovo9877_f0  denovo9903_f0  denovo9955_f0  denovo9981_f0  denovo10007_f0 denovo10085_f0 denovo10189_f0 denovo10215_f0 denovo10241_f0 denovo10293_f0 denovo10319_f0 denovo10371_f0 denovo10397_f0 denovo10423_f0 denovo10449_f0 denovo10501_f0 denovo10527_f0 denovo10553_f0 denovo10631_f0 denovo10683_f0 denovo10709_f0 denovo10787_f0 denovo10865_f0 denovo10891_f0 denovo10943_f0 denovo10995_f0 denovo11047_f0 denovo11099_f0 denovo11125_f0 denovo11177_f0 denovo11203_f0 denovo11255_f0 denovo11281_f0 denovo11333_f0 denovo11359_f0 denovo11463_f0 denovo11567_f0 denovo11931_f0 denovo11983_f0 denovo12087_f0 
Callid                        denovo27_f0    denovo79_f0    denovo105_f0   denovo131_f0   denovo209_f0   denovo235_f0   denovo261_f0   denovo287_f0   denovo339_f0   denovo417_f0   denovo521_f0   denovo573_f0   denovo599_f0   denovo729_f0   denovo781_f0   denovo937_f0   denovo963_f0   denovo1041_f0  denovo1067_f0  denovo1145_f0  denovo1301_f0  denovo1483_f0  denovo1509_f0  denovo1639_f0  denovo1743_f0  denovo1769_f0  denovo1795_f0  denovo1951_f0  denovo2133_f0  denovo2185_f0  denovo2237_f0  denovo2263_f0  denovo2341_f0  denovo2445_f0  denovo2471_f0  denovo2497_f0  denovo2523_f0  denovo2575_f0  denovo2601_f0  denovo2653_f0  denovo2757_f0  denovo2783_f0  denovo2835_f0  denovo2861_f0  denovo2913_f0  denovo2991_f0  denovo3043_f0  denovo3147_f0  denovo3225_f0  denovo3251_f0  denovo3277_f0  denovo3303_f0  denovo3329_f0  denovo3433_f0  denovo3485_f0  denovo3589_f0  denovo3615_f0  denovo3667_f0  denovo3693_f0  denovo3719_f0  denovo3771_f0  denovo3849_f0  denovo3953_f0  denovo3979_f0  denovo4031_f0  denovo4057_f0  denovo4187_f0  denovo4291_f0  denovo4317_f0  denovo4343_f0  denovo4395_f0  denovo4447_f0  denovo4551_f0  denovo4629_f0  denovo4733_f0  denovo4759_f0  denovo4785_f0  denovo4837_f0  denovo4889_f0  denovo4915_f0  denovo4941_f0  denovo4967_f0  denovo4993_f0  denovo5201_f0  denovo5227_f0  denovo5253_f0  denovo5383_f0  denovo5435_f0  denovo5513_f0  denovo5591_f0  denovo5617_f0  denovo5643_f0  denovo5929_f0  denovo6007_f0  denovo6059_f0  denovo6111_f0  denovo6215_f0  denovo6241_f0  denovo6267_f0  denovo6579_f0  denovo6605_f0  denovo6683_f0  denovo6709_f0  denovo6761_f0  denovo6813_f0  denovo6865_f0  denovo6995_f0  denovo7073_f0  denovo7125_f0  denovo7151_f0  denovo7255_f0  denovo7333_f0  denovo7385_f0  denovo7411_f0  denovo7515_f0  denovo7541_f0  denovo7567_f0  denovo7619_f0  denovo7671_f0  denovo7697_f0  denovo7749_f0  denovo7801_f0  denovo7983_f0  denovo8035_f0  denovo8061_f0  denovo8217_f0  denovo8243_f0  denovo8269_f0  denovo8295_f0  denovo8399_f0  denovo8425_f0  denovo8451_f0  denovo8581_f0  denovo8633_f0  denovo8659_f0  denovo8685_f0  denovo8763_f0  denovo8815_f0  denovo8841_f0  denovo8893_f0  denovo8919_f0  denovo8945_f0  denovo8971_f0  denovo8997_f0  denovo9127_f0  denovo9153_f0  denovo9179_f0  denovo9309_f0  denovo9335_f0  denovo9361_f0  denovo9387_f0  denovo9439_f0  denovo9491_f0  denovo9543_f0  denovo9595_f0  denovo9673_f0  denovo9699_f0  denovo9725_f0  denovo9803_f0  denovo9829_f0  denovo9855_f0  denovo9881_f0  denovo9933_f0  denovo9959_f0  denovo9985_f0  denovo10063_f0 denovo10167_f0 denovo10193_f0 denovo10219_f0 denovo10271_f0 denovo10297_f0 denovo10349_f0 denovo10375_f0 denovo10401_f0 denovo10427_f0 denovo10479_f0 denovo10505_f0 denovo10531_f0 denovo10609_f0 denovo10661_f0 denovo10687_f0 denovo10765_f0 denovo10843_f0 denovo10869_f0 denovo10921_f0 denovo10973_f0 denovo11025_f0 denovo11077_f0 denovo11103_f0 denovo11155_f0 denovo11181_f0 denovo11233_f0 denovo11259_f0 denovo11311_f0 denovo11337_f0 denovo11441_f0 denovo11545_f0 denovo11909_f0 denovo11961_f0 denovo12065_f0 
FG120070B                     denovo34_f0    denovo86_f0    denovo112_f0   denovo138_f0   denovo216_f0   denovo242_f0   denovo268_f0   denovo294_f0   denovo346_f0   denovo424_f0   denovo528_f0   denovo580_f0   denovo606_f0   denovo736_f0   denovo788_f0   denovo944_f0   denovo970_f0   denovo1048_f0  denovo1074_f0  denovo1152_f0  denovo1308_f0  denovo1490_f0  denovo1516_f0  denovo1646_f0  denovo1750_f0  denovo1776_f0  denovo1802_f0  denovo1958_f0  denovo2140_f0  denovo2192_f0  denovo2244_f0  denovo2270_f0  denovo2348_f0  denovo2452_f0  denovo2478_f0  denovo2504_f0  denovo2530_f0  denovo2582_f0  denovo2608_f0  denovo2660_f0  denovo2764_f0  denovo2790_f0  denovo2842_f0  denovo2868_f0  denovo2920_f0  denovo2998_f0  denovo3050_f0  denovo3154_f0  denovo3232_f0  denovo3258_f0  denovo3284_f0  denovo3310_f0  denovo3336_f0  denovo3440_f0  denovo3492_f0  denovo3596_f0  denovo3622_f0  denovo3674_f0  denovo3700_f0  denovo3726_f0  denovo3778_f0  denovo3856_f0  denovo3960_f0  denovo3986_f0  denovo4038_f0  denovo4064_f0  denovo4194_f0  denovo4298_f0  denovo4324_f0  denovo4350_f0  denovo4402_f0  denovo4454_f0  denovo4558_f0  denovo4636_f0  denovo4740_f0  denovo4766_f0  denovo4792_f0  denovo4844_f0  denovo4896_f0  denovo4922_f0  denovo4948_f0  denovo4974_f0  denovo5000_f0  denovo5208_f0  denovo5234_f0  denovo5260_f0  denovo5390_f0  denovo5442_f0  denovo5520_f0  denovo5598_f0  denovo5624_f0  denovo5650_f0  denovo5936_f0  denovo6014_f0  denovo6066_f0  denovo6118_f0  denovo6222_f0  denovo6248_f0  denovo6274_f0  denovo6586_f0  denovo6612_f0  denovo6690_f0  denovo6716_f0  denovo6768_f0  denovo6820_f0  denovo6872_f0  denovo7002_f0  denovo7080_f0  denovo7132_f0  denovo7158_f0  denovo7262_f0  denovo7340_f0  denovo7392_f0  denovo7418_f0  denovo7522_f0  denovo7548_f0  denovo7574_f0  denovo7626_f0  denovo7678_f0  denovo7704_f0  denovo7756_f0  denovo7808_f0  denovo7990_f0  denovo8042_f0  denovo8068_f0  denovo8224_f0  denovo8250_f0  denovo8276_f0  denovo8302_f0  denovo8406_f0  denovo8432_f0  denovo8458_f0  denovo8588_f0  denovo8640_f0  denovo8666_f0  denovo8692_f0  denovo8770_f0  denovo8822_f0  denovo8848_f0  denovo8900_f0  denovo8926_f0  denovo8952_f0  denovo8978_f0  denovo9004_f0  denovo9134_f0  denovo9160_f0  denovo9186_f0  denovo9316_f0  denovo9342_f0  denovo9368_f0  denovo9394_f0  denovo9446_f0  denovo9498_f0  denovo9550_f0  denovo9602_f0  denovo9680_f0  denovo9706_f0  denovo9732_f0  denovo9810_f0  denovo9836_f0  denovo9862_f0  denovo9888_f0  denovo9940_f0  denovo9966_f0  denovo9992_f0  denovo10070_f0 denovo10174_f0 denovo10200_f0 denovo10226_f0 denovo10278_f0 denovo10304_f0 denovo10356_f0 denovo10382_f0 denovo10408_f0 denovo10434_f0 denovo10486_f0 denovo10512_f0 denovo10538_f0 denovo10616_f0 denovo10668_f0 denovo10694_f0 denovo10772_f0 denovo10850_f0 denovo10876_f0 denovo10928_f0 denovo10980_f0 denovo11032_f0 denovo11084_f0 denovo11110_f0 denovo11162_f0 denovo11188_f0 denovo11240_f0 denovo11266_f0 denovo11318_f0 denovo11344_f0 denovo11448_f0 denovo11552_f0 denovo11916_f0 denovo11968_f0 denovo12072_f0 
SW130007                      denovo48_f0    denovo100_f0   denovo126_f0   denovo152_f0   denovo230_f0   denovo256_f0   denovo282_f0   denovo308_f0   denovo360_f0   denovo438_f0   denovo542_f0   denovo594_f0   denovo620_f0   denovo750_f0   denovo802_f0   denovo958_f0   denovo984_f0   denovo1062_f0  denovo1088_f0  denovo1166_f0  denovo1322_f0  denovo1504_f0  denovo1530_f0  denovo1660_f0  denovo1764_f0  denovo1790_f0  denovo1816_f0  denovo1972_f0  denovo2154_f0  denovo2206_f0  denovo2258_f0  denovo2284_f0  denovo2362_f0  denovo2466_f0  denovo2492_f0  denovo2518_f0  denovo2544_f0  denovo2596_f0  denovo2622_f0  denovo2674_f0  denovo2778_f0  denovo2804_f0  denovo2856_f0  denovo2882_f0  denovo2934_f0  denovo3012_f0  denovo3064_f0  denovo3168_f0  denovo3246_f0  denovo3272_f0  denovo3298_f0  denovo3324_f0  denovo3350_f0  denovo3454_f0  denovo3506_f0  denovo3610_f0  denovo3636_f0  denovo3688_f0  denovo3714_f0  denovo3740_f0  denovo3792_f0  denovo3870_f0  denovo3974_f0  denovo4000_f0  denovo4052_f0  denovo4078_f0  denovo4208_f0  denovo4312_f0  denovo4338_f0  denovo4364_f0  denovo4416_f0  denovo4468_f0  denovo4572_f0  denovo4650_f0  denovo4754_f0  denovo4780_f0  denovo4806_f0  denovo4858_f0  denovo4910_f0  denovo4936_f0  denovo4962_f0  denovo4988_f0  denovo5014_f0  denovo5222_f0  denovo5248_f0  denovo5274_f0  denovo5404_f0  denovo5456_f0  denovo5534_f0  denovo5612_f0  denovo5638_f0  denovo5664_f0  denovo5950_f0  denovo6028_f0  denovo6080_f0  denovo6132_f0  denovo6236_f0  denovo6262_f0  denovo6288_f0  denovo6600_f0  denovo6626_f0  denovo6704_f0  denovo6730_f0  denovo6782_f0  denovo6834_f0  denovo6886_f0  denovo7016_f0  denovo7094_f0  denovo7146_f0  denovo7172_f0  denovo7276_f0  denovo7354_f0  denovo7406_f0  denovo7432_f0  denovo7536_f0  denovo7562_f0  denovo7588_f0  denovo7640_f0  denovo7692_f0  denovo7718_f0  denovo7770_f0  denovo7822_f0  denovo8004_f0  denovo8056_f0  denovo8082_f0  denovo8238_f0  denovo8264_f0  denovo8290_f0  denovo8316_f0  denovo8420_f0  denovo8446_f0  denovo8472_f0  denovo8602_f0  denovo8654_f0  denovo8680_f0  denovo8706_f0  denovo8784_f0  denovo8836_f0  denovo8862_f0  denovo8914_f0  denovo8940_f0  denovo8966_f0  denovo8992_f0  denovo9018_f0  denovo9148_f0  denovo9174_f0  denovo9200_f0  denovo9330_f0  denovo9356_f0  denovo9382_f0  denovo9408_f0  denovo9460_f0  denovo9512_f0  denovo9564_f0  denovo9616_f0  denovo9694_f0  denovo9720_f0  denovo9746_f0  denovo9824_f0  denovo9850_f0  denovo9876_f0  denovo9902_f0  denovo9954_f0  denovo9980_f0  denovo10006_f0 denovo10084_f0 denovo10188_f0 denovo10214_f0 denovo10240_f0 denovo10292_f0 denovo10318_f0 denovo10370_f0 denovo10396_f0 denovo10422_f0 denovo10448_f0 denovo10500_f0 denovo10526_f0 denovo10552_f0 denovo10630_f0 denovo10682_f0 denovo10708_f0 denovo10786_f0 denovo10864_f0 denovo10890_f0 denovo10942_f0 denovo10994_f0 denovo11046_f0 denovo11098_f0 denovo11124_f0 denovo11176_f0 denovo11202_f0 denovo11254_f0 denovo11280_f0 denovo11332_f0 denovo11358_f0 denovo11462_f0 denovo11566_f0 denovo11930_f0 denovo11982_f0 denovo12086_f0 
GNV120032                     denovo40_f0    denovo92_f0    denovo118_f0   denovo144_f0   denovo222_f0   denovo248_f0   denovo274_f0   denovo300_f0   denovo352_f0   denovo430_f0   denovo534_f0   denovo586_f0   denovo612_f0   denovo742_f0   denovo794_f0   denovo950_f0   denovo976_f0   denovo1054_f0  denovo1080_f0  denovo1158_f0  denovo1314_f0  denovo1496_f0  denovo1522_f0  denovo1652_f0  denovo1756_f0  denovo1782_f0  denovo1808_f0  denovo1964_f0  denovo2146_f0  denovo2198_f0  denovo2250_f0  denovo2276_f0  denovo2354_f0  denovo2458_f0  denovo2484_f0  denovo2510_f0  denovo2536_f0  denovo2588_f0  denovo2614_f0  denovo2666_f0  denovo2770_f0  denovo2796_f0  denovo2848_f0  denovo2874_f0  denovo2926_f0  denovo3004_f0  denovo3056_f0  denovo3160_f0  denovo3238_f0  denovo3264_f0  denovo3290_f0  denovo3316_f0  denovo3342_f0  denovo3446_f0  denovo3498_f0  denovo3602_f0  denovo3628_f0  denovo3680_f0  denovo3706_f0  denovo3732_f0  denovo3784_f0  denovo3862_f0  denovo3966_f0  denovo3992_f0  denovo4044_f0  denovo4070_f0  denovo4200_f0  denovo4304_f0  denovo4330_f0  denovo4356_f0  denovo4408_f0  denovo4460_f0  denovo4564_f0  denovo4642_f0  denovo4746_f0  denovo4772_f0  denovo4798_f0  denovo4850_f0  denovo4902_f0  denovo4928_f0  denovo4954_f0  denovo4980_f0  denovo5006_f0  denovo5214_f0  denovo5240_f0  denovo5266_f0  denovo5396_f0  denovo5448_f0  denovo5526_f0  denovo5604_f0  denovo5630_f0  denovo5656_f0  denovo5942_f0  denovo6020_f0  denovo6072_f0  denovo6124_f0  denovo6228_f0  denovo6254_f0  denovo6280_f0  denovo6592_f0  denovo6618_f0  denovo6696_f0  denovo6722_f0  denovo6774_f0  denovo6826_f0  denovo6878_f0  denovo7008_f0  denovo7086_f0  denovo7138_f0  denovo7164_f0  denovo7268_f0  denovo7346_f0  denovo7398_f0  denovo7424_f0  denovo7528_f0  denovo7554_f0  denovo7580_f0  denovo7632_f0  denovo7684_f0  denovo7710_f0  denovo7762_f0  denovo7814_f0  denovo7996_f0  denovo8048_f0  denovo8074_f0  denovo8230_f0  denovo8256_f0  denovo8282_f0  denovo8308_f0  denovo8412_f0  denovo8438_f0  denovo8464_f0  denovo8594_f0  denovo8646_f0  denovo8672_f0  denovo8698_f0  denovo8776_f0  denovo8828_f0  denovo8854_f0  denovo8906_f0  denovo8932_f0  denovo8958_f0  denovo8984_f0  denovo9010_f0  denovo9140_f0  denovo9166_f0  denovo9192_f0  denovo9322_f0  denovo9348_f0  denovo9374_f0  denovo9400_f0  denovo9452_f0  denovo9504_f0  denovo9556_f0  denovo9608_f0  denovo9686_f0  denovo9712_f0  denovo9738_f0  denovo9816_f0  denovo9842_f0  denovo9868_f0  denovo9894_f0  denovo9946_f0  denovo9972_f0  denovo9998_f0  denovo10076_f0 denovo10180_f0 denovo10206_f0 denovo10232_f0 denovo10284_f0 denovo10310_f0 denovo10362_f0 denovo10388_f0 denovo10414_f0 denovo10440_f0 denovo10492_f0 denovo10518_f0 denovo10544_f0 denovo10622_f0 denovo10674_f0 denovo10700_f0 denovo10778_f0 denovo10856_f0 denovo10882_f0 denovo10934_f0 denovo10986_f0 denovo11038_f0 denovo11090_f0 denovo11116_f0 denovo11168_f0 denovo11194_f0 denovo11246_f0 denovo11272_f0 denovo11324_f0 denovo11350_f0 denovo11454_f0 denovo11558_f0 denovo11922_f0 denovo11974_f0 denovo12078_f0 
PXYLO                         denovo44_f0    denovo96_f0    denovo122_f0   denovo148_f0   denovo226_f0   denovo252_f0   denovo278_f0   denovo304_f0   denovo356_f0   denovo434_f0   denovo538_f0   denovo590_f0   denovo616_f0   denovo746_f0   denovo798_f0   denovo954_f0   denovo980_f0   denovo1058_f0  denovo1084_f0  denovo1162_f0  denovo1318_f0  denovo1500_f0  denovo1526_f0  denovo1656_f0  denovo1760_f0  denovo1786_f0  denovo1812_f0  denovo1968_f0  denovo2150_f0  denovo2202_f0  denovo2254_f0  denovo2280_f0  denovo2358_f0  denovo2462_f0  denovo2488_f0  denovo2514_f0  denovo2540_f0  denovo2592_f0  denovo2618_f0  denovo2670_f0  denovo2774_f0  denovo2800_f0  denovo2852_f0  denovo2878_f0  denovo2930_f0  denovo3008_f0  denovo3060_f0  denovo3164_f0  denovo3242_f0  denovo3268_f0  denovo3294_f0  denovo3320_f0  denovo3346_f0  denovo3450_f0  denovo3502_f0  denovo3606_f0  denovo3632_f0  denovo3684_f0  denovo3710_f0  denovo3736_f0  denovo3788_f0  denovo3866_f0  denovo3970_f0  denovo3996_f0  denovo4048_f0  denovo4074_f0  denovo4204_f0  denovo4308_f0  denovo4334_f0  denovo4360_f0  denovo4412_f0  denovo4464_f0  denovo4568_f0  denovo4646_f0  denovo4750_f0  denovo4776_f0  denovo4802_f0  denovo4854_f0  denovo4906_f0  denovo4932_f0  denovo4958_f0  denovo4984_f0  denovo5010_f0  denovo5218_f0  denovo5244_f0  denovo5270_f0  denovo5400_f0  denovo5452_f0  denovo5530_f0  denovo5608_f0  denovo5634_f0  denovo5660_f0  denovo5946_f0  denovo6024_f0  denovo6076_f0  denovo6128_f0  denovo6232_f0  denovo6258_f0  denovo6284_f0  denovo6596_f0  denovo6622_f0  denovo6700_f0  denovo6726_f0  denovo6778_f0  denovo6830_f0  denovo6882_f0  denovo7012_f0  denovo7090_f0  denovo7142_f0  denovo7168_f0  denovo7272_f0  denovo7350_f0  denovo7402_f0  denovo7428_f0  denovo7532_f0  denovo7558_f0  denovo7584_f0  denovo7636_f0  denovo7688_f0  denovo7714_f0  denovo7766_f0  denovo7818_f0  denovo8000_f0  denovo8052_f0  denovo8078_f0  denovo8234_f0  denovo8260_f0  denovo8286_f0  denovo8312_f0  denovo8416_f0  denovo8442_f0  denovo8468_f0  denovo8598_f0  denovo8650_f0  denovo8676_f0  denovo8702_f0  denovo8780_f0  denovo8832_f0  denovo8858_f0  denovo8910_f0  denovo8936_f0  denovo8962_f0  denovo8988_f0  denovo9014_f0  denovo9144_f0  denovo9170_f0  denovo9196_f0  denovo9326_f0  denovo9352_f0  denovo9378_f0  denovo9404_f0  denovo9456_f0  denovo9508_f0  denovo9560_f0  denovo9612_f0  denovo9690_f0  denovo9716_f0  denovo9742_f0  denovo9820_f0  denovo9846_f0  denovo9872_f0  denovo9898_f0  denovo9950_f0  denovo9976_f0  denovo10002_f0 denovo10080_f0 denovo10184_f0 denovo10210_f0 denovo10236_f0 denovo10288_f0 denovo10314_f0 denovo10366_f0 denovo10392_f0 denovo10418_f0 denovo10444_f0 denovo10496_f0 denovo10522_f0 denovo10548_f0 denovo10626_f0 denovo10678_f0 denovo10704_f0 denovo10782_f0 denovo10860_f0 denovo10886_f0 denovo10938_f0 denovo10990_f0 denovo11042_f0 denovo11094_f0 denovo11120_f0 denovo11172_f0 denovo11198_f0 denovo11250_f0 denovo11276_f0 denovo11328_f0 denovo11354_f0 denovo11458_f0 denovo11562_f0 denovo11926_f0 denovo11978_f0 denovo12082_f0 
FG120079                      denovo37_f0    denovo89_f0    denovo115_f0   denovo141_f0   denovo219_f0   denovo245_f0   denovo271_f0   denovo297_f0   denovo349_f0   denovo427_f0   denovo531_f0   denovo583_f0   denovo609_f0   denovo739_f0   denovo791_f0   denovo947_f0   denovo973_f0   denovo1051_f0  denovo1077_f0  denovo1155_f0  denovo1311_f0  denovo1493_f0  denovo1519_f0  denovo1649_f0  denovo1753_f0  denovo1779_f0  denovo1805_f0  denovo1961_f0  denovo2143_f0  denovo2195_f0  denovo2247_f0  denovo2273_f0  denovo2351_f0  denovo2455_f0  denovo2481_f0  denovo2507_f0  denovo2533_f0  denovo2585_f0  denovo2611_f0  denovo2663_f0  denovo2767_f0  denovo2793_f0  denovo2845_f0  denovo2871_f0  denovo2923_f0  denovo3001_f0  denovo3053_f0  denovo3157_f0  denovo3235_f0  denovo3261_f0  denovo3287_f0  denovo3313_f0  denovo3339_f0  denovo3443_f0  denovo3495_f0  denovo3599_f0  denovo3625_f0  denovo3677_f0  denovo3703_f0  denovo3729_f0  denovo3781_f0  denovo3859_f0  denovo3963_f0  denovo3989_f0  denovo4041_f0  denovo4067_f0  denovo4197_f0  denovo4301_f0  denovo4327_f0  denovo4353_f0  denovo4405_f0  denovo4457_f0  denovo4561_f0  denovo4639_f0  denovo4743_f0  denovo4769_f0  denovo4795_f0  denovo4847_f0  denovo4899_f0  denovo4925_f0  denovo4951_f0  denovo4977_f0  denovo5003_f0  denovo5211_f0  denovo5237_f0  denovo5263_f0  denovo5393_f0  denovo5445_f0  denovo5523_f0  denovo5601_f0  denovo5627_f0  denovo5653_f0  denovo5939_f0  denovo6017_f0  denovo6069_f0  denovo6121_f0  denovo6225_f0  denovo6251_f0  denovo6277_f0  denovo6589_f0  denovo6615_f0  denovo6693_f0  denovo6719_f0  denovo6771_f0  denovo6823_f0  denovo6875_f0  denovo7005_f0  denovo7083_f0  denovo7135_f0  denovo7161_f0  denovo7265_f0  denovo7343_f0  denovo7395_f0  denovo7421_f0  denovo7525_f0  denovo7551_f0  denovo7577_f0  denovo7629_f0  denovo7681_f0  denovo7707_f0  denovo7759_f0  denovo7811_f0  denovo7993_f0  denovo8045_f0  denovo8071_f0  denovo8227_f0  denovo8253_f0  denovo8279_f0  denovo8305_f0  denovo8409_f0  denovo8435_f0  denovo8461_f0  denovo8591_f0  denovo8643_f0  denovo8669_f0  denovo8695_f0  denovo8773_f0  denovo8825_f0  denovo8851_f0  denovo8903_f0  denovo8929_f0  denovo8955_f0  denovo8981_f0  denovo9007_f0  denovo9137_f0  denovo9163_f0  denovo9189_f0  denovo9319_f0  denovo9345_f0  denovo9371_f0  denovo9397_f0  denovo9449_f0  denovo9501_f0  denovo9553_f0  denovo9605_f0  denovo9683_f0  denovo9709_f0  denovo9735_f0  denovo9813_f0  denovo9839_f0  denovo9865_f0  denovo9891_f0  denovo9943_f0  denovo9969_f0  denovo9995_f0  denovo10073_f0 denovo10177_f0 denovo10203_f0 denovo10229_f0 denovo10281_f0 denovo10307_f0 denovo10359_f0 denovo10385_f0 denovo10411_f0 denovo10437_f0 denovo10489_f0 denovo10515_f0 denovo10541_f0 denovo10619_f0 denovo10671_f0 denovo10697_f0 denovo10775_f0 denovo10853_f0 denovo10879_f0 denovo10931_f0 denovo10983_f0 denovo11035_f0 denovo11087_f0 denovo11113_f0 denovo11165_f0 denovo11191_f0 denovo11243_f0 denovo11269_f0 denovo11321_f0 denovo11347_f0 denovo11451_f0 denovo11555_f0 denovo11919_f0 denovo11971_f0 denovo12075_f0 
SRR850324                     denovo47_f0    denovo99_f0    denovo125_f0   denovo151_f0   denovo229_f0   denovo255_f0   denovo281_f0   denovo307_f0   denovo359_f0   denovo437_f0   denovo541_f0   denovo593_f0   denovo619_f0   denovo749_f0   denovo801_f0   denovo957_f0   denovo983_f0   denovo1061_f0  denovo1087_f0  denovo1165_f0  denovo1321_f0  denovo1503_f0  denovo1529_f0  denovo1659_f0  denovo1763_f0  denovo1789_f0  denovo1815_f0  denovo1971_f0  denovo2153_f0  denovo2205_f0  denovo2257_f0  denovo2283_f0  denovo2361_f0  denovo2465_f0  denovo2491_f0  denovo2517_f0  denovo2543_f0  denovo2595_f0  denovo2621_f0  denovo2673_f0  denovo2777_f0  denovo2803_f0  denovo2855_f0  denovo2881_f0  denovo2933_f0  denovo3011_f0  denovo3063_f0  denovo3167_f0  denovo3245_f0  denovo3271_f0  denovo3297_f0  denovo3323_f0  denovo3349_f0  denovo3453_f0  denovo3505_f0  denovo3609_f0  denovo3635_f0  denovo3687_f0  denovo3713_f0  denovo3739_f0  denovo3791_f0  denovo3869_f0  denovo3973_f0  denovo3999_f0  denovo4051_f0  denovo4077_f0  denovo4207_f0  denovo4311_f0  denovo4337_f0  denovo4363_f0  denovo4415_f0  denovo4467_f0  denovo4571_f0  denovo4649_f0  denovo4753_f0  denovo4779_f0  denovo4805_f0  denovo4857_f0  denovo4909_f0  denovo4935_f0  denovo4961_f0  denovo4987_f0  denovo5013_f0  denovo5221_f0  denovo5247_f0  denovo5273_f0  denovo5403_f0  denovo5455_f0  denovo5533_f0  denovo5611_f0  denovo5637_f0  denovo5663_f0  denovo5949_f0  denovo6027_f0  denovo6079_f0  denovo6131_f0  denovo6235_f0  denovo6261_f0  denovo6287_f0  denovo6599_f0  denovo6625_f0  denovo6703_f0  denovo6729_f0  denovo6781_f0  denovo6833_f0  denovo6885_f0  denovo7015_f0  denovo7093_f0  denovo7145_f0  denovo7171_f0  denovo7275_f0  denovo7353_f0  denovo7405_f0  denovo7431_f0  denovo7535_f0  denovo7561_f0  denovo7587_f0  denovo7639_f0  denovo7691_f0  denovo7717_f0  denovo7769_f0  denovo7821_f0  denovo8003_f0  denovo8055_f0  denovo8081_f0  denovo8237_f0  denovo8263_f0  denovo8289_f0  denovo8315_f0  denovo8419_f0  denovo8445_f0  denovo8471_f0  denovo8601_f0  denovo8653_f0  denovo8679_f0  denovo8705_f0  denovo8783_f0  denovo8835_f0  denovo8861_f0  denovo8913_f0  denovo8939_f0  denovo8965_f0  denovo8991_f0  denovo9017_f0  denovo9147_f0  denovo9173_f0  denovo9199_f0  denovo9329_f0  denovo9355_f0  denovo9381_f0  denovo9407_f0  denovo9459_f0  denovo9511_f0  denovo9563_f0  denovo9615_f0  denovo9693_f0  denovo9719_f0  denovo9745_f0  denovo9823_f0  denovo9849_f0  denovo9875_f0  denovo9901_f0  denovo9953_f0  denovo9979_f0  denovo10005_f0 denovo10083_f0 denovo10187_f0 denovo10213_f0 denovo10239_f0 denovo10291_f0 denovo10317_f0 denovo10369_f0 denovo10395_f0 denovo10421_f0 denovo10447_f0 denovo10499_f0 denovo10525_f0 denovo10551_f0 denovo10629_f0 denovo10681_f0 denovo10707_f0 denovo10785_f0 denovo10863_f0 denovo10889_f0 denovo10941_f0 denovo10993_f0 denovo11045_f0 denovo11097_f0 denovo11123_f0 denovo11175_f0 denovo11201_f0 denovo11253_f0 denovo11279_f0 denovo11331_f0 denovo11357_f0 denovo11461_f0 denovo11565_f0 denovo11929_f0 denovo11981_f0 denovo12085_f0 
FG120055B                     denovo33_f0    denovo85_f0    denovo111_f0   denovo137_f0   denovo215_f0   denovo241_f0   denovo267_f0   denovo293_f0   denovo345_f0   denovo423_f0   denovo527_f0   denovo579_f0   denovo605_f0   denovo735_f0   denovo787_f0   denovo943_f0   denovo969_f0   denovo1047_f0  denovo1073_f0  denovo1151_f0  denovo1307_f0  denovo1489_f0  denovo1515_f0  denovo1645_f0  denovo1749_f0  denovo1775_f0  denovo1801_f0  denovo1957_f0  denovo2139_f0  denovo2191_f0  denovo2243_f0  denovo2269_f0  denovo2347_f0  denovo2451_f0  denovo2477_f0  denovo2503_f0  denovo2529_f0  denovo2581_f0  denovo2607_f0  denovo2659_f0  denovo2763_f0  denovo2789_f0  denovo2841_f0  denovo2867_f0  denovo2919_f0  denovo2997_f0  denovo3049_f0  denovo3153_f0  denovo3231_f0  denovo3257_f0  denovo3283_f0  denovo3309_f0  denovo3335_f0  denovo3439_f0  denovo3491_f0  denovo3595_f0  denovo3621_f0  denovo3673_f0  denovo3699_f0  denovo3725_f0  denovo3777_f0  denovo3855_f0  denovo3959_f0  denovo3985_f0  denovo4037_f0  denovo4063_f0  denovo4193_f0  denovo4297_f0  denovo4323_f0  denovo4349_f0  denovo4401_f0  denovo4453_f0  denovo4557_f0  denovo4635_f0  denovo4739_f0  denovo4765_f0  denovo4791_f0  denovo4843_f0  denovo4895_f0  denovo4921_f0  denovo4947_f0  denovo4973_f0  denovo4999_f0  denovo5207_f0  denovo5233_f0  denovo5259_f0  denovo5389_f0  denovo5441_f0  denovo5519_f0  denovo5597_f0  denovo5623_f0  denovo5649_f0  denovo5935_f0  denovo6013_f0  denovo6065_f0  denovo6117_f0  denovo6221_f0  denovo6247_f0  denovo6273_f0  denovo6585_f0  denovo6611_f0  denovo6689_f0  denovo6715_f0  denovo6767_f0  denovo6819_f0  denovo6871_f0  denovo7001_f0  denovo7079_f0  denovo7131_f0  denovo7157_f0  denovo7261_f0  denovo7339_f0  denovo7391_f0  denovo7417_f0  denovo7521_f0  denovo7547_f0  denovo7573_f0  denovo7625_f0  denovo7677_f0  denovo7703_f0  denovo7755_f0  denovo7807_f0  denovo7989_f0  denovo8041_f0  denovo8067_f0  denovo8223_f0  denovo8249_f0  denovo8275_f0  denovo8301_f0  denovo8405_f0  denovo8431_f0  denovo8457_f0  denovo8587_f0  denovo8639_f0  denovo8665_f0  denovo8691_f0  denovo8769_f0  denovo8821_f0  denovo8847_f0  denovo8899_f0  denovo8925_f0  denovo8951_f0  denovo8977_f0  denovo9003_f0  denovo9133_f0  denovo9159_f0  denovo9185_f0  denovo9315_f0  denovo9341_f0  denovo9367_f0  denovo9393_f0  denovo9445_f0  denovo9497_f0  denovo9549_f0  denovo9601_f0  denovo9679_f0  denovo9705_f0  denovo9731_f0  denovo9809_f0  denovo9835_f0  denovo9861_f0  denovo9887_f0  denovo9939_f0  denovo9965_f0  denovo9991_f0  denovo10069_f0 denovo10173_f0 denovo10199_f0 denovo10225_f0 denovo10277_f0 denovo10303_f0 denovo10355_f0 denovo10381_f0 denovo10407_f0 denovo10433_f0 denovo10485_f0 denovo10511_f0 denovo10537_f0 denovo10615_f0 denovo10667_f0 denovo10693_f0 denovo10771_f0 denovo10849_f0 denovo10875_f0 denovo10927_f0 denovo10979_f0 denovo11031_f0 denovo11083_f0 denovo11109_f0 denovo11161_f0 denovo11187_f0 denovo11239_f0 denovo11265_f0 denovo11317_f0 denovo11343_f0 denovo11447_f0 denovo11551_f0 denovo11915_f0 denovo11967_f0 denovo12071_f0 
FG120122                      denovo38_f0    denovo90_f0    denovo116_f0   denovo142_f0   denovo220_f0   denovo246_f0   denovo272_f0   denovo298_f0   denovo350_f0   denovo428_f0   denovo532_f0   denovo584_f0   denovo610_f0   denovo740_f0   denovo792_f0   denovo948_f0   denovo974_f0   denovo1052_f0  denovo1078_f0  denovo1156_f0  denovo1312_f0  denovo1494_f0  denovo1520_f0  denovo1650_f0  denovo1754_f0  denovo1780_f0  denovo1806_f0  denovo1962_f0  denovo2144_f0  denovo2196_f0  denovo2248_f0  denovo2274_f0  denovo2352_f0  denovo2456_f0  denovo2482_f0  denovo2508_f0  denovo2534_f0  denovo2586_f0  denovo2612_f0  denovo2664_f0  denovo2768_f0  denovo2794_f0  denovo2846_f0  denovo2872_f0  denovo2924_f0  denovo3002_f0  denovo3054_f0  denovo3158_f0  denovo3236_f0  denovo3262_f0  denovo3288_f0  denovo3314_f0  denovo3340_f0  denovo3444_f0  denovo3496_f0  denovo3600_f0  denovo3626_f0  denovo3678_f0  denovo3704_f0  denovo3730_f0  denovo3782_f0  denovo3860_f0  denovo3964_f0  denovo3990_f0  denovo4042_f0  denovo4068_f0  denovo4198_f0  denovo4302_f0  denovo4328_f0  denovo4354_f0  denovo4406_f0  denovo4458_f0  denovo4562_f0  denovo4640_f0  denovo4744_f0  denovo4770_f0  denovo4796_f0  denovo4848_f0  denovo4900_f0  denovo4926_f0  denovo4952_f0  denovo4978_f0  denovo5004_f0  denovo5212_f0  denovo5238_f0  denovo5264_f0  denovo5394_f0  denovo5446_f0  denovo5524_f0  denovo5602_f0  denovo5628_f0  denovo5654_f0  denovo5940_f0  denovo6018_f0  denovo6070_f0  denovo6122_f0  denovo6226_f0  denovo6252_f0  denovo6278_f0  denovo6590_f0  denovo6616_f0  denovo6694_f0  denovo6720_f0  denovo6772_f0  denovo6824_f0  denovo6876_f0  denovo7006_f0  denovo7084_f0  denovo7136_f0  denovo7162_f0  denovo7266_f0  denovo7344_f0  denovo7396_f0  denovo7422_f0  denovo7526_f0  denovo7552_f0  denovo7578_f0  denovo7630_f0  denovo7682_f0  denovo7708_f0  denovo7760_f0  denovo7812_f0  denovo7994_f0  denovo8046_f0  denovo8072_f0  denovo8228_f0  denovo8254_f0  denovo8280_f0  denovo8306_f0  denovo8410_f0  denovo8436_f0  denovo8462_f0  denovo8592_f0  denovo8644_f0  denovo8670_f0  denovo8696_f0  denovo8774_f0  denovo8826_f0  denovo8852_f0  denovo8904_f0  denovo8930_f0  denovo8956_f0  denovo8982_f0  denovo9008_f0  denovo9138_f0  denovo9164_f0  denovo9190_f0  denovo9320_f0  denovo9346_f0  denovo9372_f0  denovo9398_f0  denovo9450_f0  denovo9502_f0  denovo9554_f0  denovo9606_f0  denovo9684_f0  denovo9710_f0  denovo9736_f0  denovo9814_f0  denovo9840_f0  denovo9866_f0  denovo9892_f0  denovo9944_f0  denovo9970_f0  denovo9996_f0  denovo10074_f0 denovo10178_f0 denovo10204_f0 denovo10230_f0 denovo10282_f0 denovo10308_f0 denovo10360_f0 denovo10386_f0 denovo10412_f0 denovo10438_f0 denovo10490_f0 denovo10516_f0 denovo10542_f0 denovo10620_f0 denovo10672_f0 denovo10698_f0 denovo10776_f0 denovo10854_f0 denovo10880_f0 denovo10932_f0 denovo10984_f0 denovo11036_f0 denovo11088_f0 denovo11114_f0 denovo11166_f0 denovo11192_f0 denovo11244_f0 denovo11270_f0 denovo11322_f0 denovo11348_f0 denovo11452_f0 denovo11556_f0 denovo11920_f0 denovo11972_f0 denovo12076_f0 
FG120024                      denovo30_f0    denovo82_f0    denovo108_f0   denovo134_f0   denovo212_f0   denovo238_f0   denovo264_f0   denovo290_f0   denovo342_f0   denovo420_f0   denovo524_f0   denovo576_f0   denovo602_f0   denovo732_f0   denovo784_f0   denovo940_f0   denovo966_f0   denovo1044_f0  denovo1070_f0  denovo1148_f0  denovo1304_f0  denovo1486_f0  denovo1512_f0  denovo1642_f0  denovo1746_f0  denovo1772_f0  denovo1798_f0  denovo1954_f0  denovo2136_f0  denovo2188_f0  denovo2240_f0  denovo2266_f0  denovo2344_f0  denovo2448_f0  denovo2474_f0  denovo2500_f0  denovo2526_f0  denovo2578_f0  denovo2604_f0  denovo2656_f0  denovo2760_f0  denovo2786_f0  denovo2838_f0  denovo2864_f0  denovo2916_f0  denovo2994_f0  denovo3046_f0  denovo3150_f0  denovo3228_f0  denovo3254_f0  denovo3280_f0  denovo3306_f0  denovo3332_f0  denovo3436_f0  denovo3488_f0  denovo3592_f0  denovo3618_f0  denovo3670_f0  denovo3696_f0  denovo3722_f0  denovo3774_f0  denovo3852_f0  denovo3956_f0  denovo3982_f0  denovo4034_f0  denovo4060_f0  denovo4190_f0  denovo4294_f0  denovo4320_f0  denovo4346_f0  denovo4398_f0  denovo4450_f0  denovo4554_f0  denovo4632_f0  denovo4736_f0  denovo4762_f0  denovo4788_f0  denovo4840_f0  denovo4892_f0  denovo4918_f0  denovo4944_f0  denovo4970_f0  denovo4996_f0  denovo5204_f0  denovo5230_f0  denovo5256_f0  denovo5386_f0  denovo5438_f0  denovo5516_f0  denovo5594_f0  denovo5620_f0  denovo5646_f0  denovo5932_f0  denovo6010_f0  denovo6062_f0  denovo6114_f0  denovo6218_f0  denovo6244_f0  denovo6270_f0  denovo6582_f0  denovo6608_f0  denovo6686_f0  denovo6712_f0  denovo6764_f0  denovo6816_f0  denovo6868_f0  denovo6998_f0  denovo7076_f0  denovo7128_f0  denovo7154_f0  denovo7258_f0  denovo7336_f0  denovo7388_f0  denovo7414_f0  denovo7518_f0  denovo7544_f0  denovo7570_f0  denovo7622_f0  denovo7674_f0  denovo7700_f0  denovo7752_f0  denovo7804_f0  denovo7986_f0  denovo8038_f0  denovo8064_f0  denovo8220_f0  denovo8246_f0  denovo8272_f0  denovo8298_f0  denovo8402_f0  denovo8428_f0  denovo8454_f0  denovo8584_f0  denovo8636_f0  denovo8662_f0  denovo8688_f0  denovo8766_f0  denovo8818_f0  denovo8844_f0  denovo8896_f0  denovo8922_f0  denovo8948_f0  denovo8974_f0  denovo9000_f0  denovo9130_f0  denovo9156_f0  denovo9182_f0  denovo9312_f0  denovo9338_f0  denovo9364_f0  denovo9390_f0  denovo9442_f0  denovo9494_f0  denovo9546_f0  denovo9598_f0  denovo9676_f0  denovo9702_f0  denovo9728_f0  denovo9806_f0  denovo9832_f0  denovo9858_f0  denovo9884_f0  denovo9936_f0  denovo9962_f0  denovo9988_f0  denovo10066_f0 denovo10170_f0 denovo10196_f0 denovo10222_f0 denovo10274_f0 denovo10300_f0 denovo10352_f0 denovo10378_f0 denovo10404_f0 denovo10430_f0 denovo10482_f0 denovo10508_f0 denovo10534_f0 denovo10612_f0 denovo10664_f0 denovo10690_f0 denovo10768_f0 denovo10846_f0 denovo10872_f0 denovo10924_f0 denovo10976_f0 denovo11028_f0 denovo11080_f0 denovo11106_f0 denovo11158_f0 denovo11184_f0 denovo11236_f0 denovo11262_f0 denovo11314_f0 denovo11340_f0 denovo11444_f0 denovo11548_f0 denovo11912_f0 denovo11964_f0 denovo12068_f0 
FG120022                      denovo29_f0    denovo81_f0    denovo107_f0   denovo133_f0   denovo211_f0   denovo237_f0   denovo263_f0   denovo289_f0   denovo341_f0   denovo419_f0   denovo523_f0   denovo575_f0   denovo601_f0   denovo731_f0   denovo783_f0   denovo939_f0   denovo965_f0   denovo1043_f0  denovo1069_f0  denovo1147_f0  denovo1303_f0  denovo1485_f0  denovo1511_f0  denovo1641_f0  denovo1745_f0  denovo1771_f0  denovo1797_f0  denovo1953_f0  denovo2135_f0  denovo2187_f0  denovo2239_f0  denovo2265_f0  denovo2343_f0  denovo2447_f0  denovo2473_f0  denovo2499_f0  denovo2525_f0  denovo2577_f0  denovo2603_f0  denovo2655_f0  denovo2759_f0  denovo2785_f0  denovo2837_f0  denovo2863_f0  denovo2915_f0  denovo2993_f0  denovo3045_f0  denovo3149_f0  denovo3227_f0  denovo3253_f0  denovo3279_f0  denovo3305_f0  denovo3331_f0  denovo3435_f0  denovo3487_f0  denovo3591_f0  denovo3617_f0  denovo3669_f0  denovo3695_f0  denovo3721_f0  denovo3773_f0  denovo3851_f0  denovo3955_f0  denovo3981_f0  denovo4033_f0  denovo4059_f0  denovo4189_f0  denovo4293_f0  denovo4319_f0  denovo4345_f0  denovo4397_f0  denovo4449_f0  denovo4553_f0  denovo4631_f0  denovo4735_f0  denovo4761_f0  denovo4787_f0  denovo4839_f0  denovo4891_f0  denovo4917_f0  denovo4943_f0  denovo4969_f0  denovo4995_f0  denovo5203_f0  denovo5229_f0  denovo5255_f0  denovo5385_f0  denovo5437_f0  denovo5515_f0  denovo5593_f0  denovo5619_f0  denovo5645_f0  denovo5931_f0  denovo6009_f0  denovo6061_f0  denovo6113_f0  denovo6217_f0  denovo6243_f0  denovo6269_f0  denovo6581_f0  denovo6607_f0  denovo6685_f0  denovo6711_f0  denovo6763_f0  denovo6815_f0  denovo6867_f0  denovo6997_f0  denovo7075_f0  denovo7127_f0  denovo7153_f0  denovo7257_f0  denovo7335_f0  denovo7387_f0  denovo7413_f0  denovo7517_f0  denovo7543_f0  denovo7569_f0  denovo7621_f0  denovo7673_f0  denovo7699_f0  denovo7751_f0  denovo7803_f0  denovo7985_f0  denovo8037_f0  denovo8063_f0  denovo8219_f0  denovo8245_f0  denovo8271_f0  denovo8297_f0  denovo8401_f0  denovo8427_f0  denovo8453_f0  denovo8583_f0  denovo8635_f0  denovo8661_f0  denovo8687_f0  denovo8765_f0  denovo8817_f0  denovo8843_f0  denovo8895_f0  denovo8921_f0  denovo8947_f0  denovo8973_f0  denovo8999_f0  denovo9129_f0  denovo9155_f0  denovo9181_f0  denovo9311_f0  denovo9337_f0  denovo9363_f0  denovo9389_f0  denovo9441_f0  denovo9493_f0  denovo9545_f0  denovo9597_f0  denovo9675_f0  denovo9701_f0  denovo9727_f0  denovo9805_f0  denovo9831_f0  denovo9857_f0  denovo9883_f0  denovo9935_f0  denovo9961_f0  denovo9987_f0  denovo10065_f0 denovo10169_f0 denovo10195_f0 denovo10221_f0 denovo10273_f0 denovo10299_f0 denovo10351_f0 denovo10377_f0 denovo10403_f0 denovo10429_f0 denovo10481_f0 denovo10507_f0 denovo10533_f0 denovo10611_f0 denovo10663_f0 denovo10689_f0 denovo10767_f0 denovo10845_f0 denovo10871_f0 denovo10923_f0 denovo10975_f0 denovo11027_f0 denovo11079_f0 denovo11105_f0 denovo11157_f0 denovo11183_f0 denovo11235_f0 denovo11261_f0 denovo11313_f0 denovo11339_f0 denovo11443_f0 denovo11547_f0 denovo11911_f0 denovo11963_f0 denovo12067_f0 
GNV139000                     denovo42_f0    denovo94_f0    denovo120_f0   denovo146_f0   denovo224_f0   denovo250_f0   denovo276_f0   denovo302_f0   denovo354_f0   denovo432_f0   denovo536_f0   denovo588_f0   denovo614_f0   denovo744_f0   denovo796_f0   denovo952_f0   denovo978_f0   denovo1056_f0  denovo1082_f0  denovo1160_f0  denovo1316_f0  denovo1498_f0  denovo1524_f0  denovo1654_f0  denovo1758_f0  denovo1784_f0  denovo1810_f0  denovo1966_f0  denovo2148_f0  denovo2200_f0  denovo2252_f0  denovo2278_f0  denovo2356_f0  denovo2460_f0  denovo2486_f0  denovo2512_f0  denovo2538_f0  denovo2590_f0  denovo2616_f0  denovo2668_f0  denovo2772_f0  denovo2798_f0  denovo2850_f0  denovo2876_f0  denovo2928_f0  denovo3006_f0  denovo3058_f0  denovo3162_f0  denovo3240_f0  denovo3266_f0  denovo3292_f0  denovo3318_f0  denovo3344_f0  denovo3448_f0  denovo3500_f0  denovo3604_f0  denovo3630_f0  denovo3682_f0  denovo3708_f0  denovo3734_f0  denovo3786_f0  denovo3864_f0  denovo3968_f0  denovo3994_f0  denovo4046_f0  denovo4072_f0  denovo4202_f0  denovo4306_f0  denovo4332_f0  denovo4358_f0  denovo4410_f0  denovo4462_f0  denovo4566_f0  denovo4644_f0  denovo4748_f0  denovo4774_f0  denovo4800_f0  denovo4852_f0  denovo4904_f0  denovo4930_f0  denovo4956_f0  denovo4982_f0  denovo5008_f0  denovo5216_f0  denovo5242_f0  denovo5268_f0  denovo5398_f0  denovo5450_f0  denovo5528_f0  denovo5606_f0  denovo5632_f0  denovo5658_f0  denovo5944_f0  denovo6022_f0  denovo6074_f0  denovo6126_f0  denovo6230_f0  denovo6256_f0  denovo6282_f0  denovo6594_f0  denovo6620_f0  denovo6698_f0  denovo6724_f0  denovo6776_f0  denovo6828_f0  denovo6880_f0  denovo7010_f0  denovo7088_f0  denovo7140_f0  denovo7166_f0  denovo7270_f0  denovo7348_f0  denovo7400_f0  denovo7426_f0  denovo7530_f0  denovo7556_f0  denovo7582_f0  denovo7634_f0  denovo7686_f0  denovo7712_f0  denovo7764_f0  denovo7816_f0  denovo7998_f0  denovo8050_f0  denovo8076_f0  denovo8232_f0  denovo8258_f0  denovo8284_f0  denovo8310_f0  denovo8414_f0  denovo8440_f0  denovo8466_f0  denovo8596_f0  denovo8648_f0  denovo8674_f0  denovo8700_f0  denovo8778_f0  denovo8830_f0  denovo8856_f0  denovo8908_f0  denovo8934_f0  denovo8960_f0  denovo8986_f0  denovo9012_f0  denovo9142_f0  denovo9168_f0  denovo9194_f0  denovo9324_f0  denovo9350_f0  denovo9376_f0  denovo9402_f0  denovo9454_f0  denovo9506_f0  denovo9558_f0  denovo9610_f0  denovo9688_f0  denovo9714_f0  denovo9740_f0  denovo9818_f0  denovo9844_f0  denovo9870_f0  denovo9896_f0  denovo9948_f0  denovo9974_f0  denovo10000_f0 denovo10078_f0 denovo10182_f0 denovo10208_f0 denovo10234_f0 denovo10286_f0 denovo10312_f0 denovo10364_f0 denovo10390_f0 denovo10416_f0 denovo10442_f0 denovo10494_f0 denovo10520_f0 denovo10546_f0 denovo10624_f0 denovo10676_f0 denovo10702_f0 denovo10780_f0 denovo10858_f0 denovo10884_f0 denovo10936_f0 denovo10988_f0 denovo11040_f0 denovo11092_f0 denovo11118_f0 denovo11170_f0 denovo11196_f0 denovo11248_f0 denovo11274_f0 denovo11326_f0 denovo11352_f0 denovo11456_f0 denovo11560_f0 denovo11924_f0 denovo11976_f0 denovo12080_f0 
Msexta                        denovo43_f0    denovo95_f0    denovo121_f0   denovo147_f0   denovo225_f0   denovo251_f0   denovo277_f0   denovo303_f0   denovo355_f0   denovo433_f0   denovo537_f0   denovo589_f0   denovo615_f0   denovo745_f0   denovo797_f0   denovo953_f0   denovo979_f0   denovo1057_f0  denovo1083_f0  denovo1161_f0  denovo1317_f0  denovo1499_f0  denovo1525_f0  denovo1655_f0  denovo1759_f0  denovo1785_f0  denovo1811_f0  denovo1967_f0  denovo2149_f0  denovo2201_f0  denovo2253_f0  denovo2279_f0  denovo2357_f0  denovo2461_f0  denovo2487_f0  denovo2513_f0  denovo2539_f0  denovo2591_f0  denovo2617_f0  denovo2669_f0  denovo2773_f0  denovo2799_f0  denovo2851_f0  denovo2877_f0  denovo2929_f0  denovo3007_f0  denovo3059_f0  denovo3163_f0  denovo3241_f0  denovo3267_f0  denovo3293_f0  denovo3319_f0  denovo3345_f0  denovo3449_f0  denovo3501_f0  denovo3605_f0  denovo3631_f0  denovo3683_f0  denovo3709_f0  denovo3735_f0  denovo3787_f0  denovo3865_f0  denovo3969_f0  denovo3995_f0  denovo4047_f0  denovo4073_f0  denovo4203_f0  denovo4307_f0  denovo4333_f0  denovo4359_f0  denovo4411_f0  denovo4463_f0  denovo4567_f0  denovo4645_f0  denovo4749_f0  denovo4775_f0  denovo4801_f0  denovo4853_f0  denovo4905_f0  denovo4931_f0  denovo4957_f0  denovo4983_f0  denovo5009_f0  denovo5217_f0  denovo5243_f0  denovo5269_f0  denovo5399_f0  denovo5451_f0  denovo5529_f0  denovo5607_f0  denovo5633_f0  denovo5659_f0  denovo5945_f0  denovo6023_f0  denovo6075_f0  denovo6127_f0  denovo6231_f0  denovo6257_f0  denovo6283_f0  denovo6595_f0  denovo6621_f0  denovo6699_f0  denovo6725_f0  denovo6777_f0  denovo6829_f0  denovo6881_f0  denovo7011_f0  denovo7089_f0  denovo7141_f0  denovo7167_f0  denovo7271_f0  denovo7349_f0  denovo7401_f0  denovo7427_f0  denovo7531_f0  denovo7557_f0  denovo7583_f0  denovo7635_f0  denovo7687_f0  denovo7713_f0  denovo7765_f0  denovo7817_f0  denovo7999_f0  denovo8051_f0  denovo8077_f0  denovo8233_f0  denovo8259_f0  denovo8285_f0  denovo8311_f0  denovo8415_f0  denovo8441_f0  denovo8467_f0  denovo8597_f0  denovo8649_f0  denovo8675_f0  denovo8701_f0  denovo8779_f0  denovo8831_f0  denovo8857_f0  denovo8909_f0  denovo8935_f0  denovo8961_f0  denovo8987_f0  denovo9013_f0  denovo9143_f0  denovo9169_f0  denovo9195_f0  denovo9325_f0  denovo9351_f0  denovo9377_f0  denovo9403_f0  denovo9455_f0  denovo9507_f0  denovo9559_f0  denovo9611_f0  denovo9689_f0  denovo9715_f0  denovo9741_f0  denovo9819_f0  denovo9845_f0  denovo9871_f0  denovo9897_f0  denovo9949_f0  denovo9975_f0  denovo10001_f0 denovo10079_f0 denovo10183_f0 denovo10209_f0 denovo10235_f0 denovo10287_f0 denovo10313_f0 denovo10365_f0 denovo10391_f0 denovo10417_f0 denovo10443_f0 denovo10495_f0 denovo10521_f0 denovo10547_f0 denovo10625_f0 denovo10677_f0 denovo10703_f0 denovo10781_f0 denovo10859_f0 denovo10885_f0 denovo10937_f0 denovo10989_f0 denovo11041_f0 denovo11093_f0 denovo11119_f0 denovo11171_f0 denovo11197_f0 denovo11249_f0 denovo11275_f0 denovo11327_f0 denovo11353_f0 denovo11457_f0 denovo11561_f0 denovo11925_f0 denovo11977_f0 denovo12081_f0 
acti2                         denovo51_f0    denovo103_f0   denovo129_f0   denovo155_f0   denovo233_f0   denovo259_f0   denovo285_f0   denovo311_f0   denovo363_f0   denovo441_f0   denovo545_f0   denovo597_f0   denovo623_f0   denovo753_f0   denovo805_f0   denovo961_f0   denovo987_f0   denovo1065_f0  denovo1091_f0  denovo1169_f0  denovo1325_f0  denovo1507_f0  denovo1533_f0  denovo1663_f0  denovo1767_f0  denovo1793_f0  denovo1819_f0  denovo1975_f0  denovo2157_f0  denovo2209_f0  denovo2261_f0  denovo2287_f0  denovo2365_f0  denovo2469_f0  denovo2495_f0  denovo2521_f0  denovo2547_f0  denovo2599_f0  denovo2625_f0  denovo2677_f0  denovo2781_f0  denovo2807_f0  denovo2859_f0  denovo2885_f0  denovo2937_f0  denovo3015_f0  denovo3067_f0  denovo3171_f0  denovo3249_f0  denovo3275_f0  denovo3301_f0  denovo3327_f0  denovo3353_f0  denovo3457_f0  denovo3509_f0  denovo3613_f0  denovo3639_f0  denovo3691_f0  denovo3717_f0  denovo3743_f0  denovo3795_f0  denovo3873_f0  denovo3977_f0  denovo4003_f0  denovo4055_f0  denovo4081_f0  denovo4211_f0  denovo4315_f0  denovo4341_f0  denovo4367_f0  denovo4419_f0  denovo4471_f0  denovo4575_f0  denovo4653_f0  denovo4757_f0  denovo4783_f0  denovo4809_f0  denovo4861_f0  denovo4913_f0  denovo4939_f0  denovo4965_f0  denovo4991_f0  denovo5017_f0  denovo5225_f0  denovo5251_f0  denovo5277_f0  denovo5407_f0  denovo5459_f0  denovo5537_f0  denovo5615_f0  denovo5641_f0  denovo5667_f0  denovo5953_f0  denovo6031_f0  denovo6083_f0  denovo6135_f0  denovo6239_f0  denovo6265_f0  denovo6291_f0  denovo6603_f0  denovo6629_f0  denovo6707_f0  denovo6733_f0  denovo6785_f0  denovo6837_f0  denovo6889_f0  denovo7019_f0  denovo7097_f0  denovo7149_f0  denovo7175_f0  denovo7279_f0  denovo7357_f0  denovo7409_f0  denovo7435_f0  denovo7539_f0  denovo7565_f0  denovo7591_f0  denovo7643_f0  denovo7695_f0  denovo7721_f0  denovo7773_f0  denovo7825_f0  denovo8007_f0  denovo8059_f0  denovo8085_f0  denovo8241_f0  denovo8267_f0  denovo8293_f0  denovo8319_f0  denovo8423_f0  denovo8449_f0  denovo8475_f0  denovo8605_f0  denovo8657_f0  denovo8683_f0  denovo8709_f0  denovo8787_f0  denovo8839_f0  denovo8865_f0  denovo8917_f0  denovo8943_f0  denovo8969_f0  denovo8995_f0  denovo9021_f0  denovo9151_f0  denovo9177_f0  denovo9203_f0  denovo9333_f0  denovo9359_f0  denovo9385_f0  denovo9411_f0  denovo9463_f0  denovo9515_f0  denovo9567_f0  denovo9619_f0  denovo9697_f0  denovo9723_f0  denovo9749_f0  denovo9827_f0  denovo9853_f0  denovo9879_f0  denovo9905_f0  denovo9957_f0  denovo9983_f0  denovo10009_f0 denovo10087_f0 denovo10191_f0 denovo10217_f0 denovo10243_f0 denovo10295_f0 denovo10321_f0 denovo10373_f0 denovo10399_f0 denovo10425_f0 denovo10451_f0 denovo10503_f0 denovo10529_f0 denovo10555_f0 denovo10633_f0 denovo10685_f0 denovo10711_f0 denovo10789_f0 denovo10867_f0 denovo10893_f0 denovo10945_f0 denovo10997_f0 denovo11049_f0 denovo11101_f0 denovo11127_f0 denovo11179_f0 denovo11205_f0 denovo11257_f0 denovo11283_f0 denovo11335_f0 denovo11361_f0 denovo11465_f0 denovo11569_f0 denovo11933_f0 denovo11985_f0 denovo12089_f0 
FG120071B                     denovo35_f0    denovo87_f0    denovo113_f0   denovo139_f0   denovo217_f0   denovo243_f0   denovo269_f0   denovo295_f0   denovo347_f0   denovo425_f0   denovo529_f0   denovo581_f0   denovo607_f0   denovo737_f0   denovo789_f0   denovo945_f0   denovo971_f0   denovo1049_f0  denovo1075_f0  denovo1153_f0  denovo1309_f0  denovo1491_f0  denovo1517_f0  denovo1647_f0  denovo1751_f0  denovo1777_f0  denovo1803_f0  denovo1959_f0  denovo2141_f0  denovo2193_f0  denovo2245_f0  denovo2271_f0  denovo2349_f0  denovo2453_f0  denovo2479_f0  denovo2505_f0  denovo2531_f0  denovo2583_f0  denovo2609_f0  denovo2661_f0  denovo2765_f0  denovo2791_f0  denovo2843_f0  denovo2869_f0  denovo2921_f0  denovo2999_f0  denovo3051_f0  denovo3155_f0  denovo3233_f0  denovo3259_f0  denovo3285_f0  denovo3311_f0  denovo3337_f0  denovo3441_f0  denovo3493_f0  denovo3597_f0  denovo3623_f0  denovo3675_f0  denovo3701_f0  denovo3727_f0  denovo3779_f0  denovo3857_f0  denovo3961_f0  denovo3987_f0  denovo4039_f0  denovo4065_f0  denovo4195_f0  denovo4299_f0  denovo4325_f0  denovo4351_f0  denovo4403_f0  denovo4455_f0  denovo4559_f0  denovo4637_f0  denovo4741_f0  denovo4767_f0  denovo4793_f0  denovo4845_f0  denovo4897_f0  denovo4923_f0  denovo4949_f0  denovo4975_f0  denovo5001_f0  denovo5209_f0  denovo5235_f0  denovo5261_f0  denovo5391_f0  denovo5443_f0  denovo5521_f0  denovo5599_f0  denovo5625_f0  denovo5651_f0  denovo5937_f0  denovo6015_f0  denovo6067_f0  denovo6119_f0  denovo6223_f0  denovo6249_f0  denovo6275_f0  denovo6587_f0  denovo6613_f0  denovo6691_f0  denovo6717_f0  denovo6769_f0  denovo6821_f0  denovo6873_f0  denovo7003_f0  denovo7081_f0  denovo7133_f0  denovo7159_f0  denovo7263_f0  denovo7341_f0  denovo7393_f0  denovo7419_f0  denovo7523_f0  denovo7549_f0  denovo7575_f0  denovo7627_f0  denovo7679_f0  denovo7705_f0  denovo7757_f0  denovo7809_f0  denovo7991_f0  denovo8043_f0  denovo8069_f0  denovo8225_f0  denovo8251_f0  denovo8277_f0  denovo8303_f0  denovo8407_f0  denovo8433_f0  denovo8459_f0  denovo8589_f0  denovo8641_f0  denovo8667_f0  denovo8693_f0  denovo8771_f0  denovo8823_f0  denovo8849_f0  denovo8901_f0  denovo8927_f0  denovo8953_f0  denovo8979_f0  denovo9005_f0  denovo9135_f0  denovo9161_f0  denovo9187_f0  denovo9317_f0  denovo9343_f0  denovo9369_f0  denovo9395_f0  denovo9447_f0  denovo9499_f0  denovo9551_f0  denovo9603_f0  denovo9681_f0  denovo9707_f0  denovo9733_f0  denovo9811_f0  denovo9837_f0  denovo9863_f0  denovo9889_f0  denovo9941_f0  denovo9967_f0  denovo9993_f0  denovo10071_f0 denovo10175_f0 denovo10201_f0 denovo10227_f0 denovo10279_f0 denovo10305_f0 denovo10357_f0 denovo10383_f0 denovo10409_f0 denovo10435_f0 denovo10487_f0 denovo10513_f0 denovo10539_f0 denovo10617_f0 denovo10669_f0 denovo10695_f0 denovo10773_f0 denovo10851_f0 denovo10877_f0 denovo10929_f0 denovo10981_f0 denovo11033_f0 denovo11085_f0 denovo11111_f0 denovo11163_f0 denovo11189_f0 denovo11241_f0 denovo11267_f0 denovo11319_f0 denovo11345_f0 denovo11449_f0 denovo11553_f0 denovo11917_f0 denovo11969_f0 denovo12073_f0 
GNV120027                     denovo39_f0    denovo91_f0    denovo117_f0   denovo143_f0   denovo221_f0   denovo247_f0   denovo273_f0   denovo299_f0   denovo351_f0   denovo429_f0   denovo533_f0   denovo585_f0   denovo611_f0   denovo741_f0   denovo793_f0   denovo949_f0   denovo975_f0   denovo1053_f0  denovo1079_f0  denovo1157_f0  denovo1313_f0  denovo1495_f0  denovo1521_f0  denovo1651_f0  denovo1755_f0  denovo1781_f0  denovo1807_f0  denovo1963_f0  denovo2145_f0  denovo2197_f0  denovo2249_f0  denovo2275_f0  denovo2353_f0  denovo2457_f0  denovo2483_f0  denovo2509_f0  denovo2535_f0  denovo2587_f0  denovo2613_f0  denovo2665_f0  denovo2769_f0  denovo2795_f0  denovo2847_f0  denovo2873_f0  denovo2925_f0  denovo3003_f0  denovo3055_f0  denovo3159_f0  denovo3237_f0  denovo3263_f0  denovo3289_f0  denovo3315_f0  denovo3341_f0  denovo3445_f0  denovo3497_f0  denovo3601_f0  denovo3627_f0  denovo3679_f0  denovo3705_f0  denovo3731_f0  denovo3783_f0  denovo3861_f0  denovo3965_f0  denovo3991_f0  denovo4043_f0  denovo4069_f0  denovo4199_f0  denovo4303_f0  denovo4329_f0  denovo4355_f0  denovo4407_f0  denovo4459_f0  denovo4563_f0  denovo4641_f0  denovo4745_f0  denovo4771_f0  denovo4797_f0  denovo4849_f0  denovo4901_f0  denovo4927_f0  denovo4953_f0  denovo4979_f0  denovo5005_f0  denovo5213_f0  denovo5239_f0  denovo5265_f0  denovo5395_f0  denovo5447_f0  denovo5525_f0  denovo5603_f0  denovo5629_f0  denovo5655_f0  denovo5941_f0  denovo6019_f0  denovo6071_f0  denovo6123_f0  denovo6227_f0  denovo6253_f0  denovo6279_f0  denovo6591_f0  denovo6617_f0  denovo6695_f0  denovo6721_f0  denovo6773_f0  denovo6825_f0  denovo6877_f0  denovo7007_f0  denovo7085_f0  denovo7137_f0  denovo7163_f0  denovo7267_f0  denovo7345_f0  denovo7397_f0  denovo7423_f0  denovo7527_f0  denovo7553_f0  denovo7579_f0  denovo7631_f0  denovo7683_f0  denovo7709_f0  denovo7761_f0  denovo7813_f0  denovo7995_f0  denovo8047_f0  denovo8073_f0  denovo8229_f0  denovo8255_f0  denovo8281_f0  denovo8307_f0  denovo8411_f0  denovo8437_f0  denovo8463_f0  denovo8593_f0  denovo8645_f0  denovo8671_f0  denovo8697_f0  denovo8775_f0  denovo8827_f0  denovo8853_f0  denovo8905_f0  denovo8931_f0  denovo8957_f0  denovo8983_f0  denovo9009_f0  denovo9139_f0  denovo9165_f0  denovo9191_f0  denovo9321_f0  denovo9347_f0  denovo9373_f0  denovo9399_f0  denovo9451_f0  denovo9503_f0  denovo9555_f0  denovo9607_f0  denovo9685_f0  denovo9711_f0  denovo9737_f0  denovo9815_f0  denovo9841_f0  denovo9867_f0  denovo9893_f0  denovo9945_f0  denovo9971_f0  denovo9997_f0  denovo10075_f0 denovo10179_f0 denovo10205_f0 denovo10231_f0 denovo10283_f0 denovo10309_f0 denovo10361_f0 denovo10387_f0 denovo10413_f0 denovo10439_f0 denovo10491_f0 denovo10517_f0 denovo10543_f0 denovo10621_f0 denovo10673_f0 denovo10699_f0 denovo10777_f0 denovo10855_f0 denovo10881_f0 denovo10933_f0 denovo10985_f0 denovo11037_f0 denovo11089_f0 denovo11115_f0 denovo11167_f0 denovo11193_f0 denovo11245_f0 denovo11271_f0 denovo11323_f0 denovo11349_f0 denovo11453_f0 denovo11557_f0 denovo11921_f0 denovo11973_f0 denovo12077_f0 
Bmoricds                      denovo26_f0    denovo78_f0    denovo104_f0   denovo130_f0   denovo208_f0   denovo234_f0   denovo260_f0   denovo286_f0   denovo338_f0   denovo416_f0   denovo520_f0   denovo572_f0   denovo598_f0   denovo728_f0   denovo780_f0   denovo936_f0   denovo962_f0   denovo1040_f0  denovo1066_f0  denovo1144_f0  denovo1300_f0  denovo1482_f0  denovo1508_f0  denovo1638_f0  denovo1742_f0  denovo1768_f0  denovo1794_f0  denovo1950_f0  denovo2132_f0  denovo2184_f0  denovo2236_f0  denovo2262_f0  denovo2340_f0  denovo2444_f0  denovo2470_f0  denovo2496_f0  denovo2522_f0  denovo2574_f0  denovo2600_f0  denovo2652_f0  denovo2756_f0  denovo2782_f0  denovo2834_f0  denovo2860_f0  denovo2912_f0  denovo2990_f0  denovo3042_f0  denovo3146_f0  denovo3224_f0  denovo3250_f0  denovo3276_f0  denovo3302_f0  denovo3328_f0  denovo3432_f0  denovo3484_f0  denovo3588_f0  denovo3614_f0  denovo3666_f0  denovo3692_f0  denovo3718_f0  denovo3770_f0  denovo3848_f0  denovo3952_f0  denovo3978_f0  denovo4030_f0  denovo4056_f0  denovo4186_f0  denovo4290_f0  denovo4316_f0  denovo4342_f0  denovo4394_f0  denovo4446_f0  denovo4550_f0  denovo4628_f0  denovo4732_f0  denovo4758_f0  denovo4784_f0  denovo4836_f0  denovo4888_f0  denovo4914_f0  denovo4940_f0  denovo4966_f0  denovo4992_f0  denovo5200_f0  denovo5226_f0  denovo5252_f0  denovo5382_f0  denovo5434_f0  denovo5512_f0  denovo5590_f0  denovo5616_f0  denovo5642_f0  denovo5928_f0  denovo6006_f0  denovo6058_f0  denovo6110_f0  denovo6214_f0  denovo6240_f0  denovo6266_f0  denovo6578_f0  denovo6604_f0  denovo6682_f0  denovo6708_f0  denovo6760_f0  denovo6812_f0  denovo6864_f0  denovo6994_f0  denovo7072_f0  denovo7124_f0  denovo7150_f0  denovo7254_f0  denovo7332_f0  denovo7384_f0  denovo7410_f0  denovo7514_f0  denovo7540_f0  denovo7566_f0  denovo7618_f0  denovo7670_f0  denovo7696_f0  denovo7748_f0  denovo7800_f0  denovo7982_f0  denovo8034_f0  denovo8060_f0  denovo8216_f0  denovo8242_f0  denovo8268_f0  denovo8294_f0  denovo8398_f0  denovo8424_f0  denovo8450_f0  denovo8580_f0  denovo8632_f0  denovo8658_f0  denovo8684_f0  denovo8762_f0  denovo8814_f0  denovo8840_f0  denovo8892_f0  denovo8918_f0  denovo8944_f0  denovo8970_f0  denovo8996_f0  denovo9126_f0  denovo9152_f0  denovo9178_f0  denovo9308_f0  denovo9334_f0  denovo9360_f0  denovo9386_f0  denovo9438_f0  denovo9490_f0  denovo9542_f0  denovo9594_f0  denovo9672_f0  denovo9698_f0  denovo9724_f0  denovo9802_f0  denovo9828_f0  denovo9854_f0  denovo9880_f0  denovo9932_f0  denovo9958_f0  denovo9984_f0  denovo10062_f0 denovo10166_f0 denovo10192_f0 denovo10218_f0 denovo10270_f0 denovo10296_f0 denovo10348_f0 denovo10374_f0 denovo10400_f0 denovo10426_f0 denovo10478_f0 denovo10504_f0 denovo10530_f0 denovo10608_f0 denovo10660_f0 denovo10686_f0 denovo10764_f0 denovo10842_f0 denovo10868_f0 denovo10920_f0 denovo10972_f0 denovo11024_f0 denovo11076_f0 denovo11102_f0 denovo11154_f0 denovo11180_f0 denovo11232_f0 denovo11258_f0 denovo11310_f0 denovo11336_f0 denovo11440_f0 denovo11544_f0 denovo11908_f0 denovo11960_f0 denovo12064_f0 
Pcit2                         denovo45_f0    denovo97_f0    denovo123_f0   denovo149_f0   denovo227_f0   denovo253_f0   denovo279_f0   denovo305_f0   denovo357_f0   denovo435_f0   denovo539_f0   denovo591_f0   denovo617_f0   denovo747_f0   denovo799_f0   denovo955_f0   denovo981_f0   denovo1059_f0  denovo1085_f0  denovo1163_f0  denovo1319_f0  denovo1501_f0  denovo1527_f0  denovo1657_f0  denovo1761_f0  denovo1787_f0  denovo1813_f0  denovo1969_f0  denovo2151_f0  denovo2203_f0  denovo2255_f0  denovo2281_f0  denovo2359_f0  denovo2463_f0  denovo2489_f0  denovo2515_f0  denovo2541_f0  denovo2593_f0  denovo2619_f0  denovo2671_f0  denovo2775_f0  denovo2801_f0  denovo2853_f0  denovo2879_f0  denovo2931_f0  denovo3009_f0  denovo3061_f0  denovo3165_f0  denovo3243_f0  denovo3269_f0  denovo3295_f0  denovo3321_f0  denovo3347_f0  denovo3451_f0  denovo3503_f0  denovo3607_f0  denovo3633_f0  denovo3685_f0  denovo3711_f0  denovo3737_f0  denovo3789_f0  denovo3867_f0  denovo3971_f0  denovo3997_f0  denovo4049_f0  denovo4075_f0  denovo4205_f0  denovo4309_f0  denovo4335_f0  denovo4361_f0  denovo4413_f0  denovo4465_f0  denovo4569_f0  denovo4647_f0  denovo4751_f0  denovo4777_f0  denovo4803_f0  denovo4855_f0  denovo4907_f0  denovo4933_f0  denovo4959_f0  denovo4985_f0  denovo5011_f0  denovo5219_f0  denovo5245_f0  denovo5271_f0  denovo5401_f0  denovo5453_f0  denovo5531_f0  denovo5609_f0  denovo5635_f0  denovo5661_f0  denovo5947_f0  denovo6025_f0  denovo6077_f0  denovo6129_f0  denovo6233_f0  denovo6259_f0  denovo6285_f0  denovo6597_f0  denovo6623_f0  denovo6701_f0  denovo6727_f0  denovo6779_f0  denovo6831_f0  denovo6883_f0  denovo7013_f0  denovo7091_f0  denovo7143_f0  denovo7169_f0  denovo7273_f0  denovo7351_f0  denovo7403_f0  denovo7429_f0  denovo7533_f0  denovo7559_f0  denovo7585_f0  denovo7637_f0  denovo7689_f0  denovo7715_f0  denovo7767_f0  denovo7819_f0  denovo8001_f0  denovo8053_f0  denovo8079_f0  denovo8235_f0  denovo8261_f0  denovo8287_f0  denovo8313_f0  denovo8417_f0  denovo8443_f0  denovo8469_f0  denovo8599_f0  denovo8651_f0  denovo8677_f0  denovo8703_f0  denovo8781_f0  denovo8833_f0  denovo8859_f0  denovo8911_f0  denovo8937_f0  denovo8963_f0  denovo8989_f0  denovo9015_f0  denovo9145_f0  denovo9171_f0  denovo9197_f0  denovo9327_f0  denovo9353_f0  denovo9379_f0  denovo9405_f0  denovo9457_f0  denovo9509_f0  denovo9561_f0  denovo9613_f0  denovo9691_f0  denovo9717_f0  denovo9743_f0  denovo9821_f0  denovo9847_f0  denovo9873_f0  denovo9899_f0  denovo9951_f0  denovo9977_f0  denovo10003_f0 denovo10081_f0 denovo10185_f0 denovo10211_f0 denovo10237_f0 denovo10289_f0 denovo10315_f0 denovo10367_f0 denovo10393_f0 denovo10419_f0 denovo10445_f0 denovo10497_f0 denovo10523_f0 denovo10549_f0 denovo10627_f0 denovo10679_f0 denovo10705_f0 denovo10783_f0 denovo10861_f0 denovo10887_f0 denovo10939_f0 denovo10991_f0 denovo11043_f0 denovo11095_f0 denovo11121_f0 denovo11173_f0 denovo11199_f0 denovo11251_f0 denovo11277_f0 denovo11329_f0 denovo11355_f0 denovo11459_f0 denovo11563_f0 denovo11927_f0 denovo11979_f0 denovo12083_f0 

Concatenation entropy_0.48_0.00_loci_50_to_249 will have the following data
OTU                           EOG69CQC1_1         EOG6SN1SH_1         EOG6PRSVJ_1         EOG680J15_1         EOG60ZR1Z_1         EOG60P4BD_1         EOG64QT4T_1         EOG6NCMGZ_1         EOG6K3M19_1         EOG60P4BJ_1         EOG6DBTK0_1         EOG6GTKHJ_1         EOG68KRFS_1         EOG6GHZSR_1         EOG666VQ4_1         EOG698V44_1         EOG6894QQ_1         EOG6868GW_1         EOG69PB31_1         EOG6JDHBD_1         EOG63212K_1         EOG68D102_1         EOG60ZR1T_1         EOG6CJVMH_1         EOG698V46_1         EOG68KRG8_1         EOG6N8R81_1         EOG63BMSV_1         EOG69KFV1_1         EOG6CC44S_1         EOG6FXRCK_1         EOG6R2400_1         EOG68KRG4_1         EOG61C70T_1         EOG6BK575_1         EOG63212W_1         EOG6F7NPJ_1         EOG695ZW4_1         EOG61NTQM_1         EOG6NZTZ3_1         EOG6J3WM7_1         EOG680J16_1         EOG68SGXM_1         EOG6RNBCT_1         EOG6RFKXS_1         EOG67SSJJ_1         EOG69GKKW_1         EOG6QFWHZ_1         EOG6RFKXB_1         EOG647FXX_1         EOG6B8JHP_1         EOG6S1TBX_1         EOG6DFPRT_1         EOG65HS25_1         EOG6FFD5C_1         EOG6R506D_1         EOG6F4SFZ_1         EOG6Q8524_1         EOG6QVCGP_1         EOG669QZR_1         EOG6JT09B_1         EOG65TCRS_1         EOG6QRH88_1         EOG6M65N6_1         EOG6QRH81_1         EOG6RJG4W_1         EOG6KKZ6X_1         EOG60VVTG_1         EOG6QC198_1         EOG6D26TG_1         EOG679FBS_1         EOG61RPZF_1         EOG6PC9WG_1         EOG60GCVD_1         EOG6R506Q_1         EOG605S4X_1         EOG65TCRN_1         EOG608ND4_1         EOG6640GX_1         EOG6SQX12_1         EOG69GKMS_1         EOG6D26TV_1         EOG605S4Z_1         EOG6N04JN_1         EOG64TPCQ_1         EOG6NP76T_1         EOG67H5V2_1         EOG66DM62_1         EOG6QFWJH_1         EOG6N04K4_1         EOG69KFTV_1         EOG6CJVMQ_1         EOG62V8KS_1         EOG6DZ204_1         EOG6C5CPH_1         EOG6FFD51_1         EOG6QNN0J_1         EOG6KSPP5_1         EOG6N5W22_1         EOG6PK2CG_1         EOG6NP779_1         EOG6933N9_1         EOG66Q6XP_1         EOG6JQ427_1         EOG6C5CNP_1         EOG6R5066_1         EOG6FTW4C_1         EOG65B1K2_1         EOG66WZD1_1         EOG6FR0WR_1         EOG6P2Q64_1         EOG666VQC_1         EOG63R3R5_1         EOG6PC9WF_1         EOG6Q58T0_1         EOG641QGH_1         EOG6933N7_1         EOG69S6BG_1         EOG6NS3FV_1         EOG65MN9B_1         EOG641QFW_1         EOG6N04J6_1         EOG6GMV1P_1         EOG6S4PKX_1         EOG6BZN62_1         EOG6SXNGX_1         EOG6CG0CT_1         EOG6HHP6B_1         EOG605S4Q_1         EOG6R7VF9_1         EOG6CZBM5_1         EOG6JT092_1         EOG64F6DK_1         EOG6FJ8D6_1         EOG6QZ7QX_1         EOG6F1X6M_1         EOG67PX9B_1         EOG6SBF2V_1         EOG6KSPQ0_1         EOG6GMV1H_1         EOG63R3RC_1         EOG68SGX8_1         EOG60K83N_1         EOG6KD6Q9_1         EOG68GW6Q_1         EOG6Q852G_1         EOG676K44_1         EOG6FN4NZ_1         EOG6S1TC2_1         EOG6HT8WD_1         EOG63FH1F_1         EOG6CRM3H_1         EOG6PRSVP_1         EOG6R5069_1         EOG6CC453_1         EOG6001PC_1         EOG676K40_1         EOG6N30SN_1         EOG6255XM_1         EOG64BB5X_1         EOG62FSN7_1         EOG6H72G0_1         EOG6PZJB1_1         EOG65QHHP_1         EOG615GHV_1         EOG62BXCZ_1         EOG6CG0D0_1         EOG69KFVF_1         EOG615GHP_1         EOG683D8Q_1         EOG64J2NS_1         EOG66HGF5_1         EOG6DFPSF_1         EOG66MBPR_1         EOG6DFPSD_1         EOG64BB5N_1         EOG6DBTJX_1         EOG6DBTHZ_1         EOG6KH2ZD_1         EOG6STS87_1         EOG67D9KV_1         EOG6SXNHG_1         EOG6GF3JF_1         EOG61RPZ9_1         EOG637RJP_1         EOG6J3WMR_1         EOG6GB79H_1         EOG6JDHBG_1         EOG6DV5QM_1         EOG6GTKHQ_1         EOG6FR0W5_1         EOG6K0QRT_1         EOG65DWSM_1         EOG6CC452_1         EOG6G4GTX_1         EOG61G37F_1         EOG6FFD5V_1         EOG6G4GTT_1         EOG6GXFR5_1         EOG6KPTG0_1         
Dplexcds                      denovo2_f0     denovo80_f0    denovo158_f0   denovo210_f0   denovo262_f0   denovo366_f0   denovo418_f0   denovo444_f0   denovo522_f0   denovo574_f0   denovo600_f0   denovo782_f0   denovo1042_f0  denovo1068_f0  denovo1146_f0  denovo1224_f0  denovo1354_f0  denovo1380_f0  denovo1406_f0  denovo1484_f0  denovo1510_f0  denovo1640_f0  denovo1666_f0  denovo1744_f0  denovo1796_f0  denovo1926_f0  denovo1952_f0  denovo1978_f0  denovo2004_f0  denovo2108_f0  denovo2134_f0  denovo2186_f0  denovo2238_f0  denovo2264_f0  denovo2290_f0  denovo2446_f0  denovo2472_f0  denovo2498_f0  denovo2524_f0  denovo2576_f0  denovo2602_f0  denovo2628_f0  denovo2654_f0  denovo2758_f0  denovo2836_f0  denovo2914_f0  denovo2992_f0  denovo3148_f0  denovo3252_f0  denovo3304_f0  denovo3330_f0  denovo3434_f0  denovo3486_f0  denovo3590_f0  denovo3616_f0  denovo3668_f0  denovo3720_f0  denovo3746_f0  denovo3772_f0  denovo3798_f0  denovo3824_f0  denovo3850_f0  denovo3954_f0  denovo3980_f0  denovo4058_f0  denovo4188_f0  denovo4318_f0  denovo4344_f0  denovo4370_f0  denovo4396_f0  denovo4552_f0  denovo4630_f0  denovo4734_f0  denovo4760_f0  denovo4786_f0  denovo4838_f0  denovo4890_f0  denovo4916_f0  denovo4942_f0  denovo4968_f0  denovo4994_f0  denovo5202_f0  denovo5228_f0  denovo5254_f0  denovo5436_f0  denovo5592_f0  denovo5644_f0  denovo5956_f0  denovo6008_f0  denovo6060_f0  denovo6086_f0  denovo6112_f0  denovo6190_f0  denovo6216_f0  denovo6242_f0  denovo6268_f0  denovo6294_f0  denovo6372_f0  denovo6502_f0  denovo6554_f0  denovo6580_f0  denovo6606_f0  denovo6658_f0  denovo6684_f0  denovo6710_f0  denovo6736_f0  denovo6762_f0  denovo6970_f0  denovo6996_f0  denovo7074_f0  denovo7126_f0  denovo7152_f0  denovo7256_f0  denovo7282_f0  denovo7308_f0  denovo7334_f0  denovo7360_f0  denovo7386_f0  denovo7490_f0  denovo7516_f0  denovo7542_f0  denovo7620_f0  denovo7672_f0  denovo7698_f0  denovo7828_f0  denovo7906_f0  denovo8036_f0  denovo8062_f0  denovo8218_f0  denovo8244_f0  denovo8270_f0  denovo8296_f0  denovo8374_f0  denovo8400_f0  denovo8426_f0  denovo8452_f0  denovo8556_f0  denovo8582_f0  denovo8634_f0  denovo8660_f0  denovo8686_f0  denovo8764_f0  denovo8842_f0  denovo8894_f0  denovo8920_f0  denovo8946_f0  denovo8972_f0  denovo8998_f0  denovo9128_f0  denovo9154_f0  denovo9180_f0  denovo9284_f0  denovo9310_f0  denovo9336_f0  denovo9362_f0  denovo9388_f0  denovo9440_f0  denovo9492_f0  denovo9700_f0  denovo9726_f0  denovo9778_f0  denovo9804_f0  denovo9830_f0  denovo9882_f0  denovo9960_f0  denovo10194_f0 denovo10220_f0 denovo10272_f0 denovo10298_f0 denovo10350_f0 denovo10402_f0 denovo10428_f0 denovo10454_f0 denovo10506_f0 denovo10532_f0 denovo10610_f0 denovo10662_f0 denovo10714_f0 denovo10740_f0 denovo10766_f0 denovo10818_f0 denovo10844_f0 denovo10922_f0 denovo11000_f0 denovo11026_f0 denovo11078_f0 denovo11156_f0 denovo11182_f0 denovo11286_f0 denovo11312_f0 denovo11338_f0 denovo11390_f0 denovo11416_f0 denovo11442_f0 denovo11468_f0 denovo11546_f0 denovo11702_f0 denovo11728_f0 denovo11910_f0 denovo12066_f0 
FG120077                      denovo10_f0    denovo88_f0    denovo166_f0   denovo218_f0   denovo270_f0   denovo374_f0   denovo426_f0   denovo452_f0   denovo530_f0   denovo582_f0   denovo608_f0   denovo790_f0   denovo1050_f0  denovo1076_f0  denovo1154_f0  denovo1232_f0  denovo1362_f0  denovo1388_f0  denovo1414_f0  denovo1492_f0  denovo1518_f0  denovo1648_f0  denovo1674_f0  denovo1752_f0  denovo1804_f0  denovo1934_f0  denovo1960_f0  denovo1986_f0  denovo2012_f0  denovo2116_f0  denovo2142_f0  denovo2194_f0  denovo2246_f0  denovo2272_f0  denovo2298_f0  denovo2454_f0  denovo2480_f0  denovo2506_f0  denovo2532_f0  denovo2584_f0  denovo2610_f0  denovo2636_f0  denovo2662_f0  denovo2766_f0  denovo2844_f0  denovo2922_f0  denovo3000_f0  denovo3156_f0  denovo3260_f0  denovo3312_f0  denovo3338_f0  denovo3442_f0  denovo3494_f0  denovo3598_f0  denovo3624_f0  denovo3676_f0  denovo3728_f0  denovo3754_f0  denovo3780_f0  denovo3806_f0  denovo3832_f0  denovo3858_f0  denovo3962_f0  denovo3988_f0  denovo4066_f0  denovo4196_f0  denovo4326_f0  denovo4352_f0  denovo4378_f0  denovo4404_f0  denovo4560_f0  denovo4638_f0  denovo4742_f0  denovo4768_f0  denovo4794_f0  denovo4846_f0  denovo4898_f0  denovo4924_f0  denovo4950_f0  denovo4976_f0  denovo5002_f0  denovo5210_f0  denovo5236_f0  denovo5262_f0  denovo5444_f0  denovo5600_f0  denovo5652_f0  denovo5964_f0  denovo6016_f0  denovo6068_f0  denovo6094_f0  denovo6120_f0  denovo6198_f0  denovo6224_f0  denovo6250_f0  denovo6276_f0  denovo6302_f0  denovo6380_f0  denovo6510_f0  denovo6562_f0  denovo6588_f0  denovo6614_f0  denovo6666_f0  denovo6692_f0  denovo6718_f0  denovo6744_f0  denovo6770_f0  denovo6978_f0  denovo7004_f0  denovo7082_f0  denovo7134_f0  denovo7160_f0  denovo7264_f0  denovo7290_f0  denovo7316_f0  denovo7342_f0  denovo7368_f0  denovo7394_f0  denovo7498_f0  denovo7524_f0  denovo7550_f0  denovo7628_f0  denovo7680_f0  denovo7706_f0  denovo7836_f0  denovo7914_f0  denovo8044_f0  denovo8070_f0  denovo8226_f0  denovo8252_f0  denovo8278_f0  denovo8304_f0  denovo8382_f0  denovo8408_f0  denovo8434_f0  denovo8460_f0  denovo8564_f0  denovo8590_f0  denovo8642_f0  denovo8668_f0  denovo8694_f0  denovo8772_f0  denovo8850_f0  denovo8902_f0  denovo8928_f0  denovo8954_f0  denovo8980_f0  denovo9006_f0  denovo9136_f0  denovo9162_f0  denovo9188_f0  denovo9292_f0  denovo9318_f0  denovo9344_f0  denovo9370_f0  denovo9396_f0  denovo9448_f0  denovo9500_f0  denovo9708_f0  denovo9734_f0  denovo9786_f0  denovo9812_f0  denovo9838_f0  denovo9890_f0  denovo9968_f0  denovo10202_f0 denovo10228_f0 denovo10280_f0 denovo10306_f0 denovo10358_f0 denovo10410_f0 denovo10436_f0 denovo10462_f0 denovo10514_f0 denovo10540_f0 denovo10618_f0 denovo10670_f0 denovo10722_f0 denovo10748_f0 denovo10774_f0 denovo10826_f0 denovo10852_f0 denovo10930_f0 denovo11008_f0 denovo11034_f0 denovo11086_f0 denovo11164_f0 denovo11190_f0 denovo11294_f0 denovo11320_f0 denovo11346_f0 denovo11398_f0 denovo11424_f0 denovo11450_f0 denovo11476_f0 denovo11554_f0 denovo11710_f0 denovo11736_f0 denovo11918_f0 denovo12074_f0 
SRR803483                     denovo20_f0    denovo98_f0    denovo176_f0   denovo228_f0   denovo280_f0   denovo384_f0   denovo436_f0   denovo462_f0   denovo540_f0   denovo592_f0   denovo618_f0   denovo800_f0   denovo1060_f0  denovo1086_f0  denovo1164_f0  denovo1242_f0  denovo1372_f0  denovo1398_f0  denovo1424_f0  denovo1502_f0  denovo1528_f0  denovo1658_f0  denovo1684_f0  denovo1762_f0  denovo1814_f0  denovo1944_f0  denovo1970_f0  denovo1996_f0  denovo2022_f0  denovo2126_f0  denovo2152_f0  denovo2204_f0  denovo2256_f0  denovo2282_f0  denovo2308_f0  denovo2464_f0  denovo2490_f0  denovo2516_f0  denovo2542_f0  denovo2594_f0  denovo2620_f0  denovo2646_f0  denovo2672_f0  denovo2776_f0  denovo2854_f0  denovo2932_f0  denovo3010_f0  denovo3166_f0  denovo3270_f0  denovo3322_f0  denovo3348_f0  denovo3452_f0  denovo3504_f0  denovo3608_f0  denovo3634_f0  denovo3686_f0  denovo3738_f0  denovo3764_f0  denovo3790_f0  denovo3816_f0  denovo3842_f0  denovo3868_f0  denovo3972_f0  denovo3998_f0  denovo4076_f0  denovo4206_f0  denovo4336_f0  denovo4362_f0  denovo4388_f0  denovo4414_f0  denovo4570_f0  denovo4648_f0  denovo4752_f0  denovo4778_f0  denovo4804_f0  denovo4856_f0  denovo4908_f0  denovo4934_f0  denovo4960_f0  denovo4986_f0  denovo5012_f0  denovo5220_f0  denovo5246_f0  denovo5272_f0  denovo5454_f0  denovo5610_f0  denovo5662_f0  denovo5974_f0  denovo6026_f0  denovo6078_f0  denovo6104_f0  denovo6130_f0  denovo6208_f0  denovo6234_f0  denovo6260_f0  denovo6286_f0  denovo6312_f0  denovo6390_f0  denovo6520_f0  denovo6572_f0  denovo6598_f0  denovo6624_f0  denovo6676_f0  denovo6702_f0  denovo6728_f0  denovo6754_f0  denovo6780_f0  denovo6988_f0  denovo7014_f0  denovo7092_f0  denovo7144_f0  denovo7170_f0  denovo7274_f0  denovo7300_f0  denovo7326_f0  denovo7352_f0  denovo7378_f0  denovo7404_f0  denovo7508_f0  denovo7534_f0  denovo7560_f0  denovo7638_f0  denovo7690_f0  denovo7716_f0  denovo7846_f0  denovo7924_f0  denovo8054_f0  denovo8080_f0  denovo8236_f0  denovo8262_f0  denovo8288_f0  denovo8314_f0  denovo8392_f0  denovo8418_f0  denovo8444_f0  denovo8470_f0  denovo8574_f0  denovo8600_f0  denovo8652_f0  denovo8678_f0  denovo8704_f0  denovo8782_f0  denovo8860_f0  denovo8912_f0  denovo8938_f0  denovo8964_f0  denovo8990_f0  denovo9016_f0  denovo9146_f0  denovo9172_f0  denovo9198_f0  denovo9302_f0  denovo9328_f0  denovo9354_f0  denovo9380_f0  denovo9406_f0  denovo9458_f0  denovo9510_f0  denovo9718_f0  denovo9744_f0  denovo9796_f0  denovo9822_f0  denovo9848_f0  denovo9900_f0  denovo9978_f0  denovo10212_f0 denovo10238_f0 denovo10290_f0 denovo10316_f0 denovo10368_f0 denovo10420_f0 denovo10446_f0 denovo10472_f0 denovo10524_f0 denovo10550_f0 denovo10628_f0 denovo10680_f0 denovo10732_f0 denovo10758_f0 denovo10784_f0 denovo10836_f0 denovo10862_f0 denovo10940_f0 denovo11018_f0 denovo11044_f0 denovo11096_f0 denovo11174_f0 denovo11200_f0 denovo11304_f0 denovo11330_f0 denovo11356_f0 denovo11408_f0 denovo11434_f0 denovo11460_f0 denovo11486_f0 denovo11564_f0 denovo11720_f0 denovo11746_f0 denovo11928_f0 denovo12084_f0 
FG120035                      denovo5_f0     denovo83_f0    denovo161_f0   denovo213_f0   denovo265_f0   denovo369_f0   denovo421_f0   denovo447_f0   denovo525_f0   denovo577_f0   denovo603_f0   denovo785_f0   denovo1045_f0  denovo1071_f0  denovo1149_f0  denovo1227_f0  denovo1357_f0  denovo1383_f0  denovo1409_f0  denovo1487_f0  denovo1513_f0  denovo1643_f0  denovo1669_f0  denovo1747_f0  denovo1799_f0  denovo1929_f0  denovo1955_f0  denovo1981_f0  denovo2007_f0  denovo2111_f0  denovo2137_f0  denovo2189_f0  denovo2241_f0  denovo2267_f0  denovo2293_f0  denovo2449_f0  denovo2475_f0  denovo2501_f0  denovo2527_f0  denovo2579_f0  denovo2605_f0  denovo2631_f0  denovo2657_f0  denovo2761_f0  denovo2839_f0  denovo2917_f0  denovo2995_f0  denovo3151_f0  denovo3255_f0  denovo3307_f0  denovo3333_f0  denovo3437_f0  denovo3489_f0  denovo3593_f0  denovo3619_f0  denovo3671_f0  denovo3723_f0  denovo3749_f0  denovo3775_f0  denovo3801_f0  denovo3827_f0  denovo3853_f0  denovo3957_f0  denovo3983_f0  denovo4061_f0  denovo4191_f0  denovo4321_f0  denovo4347_f0  denovo4373_f0  denovo4399_f0  denovo4555_f0  denovo4633_f0  denovo4737_f0  denovo4763_f0  denovo4789_f0  denovo4841_f0  denovo4893_f0  denovo4919_f0  denovo4945_f0  denovo4971_f0  denovo4997_f0  denovo5205_f0  denovo5231_f0  denovo5257_f0  denovo5439_f0  denovo5595_f0  denovo5647_f0  denovo5959_f0  denovo6011_f0  denovo6063_f0  denovo6089_f0  denovo6115_f0  denovo6193_f0  denovo6219_f0  denovo6245_f0  denovo6271_f0  denovo6297_f0  denovo6375_f0  denovo6505_f0  denovo6557_f0  denovo6583_f0  denovo6609_f0  denovo6661_f0  denovo6687_f0  denovo6713_f0  denovo6739_f0  denovo6765_f0  denovo6973_f0  denovo6999_f0  denovo7077_f0  denovo7129_f0  denovo7155_f0  denovo7259_f0  denovo7285_f0  denovo7311_f0  denovo7337_f0  denovo7363_f0  denovo7389_f0  denovo7493_f0  denovo7519_f0  denovo7545_f0  denovo7623_f0  denovo7675_f0  denovo7701_f0  denovo7831_f0  denovo7909_f0  denovo8039_f0  denovo8065_f0  denovo8221_f0  denovo8247_f0  denovo8273_f0  denovo8299_f0  denovo8377_f0  denovo8403_f0  denovo8429_f0  denovo8455_f0  denovo8559_f0  denovo8585_f0  denovo8637_f0  denovo8663_f0  denovo8689_f0  denovo8767_f0  denovo8845_f0  denovo8897_f0  denovo8923_f0  denovo8949_f0  denovo8975_f0  denovo9001_f0  denovo9131_f0  denovo9157_f0  denovo9183_f0  denovo9287_f0  denovo9313_f0  denovo9339_f0  denovo9365_f0  denovo9391_f0  denovo9443_f0  denovo9495_f0  denovo9703_f0  denovo9729_f0  denovo9781_f0  denovo9807_f0  denovo9833_f0  denovo9885_f0  denovo9963_f0  denovo10197_f0 denovo10223_f0 denovo10275_f0 denovo10301_f0 denovo10353_f0 denovo10405_f0 denovo10431_f0 denovo10457_f0 denovo10509_f0 denovo10535_f0 denovo10613_f0 denovo10665_f0 denovo10717_f0 denovo10743_f0 denovo10769_f0 denovo10821_f0 denovo10847_f0 denovo10925_f0 denovo11003_f0 denovo11029_f0 denovo11081_f0 denovo11159_f0 denovo11185_f0 denovo11289_f0 denovo11315_f0 denovo11341_f0 denovo11393_f0 denovo11419_f0 denovo11445_f0 denovo11471_f0 denovo11549_f0 denovo11705_f0 denovo11731_f0 denovo11913_f0 denovo12069_f0 
FG120046B                     denovo6_f0     denovo84_f0    denovo162_f0   denovo214_f0   denovo266_f0   denovo370_f0   denovo422_f0   denovo448_f0   denovo526_f0   denovo578_f0   denovo604_f0   denovo786_f0   denovo1046_f0  denovo1072_f0  denovo1150_f0  denovo1228_f0  denovo1358_f0  denovo1384_f0  denovo1410_f0  denovo1488_f0  denovo1514_f0  denovo1644_f0  denovo1670_f0  denovo1748_f0  denovo1800_f0  denovo1930_f0  denovo1956_f0  denovo1982_f0  denovo2008_f0  denovo2112_f0  denovo2138_f0  denovo2190_f0  denovo2242_f0  denovo2268_f0  denovo2294_f0  denovo2450_f0  denovo2476_f0  denovo2502_f0  denovo2528_f0  denovo2580_f0  denovo2606_f0  denovo2632_f0  denovo2658_f0  denovo2762_f0  denovo2840_f0  denovo2918_f0  denovo2996_f0  denovo3152_f0  denovo3256_f0  denovo3308_f0  denovo3334_f0  denovo3438_f0  denovo3490_f0  denovo3594_f0  denovo3620_f0  denovo3672_f0  denovo3724_f0  denovo3750_f0  denovo3776_f0  denovo3802_f0  denovo3828_f0  denovo3854_f0  denovo3958_f0  denovo3984_f0  denovo4062_f0  denovo4192_f0  denovo4322_f0  denovo4348_f0  denovo4374_f0  denovo4400_f0  denovo4556_f0  denovo4634_f0  denovo4738_f0  denovo4764_f0  denovo4790_f0  denovo4842_f0  denovo4894_f0  denovo4920_f0  denovo4946_f0  denovo4972_f0  denovo4998_f0  denovo5206_f0  denovo5232_f0  denovo5258_f0  denovo5440_f0  denovo5596_f0  denovo5648_f0  denovo5960_f0  denovo6012_f0  denovo6064_f0  denovo6090_f0  denovo6116_f0  denovo6194_f0  denovo6220_f0  denovo6246_f0  denovo6272_f0  denovo6298_f0  denovo6376_f0  denovo6506_f0  denovo6558_f0  denovo6584_f0  denovo6610_f0  denovo6662_f0  denovo6688_f0  denovo6714_f0  denovo6740_f0  denovo6766_f0  denovo6974_f0  denovo7000_f0  denovo7078_f0  denovo7130_f0  denovo7156_f0  denovo7260_f0  denovo7286_f0  denovo7312_f0  denovo7338_f0  denovo7364_f0  denovo7390_f0  denovo7494_f0  denovo7520_f0  denovo7546_f0  denovo7624_f0  denovo7676_f0  denovo7702_f0  denovo7832_f0  denovo7910_f0  denovo8040_f0  denovo8066_f0  denovo8222_f0  denovo8248_f0  denovo8274_f0  denovo8300_f0  denovo8378_f0  denovo8404_f0  denovo8430_f0  denovo8456_f0  denovo8560_f0  denovo8586_f0  denovo8638_f0  denovo8664_f0  denovo8690_f0  denovo8768_f0  denovo8846_f0  denovo8898_f0  denovo8924_f0  denovo8950_f0  denovo8976_f0  denovo9002_f0  denovo9132_f0  denovo9158_f0  denovo9184_f0  denovo9288_f0  denovo9314_f0  denovo9340_f0  denovo9366_f0  denovo9392_f0  denovo9444_f0  denovo9496_f0  denovo9704_f0  denovo9730_f0  denovo9782_f0  denovo9808_f0  denovo9834_f0  denovo9886_f0  denovo9964_f0  denovo10198_f0 denovo10224_f0 denovo10276_f0 denovo10302_f0 denovo10354_f0 denovo10406_f0 denovo10432_f0 denovo10458_f0 denovo10510_f0 denovo10536_f0 denovo10614_f0 denovo10666_f0 denovo10718_f0 denovo10744_f0 denovo10770_f0 denovo10822_f0 denovo10848_f0 denovo10926_f0 denovo11004_f0 denovo11030_f0 denovo11082_f0 denovo11160_f0 denovo11186_f0 denovo11290_f0 denovo11316_f0 denovo11342_f0 denovo11394_f0 denovo11420_f0 denovo11446_f0 denovo11472_f0 denovo11550_f0 denovo11706_f0 denovo11732_f0 denovo11914_f0 denovo12070_f0 
GNV129007                     denovo15_f0    denovo93_f0    denovo171_f0   denovo223_f0   denovo275_f0   denovo379_f0   denovo431_f0   denovo457_f0   denovo535_f0   denovo587_f0   denovo613_f0   denovo795_f0   denovo1055_f0  denovo1081_f0  denovo1159_f0  denovo1237_f0  denovo1367_f0  denovo1393_f0  denovo1419_f0  denovo1497_f0  denovo1523_f0  denovo1653_f0  denovo1679_f0  denovo1757_f0  denovo1809_f0  denovo1939_f0  denovo1965_f0  denovo1991_f0  denovo2017_f0  denovo2121_f0  denovo2147_f0  denovo2199_f0  denovo2251_f0  denovo2277_f0  denovo2303_f0  denovo2459_f0  denovo2485_f0  denovo2511_f0  denovo2537_f0  denovo2589_f0  denovo2615_f0  denovo2641_f0  denovo2667_f0  denovo2771_f0  denovo2849_f0  denovo2927_f0  denovo3005_f0  denovo3161_f0  denovo3265_f0  denovo3317_f0  denovo3343_f0  denovo3447_f0  denovo3499_f0  denovo3603_f0  denovo3629_f0  denovo3681_f0  denovo3733_f0  denovo3759_f0  denovo3785_f0  denovo3811_f0  denovo3837_f0  denovo3863_f0  denovo3967_f0  denovo3993_f0  denovo4071_f0  denovo4201_f0  denovo4331_f0  denovo4357_f0  denovo4383_f0  denovo4409_f0  denovo4565_f0  denovo4643_f0  denovo4747_f0  denovo4773_f0  denovo4799_f0  denovo4851_f0  denovo4903_f0  denovo4929_f0  denovo4955_f0  denovo4981_f0  denovo5007_f0  denovo5215_f0  denovo5241_f0  denovo5267_f0  denovo5449_f0  denovo5605_f0  denovo5657_f0  denovo5969_f0  denovo6021_f0  denovo6073_f0  denovo6099_f0  denovo6125_f0  denovo6203_f0  denovo6229_f0  denovo6255_f0  denovo6281_f0  denovo6307_f0  denovo6385_f0  denovo6515_f0  denovo6567_f0  denovo6593_f0  denovo6619_f0  denovo6671_f0  denovo6697_f0  denovo6723_f0  denovo6749_f0  denovo6775_f0  denovo6983_f0  denovo7009_f0  denovo7087_f0  denovo7139_f0  denovo7165_f0  denovo7269_f0  denovo7295_f0  denovo7321_f0  denovo7347_f0  denovo7373_f0  denovo7399_f0  denovo7503_f0  denovo7529_f0  denovo7555_f0  denovo7633_f0  denovo7685_f0  denovo7711_f0  denovo7841_f0  denovo7919_f0  denovo8049_f0  denovo8075_f0  denovo8231_f0  denovo8257_f0  denovo8283_f0  denovo8309_f0  denovo8387_f0  denovo8413_f0  denovo8439_f0  denovo8465_f0  denovo8569_f0  denovo8595_f0  denovo8647_f0  denovo8673_f0  denovo8699_f0  denovo8777_f0  denovo8855_f0  denovo8907_f0  denovo8933_f0  denovo8959_f0  denovo8985_f0  denovo9011_f0  denovo9141_f0  denovo9167_f0  denovo9193_f0  denovo9297_f0  denovo9323_f0  denovo9349_f0  denovo9375_f0  denovo9401_f0  denovo9453_f0  denovo9505_f0  denovo9713_f0  denovo9739_f0  denovo9791_f0  denovo9817_f0  denovo9843_f0  denovo9895_f0  denovo9973_f0  denovo10207_f0 denovo10233_f0 denovo10285_f0 denovo10311_f0 denovo10363_f0 denovo10415_f0 denovo10441_f0 denovo10467_f0 denovo10519_f0 denovo10545_f0 denovo10623_f0 denovo10675_f0 denovo10727_f0 denovo10753_f0 denovo10779_f0 denovo10831_f0 denovo10857_f0 denovo10935_f0 denovo11013_f0 denovo11039_f0 denovo11091_f0 denovo11169_f0 denovo11195_f0 denovo11299_f0 denovo11325_f0 denovo11351_f0 denovo11403_f0 denovo11429_f0 denovo11455_f0 denovo11481_f0 denovo11559_f0 denovo11715_f0 denovo11741_f0 denovo11923_f0 denovo12079_f0 
SW130126                      denovo24_f0    denovo102_f0   denovo180_f0   denovo232_f0   denovo284_f0   denovo388_f0   denovo440_f0   denovo466_f0   denovo544_f0   denovo596_f0   denovo622_f0   denovo804_f0   denovo1064_f0  denovo1090_f0  denovo1168_f0  denovo1246_f0  denovo1376_f0  denovo1402_f0  denovo1428_f0  denovo1506_f0  denovo1532_f0  denovo1662_f0  denovo1688_f0  denovo1766_f0  denovo1818_f0  denovo1948_f0  denovo1974_f0  denovo2000_f0  denovo2026_f0  denovo2130_f0  denovo2156_f0  denovo2208_f0  denovo2260_f0  denovo2286_f0  denovo2312_f0  denovo2468_f0  denovo2494_f0  denovo2520_f0  denovo2546_f0  denovo2598_f0  denovo2624_f0  denovo2650_f0  denovo2676_f0  denovo2780_f0  denovo2858_f0  denovo2936_f0  denovo3014_f0  denovo3170_f0  denovo3274_f0  denovo3326_f0  denovo3352_f0  denovo3456_f0  denovo3508_f0  denovo3612_f0  denovo3638_f0  denovo3690_f0  denovo3742_f0  denovo3768_f0  denovo3794_f0  denovo3820_f0  denovo3846_f0  denovo3872_f0  denovo3976_f0  denovo4002_f0  denovo4080_f0  denovo4210_f0  denovo4340_f0  denovo4366_f0  denovo4392_f0  denovo4418_f0  denovo4574_f0  denovo4652_f0  denovo4756_f0  denovo4782_f0  denovo4808_f0  denovo4860_f0  denovo4912_f0  denovo4938_f0  denovo4964_f0  denovo4990_f0  denovo5016_f0  denovo5224_f0  denovo5250_f0  denovo5276_f0  denovo5458_f0  denovo5614_f0  denovo5666_f0  denovo5978_f0  denovo6030_f0  denovo6082_f0  denovo6108_f0  denovo6134_f0  denovo6212_f0  denovo6238_f0  denovo6264_f0  denovo6290_f0  denovo6316_f0  denovo6394_f0  denovo6524_f0  denovo6576_f0  denovo6602_f0  denovo6628_f0  denovo6680_f0  denovo6706_f0  denovo6732_f0  denovo6758_f0  denovo6784_f0  denovo6992_f0  denovo7018_f0  denovo7096_f0  denovo7148_f0  denovo7174_f0  denovo7278_f0  denovo7304_f0  denovo7330_f0  denovo7356_f0  denovo7382_f0  denovo7408_f0  denovo7512_f0  denovo7538_f0  denovo7564_f0  denovo7642_f0  denovo7694_f0  denovo7720_f0  denovo7850_f0  denovo7928_f0  denovo8058_f0  denovo8084_f0  denovo8240_f0  denovo8266_f0  denovo8292_f0  denovo8318_f0  denovo8396_f0  denovo8422_f0  denovo8448_f0  denovo8474_f0  denovo8578_f0  denovo8604_f0  denovo8656_f0  denovo8682_f0  denovo8708_f0  denovo8786_f0  denovo8864_f0  denovo8916_f0  denovo8942_f0  denovo8968_f0  denovo8994_f0  denovo9020_f0  denovo9150_f0  denovo9176_f0  denovo9202_f0  denovo9306_f0  denovo9332_f0  denovo9358_f0  denovo9384_f0  denovo9410_f0  denovo9462_f0  denovo9514_f0  denovo9722_f0  denovo9748_f0  denovo9800_f0  denovo9826_f0  denovo9852_f0  denovo9904_f0  denovo9982_f0  denovo10216_f0 denovo10242_f0 denovo10294_f0 denovo10320_f0 denovo10372_f0 denovo10424_f0 denovo10450_f0 denovo10476_f0 denovo10528_f0 denovo10554_f0 denovo10632_f0 denovo10684_f0 denovo10736_f0 denovo10762_f0 denovo10788_f0 denovo10840_f0 denovo10866_f0 denovo10944_f0 denovo11022_f0 denovo11048_f0 denovo11100_f0 denovo11178_f0 denovo11204_f0 denovo11308_f0 denovo11334_f0 denovo11360_f0 denovo11412_f0 denovo11438_f0 denovo11464_f0 denovo11490_f0 denovo11568_f0 denovo11724_f0 denovo11750_f0 denovo11932_f0 denovo12088_f0 
SW130103                      denovo23_f0    denovo101_f0   denovo179_f0   denovo231_f0   denovo283_f0   denovo387_f0   denovo439_f0   denovo465_f0   denovo543_f0   denovo595_f0   denovo621_f0   denovo803_f0   denovo1063_f0  denovo1089_f0  denovo1167_f0  denovo1245_f0  denovo1375_f0  denovo1401_f0  denovo1427_f0  denovo1505_f0  denovo1531_f0  denovo1661_f0  denovo1687_f0  denovo1765_f0  denovo1817_f0  denovo1947_f0  denovo1973_f0  denovo1999_f0  denovo2025_f0  denovo2129_f0  denovo2155_f0  denovo2207_f0  denovo2259_f0  denovo2285_f0  denovo2311_f0  denovo2467_f0  denovo2493_f0  denovo2519_f0  denovo2545_f0  denovo2597_f0  denovo2623_f0  denovo2649_f0  denovo2675_f0  denovo2779_f0  denovo2857_f0  denovo2935_f0  denovo3013_f0  denovo3169_f0  denovo3273_f0  denovo3325_f0  denovo3351_f0  denovo3455_f0  denovo3507_f0  denovo3611_f0  denovo3637_f0  denovo3689_f0  denovo3741_f0  denovo3767_f0  denovo3793_f0  denovo3819_f0  denovo3845_f0  denovo3871_f0  denovo3975_f0  denovo4001_f0  denovo4079_f0  denovo4209_f0  denovo4339_f0  denovo4365_f0  denovo4391_f0  denovo4417_f0  denovo4573_f0  denovo4651_f0  denovo4755_f0  denovo4781_f0  denovo4807_f0  denovo4859_f0  denovo4911_f0  denovo4937_f0  denovo4963_f0  denovo4989_f0  denovo5015_f0  denovo5223_f0  denovo5249_f0  denovo5275_f0  denovo5457_f0  denovo5613_f0  denovo5665_f0  denovo5977_f0  denovo6029_f0  denovo6081_f0  denovo6107_f0  denovo6133_f0  denovo6211_f0  denovo6237_f0  denovo6263_f0  denovo6289_f0  denovo6315_f0  denovo6393_f0  denovo6523_f0  denovo6575_f0  denovo6601_f0  denovo6627_f0  denovo6679_f0  denovo6705_f0  denovo6731_f0  denovo6757_f0  denovo6783_f0  denovo6991_f0  denovo7017_f0  denovo7095_f0  denovo7147_f0  denovo7173_f0  denovo7277_f0  denovo7303_f0  denovo7329_f0  denovo7355_f0  denovo7381_f0  denovo7407_f0  denovo7511_f0  denovo7537_f0  denovo7563_f0  denovo7641_f0  denovo7693_f0  denovo7719_f0  denovo7849_f0  denovo7927_f0  denovo8057_f0  denovo8083_f0  denovo8239_f0  denovo8265_f0  denovo8291_f0  denovo8317_f0  denovo8395_f0  denovo8421_f0  denovo8447_f0  denovo8473_f0  denovo8577_f0  denovo8603_f0  denovo8655_f0  denovo8681_f0  denovo8707_f0  denovo8785_f0  denovo8863_f0  denovo8915_f0  denovo8941_f0  denovo8967_f0  denovo8993_f0  denovo9019_f0  denovo9149_f0  denovo9175_f0  denovo9201_f0  denovo9305_f0  denovo9331_f0  denovo9357_f0  denovo9383_f0  denovo9409_f0  denovo9461_f0  denovo9513_f0  denovo9721_f0  denovo9747_f0  denovo9799_f0  denovo9825_f0  denovo9851_f0  denovo9903_f0  denovo9981_f0  denovo10215_f0 denovo10241_f0 denovo10293_f0 denovo10319_f0 denovo10371_f0 denovo10423_f0 denovo10449_f0 denovo10475_f0 denovo10527_f0 denovo10553_f0 denovo10631_f0 denovo10683_f0 denovo10735_f0 denovo10761_f0 denovo10787_f0 denovo10839_f0 denovo10865_f0 denovo10943_f0 denovo11021_f0 denovo11047_f0 denovo11099_f0 denovo11177_f0 denovo11203_f0 denovo11307_f0 denovo11333_f0 denovo11359_f0 denovo11411_f0 denovo11437_f0 denovo11463_f0 denovo11489_f0 denovo11567_f0 denovo11723_f0 denovo11749_f0 denovo11931_f0 denovo12087_f0 
Callid                        denovo1_f0     denovo79_f0    denovo157_f0   denovo209_f0   denovo261_f0   denovo365_f0   denovo417_f0   denovo443_f0   denovo521_f0   denovo573_f0   denovo599_f0   denovo781_f0   denovo1041_f0  denovo1067_f0  denovo1145_f0  denovo1223_f0  denovo1353_f0  denovo1379_f0  denovo1405_f0  denovo1483_f0  denovo1509_f0  denovo1639_f0  denovo1665_f0  denovo1743_f0  denovo1795_f0  denovo1925_f0  denovo1951_f0  denovo1977_f0  denovo2003_f0  denovo2107_f0  denovo2133_f0  denovo2185_f0  denovo2237_f0  denovo2263_f0  denovo2289_f0  denovo2445_f0  denovo2471_f0  denovo2497_f0  denovo2523_f0  denovo2575_f0  denovo2601_f0  denovo2627_f0  denovo2653_f0  denovo2757_f0  denovo2835_f0  denovo2913_f0  denovo2991_f0  denovo3147_f0  denovo3251_f0  denovo3303_f0  denovo3329_f0  denovo3433_f0  denovo3485_f0  denovo3589_f0  denovo3615_f0  denovo3667_f0  denovo3719_f0  denovo3745_f0  denovo3771_f0  denovo3797_f0  denovo3823_f0  denovo3849_f0  denovo3953_f0  denovo3979_f0  denovo4057_f0  denovo4187_f0  denovo4317_f0  denovo4343_f0  denovo4369_f0  denovo4395_f0  denovo4551_f0  denovo4629_f0  denovo4733_f0  denovo4759_f0  denovo4785_f0  denovo4837_f0  denovo4889_f0  denovo4915_f0  denovo4941_f0  denovo4967_f0  denovo4993_f0  denovo5201_f0  denovo5227_f0  denovo5253_f0  denovo5435_f0  denovo5591_f0  denovo5643_f0  denovo5955_f0  denovo6007_f0  denovo6059_f0  denovo6085_f0  denovo6111_f0  denovo6189_f0  denovo6215_f0  denovo6241_f0  denovo6267_f0  denovo6293_f0  denovo6371_f0  denovo6501_f0  denovo6553_f0  denovo6579_f0  denovo6605_f0  denovo6657_f0  denovo6683_f0  denovo6709_f0  denovo6735_f0  denovo6761_f0  denovo6969_f0  denovo6995_f0  denovo7073_f0  denovo7125_f0  denovo7151_f0  denovo7255_f0  denovo7281_f0  denovo7307_f0  denovo7333_f0  denovo7359_f0  denovo7385_f0  denovo7489_f0  denovo7515_f0  denovo7541_f0  denovo7619_f0  denovo7671_f0  denovo7697_f0  denovo7827_f0  denovo7905_f0  denovo8035_f0  denovo8061_f0  denovo8217_f0  denovo8243_f0  denovo8269_f0  denovo8295_f0  denovo8373_f0  denovo8399_f0  denovo8425_f0  denovo8451_f0  denovo8555_f0  denovo8581_f0  denovo8633_f0  denovo8659_f0  denovo8685_f0  denovo8763_f0  denovo8841_f0  denovo8893_f0  denovo8919_f0  denovo8945_f0  denovo8971_f0  denovo8997_f0  denovo9127_f0  denovo9153_f0  denovo9179_f0  denovo9283_f0  denovo9309_f0  denovo9335_f0  denovo9361_f0  denovo9387_f0  denovo9439_f0  denovo9491_f0  denovo9699_f0  denovo9725_f0  denovo9777_f0  denovo9803_f0  denovo9829_f0  denovo9881_f0  denovo9959_f0  denovo10193_f0 denovo10219_f0 denovo10271_f0 denovo10297_f0 denovo10349_f0 denovo10401_f0 denovo10427_f0 denovo10453_f0 denovo10505_f0 denovo10531_f0 denovo10609_f0 denovo10661_f0 denovo10713_f0 denovo10739_f0 denovo10765_f0 denovo10817_f0 denovo10843_f0 denovo10921_f0 denovo10999_f0 denovo11025_f0 denovo11077_f0 denovo11155_f0 denovo11181_f0 denovo11285_f0 denovo11311_f0 denovo11337_f0 denovo11389_f0 denovo11415_f0 denovo11441_f0 denovo11467_f0 denovo11545_f0 denovo11701_f0 denovo11727_f0 denovo11909_f0 denovo12065_f0 
FG120070B                     denovo8_f0     denovo86_f0    denovo164_f0   denovo216_f0   denovo268_f0   denovo372_f0   denovo424_f0   denovo450_f0   denovo528_f0   denovo580_f0   denovo606_f0   denovo788_f0   denovo1048_f0  denovo1074_f0  denovo1152_f0  denovo1230_f0  denovo1360_f0  denovo1386_f0  denovo1412_f0  denovo1490_f0  denovo1516_f0  denovo1646_f0  denovo1672_f0  denovo1750_f0  denovo1802_f0  denovo1932_f0  denovo1958_f0  denovo1984_f0  denovo2010_f0  denovo2114_f0  denovo2140_f0  denovo2192_f0  denovo2244_f0  denovo2270_f0  denovo2296_f0  denovo2452_f0  denovo2478_f0  denovo2504_f0  denovo2530_f0  denovo2582_f0  denovo2608_f0  denovo2634_f0  denovo2660_f0  denovo2764_f0  denovo2842_f0  denovo2920_f0  denovo2998_f0  denovo3154_f0  denovo3258_f0  denovo3310_f0  denovo3336_f0  denovo3440_f0  denovo3492_f0  denovo3596_f0  denovo3622_f0  denovo3674_f0  denovo3726_f0  denovo3752_f0  denovo3778_f0  denovo3804_f0  denovo3830_f0  denovo3856_f0  denovo3960_f0  denovo3986_f0  denovo4064_f0  denovo4194_f0  denovo4324_f0  denovo4350_f0  denovo4376_f0  denovo4402_f0  denovo4558_f0  denovo4636_f0  denovo4740_f0  denovo4766_f0  denovo4792_f0  denovo4844_f0  denovo4896_f0  denovo4922_f0  denovo4948_f0  denovo4974_f0  denovo5000_f0  denovo5208_f0  denovo5234_f0  denovo5260_f0  denovo5442_f0  denovo5598_f0  denovo5650_f0  denovo5962_f0  denovo6014_f0  denovo6066_f0  denovo6092_f0  denovo6118_f0  denovo6196_f0  denovo6222_f0  denovo6248_f0  denovo6274_f0  denovo6300_f0  denovo6378_f0  denovo6508_f0  denovo6560_f0  denovo6586_f0  denovo6612_f0  denovo6664_f0  denovo6690_f0  denovo6716_f0  denovo6742_f0  denovo6768_f0  denovo6976_f0  denovo7002_f0  denovo7080_f0  denovo7132_f0  denovo7158_f0  denovo7262_f0  denovo7288_f0  denovo7314_f0  denovo7340_f0  denovo7366_f0  denovo7392_f0  denovo7496_f0  denovo7522_f0  denovo7548_f0  denovo7626_f0  denovo7678_f0  denovo7704_f0  denovo7834_f0  denovo7912_f0  denovo8042_f0  denovo8068_f0  denovo8224_f0  denovo8250_f0  denovo8276_f0  denovo8302_f0  denovo8380_f0  denovo8406_f0  denovo8432_f0  denovo8458_f0  denovo8562_f0  denovo8588_f0  denovo8640_f0  denovo8666_f0  denovo8692_f0  denovo8770_f0  denovo8848_f0  denovo8900_f0  denovo8926_f0  denovo8952_f0  denovo8978_f0  denovo9004_f0  denovo9134_f0  denovo9160_f0  denovo9186_f0  denovo9290_f0  denovo9316_f0  denovo9342_f0  denovo9368_f0  denovo9394_f0  denovo9446_f0  denovo9498_f0  denovo9706_f0  denovo9732_f0  denovo9784_f0  denovo9810_f0  denovo9836_f0  denovo9888_f0  denovo9966_f0  denovo10200_f0 denovo10226_f0 denovo10278_f0 denovo10304_f0 denovo10356_f0 denovo10408_f0 denovo10434_f0 denovo10460_f0 denovo10512_f0 denovo10538_f0 denovo10616_f0 denovo10668_f0 denovo10720_f0 denovo10746_f0 denovo10772_f0 denovo10824_f0 denovo10850_f0 denovo10928_f0 denovo11006_f0 denovo11032_f0 denovo11084_f0 denovo11162_f0 denovo11188_f0 denovo11292_f0 denovo11318_f0 denovo11344_f0 denovo11396_f0 denovo11422_f0 denovo11448_f0 denovo11474_f0 denovo11552_f0 denovo11708_f0 denovo11734_f0 denovo11916_f0 denovo12072_f0 
SW130007                      denovo22_f0    denovo100_f0   denovo178_f0   denovo230_f0   denovo282_f0   denovo386_f0   denovo438_f0   denovo464_f0   denovo542_f0   denovo594_f0   denovo620_f0   denovo802_f0   denovo1062_f0  denovo1088_f0  denovo1166_f0  denovo1244_f0  denovo1374_f0  denovo1400_f0  denovo1426_f0  denovo1504_f0  denovo1530_f0  denovo1660_f0  denovo1686_f0  denovo1764_f0  denovo1816_f0  denovo1946_f0  denovo1972_f0  denovo1998_f0  denovo2024_f0  denovo2128_f0  denovo2154_f0  denovo2206_f0  denovo2258_f0  denovo2284_f0  denovo2310_f0  denovo2466_f0  denovo2492_f0  denovo2518_f0  denovo2544_f0  denovo2596_f0  denovo2622_f0  denovo2648_f0  denovo2674_f0  denovo2778_f0  denovo2856_f0  denovo2934_f0  denovo3012_f0  denovo3168_f0  denovo3272_f0  denovo3324_f0  denovo3350_f0  denovo3454_f0  denovo3506_f0  denovo3610_f0  denovo3636_f0  denovo3688_f0  denovo3740_f0  denovo3766_f0  denovo3792_f0  denovo3818_f0  denovo3844_f0  denovo3870_f0  denovo3974_f0  denovo4000_f0  denovo4078_f0  denovo4208_f0  denovo4338_f0  denovo4364_f0  denovo4390_f0  denovo4416_f0  denovo4572_f0  denovo4650_f0  denovo4754_f0  denovo4780_f0  denovo4806_f0  denovo4858_f0  denovo4910_f0  denovo4936_f0  denovo4962_f0  denovo4988_f0  denovo5014_f0  denovo5222_f0  denovo5248_f0  denovo5274_f0  denovo5456_f0  denovo5612_f0  denovo5664_f0  denovo5976_f0  denovo6028_f0  denovo6080_f0  denovo6106_f0  denovo6132_f0  denovo6210_f0  denovo6236_f0  denovo6262_f0  denovo6288_f0  denovo6314_f0  denovo6392_f0  denovo6522_f0  denovo6574_f0  denovo6600_f0  denovo6626_f0  denovo6678_f0  denovo6704_f0  denovo6730_f0  denovo6756_f0  denovo6782_f0  denovo6990_f0  denovo7016_f0  denovo7094_f0  denovo7146_f0  denovo7172_f0  denovo7276_f0  denovo7302_f0  denovo7328_f0  denovo7354_f0  denovo7380_f0  denovo7406_f0  denovo7510_f0  denovo7536_f0  denovo7562_f0  denovo7640_f0  denovo7692_f0  denovo7718_f0  denovo7848_f0  denovo7926_f0  denovo8056_f0  denovo8082_f0  denovo8238_f0  denovo8264_f0  denovo8290_f0  denovo8316_f0  denovo8394_f0  denovo8420_f0  denovo8446_f0  denovo8472_f0  denovo8576_f0  denovo8602_f0  denovo8654_f0  denovo8680_f0  denovo8706_f0  denovo8784_f0  denovo8862_f0  denovo8914_f0  denovo8940_f0  denovo8966_f0  denovo8992_f0  denovo9018_f0  denovo9148_f0  denovo9174_f0  denovo9200_f0  denovo9304_f0  denovo9330_f0  denovo9356_f0  denovo9382_f0  denovo9408_f0  denovo9460_f0  denovo9512_f0  denovo9720_f0  denovo9746_f0  denovo9798_f0  denovo9824_f0  denovo9850_f0  denovo9902_f0  denovo9980_f0  denovo10214_f0 denovo10240_f0 denovo10292_f0 denovo10318_f0 denovo10370_f0 denovo10422_f0 denovo10448_f0 denovo10474_f0 denovo10526_f0 denovo10552_f0 denovo10630_f0 denovo10682_f0 denovo10734_f0 denovo10760_f0 denovo10786_f0 denovo10838_f0 denovo10864_f0 denovo10942_f0 denovo11020_f0 denovo11046_f0 denovo11098_f0 denovo11176_f0 denovo11202_f0 denovo11306_f0 denovo11332_f0 denovo11358_f0 denovo11410_f0 denovo11436_f0 denovo11462_f0 denovo11488_f0 denovo11566_f0 denovo11722_f0 denovo11748_f0 denovo11930_f0 denovo12086_f0 
GNV120032                     denovo14_f0    denovo92_f0    denovo170_f0   denovo222_f0   denovo274_f0   denovo378_f0   denovo430_f0   denovo456_f0   denovo534_f0   denovo586_f0   denovo612_f0   denovo794_f0   denovo1054_f0  denovo1080_f0  denovo1158_f0  denovo1236_f0  denovo1366_f0  denovo1392_f0  denovo1418_f0  denovo1496_f0  denovo1522_f0  denovo1652_f0  denovo1678_f0  denovo1756_f0  denovo1808_f0  denovo1938_f0  denovo1964_f0  denovo1990_f0  denovo2016_f0  denovo2120_f0  denovo2146_f0  denovo2198_f0  denovo2250_f0  denovo2276_f0  denovo2302_f0  denovo2458_f0  denovo2484_f0  denovo2510_f0  denovo2536_f0  denovo2588_f0  denovo2614_f0  denovo2640_f0  denovo2666_f0  denovo2770_f0  denovo2848_f0  denovo2926_f0  denovo3004_f0  denovo3160_f0  denovo3264_f0  denovo3316_f0  denovo3342_f0  denovo3446_f0  denovo3498_f0  denovo3602_f0  denovo3628_f0  denovo3680_f0  denovo3732_f0  denovo3758_f0  denovo3784_f0  denovo3810_f0  denovo3836_f0  denovo3862_f0  denovo3966_f0  denovo3992_f0  denovo4070_f0  denovo4200_f0  denovo4330_f0  denovo4356_f0  denovo4382_f0  denovo4408_f0  denovo4564_f0  denovo4642_f0  denovo4746_f0  denovo4772_f0  denovo4798_f0  denovo4850_f0  denovo4902_f0  denovo4928_f0  denovo4954_f0  denovo4980_f0  denovo5006_f0  denovo5214_f0  denovo5240_f0  denovo5266_f0  denovo5448_f0  denovo5604_f0  denovo5656_f0  denovo5968_f0  denovo6020_f0  denovo6072_f0  denovo6098_f0  denovo6124_f0  denovo6202_f0  denovo6228_f0  denovo6254_f0  denovo6280_f0  denovo6306_f0  denovo6384_f0  denovo6514_f0  denovo6566_f0  denovo6592_f0  denovo6618_f0  denovo6670_f0  denovo6696_f0  denovo6722_f0  denovo6748_f0  denovo6774_f0  denovo6982_f0  denovo7008_f0  denovo7086_f0  denovo7138_f0  denovo7164_f0  denovo7268_f0  denovo7294_f0  denovo7320_f0  denovo7346_f0  denovo7372_f0  denovo7398_f0  denovo7502_f0  denovo7528_f0  denovo7554_f0  denovo7632_f0  denovo7684_f0  denovo7710_f0  denovo7840_f0  denovo7918_f0  denovo8048_f0  denovo8074_f0  denovo8230_f0  denovo8256_f0  denovo8282_f0  denovo8308_f0  denovo8386_f0  denovo8412_f0  denovo8438_f0  denovo8464_f0  denovo8568_f0  denovo8594_f0  denovo8646_f0  denovo8672_f0  denovo8698_f0  denovo8776_f0  denovo8854_f0  denovo8906_f0  denovo8932_f0  denovo8958_f0  denovo8984_f0  denovo9010_f0  denovo9140_f0  denovo9166_f0  denovo9192_f0  denovo9296_f0  denovo9322_f0  denovo9348_f0  denovo9374_f0  denovo9400_f0  denovo9452_f0  denovo9504_f0  denovo9712_f0  denovo9738_f0  denovo9790_f0  denovo9816_f0  denovo9842_f0  denovo9894_f0  denovo9972_f0  denovo10206_f0 denovo10232_f0 denovo10284_f0 denovo10310_f0 denovo10362_f0 denovo10414_f0 denovo10440_f0 denovo10466_f0 denovo10518_f0 denovo10544_f0 denovo10622_f0 denovo10674_f0 denovo10726_f0 denovo10752_f0 denovo10778_f0 denovo10830_f0 denovo10856_f0 denovo10934_f0 denovo11012_f0 denovo11038_f0 denovo11090_f0 denovo11168_f0 denovo11194_f0 denovo11298_f0 denovo11324_f0 denovo11350_f0 denovo11402_f0 denovo11428_f0 denovo11454_f0 denovo11480_f0 denovo11558_f0 denovo11714_f0 denovo11740_f0 denovo11922_f0 denovo12078_f0 
PXYLO                         denovo18_f0    denovo96_f0    denovo174_f0   denovo226_f0   denovo278_f0   denovo382_f0   denovo434_f0   denovo460_f0   denovo538_f0   denovo590_f0   denovo616_f0   denovo798_f0   denovo1058_f0  denovo1084_f0  denovo1162_f0  denovo1240_f0  denovo1370_f0  denovo1396_f0  denovo1422_f0  denovo1500_f0  denovo1526_f0  denovo1656_f0  denovo1682_f0  denovo1760_f0  denovo1812_f0  denovo1942_f0  denovo1968_f0  denovo1994_f0  denovo2020_f0  denovo2124_f0  denovo2150_f0  denovo2202_f0  denovo2254_f0  denovo2280_f0  denovo2306_f0  denovo2462_f0  denovo2488_f0  denovo2514_f0  denovo2540_f0  denovo2592_f0  denovo2618_f0  denovo2644_f0  denovo2670_f0  denovo2774_f0  denovo2852_f0  denovo2930_f0  denovo3008_f0  denovo3164_f0  denovo3268_f0  denovo3320_f0  denovo3346_f0  denovo3450_f0  denovo3502_f0  denovo3606_f0  denovo3632_f0  denovo3684_f0  denovo3736_f0  denovo3762_f0  denovo3788_f0  denovo3814_f0  denovo3840_f0  denovo3866_f0  denovo3970_f0  denovo3996_f0  denovo4074_f0  denovo4204_f0  denovo4334_f0  denovo4360_f0  denovo4386_f0  denovo4412_f0  denovo4568_f0  denovo4646_f0  denovo4750_f0  denovo4776_f0  denovo4802_f0  denovo4854_f0  denovo4906_f0  denovo4932_f0  denovo4958_f0  denovo4984_f0  denovo5010_f0  denovo5218_f0  denovo5244_f0  denovo5270_f0  denovo5452_f0  denovo5608_f0  denovo5660_f0  denovo5972_f0  denovo6024_f0  denovo6076_f0  denovo6102_f0  denovo6128_f0  denovo6206_f0  denovo6232_f0  denovo6258_f0  denovo6284_f0  denovo6310_f0  denovo6388_f0  denovo6518_f0  denovo6570_f0  denovo6596_f0  denovo6622_f0  denovo6674_f0  denovo6700_f0  denovo6726_f0  denovo6752_f0  denovo6778_f0  denovo6986_f0  denovo7012_f0  denovo7090_f0  denovo7142_f0  denovo7168_f0  denovo7272_f0  denovo7298_f0  denovo7324_f0  denovo7350_f0  denovo7376_f0  denovo7402_f0  denovo7506_f0  denovo7532_f0  denovo7558_f0  denovo7636_f0  denovo7688_f0  denovo7714_f0  denovo7844_f0  denovo7922_f0  denovo8052_f0  denovo8078_f0  denovo8234_f0  denovo8260_f0  denovo8286_f0  denovo8312_f0  denovo8390_f0  denovo8416_f0  denovo8442_f0  denovo8468_f0  denovo8572_f0  denovo8598_f0  denovo8650_f0  denovo8676_f0  denovo8702_f0  denovo8780_f0  denovo8858_f0  denovo8910_f0  denovo8936_f0  denovo8962_f0  denovo8988_f0  denovo9014_f0  denovo9144_f0  denovo9170_f0  denovo9196_f0  denovo9300_f0  denovo9326_f0  denovo9352_f0  denovo9378_f0  denovo9404_f0  denovo9456_f0  denovo9508_f0  denovo9716_f0  denovo9742_f0  denovo9794_f0  denovo9820_f0  denovo9846_f0  denovo9898_f0  denovo9976_f0  denovo10210_f0 denovo10236_f0 denovo10288_f0 denovo10314_f0 denovo10366_f0 denovo10418_f0 denovo10444_f0 denovo10470_f0 denovo10522_f0 denovo10548_f0 denovo10626_f0 denovo10678_f0 denovo10730_f0 denovo10756_f0 denovo10782_f0 denovo10834_f0 denovo10860_f0 denovo10938_f0 denovo11016_f0 denovo11042_f0 denovo11094_f0 denovo11172_f0 denovo11198_f0 denovo11302_f0 denovo11328_f0 denovo11354_f0 denovo11406_f0 denovo11432_f0 denovo11458_f0 denovo11484_f0 denovo11562_f0 denovo11718_f0 denovo11744_f0 denovo11926_f0 denovo12082_f0 
FG120079                      denovo11_f0    denovo89_f0    denovo167_f0   denovo219_f0   denovo271_f0   denovo375_f0   denovo427_f0   denovo453_f0   denovo531_f0   denovo583_f0   denovo609_f0   denovo791_f0   denovo1051_f0  denovo1077_f0  denovo1155_f0  denovo1233_f0  denovo1363_f0  denovo1389_f0  denovo1415_f0  denovo1493_f0  denovo1519_f0  denovo1649_f0  denovo1675_f0  denovo1753_f0  denovo1805_f0  denovo1935_f0  denovo1961_f0  denovo1987_f0  denovo2013_f0  denovo2117_f0  denovo2143_f0  denovo2195_f0  denovo2247_f0  denovo2273_f0  denovo2299_f0  denovo2455_f0  denovo2481_f0  denovo2507_f0  denovo2533_f0  denovo2585_f0  denovo2611_f0  denovo2637_f0  denovo2663_f0  denovo2767_f0  denovo2845_f0  denovo2923_f0  denovo3001_f0  denovo3157_f0  denovo3261_f0  denovo3313_f0  denovo3339_f0  denovo3443_f0  denovo3495_f0  denovo3599_f0  denovo3625_f0  denovo3677_f0  denovo3729_f0  denovo3755_f0  denovo3781_f0  denovo3807_f0  denovo3833_f0  denovo3859_f0  denovo3963_f0  denovo3989_f0  denovo4067_f0  denovo4197_f0  denovo4327_f0  denovo4353_f0  denovo4379_f0  denovo4405_f0  denovo4561_f0  denovo4639_f0  denovo4743_f0  denovo4769_f0  denovo4795_f0  denovo4847_f0  denovo4899_f0  denovo4925_f0  denovo4951_f0  denovo4977_f0  denovo5003_f0  denovo5211_f0  denovo5237_f0  denovo5263_f0  denovo5445_f0  denovo5601_f0  denovo5653_f0  denovo5965_f0  denovo6017_f0  denovo6069_f0  denovo6095_f0  denovo6121_f0  denovo6199_f0  denovo6225_f0  denovo6251_f0  denovo6277_f0  denovo6303_f0  denovo6381_f0  denovo6511_f0  denovo6563_f0  denovo6589_f0  denovo6615_f0  denovo6667_f0  denovo6693_f0  denovo6719_f0  denovo6745_f0  denovo6771_f0  denovo6979_f0  denovo7005_f0  denovo7083_f0  denovo7135_f0  denovo7161_f0  denovo7265_f0  denovo7291_f0  denovo7317_f0  denovo7343_f0  denovo7369_f0  denovo7395_f0  denovo7499_f0  denovo7525_f0  denovo7551_f0  denovo7629_f0  denovo7681_f0  denovo7707_f0  denovo7837_f0  denovo7915_f0  denovo8045_f0  denovo8071_f0  denovo8227_f0  denovo8253_f0  denovo8279_f0  denovo8305_f0  denovo8383_f0  denovo8409_f0  denovo8435_f0  denovo8461_f0  denovo8565_f0  denovo8591_f0  denovo8643_f0  denovo8669_f0  denovo8695_f0  denovo8773_f0  denovo8851_f0  denovo8903_f0  denovo8929_f0  denovo8955_f0  denovo8981_f0  denovo9007_f0  denovo9137_f0  denovo9163_f0  denovo9189_f0  denovo9293_f0  denovo9319_f0  denovo9345_f0  denovo9371_f0  denovo9397_f0  denovo9449_f0  denovo9501_f0  denovo9709_f0  denovo9735_f0  denovo9787_f0  denovo9813_f0  denovo9839_f0  denovo9891_f0  denovo9969_f0  denovo10203_f0 denovo10229_f0 denovo10281_f0 denovo10307_f0 denovo10359_f0 denovo10411_f0 denovo10437_f0 denovo10463_f0 denovo10515_f0 denovo10541_f0 denovo10619_f0 denovo10671_f0 denovo10723_f0 denovo10749_f0 denovo10775_f0 denovo10827_f0 denovo10853_f0 denovo10931_f0 denovo11009_f0 denovo11035_f0 denovo11087_f0 denovo11165_f0 denovo11191_f0 denovo11295_f0 denovo11321_f0 denovo11347_f0 denovo11399_f0 denovo11425_f0 denovo11451_f0 denovo11477_f0 denovo11555_f0 denovo11711_f0 denovo11737_f0 denovo11919_f0 denovo12075_f0 
SRR850324                     denovo21_f0    denovo99_f0    denovo177_f0   denovo229_f0   denovo281_f0   denovo385_f0   denovo437_f0   denovo463_f0   denovo541_f0   denovo593_f0   denovo619_f0   denovo801_f0   denovo1061_f0  denovo1087_f0  denovo1165_f0  denovo1243_f0  denovo1373_f0  denovo1399_f0  denovo1425_f0  denovo1503_f0  denovo1529_f0  denovo1659_f0  denovo1685_f0  denovo1763_f0  denovo1815_f0  denovo1945_f0  denovo1971_f0  denovo1997_f0  denovo2023_f0  denovo2127_f0  denovo2153_f0  denovo2205_f0  denovo2257_f0  denovo2283_f0  denovo2309_f0  denovo2465_f0  denovo2491_f0  denovo2517_f0  denovo2543_f0  denovo2595_f0  denovo2621_f0  denovo2647_f0  denovo2673_f0  denovo2777_f0  denovo2855_f0  denovo2933_f0  denovo3011_f0  denovo3167_f0  denovo3271_f0  denovo3323_f0  denovo3349_f0  denovo3453_f0  denovo3505_f0  denovo3609_f0  denovo3635_f0  denovo3687_f0  denovo3739_f0  denovo3765_f0  denovo3791_f0  denovo3817_f0  denovo3843_f0  denovo3869_f0  denovo3973_f0  denovo3999_f0  denovo4077_f0  denovo4207_f0  denovo4337_f0  denovo4363_f0  denovo4389_f0  denovo4415_f0  denovo4571_f0  denovo4649_f0  denovo4753_f0  denovo4779_f0  denovo4805_f0  denovo4857_f0  denovo4909_f0  denovo4935_f0  denovo4961_f0  denovo4987_f0  denovo5013_f0  denovo5221_f0  denovo5247_f0  denovo5273_f0  denovo5455_f0  denovo5611_f0  denovo5663_f0  denovo5975_f0  denovo6027_f0  denovo6079_f0  denovo6105_f0  denovo6131_f0  denovo6209_f0  denovo6235_f0  denovo6261_f0  denovo6287_f0  denovo6313_f0  denovo6391_f0  denovo6521_f0  denovo6573_f0  denovo6599_f0  denovo6625_f0  denovo6677_f0  denovo6703_f0  denovo6729_f0  denovo6755_f0  denovo6781_f0  denovo6989_f0  denovo7015_f0  denovo7093_f0  denovo7145_f0  denovo7171_f0  denovo7275_f0  denovo7301_f0  denovo7327_f0  denovo7353_f0  denovo7379_f0  denovo7405_f0  denovo7509_f0  denovo7535_f0  denovo7561_f0  denovo7639_f0  denovo7691_f0  denovo7717_f0  denovo7847_f0  denovo7925_f0  denovo8055_f0  denovo8081_f0  denovo8237_f0  denovo8263_f0  denovo8289_f0  denovo8315_f0  denovo8393_f0  denovo8419_f0  denovo8445_f0  denovo8471_f0  denovo8575_f0  denovo8601_f0  denovo8653_f0  denovo8679_f0  denovo8705_f0  denovo8783_f0  denovo8861_f0  denovo8913_f0  denovo8939_f0  denovo8965_f0  denovo8991_f0  denovo9017_f0  denovo9147_f0  denovo9173_f0  denovo9199_f0  denovo9303_f0  denovo9329_f0  denovo9355_f0  denovo9381_f0  denovo9407_f0  denovo9459_f0  denovo9511_f0  denovo9719_f0  denovo9745_f0  denovo9797_f0  denovo9823_f0  denovo9849_f0  denovo9901_f0  denovo9979_f0  denovo10213_f0 denovo10239_f0 denovo10291_f0 denovo10317_f0 denovo10369_f0 denovo10421_f0 denovo10447_f0 denovo10473_f0 denovo10525_f0 denovo10551_f0 denovo10629_f0 denovo10681_f0 denovo10733_f0 denovo10759_f0 denovo10785_f0 denovo10837_f0 denovo10863_f0 denovo10941_f0 denovo11019_f0 denovo11045_f0 denovo11097_f0 denovo11175_f0 denovo11201_f0 denovo11305_f0 denovo11331_f0 denovo11357_f0 denovo11409_f0 denovo11435_f0 denovo11461_f0 denovo11487_f0 denovo11565_f0 denovo11721_f0 denovo11747_f0 denovo11929_f0 denovo12085_f0 
FG120055B                     denovo7_f0     denovo85_f0    denovo163_f0   denovo215_f0   denovo267_f0   denovo371_f0   denovo423_f0   denovo449_f0   denovo527_f0   denovo579_f0   denovo605_f0   denovo787_f0   denovo1047_f0  denovo1073_f0  denovo1151_f0  denovo1229_f0  denovo1359_f0  denovo1385_f0  denovo1411_f0  denovo1489_f0  denovo1515_f0  denovo1645_f0  denovo1671_f0  denovo1749_f0  denovo1801_f0  denovo1931_f0  denovo1957_f0  denovo1983_f0  denovo2009_f0  denovo2113_f0  denovo2139_f0  denovo2191_f0  denovo2243_f0  denovo2269_f0  denovo2295_f0  denovo2451_f0  denovo2477_f0  denovo2503_f0  denovo2529_f0  denovo2581_f0  denovo2607_f0  denovo2633_f0  denovo2659_f0  denovo2763_f0  denovo2841_f0  denovo2919_f0  denovo2997_f0  denovo3153_f0  denovo3257_f0  denovo3309_f0  denovo3335_f0  denovo3439_f0  denovo3491_f0  denovo3595_f0  denovo3621_f0  denovo3673_f0  denovo3725_f0  denovo3751_f0  denovo3777_f0  denovo3803_f0  denovo3829_f0  denovo3855_f0  denovo3959_f0  denovo3985_f0  denovo4063_f0  denovo4193_f0  denovo4323_f0  denovo4349_f0  denovo4375_f0  denovo4401_f0  denovo4557_f0  denovo4635_f0  denovo4739_f0  denovo4765_f0  denovo4791_f0  denovo4843_f0  denovo4895_f0  denovo4921_f0  denovo4947_f0  denovo4973_f0  denovo4999_f0  denovo5207_f0  denovo5233_f0  denovo5259_f0  denovo5441_f0  denovo5597_f0  denovo5649_f0  denovo5961_f0  denovo6013_f0  denovo6065_f0  denovo6091_f0  denovo6117_f0  denovo6195_f0  denovo6221_f0  denovo6247_f0  denovo6273_f0  denovo6299_f0  denovo6377_f0  denovo6507_f0  denovo6559_f0  denovo6585_f0  denovo6611_f0  denovo6663_f0  denovo6689_f0  denovo6715_f0  denovo6741_f0  denovo6767_f0  denovo6975_f0  denovo7001_f0  denovo7079_f0  denovo7131_f0  denovo7157_f0  denovo7261_f0  denovo7287_f0  denovo7313_f0  denovo7339_f0  denovo7365_f0  denovo7391_f0  denovo7495_f0  denovo7521_f0  denovo7547_f0  denovo7625_f0  denovo7677_f0  denovo7703_f0  denovo7833_f0  denovo7911_f0  denovo8041_f0  denovo8067_f0  denovo8223_f0  denovo8249_f0  denovo8275_f0  denovo8301_f0  denovo8379_f0  denovo8405_f0  denovo8431_f0  denovo8457_f0  denovo8561_f0  denovo8587_f0  denovo8639_f0  denovo8665_f0  denovo8691_f0  denovo8769_f0  denovo8847_f0  denovo8899_f0  denovo8925_f0  denovo8951_f0  denovo8977_f0  denovo9003_f0  denovo9133_f0  denovo9159_f0  denovo9185_f0  denovo9289_f0  denovo9315_f0  denovo9341_f0  denovo9367_f0  denovo9393_f0  denovo9445_f0  denovo9497_f0  denovo9705_f0  denovo9731_f0  denovo9783_f0  denovo9809_f0  denovo9835_f0  denovo9887_f0  denovo9965_f0  denovo10199_f0 denovo10225_f0 denovo10277_f0 denovo10303_f0 denovo10355_f0 denovo10407_f0 denovo10433_f0 denovo10459_f0 denovo10511_f0 denovo10537_f0 denovo10615_f0 denovo10667_f0 denovo10719_f0 denovo10745_f0 denovo10771_f0 denovo10823_f0 denovo10849_f0 denovo10927_f0 denovo11005_f0 denovo11031_f0 denovo11083_f0 denovo11161_f0 denovo11187_f0 denovo11291_f0 denovo11317_f0 denovo11343_f0 denovo11395_f0 denovo11421_f0 denovo11447_f0 denovo11473_f0 denovo11551_f0 denovo11707_f0 denovo11733_f0 denovo11915_f0 denovo12071_f0 
FG120122                      denovo12_f0    denovo90_f0    denovo168_f0   denovo220_f0   denovo272_f0   denovo376_f0   denovo428_f0   denovo454_f0   denovo532_f0   denovo584_f0   denovo610_f0   denovo792_f0   denovo1052_f0  denovo1078_f0  denovo1156_f0  denovo1234_f0  denovo1364_f0  denovo1390_f0  denovo1416_f0  denovo1494_f0  denovo1520_f0  denovo1650_f0  denovo1676_f0  denovo1754_f0  denovo1806_f0  denovo1936_f0  denovo1962_f0  denovo1988_f0  denovo2014_f0  denovo2118_f0  denovo2144_f0  denovo2196_f0  denovo2248_f0  denovo2274_f0  denovo2300_f0  denovo2456_f0  denovo2482_f0  denovo2508_f0  denovo2534_f0  denovo2586_f0  denovo2612_f0  denovo2638_f0  denovo2664_f0  denovo2768_f0  denovo2846_f0  denovo2924_f0  denovo3002_f0  denovo3158_f0  denovo3262_f0  denovo3314_f0  denovo3340_f0  denovo3444_f0  denovo3496_f0  denovo3600_f0  denovo3626_f0  denovo3678_f0  denovo3730_f0  denovo3756_f0  denovo3782_f0  denovo3808_f0  denovo3834_f0  denovo3860_f0  denovo3964_f0  denovo3990_f0  denovo4068_f0  denovo4198_f0  denovo4328_f0  denovo4354_f0  denovo4380_f0  denovo4406_f0  denovo4562_f0  denovo4640_f0  denovo4744_f0  denovo4770_f0  denovo4796_f0  denovo4848_f0  denovo4900_f0  denovo4926_f0  denovo4952_f0  denovo4978_f0  denovo5004_f0  denovo5212_f0  denovo5238_f0  denovo5264_f0  denovo5446_f0  denovo5602_f0  denovo5654_f0  denovo5966_f0  denovo6018_f0  denovo6070_f0  denovo6096_f0  denovo6122_f0  denovo6200_f0  denovo6226_f0  denovo6252_f0  denovo6278_f0  denovo6304_f0  denovo6382_f0  denovo6512_f0  denovo6564_f0  denovo6590_f0  denovo6616_f0  denovo6668_f0  denovo6694_f0  denovo6720_f0  denovo6746_f0  denovo6772_f0  denovo6980_f0  denovo7006_f0  denovo7084_f0  denovo7136_f0  denovo7162_f0  denovo7266_f0  denovo7292_f0  denovo7318_f0  denovo7344_f0  denovo7370_f0  denovo7396_f0  denovo7500_f0  denovo7526_f0  denovo7552_f0  denovo7630_f0  denovo7682_f0  denovo7708_f0  denovo7838_f0  denovo7916_f0  denovo8046_f0  denovo8072_f0  denovo8228_f0  denovo8254_f0  denovo8280_f0  denovo8306_f0  denovo8384_f0  denovo8410_f0  denovo8436_f0  denovo8462_f0  denovo8566_f0  denovo8592_f0  denovo8644_f0  denovo8670_f0  denovo8696_f0  denovo8774_f0  denovo8852_f0  denovo8904_f0  denovo8930_f0  denovo8956_f0  denovo8982_f0  denovo9008_f0  denovo9138_f0  denovo9164_f0  denovo9190_f0  denovo9294_f0  denovo9320_f0  denovo9346_f0  denovo9372_f0  denovo9398_f0  denovo9450_f0  denovo9502_f0  denovo9710_f0  denovo9736_f0  denovo9788_f0  denovo9814_f0  denovo9840_f0  denovo9892_f0  denovo9970_f0  denovo10204_f0 denovo10230_f0 denovo10282_f0 denovo10308_f0 denovo10360_f0 denovo10412_f0 denovo10438_f0 denovo10464_f0 denovo10516_f0 denovo10542_f0 denovo10620_f0 denovo10672_f0 denovo10724_f0 denovo10750_f0 denovo10776_f0 denovo10828_f0 denovo10854_f0 denovo10932_f0 denovo11010_f0 denovo11036_f0 denovo11088_f0 denovo11166_f0 denovo11192_f0 denovo11296_f0 denovo11322_f0 denovo11348_f0 denovo11400_f0 denovo11426_f0 denovo11452_f0 denovo11478_f0 denovo11556_f0 denovo11712_f0 denovo11738_f0 denovo11920_f0 denovo12076_f0 
FG120024                      denovo4_f0     denovo82_f0    denovo160_f0   denovo212_f0   denovo264_f0   denovo368_f0   denovo420_f0   denovo446_f0   denovo524_f0   denovo576_f0   denovo602_f0   denovo784_f0   denovo1044_f0  denovo1070_f0  denovo1148_f0  denovo1226_f0  denovo1356_f0  denovo1382_f0  denovo1408_f0  denovo1486_f0  denovo1512_f0  denovo1642_f0  denovo1668_f0  denovo1746_f0  denovo1798_f0  denovo1928_f0  denovo1954_f0  denovo1980_f0  denovo2006_f0  denovo2110_f0  denovo2136_f0  denovo2188_f0  denovo2240_f0  denovo2266_f0  denovo2292_f0  denovo2448_f0  denovo2474_f0  denovo2500_f0  denovo2526_f0  denovo2578_f0  denovo2604_f0  denovo2630_f0  denovo2656_f0  denovo2760_f0  denovo2838_f0  denovo2916_f0  denovo2994_f0  denovo3150_f0  denovo3254_f0  denovo3306_f0  denovo3332_f0  denovo3436_f0  denovo3488_f0  denovo3592_f0  denovo3618_f0  denovo3670_f0  denovo3722_f0  denovo3748_f0  denovo3774_f0  denovo3800_f0  denovo3826_f0  denovo3852_f0  denovo3956_f0  denovo3982_f0  denovo4060_f0  denovo4190_f0  denovo4320_f0  denovo4346_f0  denovo4372_f0  denovo4398_f0  denovo4554_f0  denovo4632_f0  denovo4736_f0  denovo4762_f0  denovo4788_f0  denovo4840_f0  denovo4892_f0  denovo4918_f0  denovo4944_f0  denovo4970_f0  denovo4996_f0  denovo5204_f0  denovo5230_f0  denovo5256_f0  denovo5438_f0  denovo5594_f0  denovo5646_f0  denovo5958_f0  denovo6010_f0  denovo6062_f0  denovo6088_f0  denovo6114_f0  denovo6192_f0  denovo6218_f0  denovo6244_f0  denovo6270_f0  denovo6296_f0  denovo6374_f0  denovo6504_f0  denovo6556_f0  denovo6582_f0  denovo6608_f0  denovo6660_f0  denovo6686_f0  denovo6712_f0  denovo6738_f0  denovo6764_f0  denovo6972_f0  denovo6998_f0  denovo7076_f0  denovo7128_f0  denovo7154_f0  denovo7258_f0  denovo7284_f0  denovo7310_f0  denovo7336_f0  denovo7362_f0  denovo7388_f0  denovo7492_f0  denovo7518_f0  denovo7544_f0  denovo7622_f0  denovo7674_f0  denovo7700_f0  denovo7830_f0  denovo7908_f0  denovo8038_f0  denovo8064_f0  denovo8220_f0  denovo8246_f0  denovo8272_f0  denovo8298_f0  denovo8376_f0  denovo8402_f0  denovo8428_f0  denovo8454_f0  denovo8558_f0  denovo8584_f0  denovo8636_f0  denovo8662_f0  denovo8688_f0  denovo8766_f0  denovo8844_f0  denovo8896_f0  denovo8922_f0  denovo8948_f0  denovo8974_f0  denovo9000_f0  denovo9130_f0  denovo9156_f0  denovo9182_f0  denovo9286_f0  denovo9312_f0  denovo9338_f0  denovo9364_f0  denovo9390_f0  denovo9442_f0  denovo9494_f0  denovo9702_f0  denovo9728_f0  denovo9780_f0  denovo9806_f0  denovo9832_f0  denovo9884_f0  denovo9962_f0  denovo10196_f0 denovo10222_f0 denovo10274_f0 denovo10300_f0 denovo10352_f0 denovo10404_f0 denovo10430_f0 denovo10456_f0 denovo10508_f0 denovo10534_f0 denovo10612_f0 denovo10664_f0 denovo10716_f0 denovo10742_f0 denovo10768_f0 denovo10820_f0 denovo10846_f0 denovo10924_f0 denovo11002_f0 denovo11028_f0 denovo11080_f0 denovo11158_f0 denovo11184_f0 denovo11288_f0 denovo11314_f0 denovo11340_f0 denovo11392_f0 denovo11418_f0 denovo11444_f0 denovo11470_f0 denovo11548_f0 denovo11704_f0 denovo11730_f0 denovo11912_f0 denovo12068_f0 
FG120022                      denovo3_f0     denovo81_f0    denovo159_f0   denovo211_f0   denovo263_f0   denovo367_f0   denovo419_f0   denovo445_f0   denovo523_f0   denovo575_f0   denovo601_f0   denovo783_f0   denovo1043_f0  denovo1069_f0  denovo1147_f0  denovo1225_f0  denovo1355_f0  denovo1381_f0  denovo1407_f0  denovo1485_f0  denovo1511_f0  denovo1641_f0  denovo1667_f0  denovo1745_f0  denovo1797_f0  denovo1927_f0  denovo1953_f0  denovo1979_f0  denovo2005_f0  denovo2109_f0  denovo2135_f0  denovo2187_f0  denovo2239_f0  denovo2265_f0  denovo2291_f0  denovo2447_f0  denovo2473_f0  denovo2499_f0  denovo2525_f0  denovo2577_f0  denovo2603_f0  denovo2629_f0  denovo2655_f0  denovo2759_f0  denovo2837_f0  denovo2915_f0  denovo2993_f0  denovo3149_f0  denovo3253_f0  denovo3305_f0  denovo3331_f0  denovo3435_f0  denovo3487_f0  denovo3591_f0  denovo3617_f0  denovo3669_f0  denovo3721_f0  denovo3747_f0  denovo3773_f0  denovo3799_f0  denovo3825_f0  denovo3851_f0  denovo3955_f0  denovo3981_f0  denovo4059_f0  denovo4189_f0  denovo4319_f0  denovo4345_f0  denovo4371_f0  denovo4397_f0  denovo4553_f0  denovo4631_f0  denovo4735_f0  denovo4761_f0  denovo4787_f0  denovo4839_f0  denovo4891_f0  denovo4917_f0  denovo4943_f0  denovo4969_f0  denovo4995_f0  denovo5203_f0  denovo5229_f0  denovo5255_f0  denovo5437_f0  denovo5593_f0  denovo5645_f0  denovo5957_f0  denovo6009_f0  denovo6061_f0  denovo6087_f0  denovo6113_f0  denovo6191_f0  denovo6217_f0  denovo6243_f0  denovo6269_f0  denovo6295_f0  denovo6373_f0  denovo6503_f0  denovo6555_f0  denovo6581_f0  denovo6607_f0  denovo6659_f0  denovo6685_f0  denovo6711_f0  denovo6737_f0  denovo6763_f0  denovo6971_f0  denovo6997_f0  denovo7075_f0  denovo7127_f0  denovo7153_f0  denovo7257_f0  denovo7283_f0  denovo7309_f0  denovo7335_f0  denovo7361_f0  denovo7387_f0  denovo7491_f0  denovo7517_f0  denovo7543_f0  denovo7621_f0  denovo7673_f0  denovo7699_f0  denovo7829_f0  denovo7907_f0  denovo8037_f0  denovo8063_f0  denovo8219_f0  denovo8245_f0  denovo8271_f0  denovo8297_f0  denovo8375_f0  denovo8401_f0  denovo8427_f0  denovo8453_f0  denovo8557_f0  denovo8583_f0  denovo8635_f0  denovo8661_f0  denovo8687_f0  denovo8765_f0  denovo8843_f0  denovo8895_f0  denovo8921_f0  denovo8947_f0  denovo8973_f0  denovo8999_f0  denovo9129_f0  denovo9155_f0  denovo9181_f0  denovo9285_f0  denovo9311_f0  denovo9337_f0  denovo9363_f0  denovo9389_f0  denovo9441_f0  denovo9493_f0  denovo9701_f0  denovo9727_f0  denovo9779_f0  denovo9805_f0  denovo9831_f0  denovo9883_f0  denovo9961_f0  denovo10195_f0 denovo10221_f0 denovo10273_f0 denovo10299_f0 denovo10351_f0 denovo10403_f0 denovo10429_f0 denovo10455_f0 denovo10507_f0 denovo10533_f0 denovo10611_f0 denovo10663_f0 denovo10715_f0 denovo10741_f0 denovo10767_f0 denovo10819_f0 denovo10845_f0 denovo10923_f0 denovo11001_f0 denovo11027_f0 denovo11079_f0 denovo11157_f0 denovo11183_f0 denovo11287_f0 denovo11313_f0 denovo11339_f0 denovo11391_f0 denovo11417_f0 denovo11443_f0 denovo11469_f0 denovo11547_f0 denovo11703_f0 denovo11729_f0 denovo11911_f0 denovo12067_f0 
GNV139000                     denovo16_f0    denovo94_f0    denovo172_f0   denovo224_f0   denovo276_f0   denovo380_f0   denovo432_f0   denovo458_f0   denovo536_f0   denovo588_f0   denovo614_f0   denovo796_f0   denovo1056_f0  denovo1082_f0  denovo1160_f0  denovo1238_f0  denovo1368_f0  denovo1394_f0  denovo1420_f0  denovo1498_f0  denovo1524_f0  denovo1654_f0  denovo1680_f0  denovo1758_f0  denovo1810_f0  denovo1940_f0  denovo1966_f0  denovo1992_f0  denovo2018_f0  denovo2122_f0  denovo2148_f0  denovo2200_f0  denovo2252_f0  denovo2278_f0  denovo2304_f0  denovo2460_f0  denovo2486_f0  denovo2512_f0  denovo2538_f0  denovo2590_f0  denovo2616_f0  denovo2642_f0  denovo2668_f0  denovo2772_f0  denovo2850_f0  denovo2928_f0  denovo3006_f0  denovo3162_f0  denovo3266_f0  denovo3318_f0  denovo3344_f0  denovo3448_f0  denovo3500_f0  denovo3604_f0  denovo3630_f0  denovo3682_f0  denovo3734_f0  denovo3760_f0  denovo3786_f0  denovo3812_f0  denovo3838_f0  denovo3864_f0  denovo3968_f0  denovo3994_f0  denovo4072_f0  denovo4202_f0  denovo4332_f0  denovo4358_f0  denovo4384_f0  denovo4410_f0  denovo4566_f0  denovo4644_f0  denovo4748_f0  denovo4774_f0  denovo4800_f0  denovo4852_f0  denovo4904_f0  denovo4930_f0  denovo4956_f0  denovo4982_f0  denovo5008_f0  denovo5216_f0  denovo5242_f0  denovo5268_f0  denovo5450_f0  denovo5606_f0  denovo5658_f0  denovo5970_f0  denovo6022_f0  denovo6074_f0  denovo6100_f0  denovo6126_f0  denovo6204_f0  denovo6230_f0  denovo6256_f0  denovo6282_f0  denovo6308_f0  denovo6386_f0  denovo6516_f0  denovo6568_f0  denovo6594_f0  denovo6620_f0  denovo6672_f0  denovo6698_f0  denovo6724_f0  denovo6750_f0  denovo6776_f0  denovo6984_f0  denovo7010_f0  denovo7088_f0  denovo7140_f0  denovo7166_f0  denovo7270_f0  denovo7296_f0  denovo7322_f0  denovo7348_f0  denovo7374_f0  denovo7400_f0  denovo7504_f0  denovo7530_f0  denovo7556_f0  denovo7634_f0  denovo7686_f0  denovo7712_f0  denovo7842_f0  denovo7920_f0  denovo8050_f0  denovo8076_f0  denovo8232_f0  denovo8258_f0  denovo8284_f0  denovo8310_f0  denovo8388_f0  denovo8414_f0  denovo8440_f0  denovo8466_f0  denovo8570_f0  denovo8596_f0  denovo8648_f0  denovo8674_f0  denovo8700_f0  denovo8778_f0  denovo8856_f0  denovo8908_f0  denovo8934_f0  denovo8960_f0  denovo8986_f0  denovo9012_f0  denovo9142_f0  denovo9168_f0  denovo9194_f0  denovo9298_f0  denovo9324_f0  denovo9350_f0  denovo9376_f0  denovo9402_f0  denovo9454_f0  denovo9506_f0  denovo9714_f0  denovo9740_f0  denovo9792_f0  denovo9818_f0  denovo9844_f0  denovo9896_f0  denovo9974_f0  denovo10208_f0 denovo10234_f0 denovo10286_f0 denovo10312_f0 denovo10364_f0 denovo10416_f0 denovo10442_f0 denovo10468_f0 denovo10520_f0 denovo10546_f0 denovo10624_f0 denovo10676_f0 denovo10728_f0 denovo10754_f0 denovo10780_f0 denovo10832_f0 denovo10858_f0 denovo10936_f0 denovo11014_f0 denovo11040_f0 denovo11092_f0 denovo11170_f0 denovo11196_f0 denovo11300_f0 denovo11326_f0 denovo11352_f0 denovo11404_f0 denovo11430_f0 denovo11456_f0 denovo11482_f0 denovo11560_f0 denovo11716_f0 denovo11742_f0 denovo11924_f0 denovo12080_f0 
Msexta                        denovo17_f0    denovo95_f0    denovo173_f0   denovo225_f0   denovo277_f0   denovo381_f0   denovo433_f0   denovo459_f0   denovo537_f0   denovo589_f0   denovo615_f0   denovo797_f0   denovo1057_f0  denovo1083_f0  denovo1161_f0  denovo1239_f0  denovo1369_f0  denovo1395_f0  denovo1421_f0  denovo1499_f0  denovo1525_f0  denovo1655_f0  denovo1681_f0  denovo1759_f0  denovo1811_f0  denovo1941_f0  denovo1967_f0  denovo1993_f0  denovo2019_f0  denovo2123_f0  denovo2149_f0  denovo2201_f0  denovo2253_f0  denovo2279_f0  denovo2305_f0  denovo2461_f0  denovo2487_f0  denovo2513_f0  denovo2539_f0  denovo2591_f0  denovo2617_f0  denovo2643_f0  denovo2669_f0  denovo2773_f0  denovo2851_f0  denovo2929_f0  denovo3007_f0  denovo3163_f0  denovo3267_f0  denovo3319_f0  denovo3345_f0  denovo3449_f0  denovo3501_f0  denovo3605_f0  denovo3631_f0  denovo3683_f0  denovo3735_f0  denovo3761_f0  denovo3787_f0  denovo3813_f0  denovo3839_f0  denovo3865_f0  denovo3969_f0  denovo3995_f0  denovo4073_f0  denovo4203_f0  denovo4333_f0  denovo4359_f0  denovo4385_f0  denovo4411_f0  denovo4567_f0  denovo4645_f0  denovo4749_f0  denovo4775_f0  denovo4801_f0  denovo4853_f0  denovo4905_f0  denovo4931_f0  denovo4957_f0  denovo4983_f0  denovo5009_f0  denovo5217_f0  denovo5243_f0  denovo5269_f0  denovo5451_f0  denovo5607_f0  denovo5659_f0  denovo5971_f0  denovo6023_f0  denovo6075_f0  denovo6101_f0  denovo6127_f0  denovo6205_f0  denovo6231_f0  denovo6257_f0  denovo6283_f0  denovo6309_f0  denovo6387_f0  denovo6517_f0  denovo6569_f0  denovo6595_f0  denovo6621_f0  denovo6673_f0  denovo6699_f0  denovo6725_f0  denovo6751_f0  denovo6777_f0  denovo6985_f0  denovo7011_f0  denovo7089_f0  denovo7141_f0  denovo7167_f0  denovo7271_f0  denovo7297_f0  denovo7323_f0  denovo7349_f0  denovo7375_f0  denovo7401_f0  denovo7505_f0  denovo7531_f0  denovo7557_f0  denovo7635_f0  denovo7687_f0  denovo7713_f0  denovo7843_f0  denovo7921_f0  denovo8051_f0  denovo8077_f0  denovo8233_f0  denovo8259_f0  denovo8285_f0  denovo8311_f0  denovo8389_f0  denovo8415_f0  denovo8441_f0  denovo8467_f0  denovo8571_f0  denovo8597_f0  denovo8649_f0  denovo8675_f0  denovo8701_f0  denovo8779_f0  denovo8857_f0  denovo8909_f0  denovo8935_f0  denovo8961_f0  denovo8987_f0  denovo9013_f0  denovo9143_f0  denovo9169_f0  denovo9195_f0  denovo9299_f0  denovo9325_f0  denovo9351_f0  denovo9377_f0  denovo9403_f0  denovo9455_f0  denovo9507_f0  denovo9715_f0  denovo9741_f0  denovo9793_f0  denovo9819_f0  denovo9845_f0  denovo9897_f0  denovo9975_f0  denovo10209_f0 denovo10235_f0 denovo10287_f0 denovo10313_f0 denovo10365_f0 denovo10417_f0 denovo10443_f0 denovo10469_f0 denovo10521_f0 denovo10547_f0 denovo10625_f0 denovo10677_f0 denovo10729_f0 denovo10755_f0 denovo10781_f0 denovo10833_f0 denovo10859_f0 denovo10937_f0 denovo11015_f0 denovo11041_f0 denovo11093_f0 denovo11171_f0 denovo11197_f0 denovo11301_f0 denovo11327_f0 denovo11353_f0 denovo11405_f0 denovo11431_f0 denovo11457_f0 denovo11483_f0 denovo11561_f0 denovo11717_f0 denovo11743_f0 denovo11925_f0 denovo12081_f0 
acti2                         denovo25_f0    denovo103_f0   denovo181_f0   denovo233_f0   denovo285_f0   denovo389_f0   denovo441_f0   denovo467_f0   denovo545_f0   denovo597_f0   denovo623_f0   denovo805_f0   denovo1065_f0  denovo1091_f0  denovo1169_f0  denovo1247_f0  denovo1377_f0  denovo1403_f0  denovo1429_f0  denovo1507_f0  denovo1533_f0  denovo1663_f0  denovo1689_f0  denovo1767_f0  denovo1819_f0  denovo1949_f0  denovo1975_f0  denovo2001_f0  denovo2027_f0  denovo2131_f0  denovo2157_f0  denovo2209_f0  denovo2261_f0  denovo2287_f0  denovo2313_f0  denovo2469_f0  denovo2495_f0  denovo2521_f0  denovo2547_f0  denovo2599_f0  denovo2625_f0  denovo2651_f0  denovo2677_f0  denovo2781_f0  denovo2859_f0  denovo2937_f0  denovo3015_f0  denovo3171_f0  denovo3275_f0  denovo3327_f0  denovo3353_f0  denovo3457_f0  denovo3509_f0  denovo3613_f0  denovo3639_f0  denovo3691_f0  denovo3743_f0  denovo3769_f0  denovo3795_f0  denovo3821_f0  denovo3847_f0  denovo3873_f0  denovo3977_f0  denovo4003_f0  denovo4081_f0  denovo4211_f0  denovo4341_f0  denovo4367_f0  denovo4393_f0  denovo4419_f0  denovo4575_f0  denovo4653_f0  denovo4757_f0  denovo4783_f0  denovo4809_f0  denovo4861_f0  denovo4913_f0  denovo4939_f0  denovo4965_f0  denovo4991_f0  denovo5017_f0  denovo5225_f0  denovo5251_f0  denovo5277_f0  denovo5459_f0  denovo5615_f0  denovo5667_f0  denovo5979_f0  denovo6031_f0  denovo6083_f0  denovo6109_f0  denovo6135_f0  denovo6213_f0  denovo6239_f0  denovo6265_f0  denovo6291_f0  denovo6317_f0  denovo6395_f0  denovo6525_f0  denovo6577_f0  denovo6603_f0  denovo6629_f0  denovo6681_f0  denovo6707_f0  denovo6733_f0  denovo6759_f0  denovo6785_f0  denovo6993_f0  denovo7019_f0  denovo7097_f0  denovo7149_f0  denovo7175_f0  denovo7279_f0  denovo7305_f0  denovo7331_f0  denovo7357_f0  denovo7383_f0  denovo7409_f0  denovo7513_f0  denovo7539_f0  denovo7565_f0  denovo7643_f0  denovo7695_f0  denovo7721_f0  denovo7851_f0  denovo7929_f0  denovo8059_f0  denovo8085_f0  denovo8241_f0  denovo8267_f0  denovo8293_f0  denovo8319_f0  denovo8397_f0  denovo8423_f0  denovo8449_f0  denovo8475_f0  denovo8579_f0  denovo8605_f0  denovo8657_f0  denovo8683_f0  denovo8709_f0  denovo8787_f0  denovo8865_f0  denovo8917_f0  denovo8943_f0  denovo8969_f0  denovo8995_f0  denovo9021_f0  denovo9151_f0  denovo9177_f0  denovo9203_f0  denovo9307_f0  denovo9333_f0  denovo9359_f0  denovo9385_f0  denovo9411_f0  denovo9463_f0  denovo9515_f0  denovo9723_f0  denovo9749_f0  denovo9801_f0  denovo9827_f0  denovo9853_f0  denovo9905_f0  denovo9983_f0  denovo10217_f0 denovo10243_f0 denovo10295_f0 denovo10321_f0 denovo10373_f0 denovo10425_f0 denovo10451_f0 denovo10477_f0 denovo10529_f0 denovo10555_f0 denovo10633_f0 denovo10685_f0 denovo10737_f0 denovo10763_f0 denovo10789_f0 denovo10841_f0 denovo10867_f0 denovo10945_f0 denovo11023_f0 denovo11049_f0 denovo11101_f0 denovo11179_f0 denovo11205_f0 denovo11309_f0 denovo11335_f0 denovo11361_f0 denovo11413_f0 denovo11439_f0 denovo11465_f0 denovo11491_f0 denovo11569_f0 denovo11725_f0 denovo11751_f0 denovo11933_f0 denovo12089_f0 
FG120071B                     denovo9_f0     denovo87_f0    denovo165_f0   denovo217_f0   denovo269_f0   denovo373_f0   denovo425_f0   denovo451_f0   denovo529_f0   denovo581_f0   denovo607_f0   denovo789_f0   denovo1049_f0  denovo1075_f0  denovo1153_f0  denovo1231_f0  denovo1361_f0  denovo1387_f0  denovo1413_f0  denovo1491_f0  denovo1517_f0  denovo1647_f0  denovo1673_f0  denovo1751_f0  denovo1803_f0  denovo1933_f0  denovo1959_f0  denovo1985_f0  denovo2011_f0  denovo2115_f0  denovo2141_f0  denovo2193_f0  denovo2245_f0  denovo2271_f0  denovo2297_f0  denovo2453_f0  denovo2479_f0  denovo2505_f0  denovo2531_f0  denovo2583_f0  denovo2609_f0  denovo2635_f0  denovo2661_f0  denovo2765_f0  denovo2843_f0  denovo2921_f0  denovo2999_f0  denovo3155_f0  denovo3259_f0  denovo3311_f0  denovo3337_f0  denovo3441_f0  denovo3493_f0  denovo3597_f0  denovo3623_f0  denovo3675_f0  denovo3727_f0  denovo3753_f0  denovo3779_f0  denovo3805_f0  denovo3831_f0  denovo3857_f0  denovo3961_f0  denovo3987_f0  denovo4065_f0  denovo4195_f0  denovo4325_f0  denovo4351_f0  denovo4377_f0  denovo4403_f0  denovo4559_f0  denovo4637_f0  denovo4741_f0  denovo4767_f0  denovo4793_f0  denovo4845_f0  denovo4897_f0  denovo4923_f0  denovo4949_f0  denovo4975_f0  denovo5001_f0  denovo5209_f0  denovo5235_f0  denovo5261_f0  denovo5443_f0  denovo5599_f0  denovo5651_f0  denovo5963_f0  denovo6015_f0  denovo6067_f0  denovo6093_f0  denovo6119_f0  denovo6197_f0  denovo6223_f0  denovo6249_f0  denovo6275_f0  denovo6301_f0  denovo6379_f0  denovo6509_f0  denovo6561_f0  denovo6587_f0  denovo6613_f0  denovo6665_f0  denovo6691_f0  denovo6717_f0  denovo6743_f0  denovo6769_f0  denovo6977_f0  denovo7003_f0  denovo7081_f0  denovo7133_f0  denovo7159_f0  denovo7263_f0  denovo7289_f0  denovo7315_f0  denovo7341_f0  denovo7367_f0  denovo7393_f0  denovo7497_f0  denovo7523_f0  denovo7549_f0  denovo7627_f0  denovo7679_f0  denovo7705_f0  denovo7835_f0  denovo7913_f0  denovo8043_f0  denovo8069_f0  denovo8225_f0  denovo8251_f0  denovo8277_f0  denovo8303_f0  denovo8381_f0  denovo8407_f0  denovo8433_f0  denovo8459_f0  denovo8563_f0  denovo8589_f0  denovo8641_f0  denovo8667_f0  denovo8693_f0  denovo8771_f0  denovo8849_f0  denovo8901_f0  denovo8927_f0  denovo8953_f0  denovo8979_f0  denovo9005_f0  denovo9135_f0  denovo9161_f0  denovo9187_f0  denovo9291_f0  denovo9317_f0  denovo9343_f0  denovo9369_f0  denovo9395_f0  denovo9447_f0  denovo9499_f0  denovo9707_f0  denovo9733_f0  denovo9785_f0  denovo9811_f0  denovo9837_f0  denovo9889_f0  denovo9967_f0  denovo10201_f0 denovo10227_f0 denovo10279_f0 denovo10305_f0 denovo10357_f0 denovo10409_f0 denovo10435_f0 denovo10461_f0 denovo10513_f0 denovo10539_f0 denovo10617_f0 denovo10669_f0 denovo10721_f0 denovo10747_f0 denovo10773_f0 denovo10825_f0 denovo10851_f0 denovo10929_f0 denovo11007_f0 denovo11033_f0 denovo11085_f0 denovo11163_f0 denovo11189_f0 denovo11293_f0 denovo11319_f0 denovo11345_f0 denovo11397_f0 denovo11423_f0 denovo11449_f0 denovo11475_f0 denovo11553_f0 denovo11709_f0 denovo11735_f0 denovo11917_f0 denovo12073_f0 
GNV120027                     denovo13_f0    denovo91_f0    denovo169_f0   denovo221_f0   denovo273_f0   denovo377_f0   denovo429_f0   denovo455_f0   denovo533_f0   denovo585_f0   denovo611_f0   denovo793_f0   denovo1053_f0  denovo1079_f0  denovo1157_f0  denovo1235_f0  denovo1365_f0  denovo1391_f0  denovo1417_f0  denovo1495_f0  denovo1521_f0  denovo1651_f0  denovo1677_f0  denovo1755_f0  denovo1807_f0  denovo1937_f0  denovo1963_f0  denovo1989_f0  denovo2015_f0  denovo2119_f0  denovo2145_f0  denovo2197_f0  denovo2249_f0  denovo2275_f0  denovo2301_f0  denovo2457_f0  denovo2483_f0  denovo2509_f0  denovo2535_f0  denovo2587_f0  denovo2613_f0  denovo2639_f0  denovo2665_f0  denovo2769_f0  denovo2847_f0  denovo2925_f0  denovo3003_f0  denovo3159_f0  denovo3263_f0  denovo3315_f0  denovo3341_f0  denovo3445_f0  denovo3497_f0  denovo3601_f0  denovo3627_f0  denovo3679_f0  denovo3731_f0  denovo3757_f0  denovo3783_f0  denovo3809_f0  denovo3835_f0  denovo3861_f0  denovo3965_f0  denovo3991_f0  denovo4069_f0  denovo4199_f0  denovo4329_f0  denovo4355_f0  denovo4381_f0  denovo4407_f0  denovo4563_f0  denovo4641_f0  denovo4745_f0  denovo4771_f0  denovo4797_f0  denovo4849_f0  denovo4901_f0  denovo4927_f0  denovo4953_f0  denovo4979_f0  denovo5005_f0  denovo5213_f0  denovo5239_f0  denovo5265_f0  denovo5447_f0  denovo5603_f0  denovo5655_f0  denovo5967_f0  denovo6019_f0  denovo6071_f0  denovo6097_f0  denovo6123_f0  denovo6201_f0  denovo6227_f0  denovo6253_f0  denovo6279_f0  denovo6305_f0  denovo6383_f0  denovo6513_f0  denovo6565_f0  denovo6591_f0  denovo6617_f0  denovo6669_f0  denovo6695_f0  denovo6721_f0  denovo6747_f0  denovo6773_f0  denovo6981_f0  denovo7007_f0  denovo7085_f0  denovo7137_f0  denovo7163_f0  denovo7267_f0  denovo7293_f0  denovo7319_f0  denovo7345_f0  denovo7371_f0  denovo7397_f0  denovo7501_f0  denovo7527_f0  denovo7553_f0  denovo7631_f0  denovo7683_f0  denovo7709_f0  denovo7839_f0  denovo7917_f0  denovo8047_f0  denovo8073_f0  denovo8229_f0  denovo8255_f0  denovo8281_f0  denovo8307_f0  denovo8385_f0  denovo8411_f0  denovo8437_f0  denovo8463_f0  denovo8567_f0  denovo8593_f0  denovo8645_f0  denovo8671_f0  denovo8697_f0  denovo8775_f0  denovo8853_f0  denovo8905_f0  denovo8931_f0  denovo8957_f0  denovo8983_f0  denovo9009_f0  denovo9139_f0  denovo9165_f0  denovo9191_f0  denovo9295_f0  denovo9321_f0  denovo9347_f0  denovo9373_f0  denovo9399_f0  denovo9451_f0  denovo9503_f0  denovo9711_f0  denovo9737_f0  denovo9789_f0  denovo9815_f0  denovo9841_f0  denovo9893_f0  denovo9971_f0  denovo10205_f0 denovo10231_f0 denovo10283_f0 denovo10309_f0 denovo10361_f0 denovo10413_f0 denovo10439_f0 denovo10465_f0 denovo10517_f0 denovo10543_f0 denovo10621_f0 denovo10673_f0 denovo10725_f0 denovo10751_f0 denovo10777_f0 denovo10829_f0 denovo10855_f0 denovo10933_f0 denovo11011_f0 denovo11037_f0 denovo11089_f0 denovo11167_f0 denovo11193_f0 denovo11297_f0 denovo11323_f0 denovo11349_f0 denovo11401_f0 denovo11427_f0 denovo11453_f0 denovo11479_f0 denovo11557_f0 denovo11713_f0 denovo11739_f0 denovo11921_f0 denovo12077_f0 
Bmoricds                      denovo0_f0     denovo78_f0    denovo156_f0   denovo208_f0   denovo260_f0   denovo364_f0   denovo416_f0   denovo442_f0   denovo520_f0   denovo572_f0   denovo598_f0   denovo780_f0   denovo1040_f0  denovo1066_f0  denovo1144_f0  denovo1222_f0  denovo1352_f0  denovo1378_f0  denovo1404_f0  denovo1482_f0  denovo1508_f0  denovo1638_f0  denovo1664_f0  denovo1742_f0  denovo1794_f0  denovo1924_f0  denovo1950_f0  denovo1976_f0  denovo2002_f0  denovo2106_f0  denovo2132_f0  denovo2184_f0  denovo2236_f0  denovo2262_f0  denovo2288_f0  denovo2444_f0  denovo2470_f0  denovo2496_f0  denovo2522_f0  denovo2574_f0  denovo2600_f0  denovo2626_f0  denovo2652_f0  denovo2756_f0  denovo2834_f0  denovo2912_f0  denovo2990_f0  denovo3146_f0  denovo3250_f0  denovo3302_f0  denovo3328_f0  denovo3432_f0  denovo3484_f0  denovo3588_f0  denovo3614_f0  denovo3666_f0  denovo3718_f0  denovo3744_f0  denovo3770_f0  denovo3796_f0  denovo3822_f0  denovo3848_f0  denovo3952_f0  denovo3978_f0  denovo4056_f0  denovo4186_f0  denovo4316_f0  denovo4342_f0  denovo4368_f0  denovo4394_f0  denovo4550_f0  denovo4628_f0  denovo4732_f0  denovo4758_f0  denovo4784_f0  denovo4836_f0  denovo4888_f0  denovo4914_f0  denovo4940_f0  denovo4966_f0  denovo4992_f0  denovo5200_f0  denovo5226_f0  denovo5252_f0  denovo5434_f0  denovo5590_f0  denovo5642_f0  denovo5954_f0  denovo6006_f0  denovo6058_f0  denovo6084_f0  denovo6110_f0  denovo6188_f0  denovo6214_f0  denovo6240_f0  denovo6266_f0  denovo6292_f0  denovo6370_f0  denovo6500_f0  denovo6552_f0  denovo6578_f0  denovo6604_f0  denovo6656_f0  denovo6682_f0  denovo6708_f0  denovo6734_f0  denovo6760_f0  denovo6968_f0  denovo6994_f0  denovo7072_f0  denovo7124_f0  denovo7150_f0  denovo7254_f0  denovo7280_f0  denovo7306_f0  denovo7332_f0  denovo7358_f0  denovo7384_f0  denovo7488_f0  denovo7514_f0  denovo7540_f0  denovo7618_f0  denovo7670_f0  denovo7696_f0  denovo7826_f0  denovo7904_f0  denovo8034_f0  denovo8060_f0  denovo8216_f0  denovo8242_f0  denovo8268_f0  denovo8294_f0  denovo8372_f0  denovo8398_f0  denovo8424_f0  denovo8450_f0  denovo8554_f0  denovo8580_f0  denovo8632_f0  denovo8658_f0  denovo8684_f0  denovo8762_f0  denovo8840_f0  denovo8892_f0  denovo8918_f0  denovo8944_f0  denovo8970_f0  denovo8996_f0  denovo9126_f0  denovo9152_f0  denovo9178_f0  denovo9282_f0  denovo9308_f0  denovo9334_f0  denovo9360_f0  denovo9386_f0  denovo9438_f0  denovo9490_f0  denovo9698_f0  denovo9724_f0  denovo9776_f0  denovo9802_f0  denovo9828_f0  denovo9880_f0  denovo9958_f0  denovo10192_f0 denovo10218_f0 denovo10270_f0 denovo10296_f0 denovo10348_f0 denovo10400_f0 denovo10426_f0 denovo10452_f0 denovo10504_f0 denovo10530_f0 denovo10608_f0 denovo10660_f0 denovo10712_f0 denovo10738_f0 denovo10764_f0 denovo10816_f0 denovo10842_f0 denovo10920_f0 denovo10998_f0 denovo11024_f0 denovo11076_f0 denovo11154_f0 denovo11180_f0 denovo11284_f0 denovo11310_f0 denovo11336_f0 denovo11388_f0 denovo11414_f0 denovo11440_f0 denovo11466_f0 denovo11544_f0 denovo11700_f0 denovo11726_f0 denovo11908_f0 denovo12064_f0 
Pcit2                         denovo19_f0    denovo97_f0    denovo175_f0   denovo227_f0   denovo279_f0   denovo383_f0   denovo435_f0   denovo461_f0   denovo539_f0   denovo591_f0   denovo617_f0   denovo799_f0   denovo1059_f0  denovo1085_f0  denovo1163_f0  denovo1241_f0  denovo1371_f0  denovo1397_f0  denovo1423_f0  denovo1501_f0  denovo1527_f0  denovo1657_f0  denovo1683_f0  denovo1761_f0  denovo1813_f0  denovo1943_f0  denovo1969_f0  denovo1995_f0  denovo2021_f0  denovo2125_f0  denovo2151_f0  denovo2203_f0  denovo2255_f0  denovo2281_f0  denovo2307_f0  denovo2463_f0  denovo2489_f0  denovo2515_f0  denovo2541_f0  denovo2593_f0  denovo2619_f0  denovo2645_f0  denovo2671_f0  denovo2775_f0  denovo2853_f0  denovo2931_f0  denovo3009_f0  denovo3165_f0  denovo3269_f0  denovo3321_f0  denovo3347_f0  denovo3451_f0  denovo3503_f0  denovo3607_f0  denovo3633_f0  denovo3685_f0  denovo3737_f0  denovo3763_f0  denovo3789_f0  denovo3815_f0  denovo3841_f0  denovo3867_f0  denovo3971_f0  denovo3997_f0  denovo4075_f0  denovo4205_f0  denovo4335_f0  denovo4361_f0  denovo4387_f0  denovo4413_f0  denovo4569_f0  denovo4647_f0  denovo4751_f0  denovo4777_f0  denovo4803_f0  denovo4855_f0  denovo4907_f0  denovo4933_f0  denovo4959_f0  denovo4985_f0  denovo5011_f0  denovo5219_f0  denovo5245_f0  denovo5271_f0  denovo5453_f0  denovo5609_f0  denovo5661_f0  denovo5973_f0  denovo6025_f0  denovo6077_f0  denovo6103_f0  denovo6129_f0  denovo6207_f0  denovo6233_f0  denovo6259_f0  denovo6285_f0  denovo6311_f0  denovo6389_f0  denovo6519_f0  denovo6571_f0  denovo6597_f0  denovo6623_f0  denovo6675_f0  denovo6701_f0  denovo6727_f0  denovo6753_f0  denovo6779_f0  denovo6987_f0  denovo7013_f0  denovo7091_f0  denovo7143_f0  denovo7169_f0  denovo7273_f0  denovo7299_f0  denovo7325_f0  denovo7351_f0  denovo7377_f0  denovo7403_f0  denovo7507_f0  denovo7533_f0  denovo7559_f0  denovo7637_f0  denovo7689_f0  denovo7715_f0  denovo7845_f0  denovo7923_f0  denovo8053_f0  denovo8079_f0  denovo8235_f0  denovo8261_f0  denovo8287_f0  denovo8313_f0  denovo8391_f0  denovo8417_f0  denovo8443_f0  denovo8469_f0  denovo8573_f0  denovo8599_f0  denovo8651_f0  denovo8677_f0  denovo8703_f0  denovo8781_f0  denovo8859_f0  denovo8911_f0  denovo8937_f0  denovo8963_f0  denovo8989_f0  denovo9015_f0  denovo9145_f0  denovo9171_f0  denovo9197_f0  denovo9301_f0  denovo9327_f0  denovo9353_f0  denovo9379_f0  denovo9405_f0  denovo9457_f0  denovo9509_f0  denovo9717_f0  denovo9743_f0  denovo9795_f0  denovo9821_f0  denovo9847_f0  denovo9899_f0  denovo9977_f0  denovo10211_f0 denovo10237_f0 denovo10289_f0 denovo10315_f0 denovo10367_f0 denovo10419_f0 denovo10445_f0 denovo10471_f0 denovo10523_f0 denovo10549_f0 denovo10627_f0 denovo10679_f0 denovo10731_f0 denovo10757_f0 denovo10783_f0 denovo10835_f0 denovo10861_f0 denovo10939_f0 denovo11017_f0 denovo11043_f0 denovo11095_f0 denovo11173_f0 denovo11199_f0 denovo11303_f0 denovo11329_f0 denovo11355_f0 denovo11407_f0 denovo11433_f0 denovo11459_f0 denovo11485_f0 denovo11563_f0 denovo11719_f0 denovo11745_f0 denovo11927_f0 denovo12083_f0 

Concatenation entropy_0.30_0.00_loci_100_to_299 will have the following data
OTU                           EOG69CQC1_1         EOG6SN1SH_1         EOG6PRSVJ_1         EOG6PRSVH_1         EOG60ZR1Z_1         EOG6FXRCN_1         EOG60P4BD_1         EOG6NCMGZ_1         EOG6K3M19_1         EOG63TZZV_1         EOG6G7C30_1         EOG68KRFS_1         EOG666VQ4_1         EOG698V44_1         EOG6F7NPM_1         EOG6894QQ_1         EOG6868GW_1         EOG69PB31_1         EOG6JDHBD_1         EOG68D102_1         EOG60ZR1T_1         EOG63JC89_1         EOG698V46_1         EOG6J6RV9_1         EOG68KRG8_1         EOG6N8R81_1         EOG63BMSV_1         EOG69KFV1_1         EOG6C2HF4_1         EOG6CC44S_1         EOG6FXRCK_1         EOG68KRG4_1         EOG61C70T_1         EOG6BK575_1         EOG6R506X_1         EOG6JT09G_1         EOG6F7NPJ_1         EOG61NTQM_1         EOG6SJ5J3_1         EOG6NZTZ3_1         EOG6J3WM7_1         EOG680J16_1         EOG68SGXM_1         EOG6RNBCT_1         EOG6RFKXS_1         EOG6B8JHB_1         EOG6QFWHZ_1         EOG6RFKXB_1         EOG647FXX_1         EOG6B8JHP_1         EOG6S1TBX_1         EOG6DFPRT_1         EOG65HS25_1         EOG6FFD5C_1         EOG6PNXM9_1         EOG6F4SFZ_1         EOG6Q8524_1         EOG669QZR_1         EOG6JT09B_1         EOG6M65N6_1         EOG6QRH81_1         EOG65HS1N_1         EOG6FR0VW_1         EOG6KKZ6X_1         EOG60VVTG_1         EOG6QC198_1         EOG6D26TG_1         EOG6FFD52_1         EOG634W9K_1         EOG6DNF7N_1         EOG61RPZF_1         EOG60GCVD_1         EOG605S4X_1         EOG65TCRN_1         EOG608ND4_1         EOG6640GX_1         EOG6SQX12_1         EOG69GKMS_1         EOG6B8JHC_1         EOG6Q2DJZ_1         EOG6D26TV_1         EOG6NS3G8_1         EOG64TPCQ_1         EOG6229Q2_1         EOG63TZZW_1         EOG6BG90S_1         EOG66DM62_1         EOG6QFWJH_1         EOG69CQBW_1         EOG6N04K4_1         EOG69KFTV_1         EOG62V8KS_1         EOG6C5CPH_1         EOG6FFD51_1         EOG6QNN0J_1         EOG6KSPP5_1         EOG6N5W22_1         EOG6PK2CG_1         EOG6NP779_1         EOG6933N9_1         EOG66Q6XP_1         EOG6JQ427_1         EOG6C5CNP_1         EOG6R5066_1         EOG6FTW4C_1         EOG641QFN_1         EOG65B1K2_1         EOG66WZD1_1         EOG65TCRK_1         EOG6FR0WR_1         EOG6P2Q64_1         EOG666VQC_1         EOG6PC9WF_1         EOG6Q58T0_1         EOG641QGH_1         EOG6933N7_1         EOG69S6BG_1         EOG6PK2CM_1         EOG6NS3FV_1         EOG65MN9B_1         EOG641QFW_1         EOG6N04J6_1         EOG6GMV1P_1         EOG6S4PKX_1         EOG6K6G7S_1         EOG6BZN62_1         EOG6SXNGX_1         EOG6QC18P_1         EOG6CG0CT_1         EOG605S4Q_1         EOG6R7VF9_1         EOG6CZBM5_1         EOG63R3RK_1         EOG64F6DK_1         EOG6FJ8D6_1         EOG6001NN_1         EOG695ZWC_1         EOG67PX9B_1         EOG6P8FNH_1         EOG6KSPQ0_1         EOG63R3RC_1         EOG6907D7_1         EOG6JWVHT_1         EOG68SGX8_1         EOG6KD6Q9_1         EOG68GW6Q_1         EOG6Q852G_1         EOG676K44_1         EOG64MXWZ_1         EOG6S1TC2_1         EOG6Q58SW_1         EOG6CRM3H_1         EOG6PRSVP_1         EOG6R5069_1         EOG676K40_1         EOG64MXWK_1         EOG6N30SN_1         EOG6DR9GZ_1         EOG6255XM_1         EOG62FSN7_1         EOG6H72G0_1         EOG6PZJB1_1         EOG65QHHP_1         EOG615GHV_1         EOG66WZCQ_1         EOG698V4H_1         EOG62BXCZ_1         EOG6CG0D0_1         EOG69KFVF_1         EOG615GHP_1         EOG66HGF5_1         EOG6DFPSF_1         EOG66MBPR_1         EOG6DFPSD_1         EOG67D9KC_1         EOG6HHP5S_1         EOG6DBTJX_1         EOG6DBTHZ_1         EOG6KH2ZD_1         EOG6STS87_1         EOG67D9KV_1         EOG61RPZ9_1         EOG637RJP_1         EOG66MBP7_1         EOG6JDHBG_1         EOG6DV5QM_1         EOG6FR0W5_1         EOG6K0QRT_1         EOG65DWSM_1         EOG6CC452_1         EOG6G4GTX_1         EOG64BB5H_1         EOG64J2NC_1         EOG6H72G3_1         EOG6SBF2B_1         EOG6FFD5V_1         EOG6G4GTT_1         EOG6GXFR5_1         EOG61C70W_1         EOG6KPTG0_1         
Dplexcds                      denovo2_f0     denovo80_f0    denovo158_f0   denovo184_f0   denovo262_f0   denovo314_f0   denovo366_f0   denovo444_f0   denovo522_f0   denovo678_f0   denovo834_f0   denovo1042_f0  denovo1146_f0  denovo1224_f0  denovo1250_f0  denovo1354_f0  denovo1380_f0  denovo1406_f0  denovo1484_f0  denovo1640_f0  denovo1666_f0  denovo1718_f0  denovo1796_f0  denovo1848_f0  denovo1926_f0  denovo1952_f0  denovo1978_f0  denovo2004_f0  denovo2082_f0  denovo2108_f0  denovo2134_f0  denovo2238_f0  denovo2264_f0  denovo2290_f0  denovo2316_f0  denovo2368_f0  denovo2472_f0  denovo2524_f0  denovo2550_f0  denovo2576_f0  denovo2602_f0  denovo2628_f0  denovo2654_f0  denovo2758_f0  denovo2836_f0  denovo2888_f0  denovo3148_f0  denovo3252_f0  denovo3304_f0  denovo3330_f0  denovo3434_f0  denovo3486_f0  denovo3590_f0  denovo3616_f0  denovo3642_f0  denovo3720_f0  denovo3746_f0  denovo3798_f0  denovo3824_f0  denovo3980_f0  denovo4058_f0  denovo4136_f0  denovo4240_f0  denovo4318_f0  denovo4344_f0  denovo4370_f0  denovo4396_f0  denovo4422_f0  denovo4500_f0  denovo4526_f0  denovo4630_f0  denovo4760_f0  denovo4838_f0  denovo4890_f0  denovo4916_f0  denovo4942_f0  denovo4968_f0  denovo4994_f0  denovo5098_f0  denovo5150_f0  denovo5202_f0  denovo5410_f0  denovo5436_f0  denovo5540_f0  denovo5566_f0  denovo5904_f0  denovo5956_f0  denovo6008_f0  denovo6034_f0  denovo6060_f0  denovo6086_f0  denovo6190_f0  denovo6242_f0  denovo6268_f0  denovo6294_f0  denovo6372_f0  denovo6502_f0  denovo6554_f0  denovo6580_f0  denovo6606_f0  denovo6658_f0  denovo6684_f0  denovo6710_f0  denovo6736_f0  denovo6762_f0  denovo6892_f0  denovo6970_f0  denovo6996_f0  denovo7022_f0  denovo7074_f0  denovo7126_f0  denovo7152_f0  denovo7282_f0  denovo7308_f0  denovo7334_f0  denovo7360_f0  denovo7386_f0  denovo7464_f0  denovo7490_f0  denovo7516_f0  denovo7542_f0  denovo7620_f0  denovo7672_f0  denovo7698_f0  denovo7776_f0  denovo7828_f0  denovo7906_f0  denovo8010_f0  denovo8036_f0  denovo8218_f0  denovo8244_f0  denovo8270_f0  denovo8348_f0  denovo8374_f0  denovo8400_f0  denovo8478_f0  denovo8530_f0  denovo8556_f0  denovo8608_f0  denovo8634_f0  denovo8686_f0  denovo8712_f0  denovo8738_f0  denovo8764_f0  denovo8894_f0  denovo8920_f0  denovo8946_f0  denovo8972_f0  denovo9102_f0  denovo9128_f0  denovo9232_f0  denovo9284_f0  denovo9310_f0  denovo9336_f0  denovo9440_f0  denovo9466_f0  denovo9492_f0  denovo9648_f0  denovo9700_f0  denovo9778_f0  denovo9804_f0  denovo9830_f0  denovo9882_f0  denovo9960_f0  denovo10012_f0 denovo10038_f0 denovo10194_f0 denovo10220_f0 denovo10272_f0 denovo10298_f0 denovo10428_f0 denovo10454_f0 denovo10506_f0 denovo10532_f0 denovo10558_f0 denovo10584_f0 denovo10662_f0 denovo10714_f0 denovo10740_f0 denovo10766_f0 denovo10818_f0 denovo11000_f0 denovo11026_f0 denovo11052_f0 denovo11182_f0 denovo11286_f0 denovo11338_f0 denovo11390_f0 denovo11416_f0 denovo11442_f0 denovo11468_f0 denovo11494_f0 denovo11520_f0 denovo11572_f0 denovo11598_f0 denovo11702_f0 denovo11728_f0 denovo11910_f0 denovo11936_f0 denovo12066_f0 
FG120077                      denovo10_f0    denovo88_f0    denovo166_f0   denovo192_f0   denovo270_f0   denovo322_f0   denovo374_f0   denovo452_f0   denovo530_f0   denovo686_f0   denovo842_f0   denovo1050_f0  denovo1154_f0  denovo1232_f0  denovo1258_f0  denovo1362_f0  denovo1388_f0  denovo1414_f0  denovo1492_f0  denovo1648_f0  denovo1674_f0  denovo1726_f0  denovo1804_f0  denovo1856_f0  denovo1934_f0  denovo1960_f0  denovo1986_f0  denovo2012_f0  denovo2090_f0  denovo2116_f0  denovo2142_f0  denovo2246_f0  denovo2272_f0  denovo2298_f0  denovo2324_f0  denovo2376_f0  denovo2480_f0  denovo2532_f0  denovo2558_f0  denovo2584_f0  denovo2610_f0  denovo2636_f0  denovo2662_f0  denovo2766_f0  denovo2844_f0  denovo2896_f0  denovo3156_f0  denovo3260_f0  denovo3312_f0  denovo3338_f0  denovo3442_f0  denovo3494_f0  denovo3598_f0  denovo3624_f0  denovo3650_f0  denovo3728_f0  denovo3754_f0  denovo3806_f0  denovo3832_f0  denovo3988_f0  denovo4066_f0  denovo4144_f0  denovo4248_f0  denovo4326_f0  denovo4352_f0  denovo4378_f0  denovo4404_f0  denovo4430_f0  denovo4508_f0  denovo4534_f0  denovo4638_f0  denovo4768_f0  denovo4846_f0  denovo4898_f0  denovo4924_f0  denovo4950_f0  denovo4976_f0  denovo5002_f0  denovo5106_f0  denovo5158_f0  denovo5210_f0  denovo5418_f0  denovo5444_f0  denovo5548_f0  denovo5574_f0  denovo5912_f0  denovo5964_f0  denovo6016_f0  denovo6042_f0  denovo6068_f0  denovo6094_f0  denovo6198_f0  denovo6250_f0  denovo6276_f0  denovo6302_f0  denovo6380_f0  denovo6510_f0  denovo6562_f0  denovo6588_f0  denovo6614_f0  denovo6666_f0  denovo6692_f0  denovo6718_f0  denovo6744_f0  denovo6770_f0  denovo6900_f0  denovo6978_f0  denovo7004_f0  denovo7030_f0  denovo7082_f0  denovo7134_f0  denovo7160_f0  denovo7290_f0  denovo7316_f0  denovo7342_f0  denovo7368_f0  denovo7394_f0  denovo7472_f0  denovo7498_f0  denovo7524_f0  denovo7550_f0  denovo7628_f0  denovo7680_f0  denovo7706_f0  denovo7784_f0  denovo7836_f0  denovo7914_f0  denovo8018_f0  denovo8044_f0  denovo8226_f0  denovo8252_f0  denovo8278_f0  denovo8356_f0  denovo8382_f0  denovo8408_f0  denovo8486_f0  denovo8538_f0  denovo8564_f0  denovo8616_f0  denovo8642_f0  denovo8694_f0  denovo8720_f0  denovo8746_f0  denovo8772_f0  denovo8902_f0  denovo8928_f0  denovo8954_f0  denovo8980_f0  denovo9110_f0  denovo9136_f0  denovo9240_f0  denovo9292_f0  denovo9318_f0  denovo9344_f0  denovo9448_f0  denovo9474_f0  denovo9500_f0  denovo9656_f0  denovo9708_f0  denovo9786_f0  denovo9812_f0  denovo9838_f0  denovo9890_f0  denovo9968_f0  denovo10020_f0 denovo10046_f0 denovo10202_f0 denovo10228_f0 denovo10280_f0 denovo10306_f0 denovo10436_f0 denovo10462_f0 denovo10514_f0 denovo10540_f0 denovo10566_f0 denovo10592_f0 denovo10670_f0 denovo10722_f0 denovo10748_f0 denovo10774_f0 denovo10826_f0 denovo11008_f0 denovo11034_f0 denovo11060_f0 denovo11190_f0 denovo11294_f0 denovo11346_f0 denovo11398_f0 denovo11424_f0 denovo11450_f0 denovo11476_f0 denovo11502_f0 denovo11528_f0 denovo11580_f0 denovo11606_f0 denovo11710_f0 denovo11736_f0 denovo11918_f0 denovo11944_f0 denovo12074_f0 
SRR803483                     denovo20_f0    denovo98_f0    denovo176_f0   denovo202_f0   denovo280_f0   denovo332_f0   denovo384_f0   denovo462_f0   denovo540_f0   denovo696_f0   denovo852_f0   denovo1060_f0  denovo1164_f0  denovo1242_f0  denovo1268_f0  denovo1372_f0  denovo1398_f0  denovo1424_f0  denovo1502_f0  denovo1658_f0  denovo1684_f0  denovo1736_f0  denovo1814_f0  denovo1866_f0  denovo1944_f0  denovo1970_f0  denovo1996_f0  denovo2022_f0  denovo2100_f0  denovo2126_f0  denovo2152_f0  denovo2256_f0  denovo2282_f0  denovo2308_f0  denovo2334_f0  denovo2386_f0  denovo2490_f0  denovo2542_f0  denovo2568_f0  denovo2594_f0  denovo2620_f0  denovo2646_f0  denovo2672_f0  denovo2776_f0  denovo2854_f0  denovo2906_f0  denovo3166_f0  denovo3270_f0  denovo3322_f0  denovo3348_f0  denovo3452_f0  denovo3504_f0  denovo3608_f0  denovo3634_f0  denovo3660_f0  denovo3738_f0  denovo3764_f0  denovo3816_f0  denovo3842_f0  denovo3998_f0  denovo4076_f0  denovo4154_f0  denovo4258_f0  denovo4336_f0  denovo4362_f0  denovo4388_f0  denovo4414_f0  denovo4440_f0  denovo4518_f0  denovo4544_f0  denovo4648_f0  denovo4778_f0  denovo4856_f0  denovo4908_f0  denovo4934_f0  denovo4960_f0  denovo4986_f0  denovo5012_f0  denovo5116_f0  denovo5168_f0  denovo5220_f0  denovo5428_f0  denovo5454_f0  denovo5558_f0  denovo5584_f0  denovo5922_f0  denovo5974_f0  denovo6026_f0  denovo6052_f0  denovo6078_f0  denovo6104_f0  denovo6208_f0  denovo6260_f0  denovo6286_f0  denovo6312_f0  denovo6390_f0  denovo6520_f0  denovo6572_f0  denovo6598_f0  denovo6624_f0  denovo6676_f0  denovo6702_f0  denovo6728_f0  denovo6754_f0  denovo6780_f0  denovo6910_f0  denovo6988_f0  denovo7014_f0  denovo7040_f0  denovo7092_f0  denovo7144_f0  denovo7170_f0  denovo7300_f0  denovo7326_f0  denovo7352_f0  denovo7378_f0  denovo7404_f0  denovo7482_f0  denovo7508_f0  denovo7534_f0  denovo7560_f0  denovo7638_f0  denovo7690_f0  denovo7716_f0  denovo7794_f0  denovo7846_f0  denovo7924_f0  denovo8028_f0  denovo8054_f0  denovo8236_f0  denovo8262_f0  denovo8288_f0  denovo8366_f0  denovo8392_f0  denovo8418_f0  denovo8496_f0  denovo8548_f0  denovo8574_f0  denovo8626_f0  denovo8652_f0  denovo8704_f0  denovo8730_f0  denovo8756_f0  denovo8782_f0  denovo8912_f0  denovo8938_f0  denovo8964_f0  denovo8990_f0  denovo9120_f0  denovo9146_f0  denovo9250_f0  denovo9302_f0  denovo9328_f0  denovo9354_f0  denovo9458_f0  denovo9484_f0  denovo9510_f0  denovo9666_f0  denovo9718_f0  denovo9796_f0  denovo9822_f0  denovo9848_f0  denovo9900_f0  denovo9978_f0  denovo10030_f0 denovo10056_f0 denovo10212_f0 denovo10238_f0 denovo10290_f0 denovo10316_f0 denovo10446_f0 denovo10472_f0 denovo10524_f0 denovo10550_f0 denovo10576_f0 denovo10602_f0 denovo10680_f0 denovo10732_f0 denovo10758_f0 denovo10784_f0 denovo10836_f0 denovo11018_f0 denovo11044_f0 denovo11070_f0 denovo11200_f0 denovo11304_f0 denovo11356_f0 denovo11408_f0 denovo11434_f0 denovo11460_f0 denovo11486_f0 denovo11512_f0 denovo11538_f0 denovo11590_f0 denovo11616_f0 denovo11720_f0 denovo11746_f0 denovo11928_f0 denovo11954_f0 denovo12084_f0 
FG120035                      denovo5_f0     denovo83_f0    denovo161_f0   denovo187_f0   denovo265_f0   denovo317_f0   denovo369_f0   denovo447_f0   denovo525_f0   denovo681_f0   denovo837_f0   denovo1045_f0  denovo1149_f0  denovo1227_f0  denovo1253_f0  denovo1357_f0  denovo1383_f0  denovo1409_f0  denovo1487_f0  denovo1643_f0  denovo1669_f0  denovo1721_f0  denovo1799_f0  denovo1851_f0  denovo1929_f0  denovo1955_f0  denovo1981_f0  denovo2007_f0  denovo2085_f0  denovo2111_f0  denovo2137_f0  denovo2241_f0  denovo2267_f0  denovo2293_f0  denovo2319_f0  denovo2371_f0  denovo2475_f0  denovo2527_f0  denovo2553_f0  denovo2579_f0  denovo2605_f0  denovo2631_f0  denovo2657_f0  denovo2761_f0  denovo2839_f0  denovo2891_f0  denovo3151_f0  denovo3255_f0  denovo3307_f0  denovo3333_f0  denovo3437_f0  denovo3489_f0  denovo3593_f0  denovo3619_f0  denovo3645_f0  denovo3723_f0  denovo3749_f0  denovo3801_f0  denovo3827_f0  denovo3983_f0  denovo4061_f0  denovo4139_f0  denovo4243_f0  denovo4321_f0  denovo4347_f0  denovo4373_f0  denovo4399_f0  denovo4425_f0  denovo4503_f0  denovo4529_f0  denovo4633_f0  denovo4763_f0  denovo4841_f0  denovo4893_f0  denovo4919_f0  denovo4945_f0  denovo4971_f0  denovo4997_f0  denovo5101_f0  denovo5153_f0  denovo5205_f0  denovo5413_f0  denovo5439_f0  denovo5543_f0  denovo5569_f0  denovo5907_f0  denovo5959_f0  denovo6011_f0  denovo6037_f0  denovo6063_f0  denovo6089_f0  denovo6193_f0  denovo6245_f0  denovo6271_f0  denovo6297_f0  denovo6375_f0  denovo6505_f0  denovo6557_f0  denovo6583_f0  denovo6609_f0  denovo6661_f0  denovo6687_f0  denovo6713_f0  denovo6739_f0  denovo6765_f0  denovo6895_f0  denovo6973_f0  denovo6999_f0  denovo7025_f0  denovo7077_f0  denovo7129_f0  denovo7155_f0  denovo7285_f0  denovo7311_f0  denovo7337_f0  denovo7363_f0  denovo7389_f0  denovo7467_f0  denovo7493_f0  denovo7519_f0  denovo7545_f0  denovo7623_f0  denovo7675_f0  denovo7701_f0  denovo7779_f0  denovo7831_f0  denovo7909_f0  denovo8013_f0  denovo8039_f0  denovo8221_f0  denovo8247_f0  denovo8273_f0  denovo8351_f0  denovo8377_f0  denovo8403_f0  denovo8481_f0  denovo8533_f0  denovo8559_f0  denovo8611_f0  denovo8637_f0  denovo8689_f0  denovo8715_f0  denovo8741_f0  denovo8767_f0  denovo8897_f0  denovo8923_f0  denovo8949_f0  denovo8975_f0  denovo9105_f0  denovo9131_f0  denovo9235_f0  denovo9287_f0  denovo9313_f0  denovo9339_f0  denovo9443_f0  denovo9469_f0  denovo9495_f0  denovo9651_f0  denovo9703_f0  denovo9781_f0  denovo9807_f0  denovo9833_f0  denovo9885_f0  denovo9963_f0  denovo10015_f0 denovo10041_f0 denovo10197_f0 denovo10223_f0 denovo10275_f0 denovo10301_f0 denovo10431_f0 denovo10457_f0 denovo10509_f0 denovo10535_f0 denovo10561_f0 denovo10587_f0 denovo10665_f0 denovo10717_f0 denovo10743_f0 denovo10769_f0 denovo10821_f0 denovo11003_f0 denovo11029_f0 denovo11055_f0 denovo11185_f0 denovo11289_f0 denovo11341_f0 denovo11393_f0 denovo11419_f0 denovo11445_f0 denovo11471_f0 denovo11497_f0 denovo11523_f0 denovo11575_f0 denovo11601_f0 denovo11705_f0 denovo11731_f0 denovo11913_f0 denovo11939_f0 denovo12069_f0 
FG120046B                     denovo6_f0     denovo84_f0    denovo162_f0   denovo188_f0   denovo266_f0   denovo318_f0   denovo370_f0   denovo448_f0   denovo526_f0   denovo682_f0   denovo838_f0   denovo1046_f0  denovo1150_f0  denovo1228_f0  denovo1254_f0  denovo1358_f0  denovo1384_f0  denovo1410_f0  denovo1488_f0  denovo1644_f0  denovo1670_f0  denovo1722_f0  denovo1800_f0  denovo1852_f0  denovo1930_f0  denovo1956_f0  denovo1982_f0  denovo2008_f0  denovo2086_f0  denovo2112_f0  denovo2138_f0  denovo2242_f0  denovo2268_f0  denovo2294_f0  denovo2320_f0  denovo2372_f0  denovo2476_f0  denovo2528_f0  denovo2554_f0  denovo2580_f0  denovo2606_f0  denovo2632_f0  denovo2658_f0  denovo2762_f0  denovo2840_f0  denovo2892_f0  denovo3152_f0  denovo3256_f0  denovo3308_f0  denovo3334_f0  denovo3438_f0  denovo3490_f0  denovo3594_f0  denovo3620_f0  denovo3646_f0  denovo3724_f0  denovo3750_f0  denovo3802_f0  denovo3828_f0  denovo3984_f0  denovo4062_f0  denovo4140_f0  denovo4244_f0  denovo4322_f0  denovo4348_f0  denovo4374_f0  denovo4400_f0  denovo4426_f0  denovo4504_f0  denovo4530_f0  denovo4634_f0  denovo4764_f0  denovo4842_f0  denovo4894_f0  denovo4920_f0  denovo4946_f0  denovo4972_f0  denovo4998_f0  denovo5102_f0  denovo5154_f0  denovo5206_f0  denovo5414_f0  denovo5440_f0  denovo5544_f0  denovo5570_f0  denovo5908_f0  denovo5960_f0  denovo6012_f0  denovo6038_f0  denovo6064_f0  denovo6090_f0  denovo6194_f0  denovo6246_f0  denovo6272_f0  denovo6298_f0  denovo6376_f0  denovo6506_f0  denovo6558_f0  denovo6584_f0  denovo6610_f0  denovo6662_f0  denovo6688_f0  denovo6714_f0  denovo6740_f0  denovo6766_f0  denovo6896_f0  denovo6974_f0  denovo7000_f0  denovo7026_f0  denovo7078_f0  denovo7130_f0  denovo7156_f0  denovo7286_f0  denovo7312_f0  denovo7338_f0  denovo7364_f0  denovo7390_f0  denovo7468_f0  denovo7494_f0  denovo7520_f0  denovo7546_f0  denovo7624_f0  denovo7676_f0  denovo7702_f0  denovo7780_f0  denovo7832_f0  denovo7910_f0  denovo8014_f0  denovo8040_f0  denovo8222_f0  denovo8248_f0  denovo8274_f0  denovo8352_f0  denovo8378_f0  denovo8404_f0  denovo8482_f0  denovo8534_f0  denovo8560_f0  denovo8612_f0  denovo8638_f0  denovo8690_f0  denovo8716_f0  denovo8742_f0  denovo8768_f0  denovo8898_f0  denovo8924_f0  denovo8950_f0  denovo8976_f0  denovo9106_f0  denovo9132_f0  denovo9236_f0  denovo9288_f0  denovo9314_f0  denovo9340_f0  denovo9444_f0  denovo9470_f0  denovo9496_f0  denovo9652_f0  denovo9704_f0  denovo9782_f0  denovo9808_f0  denovo9834_f0  denovo9886_f0  denovo9964_f0  denovo10016_f0 denovo10042_f0 denovo10198_f0 denovo10224_f0 denovo10276_f0 denovo10302_f0 denovo10432_f0 denovo10458_f0 denovo10510_f0 denovo10536_f0 denovo10562_f0 denovo10588_f0 denovo10666_f0 denovo10718_f0 denovo10744_f0 denovo10770_f0 denovo10822_f0 denovo11004_f0 denovo11030_f0 denovo11056_f0 denovo11186_f0 denovo11290_f0 denovo11342_f0 denovo11394_f0 denovo11420_f0 denovo11446_f0 denovo11472_f0 denovo11498_f0 denovo11524_f0 denovo11576_f0 denovo11602_f0 denovo11706_f0 denovo11732_f0 denovo11914_f0 denovo11940_f0 denovo12070_f0 
GNV129007                     denovo15_f0    denovo93_f0    denovo171_f0   denovo197_f0   denovo275_f0   denovo327_f0   denovo379_f0   denovo457_f0   denovo535_f0   denovo691_f0   denovo847_f0   denovo1055_f0  denovo1159_f0  denovo1237_f0  denovo1263_f0  denovo1367_f0  denovo1393_f0  denovo1419_f0  denovo1497_f0  denovo1653_f0  denovo1679_f0  denovo1731_f0  denovo1809_f0  denovo1861_f0  denovo1939_f0  denovo1965_f0  denovo1991_f0  denovo2017_f0  denovo2095_f0  denovo2121_f0  denovo2147_f0  denovo2251_f0  denovo2277_f0  denovo2303_f0  denovo2329_f0  denovo2381_f0  denovo2485_f0  denovo2537_f0  denovo2563_f0  denovo2589_f0  denovo2615_f0  denovo2641_f0  denovo2667_f0  denovo2771_f0  denovo2849_f0  denovo2901_f0  denovo3161_f0  denovo3265_f0  denovo3317_f0  denovo3343_f0  denovo3447_f0  denovo3499_f0  denovo3603_f0  denovo3629_f0  denovo3655_f0  denovo3733_f0  denovo3759_f0  denovo3811_f0  denovo3837_f0  denovo3993_f0  denovo4071_f0  denovo4149_f0  denovo4253_f0  denovo4331_f0  denovo4357_f0  denovo4383_f0  denovo4409_f0  denovo4435_f0  denovo4513_f0  denovo4539_f0  denovo4643_f0  denovo4773_f0  denovo4851_f0  denovo4903_f0  denovo4929_f0  denovo4955_f0  denovo4981_f0  denovo5007_f0  denovo5111_f0  denovo5163_f0  denovo5215_f0  denovo5423_f0  denovo5449_f0  denovo5553_f0  denovo5579_f0  denovo5917_f0  denovo5969_f0  denovo6021_f0  denovo6047_f0  denovo6073_f0  denovo6099_f0  denovo6203_f0  denovo6255_f0  denovo6281_f0  denovo6307_f0  denovo6385_f0  denovo6515_f0  denovo6567_f0  denovo6593_f0  denovo6619_f0  denovo6671_f0  denovo6697_f0  denovo6723_f0  denovo6749_f0  denovo6775_f0  denovo6905_f0  denovo6983_f0  denovo7009_f0  denovo7035_f0  denovo7087_f0  denovo7139_f0  denovo7165_f0  denovo7295_f0  denovo7321_f0  denovo7347_f0  denovo7373_f0  denovo7399_f0  denovo7477_f0  denovo7503_f0  denovo7529_f0  denovo7555_f0  denovo7633_f0  denovo7685_f0  denovo7711_f0  denovo7789_f0  denovo7841_f0  denovo7919_f0  denovo8023_f0  denovo8049_f0  denovo8231_f0  denovo8257_f0  denovo8283_f0  denovo8361_f0  denovo8387_f0  denovo8413_f0  denovo8491_f0  denovo8543_f0  denovo8569_f0  denovo8621_f0  denovo8647_f0  denovo8699_f0  denovo8725_f0  denovo8751_f0  denovo8777_f0  denovo8907_f0  denovo8933_f0  denovo8959_f0  denovo8985_f0  denovo9115_f0  denovo9141_f0  denovo9245_f0  denovo9297_f0  denovo9323_f0  denovo9349_f0  denovo9453_f0  denovo9479_f0  denovo9505_f0  denovo9661_f0  denovo9713_f0  denovo9791_f0  denovo9817_f0  denovo9843_f0  denovo9895_f0  denovo9973_f0  denovo10025_f0 denovo10051_f0 denovo10207_f0 denovo10233_f0 denovo10285_f0 denovo10311_f0 denovo10441_f0 denovo10467_f0 denovo10519_f0 denovo10545_f0 denovo10571_f0 denovo10597_f0 denovo10675_f0 denovo10727_f0 denovo10753_f0 denovo10779_f0 denovo10831_f0 denovo11013_f0 denovo11039_f0 denovo11065_f0 denovo11195_f0 denovo11299_f0 denovo11351_f0 denovo11403_f0 denovo11429_f0 denovo11455_f0 denovo11481_f0 denovo11507_f0 denovo11533_f0 denovo11585_f0 denovo11611_f0 denovo11715_f0 denovo11741_f0 denovo11923_f0 denovo11949_f0 denovo12079_f0 
SW130126                      denovo24_f0    denovo102_f0   denovo180_f0   denovo206_f0   denovo284_f0   denovo336_f0   denovo388_f0   denovo466_f0   denovo544_f0   denovo700_f0   denovo856_f0   denovo1064_f0  denovo1168_f0  denovo1246_f0  denovo1272_f0  denovo1376_f0  denovo1402_f0  denovo1428_f0  denovo1506_f0  denovo1662_f0  denovo1688_f0  denovo1740_f0  denovo1818_f0  denovo1870_f0  denovo1948_f0  denovo1974_f0  denovo2000_f0  denovo2026_f0  denovo2104_f0  denovo2130_f0  denovo2156_f0  denovo2260_f0  denovo2286_f0  denovo2312_f0  denovo2338_f0  denovo2390_f0  denovo2494_f0  denovo2546_f0  denovo2572_f0  denovo2598_f0  denovo2624_f0  denovo2650_f0  denovo2676_f0  denovo2780_f0  denovo2858_f0  denovo2910_f0  denovo3170_f0  denovo3274_f0  denovo3326_f0  denovo3352_f0  denovo3456_f0  denovo3508_f0  denovo3612_f0  denovo3638_f0  denovo3664_f0  denovo3742_f0  denovo3768_f0  denovo3820_f0  denovo3846_f0  denovo4002_f0  denovo4080_f0  denovo4158_f0  denovo4262_f0  denovo4340_f0  denovo4366_f0  denovo4392_f0  denovo4418_f0  denovo4444_f0  denovo4522_f0  denovo4548_f0  denovo4652_f0  denovo4782_f0  denovo4860_f0  denovo4912_f0  denovo4938_f0  denovo4964_f0  denovo4990_f0  denovo5016_f0  denovo5120_f0  denovo5172_f0  denovo5224_f0  denovo5432_f0  denovo5458_f0  denovo5562_f0  denovo5588_f0  denovo5926_f0  denovo5978_f0  denovo6030_f0  denovo6056_f0  denovo6082_f0  denovo6108_f0  denovo6212_f0  denovo6264_f0  denovo6290_f0  denovo6316_f0  denovo6394_f0  denovo6524_f0  denovo6576_f0  denovo6602_f0  denovo6628_f0  denovo6680_f0  denovo6706_f0  denovo6732_f0  denovo6758_f0  denovo6784_f0  denovo6914_f0  denovo6992_f0  denovo7018_f0  denovo7044_f0  denovo7096_f0  denovo7148_f0  denovo7174_f0  denovo7304_f0  denovo7330_f0  denovo7356_f0  denovo7382_f0  denovo7408_f0  denovo7486_f0  denovo7512_f0  denovo7538_f0  denovo7564_f0  denovo7642_f0  denovo7694_f0  denovo7720_f0  denovo7798_f0  denovo7850_f0  denovo7928_f0  denovo8032_f0  denovo8058_f0  denovo8240_f0  denovo8266_f0  denovo8292_f0  denovo8370_f0  denovo8396_f0  denovo8422_f0  denovo8500_f0  denovo8552_f0  denovo8578_f0  denovo8630_f0  denovo8656_f0  denovo8708_f0  denovo8734_f0  denovo8760_f0  denovo8786_f0  denovo8916_f0  denovo8942_f0  denovo8968_f0  denovo8994_f0  denovo9124_f0  denovo9150_f0  denovo9254_f0  denovo9306_f0  denovo9332_f0  denovo9358_f0  denovo9462_f0  denovo9488_f0  denovo9514_f0  denovo9670_f0  denovo9722_f0  denovo9800_f0  denovo9826_f0  denovo9852_f0  denovo9904_f0  denovo9982_f0  denovo10034_f0 denovo10060_f0 denovo10216_f0 denovo10242_f0 denovo10294_f0 denovo10320_f0 denovo10450_f0 denovo10476_f0 denovo10528_f0 denovo10554_f0 denovo10580_f0 denovo10606_f0 denovo10684_f0 denovo10736_f0 denovo10762_f0 denovo10788_f0 denovo10840_f0 denovo11022_f0 denovo11048_f0 denovo11074_f0 denovo11204_f0 denovo11308_f0 denovo11360_f0 denovo11412_f0 denovo11438_f0 denovo11464_f0 denovo11490_f0 denovo11516_f0 denovo11542_f0 denovo11594_f0 denovo11620_f0 denovo11724_f0 denovo11750_f0 denovo11932_f0 denovo11958_f0 denovo12088_f0 
SW130103                      denovo23_f0    denovo101_f0   denovo179_f0   denovo205_f0   denovo283_f0   denovo335_f0   denovo387_f0   denovo465_f0   denovo543_f0   denovo699_f0   denovo855_f0   denovo1063_f0  denovo1167_f0  denovo1245_f0  denovo1271_f0  denovo1375_f0  denovo1401_f0  denovo1427_f0  denovo1505_f0  denovo1661_f0  denovo1687_f0  denovo1739_f0  denovo1817_f0  denovo1869_f0  denovo1947_f0  denovo1973_f0  denovo1999_f0  denovo2025_f0  denovo2103_f0  denovo2129_f0  denovo2155_f0  denovo2259_f0  denovo2285_f0  denovo2311_f0  denovo2337_f0  denovo2389_f0  denovo2493_f0  denovo2545_f0  denovo2571_f0  denovo2597_f0  denovo2623_f0  denovo2649_f0  denovo2675_f0  denovo2779_f0  denovo2857_f0  denovo2909_f0  denovo3169_f0  denovo3273_f0  denovo3325_f0  denovo3351_f0  denovo3455_f0  denovo3507_f0  denovo3611_f0  denovo3637_f0  denovo3663_f0  denovo3741_f0  denovo3767_f0  denovo3819_f0  denovo3845_f0  denovo4001_f0  denovo4079_f0  denovo4157_f0  denovo4261_f0  denovo4339_f0  denovo4365_f0  denovo4391_f0  denovo4417_f0  denovo4443_f0  denovo4521_f0  denovo4547_f0  denovo4651_f0  denovo4781_f0  denovo4859_f0  denovo4911_f0  denovo4937_f0  denovo4963_f0  denovo4989_f0  denovo5015_f0  denovo5119_f0  denovo5171_f0  denovo5223_f0  denovo5431_f0  denovo5457_f0  denovo5561_f0  denovo5587_f0  denovo5925_f0  denovo5977_f0  denovo6029_f0  denovo6055_f0  denovo6081_f0  denovo6107_f0  denovo6211_f0  denovo6263_f0  denovo6289_f0  denovo6315_f0  denovo6393_f0  denovo6523_f0  denovo6575_f0  denovo6601_f0  denovo6627_f0  denovo6679_f0  denovo6705_f0  denovo6731_f0  denovo6757_f0  denovo6783_f0  denovo6913_f0  denovo6991_f0  denovo7017_f0  denovo7043_f0  denovo7095_f0  denovo7147_f0  denovo7173_f0  denovo7303_f0  denovo7329_f0  denovo7355_f0  denovo7381_f0  denovo7407_f0  denovo7485_f0  denovo7511_f0  denovo7537_f0  denovo7563_f0  denovo7641_f0  denovo7693_f0  denovo7719_f0  denovo7797_f0  denovo7849_f0  denovo7927_f0  denovo8031_f0  denovo8057_f0  denovo8239_f0  denovo8265_f0  denovo8291_f0  denovo8369_f0  denovo8395_f0  denovo8421_f0  denovo8499_f0  denovo8551_f0  denovo8577_f0  denovo8629_f0  denovo8655_f0  denovo8707_f0  denovo8733_f0  denovo8759_f0  denovo8785_f0  denovo8915_f0  denovo8941_f0  denovo8967_f0  denovo8993_f0  denovo9123_f0  denovo9149_f0  denovo9253_f0  denovo9305_f0  denovo9331_f0  denovo9357_f0  denovo9461_f0  denovo9487_f0  denovo9513_f0  denovo9669_f0  denovo9721_f0  denovo9799_f0  denovo9825_f0  denovo9851_f0  denovo9903_f0  denovo9981_f0  denovo10033_f0 denovo10059_f0 denovo10215_f0 denovo10241_f0 denovo10293_f0 denovo10319_f0 denovo10449_f0 denovo10475_f0 denovo10527_f0 denovo10553_f0 denovo10579_f0 denovo10605_f0 denovo10683_f0 denovo10735_f0 denovo10761_f0 denovo10787_f0 denovo10839_f0 denovo11021_f0 denovo11047_f0 denovo11073_f0 denovo11203_f0 denovo11307_f0 denovo11359_f0 denovo11411_f0 denovo11437_f0 denovo11463_f0 denovo11489_f0 denovo11515_f0 denovo11541_f0 denovo11593_f0 denovo11619_f0 denovo11723_f0 denovo11749_f0 denovo11931_f0 denovo11957_f0 denovo12087_f0 
Callid                        denovo1_f0     denovo79_f0    denovo157_f0   denovo183_f0   denovo261_f0   denovo313_f0   denovo365_f0   denovo443_f0   denovo521_f0   denovo677_f0   denovo833_f0   denovo1041_f0  denovo1145_f0  denovo1223_f0  denovo1249_f0  denovo1353_f0  denovo1379_f0  denovo1405_f0  denovo1483_f0  denovo1639_f0  denovo1665_f0  denovo1717_f0  denovo1795_f0  denovo1847_f0  denovo1925_f0  denovo1951_f0  denovo1977_f0  denovo2003_f0  denovo2081_f0  denovo2107_f0  denovo2133_f0  denovo2237_f0  denovo2263_f0  denovo2289_f0  denovo2315_f0  denovo2367_f0  denovo2471_f0  denovo2523_f0  denovo2549_f0  denovo2575_f0  denovo2601_f0  denovo2627_f0  denovo2653_f0  denovo2757_f0  denovo2835_f0  denovo2887_f0  denovo3147_f0  denovo3251_f0  denovo3303_f0  denovo3329_f0  denovo3433_f0  denovo3485_f0  denovo3589_f0  denovo3615_f0  denovo3641_f0  denovo3719_f0  denovo3745_f0  denovo3797_f0  denovo3823_f0  denovo3979_f0  denovo4057_f0  denovo4135_f0  denovo4239_f0  denovo4317_f0  denovo4343_f0  denovo4369_f0  denovo4395_f0  denovo4421_f0  denovo4499_f0  denovo4525_f0  denovo4629_f0  denovo4759_f0  denovo4837_f0  denovo4889_f0  denovo4915_f0  denovo4941_f0  denovo4967_f0  denovo4993_f0  denovo5097_f0  denovo5149_f0  denovo5201_f0  denovo5409_f0  denovo5435_f0  denovo5539_f0  denovo5565_f0  denovo5903_f0  denovo5955_f0  denovo6007_f0  denovo6033_f0  denovo6059_f0  denovo6085_f0  denovo6189_f0  denovo6241_f0  denovo6267_f0  denovo6293_f0  denovo6371_f0  denovo6501_f0  denovo6553_f0  denovo6579_f0  denovo6605_f0  denovo6657_f0  denovo6683_f0  denovo6709_f0  denovo6735_f0  denovo6761_f0  denovo6891_f0  denovo6969_f0  denovo6995_f0  denovo7021_f0  denovo7073_f0  denovo7125_f0  denovo7151_f0  denovo7281_f0  denovo7307_f0  denovo7333_f0  denovo7359_f0  denovo7385_f0  denovo7463_f0  denovo7489_f0  denovo7515_f0  denovo7541_f0  denovo7619_f0  denovo7671_f0  denovo7697_f0  denovo7775_f0  denovo7827_f0  denovo7905_f0  denovo8009_f0  denovo8035_f0  denovo8217_f0  denovo8243_f0  denovo8269_f0  denovo8347_f0  denovo8373_f0  denovo8399_f0  denovo8477_f0  denovo8529_f0  denovo8555_f0  denovo8607_f0  denovo8633_f0  denovo8685_f0  denovo8711_f0  denovo8737_f0  denovo8763_f0  denovo8893_f0  denovo8919_f0  denovo8945_f0  denovo8971_f0  denovo9101_f0  denovo9127_f0  denovo9231_f0  denovo9283_f0  denovo9309_f0  denovo9335_f0  denovo9439_f0  denovo9465_f0  denovo9491_f0  denovo9647_f0  denovo9699_f0  denovo9777_f0  denovo9803_f0  denovo9829_f0  denovo9881_f0  denovo9959_f0  denovo10011_f0 denovo10037_f0 denovo10193_f0 denovo10219_f0 denovo10271_f0 denovo10297_f0 denovo10427_f0 denovo10453_f0 denovo10505_f0 denovo10531_f0 denovo10557_f0 denovo10583_f0 denovo10661_f0 denovo10713_f0 denovo10739_f0 denovo10765_f0 denovo10817_f0 denovo10999_f0 denovo11025_f0 denovo11051_f0 denovo11181_f0 denovo11285_f0 denovo11337_f0 denovo11389_f0 denovo11415_f0 denovo11441_f0 denovo11467_f0 denovo11493_f0 denovo11519_f0 denovo11571_f0 denovo11597_f0 denovo11701_f0 denovo11727_f0 denovo11909_f0 denovo11935_f0 denovo12065_f0 
FG120070B                     denovo8_f0     denovo86_f0    denovo164_f0   denovo190_f0   denovo268_f0   denovo320_f0   denovo372_f0   denovo450_f0   denovo528_f0   denovo684_f0   denovo840_f0   denovo1048_f0  denovo1152_f0  denovo1230_f0  denovo1256_f0  denovo1360_f0  denovo1386_f0  denovo1412_f0  denovo1490_f0  denovo1646_f0  denovo1672_f0  denovo1724_f0  denovo1802_f0  denovo1854_f0  denovo1932_f0  denovo1958_f0  denovo1984_f0  denovo2010_f0  denovo2088_f0  denovo2114_f0  denovo2140_f0  denovo2244_f0  denovo2270_f0  denovo2296_f0  denovo2322_f0  denovo2374_f0  denovo2478_f0  denovo2530_f0  denovo2556_f0  denovo2582_f0  denovo2608_f0  denovo2634_f0  denovo2660_f0  denovo2764_f0  denovo2842_f0  denovo2894_f0  denovo3154_f0  denovo3258_f0  denovo3310_f0  denovo3336_f0  denovo3440_f0  denovo3492_f0  denovo3596_f0  denovo3622_f0  denovo3648_f0  denovo3726_f0  denovo3752_f0  denovo3804_f0  denovo3830_f0  denovo3986_f0  denovo4064_f0  denovo4142_f0  denovo4246_f0  denovo4324_f0  denovo4350_f0  denovo4376_f0  denovo4402_f0  denovo4428_f0  denovo4506_f0  denovo4532_f0  denovo4636_f0  denovo4766_f0  denovo4844_f0  denovo4896_f0  denovo4922_f0  denovo4948_f0  denovo4974_f0  denovo5000_f0  denovo5104_f0  denovo5156_f0  denovo5208_f0  denovo5416_f0  denovo5442_f0  denovo5546_f0  denovo5572_f0  denovo5910_f0  denovo5962_f0  denovo6014_f0  denovo6040_f0  denovo6066_f0  denovo6092_f0  denovo6196_f0  denovo6248_f0  denovo6274_f0  denovo6300_f0  denovo6378_f0  denovo6508_f0  denovo6560_f0  denovo6586_f0  denovo6612_f0  denovo6664_f0  denovo6690_f0  denovo6716_f0  denovo6742_f0  denovo6768_f0  denovo6898_f0  denovo6976_f0  denovo7002_f0  denovo7028_f0  denovo7080_f0  denovo7132_f0  denovo7158_f0  denovo7288_f0  denovo7314_f0  denovo7340_f0  denovo7366_f0  denovo7392_f0  denovo7470_f0  denovo7496_f0  denovo7522_f0  denovo7548_f0  denovo7626_f0  denovo7678_f0  denovo7704_f0  denovo7782_f0  denovo7834_f0  denovo7912_f0  denovo8016_f0  denovo8042_f0  denovo8224_f0  denovo8250_f0  denovo8276_f0  denovo8354_f0  denovo8380_f0  denovo8406_f0  denovo8484_f0  denovo8536_f0  denovo8562_f0  denovo8614_f0  denovo8640_f0  denovo8692_f0  denovo8718_f0  denovo8744_f0  denovo8770_f0  denovo8900_f0  denovo8926_f0  denovo8952_f0  denovo8978_f0  denovo9108_f0  denovo9134_f0  denovo9238_f0  denovo9290_f0  denovo9316_f0  denovo9342_f0  denovo9446_f0  denovo9472_f0  denovo9498_f0  denovo9654_f0  denovo9706_f0  denovo9784_f0  denovo9810_f0  denovo9836_f0  denovo9888_f0  denovo9966_f0  denovo10018_f0 denovo10044_f0 denovo10200_f0 denovo10226_f0 denovo10278_f0 denovo10304_f0 denovo10434_f0 denovo10460_f0 denovo10512_f0 denovo10538_f0 denovo10564_f0 denovo10590_f0 denovo10668_f0 denovo10720_f0 denovo10746_f0 denovo10772_f0 denovo10824_f0 denovo11006_f0 denovo11032_f0 denovo11058_f0 denovo11188_f0 denovo11292_f0 denovo11344_f0 denovo11396_f0 denovo11422_f0 denovo11448_f0 denovo11474_f0 denovo11500_f0 denovo11526_f0 denovo11578_f0 denovo11604_f0 denovo11708_f0 denovo11734_f0 denovo11916_f0 denovo11942_f0 denovo12072_f0 
SW130007                      denovo22_f0    denovo100_f0   denovo178_f0   denovo204_f0   denovo282_f0   denovo334_f0   denovo386_f0   denovo464_f0   denovo542_f0   denovo698_f0   denovo854_f0   denovo1062_f0  denovo1166_f0  denovo1244_f0  denovo1270_f0  denovo1374_f0  denovo1400_f0  denovo1426_f0  denovo1504_f0  denovo1660_f0  denovo1686_f0  denovo1738_f0  denovo1816_f0  denovo1868_f0  denovo1946_f0  denovo1972_f0  denovo1998_f0  denovo2024_f0  denovo2102_f0  denovo2128_f0  denovo2154_f0  denovo2258_f0  denovo2284_f0  denovo2310_f0  denovo2336_f0  denovo2388_f0  denovo2492_f0  denovo2544_f0  denovo2570_f0  denovo2596_f0  denovo2622_f0  denovo2648_f0  denovo2674_f0  denovo2778_f0  denovo2856_f0  denovo2908_f0  denovo3168_f0  denovo3272_f0  denovo3324_f0  denovo3350_f0  denovo3454_f0  denovo3506_f0  denovo3610_f0  denovo3636_f0  denovo3662_f0  denovo3740_f0  denovo3766_f0  denovo3818_f0  denovo3844_f0  denovo4000_f0  denovo4078_f0  denovo4156_f0  denovo4260_f0  denovo4338_f0  denovo4364_f0  denovo4390_f0  denovo4416_f0  denovo4442_f0  denovo4520_f0  denovo4546_f0  denovo4650_f0  denovo4780_f0  denovo4858_f0  denovo4910_f0  denovo4936_f0  denovo4962_f0  denovo4988_f0  denovo5014_f0  denovo5118_f0  denovo5170_f0  denovo5222_f0  denovo5430_f0  denovo5456_f0  denovo5560_f0  denovo5586_f0  denovo5924_f0  denovo5976_f0  denovo6028_f0  denovo6054_f0  denovo6080_f0  denovo6106_f0  denovo6210_f0  denovo6262_f0  denovo6288_f0  denovo6314_f0  denovo6392_f0  denovo6522_f0  denovo6574_f0  denovo6600_f0  denovo6626_f0  denovo6678_f0  denovo6704_f0  denovo6730_f0  denovo6756_f0  denovo6782_f0  denovo6912_f0  denovo6990_f0  denovo7016_f0  denovo7042_f0  denovo7094_f0  denovo7146_f0  denovo7172_f0  denovo7302_f0  denovo7328_f0  denovo7354_f0  denovo7380_f0  denovo7406_f0  denovo7484_f0  denovo7510_f0  denovo7536_f0  denovo7562_f0  denovo7640_f0  denovo7692_f0  denovo7718_f0  denovo7796_f0  denovo7848_f0  denovo7926_f0  denovo8030_f0  denovo8056_f0  denovo8238_f0  denovo8264_f0  denovo8290_f0  denovo8368_f0  denovo8394_f0  denovo8420_f0  denovo8498_f0  denovo8550_f0  denovo8576_f0  denovo8628_f0  denovo8654_f0  denovo8706_f0  denovo8732_f0  denovo8758_f0  denovo8784_f0  denovo8914_f0  denovo8940_f0  denovo8966_f0  denovo8992_f0  denovo9122_f0  denovo9148_f0  denovo9252_f0  denovo9304_f0  denovo9330_f0  denovo9356_f0  denovo9460_f0  denovo9486_f0  denovo9512_f0  denovo9668_f0  denovo9720_f0  denovo9798_f0  denovo9824_f0  denovo9850_f0  denovo9902_f0  denovo9980_f0  denovo10032_f0 denovo10058_f0 denovo10214_f0 denovo10240_f0 denovo10292_f0 denovo10318_f0 denovo10448_f0 denovo10474_f0 denovo10526_f0 denovo10552_f0 denovo10578_f0 denovo10604_f0 denovo10682_f0 denovo10734_f0 denovo10760_f0 denovo10786_f0 denovo10838_f0 denovo11020_f0 denovo11046_f0 denovo11072_f0 denovo11202_f0 denovo11306_f0 denovo11358_f0 denovo11410_f0 denovo11436_f0 denovo11462_f0 denovo11488_f0 denovo11514_f0 denovo11540_f0 denovo11592_f0 denovo11618_f0 denovo11722_f0 denovo11748_f0 denovo11930_f0 denovo11956_f0 denovo12086_f0 
GNV120032                     denovo14_f0    denovo92_f0    denovo170_f0   denovo196_f0   denovo274_f0   denovo326_f0   denovo378_f0   denovo456_f0   denovo534_f0   denovo690_f0   denovo846_f0   denovo1054_f0  denovo1158_f0  denovo1236_f0  denovo1262_f0  denovo1366_f0  denovo1392_f0  denovo1418_f0  denovo1496_f0  denovo1652_f0  denovo1678_f0  denovo1730_f0  denovo1808_f0  denovo1860_f0  denovo1938_f0  denovo1964_f0  denovo1990_f0  denovo2016_f0  denovo2094_f0  denovo2120_f0  denovo2146_f0  denovo2250_f0  denovo2276_f0  denovo2302_f0  denovo2328_f0  denovo2380_f0  denovo2484_f0  denovo2536_f0  denovo2562_f0  denovo2588_f0  denovo2614_f0  denovo2640_f0  denovo2666_f0  denovo2770_f0  denovo2848_f0  denovo2900_f0  denovo3160_f0  denovo3264_f0  denovo3316_f0  denovo3342_f0  denovo3446_f0  denovo3498_f0  denovo3602_f0  denovo3628_f0  denovo3654_f0  denovo3732_f0  denovo3758_f0  denovo3810_f0  denovo3836_f0  denovo3992_f0  denovo4070_f0  denovo4148_f0  denovo4252_f0  denovo4330_f0  denovo4356_f0  denovo4382_f0  denovo4408_f0  denovo4434_f0  denovo4512_f0  denovo4538_f0  denovo4642_f0  denovo4772_f0  denovo4850_f0  denovo4902_f0  denovo4928_f0  denovo4954_f0  denovo4980_f0  denovo5006_f0  denovo5110_f0  denovo5162_f0  denovo5214_f0  denovo5422_f0  denovo5448_f0  denovo5552_f0  denovo5578_f0  denovo5916_f0  denovo5968_f0  denovo6020_f0  denovo6046_f0  denovo6072_f0  denovo6098_f0  denovo6202_f0  denovo6254_f0  denovo6280_f0  denovo6306_f0  denovo6384_f0  denovo6514_f0  denovo6566_f0  denovo6592_f0  denovo6618_f0  denovo6670_f0  denovo6696_f0  denovo6722_f0  denovo6748_f0  denovo6774_f0  denovo6904_f0  denovo6982_f0  denovo7008_f0  denovo7034_f0  denovo7086_f0  denovo7138_f0  denovo7164_f0  denovo7294_f0  denovo7320_f0  denovo7346_f0  denovo7372_f0  denovo7398_f0  denovo7476_f0  denovo7502_f0  denovo7528_f0  denovo7554_f0  denovo7632_f0  denovo7684_f0  denovo7710_f0  denovo7788_f0  denovo7840_f0  denovo7918_f0  denovo8022_f0  denovo8048_f0  denovo8230_f0  denovo8256_f0  denovo8282_f0  denovo8360_f0  denovo8386_f0  denovo8412_f0  denovo8490_f0  denovo8542_f0  denovo8568_f0  denovo8620_f0  denovo8646_f0  denovo8698_f0  denovo8724_f0  denovo8750_f0  denovo8776_f0  denovo8906_f0  denovo8932_f0  denovo8958_f0  denovo8984_f0  denovo9114_f0  denovo9140_f0  denovo9244_f0  denovo9296_f0  denovo9322_f0  denovo9348_f0  denovo9452_f0  denovo9478_f0  denovo9504_f0  denovo9660_f0  denovo9712_f0  denovo9790_f0  denovo9816_f0  denovo9842_f0  denovo9894_f0  denovo9972_f0  denovo10024_f0 denovo10050_f0 denovo10206_f0 denovo10232_f0 denovo10284_f0 denovo10310_f0 denovo10440_f0 denovo10466_f0 denovo10518_f0 denovo10544_f0 denovo10570_f0 denovo10596_f0 denovo10674_f0 denovo10726_f0 denovo10752_f0 denovo10778_f0 denovo10830_f0 denovo11012_f0 denovo11038_f0 denovo11064_f0 denovo11194_f0 denovo11298_f0 denovo11350_f0 denovo11402_f0 denovo11428_f0 denovo11454_f0 denovo11480_f0 denovo11506_f0 denovo11532_f0 denovo11584_f0 denovo11610_f0 denovo11714_f0 denovo11740_f0 denovo11922_f0 denovo11948_f0 denovo12078_f0 
PXYLO                         denovo18_f0    denovo96_f0    denovo174_f0   denovo200_f0   denovo278_f0   denovo330_f0   denovo382_f0   denovo460_f0   denovo538_f0   denovo694_f0   denovo850_f0   denovo1058_f0  denovo1162_f0  denovo1240_f0  denovo1266_f0  denovo1370_f0  denovo1396_f0  denovo1422_f0  denovo1500_f0  denovo1656_f0  denovo1682_f0  denovo1734_f0  denovo1812_f0  denovo1864_f0  denovo1942_f0  denovo1968_f0  denovo1994_f0  denovo2020_f0  denovo2098_f0  denovo2124_f0  denovo2150_f0  denovo2254_f0  denovo2280_f0  denovo2306_f0  denovo2332_f0  denovo2384_f0  denovo2488_f0  denovo2540_f0  denovo2566_f0  denovo2592_f0  denovo2618_f0  denovo2644_f0  denovo2670_f0  denovo2774_f0  denovo2852_f0  denovo2904_f0  denovo3164_f0  denovo3268_f0  denovo3320_f0  denovo3346_f0  denovo3450_f0  denovo3502_f0  denovo3606_f0  denovo3632_f0  denovo3658_f0  denovo3736_f0  denovo3762_f0  denovo3814_f0  denovo3840_f0  denovo3996_f0  denovo4074_f0  denovo4152_f0  denovo4256_f0  denovo4334_f0  denovo4360_f0  denovo4386_f0  denovo4412_f0  denovo4438_f0  denovo4516_f0  denovo4542_f0  denovo4646_f0  denovo4776_f0  denovo4854_f0  denovo4906_f0  denovo4932_f0  denovo4958_f0  denovo4984_f0  denovo5010_f0  denovo5114_f0  denovo5166_f0  denovo5218_f0  denovo5426_f0  denovo5452_f0  denovo5556_f0  denovo5582_f0  denovo5920_f0  denovo5972_f0  denovo6024_f0  denovo6050_f0  denovo6076_f0  denovo6102_f0  denovo6206_f0  denovo6258_f0  denovo6284_f0  denovo6310_f0  denovo6388_f0  denovo6518_f0  denovo6570_f0  denovo6596_f0  denovo6622_f0  denovo6674_f0  denovo6700_f0  denovo6726_f0  denovo6752_f0  denovo6778_f0  denovo6908_f0  denovo6986_f0  denovo7012_f0  denovo7038_f0  denovo7090_f0  denovo7142_f0  denovo7168_f0  denovo7298_f0  denovo7324_f0  denovo7350_f0  denovo7376_f0  denovo7402_f0  denovo7480_f0  denovo7506_f0  denovo7532_f0  denovo7558_f0  denovo7636_f0  denovo7688_f0  denovo7714_f0  denovo7792_f0  denovo7844_f0  denovo7922_f0  denovo8026_f0  denovo8052_f0  denovo8234_f0  denovo8260_f0  denovo8286_f0  denovo8364_f0  denovo8390_f0  denovo8416_f0  denovo8494_f0  denovo8546_f0  denovo8572_f0  denovo8624_f0  denovo8650_f0  denovo8702_f0  denovo8728_f0  denovo8754_f0  denovo8780_f0  denovo8910_f0  denovo8936_f0  denovo8962_f0  denovo8988_f0  denovo9118_f0  denovo9144_f0  denovo9248_f0  denovo9300_f0  denovo9326_f0  denovo9352_f0  denovo9456_f0  denovo9482_f0  denovo9508_f0  denovo9664_f0  denovo9716_f0  denovo9794_f0  denovo9820_f0  denovo9846_f0  denovo9898_f0  denovo9976_f0  denovo10028_f0 denovo10054_f0 denovo10210_f0 denovo10236_f0 denovo10288_f0 denovo10314_f0 denovo10444_f0 denovo10470_f0 denovo10522_f0 denovo10548_f0 denovo10574_f0 denovo10600_f0 denovo10678_f0 denovo10730_f0 denovo10756_f0 denovo10782_f0 denovo10834_f0 denovo11016_f0 denovo11042_f0 denovo11068_f0 denovo11198_f0 denovo11302_f0 denovo11354_f0 denovo11406_f0 denovo11432_f0 denovo11458_f0 denovo11484_f0 denovo11510_f0 denovo11536_f0 denovo11588_f0 denovo11614_f0 denovo11718_f0 denovo11744_f0 denovo11926_f0 denovo11952_f0 denovo12082_f0 
FG120079                      denovo11_f0    denovo89_f0    denovo167_f0   denovo193_f0   denovo271_f0   denovo323_f0   denovo375_f0   denovo453_f0   denovo531_f0   denovo687_f0   denovo843_f0   denovo1051_f0  denovo1155_f0  denovo1233_f0  denovo1259_f0  denovo1363_f0  denovo1389_f0  denovo1415_f0  denovo1493_f0  denovo1649_f0  denovo1675_f0  denovo1727_f0  denovo1805_f0  denovo1857_f0  denovo1935_f0  denovo1961_f0  denovo1987_f0  denovo2013_f0  denovo2091_f0  denovo2117_f0  denovo2143_f0  denovo2247_f0  denovo2273_f0  denovo2299_f0  denovo2325_f0  denovo2377_f0  denovo2481_f0  denovo2533_f0  denovo2559_f0  denovo2585_f0  denovo2611_f0  denovo2637_f0  denovo2663_f0  denovo2767_f0  denovo2845_f0  denovo2897_f0  denovo3157_f0  denovo3261_f0  denovo3313_f0  denovo3339_f0  denovo3443_f0  denovo3495_f0  denovo3599_f0  denovo3625_f0  denovo3651_f0  denovo3729_f0  denovo3755_f0  denovo3807_f0  denovo3833_f0  denovo3989_f0  denovo4067_f0  denovo4145_f0  denovo4249_f0  denovo4327_f0  denovo4353_f0  denovo4379_f0  denovo4405_f0  denovo4431_f0  denovo4509_f0  denovo4535_f0  denovo4639_f0  denovo4769_f0  denovo4847_f0  denovo4899_f0  denovo4925_f0  denovo4951_f0  denovo4977_f0  denovo5003_f0  denovo5107_f0  denovo5159_f0  denovo5211_f0  denovo5419_f0  denovo5445_f0  denovo5549_f0  denovo5575_f0  denovo5913_f0  denovo5965_f0  denovo6017_f0  denovo6043_f0  denovo6069_f0  denovo6095_f0  denovo6199_f0  denovo6251_f0  denovo6277_f0  denovo6303_f0  denovo6381_f0  denovo6511_f0  denovo6563_f0  denovo6589_f0  denovo6615_f0  denovo6667_f0  denovo6693_f0  denovo6719_f0  denovo6745_f0  denovo6771_f0  denovo6901_f0  denovo6979_f0  denovo7005_f0  denovo7031_f0  denovo7083_f0  denovo7135_f0  denovo7161_f0  denovo7291_f0  denovo7317_f0  denovo7343_f0  denovo7369_f0  denovo7395_f0  denovo7473_f0  denovo7499_f0  denovo7525_f0  denovo7551_f0  denovo7629_f0  denovo7681_f0  denovo7707_f0  denovo7785_f0  denovo7837_f0  denovo7915_f0  denovo8019_f0  denovo8045_f0  denovo8227_f0  denovo8253_f0  denovo8279_f0  denovo8357_f0  denovo8383_f0  denovo8409_f0  denovo8487_f0  denovo8539_f0  denovo8565_f0  denovo8617_f0  denovo8643_f0  denovo8695_f0  denovo8721_f0  denovo8747_f0  denovo8773_f0  denovo8903_f0  denovo8929_f0  denovo8955_f0  denovo8981_f0  denovo9111_f0  denovo9137_f0  denovo9241_f0  denovo9293_f0  denovo9319_f0  denovo9345_f0  denovo9449_f0  denovo9475_f0  denovo9501_f0  denovo9657_f0  denovo9709_f0  denovo9787_f0  denovo9813_f0  denovo9839_f0  denovo9891_f0  denovo9969_f0  denovo10021_f0 denovo10047_f0 denovo10203_f0 denovo10229_f0 denovo10281_f0 denovo10307_f0 denovo10437_f0 denovo10463_f0 denovo10515_f0 denovo10541_f0 denovo10567_f0 denovo10593_f0 denovo10671_f0 denovo10723_f0 denovo10749_f0 denovo10775_f0 denovo10827_f0 denovo11009_f0 denovo11035_f0 denovo11061_f0 denovo11191_f0 denovo11295_f0 denovo11347_f0 denovo11399_f0 denovo11425_f0 denovo11451_f0 denovo11477_f0 denovo11503_f0 denovo11529_f0 denovo11581_f0 denovo11607_f0 denovo11711_f0 denovo11737_f0 denovo11919_f0 denovo11945_f0 denovo12075_f0 
SRR850324                     denovo21_f0    denovo99_f0    denovo177_f0   denovo203_f0   denovo281_f0   denovo333_f0   denovo385_f0   denovo463_f0   denovo541_f0   denovo697_f0   denovo853_f0   denovo1061_f0  denovo1165_f0  denovo1243_f0  denovo1269_f0  denovo1373_f0  denovo1399_f0  denovo1425_f0  denovo1503_f0  denovo1659_f0  denovo1685_f0  denovo1737_f0  denovo1815_f0  denovo1867_f0  denovo1945_f0  denovo1971_f0  denovo1997_f0  denovo2023_f0  denovo2101_f0  denovo2127_f0  denovo2153_f0  denovo2257_f0  denovo2283_f0  denovo2309_f0  denovo2335_f0  denovo2387_f0  denovo2491_f0  denovo2543_f0  denovo2569_f0  denovo2595_f0  denovo2621_f0  denovo2647_f0  denovo2673_f0  denovo2777_f0  denovo2855_f0  denovo2907_f0  denovo3167_f0  denovo3271_f0  denovo3323_f0  denovo3349_f0  denovo3453_f0  denovo3505_f0  denovo3609_f0  denovo3635_f0  denovo3661_f0  denovo3739_f0  denovo3765_f0  denovo3817_f0  denovo3843_f0  denovo3999_f0  denovo4077_f0  denovo4155_f0  denovo4259_f0  denovo4337_f0  denovo4363_f0  denovo4389_f0  denovo4415_f0  denovo4441_f0  denovo4519_f0  denovo4545_f0  denovo4649_f0  denovo4779_f0  denovo4857_f0  denovo4909_f0  denovo4935_f0  denovo4961_f0  denovo4987_f0  denovo5013_f0  denovo5117_f0  denovo5169_f0  denovo5221_f0  denovo5429_f0  denovo5455_f0  denovo5559_f0  denovo5585_f0  denovo5923_f0  denovo5975_f0  denovo6027_f0  denovo6053_f0  denovo6079_f0  denovo6105_f0  denovo6209_f0  denovo6261_f0  denovo6287_f0  denovo6313_f0  denovo6391_f0  denovo6521_f0  denovo6573_f0  denovo6599_f0  denovo6625_f0  denovo6677_f0  denovo6703_f0  denovo6729_f0  denovo6755_f0  denovo6781_f0  denovo6911_f0  denovo6989_f0  denovo7015_f0  denovo7041_f0  denovo7093_f0  denovo7145_f0  denovo7171_f0  denovo7301_f0  denovo7327_f0  denovo7353_f0  denovo7379_f0  denovo7405_f0  denovo7483_f0  denovo7509_f0  denovo7535_f0  denovo7561_f0  denovo7639_f0  denovo7691_f0  denovo7717_f0  denovo7795_f0  denovo7847_f0  denovo7925_f0  denovo8029_f0  denovo8055_f0  denovo8237_f0  denovo8263_f0  denovo8289_f0  denovo8367_f0  denovo8393_f0  denovo8419_f0  denovo8497_f0  denovo8549_f0  denovo8575_f0  denovo8627_f0  denovo8653_f0  denovo8705_f0  denovo8731_f0  denovo8757_f0  denovo8783_f0  denovo8913_f0  denovo8939_f0  denovo8965_f0  denovo8991_f0  denovo9121_f0  denovo9147_f0  denovo9251_f0  denovo9303_f0  denovo9329_f0  denovo9355_f0  denovo9459_f0  denovo9485_f0  denovo9511_f0  denovo9667_f0  denovo9719_f0  denovo9797_f0  denovo9823_f0  denovo9849_f0  denovo9901_f0  denovo9979_f0  denovo10031_f0 denovo10057_f0 denovo10213_f0 denovo10239_f0 denovo10291_f0 denovo10317_f0 denovo10447_f0 denovo10473_f0 denovo10525_f0 denovo10551_f0 denovo10577_f0 denovo10603_f0 denovo10681_f0 denovo10733_f0 denovo10759_f0 denovo10785_f0 denovo10837_f0 denovo11019_f0 denovo11045_f0 denovo11071_f0 denovo11201_f0 denovo11305_f0 denovo11357_f0 denovo11409_f0 denovo11435_f0 denovo11461_f0 denovo11487_f0 denovo11513_f0 denovo11539_f0 denovo11591_f0 denovo11617_f0 denovo11721_f0 denovo11747_f0 denovo11929_f0 denovo11955_f0 denovo12085_f0 
FG120055B                     denovo7_f0     denovo85_f0    denovo163_f0   denovo189_f0   denovo267_f0   denovo319_f0   denovo371_f0   denovo449_f0   denovo527_f0   denovo683_f0   denovo839_f0   denovo1047_f0  denovo1151_f0  denovo1229_f0  denovo1255_f0  denovo1359_f0  denovo1385_f0  denovo1411_f0  denovo1489_f0  denovo1645_f0  denovo1671_f0  denovo1723_f0  denovo1801_f0  denovo1853_f0  denovo1931_f0  denovo1957_f0  denovo1983_f0  denovo2009_f0  denovo2087_f0  denovo2113_f0  denovo2139_f0  denovo2243_f0  denovo2269_f0  denovo2295_f0  denovo2321_f0  denovo2373_f0  denovo2477_f0  denovo2529_f0  denovo2555_f0  denovo2581_f0  denovo2607_f0  denovo2633_f0  denovo2659_f0  denovo2763_f0  denovo2841_f0  denovo2893_f0  denovo3153_f0  denovo3257_f0  denovo3309_f0  denovo3335_f0  denovo3439_f0  denovo3491_f0  denovo3595_f0  denovo3621_f0  denovo3647_f0  denovo3725_f0  denovo3751_f0  denovo3803_f0  denovo3829_f0  denovo3985_f0  denovo4063_f0  denovo4141_f0  denovo4245_f0  denovo4323_f0  denovo4349_f0  denovo4375_f0  denovo4401_f0  denovo4427_f0  denovo4505_f0  denovo4531_f0  denovo4635_f0  denovo4765_f0  denovo4843_f0  denovo4895_f0  denovo4921_f0  denovo4947_f0  denovo4973_f0  denovo4999_f0  denovo5103_f0  denovo5155_f0  denovo5207_f0  denovo5415_f0  denovo5441_f0  denovo5545_f0  denovo5571_f0  denovo5909_f0  denovo5961_f0  denovo6013_f0  denovo6039_f0  denovo6065_f0  denovo6091_f0  denovo6195_f0  denovo6247_f0  denovo6273_f0  denovo6299_f0  denovo6377_f0  denovo6507_f0  denovo6559_f0  denovo6585_f0  denovo6611_f0  denovo6663_f0  denovo6689_f0  denovo6715_f0  denovo6741_f0  denovo6767_f0  denovo6897_f0  denovo6975_f0  denovo7001_f0  denovo7027_f0  denovo7079_f0  denovo7131_f0  denovo7157_f0  denovo7287_f0  denovo7313_f0  denovo7339_f0  denovo7365_f0  denovo7391_f0  denovo7469_f0  denovo7495_f0  denovo7521_f0  denovo7547_f0  denovo7625_f0  denovo7677_f0  denovo7703_f0  denovo7781_f0  denovo7833_f0  denovo7911_f0  denovo8015_f0  denovo8041_f0  denovo8223_f0  denovo8249_f0  denovo8275_f0  denovo8353_f0  denovo8379_f0  denovo8405_f0  denovo8483_f0  denovo8535_f0  denovo8561_f0  denovo8613_f0  denovo8639_f0  denovo8691_f0  denovo8717_f0  denovo8743_f0  denovo8769_f0  denovo8899_f0  denovo8925_f0  denovo8951_f0  denovo8977_f0  denovo9107_f0  denovo9133_f0  denovo9237_f0  denovo9289_f0  denovo9315_f0  denovo9341_f0  denovo9445_f0  denovo9471_f0  denovo9497_f0  denovo9653_f0  denovo9705_f0  denovo9783_f0  denovo9809_f0  denovo9835_f0  denovo9887_f0  denovo9965_f0  denovo10017_f0 denovo10043_f0 denovo10199_f0 denovo10225_f0 denovo10277_f0 denovo10303_f0 denovo10433_f0 denovo10459_f0 denovo10511_f0 denovo10537_f0 denovo10563_f0 denovo10589_f0 denovo10667_f0 denovo10719_f0 denovo10745_f0 denovo10771_f0 denovo10823_f0 denovo11005_f0 denovo11031_f0 denovo11057_f0 denovo11187_f0 denovo11291_f0 denovo11343_f0 denovo11395_f0 denovo11421_f0 denovo11447_f0 denovo11473_f0 denovo11499_f0 denovo11525_f0 denovo11577_f0 denovo11603_f0 denovo11707_f0 denovo11733_f0 denovo11915_f0 denovo11941_f0 denovo12071_f0 
FG120122                      denovo12_f0    denovo90_f0    denovo168_f0   denovo194_f0   denovo272_f0   denovo324_f0   denovo376_f0   denovo454_f0   denovo532_f0   denovo688_f0   denovo844_f0   denovo1052_f0  denovo1156_f0  denovo1234_f0  denovo1260_f0  denovo1364_f0  denovo1390_f0  denovo1416_f0  denovo1494_f0  denovo1650_f0  denovo1676_f0  denovo1728_f0  denovo1806_f0  denovo1858_f0  denovo1936_f0  denovo1962_f0  denovo1988_f0  denovo2014_f0  denovo2092_f0  denovo2118_f0  denovo2144_f0  denovo2248_f0  denovo2274_f0  denovo2300_f0  denovo2326_f0  denovo2378_f0  denovo2482_f0  denovo2534_f0  denovo2560_f0  denovo2586_f0  denovo2612_f0  denovo2638_f0  denovo2664_f0  denovo2768_f0  denovo2846_f0  denovo2898_f0  denovo3158_f0  denovo3262_f0  denovo3314_f0  denovo3340_f0  denovo3444_f0  denovo3496_f0  denovo3600_f0  denovo3626_f0  denovo3652_f0  denovo3730_f0  denovo3756_f0  denovo3808_f0  denovo3834_f0  denovo3990_f0  denovo4068_f0  denovo4146_f0  denovo4250_f0  denovo4328_f0  denovo4354_f0  denovo4380_f0  denovo4406_f0  denovo4432_f0  denovo4510_f0  denovo4536_f0  denovo4640_f0  denovo4770_f0  denovo4848_f0  denovo4900_f0  denovo4926_f0  denovo4952_f0  denovo4978_f0  denovo5004_f0  denovo5108_f0  denovo5160_f0  denovo5212_f0  denovo5420_f0  denovo5446_f0  denovo5550_f0  denovo5576_f0  denovo5914_f0  denovo5966_f0  denovo6018_f0  denovo6044_f0  denovo6070_f0  denovo6096_f0  denovo6200_f0  denovo6252_f0  denovo6278_f0  denovo6304_f0  denovo6382_f0  denovo6512_f0  denovo6564_f0  denovo6590_f0  denovo6616_f0  denovo6668_f0  denovo6694_f0  denovo6720_f0  denovo6746_f0  denovo6772_f0  denovo6902_f0  denovo6980_f0  denovo7006_f0  denovo7032_f0  denovo7084_f0  denovo7136_f0  denovo7162_f0  denovo7292_f0  denovo7318_f0  denovo7344_f0  denovo7370_f0  denovo7396_f0  denovo7474_f0  denovo7500_f0  denovo7526_f0  denovo7552_f0  denovo7630_f0  denovo7682_f0  denovo7708_f0  denovo7786_f0  denovo7838_f0  denovo7916_f0  denovo8020_f0  denovo8046_f0  denovo8228_f0  denovo8254_f0  denovo8280_f0  denovo8358_f0  denovo8384_f0  denovo8410_f0  denovo8488_f0  denovo8540_f0  denovo8566_f0  denovo8618_f0  denovo8644_f0  denovo8696_f0  denovo8722_f0  denovo8748_f0  denovo8774_f0  denovo8904_f0  denovo8930_f0  denovo8956_f0  denovo8982_f0  denovo9112_f0  denovo9138_f0  denovo9242_f0  denovo9294_f0  denovo9320_f0  denovo9346_f0  denovo9450_f0  denovo9476_f0  denovo9502_f0  denovo9658_f0  denovo9710_f0  denovo9788_f0  denovo9814_f0  denovo9840_f0  denovo9892_f0  denovo9970_f0  denovo10022_f0 denovo10048_f0 denovo10204_f0 denovo10230_f0 denovo10282_f0 denovo10308_f0 denovo10438_f0 denovo10464_f0 denovo10516_f0 denovo10542_f0 denovo10568_f0 denovo10594_f0 denovo10672_f0 denovo10724_f0 denovo10750_f0 denovo10776_f0 denovo10828_f0 denovo11010_f0 denovo11036_f0 denovo11062_f0 denovo11192_f0 denovo11296_f0 denovo11348_f0 denovo11400_f0 denovo11426_f0 denovo11452_f0 denovo11478_f0 denovo11504_f0 denovo11530_f0 denovo11582_f0 denovo11608_f0 denovo11712_f0 denovo11738_f0 denovo11920_f0 denovo11946_f0 denovo12076_f0 
FG120024                      denovo4_f0     denovo82_f0    denovo160_f0   denovo186_f0   denovo264_f0   denovo316_f0   denovo368_f0   denovo446_f0   denovo524_f0   denovo680_f0   denovo836_f0   denovo1044_f0  denovo1148_f0  denovo1226_f0  denovo1252_f0  denovo1356_f0  denovo1382_f0  denovo1408_f0  denovo1486_f0  denovo1642_f0  denovo1668_f0  denovo1720_f0  denovo1798_f0  denovo1850_f0  denovo1928_f0  denovo1954_f0  denovo1980_f0  denovo2006_f0  denovo2084_f0  denovo2110_f0  denovo2136_f0  denovo2240_f0  denovo2266_f0  denovo2292_f0  denovo2318_f0  denovo2370_f0  denovo2474_f0  denovo2526_f0  denovo2552_f0  denovo2578_f0  denovo2604_f0  denovo2630_f0  denovo2656_f0  denovo2760_f0  denovo2838_f0  denovo2890_f0  denovo3150_f0  denovo3254_f0  denovo3306_f0  denovo3332_f0  denovo3436_f0  denovo3488_f0  denovo3592_f0  denovo3618_f0  denovo3644_f0  denovo3722_f0  denovo3748_f0  denovo3800_f0  denovo3826_f0  denovo3982_f0  denovo4060_f0  denovo4138_f0  denovo4242_f0  denovo4320_f0  denovo4346_f0  denovo4372_f0  denovo4398_f0  denovo4424_f0  denovo4502_f0  denovo4528_f0  denovo4632_f0  denovo4762_f0  denovo4840_f0  denovo4892_f0  denovo4918_f0  denovo4944_f0  denovo4970_f0  denovo4996_f0  denovo5100_f0  denovo5152_f0  denovo5204_f0  denovo5412_f0  denovo5438_f0  denovo5542_f0  denovo5568_f0  denovo5906_f0  denovo5958_f0  denovo6010_f0  denovo6036_f0  denovo6062_f0  denovo6088_f0  denovo6192_f0  denovo6244_f0  denovo6270_f0  denovo6296_f0  denovo6374_f0  denovo6504_f0  denovo6556_f0  denovo6582_f0  denovo6608_f0  denovo6660_f0  denovo6686_f0  denovo6712_f0  denovo6738_f0  denovo6764_f0  denovo6894_f0  denovo6972_f0  denovo6998_f0  denovo7024_f0  denovo7076_f0  denovo7128_f0  denovo7154_f0  denovo7284_f0  denovo7310_f0  denovo7336_f0  denovo7362_f0  denovo7388_f0  denovo7466_f0  denovo7492_f0  denovo7518_f0  denovo7544_f0  denovo7622_f0  denovo7674_f0  denovo7700_f0  denovo7778_f0  denovo7830_f0  denovo7908_f0  denovo8012_f0  denovo8038_f0  denovo8220_f0  denovo8246_f0  denovo8272_f0  denovo8350_f0  denovo8376_f0  denovo8402_f0  denovo8480_f0  denovo8532_f0  denovo8558_f0  denovo8610_f0  denovo8636_f0  denovo8688_f0  denovo8714_f0  denovo8740_f0  denovo8766_f0  denovo8896_f0  denovo8922_f0  denovo8948_f0  denovo8974_f0  denovo9104_f0  denovo9130_f0  denovo9234_f0  denovo9286_f0  denovo9312_f0  denovo9338_f0  denovo9442_f0  denovo9468_f0  denovo9494_f0  denovo9650_f0  denovo9702_f0  denovo9780_f0  denovo9806_f0  denovo9832_f0  denovo9884_f0  denovo9962_f0  denovo10014_f0 denovo10040_f0 denovo10196_f0 denovo10222_f0 denovo10274_f0 denovo10300_f0 denovo10430_f0 denovo10456_f0 denovo10508_f0 denovo10534_f0 denovo10560_f0 denovo10586_f0 denovo10664_f0 denovo10716_f0 denovo10742_f0 denovo10768_f0 denovo10820_f0 denovo11002_f0 denovo11028_f0 denovo11054_f0 denovo11184_f0 denovo11288_f0 denovo11340_f0 denovo11392_f0 denovo11418_f0 denovo11444_f0 denovo11470_f0 denovo11496_f0 denovo11522_f0 denovo11574_f0 denovo11600_f0 denovo11704_f0 denovo11730_f0 denovo11912_f0 denovo11938_f0 denovo12068_f0 
FG120022                      denovo3_f0     denovo81_f0    denovo159_f0   denovo185_f0   denovo263_f0   denovo315_f0   denovo367_f0   denovo445_f0   denovo523_f0   denovo679_f0   denovo835_f0   denovo1043_f0  denovo1147_f0  denovo1225_f0  denovo1251_f0  denovo1355_f0  denovo1381_f0  denovo1407_f0  denovo1485_f0  denovo1641_f0  denovo1667_f0  denovo1719_f0  denovo1797_f0  denovo1849_f0  denovo1927_f0  denovo1953_f0  denovo1979_f0  denovo2005_f0  denovo2083_f0  denovo2109_f0  denovo2135_f0  denovo2239_f0  denovo2265_f0  denovo2291_f0  denovo2317_f0  denovo2369_f0  denovo2473_f0  denovo2525_f0  denovo2551_f0  denovo2577_f0  denovo2603_f0  denovo2629_f0  denovo2655_f0  denovo2759_f0  denovo2837_f0  denovo2889_f0  denovo3149_f0  denovo3253_f0  denovo3305_f0  denovo3331_f0  denovo3435_f0  denovo3487_f0  denovo3591_f0  denovo3617_f0  denovo3643_f0  denovo3721_f0  denovo3747_f0  denovo3799_f0  denovo3825_f0  denovo3981_f0  denovo4059_f0  denovo4137_f0  denovo4241_f0  denovo4319_f0  denovo4345_f0  denovo4371_f0  denovo4397_f0  denovo4423_f0  denovo4501_f0  denovo4527_f0  denovo4631_f0  denovo4761_f0  denovo4839_f0  denovo4891_f0  denovo4917_f0  denovo4943_f0  denovo4969_f0  denovo4995_f0  denovo5099_f0  denovo5151_f0  denovo5203_f0  denovo5411_f0  denovo5437_f0  denovo5541_f0  denovo5567_f0  denovo5905_f0  denovo5957_f0  denovo6009_f0  denovo6035_f0  denovo6061_f0  denovo6087_f0  denovo6191_f0  denovo6243_f0  denovo6269_f0  denovo6295_f0  denovo6373_f0  denovo6503_f0  denovo6555_f0  denovo6581_f0  denovo6607_f0  denovo6659_f0  denovo6685_f0  denovo6711_f0  denovo6737_f0  denovo6763_f0  denovo6893_f0  denovo6971_f0  denovo6997_f0  denovo7023_f0  denovo7075_f0  denovo7127_f0  denovo7153_f0  denovo7283_f0  denovo7309_f0  denovo7335_f0  denovo7361_f0  denovo7387_f0  denovo7465_f0  denovo7491_f0  denovo7517_f0  denovo7543_f0  denovo7621_f0  denovo7673_f0  denovo7699_f0  denovo7777_f0  denovo7829_f0  denovo7907_f0  denovo8011_f0  denovo8037_f0  denovo8219_f0  denovo8245_f0  denovo8271_f0  denovo8349_f0  denovo8375_f0  denovo8401_f0  denovo8479_f0  denovo8531_f0  denovo8557_f0  denovo8609_f0  denovo8635_f0  denovo8687_f0  denovo8713_f0  denovo8739_f0  denovo8765_f0  denovo8895_f0  denovo8921_f0  denovo8947_f0  denovo8973_f0  denovo9103_f0  denovo9129_f0  denovo9233_f0  denovo9285_f0  denovo9311_f0  denovo9337_f0  denovo9441_f0  denovo9467_f0  denovo9493_f0  denovo9649_f0  denovo9701_f0  denovo9779_f0  denovo9805_f0  denovo9831_f0  denovo9883_f0  denovo9961_f0  denovo10013_f0 denovo10039_f0 denovo10195_f0 denovo10221_f0 denovo10273_f0 denovo10299_f0 denovo10429_f0 denovo10455_f0 denovo10507_f0 denovo10533_f0 denovo10559_f0 denovo10585_f0 denovo10663_f0 denovo10715_f0 denovo10741_f0 denovo10767_f0 denovo10819_f0 denovo11001_f0 denovo11027_f0 denovo11053_f0 denovo11183_f0 denovo11287_f0 denovo11339_f0 denovo11391_f0 denovo11417_f0 denovo11443_f0 denovo11469_f0 denovo11495_f0 denovo11521_f0 denovo11573_f0 denovo11599_f0 denovo11703_f0 denovo11729_f0 denovo11911_f0 denovo11937_f0 denovo12067_f0 
GNV139000                     denovo16_f0    denovo94_f0    denovo172_f0   denovo198_f0   denovo276_f0   denovo328_f0   denovo380_f0   denovo458_f0   denovo536_f0   denovo692_f0   denovo848_f0   denovo1056_f0  denovo1160_f0  denovo1238_f0  denovo1264_f0  denovo1368_f0  denovo1394_f0  denovo1420_f0  denovo1498_f0  denovo1654_f0  denovo1680_f0  denovo1732_f0  denovo1810_f0  denovo1862_f0  denovo1940_f0  denovo1966_f0  denovo1992_f0  denovo2018_f0  denovo2096_f0  denovo2122_f0  denovo2148_f0  denovo2252_f0  denovo2278_f0  denovo2304_f0  denovo2330_f0  denovo2382_f0  denovo2486_f0  denovo2538_f0  denovo2564_f0  denovo2590_f0  denovo2616_f0  denovo2642_f0  denovo2668_f0  denovo2772_f0  denovo2850_f0  denovo2902_f0  denovo3162_f0  denovo3266_f0  denovo3318_f0  denovo3344_f0  denovo3448_f0  denovo3500_f0  denovo3604_f0  denovo3630_f0  denovo3656_f0  denovo3734_f0  denovo3760_f0  denovo3812_f0  denovo3838_f0  denovo3994_f0  denovo4072_f0  denovo4150_f0  denovo4254_f0  denovo4332_f0  denovo4358_f0  denovo4384_f0  denovo4410_f0  denovo4436_f0  denovo4514_f0  denovo4540_f0  denovo4644_f0  denovo4774_f0  denovo4852_f0  denovo4904_f0  denovo4930_f0  denovo4956_f0  denovo4982_f0  denovo5008_f0  denovo5112_f0  denovo5164_f0  denovo5216_f0  denovo5424_f0  denovo5450_f0  denovo5554_f0  denovo5580_f0  denovo5918_f0  denovo5970_f0  denovo6022_f0  denovo6048_f0  denovo6074_f0  denovo6100_f0  denovo6204_f0  denovo6256_f0  denovo6282_f0  denovo6308_f0  denovo6386_f0  denovo6516_f0  denovo6568_f0  denovo6594_f0  denovo6620_f0  denovo6672_f0  denovo6698_f0  denovo6724_f0  denovo6750_f0  denovo6776_f0  denovo6906_f0  denovo6984_f0  denovo7010_f0  denovo7036_f0  denovo7088_f0  denovo7140_f0  denovo7166_f0  denovo7296_f0  denovo7322_f0  denovo7348_f0  denovo7374_f0  denovo7400_f0  denovo7478_f0  denovo7504_f0  denovo7530_f0  denovo7556_f0  denovo7634_f0  denovo7686_f0  denovo7712_f0  denovo7790_f0  denovo7842_f0  denovo7920_f0  denovo8024_f0  denovo8050_f0  denovo8232_f0  denovo8258_f0  denovo8284_f0  denovo8362_f0  denovo8388_f0  denovo8414_f0  denovo8492_f0  denovo8544_f0  denovo8570_f0  denovo8622_f0  denovo8648_f0  denovo8700_f0  denovo8726_f0  denovo8752_f0  denovo8778_f0  denovo8908_f0  denovo8934_f0  denovo8960_f0  denovo8986_f0  denovo9116_f0  denovo9142_f0  denovo9246_f0  denovo9298_f0  denovo9324_f0  denovo9350_f0  denovo9454_f0  denovo9480_f0  denovo9506_f0  denovo9662_f0  denovo9714_f0  denovo9792_f0  denovo9818_f0  denovo9844_f0  denovo9896_f0  denovo9974_f0  denovo10026_f0 denovo10052_f0 denovo10208_f0 denovo10234_f0 denovo10286_f0 denovo10312_f0 denovo10442_f0 denovo10468_f0 denovo10520_f0 denovo10546_f0 denovo10572_f0 denovo10598_f0 denovo10676_f0 denovo10728_f0 denovo10754_f0 denovo10780_f0 denovo10832_f0 denovo11014_f0 denovo11040_f0 denovo11066_f0 denovo11196_f0 denovo11300_f0 denovo11352_f0 denovo11404_f0 denovo11430_f0 denovo11456_f0 denovo11482_f0 denovo11508_f0 denovo11534_f0 denovo11586_f0 denovo11612_f0 denovo11716_f0 denovo11742_f0 denovo11924_f0 denovo11950_f0 denovo12080_f0 
Msexta                        denovo17_f0    denovo95_f0    denovo173_f0   denovo199_f0   denovo277_f0   denovo329_f0   denovo381_f0   denovo459_f0   denovo537_f0   denovo693_f0   denovo849_f0   denovo1057_f0  denovo1161_f0  denovo1239_f0  denovo1265_f0  denovo1369_f0  denovo1395_f0  denovo1421_f0  denovo1499_f0  denovo1655_f0  denovo1681_f0  denovo1733_f0  denovo1811_f0  denovo1863_f0  denovo1941_f0  denovo1967_f0  denovo1993_f0  denovo2019_f0  denovo2097_f0  denovo2123_f0  denovo2149_f0  denovo2253_f0  denovo2279_f0  denovo2305_f0  denovo2331_f0  denovo2383_f0  denovo2487_f0  denovo2539_f0  denovo2565_f0  denovo2591_f0  denovo2617_f0  denovo2643_f0  denovo2669_f0  denovo2773_f0  denovo2851_f0  denovo2903_f0  denovo3163_f0  denovo3267_f0  denovo3319_f0  denovo3345_f0  denovo3449_f0  denovo3501_f0  denovo3605_f0  denovo3631_f0  denovo3657_f0  denovo3735_f0  denovo3761_f0  denovo3813_f0  denovo3839_f0  denovo3995_f0  denovo4073_f0  denovo4151_f0  denovo4255_f0  denovo4333_f0  denovo4359_f0  denovo4385_f0  denovo4411_f0  denovo4437_f0  denovo4515_f0  denovo4541_f0  denovo4645_f0  denovo4775_f0  denovo4853_f0  denovo4905_f0  denovo4931_f0  denovo4957_f0  denovo4983_f0  denovo5009_f0  denovo5113_f0  denovo5165_f0  denovo5217_f0  denovo5425_f0  denovo5451_f0  denovo5555_f0  denovo5581_f0  denovo5919_f0  denovo5971_f0  denovo6023_f0  denovo6049_f0  denovo6075_f0  denovo6101_f0  denovo6205_f0  denovo6257_f0  denovo6283_f0  denovo6309_f0  denovo6387_f0  denovo6517_f0  denovo6569_f0  denovo6595_f0  denovo6621_f0  denovo6673_f0  denovo6699_f0  denovo6725_f0  denovo6751_f0  denovo6777_f0  denovo6907_f0  denovo6985_f0  denovo7011_f0  denovo7037_f0  denovo7089_f0  denovo7141_f0  denovo7167_f0  denovo7297_f0  denovo7323_f0  denovo7349_f0  denovo7375_f0  denovo7401_f0  denovo7479_f0  denovo7505_f0  denovo7531_f0  denovo7557_f0  denovo7635_f0  denovo7687_f0  denovo7713_f0  denovo7791_f0  denovo7843_f0  denovo7921_f0  denovo8025_f0  denovo8051_f0  denovo8233_f0  denovo8259_f0  denovo8285_f0  denovo8363_f0  denovo8389_f0  denovo8415_f0  denovo8493_f0  denovo8545_f0  denovo8571_f0  denovo8623_f0  denovo8649_f0  denovo8701_f0  denovo8727_f0  denovo8753_f0  denovo8779_f0  denovo8909_f0  denovo8935_f0  denovo8961_f0  denovo8987_f0  denovo9117_f0  denovo9143_f0  denovo9247_f0  denovo9299_f0  denovo9325_f0  denovo9351_f0  denovo9455_f0  denovo9481_f0  denovo9507_f0  denovo9663_f0  denovo9715_f0  denovo9793_f0  denovo9819_f0  denovo9845_f0  denovo9897_f0  denovo9975_f0  denovo10027_f0 denovo10053_f0 denovo10209_f0 denovo10235_f0 denovo10287_f0 denovo10313_f0 denovo10443_f0 denovo10469_f0 denovo10521_f0 denovo10547_f0 denovo10573_f0 denovo10599_f0 denovo10677_f0 denovo10729_f0 denovo10755_f0 denovo10781_f0 denovo10833_f0 denovo11015_f0 denovo11041_f0 denovo11067_f0 denovo11197_f0 denovo11301_f0 denovo11353_f0 denovo11405_f0 denovo11431_f0 denovo11457_f0 denovo11483_f0 denovo11509_f0 denovo11535_f0 denovo11587_f0 denovo11613_f0 denovo11717_f0 denovo11743_f0 denovo11925_f0 denovo11951_f0 denovo12081_f0 
acti2                         denovo25_f0    denovo103_f0   denovo181_f0   denovo207_f0   denovo285_f0   denovo337_f0   denovo389_f0   denovo467_f0   denovo545_f0   denovo701_f0   denovo857_f0   denovo1065_f0  denovo1169_f0  denovo1247_f0  denovo1273_f0  denovo1377_f0  denovo1403_f0  denovo1429_f0  denovo1507_f0  denovo1663_f0  denovo1689_f0  denovo1741_f0  denovo1819_f0  denovo1871_f0  denovo1949_f0  denovo1975_f0  denovo2001_f0  denovo2027_f0  denovo2105_f0  denovo2131_f0  denovo2157_f0  denovo2261_f0  denovo2287_f0  denovo2313_f0  denovo2339_f0  denovo2391_f0  denovo2495_f0  denovo2547_f0  denovo2573_f0  denovo2599_f0  denovo2625_f0  denovo2651_f0  denovo2677_f0  denovo2781_f0  denovo2859_f0  denovo2911_f0  denovo3171_f0  denovo3275_f0  denovo3327_f0  denovo3353_f0  denovo3457_f0  denovo3509_f0  denovo3613_f0  denovo3639_f0  denovo3665_f0  denovo3743_f0  denovo3769_f0  denovo3821_f0  denovo3847_f0  denovo4003_f0  denovo4081_f0  denovo4159_f0  denovo4263_f0  denovo4341_f0  denovo4367_f0  denovo4393_f0  denovo4419_f0  denovo4445_f0  denovo4523_f0  denovo4549_f0  denovo4653_f0  denovo4783_f0  denovo4861_f0  denovo4913_f0  denovo4939_f0  denovo4965_f0  denovo4991_f0  denovo5017_f0  denovo5121_f0  denovo5173_f0  denovo5225_f0  denovo5433_f0  denovo5459_f0  denovo5563_f0  denovo5589_f0  denovo5927_f0  denovo5979_f0  denovo6031_f0  denovo6057_f0  denovo6083_f0  denovo6109_f0  denovo6213_f0  denovo6265_f0  denovo6291_f0  denovo6317_f0  denovo6395_f0  denovo6525_f0  denovo6577_f0  denovo6603_f0  denovo6629_f0  denovo6681_f0  denovo6707_f0  denovo6733_f0  denovo6759_f0  denovo6785_f0  denovo6915_f0  denovo6993_f0  denovo7019_f0  denovo7045_f0  denovo7097_f0  denovo7149_f0  denovo7175_f0  denovo7305_f0  denovo7331_f0  denovo7357_f0  denovo7383_f0  denovo7409_f0  denovo7487_f0  denovo7513_f0  denovo7539_f0  denovo7565_f0  denovo7643_f0  denovo7695_f0  denovo7721_f0  denovo7799_f0  denovo7851_f0  denovo7929_f0  denovo8033_f0  denovo8059_f0  denovo8241_f0  denovo8267_f0  denovo8293_f0  denovo8371_f0  denovo8397_f0  denovo8423_f0  denovo8501_f0  denovo8553_f0  denovo8579_f0  denovo8631_f0  denovo8657_f0  denovo8709_f0  denovo8735_f0  denovo8761_f0  denovo8787_f0  denovo8917_f0  denovo8943_f0  denovo8969_f0  denovo8995_f0  denovo9125_f0  denovo9151_f0  denovo9255_f0  denovo9307_f0  denovo9333_f0  denovo9359_f0  denovo9463_f0  denovo9489_f0  denovo9515_f0  denovo9671_f0  denovo9723_f0  denovo9801_f0  denovo9827_f0  denovo9853_f0  denovo9905_f0  denovo9983_f0  denovo10035_f0 denovo10061_f0 denovo10217_f0 denovo10243_f0 denovo10295_f0 denovo10321_f0 denovo10451_f0 denovo10477_f0 denovo10529_f0 denovo10555_f0 denovo10581_f0 denovo10607_f0 denovo10685_f0 denovo10737_f0 denovo10763_f0 denovo10789_f0 denovo10841_f0 denovo11023_f0 denovo11049_f0 denovo11075_f0 denovo11205_f0 denovo11309_f0 denovo11361_f0 denovo11413_f0 denovo11439_f0 denovo11465_f0 denovo11491_f0 denovo11517_f0 denovo11543_f0 denovo11595_f0 denovo11621_f0 denovo11725_f0 denovo11751_f0 denovo11933_f0 denovo11959_f0 denovo12089_f0 
FG120071B                     denovo9_f0     denovo87_f0    denovo165_f0   denovo191_f0   denovo269_f0   denovo321_f0   denovo373_f0   denovo451_f0   denovo529_f0   denovo685_f0   denovo841_f0   denovo1049_f0  denovo1153_f0  denovo1231_f0  denovo1257_f0  denovo1361_f0  denovo1387_f0  denovo1413_f0  denovo1491_f0  denovo1647_f0  denovo1673_f0  denovo1725_f0  denovo1803_f0  denovo1855_f0  denovo1933_f0  denovo1959_f0  denovo1985_f0  denovo2011_f0  denovo2089_f0  denovo2115_f0  denovo2141_f0  denovo2245_f0  denovo2271_f0  denovo2297_f0  denovo2323_f0  denovo2375_f0  denovo2479_f0  denovo2531_f0  denovo2557_f0  denovo2583_f0  denovo2609_f0  denovo2635_f0  denovo2661_f0  denovo2765_f0  denovo2843_f0  denovo2895_f0  denovo3155_f0  denovo3259_f0  denovo3311_f0  denovo3337_f0  denovo3441_f0  denovo3493_f0  denovo3597_f0  denovo3623_f0  denovo3649_f0  denovo3727_f0  denovo3753_f0  denovo3805_f0  denovo3831_f0  denovo3987_f0  denovo4065_f0  denovo4143_f0  denovo4247_f0  denovo4325_f0  denovo4351_f0  denovo4377_f0  denovo4403_f0  denovo4429_f0  denovo4507_f0  denovo4533_f0  denovo4637_f0  denovo4767_f0  denovo4845_f0  denovo4897_f0  denovo4923_f0  denovo4949_f0  denovo4975_f0  denovo5001_f0  denovo5105_f0  denovo5157_f0  denovo5209_f0  denovo5417_f0  denovo5443_f0  denovo5547_f0  denovo5573_f0  denovo5911_f0  denovo5963_f0  denovo6015_f0  denovo6041_f0  denovo6067_f0  denovo6093_f0  denovo6197_f0  denovo6249_f0  denovo6275_f0  denovo6301_f0  denovo6379_f0  denovo6509_f0  denovo6561_f0  denovo6587_f0  denovo6613_f0  denovo6665_f0  denovo6691_f0  denovo6717_f0  denovo6743_f0  denovo6769_f0  denovo6899_f0  denovo6977_f0  denovo7003_f0  denovo7029_f0  denovo7081_f0  denovo7133_f0  denovo7159_f0  denovo7289_f0  denovo7315_f0  denovo7341_f0  denovo7367_f0  denovo7393_f0  denovo7471_f0  denovo7497_f0  denovo7523_f0  denovo7549_f0  denovo7627_f0  denovo7679_f0  denovo7705_f0  denovo7783_f0  denovo7835_f0  denovo7913_f0  denovo8017_f0  denovo8043_f0  denovo8225_f0  denovo8251_f0  denovo8277_f0  denovo8355_f0  denovo8381_f0  denovo8407_f0  denovo8485_f0  denovo8537_f0  denovo8563_f0  denovo8615_f0  denovo8641_f0  denovo8693_f0  denovo8719_f0  denovo8745_f0  denovo8771_f0  denovo8901_f0  denovo8927_f0  denovo8953_f0  denovo8979_f0  denovo9109_f0  denovo9135_f0  denovo9239_f0  denovo9291_f0  denovo9317_f0  denovo9343_f0  denovo9447_f0  denovo9473_f0  denovo9499_f0  denovo9655_f0  denovo9707_f0  denovo9785_f0  denovo9811_f0  denovo9837_f0  denovo9889_f0  denovo9967_f0  denovo10019_f0 denovo10045_f0 denovo10201_f0 denovo10227_f0 denovo10279_f0 denovo10305_f0 denovo10435_f0 denovo10461_f0 denovo10513_f0 denovo10539_f0 denovo10565_f0 denovo10591_f0 denovo10669_f0 denovo10721_f0 denovo10747_f0 denovo10773_f0 denovo10825_f0 denovo11007_f0 denovo11033_f0 denovo11059_f0 denovo11189_f0 denovo11293_f0 denovo11345_f0 denovo11397_f0 denovo11423_f0 denovo11449_f0 denovo11475_f0 denovo11501_f0 denovo11527_f0 denovo11579_f0 denovo11605_f0 denovo11709_f0 denovo11735_f0 denovo11917_f0 denovo11943_f0 denovo12073_f0 
GNV120027                     denovo13_f0    denovo91_f0    denovo169_f0   denovo195_f0   denovo273_f0   denovo325_f0   denovo377_f0   denovo455_f0   denovo533_f0   denovo689_f0   denovo845_f0   denovo1053_f0  denovo1157_f0  denovo1235_f0  denovo1261_f0  denovo1365_f0  denovo1391_f0  denovo1417_f0  denovo1495_f0  denovo1651_f0  denovo1677_f0  denovo1729_f0  denovo1807_f0  denovo1859_f0  denovo1937_f0  denovo1963_f0  denovo1989_f0  denovo2015_f0  denovo2093_f0  denovo2119_f0  denovo2145_f0  denovo2249_f0  denovo2275_f0  denovo2301_f0  denovo2327_f0  denovo2379_f0  denovo2483_f0  denovo2535_f0  denovo2561_f0  denovo2587_f0  denovo2613_f0  denovo2639_f0  denovo2665_f0  denovo2769_f0  denovo2847_f0  denovo2899_f0  denovo3159_f0  denovo3263_f0  denovo3315_f0  denovo3341_f0  denovo3445_f0  denovo3497_f0  denovo3601_f0  denovo3627_f0  denovo3653_f0  denovo3731_f0  denovo3757_f0  denovo3809_f0  denovo3835_f0  denovo3991_f0  denovo4069_f0  denovo4147_f0  denovo4251_f0  denovo4329_f0  denovo4355_f0  denovo4381_f0  denovo4407_f0  denovo4433_f0  denovo4511_f0  denovo4537_f0  denovo4641_f0  denovo4771_f0  denovo4849_f0  denovo4901_f0  denovo4927_f0  denovo4953_f0  denovo4979_f0  denovo5005_f0  denovo5109_f0  denovo5161_f0  denovo5213_f0  denovo5421_f0  denovo5447_f0  denovo5551_f0  denovo5577_f0  denovo5915_f0  denovo5967_f0  denovo6019_f0  denovo6045_f0  denovo6071_f0  denovo6097_f0  denovo6201_f0  denovo6253_f0  denovo6279_f0  denovo6305_f0  denovo6383_f0  denovo6513_f0  denovo6565_f0  denovo6591_f0  denovo6617_f0  denovo6669_f0  denovo6695_f0  denovo6721_f0  denovo6747_f0  denovo6773_f0  denovo6903_f0  denovo6981_f0  denovo7007_f0  denovo7033_f0  denovo7085_f0  denovo7137_f0  denovo7163_f0  denovo7293_f0  denovo7319_f0  denovo7345_f0  denovo7371_f0  denovo7397_f0  denovo7475_f0  denovo7501_f0  denovo7527_f0  denovo7553_f0  denovo7631_f0  denovo7683_f0  denovo7709_f0  denovo7787_f0  denovo7839_f0  denovo7917_f0  denovo8021_f0  denovo8047_f0  denovo8229_f0  denovo8255_f0  denovo8281_f0  denovo8359_f0  denovo8385_f0  denovo8411_f0  denovo8489_f0  denovo8541_f0  denovo8567_f0  denovo8619_f0  denovo8645_f0  denovo8697_f0  denovo8723_f0  denovo8749_f0  denovo8775_f0  denovo8905_f0  denovo8931_f0  denovo8957_f0  denovo8983_f0  denovo9113_f0  denovo9139_f0  denovo9243_f0  denovo9295_f0  denovo9321_f0  denovo9347_f0  denovo9451_f0  denovo9477_f0  denovo9503_f0  denovo9659_f0  denovo9711_f0  denovo9789_f0  denovo9815_f0  denovo9841_f0  denovo9893_f0  denovo9971_f0  denovo10023_f0 denovo10049_f0 denovo10205_f0 denovo10231_f0 denovo10283_f0 denovo10309_f0 denovo10439_f0 denovo10465_f0 denovo10517_f0 denovo10543_f0 denovo10569_f0 denovo10595_f0 denovo10673_f0 denovo10725_f0 denovo10751_f0 denovo10777_f0 denovo10829_f0 denovo11011_f0 denovo11037_f0 denovo11063_f0 denovo11193_f0 denovo11297_f0 denovo11349_f0 denovo11401_f0 denovo11427_f0 denovo11453_f0 denovo11479_f0 denovo11505_f0 denovo11531_f0 denovo11583_f0 denovo11609_f0 denovo11713_f0 denovo11739_f0 denovo11921_f0 denovo11947_f0 denovo12077_f0 
Bmoricds                      denovo0_f0     denovo78_f0    denovo156_f0   denovo182_f0   denovo260_f0   denovo312_f0   denovo364_f0   denovo442_f0   denovo520_f0   denovo676_f0   denovo832_f0   denovo1040_f0  denovo1144_f0  denovo1222_f0  denovo1248_f0  denovo1352_f0  denovo1378_f0  denovo1404_f0  denovo1482_f0  denovo1638_f0  denovo1664_f0  denovo1716_f0  denovo1794_f0  denovo1846_f0  denovo1924_f0  denovo1950_f0  denovo1976_f0  denovo2002_f0  denovo2080_f0  denovo2106_f0  denovo2132_f0  denovo2236_f0  denovo2262_f0  denovo2288_f0  denovo2314_f0  denovo2366_f0  denovo2470_f0  denovo2522_f0  denovo2548_f0  denovo2574_f0  denovo2600_f0  denovo2626_f0  denovo2652_f0  denovo2756_f0  denovo2834_f0  denovo2886_f0  denovo3146_f0  denovo3250_f0  denovo3302_f0  denovo3328_f0  denovo3432_f0  denovo3484_f0  denovo3588_f0  denovo3614_f0  denovo3640_f0  denovo3718_f0  denovo3744_f0  denovo3796_f0  denovo3822_f0  denovo3978_f0  denovo4056_f0  denovo4134_f0  denovo4238_f0  denovo4316_f0  denovo4342_f0  denovo4368_f0  denovo4394_f0  denovo4420_f0  denovo4498_f0  denovo4524_f0  denovo4628_f0  denovo4758_f0  denovo4836_f0  denovo4888_f0  denovo4914_f0  denovo4940_f0  denovo4966_f0  denovo4992_f0  denovo5096_f0  denovo5148_f0  denovo5200_f0  denovo5408_f0  denovo5434_f0  denovo5538_f0  denovo5564_f0  denovo5902_f0  denovo5954_f0  denovo6006_f0  denovo6032_f0  denovo6058_f0  denovo6084_f0  denovo6188_f0  denovo6240_f0  denovo6266_f0  denovo6292_f0  denovo6370_f0  denovo6500_f0  denovo6552_f0  denovo6578_f0  denovo6604_f0  denovo6656_f0  denovo6682_f0  denovo6708_f0  denovo6734_f0  denovo6760_f0  denovo6890_f0  denovo6968_f0  denovo6994_f0  denovo7020_f0  denovo7072_f0  denovo7124_f0  denovo7150_f0  denovo7280_f0  denovo7306_f0  denovo7332_f0  denovo7358_f0  denovo7384_f0  denovo7462_f0  denovo7488_f0  denovo7514_f0  denovo7540_f0  denovo7618_f0  denovo7670_f0  denovo7696_f0  denovo7774_f0  denovo7826_f0  denovo7904_f0  denovo8008_f0  denovo8034_f0  denovo8216_f0  denovo8242_f0  denovo8268_f0  denovo8346_f0  denovo8372_f0  denovo8398_f0  denovo8476_f0  denovo8528_f0  denovo8554_f0  denovo8606_f0  denovo8632_f0  denovo8684_f0  denovo8710_f0  denovo8736_f0  denovo8762_f0  denovo8892_f0  denovo8918_f0  denovo8944_f0  denovo8970_f0  denovo9100_f0  denovo9126_f0  denovo9230_f0  denovo9282_f0  denovo9308_f0  denovo9334_f0  denovo9438_f0  denovo9464_f0  denovo9490_f0  denovo9646_f0  denovo9698_f0  denovo9776_f0  denovo9802_f0  denovo9828_f0  denovo9880_f0  denovo9958_f0  denovo10010_f0 denovo10036_f0 denovo10192_f0 denovo10218_f0 denovo10270_f0 denovo10296_f0 denovo10426_f0 denovo10452_f0 denovo10504_f0 denovo10530_f0 denovo10556_f0 denovo10582_f0 denovo10660_f0 denovo10712_f0 denovo10738_f0 denovo10764_f0 denovo10816_f0 denovo10998_f0 denovo11024_f0 denovo11050_f0 denovo11180_f0 denovo11284_f0 denovo11336_f0 denovo11388_f0 denovo11414_f0 denovo11440_f0 denovo11466_f0 denovo11492_f0 denovo11518_f0 denovo11570_f0 denovo11596_f0 denovo11700_f0 denovo11726_f0 denovo11908_f0 denovo11934_f0 denovo12064_f0 
Pcit2                         denovo19_f0    denovo97_f0    denovo175_f0   denovo201_f0   denovo279_f0   denovo331_f0   denovo383_f0   denovo461_f0   denovo539_f0   denovo695_f0   denovo851_f0   denovo1059_f0  denovo1163_f0  denovo1241_f0  denovo1267_f0  denovo1371_f0  denovo1397_f0  denovo1423_f0  denovo1501_f0  denovo1657_f0  denovo1683_f0  denovo1735_f0  denovo1813_f0  denovo1865_f0  denovo1943_f0  denovo1969_f0  denovo1995_f0  denovo2021_f0  denovo2099_f0  denovo2125_f0  denovo2151_f0  denovo2255_f0  denovo2281_f0  denovo2307_f0  denovo2333_f0  denovo2385_f0  denovo2489_f0  denovo2541_f0  denovo2567_f0  denovo2593_f0  denovo2619_f0  denovo2645_f0  denovo2671_f0  denovo2775_f0  denovo2853_f0  denovo2905_f0  denovo3165_f0  denovo3269_f0  denovo3321_f0  denovo3347_f0  denovo3451_f0  denovo3503_f0  denovo3607_f0  denovo3633_f0  denovo3659_f0  denovo3737_f0  denovo3763_f0  denovo3815_f0  denovo3841_f0  denovo3997_f0  denovo4075_f0  denovo4153_f0  denovo4257_f0  denovo4335_f0  denovo4361_f0  denovo4387_f0  denovo4413_f0  denovo4439_f0  denovo4517_f0  denovo4543_f0  denovo4647_f0  denovo4777_f0  denovo4855_f0  denovo4907_f0  denovo4933_f0  denovo4959_f0  denovo4985_f0  denovo5011_f0  denovo5115_f0  denovo5167_f0  denovo5219_f0  denovo5427_f0  denovo5453_f0  denovo5557_f0  denovo5583_f0  denovo5921_f0  denovo5973_f0  denovo6025_f0  denovo6051_f0  denovo6077_f0  denovo6103_f0  denovo6207_f0  denovo6259_f0  denovo6285_f0  denovo6311_f0  denovo6389_f0  denovo6519_f0  denovo6571_f0  denovo6597_f0  denovo6623_f0  denovo6675_f0  denovo6701_f0  denovo6727_f0  denovo6753_f0  denovo6779_f0  denovo6909_f0  denovo6987_f0  denovo7013_f0  denovo7039_f0  denovo7091_f0  denovo7143_f0  denovo7169_f0  denovo7299_f0  denovo7325_f0  denovo7351_f0  denovo7377_f0  denovo7403_f0  denovo7481_f0  denovo7507_f0  denovo7533_f0  denovo7559_f0  denovo7637_f0  denovo7689_f0  denovo7715_f0  denovo7793_f0  denovo7845_f0  denovo7923_f0  denovo8027_f0  denovo8053_f0  denovo8235_f0  denovo8261_f0  denovo8287_f0  denovo8365_f0  denovo8391_f0  denovo8417_f0  denovo8495_f0  denovo8547_f0  denovo8573_f0  denovo8625_f0  denovo8651_f0  denovo8703_f0  denovo8729_f0  denovo8755_f0  denovo8781_f0  denovo8911_f0  denovo8937_f0  denovo8963_f0  denovo8989_f0  denovo9119_f0  denovo9145_f0  denovo9249_f0  denovo9301_f0  denovo9327_f0  denovo9353_f0  denovo9457_f0  denovo9483_f0  denovo9509_f0  denovo9665_f0  denovo9717_f0  denovo9795_f0  denovo9821_f0  denovo9847_f0  denovo9899_f0  denovo9977_f0  denovo10029_f0 denovo10055_f0 denovo10211_f0 denovo10237_f0 denovo10289_f0 denovo10315_f0 denovo10445_f0 denovo10471_f0 denovo10523_f0 denovo10549_f0 denovo10575_f0 denovo10601_f0 denovo10679_f0 denovo10731_f0 denovo10757_f0 denovo10783_f0 denovo10835_f0 denovo11017_f0 denovo11043_f0 denovo11069_f0 denovo11199_f0 denovo11303_f0 denovo11355_f0 denovo11407_f0 denovo11433_f0 denovo11459_f0 denovo11485_f0 denovo11511_f0 denovo11537_f0 denovo11589_f0 denovo11615_f0 denovo11719_f0 denovo11745_f0 denovo11927_f0 denovo11953_f0 denovo12083_f0 

Concatenation entropy_0.00_0.00_loci_150_to_349 will have the following data
OTU                           EOG69CQC1_1         EOG69KFV8_1         EOG6SN1SH_1         EOG6PRSVJ_1         EOG6PRSVH_1         EOG60ZR1Z_1         EOG6FXRCN_1         EOG60P4BD_1         EOG6NCMGZ_1         EOG670TN2_1         EOG63TZZV_1         EOG6G7C30_1         EOG6K6G7Z_1         EOG6SXNH7_1         EOG65X80D_1         EOG68KRFS_1         EOG6MSD2X_1         EOG698V44_1         EOG6F7NPM_1         EOG676K3N_1         EOG6894QQ_1         EOG6868GW_1         EOG69PB31_1         EOG6JDHBD_1         EOG6C87X4_1         EOG68D102_1         EOG60ZR1T_1         EOG63JC89_1         EOG6J6RV9_1         EOG68D104_1         EOG68KRG8_1         EOG6N8R81_1         EOG63BMSV_1         EOG69KFV1_1         EOG6CJVMF_1         EOG6C2HF4_1         EOG6CC44S_1         EOG6FXRCK_1         EOG68KRG4_1         EOG61C70T_1         EOG6BK575_1         EOG6R506X_1         EOG6JT09G_1         EOG6HQDPG_1         EOG6H4677_1         EOG6SJ5J3_1         EOG6NZTZ3_1         EOG6J3WM7_1         EOG680J16_1         EOG602WWN_1         EOG6RNBCT_1         EOG63BMSB_1         EOG6B8JHB_1         EOG647FXX_1         EOG6B8JHP_1         EOG6S1TBX_1         EOG65HS25_1         EOG6FFD5C_1         EOG6PNXM9_1         EOG6F4SFZ_1         EOG6Q8524_1         EOG669QZR_1         EOG6JT09B_1         EOG6CJVMM_1         EOG6QRH81_1         EOG6NCMH9_1         EOG65HS1N_1         EOG6CC44T_1         EOG6FR0VW_1         EOG6KKZ6X_1         EOG60VVTG_1         EOG6QC198_1         EOG6FFD52_1         EOG634W9K_1         EOG6DNF7N_1         EOG61RPZF_1         EOG605S4X_1         EOG69GKMS_1         EOG6FR0VX_1         EOG6B8JHC_1         EOG6QVCGS_1         EOG6Q2DJZ_1         EOG6CC44M_1         EOG6NS3G8_1         EOG68GW77_1         EOG6229Q2_1         EOG63TZZW_1         EOG6MW89D_1         EOG6SJ5K0_1         EOG6MKNM3_1         EOG63V00H_1         EOG6BG90S_1         EOG66DM62_1         EOG69CQBW_1         EOG69KFTV_1         EOG62V8KS_1         EOG6FFD51_1         EOG6QNN0J_1         EOG6PC9WN_1         EOG6Q8529_1         EOG6KSPP5_1         EOG61ZFF6_1         EOG6N5W22_1         EOG63V009_1         EOG6PK2CG_1         EOG68GW6V_1         EOG66Q6XP_1         EOG6C5CNP_1         EOG6R5066_1         EOG641QFN_1         EOG65B1K2_1         EOG65TCRK_1         EOG6PNXN7_1         EOG6P2Q64_1         EOG666VQC_1         EOG6PC9WF_1         EOG6Q58T0_1         EOG641QGH_1         EOG6933N7_1         EOG69S6BG_1         EOG66Q6XR_1         EOG6PK2CM_1         EOG6NS3FV_1         EOG641QFW_1         EOG6N04J6_1         EOG6CRM3G_1         EOG6S4PKX_1         EOG6K6G7S_1         EOG6BZN62_1         EOG68SGZ1_1         EOG6SXNGX_1         EOG6QZ7Q8_1         EOG6QC18P_1         EOG6CG0CT_1         EOG6PK2CQ_1         EOG6JWVJQ_1         EOG6PG64S_1         EOG63R3RK_1         EOG64F6DK_1         EOG6FJ8D6_1         EOG6001NN_1         EOG695ZWC_1         EOG67PX9B_1         EOG6P8FNH_1         EOG6KSPQ0_1         EOG63R3RC_1         EOG6907D7_1         EOG6JWVHT_1         EOG68SGX8_1         EOG6933NR_1         EOG62JNVR_1         EOG64MXWZ_1         EOG6S1TC2_1         EOG6PC9X3_1         EOG6Q58SW_1         EOG6CRM3H_1         EOG6R5069_1         EOG64MXWK_1         EOG6N30SN_1         EOG68KRFQ_1         EOG6DR9GZ_1         EOG62FSN7_1         EOG6PZJB1_1         EOG65QHHP_1         EOG615GHV_1         EOG66WZCQ_1         EOG698V4H_1         EOG6JDHB2_1         EOG62BXCX_1         EOG69KFVF_1         EOG615GHP_1         EOG66HGF5_1         EOG6DFPSF_1         EOG66MBPR_1         EOG6DFPSD_1         EOG67D9KC_1         EOG6HHP5S_1         EOG6DBTHZ_1         EOG6KH2ZD_1         EOG6STS87_1         EOG67D9KV_1         EOG65MN8T_1         EOG61RPZ9_1         EOG66MBP7_1         EOG6QJRRQ_1         EOG6Q852B_1         EOG6DV5QM_1         EOG6K0QRT_1         EOG65DWSM_1         EOG6G4GTX_1         EOG64BB5H_1         EOG64J2NC_1         EOG6H72G3_1         EOG6SBF2B_1         EOG6FFD5V_1         EOG6G4GTT_1         EOG6K3M11_1         EOG66147J_1         EOG63FH1C_1         EOG61C70W_1         
Dplexcds                      denovo2_f0     denovo54_f0    denovo80_f0    denovo158_f0   denovo184_f0   denovo262_f0   denovo314_f0   denovo366_f0   denovo444_f0   denovo548_f0   denovo678_f0   denovo834_f0   denovo860_f0   denovo886_f0   denovo912_f0   denovo1042_f0  denovo1172_f0  denovo1224_f0  denovo1250_f0  denovo1276_f0  denovo1354_f0  denovo1380_f0  denovo1406_f0  denovo1484_f0  denovo1562_f0  denovo1640_f0  denovo1666_f0  denovo1718_f0  denovo1848_f0  denovo1900_f0  denovo1926_f0  denovo1952_f0  denovo1978_f0  denovo2004_f0  denovo2056_f0  denovo2082_f0  denovo2108_f0  denovo2134_f0  denovo2238_f0  denovo2264_f0  denovo2290_f0  denovo2316_f0  denovo2368_f0  denovo2394_f0  denovo2420_f0  denovo2550_f0  denovo2576_f0  denovo2602_f0  denovo2628_f0  denovo2732_f0  denovo2758_f0  denovo2810_f0  denovo2888_f0  denovo3304_f0  denovo3330_f0  denovo3434_f0  denovo3590_f0  denovo3616_f0  denovo3642_f0  denovo3720_f0  denovo3746_f0  denovo3798_f0  denovo3824_f0  denovo3876_f0  denovo4058_f0  denovo4084_f0  denovo4136_f0  denovo4162_f0  denovo4240_f0  denovo4318_f0  denovo4344_f0  denovo4370_f0  denovo4422_f0  denovo4500_f0  denovo4526_f0  denovo4630_f0  denovo4838_f0  denovo4994_f0  denovo5020_f0  denovo5098_f0  denovo5124_f0  denovo5150_f0  denovo5332_f0  denovo5410_f0  denovo5488_f0  denovo5540_f0  denovo5566_f0  denovo5722_f0  denovo5774_f0  denovo5852_f0  denovo5878_f0  denovo5904_f0  denovo5956_f0  denovo6034_f0  denovo6086_f0  denovo6190_f0  denovo6268_f0  denovo6294_f0  denovo6320_f0  denovo6346_f0  denovo6372_f0  denovo6476_f0  denovo6502_f0  denovo6528_f0  denovo6554_f0  denovo6632_f0  denovo6658_f0  denovo6710_f0  denovo6736_f0  denovo6892_f0  denovo6970_f0  denovo7022_f0  denovo7048_f0  denovo7126_f0  denovo7152_f0  denovo7282_f0  denovo7308_f0  denovo7334_f0  denovo7360_f0  denovo7386_f0  denovo7438_f0  denovo7464_f0  denovo7490_f0  denovo7542_f0  denovo7620_f0  denovo7646_f0  denovo7698_f0  denovo7776_f0  denovo7828_f0  denovo7854_f0  denovo7906_f0  denovo7932_f0  denovo8010_f0  denovo8036_f0  denovo8088_f0  denovo8140_f0  denovo8192_f0  denovo8348_f0  denovo8374_f0  denovo8400_f0  denovo8478_f0  denovo8530_f0  denovo8556_f0  denovo8608_f0  denovo8634_f0  denovo8686_f0  denovo8712_f0  denovo8738_f0  denovo8764_f0  denovo9050_f0  denovo9076_f0  denovo9102_f0  denovo9128_f0  denovo9206_f0  denovo9232_f0  denovo9284_f0  denovo9336_f0  denovo9466_f0  denovo9492_f0  denovo9622_f0  denovo9648_f0  denovo9778_f0  denovo9830_f0  denovo9882_f0  denovo9960_f0  denovo10012_f0 denovo10038_f0 denovo10116_f0 denovo10142_f0 denovo10272_f0 denovo10298_f0 denovo10428_f0 denovo10454_f0 denovo10506_f0 denovo10532_f0 denovo10558_f0 denovo10584_f0 denovo10714_f0 denovo10740_f0 denovo10766_f0 denovo10818_f0 denovo10896_f0 denovo11000_f0 denovo11052_f0 denovo11130_f0 denovo11208_f0 denovo11286_f0 denovo11390_f0 denovo11416_f0 denovo11468_f0 denovo11494_f0 denovo11520_f0 denovo11572_f0 denovo11598_f0 denovo11702_f0 denovo11728_f0 denovo11754_f0 denovo11858_f0 denovo11884_f0 denovo11936_f0 
FG120077                      denovo10_f0    denovo62_f0    denovo88_f0    denovo166_f0   denovo192_f0   denovo270_f0   denovo322_f0   denovo374_f0   denovo452_f0   denovo556_f0   denovo686_f0   denovo842_f0   denovo868_f0   denovo894_f0   denovo920_f0   denovo1050_f0  denovo1180_f0  denovo1232_f0  denovo1258_f0  denovo1284_f0  denovo1362_f0  denovo1388_f0  denovo1414_f0  denovo1492_f0  denovo1570_f0  denovo1648_f0  denovo1674_f0  denovo1726_f0  denovo1856_f0  denovo1908_f0  denovo1934_f0  denovo1960_f0  denovo1986_f0  denovo2012_f0  denovo2064_f0  denovo2090_f0  denovo2116_f0  denovo2142_f0  denovo2246_f0  denovo2272_f0  denovo2298_f0  denovo2324_f0  denovo2376_f0  denovo2402_f0  denovo2428_f0  denovo2558_f0  denovo2584_f0  denovo2610_f0  denovo2636_f0  denovo2740_f0  denovo2766_f0  denovo2818_f0  denovo2896_f0  denovo3312_f0  denovo3338_f0  denovo3442_f0  denovo3598_f0  denovo3624_f0  denovo3650_f0  denovo3728_f0  denovo3754_f0  denovo3806_f0  denovo3832_f0  denovo3884_f0  denovo4066_f0  denovo4092_f0  denovo4144_f0  denovo4170_f0  denovo4248_f0  denovo4326_f0  denovo4352_f0  denovo4378_f0  denovo4430_f0  denovo4508_f0  denovo4534_f0  denovo4638_f0  denovo4846_f0  denovo5002_f0  denovo5028_f0  denovo5106_f0  denovo5132_f0  denovo5158_f0  denovo5340_f0  denovo5418_f0  denovo5496_f0  denovo5548_f0  denovo5574_f0  denovo5730_f0  denovo5782_f0  denovo5860_f0  denovo5886_f0  denovo5912_f0  denovo5964_f0  denovo6042_f0  denovo6094_f0  denovo6198_f0  denovo6276_f0  denovo6302_f0  denovo6328_f0  denovo6354_f0  denovo6380_f0  denovo6484_f0  denovo6510_f0  denovo6536_f0  denovo6562_f0  denovo6640_f0  denovo6666_f0  denovo6718_f0  denovo6744_f0  denovo6900_f0  denovo6978_f0  denovo7030_f0  denovo7056_f0  denovo7134_f0  denovo7160_f0  denovo7290_f0  denovo7316_f0  denovo7342_f0  denovo7368_f0  denovo7394_f0  denovo7446_f0  denovo7472_f0  denovo7498_f0  denovo7550_f0  denovo7628_f0  denovo7654_f0  denovo7706_f0  denovo7784_f0  denovo7836_f0  denovo7862_f0  denovo7914_f0  denovo7940_f0  denovo8018_f0  denovo8044_f0  denovo8096_f0  denovo8148_f0  denovo8200_f0  denovo8356_f0  denovo8382_f0  denovo8408_f0  denovo8486_f0  denovo8538_f0  denovo8564_f0  denovo8616_f0  denovo8642_f0  denovo8694_f0  denovo8720_f0  denovo8746_f0  denovo8772_f0  denovo9058_f0  denovo9084_f0  denovo9110_f0  denovo9136_f0  denovo9214_f0  denovo9240_f0  denovo9292_f0  denovo9344_f0  denovo9474_f0  denovo9500_f0  denovo9630_f0  denovo9656_f0  denovo9786_f0  denovo9838_f0  denovo9890_f0  denovo9968_f0  denovo10020_f0 denovo10046_f0 denovo10124_f0 denovo10150_f0 denovo10280_f0 denovo10306_f0 denovo10436_f0 denovo10462_f0 denovo10514_f0 denovo10540_f0 denovo10566_f0 denovo10592_f0 denovo10722_f0 denovo10748_f0 denovo10774_f0 denovo10826_f0 denovo10904_f0 denovo11008_f0 denovo11060_f0 denovo11138_f0 denovo11216_f0 denovo11294_f0 denovo11398_f0 denovo11424_f0 denovo11476_f0 denovo11502_f0 denovo11528_f0 denovo11580_f0 denovo11606_f0 denovo11710_f0 denovo11736_f0 denovo11762_f0 denovo11866_f0 denovo11892_f0 denovo11944_f0 
SRR803483                     denovo20_f0    denovo72_f0    denovo98_f0    denovo176_f0   denovo202_f0   denovo280_f0   denovo332_f0   denovo384_f0   denovo462_f0   denovo566_f0   denovo696_f0   denovo852_f0   denovo878_f0   denovo904_f0   denovo930_f0   denovo1060_f0  denovo1190_f0  denovo1242_f0  denovo1268_f0  denovo1294_f0  denovo1372_f0  denovo1398_f0  denovo1424_f0  denovo1502_f0  denovo1580_f0  denovo1658_f0  denovo1684_f0  denovo1736_f0  denovo1866_f0  denovo1918_f0  denovo1944_f0  denovo1970_f0  denovo1996_f0  denovo2022_f0  denovo2074_f0  denovo2100_f0  denovo2126_f0  denovo2152_f0  denovo2256_f0  denovo2282_f0  denovo2308_f0  denovo2334_f0  denovo2386_f0  denovo2412_f0  denovo2438_f0  denovo2568_f0  denovo2594_f0  denovo2620_f0  denovo2646_f0  denovo2750_f0  denovo2776_f0  denovo2828_f0  denovo2906_f0  denovo3322_f0  denovo3348_f0  denovo3452_f0  denovo3608_f0  denovo3634_f0  denovo3660_f0  denovo3738_f0  denovo3764_f0  denovo3816_f0  denovo3842_f0  denovo3894_f0  denovo4076_f0  denovo4102_f0  denovo4154_f0  denovo4180_f0  denovo4258_f0  denovo4336_f0  denovo4362_f0  denovo4388_f0  denovo4440_f0  denovo4518_f0  denovo4544_f0  denovo4648_f0  denovo4856_f0  denovo5012_f0  denovo5038_f0  denovo5116_f0  denovo5142_f0  denovo5168_f0  denovo5350_f0  denovo5428_f0  denovo5506_f0  denovo5558_f0  denovo5584_f0  denovo5740_f0  denovo5792_f0  denovo5870_f0  denovo5896_f0  denovo5922_f0  denovo5974_f0  denovo6052_f0  denovo6104_f0  denovo6208_f0  denovo6286_f0  denovo6312_f0  denovo6338_f0  denovo6364_f0  denovo6390_f0  denovo6494_f0  denovo6520_f0  denovo6546_f0  denovo6572_f0  denovo6650_f0  denovo6676_f0  denovo6728_f0  denovo6754_f0  denovo6910_f0  denovo6988_f0  denovo7040_f0  denovo7066_f0  denovo7144_f0  denovo7170_f0  denovo7300_f0  denovo7326_f0  denovo7352_f0  denovo7378_f0  denovo7404_f0  denovo7456_f0  denovo7482_f0  denovo7508_f0  denovo7560_f0  denovo7638_f0  denovo7664_f0  denovo7716_f0  denovo7794_f0  denovo7846_f0  denovo7872_f0  denovo7924_f0  denovo7950_f0  denovo8028_f0  denovo8054_f0  denovo8106_f0  denovo8158_f0  denovo8210_f0  denovo8366_f0  denovo8392_f0  denovo8418_f0  denovo8496_f0  denovo8548_f0  denovo8574_f0  denovo8626_f0  denovo8652_f0  denovo8704_f0  denovo8730_f0  denovo8756_f0  denovo8782_f0  denovo9068_f0  denovo9094_f0  denovo9120_f0  denovo9146_f0  denovo9224_f0  denovo9250_f0  denovo9302_f0  denovo9354_f0  denovo9484_f0  denovo9510_f0  denovo9640_f0  denovo9666_f0  denovo9796_f0  denovo9848_f0  denovo9900_f0  denovo9978_f0  denovo10030_f0 denovo10056_f0 denovo10134_f0 denovo10160_f0 denovo10290_f0 denovo10316_f0 denovo10446_f0 denovo10472_f0 denovo10524_f0 denovo10550_f0 denovo10576_f0 denovo10602_f0 denovo10732_f0 denovo10758_f0 denovo10784_f0 denovo10836_f0 denovo10914_f0 denovo11018_f0 denovo11070_f0 denovo11148_f0 denovo11226_f0 denovo11304_f0 denovo11408_f0 denovo11434_f0 denovo11486_f0 denovo11512_f0 denovo11538_f0 denovo11590_f0 denovo11616_f0 denovo11720_f0 denovo11746_f0 denovo11772_f0 denovo11876_f0 denovo11902_f0 denovo11954_f0 
FG120035                      denovo5_f0     denovo57_f0    denovo83_f0    denovo161_f0   denovo187_f0   denovo265_f0   denovo317_f0   denovo369_f0   denovo447_f0   denovo551_f0   denovo681_f0   denovo837_f0   denovo863_f0   denovo889_f0   denovo915_f0   denovo1045_f0  denovo1175_f0  denovo1227_f0  denovo1253_f0  denovo1279_f0  denovo1357_f0  denovo1383_f0  denovo1409_f0  denovo1487_f0  denovo1565_f0  denovo1643_f0  denovo1669_f0  denovo1721_f0  denovo1851_f0  denovo1903_f0  denovo1929_f0  denovo1955_f0  denovo1981_f0  denovo2007_f0  denovo2059_f0  denovo2085_f0  denovo2111_f0  denovo2137_f0  denovo2241_f0  denovo2267_f0  denovo2293_f0  denovo2319_f0  denovo2371_f0  denovo2397_f0  denovo2423_f0  denovo2553_f0  denovo2579_f0  denovo2605_f0  denovo2631_f0  denovo2735_f0  denovo2761_f0  denovo2813_f0  denovo2891_f0  denovo3307_f0  denovo3333_f0  denovo3437_f0  denovo3593_f0  denovo3619_f0  denovo3645_f0  denovo3723_f0  denovo3749_f0  denovo3801_f0  denovo3827_f0  denovo3879_f0  denovo4061_f0  denovo4087_f0  denovo4139_f0  denovo4165_f0  denovo4243_f0  denovo4321_f0  denovo4347_f0  denovo4373_f0  denovo4425_f0  denovo4503_f0  denovo4529_f0  denovo4633_f0  denovo4841_f0  denovo4997_f0  denovo5023_f0  denovo5101_f0  denovo5127_f0  denovo5153_f0  denovo5335_f0  denovo5413_f0  denovo5491_f0  denovo5543_f0  denovo5569_f0  denovo5725_f0  denovo5777_f0  denovo5855_f0  denovo5881_f0  denovo5907_f0  denovo5959_f0  denovo6037_f0  denovo6089_f0  denovo6193_f0  denovo6271_f0  denovo6297_f0  denovo6323_f0  denovo6349_f0  denovo6375_f0  denovo6479_f0  denovo6505_f0  denovo6531_f0  denovo6557_f0  denovo6635_f0  denovo6661_f0  denovo6713_f0  denovo6739_f0  denovo6895_f0  denovo6973_f0  denovo7025_f0  denovo7051_f0  denovo7129_f0  denovo7155_f0  denovo7285_f0  denovo7311_f0  denovo7337_f0  denovo7363_f0  denovo7389_f0  denovo7441_f0  denovo7467_f0  denovo7493_f0  denovo7545_f0  denovo7623_f0  denovo7649_f0  denovo7701_f0  denovo7779_f0  denovo7831_f0  denovo7857_f0  denovo7909_f0  denovo7935_f0  denovo8013_f0  denovo8039_f0  denovo8091_f0  denovo8143_f0  denovo8195_f0  denovo8351_f0  denovo8377_f0  denovo8403_f0  denovo8481_f0  denovo8533_f0  denovo8559_f0  denovo8611_f0  denovo8637_f0  denovo8689_f0  denovo8715_f0  denovo8741_f0  denovo8767_f0  denovo9053_f0  denovo9079_f0  denovo9105_f0  denovo9131_f0  denovo9209_f0  denovo9235_f0  denovo9287_f0  denovo9339_f0  denovo9469_f0  denovo9495_f0  denovo9625_f0  denovo9651_f0  denovo9781_f0  denovo9833_f0  denovo9885_f0  denovo9963_f0  denovo10015_f0 denovo10041_f0 denovo10119_f0 denovo10145_f0 denovo10275_f0 denovo10301_f0 denovo10431_f0 denovo10457_f0 denovo10509_f0 denovo10535_f0 denovo10561_f0 denovo10587_f0 denovo10717_f0 denovo10743_f0 denovo10769_f0 denovo10821_f0 denovo10899_f0 denovo11003_f0 denovo11055_f0 denovo11133_f0 denovo11211_f0 denovo11289_f0 denovo11393_f0 denovo11419_f0 denovo11471_f0 denovo11497_f0 denovo11523_f0 denovo11575_f0 denovo11601_f0 denovo11705_f0 denovo11731_f0 denovo11757_f0 denovo11861_f0 denovo11887_f0 denovo11939_f0 
FG120046B                     denovo6_f0     denovo58_f0    denovo84_f0    denovo162_f0   denovo188_f0   denovo266_f0   denovo318_f0   denovo370_f0   denovo448_f0   denovo552_f0   denovo682_f0   denovo838_f0   denovo864_f0   denovo890_f0   denovo916_f0   denovo1046_f0  denovo1176_f0  denovo1228_f0  denovo1254_f0  denovo1280_f0  denovo1358_f0  denovo1384_f0  denovo1410_f0  denovo1488_f0  denovo1566_f0  denovo1644_f0  denovo1670_f0  denovo1722_f0  denovo1852_f0  denovo1904_f0  denovo1930_f0  denovo1956_f0  denovo1982_f0  denovo2008_f0  denovo2060_f0  denovo2086_f0  denovo2112_f0  denovo2138_f0  denovo2242_f0  denovo2268_f0  denovo2294_f0  denovo2320_f0  denovo2372_f0  denovo2398_f0  denovo2424_f0  denovo2554_f0  denovo2580_f0  denovo2606_f0  denovo2632_f0  denovo2736_f0  denovo2762_f0  denovo2814_f0  denovo2892_f0  denovo3308_f0  denovo3334_f0  denovo3438_f0  denovo3594_f0  denovo3620_f0  denovo3646_f0  denovo3724_f0  denovo3750_f0  denovo3802_f0  denovo3828_f0  denovo3880_f0  denovo4062_f0  denovo4088_f0  denovo4140_f0  denovo4166_f0  denovo4244_f0  denovo4322_f0  denovo4348_f0  denovo4374_f0  denovo4426_f0  denovo4504_f0  denovo4530_f0  denovo4634_f0  denovo4842_f0  denovo4998_f0  denovo5024_f0  denovo5102_f0  denovo5128_f0  denovo5154_f0  denovo5336_f0  denovo5414_f0  denovo5492_f0  denovo5544_f0  denovo5570_f0  denovo5726_f0  denovo5778_f0  denovo5856_f0  denovo5882_f0  denovo5908_f0  denovo5960_f0  denovo6038_f0  denovo6090_f0  denovo6194_f0  denovo6272_f0  denovo6298_f0  denovo6324_f0  denovo6350_f0  denovo6376_f0  denovo6480_f0  denovo6506_f0  denovo6532_f0  denovo6558_f0  denovo6636_f0  denovo6662_f0  denovo6714_f0  denovo6740_f0  denovo6896_f0  denovo6974_f0  denovo7026_f0  denovo7052_f0  denovo7130_f0  denovo7156_f0  denovo7286_f0  denovo7312_f0  denovo7338_f0  denovo7364_f0  denovo7390_f0  denovo7442_f0  denovo7468_f0  denovo7494_f0  denovo7546_f0  denovo7624_f0  denovo7650_f0  denovo7702_f0  denovo7780_f0  denovo7832_f0  denovo7858_f0  denovo7910_f0  denovo7936_f0  denovo8014_f0  denovo8040_f0  denovo8092_f0  denovo8144_f0  denovo8196_f0  denovo8352_f0  denovo8378_f0  denovo8404_f0  denovo8482_f0  denovo8534_f0  denovo8560_f0  denovo8612_f0  denovo8638_f0  denovo8690_f0  denovo8716_f0  denovo8742_f0  denovo8768_f0  denovo9054_f0  denovo9080_f0  denovo9106_f0  denovo9132_f0  denovo9210_f0  denovo9236_f0  denovo9288_f0  denovo9340_f0  denovo9470_f0  denovo9496_f0  denovo9626_f0  denovo9652_f0  denovo9782_f0  denovo9834_f0  denovo9886_f0  denovo9964_f0  denovo10016_f0 denovo10042_f0 denovo10120_f0 denovo10146_f0 denovo10276_f0 denovo10302_f0 denovo10432_f0 denovo10458_f0 denovo10510_f0 denovo10536_f0 denovo10562_f0 denovo10588_f0 denovo10718_f0 denovo10744_f0 denovo10770_f0 denovo10822_f0 denovo10900_f0 denovo11004_f0 denovo11056_f0 denovo11134_f0 denovo11212_f0 denovo11290_f0 denovo11394_f0 denovo11420_f0 denovo11472_f0 denovo11498_f0 denovo11524_f0 denovo11576_f0 denovo11602_f0 denovo11706_f0 denovo11732_f0 denovo11758_f0 denovo11862_f0 denovo11888_f0 denovo11940_f0 
GNV129007                     denovo15_f0    denovo67_f0    denovo93_f0    denovo171_f0   denovo197_f0   denovo275_f0   denovo327_f0   denovo379_f0   denovo457_f0   denovo561_f0   denovo691_f0   denovo847_f0   denovo873_f0   denovo899_f0   denovo925_f0   denovo1055_f0  denovo1185_f0  denovo1237_f0  denovo1263_f0  denovo1289_f0  denovo1367_f0  denovo1393_f0  denovo1419_f0  denovo1497_f0  denovo1575_f0  denovo1653_f0  denovo1679_f0  denovo1731_f0  denovo1861_f0  denovo1913_f0  denovo1939_f0  denovo1965_f0  denovo1991_f0  denovo2017_f0  denovo2069_f0  denovo2095_f0  denovo2121_f0  denovo2147_f0  denovo2251_f0  denovo2277_f0  denovo2303_f0  denovo2329_f0  denovo2381_f0  denovo2407_f0  denovo2433_f0  denovo2563_f0  denovo2589_f0  denovo2615_f0  denovo2641_f0  denovo2745_f0  denovo2771_f0  denovo2823_f0  denovo2901_f0  denovo3317_f0  denovo3343_f0  denovo3447_f0  denovo3603_f0  denovo3629_f0  denovo3655_f0  denovo3733_f0  denovo3759_f0  denovo3811_f0  denovo3837_f0  denovo3889_f0  denovo4071_f0  denovo4097_f0  denovo4149_f0  denovo4175_f0  denovo4253_f0  denovo4331_f0  denovo4357_f0  denovo4383_f0  denovo4435_f0  denovo4513_f0  denovo4539_f0  denovo4643_f0  denovo4851_f0  denovo5007_f0  denovo5033_f0  denovo5111_f0  denovo5137_f0  denovo5163_f0  denovo5345_f0  denovo5423_f0  denovo5501_f0  denovo5553_f0  denovo5579_f0  denovo5735_f0  denovo5787_f0  denovo5865_f0  denovo5891_f0  denovo5917_f0  denovo5969_f0  denovo6047_f0  denovo6099_f0  denovo6203_f0  denovo6281_f0  denovo6307_f0  denovo6333_f0  denovo6359_f0  denovo6385_f0  denovo6489_f0  denovo6515_f0  denovo6541_f0  denovo6567_f0  denovo6645_f0  denovo6671_f0  denovo6723_f0  denovo6749_f0  denovo6905_f0  denovo6983_f0  denovo7035_f0  denovo7061_f0  denovo7139_f0  denovo7165_f0  denovo7295_f0  denovo7321_f0  denovo7347_f0  denovo7373_f0  denovo7399_f0  denovo7451_f0  denovo7477_f0  denovo7503_f0  denovo7555_f0  denovo7633_f0  denovo7659_f0  denovo7711_f0  denovo7789_f0  denovo7841_f0  denovo7867_f0  denovo7919_f0  denovo7945_f0  denovo8023_f0  denovo8049_f0  denovo8101_f0  denovo8153_f0  denovo8205_f0  denovo8361_f0  denovo8387_f0  denovo8413_f0  denovo8491_f0  denovo8543_f0  denovo8569_f0  denovo8621_f0  denovo8647_f0  denovo8699_f0  denovo8725_f0  denovo8751_f0  denovo8777_f0  denovo9063_f0  denovo9089_f0  denovo9115_f0  denovo9141_f0  denovo9219_f0  denovo9245_f0  denovo9297_f0  denovo9349_f0  denovo9479_f0  denovo9505_f0  denovo9635_f0  denovo9661_f0  denovo9791_f0  denovo9843_f0  denovo9895_f0  denovo9973_f0  denovo10025_f0 denovo10051_f0 denovo10129_f0 denovo10155_f0 denovo10285_f0 denovo10311_f0 denovo10441_f0 denovo10467_f0 denovo10519_f0 denovo10545_f0 denovo10571_f0 denovo10597_f0 denovo10727_f0 denovo10753_f0 denovo10779_f0 denovo10831_f0 denovo10909_f0 denovo11013_f0 denovo11065_f0 denovo11143_f0 denovo11221_f0 denovo11299_f0 denovo11403_f0 denovo11429_f0 denovo11481_f0 denovo11507_f0 denovo11533_f0 denovo11585_f0 denovo11611_f0 denovo11715_f0 denovo11741_f0 denovo11767_f0 denovo11871_f0 denovo11897_f0 denovo11949_f0 
SW130126                      denovo24_f0    denovo76_f0    denovo102_f0   denovo180_f0   denovo206_f0   denovo284_f0   denovo336_f0   denovo388_f0   denovo466_f0   denovo570_f0   denovo700_f0   denovo856_f0   denovo882_f0   denovo908_f0   denovo934_f0   denovo1064_f0  denovo1194_f0  denovo1246_f0  denovo1272_f0  denovo1298_f0  denovo1376_f0  denovo1402_f0  denovo1428_f0  denovo1506_f0  denovo1584_f0  denovo1662_f0  denovo1688_f0  denovo1740_f0  denovo1870_f0  denovo1922_f0  denovo1948_f0  denovo1974_f0  denovo2000_f0  denovo2026_f0  denovo2078_f0  denovo2104_f0  denovo2130_f0  denovo2156_f0  denovo2260_f0  denovo2286_f0  denovo2312_f0  denovo2338_f0  denovo2390_f0  denovo2416_f0  denovo2442_f0  denovo2572_f0  denovo2598_f0  denovo2624_f0  denovo2650_f0  denovo2754_f0  denovo2780_f0  denovo2832_f0  denovo2910_f0  denovo3326_f0  denovo3352_f0  denovo3456_f0  denovo3612_f0  denovo3638_f0  denovo3664_f0  denovo3742_f0  denovo3768_f0  denovo3820_f0  denovo3846_f0  denovo3898_f0  denovo4080_f0  denovo4106_f0  denovo4158_f0  denovo4184_f0  denovo4262_f0  denovo4340_f0  denovo4366_f0  denovo4392_f0  denovo4444_f0  denovo4522_f0  denovo4548_f0  denovo4652_f0  denovo4860_f0  denovo5016_f0  denovo5042_f0  denovo5120_f0  denovo5146_f0  denovo5172_f0  denovo5354_f0  denovo5432_f0  denovo5510_f0  denovo5562_f0  denovo5588_f0  denovo5744_f0  denovo5796_f0  denovo5874_f0  denovo5900_f0  denovo5926_f0  denovo5978_f0  denovo6056_f0  denovo6108_f0  denovo6212_f0  denovo6290_f0  denovo6316_f0  denovo6342_f0  denovo6368_f0  denovo6394_f0  denovo6498_f0  denovo6524_f0  denovo6550_f0  denovo6576_f0  denovo6654_f0  denovo6680_f0  denovo6732_f0  denovo6758_f0  denovo6914_f0  denovo6992_f0  denovo7044_f0  denovo7070_f0  denovo7148_f0  denovo7174_f0  denovo7304_f0  denovo7330_f0  denovo7356_f0  denovo7382_f0  denovo7408_f0  denovo7460_f0  denovo7486_f0  denovo7512_f0  denovo7564_f0  denovo7642_f0  denovo7668_f0  denovo7720_f0  denovo7798_f0  denovo7850_f0  denovo7876_f0  denovo7928_f0  denovo7954_f0  denovo8032_f0  denovo8058_f0  denovo8110_f0  denovo8162_f0  denovo8214_f0  denovo8370_f0  denovo8396_f0  denovo8422_f0  denovo8500_f0  denovo8552_f0  denovo8578_f0  denovo8630_f0  denovo8656_f0  denovo8708_f0  denovo8734_f0  denovo8760_f0  denovo8786_f0  denovo9072_f0  denovo9098_f0  denovo9124_f0  denovo9150_f0  denovo9228_f0  denovo9254_f0  denovo9306_f0  denovo9358_f0  denovo9488_f0  denovo9514_f0  denovo9644_f0  denovo9670_f0  denovo9800_f0  denovo9852_f0  denovo9904_f0  denovo9982_f0  denovo10034_f0 denovo10060_f0 denovo10138_f0 denovo10164_f0 denovo10294_f0 denovo10320_f0 denovo10450_f0 denovo10476_f0 denovo10528_f0 denovo10554_f0 denovo10580_f0 denovo10606_f0 denovo10736_f0 denovo10762_f0 denovo10788_f0 denovo10840_f0 denovo10918_f0 denovo11022_f0 denovo11074_f0 denovo11152_f0 denovo11230_f0 denovo11308_f0 denovo11412_f0 denovo11438_f0 denovo11490_f0 denovo11516_f0 denovo11542_f0 denovo11594_f0 denovo11620_f0 denovo11724_f0 denovo11750_f0 denovo11776_f0 denovo11880_f0 denovo11906_f0 denovo11958_f0 
SW130103                      denovo23_f0    denovo75_f0    denovo101_f0   denovo179_f0   denovo205_f0   denovo283_f0   denovo335_f0   denovo387_f0   denovo465_f0   denovo569_f0   denovo699_f0   denovo855_f0   denovo881_f0   denovo907_f0   denovo933_f0   denovo1063_f0  denovo1193_f0  denovo1245_f0  denovo1271_f0  denovo1297_f0  denovo1375_f0  denovo1401_f0  denovo1427_f0  denovo1505_f0  denovo1583_f0  denovo1661_f0  denovo1687_f0  denovo1739_f0  denovo1869_f0  denovo1921_f0  denovo1947_f0  denovo1973_f0  denovo1999_f0  denovo2025_f0  denovo2077_f0  denovo2103_f0  denovo2129_f0  denovo2155_f0  denovo2259_f0  denovo2285_f0  denovo2311_f0  denovo2337_f0  denovo2389_f0  denovo2415_f0  denovo2441_f0  denovo2571_f0  denovo2597_f0  denovo2623_f0  denovo2649_f0  denovo2753_f0  denovo2779_f0  denovo2831_f0  denovo2909_f0  denovo3325_f0  denovo3351_f0  denovo3455_f0  denovo3611_f0  denovo3637_f0  denovo3663_f0  denovo3741_f0  denovo3767_f0  denovo3819_f0  denovo3845_f0  denovo3897_f0  denovo4079_f0  denovo4105_f0  denovo4157_f0  denovo4183_f0  denovo4261_f0  denovo4339_f0  denovo4365_f0  denovo4391_f0  denovo4443_f0  denovo4521_f0  denovo4547_f0  denovo4651_f0  denovo4859_f0  denovo5015_f0  denovo5041_f0  denovo5119_f0  denovo5145_f0  denovo5171_f0  denovo5353_f0  denovo5431_f0  denovo5509_f0  denovo5561_f0  denovo5587_f0  denovo5743_f0  denovo5795_f0  denovo5873_f0  denovo5899_f0  denovo5925_f0  denovo5977_f0  denovo6055_f0  denovo6107_f0  denovo6211_f0  denovo6289_f0  denovo6315_f0  denovo6341_f0  denovo6367_f0  denovo6393_f0  denovo6497_f0  denovo6523_f0  denovo6549_f0  denovo6575_f0  denovo6653_f0  denovo6679_f0  denovo6731_f0  denovo6757_f0  denovo6913_f0  denovo6991_f0  denovo7043_f0  denovo7069_f0  denovo7147_f0  denovo7173_f0  denovo7303_f0  denovo7329_f0  denovo7355_f0  denovo7381_f0  denovo7407_f0  denovo7459_f0  denovo7485_f0  denovo7511_f0  denovo7563_f0  denovo7641_f0  denovo7667_f0  denovo7719_f0  denovo7797_f0  denovo7849_f0  denovo7875_f0  denovo7927_f0  denovo7953_f0  denovo8031_f0  denovo8057_f0  denovo8109_f0  denovo8161_f0  denovo8213_f0  denovo8369_f0  denovo8395_f0  denovo8421_f0  denovo8499_f0  denovo8551_f0  denovo8577_f0  denovo8629_f0  denovo8655_f0  denovo8707_f0  denovo8733_f0  denovo8759_f0  denovo8785_f0  denovo9071_f0  denovo9097_f0  denovo9123_f0  denovo9149_f0  denovo9227_f0  denovo9253_f0  denovo9305_f0  denovo9357_f0  denovo9487_f0  denovo9513_f0  denovo9643_f0  denovo9669_f0  denovo9799_f0  denovo9851_f0  denovo9903_f0  denovo9981_f0  denovo10033_f0 denovo10059_f0 denovo10137_f0 denovo10163_f0 denovo10293_f0 denovo10319_f0 denovo10449_f0 denovo10475_f0 denovo10527_f0 denovo10553_f0 denovo10579_f0 denovo10605_f0 denovo10735_f0 denovo10761_f0 denovo10787_f0 denovo10839_f0 denovo10917_f0 denovo11021_f0 denovo11073_f0 denovo11151_f0 denovo11229_f0 denovo11307_f0 denovo11411_f0 denovo11437_f0 denovo11489_f0 denovo11515_f0 denovo11541_f0 denovo11593_f0 denovo11619_f0 denovo11723_f0 denovo11749_f0 denovo11775_f0 denovo11879_f0 denovo11905_f0 denovo11957_f0 
Callid                        denovo1_f0     denovo53_f0    denovo79_f0    denovo157_f0   denovo183_f0   denovo261_f0   denovo313_f0   denovo365_f0   denovo443_f0   denovo547_f0   denovo677_f0   denovo833_f0   denovo859_f0   denovo885_f0   denovo911_f0   denovo1041_f0  denovo1171_f0  denovo1223_f0  denovo1249_f0  denovo1275_f0  denovo1353_f0  denovo1379_f0  denovo1405_f0  denovo1483_f0  denovo1561_f0  denovo1639_f0  denovo1665_f0  denovo1717_f0  denovo1847_f0  denovo1899_f0  denovo1925_f0  denovo1951_f0  denovo1977_f0  denovo2003_f0  denovo2055_f0  denovo2081_f0  denovo2107_f0  denovo2133_f0  denovo2237_f0  denovo2263_f0  denovo2289_f0  denovo2315_f0  denovo2367_f0  denovo2393_f0  denovo2419_f0  denovo2549_f0  denovo2575_f0  denovo2601_f0  denovo2627_f0  denovo2731_f0  denovo2757_f0  denovo2809_f0  denovo2887_f0  denovo3303_f0  denovo3329_f0  denovo3433_f0  denovo3589_f0  denovo3615_f0  denovo3641_f0  denovo3719_f0  denovo3745_f0  denovo3797_f0  denovo3823_f0  denovo3875_f0  denovo4057_f0  denovo4083_f0  denovo4135_f0  denovo4161_f0  denovo4239_f0  denovo4317_f0  denovo4343_f0  denovo4369_f0  denovo4421_f0  denovo4499_f0  denovo4525_f0  denovo4629_f0  denovo4837_f0  denovo4993_f0  denovo5019_f0  denovo5097_f0  denovo5123_f0  denovo5149_f0  denovo5331_f0  denovo5409_f0  denovo5487_f0  denovo5539_f0  denovo5565_f0  denovo5721_f0  denovo5773_f0  denovo5851_f0  denovo5877_f0  denovo5903_f0  denovo5955_f0  denovo6033_f0  denovo6085_f0  denovo6189_f0  denovo6267_f0  denovo6293_f0  denovo6319_f0  denovo6345_f0  denovo6371_f0  denovo6475_f0  denovo6501_f0  denovo6527_f0  denovo6553_f0  denovo6631_f0  denovo6657_f0  denovo6709_f0  denovo6735_f0  denovo6891_f0  denovo6969_f0  denovo7021_f0  denovo7047_f0  denovo7125_f0  denovo7151_f0  denovo7281_f0  denovo7307_f0  denovo7333_f0  denovo7359_f0  denovo7385_f0  denovo7437_f0  denovo7463_f0  denovo7489_f0  denovo7541_f0  denovo7619_f0  denovo7645_f0  denovo7697_f0  denovo7775_f0  denovo7827_f0  denovo7853_f0  denovo7905_f0  denovo7931_f0  denovo8009_f0  denovo8035_f0  denovo8087_f0  denovo8139_f0  denovo8191_f0  denovo8347_f0  denovo8373_f0  denovo8399_f0  denovo8477_f0  denovo8529_f0  denovo8555_f0  denovo8607_f0  denovo8633_f0  denovo8685_f0  denovo8711_f0  denovo8737_f0  denovo8763_f0  denovo9049_f0  denovo9075_f0  denovo9101_f0  denovo9127_f0  denovo9205_f0  denovo9231_f0  denovo9283_f0  denovo9335_f0  denovo9465_f0  denovo9491_f0  denovo9621_f0  denovo9647_f0  denovo9777_f0  denovo9829_f0  denovo9881_f0  denovo9959_f0  denovo10011_f0 denovo10037_f0 denovo10115_f0 denovo10141_f0 denovo10271_f0 denovo10297_f0 denovo10427_f0 denovo10453_f0 denovo10505_f0 denovo10531_f0 denovo10557_f0 denovo10583_f0 denovo10713_f0 denovo10739_f0 denovo10765_f0 denovo10817_f0 denovo10895_f0 denovo10999_f0 denovo11051_f0 denovo11129_f0 denovo11207_f0 denovo11285_f0 denovo11389_f0 denovo11415_f0 denovo11467_f0 denovo11493_f0 denovo11519_f0 denovo11571_f0 denovo11597_f0 denovo11701_f0 denovo11727_f0 denovo11753_f0 denovo11857_f0 denovo11883_f0 denovo11935_f0 
FG120070B                     denovo8_f0     denovo60_f0    denovo86_f0    denovo164_f0   denovo190_f0   denovo268_f0   denovo320_f0   denovo372_f0   denovo450_f0   denovo554_f0   denovo684_f0   denovo840_f0   denovo866_f0   denovo892_f0   denovo918_f0   denovo1048_f0  denovo1178_f0  denovo1230_f0  denovo1256_f0  denovo1282_f0  denovo1360_f0  denovo1386_f0  denovo1412_f0  denovo1490_f0  denovo1568_f0  denovo1646_f0  denovo1672_f0  denovo1724_f0  denovo1854_f0  denovo1906_f0  denovo1932_f0  denovo1958_f0  denovo1984_f0  denovo2010_f0  denovo2062_f0  denovo2088_f0  denovo2114_f0  denovo2140_f0  denovo2244_f0  denovo2270_f0  denovo2296_f0  denovo2322_f0  denovo2374_f0  denovo2400_f0  denovo2426_f0  denovo2556_f0  denovo2582_f0  denovo2608_f0  denovo2634_f0  denovo2738_f0  denovo2764_f0  denovo2816_f0  denovo2894_f0  denovo3310_f0  denovo3336_f0  denovo3440_f0  denovo3596_f0  denovo3622_f0  denovo3648_f0  denovo3726_f0  denovo3752_f0  denovo3804_f0  denovo3830_f0  denovo3882_f0  denovo4064_f0  denovo4090_f0  denovo4142_f0  denovo4168_f0  denovo4246_f0  denovo4324_f0  denovo4350_f0  denovo4376_f0  denovo4428_f0  denovo4506_f0  denovo4532_f0  denovo4636_f0  denovo4844_f0  denovo5000_f0  denovo5026_f0  denovo5104_f0  denovo5130_f0  denovo5156_f0  denovo5338_f0  denovo5416_f0  denovo5494_f0  denovo5546_f0  denovo5572_f0  denovo5728_f0  denovo5780_f0  denovo5858_f0  denovo5884_f0  denovo5910_f0  denovo5962_f0  denovo6040_f0  denovo6092_f0  denovo6196_f0  denovo6274_f0  denovo6300_f0  denovo6326_f0  denovo6352_f0  denovo6378_f0  denovo6482_f0  denovo6508_f0  denovo6534_f0  denovo6560_f0  denovo6638_f0  denovo6664_f0  denovo6716_f0  denovo6742_f0  denovo6898_f0  denovo6976_f0  denovo7028_f0  denovo7054_f0  denovo7132_f0  denovo7158_f0  denovo7288_f0  denovo7314_f0  denovo7340_f0  denovo7366_f0  denovo7392_f0  denovo7444_f0  denovo7470_f0  denovo7496_f0  denovo7548_f0  denovo7626_f0  denovo7652_f0  denovo7704_f0  denovo7782_f0  denovo7834_f0  denovo7860_f0  denovo7912_f0  denovo7938_f0  denovo8016_f0  denovo8042_f0  denovo8094_f0  denovo8146_f0  denovo8198_f0  denovo8354_f0  denovo8380_f0  denovo8406_f0  denovo8484_f0  denovo8536_f0  denovo8562_f0  denovo8614_f0  denovo8640_f0  denovo8692_f0  denovo8718_f0  denovo8744_f0  denovo8770_f0  denovo9056_f0  denovo9082_f0  denovo9108_f0  denovo9134_f0  denovo9212_f0  denovo9238_f0  denovo9290_f0  denovo9342_f0  denovo9472_f0  denovo9498_f0  denovo9628_f0  denovo9654_f0  denovo9784_f0  denovo9836_f0  denovo9888_f0  denovo9966_f0  denovo10018_f0 denovo10044_f0 denovo10122_f0 denovo10148_f0 denovo10278_f0 denovo10304_f0 denovo10434_f0 denovo10460_f0 denovo10512_f0 denovo10538_f0 denovo10564_f0 denovo10590_f0 denovo10720_f0 denovo10746_f0 denovo10772_f0 denovo10824_f0 denovo10902_f0 denovo11006_f0 denovo11058_f0 denovo11136_f0 denovo11214_f0 denovo11292_f0 denovo11396_f0 denovo11422_f0 denovo11474_f0 denovo11500_f0 denovo11526_f0 denovo11578_f0 denovo11604_f0 denovo11708_f0 denovo11734_f0 denovo11760_f0 denovo11864_f0 denovo11890_f0 denovo11942_f0 
SW130007                      denovo22_f0    denovo74_f0    denovo100_f0   denovo178_f0   denovo204_f0   denovo282_f0   denovo334_f0   denovo386_f0   denovo464_f0   denovo568_f0   denovo698_f0   denovo854_f0   denovo880_f0   denovo906_f0   denovo932_f0   denovo1062_f0  denovo1192_f0  denovo1244_f0  denovo1270_f0  denovo1296_f0  denovo1374_f0  denovo1400_f0  denovo1426_f0  denovo1504_f0  denovo1582_f0  denovo1660_f0  denovo1686_f0  denovo1738_f0  denovo1868_f0  denovo1920_f0  denovo1946_f0  denovo1972_f0  denovo1998_f0  denovo2024_f0  denovo2076_f0  denovo2102_f0  denovo2128_f0  denovo2154_f0  denovo2258_f0  denovo2284_f0  denovo2310_f0  denovo2336_f0  denovo2388_f0  denovo2414_f0  denovo2440_f0  denovo2570_f0  denovo2596_f0  denovo2622_f0  denovo2648_f0  denovo2752_f0  denovo2778_f0  denovo2830_f0  denovo2908_f0  denovo3324_f0  denovo3350_f0  denovo3454_f0  denovo3610_f0  denovo3636_f0  denovo3662_f0  denovo3740_f0  denovo3766_f0  denovo3818_f0  denovo3844_f0  denovo3896_f0  denovo4078_f0  denovo4104_f0  denovo4156_f0  denovo4182_f0  denovo4260_f0  denovo4338_f0  denovo4364_f0  denovo4390_f0  denovo4442_f0  denovo4520_f0  denovo4546_f0  denovo4650_f0  denovo4858_f0  denovo5014_f0  denovo5040_f0  denovo5118_f0  denovo5144_f0  denovo5170_f0  denovo5352_f0  denovo5430_f0  denovo5508_f0  denovo5560_f0  denovo5586_f0  denovo5742_f0  denovo5794_f0  denovo5872_f0  denovo5898_f0  denovo5924_f0  denovo5976_f0  denovo6054_f0  denovo6106_f0  denovo6210_f0  denovo6288_f0  denovo6314_f0  denovo6340_f0  denovo6366_f0  denovo6392_f0  denovo6496_f0  denovo6522_f0  denovo6548_f0  denovo6574_f0  denovo6652_f0  denovo6678_f0  denovo6730_f0  denovo6756_f0  denovo6912_f0  denovo6990_f0  denovo7042_f0  denovo7068_f0  denovo7146_f0  denovo7172_f0  denovo7302_f0  denovo7328_f0  denovo7354_f0  denovo7380_f0  denovo7406_f0  denovo7458_f0  denovo7484_f0  denovo7510_f0  denovo7562_f0  denovo7640_f0  denovo7666_f0  denovo7718_f0  denovo7796_f0  denovo7848_f0  denovo7874_f0  denovo7926_f0  denovo7952_f0  denovo8030_f0  denovo8056_f0  denovo8108_f0  denovo8160_f0  denovo8212_f0  denovo8368_f0  denovo8394_f0  denovo8420_f0  denovo8498_f0  denovo8550_f0  denovo8576_f0  denovo8628_f0  denovo8654_f0  denovo8706_f0  denovo8732_f0  denovo8758_f0  denovo8784_f0  denovo9070_f0  denovo9096_f0  denovo9122_f0  denovo9148_f0  denovo9226_f0  denovo9252_f0  denovo9304_f0  denovo9356_f0  denovo9486_f0  denovo9512_f0  denovo9642_f0  denovo9668_f0  denovo9798_f0  denovo9850_f0  denovo9902_f0  denovo9980_f0  denovo10032_f0 denovo10058_f0 denovo10136_f0 denovo10162_f0 denovo10292_f0 denovo10318_f0 denovo10448_f0 denovo10474_f0 denovo10526_f0 denovo10552_f0 denovo10578_f0 denovo10604_f0 denovo10734_f0 denovo10760_f0 denovo10786_f0 denovo10838_f0 denovo10916_f0 denovo11020_f0 denovo11072_f0 denovo11150_f0 denovo11228_f0 denovo11306_f0 denovo11410_f0 denovo11436_f0 denovo11488_f0 denovo11514_f0 denovo11540_f0 denovo11592_f0 denovo11618_f0 denovo11722_f0 denovo11748_f0 denovo11774_f0 denovo11878_f0 denovo11904_f0 denovo11956_f0 
GNV120032                     denovo14_f0    denovo66_f0    denovo92_f0    denovo170_f0   denovo196_f0   denovo274_f0   denovo326_f0   denovo378_f0   denovo456_f0   denovo560_f0   denovo690_f0   denovo846_f0   denovo872_f0   denovo898_f0   denovo924_f0   denovo1054_f0  denovo1184_f0  denovo1236_f0  denovo1262_f0  denovo1288_f0  denovo1366_f0  denovo1392_f0  denovo1418_f0  denovo1496_f0  denovo1574_f0  denovo1652_f0  denovo1678_f0  denovo1730_f0  denovo1860_f0  denovo1912_f0  denovo1938_f0  denovo1964_f0  denovo1990_f0  denovo2016_f0  denovo2068_f0  denovo2094_f0  denovo2120_f0  denovo2146_f0  denovo2250_f0  denovo2276_f0  denovo2302_f0  denovo2328_f0  denovo2380_f0  denovo2406_f0  denovo2432_f0  denovo2562_f0  denovo2588_f0  denovo2614_f0  denovo2640_f0  denovo2744_f0  denovo2770_f0  denovo2822_f0  denovo2900_f0  denovo3316_f0  denovo3342_f0  denovo3446_f0  denovo3602_f0  denovo3628_f0  denovo3654_f0  denovo3732_f0  denovo3758_f0  denovo3810_f0  denovo3836_f0  denovo3888_f0  denovo4070_f0  denovo4096_f0  denovo4148_f0  denovo4174_f0  denovo4252_f0  denovo4330_f0  denovo4356_f0  denovo4382_f0  denovo4434_f0  denovo4512_f0  denovo4538_f0  denovo4642_f0  denovo4850_f0  denovo5006_f0  denovo5032_f0  denovo5110_f0  denovo5136_f0  denovo5162_f0  denovo5344_f0  denovo5422_f0  denovo5500_f0  denovo5552_f0  denovo5578_f0  denovo5734_f0  denovo5786_f0  denovo5864_f0  denovo5890_f0  denovo5916_f0  denovo5968_f0  denovo6046_f0  denovo6098_f0  denovo6202_f0  denovo6280_f0  denovo6306_f0  denovo6332_f0  denovo6358_f0  denovo6384_f0  denovo6488_f0  denovo6514_f0  denovo6540_f0  denovo6566_f0  denovo6644_f0  denovo6670_f0  denovo6722_f0  denovo6748_f0  denovo6904_f0  denovo6982_f0  denovo7034_f0  denovo7060_f0  denovo7138_f0  denovo7164_f0  denovo7294_f0  denovo7320_f0  denovo7346_f0  denovo7372_f0  denovo7398_f0  denovo7450_f0  denovo7476_f0  denovo7502_f0  denovo7554_f0  denovo7632_f0  denovo7658_f0  denovo7710_f0  denovo7788_f0  denovo7840_f0  denovo7866_f0  denovo7918_f0  denovo7944_f0  denovo8022_f0  denovo8048_f0  denovo8100_f0  denovo8152_f0  denovo8204_f0  denovo8360_f0  denovo8386_f0  denovo8412_f0  denovo8490_f0  denovo8542_f0  denovo8568_f0  denovo8620_f0  denovo8646_f0  denovo8698_f0  denovo8724_f0  denovo8750_f0  denovo8776_f0  denovo9062_f0  denovo9088_f0  denovo9114_f0  denovo9140_f0  denovo9218_f0  denovo9244_f0  denovo9296_f0  denovo9348_f0  denovo9478_f0  denovo9504_f0  denovo9634_f0  denovo9660_f0  denovo9790_f0  denovo9842_f0  denovo9894_f0  denovo9972_f0  denovo10024_f0 denovo10050_f0 denovo10128_f0 denovo10154_f0 denovo10284_f0 denovo10310_f0 denovo10440_f0 denovo10466_f0 denovo10518_f0 denovo10544_f0 denovo10570_f0 denovo10596_f0 denovo10726_f0 denovo10752_f0 denovo10778_f0 denovo10830_f0 denovo10908_f0 denovo11012_f0 denovo11064_f0 denovo11142_f0 denovo11220_f0 denovo11298_f0 denovo11402_f0 denovo11428_f0 denovo11480_f0 denovo11506_f0 denovo11532_f0 denovo11584_f0 denovo11610_f0 denovo11714_f0 denovo11740_f0 denovo11766_f0 denovo11870_f0 denovo11896_f0 denovo11948_f0 
PXYLO                         denovo18_f0    denovo70_f0    denovo96_f0    denovo174_f0   denovo200_f0   denovo278_f0   denovo330_f0   denovo382_f0   denovo460_f0   denovo564_f0   denovo694_f0   denovo850_f0   denovo876_f0   denovo902_f0   denovo928_f0   denovo1058_f0  denovo1188_f0  denovo1240_f0  denovo1266_f0  denovo1292_f0  denovo1370_f0  denovo1396_f0  denovo1422_f0  denovo1500_f0  denovo1578_f0  denovo1656_f0  denovo1682_f0  denovo1734_f0  denovo1864_f0  denovo1916_f0  denovo1942_f0  denovo1968_f0  denovo1994_f0  denovo2020_f0  denovo2072_f0  denovo2098_f0  denovo2124_f0  denovo2150_f0  denovo2254_f0  denovo2280_f0  denovo2306_f0  denovo2332_f0  denovo2384_f0  denovo2410_f0  denovo2436_f0  denovo2566_f0  denovo2592_f0  denovo2618_f0  denovo2644_f0  denovo2748_f0  denovo2774_f0  denovo2826_f0  denovo2904_f0  denovo3320_f0  denovo3346_f0  denovo3450_f0  denovo3606_f0  denovo3632_f0  denovo3658_f0  denovo3736_f0  denovo3762_f0  denovo3814_f0  denovo3840_f0  denovo3892_f0  denovo4074_f0  denovo4100_f0  denovo4152_f0  denovo4178_f0  denovo4256_f0  denovo4334_f0  denovo4360_f0  denovo4386_f0  denovo4438_f0  denovo4516_f0  denovo4542_f0  denovo4646_f0  denovo4854_f0  denovo5010_f0  denovo5036_f0  denovo5114_f0  denovo5140_f0  denovo5166_f0  denovo5348_f0  denovo5426_f0  denovo5504_f0  denovo5556_f0  denovo5582_f0  denovo5738_f0  denovo5790_f0  denovo5868_f0  denovo5894_f0  denovo5920_f0  denovo5972_f0  denovo6050_f0  denovo6102_f0  denovo6206_f0  denovo6284_f0  denovo6310_f0  denovo6336_f0  denovo6362_f0  denovo6388_f0  denovo6492_f0  denovo6518_f0  denovo6544_f0  denovo6570_f0  denovo6648_f0  denovo6674_f0  denovo6726_f0  denovo6752_f0  denovo6908_f0  denovo6986_f0  denovo7038_f0  denovo7064_f0  denovo7142_f0  denovo7168_f0  denovo7298_f0  denovo7324_f0  denovo7350_f0  denovo7376_f0  denovo7402_f0  denovo7454_f0  denovo7480_f0  denovo7506_f0  denovo7558_f0  denovo7636_f0  denovo7662_f0  denovo7714_f0  denovo7792_f0  denovo7844_f0  denovo7870_f0  denovo7922_f0  denovo7948_f0  denovo8026_f0  denovo8052_f0  denovo8104_f0  denovo8156_f0  denovo8208_f0  denovo8364_f0  denovo8390_f0  denovo8416_f0  denovo8494_f0  denovo8546_f0  denovo8572_f0  denovo8624_f0  denovo8650_f0  denovo8702_f0  denovo8728_f0  denovo8754_f0  denovo8780_f0  denovo9066_f0  denovo9092_f0  denovo9118_f0  denovo9144_f0  denovo9222_f0  denovo9248_f0  denovo9300_f0  denovo9352_f0  denovo9482_f0  denovo9508_f0  denovo9638_f0  denovo9664_f0  denovo9794_f0  denovo9846_f0  denovo9898_f0  denovo9976_f0  denovo10028_f0 denovo10054_f0 denovo10132_f0 denovo10158_f0 denovo10288_f0 denovo10314_f0 denovo10444_f0 denovo10470_f0 denovo10522_f0 denovo10548_f0 denovo10574_f0 denovo10600_f0 denovo10730_f0 denovo10756_f0 denovo10782_f0 denovo10834_f0 denovo10912_f0 denovo11016_f0 denovo11068_f0 denovo11146_f0 denovo11224_f0 denovo11302_f0 denovo11406_f0 denovo11432_f0 denovo11484_f0 denovo11510_f0 denovo11536_f0 denovo11588_f0 denovo11614_f0 denovo11718_f0 denovo11744_f0 denovo11770_f0 denovo11874_f0 denovo11900_f0 denovo11952_f0 
FG120079                      denovo11_f0    denovo63_f0    denovo89_f0    denovo167_f0   denovo193_f0   denovo271_f0   denovo323_f0   denovo375_f0   denovo453_f0   denovo557_f0   denovo687_f0   denovo843_f0   denovo869_f0   denovo895_f0   denovo921_f0   denovo1051_f0  denovo1181_f0  denovo1233_f0  denovo1259_f0  denovo1285_f0  denovo1363_f0  denovo1389_f0  denovo1415_f0  denovo1493_f0  denovo1571_f0  denovo1649_f0  denovo1675_f0  denovo1727_f0  denovo1857_f0  denovo1909_f0  denovo1935_f0  denovo1961_f0  denovo1987_f0  denovo2013_f0  denovo2065_f0  denovo2091_f0  denovo2117_f0  denovo2143_f0  denovo2247_f0  denovo2273_f0  denovo2299_f0  denovo2325_f0  denovo2377_f0  denovo2403_f0  denovo2429_f0  denovo2559_f0  denovo2585_f0  denovo2611_f0  denovo2637_f0  denovo2741_f0  denovo2767_f0  denovo2819_f0  denovo2897_f0  denovo3313_f0  denovo3339_f0  denovo3443_f0  denovo3599_f0  denovo3625_f0  denovo3651_f0  denovo3729_f0  denovo3755_f0  denovo3807_f0  denovo3833_f0  denovo3885_f0  denovo4067_f0  denovo4093_f0  denovo4145_f0  denovo4171_f0  denovo4249_f0  denovo4327_f0  denovo4353_f0  denovo4379_f0  denovo4431_f0  denovo4509_f0  denovo4535_f0  denovo4639_f0  denovo4847_f0  denovo5003_f0  denovo5029_f0  denovo5107_f0  denovo5133_f0  denovo5159_f0  denovo5341_f0  denovo5419_f0  denovo5497_f0  denovo5549_f0  denovo5575_f0  denovo5731_f0  denovo5783_f0  denovo5861_f0  denovo5887_f0  denovo5913_f0  denovo5965_f0  denovo6043_f0  denovo6095_f0  denovo6199_f0  denovo6277_f0  denovo6303_f0  denovo6329_f0  denovo6355_f0  denovo6381_f0  denovo6485_f0  denovo6511_f0  denovo6537_f0  denovo6563_f0  denovo6641_f0  denovo6667_f0  denovo6719_f0  denovo6745_f0  denovo6901_f0  denovo6979_f0  denovo7031_f0  denovo7057_f0  denovo7135_f0  denovo7161_f0  denovo7291_f0  denovo7317_f0  denovo7343_f0  denovo7369_f0  denovo7395_f0  denovo7447_f0  denovo7473_f0  denovo7499_f0  denovo7551_f0  denovo7629_f0  denovo7655_f0  denovo7707_f0  denovo7785_f0  denovo7837_f0  denovo7863_f0  denovo7915_f0  denovo7941_f0  denovo8019_f0  denovo8045_f0  denovo8097_f0  denovo8149_f0  denovo8201_f0  denovo8357_f0  denovo8383_f0  denovo8409_f0  denovo8487_f0  denovo8539_f0  denovo8565_f0  denovo8617_f0  denovo8643_f0  denovo8695_f0  denovo8721_f0  denovo8747_f0  denovo8773_f0  denovo9059_f0  denovo9085_f0  denovo9111_f0  denovo9137_f0  denovo9215_f0  denovo9241_f0  denovo9293_f0  denovo9345_f0  denovo9475_f0  denovo9501_f0  denovo9631_f0  denovo9657_f0  denovo9787_f0  denovo9839_f0  denovo9891_f0  denovo9969_f0  denovo10021_f0 denovo10047_f0 denovo10125_f0 denovo10151_f0 denovo10281_f0 denovo10307_f0 denovo10437_f0 denovo10463_f0 denovo10515_f0 denovo10541_f0 denovo10567_f0 denovo10593_f0 denovo10723_f0 denovo10749_f0 denovo10775_f0 denovo10827_f0 denovo10905_f0 denovo11009_f0 denovo11061_f0 denovo11139_f0 denovo11217_f0 denovo11295_f0 denovo11399_f0 denovo11425_f0 denovo11477_f0 denovo11503_f0 denovo11529_f0 denovo11581_f0 denovo11607_f0 denovo11711_f0 denovo11737_f0 denovo11763_f0 denovo11867_f0 denovo11893_f0 denovo11945_f0 
SRR850324                     denovo21_f0    denovo73_f0    denovo99_f0    denovo177_f0   denovo203_f0   denovo281_f0   denovo333_f0   denovo385_f0   denovo463_f0   denovo567_f0   denovo697_f0   denovo853_f0   denovo879_f0   denovo905_f0   denovo931_f0   denovo1061_f0  denovo1191_f0  denovo1243_f0  denovo1269_f0  denovo1295_f0  denovo1373_f0  denovo1399_f0  denovo1425_f0  denovo1503_f0  denovo1581_f0  denovo1659_f0  denovo1685_f0  denovo1737_f0  denovo1867_f0  denovo1919_f0  denovo1945_f0  denovo1971_f0  denovo1997_f0  denovo2023_f0  denovo2075_f0  denovo2101_f0  denovo2127_f0  denovo2153_f0  denovo2257_f0  denovo2283_f0  denovo2309_f0  denovo2335_f0  denovo2387_f0  denovo2413_f0  denovo2439_f0  denovo2569_f0  denovo2595_f0  denovo2621_f0  denovo2647_f0  denovo2751_f0  denovo2777_f0  denovo2829_f0  denovo2907_f0  denovo3323_f0  denovo3349_f0  denovo3453_f0  denovo3609_f0  denovo3635_f0  denovo3661_f0  denovo3739_f0  denovo3765_f0  denovo3817_f0  denovo3843_f0  denovo3895_f0  denovo4077_f0  denovo4103_f0  denovo4155_f0  denovo4181_f0  denovo4259_f0  denovo4337_f0  denovo4363_f0  denovo4389_f0  denovo4441_f0  denovo4519_f0  denovo4545_f0  denovo4649_f0  denovo4857_f0  denovo5013_f0  denovo5039_f0  denovo5117_f0  denovo5143_f0  denovo5169_f0  denovo5351_f0  denovo5429_f0  denovo5507_f0  denovo5559_f0  denovo5585_f0  denovo5741_f0  denovo5793_f0  denovo5871_f0  denovo5897_f0  denovo5923_f0  denovo5975_f0  denovo6053_f0  denovo6105_f0  denovo6209_f0  denovo6287_f0  denovo6313_f0  denovo6339_f0  denovo6365_f0  denovo6391_f0  denovo6495_f0  denovo6521_f0  denovo6547_f0  denovo6573_f0  denovo6651_f0  denovo6677_f0  denovo6729_f0  denovo6755_f0  denovo6911_f0  denovo6989_f0  denovo7041_f0  denovo7067_f0  denovo7145_f0  denovo7171_f0  denovo7301_f0  denovo7327_f0  denovo7353_f0  denovo7379_f0  denovo7405_f0  denovo7457_f0  denovo7483_f0  denovo7509_f0  denovo7561_f0  denovo7639_f0  denovo7665_f0  denovo7717_f0  denovo7795_f0  denovo7847_f0  denovo7873_f0  denovo7925_f0  denovo7951_f0  denovo8029_f0  denovo8055_f0  denovo8107_f0  denovo8159_f0  denovo8211_f0  denovo8367_f0  denovo8393_f0  denovo8419_f0  denovo8497_f0  denovo8549_f0  denovo8575_f0  denovo8627_f0  denovo8653_f0  denovo8705_f0  denovo8731_f0  denovo8757_f0  denovo8783_f0  denovo9069_f0  denovo9095_f0  denovo9121_f0  denovo9147_f0  denovo9225_f0  denovo9251_f0  denovo9303_f0  denovo9355_f0  denovo9485_f0  denovo9511_f0  denovo9641_f0  denovo9667_f0  denovo9797_f0  denovo9849_f0  denovo9901_f0  denovo9979_f0  denovo10031_f0 denovo10057_f0 denovo10135_f0 denovo10161_f0 denovo10291_f0 denovo10317_f0 denovo10447_f0 denovo10473_f0 denovo10525_f0 denovo10551_f0 denovo10577_f0 denovo10603_f0 denovo10733_f0 denovo10759_f0 denovo10785_f0 denovo10837_f0 denovo10915_f0 denovo11019_f0 denovo11071_f0 denovo11149_f0 denovo11227_f0 denovo11305_f0 denovo11409_f0 denovo11435_f0 denovo11487_f0 denovo11513_f0 denovo11539_f0 denovo11591_f0 denovo11617_f0 denovo11721_f0 denovo11747_f0 denovo11773_f0 denovo11877_f0 denovo11903_f0 denovo11955_f0 
FG120055B                     denovo7_f0     denovo59_f0    denovo85_f0    denovo163_f0   denovo189_f0   denovo267_f0   denovo319_f0   denovo371_f0   denovo449_f0   denovo553_f0   denovo683_f0   denovo839_f0   denovo865_f0   denovo891_f0   denovo917_f0   denovo1047_f0  denovo1177_f0  denovo1229_f0  denovo1255_f0  denovo1281_f0  denovo1359_f0  denovo1385_f0  denovo1411_f0  denovo1489_f0  denovo1567_f0  denovo1645_f0  denovo1671_f0  denovo1723_f0  denovo1853_f0  denovo1905_f0  denovo1931_f0  denovo1957_f0  denovo1983_f0  denovo2009_f0  denovo2061_f0  denovo2087_f0  denovo2113_f0  denovo2139_f0  denovo2243_f0  denovo2269_f0  denovo2295_f0  denovo2321_f0  denovo2373_f0  denovo2399_f0  denovo2425_f0  denovo2555_f0  denovo2581_f0  denovo2607_f0  denovo2633_f0  denovo2737_f0  denovo2763_f0  denovo2815_f0  denovo2893_f0  denovo3309_f0  denovo3335_f0  denovo3439_f0  denovo3595_f0  denovo3621_f0  denovo3647_f0  denovo3725_f0  denovo3751_f0  denovo3803_f0  denovo3829_f0  denovo3881_f0  denovo4063_f0  denovo4089_f0  denovo4141_f0  denovo4167_f0  denovo4245_f0  denovo4323_f0  denovo4349_f0  denovo4375_f0  denovo4427_f0  denovo4505_f0  denovo4531_f0  denovo4635_f0  denovo4843_f0  denovo4999_f0  denovo5025_f0  denovo5103_f0  denovo5129_f0  denovo5155_f0  denovo5337_f0  denovo5415_f0  denovo5493_f0  denovo5545_f0  denovo5571_f0  denovo5727_f0  denovo5779_f0  denovo5857_f0  denovo5883_f0  denovo5909_f0  denovo5961_f0  denovo6039_f0  denovo6091_f0  denovo6195_f0  denovo6273_f0  denovo6299_f0  denovo6325_f0  denovo6351_f0  denovo6377_f0  denovo6481_f0  denovo6507_f0  denovo6533_f0  denovo6559_f0  denovo6637_f0  denovo6663_f0  denovo6715_f0  denovo6741_f0  denovo6897_f0  denovo6975_f0  denovo7027_f0  denovo7053_f0  denovo7131_f0  denovo7157_f0  denovo7287_f0  denovo7313_f0  denovo7339_f0  denovo7365_f0  denovo7391_f0  denovo7443_f0  denovo7469_f0  denovo7495_f0  denovo7547_f0  denovo7625_f0  denovo7651_f0  denovo7703_f0  denovo7781_f0  denovo7833_f0  denovo7859_f0  denovo7911_f0  denovo7937_f0  denovo8015_f0  denovo8041_f0  denovo8093_f0  denovo8145_f0  denovo8197_f0  denovo8353_f0  denovo8379_f0  denovo8405_f0  denovo8483_f0  denovo8535_f0  denovo8561_f0  denovo8613_f0  denovo8639_f0  denovo8691_f0  denovo8717_f0  denovo8743_f0  denovo8769_f0  denovo9055_f0  denovo9081_f0  denovo9107_f0  denovo9133_f0  denovo9211_f0  denovo9237_f0  denovo9289_f0  denovo9341_f0  denovo9471_f0  denovo9497_f0  denovo9627_f0  denovo9653_f0  denovo9783_f0  denovo9835_f0  denovo9887_f0  denovo9965_f0  denovo10017_f0 denovo10043_f0 denovo10121_f0 denovo10147_f0 denovo10277_f0 denovo10303_f0 denovo10433_f0 denovo10459_f0 denovo10511_f0 denovo10537_f0 denovo10563_f0 denovo10589_f0 denovo10719_f0 denovo10745_f0 denovo10771_f0 denovo10823_f0 denovo10901_f0 denovo11005_f0 denovo11057_f0 denovo11135_f0 denovo11213_f0 denovo11291_f0 denovo11395_f0 denovo11421_f0 denovo11473_f0 denovo11499_f0 denovo11525_f0 denovo11577_f0 denovo11603_f0 denovo11707_f0 denovo11733_f0 denovo11759_f0 denovo11863_f0 denovo11889_f0 denovo11941_f0 
FG120122                      denovo12_f0    denovo64_f0    denovo90_f0    denovo168_f0   denovo194_f0   denovo272_f0   denovo324_f0   denovo376_f0   denovo454_f0   denovo558_f0   denovo688_f0   denovo844_f0   denovo870_f0   denovo896_f0   denovo922_f0   denovo1052_f0  denovo1182_f0  denovo1234_f0  denovo1260_f0  denovo1286_f0  denovo1364_f0  denovo1390_f0  denovo1416_f0  denovo1494_f0  denovo1572_f0  denovo1650_f0  denovo1676_f0  denovo1728_f0  denovo1858_f0  denovo1910_f0  denovo1936_f0  denovo1962_f0  denovo1988_f0  denovo2014_f0  denovo2066_f0  denovo2092_f0  denovo2118_f0  denovo2144_f0  denovo2248_f0  denovo2274_f0  denovo2300_f0  denovo2326_f0  denovo2378_f0  denovo2404_f0  denovo2430_f0  denovo2560_f0  denovo2586_f0  denovo2612_f0  denovo2638_f0  denovo2742_f0  denovo2768_f0  denovo2820_f0  denovo2898_f0  denovo3314_f0  denovo3340_f0  denovo3444_f0  denovo3600_f0  denovo3626_f0  denovo3652_f0  denovo3730_f0  denovo3756_f0  denovo3808_f0  denovo3834_f0  denovo3886_f0  denovo4068_f0  denovo4094_f0  denovo4146_f0  denovo4172_f0  denovo4250_f0  denovo4328_f0  denovo4354_f0  denovo4380_f0  denovo4432_f0  denovo4510_f0  denovo4536_f0  denovo4640_f0  denovo4848_f0  denovo5004_f0  denovo5030_f0  denovo5108_f0  denovo5134_f0  denovo5160_f0  denovo5342_f0  denovo5420_f0  denovo5498_f0  denovo5550_f0  denovo5576_f0  denovo5732_f0  denovo5784_f0  denovo5862_f0  denovo5888_f0  denovo5914_f0  denovo5966_f0  denovo6044_f0  denovo6096_f0  denovo6200_f0  denovo6278_f0  denovo6304_f0  denovo6330_f0  denovo6356_f0  denovo6382_f0  denovo6486_f0  denovo6512_f0  denovo6538_f0  denovo6564_f0  denovo6642_f0  denovo6668_f0  denovo6720_f0  denovo6746_f0  denovo6902_f0  denovo6980_f0  denovo7032_f0  denovo7058_f0  denovo7136_f0  denovo7162_f0  denovo7292_f0  denovo7318_f0  denovo7344_f0  denovo7370_f0  denovo7396_f0  denovo7448_f0  denovo7474_f0  denovo7500_f0  denovo7552_f0  denovo7630_f0  denovo7656_f0  denovo7708_f0  denovo7786_f0  denovo7838_f0  denovo7864_f0  denovo7916_f0  denovo7942_f0  denovo8020_f0  denovo8046_f0  denovo8098_f0  denovo8150_f0  denovo8202_f0  denovo8358_f0  denovo8384_f0  denovo8410_f0  denovo8488_f0  denovo8540_f0  denovo8566_f0  denovo8618_f0  denovo8644_f0  denovo8696_f0  denovo8722_f0  denovo8748_f0  denovo8774_f0  denovo9060_f0  denovo9086_f0  denovo9112_f0  denovo9138_f0  denovo9216_f0  denovo9242_f0  denovo9294_f0  denovo9346_f0  denovo9476_f0  denovo9502_f0  denovo9632_f0  denovo9658_f0  denovo9788_f0  denovo9840_f0  denovo9892_f0  denovo9970_f0  denovo10022_f0 denovo10048_f0 denovo10126_f0 denovo10152_f0 denovo10282_f0 denovo10308_f0 denovo10438_f0 denovo10464_f0 denovo10516_f0 denovo10542_f0 denovo10568_f0 denovo10594_f0 denovo10724_f0 denovo10750_f0 denovo10776_f0 denovo10828_f0 denovo10906_f0 denovo11010_f0 denovo11062_f0 denovo11140_f0 denovo11218_f0 denovo11296_f0 denovo11400_f0 denovo11426_f0 denovo11478_f0 denovo11504_f0 denovo11530_f0 denovo11582_f0 denovo11608_f0 denovo11712_f0 denovo11738_f0 denovo11764_f0 denovo11868_f0 denovo11894_f0 denovo11946_f0 
FG120024                      denovo4_f0     denovo56_f0    denovo82_f0    denovo160_f0   denovo186_f0   denovo264_f0   denovo316_f0   denovo368_f0   denovo446_f0   denovo550_f0   denovo680_f0   denovo836_f0   denovo862_f0   denovo888_f0   denovo914_f0   denovo1044_f0  denovo1174_f0  denovo1226_f0  denovo1252_f0  denovo1278_f0  denovo1356_f0  denovo1382_f0  denovo1408_f0  denovo1486_f0  denovo1564_f0  denovo1642_f0  denovo1668_f0  denovo1720_f0  denovo1850_f0  denovo1902_f0  denovo1928_f0  denovo1954_f0  denovo1980_f0  denovo2006_f0  denovo2058_f0  denovo2084_f0  denovo2110_f0  denovo2136_f0  denovo2240_f0  denovo2266_f0  denovo2292_f0  denovo2318_f0  denovo2370_f0  denovo2396_f0  denovo2422_f0  denovo2552_f0  denovo2578_f0  denovo2604_f0  denovo2630_f0  denovo2734_f0  denovo2760_f0  denovo2812_f0  denovo2890_f0  denovo3306_f0  denovo3332_f0  denovo3436_f0  denovo3592_f0  denovo3618_f0  denovo3644_f0  denovo3722_f0  denovo3748_f0  denovo3800_f0  denovo3826_f0  denovo3878_f0  denovo4060_f0  denovo4086_f0  denovo4138_f0  denovo4164_f0  denovo4242_f0  denovo4320_f0  denovo4346_f0  denovo4372_f0  denovo4424_f0  denovo4502_f0  denovo4528_f0  denovo4632_f0  denovo4840_f0  denovo4996_f0  denovo5022_f0  denovo5100_f0  denovo5126_f0  denovo5152_f0  denovo5334_f0  denovo5412_f0  denovo5490_f0  denovo5542_f0  denovo5568_f0  denovo5724_f0  denovo5776_f0  denovo5854_f0  denovo5880_f0  denovo5906_f0  denovo5958_f0  denovo6036_f0  denovo6088_f0  denovo6192_f0  denovo6270_f0  denovo6296_f0  denovo6322_f0  denovo6348_f0  denovo6374_f0  denovo6478_f0  denovo6504_f0  denovo6530_f0  denovo6556_f0  denovo6634_f0  denovo6660_f0  denovo6712_f0  denovo6738_f0  denovo6894_f0  denovo6972_f0  denovo7024_f0  denovo7050_f0  denovo7128_f0  denovo7154_f0  denovo7284_f0  denovo7310_f0  denovo7336_f0  denovo7362_f0  denovo7388_f0  denovo7440_f0  denovo7466_f0  denovo7492_f0  denovo7544_f0  denovo7622_f0  denovo7648_f0  denovo7700_f0  denovo7778_f0  denovo7830_f0  denovo7856_f0  denovo7908_f0  denovo7934_f0  denovo8012_f0  denovo8038_f0  denovo8090_f0  denovo8142_f0  denovo8194_f0  denovo8350_f0  denovo8376_f0  denovo8402_f0  denovo8480_f0  denovo8532_f0  denovo8558_f0  denovo8610_f0  denovo8636_f0  denovo8688_f0  denovo8714_f0  denovo8740_f0  denovo8766_f0  denovo9052_f0  denovo9078_f0  denovo9104_f0  denovo9130_f0  denovo9208_f0  denovo9234_f0  denovo9286_f0  denovo9338_f0  denovo9468_f0  denovo9494_f0  denovo9624_f0  denovo9650_f0  denovo9780_f0  denovo9832_f0  denovo9884_f0  denovo9962_f0  denovo10014_f0 denovo10040_f0 denovo10118_f0 denovo10144_f0 denovo10274_f0 denovo10300_f0 denovo10430_f0 denovo10456_f0 denovo10508_f0 denovo10534_f0 denovo10560_f0 denovo10586_f0 denovo10716_f0 denovo10742_f0 denovo10768_f0 denovo10820_f0 denovo10898_f0 denovo11002_f0 denovo11054_f0 denovo11132_f0 denovo11210_f0 denovo11288_f0 denovo11392_f0 denovo11418_f0 denovo11470_f0 denovo11496_f0 denovo11522_f0 denovo11574_f0 denovo11600_f0 denovo11704_f0 denovo11730_f0 denovo11756_f0 denovo11860_f0 denovo11886_f0 denovo11938_f0 
FG120022                      denovo3_f0     denovo55_f0    denovo81_f0    denovo159_f0   denovo185_f0   denovo263_f0   denovo315_f0   denovo367_f0   denovo445_f0   denovo549_f0   denovo679_f0   denovo835_f0   denovo861_f0   denovo887_f0   denovo913_f0   denovo1043_f0  denovo1173_f0  denovo1225_f0  denovo1251_f0  denovo1277_f0  denovo1355_f0  denovo1381_f0  denovo1407_f0  denovo1485_f0  denovo1563_f0  denovo1641_f0  denovo1667_f0  denovo1719_f0  denovo1849_f0  denovo1901_f0  denovo1927_f0  denovo1953_f0  denovo1979_f0  denovo2005_f0  denovo2057_f0  denovo2083_f0  denovo2109_f0  denovo2135_f0  denovo2239_f0  denovo2265_f0  denovo2291_f0  denovo2317_f0  denovo2369_f0  denovo2395_f0  denovo2421_f0  denovo2551_f0  denovo2577_f0  denovo2603_f0  denovo2629_f0  denovo2733_f0  denovo2759_f0  denovo2811_f0  denovo2889_f0  denovo3305_f0  denovo3331_f0  denovo3435_f0  denovo3591_f0  denovo3617_f0  denovo3643_f0  denovo3721_f0  denovo3747_f0  denovo3799_f0  denovo3825_f0  denovo3877_f0  denovo4059_f0  denovo4085_f0  denovo4137_f0  denovo4163_f0  denovo4241_f0  denovo4319_f0  denovo4345_f0  denovo4371_f0  denovo4423_f0  denovo4501_f0  denovo4527_f0  denovo4631_f0  denovo4839_f0  denovo4995_f0  denovo5021_f0  denovo5099_f0  denovo5125_f0  denovo5151_f0  denovo5333_f0  denovo5411_f0  denovo5489_f0  denovo5541_f0  denovo5567_f0  denovo5723_f0  denovo5775_f0  denovo5853_f0  denovo5879_f0  denovo5905_f0  denovo5957_f0  denovo6035_f0  denovo6087_f0  denovo6191_f0  denovo6269_f0  denovo6295_f0  denovo6321_f0  denovo6347_f0  denovo6373_f0  denovo6477_f0  denovo6503_f0  denovo6529_f0  denovo6555_f0  denovo6633_f0  denovo6659_f0  denovo6711_f0  denovo6737_f0  denovo6893_f0  denovo6971_f0  denovo7023_f0  denovo7049_f0  denovo7127_f0  denovo7153_f0  denovo7283_f0  denovo7309_f0  denovo7335_f0  denovo7361_f0  denovo7387_f0  denovo7439_f0  denovo7465_f0  denovo7491_f0  denovo7543_f0  denovo7621_f0  denovo7647_f0  denovo7699_f0  denovo7777_f0  denovo7829_f0  denovo7855_f0  denovo7907_f0  denovo7933_f0  denovo8011_f0  denovo8037_f0  denovo8089_f0  denovo8141_f0  denovo8193_f0  denovo8349_f0  denovo8375_f0  denovo8401_f0  denovo8479_f0  denovo8531_f0  denovo8557_f0  denovo8609_f0  denovo8635_f0  denovo8687_f0  denovo8713_f0  denovo8739_f0  denovo8765_f0  denovo9051_f0  denovo9077_f0  denovo9103_f0  denovo9129_f0  denovo9207_f0  denovo9233_f0  denovo9285_f0  denovo9337_f0  denovo9467_f0  denovo9493_f0  denovo9623_f0  denovo9649_f0  denovo9779_f0  denovo9831_f0  denovo9883_f0  denovo9961_f0  denovo10013_f0 denovo10039_f0 denovo10117_f0 denovo10143_f0 denovo10273_f0 denovo10299_f0 denovo10429_f0 denovo10455_f0 denovo10507_f0 denovo10533_f0 denovo10559_f0 denovo10585_f0 denovo10715_f0 denovo10741_f0 denovo10767_f0 denovo10819_f0 denovo10897_f0 denovo11001_f0 denovo11053_f0 denovo11131_f0 denovo11209_f0 denovo11287_f0 denovo11391_f0 denovo11417_f0 denovo11469_f0 denovo11495_f0 denovo11521_f0 denovo11573_f0 denovo11599_f0 denovo11703_f0 denovo11729_f0 denovo11755_f0 denovo11859_f0 denovo11885_f0 denovo11937_f0 
GNV139000                     denovo16_f0    denovo68_f0    denovo94_f0    denovo172_f0   denovo198_f0   denovo276_f0   denovo328_f0   denovo380_f0   denovo458_f0   denovo562_f0   denovo692_f0   denovo848_f0   denovo874_f0   denovo900_f0   denovo926_f0   denovo1056_f0  denovo1186_f0  denovo1238_f0  denovo1264_f0  denovo1290_f0  denovo1368_f0  denovo1394_f0  denovo1420_f0  denovo1498_f0  denovo1576_f0  denovo1654_f0  denovo1680_f0  denovo1732_f0  denovo1862_f0  denovo1914_f0  denovo1940_f0  denovo1966_f0  denovo1992_f0  denovo2018_f0  denovo2070_f0  denovo2096_f0  denovo2122_f0  denovo2148_f0  denovo2252_f0  denovo2278_f0  denovo2304_f0  denovo2330_f0  denovo2382_f0  denovo2408_f0  denovo2434_f0  denovo2564_f0  denovo2590_f0  denovo2616_f0  denovo2642_f0  denovo2746_f0  denovo2772_f0  denovo2824_f0  denovo2902_f0  denovo3318_f0  denovo3344_f0  denovo3448_f0  denovo3604_f0  denovo3630_f0  denovo3656_f0  denovo3734_f0  denovo3760_f0  denovo3812_f0  denovo3838_f0  denovo3890_f0  denovo4072_f0  denovo4098_f0  denovo4150_f0  denovo4176_f0  denovo4254_f0  denovo4332_f0  denovo4358_f0  denovo4384_f0  denovo4436_f0  denovo4514_f0  denovo4540_f0  denovo4644_f0  denovo4852_f0  denovo5008_f0  denovo5034_f0  denovo5112_f0  denovo5138_f0  denovo5164_f0  denovo5346_f0  denovo5424_f0  denovo5502_f0  denovo5554_f0  denovo5580_f0  denovo5736_f0  denovo5788_f0  denovo5866_f0  denovo5892_f0  denovo5918_f0  denovo5970_f0  denovo6048_f0  denovo6100_f0  denovo6204_f0  denovo6282_f0  denovo6308_f0  denovo6334_f0  denovo6360_f0  denovo6386_f0  denovo6490_f0  denovo6516_f0  denovo6542_f0  denovo6568_f0  denovo6646_f0  denovo6672_f0  denovo6724_f0  denovo6750_f0  denovo6906_f0  denovo6984_f0  denovo7036_f0  denovo7062_f0  denovo7140_f0  denovo7166_f0  denovo7296_f0  denovo7322_f0  denovo7348_f0  denovo7374_f0  denovo7400_f0  denovo7452_f0  denovo7478_f0  denovo7504_f0  denovo7556_f0  denovo7634_f0  denovo7660_f0  denovo7712_f0  denovo7790_f0  denovo7842_f0  denovo7868_f0  denovo7920_f0  denovo7946_f0  denovo8024_f0  denovo8050_f0  denovo8102_f0  denovo8154_f0  denovo8206_f0  denovo8362_f0  denovo8388_f0  denovo8414_f0  denovo8492_f0  denovo8544_f0  denovo8570_f0  denovo8622_f0  denovo8648_f0  denovo8700_f0  denovo8726_f0  denovo8752_f0  denovo8778_f0  denovo9064_f0  denovo9090_f0  denovo9116_f0  denovo9142_f0  denovo9220_f0  denovo9246_f0  denovo9298_f0  denovo9350_f0  denovo9480_f0  denovo9506_f0  denovo9636_f0  denovo9662_f0  denovo9792_f0  denovo9844_f0  denovo9896_f0  denovo9974_f0  denovo10026_f0 denovo10052_f0 denovo10130_f0 denovo10156_f0 denovo10286_f0 denovo10312_f0 denovo10442_f0 denovo10468_f0 denovo10520_f0 denovo10546_f0 denovo10572_f0 denovo10598_f0 denovo10728_f0 denovo10754_f0 denovo10780_f0 denovo10832_f0 denovo10910_f0 denovo11014_f0 denovo11066_f0 denovo11144_f0 denovo11222_f0 denovo11300_f0 denovo11404_f0 denovo11430_f0 denovo11482_f0 denovo11508_f0 denovo11534_f0 denovo11586_f0 denovo11612_f0 denovo11716_f0 denovo11742_f0 denovo11768_f0 denovo11872_f0 denovo11898_f0 denovo11950_f0 
Msexta                        denovo17_f0    denovo69_f0    denovo95_f0    denovo173_f0   denovo199_f0   denovo277_f0   denovo329_f0   denovo381_f0   denovo459_f0   denovo563_f0   denovo693_f0   denovo849_f0   denovo875_f0   denovo901_f0   denovo927_f0   denovo1057_f0  denovo1187_f0  denovo1239_f0  denovo1265_f0  denovo1291_f0  denovo1369_f0  denovo1395_f0  denovo1421_f0  denovo1499_f0  denovo1577_f0  denovo1655_f0  denovo1681_f0  denovo1733_f0  denovo1863_f0  denovo1915_f0  denovo1941_f0  denovo1967_f0  denovo1993_f0  denovo2019_f0  denovo2071_f0  denovo2097_f0  denovo2123_f0  denovo2149_f0  denovo2253_f0  denovo2279_f0  denovo2305_f0  denovo2331_f0  denovo2383_f0  denovo2409_f0  denovo2435_f0  denovo2565_f0  denovo2591_f0  denovo2617_f0  denovo2643_f0  denovo2747_f0  denovo2773_f0  denovo2825_f0  denovo2903_f0  denovo3319_f0  denovo3345_f0  denovo3449_f0  denovo3605_f0  denovo3631_f0  denovo3657_f0  denovo3735_f0  denovo3761_f0  denovo3813_f0  denovo3839_f0  denovo3891_f0  denovo4073_f0  denovo4099_f0  denovo4151_f0  denovo4177_f0  denovo4255_f0  denovo4333_f0  denovo4359_f0  denovo4385_f0  denovo4437_f0  denovo4515_f0  denovo4541_f0  denovo4645_f0  denovo4853_f0  denovo5009_f0  denovo5035_f0  denovo5113_f0  denovo5139_f0  denovo5165_f0  denovo5347_f0  denovo5425_f0  denovo5503_f0  denovo5555_f0  denovo5581_f0  denovo5737_f0  denovo5789_f0  denovo5867_f0  denovo5893_f0  denovo5919_f0  denovo5971_f0  denovo6049_f0  denovo6101_f0  denovo6205_f0  denovo6283_f0  denovo6309_f0  denovo6335_f0  denovo6361_f0  denovo6387_f0  denovo6491_f0  denovo6517_f0  denovo6543_f0  denovo6569_f0  denovo6647_f0  denovo6673_f0  denovo6725_f0  denovo6751_f0  denovo6907_f0  denovo6985_f0  denovo7037_f0  denovo7063_f0  denovo7141_f0  denovo7167_f0  denovo7297_f0  denovo7323_f0  denovo7349_f0  denovo7375_f0  denovo7401_f0  denovo7453_f0  denovo7479_f0  denovo7505_f0  denovo7557_f0  denovo7635_f0  denovo7661_f0  denovo7713_f0  denovo7791_f0  denovo7843_f0  denovo7869_f0  denovo7921_f0  denovo7947_f0  denovo8025_f0  denovo8051_f0  denovo8103_f0  denovo8155_f0  denovo8207_f0  denovo8363_f0  denovo8389_f0  denovo8415_f0  denovo8493_f0  denovo8545_f0  denovo8571_f0  denovo8623_f0  denovo8649_f0  denovo8701_f0  denovo8727_f0  denovo8753_f0  denovo8779_f0  denovo9065_f0  denovo9091_f0  denovo9117_f0  denovo9143_f0  denovo9221_f0  denovo9247_f0  denovo9299_f0  denovo9351_f0  denovo9481_f0  denovo9507_f0  denovo9637_f0  denovo9663_f0  denovo9793_f0  denovo9845_f0  denovo9897_f0  denovo9975_f0  denovo10027_f0 denovo10053_f0 denovo10131_f0 denovo10157_f0 denovo10287_f0 denovo10313_f0 denovo10443_f0 denovo10469_f0 denovo10521_f0 denovo10547_f0 denovo10573_f0 denovo10599_f0 denovo10729_f0 denovo10755_f0 denovo10781_f0 denovo10833_f0 denovo10911_f0 denovo11015_f0 denovo11067_f0 denovo11145_f0 denovo11223_f0 denovo11301_f0 denovo11405_f0 denovo11431_f0 denovo11483_f0 denovo11509_f0 denovo11535_f0 denovo11587_f0 denovo11613_f0 denovo11717_f0 denovo11743_f0 denovo11769_f0 denovo11873_f0 denovo11899_f0 denovo11951_f0 
acti2                         denovo25_f0    denovo77_f0    denovo103_f0   denovo181_f0   denovo207_f0   denovo285_f0   denovo337_f0   denovo389_f0   denovo467_f0   denovo571_f0   denovo701_f0   denovo857_f0   denovo883_f0   denovo909_f0   denovo935_f0   denovo1065_f0  denovo1195_f0  denovo1247_f0  denovo1273_f0  denovo1299_f0  denovo1377_f0  denovo1403_f0  denovo1429_f0  denovo1507_f0  denovo1585_f0  denovo1663_f0  denovo1689_f0  denovo1741_f0  denovo1871_f0  denovo1923_f0  denovo1949_f0  denovo1975_f0  denovo2001_f0  denovo2027_f0  denovo2079_f0  denovo2105_f0  denovo2131_f0  denovo2157_f0  denovo2261_f0  denovo2287_f0  denovo2313_f0  denovo2339_f0  denovo2391_f0  denovo2417_f0  denovo2443_f0  denovo2573_f0  denovo2599_f0  denovo2625_f0  denovo2651_f0  denovo2755_f0  denovo2781_f0  denovo2833_f0  denovo2911_f0  denovo3327_f0  denovo3353_f0  denovo3457_f0  denovo3613_f0  denovo3639_f0  denovo3665_f0  denovo3743_f0  denovo3769_f0  denovo3821_f0  denovo3847_f0  denovo3899_f0  denovo4081_f0  denovo4107_f0  denovo4159_f0  denovo4185_f0  denovo4263_f0  denovo4341_f0  denovo4367_f0  denovo4393_f0  denovo4445_f0  denovo4523_f0  denovo4549_f0  denovo4653_f0  denovo4861_f0  denovo5017_f0  denovo5043_f0  denovo5121_f0  denovo5147_f0  denovo5173_f0  denovo5355_f0  denovo5433_f0  denovo5511_f0  denovo5563_f0  denovo5589_f0  denovo5745_f0  denovo5797_f0  denovo5875_f0  denovo5901_f0  denovo5927_f0  denovo5979_f0  denovo6057_f0  denovo6109_f0  denovo6213_f0  denovo6291_f0  denovo6317_f0  denovo6343_f0  denovo6369_f0  denovo6395_f0  denovo6499_f0  denovo6525_f0  denovo6551_f0  denovo6577_f0  denovo6655_f0  denovo6681_f0  denovo6733_f0  denovo6759_f0  denovo6915_f0  denovo6993_f0  denovo7045_f0  denovo7071_f0  denovo7149_f0  denovo7175_f0  denovo7305_f0  denovo7331_f0  denovo7357_f0  denovo7383_f0  denovo7409_f0  denovo7461_f0  denovo7487_f0  denovo7513_f0  denovo7565_f0  denovo7643_f0  denovo7669_f0  denovo7721_f0  denovo7799_f0  denovo7851_f0  denovo7877_f0  denovo7929_f0  denovo7955_f0  denovo8033_f0  denovo8059_f0  denovo8111_f0  denovo8163_f0  denovo8215_f0  denovo8371_f0  denovo8397_f0  denovo8423_f0  denovo8501_f0  denovo8553_f0  denovo8579_f0  denovo8631_f0  denovo8657_f0  denovo8709_f0  denovo8735_f0  denovo8761_f0  denovo8787_f0  denovo9073_f0  denovo9099_f0  denovo9125_f0  denovo9151_f0  denovo9229_f0  denovo9255_f0  denovo9307_f0  denovo9359_f0  denovo9489_f0  denovo9515_f0  denovo9645_f0  denovo9671_f0  denovo9801_f0  denovo9853_f0  denovo9905_f0  denovo9983_f0  denovo10035_f0 denovo10061_f0 denovo10139_f0 denovo10165_f0 denovo10295_f0 denovo10321_f0 denovo10451_f0 denovo10477_f0 denovo10529_f0 denovo10555_f0 denovo10581_f0 denovo10607_f0 denovo10737_f0 denovo10763_f0 denovo10789_f0 denovo10841_f0 denovo10919_f0 denovo11023_f0 denovo11075_f0 denovo11153_f0 denovo11231_f0 denovo11309_f0 denovo11413_f0 denovo11439_f0 denovo11491_f0 denovo11517_f0 denovo11543_f0 denovo11595_f0 denovo11621_f0 denovo11725_f0 denovo11751_f0 denovo11777_f0 denovo11881_f0 denovo11907_f0 denovo11959_f0 
FG120071B                     denovo9_f0     denovo61_f0    denovo87_f0    denovo165_f0   denovo191_f0   denovo269_f0   denovo321_f0   denovo373_f0   denovo451_f0   denovo555_f0   denovo685_f0   denovo841_f0   denovo867_f0   denovo893_f0   denovo919_f0   denovo1049_f0  denovo1179_f0  denovo1231_f0  denovo1257_f0  denovo1283_f0  denovo1361_f0  denovo1387_f0  denovo1413_f0  denovo1491_f0  denovo1569_f0  denovo1647_f0  denovo1673_f0  denovo1725_f0  denovo1855_f0  denovo1907_f0  denovo1933_f0  denovo1959_f0  denovo1985_f0  denovo2011_f0  denovo2063_f0  denovo2089_f0  denovo2115_f0  denovo2141_f0  denovo2245_f0  denovo2271_f0  denovo2297_f0  denovo2323_f0  denovo2375_f0  denovo2401_f0  denovo2427_f0  denovo2557_f0  denovo2583_f0  denovo2609_f0  denovo2635_f0  denovo2739_f0  denovo2765_f0  denovo2817_f0  denovo2895_f0  denovo3311_f0  denovo3337_f0  denovo3441_f0  denovo3597_f0  denovo3623_f0  denovo3649_f0  denovo3727_f0  denovo3753_f0  denovo3805_f0  denovo3831_f0  denovo3883_f0  denovo4065_f0  denovo4091_f0  denovo4143_f0  denovo4169_f0  denovo4247_f0  denovo4325_f0  denovo4351_f0  denovo4377_f0  denovo4429_f0  denovo4507_f0  denovo4533_f0  denovo4637_f0  denovo4845_f0  denovo5001_f0  denovo5027_f0  denovo5105_f0  denovo5131_f0  denovo5157_f0  denovo5339_f0  denovo5417_f0  denovo5495_f0  denovo5547_f0  denovo5573_f0  denovo5729_f0  denovo5781_f0  denovo5859_f0  denovo5885_f0  denovo5911_f0  denovo5963_f0  denovo6041_f0  denovo6093_f0  denovo6197_f0  denovo6275_f0  denovo6301_f0  denovo6327_f0  denovo6353_f0  denovo6379_f0  denovo6483_f0  denovo6509_f0  denovo6535_f0  denovo6561_f0  denovo6639_f0  denovo6665_f0  denovo6717_f0  denovo6743_f0  denovo6899_f0  denovo6977_f0  denovo7029_f0  denovo7055_f0  denovo7133_f0  denovo7159_f0  denovo7289_f0  denovo7315_f0  denovo7341_f0  denovo7367_f0  denovo7393_f0  denovo7445_f0  denovo7471_f0  denovo7497_f0  denovo7549_f0  denovo7627_f0  denovo7653_f0  denovo7705_f0  denovo7783_f0  denovo7835_f0  denovo7861_f0  denovo7913_f0  denovo7939_f0  denovo8017_f0  denovo8043_f0  denovo8095_f0  denovo8147_f0  denovo8199_f0  denovo8355_f0  denovo8381_f0  denovo8407_f0  denovo8485_f0  denovo8537_f0  denovo8563_f0  denovo8615_f0  denovo8641_f0  denovo8693_f0  denovo8719_f0  denovo8745_f0  denovo8771_f0  denovo9057_f0  denovo9083_f0  denovo9109_f0  denovo9135_f0  denovo9213_f0  denovo9239_f0  denovo9291_f0  denovo9343_f0  denovo9473_f0  denovo9499_f0  denovo9629_f0  denovo9655_f0  denovo9785_f0  denovo9837_f0  denovo9889_f0  denovo9967_f0  denovo10019_f0 denovo10045_f0 denovo10123_f0 denovo10149_f0 denovo10279_f0 denovo10305_f0 denovo10435_f0 denovo10461_f0 denovo10513_f0 denovo10539_f0 denovo10565_f0 denovo10591_f0 denovo10721_f0 denovo10747_f0 denovo10773_f0 denovo10825_f0 denovo10903_f0 denovo11007_f0 denovo11059_f0 denovo11137_f0 denovo11215_f0 denovo11293_f0 denovo11397_f0 denovo11423_f0 denovo11475_f0 denovo11501_f0 denovo11527_f0 denovo11579_f0 denovo11605_f0 denovo11709_f0 denovo11735_f0 denovo11761_f0 denovo11865_f0 denovo11891_f0 denovo11943_f0
[truncated: 196,331 more chars]
