# Supplementary material for: ReproPhylo: An Environment for Reproducible Phylogenomics
Source: PLoS Comput Biol. 2015 Sep 3;11(9):e1004447. doi: 10.1371/journal.pcbi.1004447 (PMC4559436; doi:10.1371/journal.pcbi.1004447)
Supplement: S1 Results — A results archive produced by ReproPhylo, containing the serialized Project, input and output files, scripts and an HTML report. http://dx.doi.org/10.6084/m9.figshare.1409488 (ZIP) [file pcbi.1004447.s004.zip › report/report.html]

# reprophylo analysis from Wed Apr 22 21:27:34 2015


  

## Data

  

### Species Representation In Sequence Data

  
  

| species | EOG69CQC1\_1 | EOG6M65NR\_1 | EOG69KFV8\_1 | EOG6SN1SH\_1 | EOG654933\_1 | EOG66147B\_1 | EOG6PRSVJ\_1 | EOG6PRSVH\_1 | EOG680J15\_1 | EOG6S7JTK\_1 | EOG60ZR1Z\_1 | EOG6DBTJG\_1 | EOG6FXRCN\_1 | EOG69ZXTF\_1 | EOG60P4BD\_1 | EOG6BCDR6\_1 | EOG64QT4T\_1 | EOG6NCMGZ\_1 | EOG66147D\_1 | EOG67SSJD\_1 | EOG6K3M19\_1 | EOG670TN2\_1 | EOG60P4BJ\_1 | EOG6DBTK0\_1 | EOG647FZH\_1 | EOG6D5327\_1 | EOG63TZZV\_1 | EOG6HDSZD\_1 | EOG64MXX7\_1 | EOG68WC5N\_1 | EOG6GTKHJ\_1 | EOG68SGXD\_1 | EOG6G7C30\_1 | EOG6K6G7Z\_1 | EOG6SXNH7\_1 | EOG65X80D\_1 | EOG6NVZQC\_1 | EOG6MPHV2\_1 | EOG6SJ5J4\_1 | EOG6C87X0\_1 | EOG68KRFS\_1 | EOG6GHZSR\_1 | EOG65493R\_1 | EOG6868HF\_1 | EOG666VQ4\_1 | EOG6MSD2X\_1 | EOG63FH1T\_1 | EOG698V44\_1 | EOG6F7NPM\_1 | EOG676K3N\_1 | EOG651DV5\_1 | EOG6KWJXS\_1 | EOG6894QQ\_1 | EOG6868GW\_1 | EOG69PB31\_1 | EOG6SJ5JV\_1 | EOG65492X\_1 | EOG6JDHBD\_1 | EOG63212K\_1 | EOG67H5VB\_1 | EOG6C87X4\_1 | EOG61C717\_1 | EOG673PVJ\_1 | EOG68D102\_1 | EOG60ZR1T\_1 | EOG68WC5K\_1 | EOG63JC89\_1 | EOG6CJVMH\_1 | EOG60VVTB\_1 | EOG698V46\_1 | EOG6GHZSZ\_1 | EOG6J6RV9\_1 | EOG6S4PM1\_1 | EOG68D104\_1 | EOG68KRG8\_1 | EOG6N8R81\_1 | EOG63BMSV\_1 | EOG69KFV1\_1 | EOG6H1B0Q\_1 | EOG6CJVMF\_1 | EOG6C2HF4\_1 | EOG6CC44S\_1 | EOG6FXRCK\_1 | EOG6M65N3\_1 | EOG6R2400\_1 | EOG6BK57N\_1 | EOG68KRG4\_1 | EOG61C70T\_1 | EOG6BK575\_1 | EOG6R506X\_1 | EOG6SJ5JS\_1 | EOG6JT09G\_1 | EOG6HQDPG\_1 | EOG6H4677\_1 | EOG63212W\_1 | EOG6F7NPJ\_1 | EOG695ZW4\_1 | EOG61NTQM\_1 | EOG6SJ5J3\_1 | EOG6NZTZ3\_1 | EOG6J3WM7\_1 | EOG680J16\_1 | EOG68SGXM\_1 | EOG60K843\_1 | EOG65X811\_1 | EOG602WWN\_1 | EOG6RNBCT\_1 | EOG6M39DN\_1 | EOG63BMSB\_1 | EOG6RFKXS\_1 | EOG6RXZ4G\_1 | EOG6B8JHB\_1 | EOG67SSJJ\_1 | EOG654930\_1 | EOG6PVP2X\_1 | EOG69GKKW\_1 | EOG6RXZ3G\_1 | EOG6NZTXP\_1 | EOG602WX4\_1 | EOG6KH2ZX\_1 | EOG61JZGH\_1 | EOG6QFWHZ\_1 | EOG6SJ5J9\_1 | EOG6STS8H\_1 | EOG68PMPX\_1 | EOG6RFKXB\_1 | EOG62BXD6\_1 | EOG647FXX\_1 | EOG6B8JHP\_1 | EOG6F7NPN\_1 | EOG6GHZSG\_1 | EOG6P8FN9\_1 | EOG6S1TBX\_1 | EOG6R506R\_1 | EOG6DFPRT\_1 | EOG65TCRW\_1 | EOG6HX54P\_1 | EOG60VVST\_1 | EOG65HS25\_1 | EOG6FFD5C\_1 | EOG6PNXM9\_1 | EOG6R506D\_1 | EOG670TNG\_1 | EOG6F4SFZ\_1 | EOG6Q8524\_1 | EOG6QVCGP\_1 | EOG669QZR\_1 | EOG6JT09B\_1 | EOG65TCRS\_1 | EOG6CJVMM\_1 | EOG6K9BH5\_1 | EOG63N7HX\_1 | EOG6QRH88\_1 | EOG6M65N6\_1 | EOG6JQ426\_1 | EOG61G385\_1 | EOG6QRH81\_1 | EOG6NCMH9\_1 | EOG6FN4N0\_1 | EOG65HS1N\_1 | EOG6CC44T\_1 | EOG6RJG4W\_1 | EOG6K9BGH\_1 | EOG6FR0VW\_1 | EOG6N8R82\_1 | EOG6K0QSG\_1 | EOG6KKZ6X\_1 | EOG60VVTG\_1 | EOG6QC198\_1 | EOG6D26TG\_1 | EOG6FFD52\_1 | EOG6QZ7R6\_1 | EOG6KKZ71\_1 | EOG634W9K\_1 | EOG6DNF7N\_1 | EOG679FBS\_1 | EOG6BG909\_1 | EOG63BMSG\_1 | EOG61RPZF\_1 | EOG6BVRZ3\_1 | EOG6MSD3H\_1 | EOG60CHM6\_1 | EOG6PC9WG\_1 | EOG60GCVD\_1 | EOG6R506Q\_1 | EOG66MBNX\_1 | EOG605S4X\_1 | EOG66HGF2\_1 | EOG65TCRN\_1 | EOG608ND4\_1 | EOG6640GX\_1 | EOG6SQX12\_1 | EOG69GKMS\_1 | EOG6FR0VX\_1 | EOG6P2Q5K\_1 | EOG634W9M\_1 | EOG6B8JHC\_1 | EOG6QVCGS\_1 | EOG6Q2DJZ\_1 | EOG64TPC4\_1 | EOG6D26TV\_1 | EOG605S4Z\_1 | EOG6N04JN\_1 | EOG6DNF8K\_1 | EOG68GW71\_1 | EOG6CC44M\_1 | EOG63JC8S\_1 | EOG67WNS9\_1 | EOG6NS3G8\_1 | EOG64TPCQ\_1 | EOG6PNXM6\_1 | EOG68GW77\_1 | EOG6NS3GS\_1 | EOG6229Q2\_1 | EOG63TZZW\_1 | EOG6NP76T\_1 | EOG63JC90\_1 | EOG67H5V2\_1 | EOG63R3QZ\_1 | EOG65QHJ5\_1 | EOG6MW89D\_1 | EOG64F6DH\_1 | EOG6SJ5K0\_1 | EOG69GKM6\_1 | EOG66HGFW\_1 | EOG6MKNM3\_1 | EOG63V00H\_1 | EOG6BG90S\_1 | EOG6NP77P\_1 | EOG66DM62\_1 | EOG6JQ41Z\_1 | EOG6QFWJH\_1 | EOG69CQBW\_1 | EOG6N04K4\_1 | EOG69KFTV\_1 | EOG6CJVMQ\_1 | EOG6SBF24\_1 | EOG6P8FNB\_1 | EOG62V8KS\_1 | EOG6DZ204\_1 | EOG6C5CPH\_1 | EOG6FFD51\_1 | EOG6QNN0J\_1 | EOG6PC9WN\_1 | EOG6Q8529\_1 | EOG6KSPP5\_1 | EOG6G1MMG\_1 | EOG63XV6Q\_1 | EOG6D531X\_1 | EOG61ZFF6\_1 | EOG6N5W22\_1 | EOG63V009\_1 | EOG6PK2CG\_1 | EOG6NP779\_1 | EOG6933N9\_1 | EOG68GW6V\_1 | EOG66Q6XP\_1 | EOG6JQ427\_1 | EOG6C5CNP\_1 | EOG6R5066\_1 | EOG6FTW4C\_1 | EOG6GXFRF\_1 | EOG65759X\_1 | EOG6KWJX8\_1 | EOG6QNN0N\_1 | EOG641QFN\_1 | EOG6S1TBT\_1 | EOG6GB79M\_1 | EOG65B1K2\_1 | EOG66WZD1\_1 | EOG65TCRK\_1 | EOG6PNXN7\_1 | EOG6FR0WR\_1 | EOG6QRH78\_1 | EOG6P2Q64\_1 | EOG666VQC\_1 | EOG6FJ8F2\_1 | EOG6HHP6H\_1 | EOG6DJK1Q\_1 | EOG63R3R5\_1 | EOG6PC9WF\_1 | EOG6Q58T0\_1 | EOG641QGH\_1 | EOG6933N7\_1 | EOG69S6BG\_1 | EOG64XJM7\_1 | EOG66Q6XR\_1 | EOG6PK2CM\_1 | EOG6NS3FV\_1 | EOG65MN9B\_1 | EOG641QFW\_1 | EOG63R3R9\_1 | EOG6DZ1ZZ\_1 | EOG6N04J6\_1 | EOG6CRM3G\_1 | EOG6GMV1P\_1 | EOG6S4PKX\_1 | EOG65MN9F\_1 | EOG6NP773\_1 | EOG6K6G7S\_1 | EOG6DZ201\_1 | EOG6BZN62\_1 | EOG68SGZ1\_1 | EOG69PB2Z\_1 | EOG6SXNGX\_1 | EOG6QZ7Q8\_1 | EOG6DR9GP\_1 | EOG6QFWJ6\_1 | EOG6QC18P\_1 | EOG6CG0CT\_1 | EOG6HHP6B\_1 | EOG6PK2CQ\_1 | EOG67D9KJ\_1 | EOG6JWVJQ\_1 | EOG6F1X6H\_1 | EOG6PG64S\_1 | EOG605S4Q\_1 | EOG6R7VF9\_1 | EOG6CZBM5\_1 | EOG6JT092\_1 | EOG6B5P96\_1 | EOG63R3RK\_1 | EOG64F6DK\_1 | EOG6FJ8D6\_1 | EOG6QZ7QX\_1 | EOG6F1X6M\_1 | EOG6001NN\_1 | EOG62V8KH\_1 | EOG695ZWC\_1 | EOG67PX9B\_1 | EOG6SBF2V\_1 | EOG6P8FNH\_1 | EOG6KSPQ0\_1 | EOG6GMV1H\_1 | EOG63R3RC\_1 | EOG6907D7\_1 | EOG6JWVHT\_1 | EOG68SGX8\_1 | EOG6HDSZ9\_1 | EOG6BK57S\_1 | EOG60K83N\_1 | EOG6SXNHD\_1 | EOG6KD6Q9\_1 | EOG68GW6Q\_1 | EOG6Q852G\_1 | EOG676K44\_1 | EOG6FN4NZ\_1 | EOG6Q58ST\_1 | EOG6933NR\_1 | EOG62JNVR\_1 | EOG64MXWZ\_1 | EOG6S1TC2\_1 | EOG6HT8WD\_1 | EOG63FH1F\_1 | EOG6PC9X3\_1 | EOG6Q58SW\_1 | EOG63N7HF\_1 | EOG6CRM3H\_1 | EOG6PRSVP\_1 | EOG6R5069\_1 | EOG6CC453\_1 | EOG6001PC\_1 | EOG63BMS7\_1 | EOG676K40\_1 | EOG64MXWK\_1 | EOG6N30SN\_1 | EOG6575BP\_1 | EOG67D9M5\_1 | EOG615GJK\_1 | EOG6RBQP0\_1 | EOG68KRFQ\_1 | EOG6DR9GZ\_1 | EOG6Q2DKD\_1 | EOG6255XM\_1 | EOG64BB5X\_1 | EOG6PG646\_1 | EOG62FSN7\_1 | EOG6H72G0\_1 | EOG6PZJB1\_1 | EOG64MXWG\_1 | EOG65QHHP\_1 | EOG6C87WM\_1 | EOG698V4R\_1 | EOG615GHV\_1 | EOG6FFD5G\_1 | EOG66WZCQ\_1 | EOG698V4H\_1 | EOG6NVZQ8\_1 | EOG6001NK\_1 | EOG6JDHB2\_1 | EOG62BXCX\_1 | EOG60K833\_1 | EOG62BXCZ\_1 | EOG6CG0D0\_1 | EOG6JM7SX\_1 | EOG69KFVF\_1 | EOG615GHP\_1 | EOG6KH2ZR\_1 | EOG683D8Q\_1 | EOG6PRSVM\_1 | EOG64J2NS\_1 | EOG66HGF5\_1 | EOG6DFPSF\_1 | EOG60P4BZ\_1 | EOG66MBPR\_1 | EOG6DFPSD\_1 | EOG67D9KC\_1 | EOG6HHP5S\_1 | EOG64BB5N\_1 | EOG6STS7W\_1 | EOG6DBTJX\_1 | EOG6FXRC9\_1 | EOG6DBTHZ\_1 | EOG6KH2ZD\_1 | EOG6STS87\_1 | EOG65TCS9\_1 | EOG67D9KV\_1 | EOG6SXNHG\_1 | EOG6255XS\_1 | EOG65MN8T\_1 | EOG6GF3JF\_1 | EOG66Q6X8\_1 | EOG6KSPPS\_1 | EOG61RPZ9\_1 | EOG637RJP\_1 | EOG66MBP7\_1 | EOG6J3WMR\_1 | EOG6B2T20\_1 | EOG6QJRRQ\_1 | EOG6GB79H\_1 | EOG6JDHBG\_1 | EOG6Q852B\_1 | EOG676K3Q\_1 | EOG6RV2WT\_1 | EOG6DV5QM\_1 | EOG6GTKHQ\_1 | EOG6FR0W5\_1 | EOG6NVZPZ\_1 | EOG6K0QRT\_1 | EOG65DWSM\_1 | EOG6CC452\_1 | EOG6G4GTX\_1 | EOG64BB5H\_1 | EOG64J2NC\_1 | EOG61G37F\_1 | EOG6H72G3\_1 | EOG6SBF2B\_1 | EOG695ZW1\_1 | EOG68WC58\_1 | EOG6CG0CX\_1 | EOG6FFD5V\_1 | EOG6G4GTT\_1 | EOG6K3M11\_1 | EOG651DTX\_1 | EOG60VVT3\_1 | EOG62NJ41\_1 | EOG66147J\_1 | EOG63FH1C\_1 | EOG6GXFR5\_1 | EOG61C70W\_1 | EOG6DV5QK\_1 | EOG6HQDPF\_1 | EOG6NVZPX\_1 | EOG6GXFR3\_1 | EOG6KPTG0\_1 |
| --- | --- | --- | --- | --- | --- | --- | --- | --- | --- | --- | --- | --- | --- | --- | --- | --- | --- | --- | --- | --- | --- | --- | --- | --- | --- | --- | --- | --- | --- | --- | --- | --- | --- | --- | --- | --- | --- | --- | --- | --- | --- | --- | --- | --- | --- | --- | --- | --- | --- | --- | --- | --- | --- | --- | --- | --- | --- | --- | --- | --- | --- | --- | --- | --- | --- | --- | --- | --- | --- | --- | --- | --- | --- | --- | --- | --- | --- | --- | --- | --- | --- | --- | --- | --- | --- | --- | --- | --- | --- | --- | --- | --- | --- | --- | --- | --- | --- | --- | --- | --- | --- | --- | --- | --- | --- | --- | --- | --- | --- | --- | --- | --- | --- | --- | --- | --- | --- | --- | --- | --- | --- | --- | --- | --- | --- | --- | --- | --- | --- | --- | --- | --- | --- | --- | --- | --- | --- | --- | --- | --- | --- | --- | --- | --- | --- | --- | --- | --- | --- | --- | --- | --- | --- | --- | --- | --- | --- | --- | --- | --- | --- | --- | --- | --- | --- | --- | --- | --- | --- | --- | --- | --- | --- | --- | --- | --- | --- | --- | --- | --- | --- | --- | --- | --- | --- | --- | --- | --- | --- | --- | --- | --- | --- | --- | --- | --- | --- | --- | --- | --- | --- | --- | --- | --- | --- | --- | --- | --- | --- | --- | --- | --- | --- | --- | --- | --- | --- | --- | --- | --- | --- | --- | --- | --- | --- | --- | --- | --- | --- | --- | --- | --- | --- | --- | --- | --- | --- | --- | --- | --- | --- | --- | --- | --- | --- | --- | --- | --- | --- | --- | --- | --- | --- | --- | --- | --- | --- | --- | --- | --- | --- | --- | --- | --- | --- | --- | --- | --- | --- | --- | --- | --- | --- | --- | --- | --- | --- | --- | --- | --- | --- | --- | --- | --- | --- | --- | --- | --- | --- | --- | --- | --- | --- | --- | --- | --- | --- | --- | --- | --- | --- | --- | --- | --- | --- | --- | --- | --- | --- | --- | --- | --- | --- | --- | --- | --- | --- | --- | --- | --- | --- | --- | --- | --- | --- | --- | --- | --- | --- | --- | --- | --- | --- | --- | --- | --- | --- | --- | --- | --- | --- | --- | --- | --- | --- | --- | --- | --- | --- | --- | --- | --- | --- | --- | --- | --- | --- | --- | --- | --- | --- | --- | --- | --- | --- | --- | --- | --- | --- | --- | --- | --- | --- | --- | --- | --- | --- | --- | --- | --- | --- | --- | --- | --- | --- | --- | --- | --- | --- | --- | --- | --- | --- | --- | --- | --- | --- | --- | --- | --- | --- | --- | --- | --- | --- | --- | --- | --- | --- | --- | --- | --- | --- | --- | --- | --- | --- | --- | --- | --- | --- | --- | --- | --- | --- | --- | --- | --- | --- | --- | --- | --- | --- | --- | --- | --- | --- | --- | --- | --- | --- | --- | --- | --- | --- | --- | --- | --- | --- | --- | --- | --- | --- | --- | --- | --- | --- | --- | --- | --- | --- | --- | --- | --- | --- |
| Actias luna\* | 1 | 1 | 1 | 1 | 1 | 1 | 1 | 1 | 1 | 1 | 1 | 1 | 1 | 1 | 1 | 1 | 1 | 1 | 1 | 1 | 1 | 1 | 1 | 1 | 1 | 1 | 1 | 1 | 1 | 1 | 1 | 1 | 1 | 1 | 1 | 1 | 1 | 1 | 1 | 1 | 1 | 1 | 1 | 1 | 1 | 1 | 1 | 1 | 1 | 1 | 1 | 1 | 1 | 1 | 1 | 1 | 1 | 1 | 1 | 1 | 1 | 1 | 1 | 1 | 1 | 1 | 1 | 1 | 1 | 1 | 1 | 1 | 1 | 1 | 1 | 1 | 1 | 1 | 1 | 1 | 1 | 1 | 1 | 1 | 1 | 1 | 1 | 1 | 1 | 1 | 1 | 1 | 1 | 1 | 1 | 1 | 1 | 1 | 1 | 1 | 1 | 1 | 1 | 1 | 1 | 1 | 1 | 1 | 1 | 1 | 1 | 1 | 1 | 1 | 1 | 1 | 1 | 1 | 1 | 1 | 1 | 1 | 1 | 1 | 1 | 1 | 1 | 1 | 1 | 1 | 1 | 1 | 1 | 1 | 1 | 1 | 1 | 1 | 1 | 1 | 1 | 1 | 1 | 1 | 1 | 1 | 1 | 1 | 1 | 1 | 1 | 1 | 1 | 1 | 1 | 1 | 1 | 1 | 1 | 1 | 1 | 1 | 1 | 1 | 1 | 1 | 1 | 1 | 1 | 1 | 1 | 1 | 1 | 1 | 1 | 1 | 1 | 1 | 1 | 1 | 1 | 1 | 1 | 1 | 1 | 1 | 1 | 1 | 1 | 1 | 1 | 1 | 1 | 1 | 1 | 1 | 1 | 1 | 1 | 1 | 1 | 1 | 1 | 1 | 1 | 1 | 1 | 1 | 1 | 1 | 1 | 1 | 1 | 1 | 1 | 1 | 1 | 1 | 1 | 1 | 1 | 1 | 1 | 1 | 1 | 1 | 1 | 1 | 1 | 1 | 1 | 1 | 1 | 1 | 1 | 1 | 1 | 1 | 1 | 1 | 1 | 1 | 1 | 1 | 1 | 1 | 1 | 1 | 1 | 1 | 1 | 1 | 1 | 1 | 1 | 1 | 1 | 1 | 1 | 1 | 1 | 1 | 1 | 1 | 1 | 1 | 1 | 1 | 1 | 1 | 1 | 1 | 1 | 1 | 1 | 1 | 1 | 1 | 1 | 1 | 1 | 1 | 1 | 1 | 1 | 1 | 1 | 1 | 1 | 1 | 1 | 1 | 1 | 1 | 1 | 1 | 1 | 1 | 1 | 1 | 1 | 1 | 1 | 1 | 1 | 1 | 1 | 1 | 1 | 1 | 1 | 1 | 1 | 1 | 1 | 1 | 1 | 1 | 1 | 1 | 1 | 1 | 1 | 1 | 1 | 1 | 1 | 1 | 1 | 1 | 1 | 1 | 1 | 1 | 1 | 1 | 1 | 1 | 1 | 1 | 1 | 1 | 1 | 1 | 1 | 1 | 1 | 1 | 1 | 1 | 1 | 1 | 1 | 1 | 1 | 1 | 1 | 1 | 1 | 1 | 1 | 1 | 1 | 1 | 1 | 1 | 1 | 1 | 1 | 1 | 1 | 1 | 1 | 1 | 1 | 1 | 1 | 1 | 1 | 1 | 1 | 1 | 1 | 1 | 1 | 1 | 1 | 1 | 1 | 1 | 1 | 1 | 1 | 1 | 1 | 1 | 1 | 1 | 1 | 1 | 1 | 1 | 1 | 1 | 1 | 1 | 1 | 1 | 1 | 1 | 1 | 1 | 1 | 1 | 1 | 1 | 1 | 1 | 1 | 1 | 1 | 1 | 1 | 1 | 1 | 1 | 1 | 1 | 1 | 1 | 1 | 1 | 1 | 1 | 1 | 1 | 1 | 1 | 1 | 1 | 1 | 1 | 1 | 1 | 1 | 1 | 1 | 1 | 1 | 1 | 1 | 1 | 1 | 1 | 1 | 1 | 1 | 1 | 1 | 1 | 1 | 1 | 1 | 1 | 1 |
| Anigraea sp. | 1 | 1 | 1 | 1 | 1 | 1 | 1 | 1 | 1 | 1 | 1 | 1 | 1 | 1 | 1 | 1 | 1 | 1 | 1 | 1 | 1 | 1 | 1 | 1 | 1 | 1 | 1 | 1 | 1 | 1 | 1 | 1 | 1 | 1 | 1 | 1 | 1 | 1 | 1 | 1 | 1 | 1 | 1 | 1 | 1 | 1 | 1 | 1 | 1 | 1 | 1 | 1 | 1 | 1 | 1 | 1 | 1 | 1 | 1 | 1 | 1 | 1 | 1 | 1 | 1 | 1 | 1 | 1 | 1 | 1 | 1 | 1 | 1 | 1 | 1 | 1 | 1 | 1 | 1 | 1 | 1 | 1 | 1 | 1 | 1 | 1 | 1 | 1 | 1 | 1 | 1 | 1 | 1 | 1 | 1 | 1 | 1 | 1 | 1 | 1 | 1 | 1 | 1 | 1 | 1 | 1 | 1 | 1 | 1 | 1 | 1 | 1 | 1 | 1 | 1 | 1 | 1 | 1 | 1 | 1 | 1 | 1 | 1 | 1 | 1 | 1 | 1 | 1 | 1 | 1 | 1 | 1 | 1 | 1 | 1 | 1 | 1 | 1 | 1 | 1 | 1 | 1 | 1 | 1 | 1 | 1 | 1 | 1 | 1 | 1 | 1 | 1 | 1 | 1 | 1 | 1 | 1 | 1 | 1 | 1 | 1 | 1 | 1 | 1 | 1 | 1 | 1 | 1 | 1 | 1 | 1 | 1 | 1 | 1 | 1 | 1 | 1 | 1 | 1 | 1 | 1 | 1 | 1 | 1 | 1 | 1 | 1 | 1 | 1 | 1 | 1 | 1 | 1 | 1 | 1 | 1 | 1 | 1 | 1 | 1 | 1 | 1 | 1 | 1 | 1 | 1 | 1 | 1 | 1 | 1 | 1 | 1 | 1 | 1 | 1 | 1 | 1 | 1 | 1 | 1 | 1 | 1 | 1 | 1 | 1 | 1 | 1 | 1 | 1 | 1 | 1 | 1 | 1 | 1 | 1 | 1 | 1 | 1 | 1 | 1 | 1 | 1 | 1 | 1 | 1 | 1 | 1 | 1 | 1 | 1 | 1 | 1 | 1 | 1 | 1 | 1 | 1 | 1 | 1 | 1 | 1 | 1 | 1 | 1 | 1 | 1 | 1 | 1 | 1 | 1 | 1 | 1 | 1 | 1 | 1 | 1 | 1 | 1 | 1 | 1 | 1 | 1 | 1 | 1 | 1 | 1 | 1 | 1 | 1 | 1 | 1 | 1 | 1 | 1 | 1 | 1 | 1 | 1 | 1 | 1 | 1 | 1 | 1 | 1 | 1 | 1 | 1 | 1 | 1 | 1 | 1 | 1 | 1 | 1 | 1 | 1 | 1 | 1 | 1 | 1 | 1 | 1 | 1 | 1 | 1 | 1 | 1 | 1 | 1 | 1 | 1 | 1 | 1 | 1 | 1 | 1 | 1 | 1 | 1 | 1 | 1 | 1 | 1 | 1 | 1 | 1 | 1 | 1 | 1 | 1 | 1 | 1 | 1 | 1 | 1 | 1 | 1 | 1 | 1 | 1 | 1 | 1 | 1 | 1 | 1 | 1 | 1 | 1 | 1 | 1 | 1 | 1 | 1 | 1 | 1 | 1 | 1 | 1 | 1 | 1 | 1 | 1 | 1 | 1 | 1 | 1 | 1 | 1 | 1 | 1 | 1 | 1 | 1 | 1 | 1 | 1 | 1 | 1 | 1 | 1 | 1 | 1 | 1 | 1 | 1 | 1 | 1 | 1 | 1 | 1 | 1 | 1 | 1 | 1 | 1 | 1 | 1 | 1 | 1 | 1 | 1 | 1 | 1 | 1 | 1 | 1 | 1 | 1 | 1 | 1 | 1 | 1 | 1 | 1 | 1 | 1 | 1 | 1 | 1 | 1 | 1 | 1 | 1 | 1 | 1 | 1 | 1 | 1 | 1 | 1 | 1 | 1 | 1 | 1 | 1 | 1 | 1 | 1 | 1 | 1 | 1 | 1 | 1 | 1 | 1 |
| Artace sp.\* | 1 | 1 | 1 | 1 | 1 | 1 | 1 | 1 | 1 | 1 | 1 | 1 | 1 | 1 | 1 | 1 | 1 | 1 | 1 | 1 | 1 | 1 | 1 | 1 | 1 | 1 | 1 | 1 | 1 | 1 | 1 | 1 | 1 | 1 | 1 | 1 | 1 | 1 | 1 | 1 | 1 | 1 | 1 | 1 | 1 | 1 | 1 | 1 | 1 | 1 | 1 | 1 | 1 | 1 | 1 | 1 | 1 | 1 | 1 | 1 | 1 | 1 | 1 | 1 | 1 | 1 | 1 | 1 | 1 | 1 | 1 | 1 | 1 | 1 | 1 | 1 | 1 | 1 | 1 | 1 | 1 | 1 | 1 | 1 | 1 | 1 | 1 | 1 | 1 | 1 | 1 | 1 | 1 | 1 | 1 | 1 | 1 | 1 | 1 | 1 | 1 | 1 | 1 | 1 | 1 | 1 | 1 | 1 | 1 | 1 | 1 | 1 | 1 | 1 | 1 | 1 | 1 | 1 | 1 | 1 | 1 | 1 | 1 | 1 | 1 | 1 | 1 | 1 | 1 | 1 | 1 | 1 | 1 | 1 | 1 | 1 | 1 | 1 | 1 | 1 | 1 | 1 | 1 | 1 | 1 | 1 | 1 | 1 | 1 | 1 | 1 | 1 | 1 | 1 | 1 | 1 | 1 | 1 | 1 | 1 | 1 | 1 | 1 | 1 | 1 | 1 | 1 | 1 | 1 | 1 | 1 | 1 | 1 | 1 | 1 | 1 | 1 | 1 | 1 | 1 | 1 | 1 | 1 | 1 | 1 | 1 | 1 | 1 | 1 | 1 | 1 | 1 | 1 | 1 | 1 | 1 | 1 | 1 | 1 | 1 | 1 | 1 | 1 | 1 | 1 | 1 | 1 | 1 | 1 | 1 | 1 | 1 | 1 | 1 | 1 | 1 | 1 | 1 | 1 | 1 | 1 | 1 | 1 | 1 | 1 | 1 | 1 | 1 | 1 | 1 | 1 | 1 | 1 | 1 | 1 | 1 | 1 | 1 | 1 | 1 | 1 | 1 | 1 | 1 | 1 | 1 | 1 | 1 | 1 | 1 | 1 | 1 | 1 | 1 | 1 | 1 | 1 | 1 | 1 | 1 | 1 | 1 | 1 | 1 | 1 | 1 | 1 | 1 | 1 | 1 | 1 | 1 | 1 | 1 | 1 | 1 | 1 | 1 | 1 | 1 | 1 | 1 | 1 | 1 | 1 | 1 | 1 | 1 | 1 | 1 | 1 | 1 | 1 | 1 | 1 | 1 | 1 | 1 | 1 | 1 | 1 | 1 | 1 | 1 | 1 | 1 | 1 | 1 | 1 | 1 | 1 | 1 | 1 | 1 | 1 | 1 | 1 | 1 | 1 | 1 | 1 | 1 | 1 | 1 | 1 | 1 | 1 | 1 | 1 | 1 | 1 | 1 | 1 | 1 | 1 | 1 | 1 | 1 | 1 | 1 | 1 | 1 | 1 | 1 | 1 | 1 | 1 | 1 | 1 | 1 | 1 | 1 | 1 | 1 | 1 | 1 | 1 | 1 | 1 | 1 | 1 | 1 | 1 | 1 | 1 | 1 | 1 | 1 | 1 | 1 | 1 | 1 | 1 | 1 | 1 | 1 | 1 | 1 | 1 | 1 | 1 | 1 | 1 | 1 | 1 | 1 | 1 | 1 | 1 | 1 | 1 | 1 | 1 | 1 | 1 | 1 | 1 | 1 | 1 | 1 | 1 | 1 | 1 | 1 | 1 | 1 | 1 | 1 | 1 | 1 | 1 | 1 | 1 | 1 | 1 | 1 | 1 | 1 | 1 | 1 | 1 | 1 | 1 | 1 | 1 | 1 | 1 | 1 | 1 | 1 | 1 | 1 | 1 | 1 | 1 | 1 | 1 | 1 | 1 | 1 | 1 | 1 | 1 | 1 | 1 | 1 | 1 | 1 | 1 | 1 | 1 | 1 | 1 | 1 | 1 | 1 | 1 | 1 | 1 | 1 | 1 | 1 | 1 | 1 | 1 |
| Bombyx mori\* | 1 | 1 | 1 | 1 | 1 | 1 | 1 | 1 | 1 | 1 | 1 | 1 | 1 | 1 | 1 | 1 | 1 | 1 | 1 | 1 | 1 | 1 | 1 | 1 | 1 | 1 | 1 | 1 | 1 | 1 | 1 | 1 | 1 | 1 | 1 | 1 | 1 | 1 | 1 | 1 | 1 | 1 | 1 | 1 | 1 | 1 | 1 | 1 | 1 | 1 | 1 | 1 | 1 | 1 | 1 | 1 | 1 | 1 | 1 | 1 | 1 | 1 | 1 | 1 | 1 | 1 | 1 | 1 | 1 | 1 | 1 | 1 | 1 | 1 | 1 | 1 | 1 | 1 | 1 | 1 | 1 | 1 | 1 | 1 | 1 | 1 | 1 | 1 | 1 | 1 | 1 | 1 | 1 | 1 | 1 | 1 | 1 | 1 | 1 | 1 | 1 | 1 | 1 | 1 | 1 | 1 | 1 | 1 | 1 | 1 | 1 | 1 | 1 | 1 | 1 | 1 | 1 | 1 | 1 | 1 | 1 | 1 | 1 | 1 | 1 | 1 | 1 | 1 | 1 | 1 | 1 | 1 | 1 | 1 | 1 | 1 | 1 | 1 | 1 | 1 | 1 | 1 | 1 | 1 | 1 | 1 | 1 | 1 | 1 | 1 | 1 | 1 | 1 | 1 | 1 | 1 | 1 | 1 | 1 | 1 | 1 | 1 | 1 | 1 | 1 | 1 | 1 | 1 | 1 | 1 | 1 | 1 | 1 | 1 | 1 | 1 | 1 | 1 | 1 | 1 | 1 | 1 | 1 | 1 | 1 | 1 | 1 | 1 | 1 | 1 | 1 | 1 | 1 | 1 | 1 | 1 | 1 | 1 | 1 | 1 | 1 | 1 | 1 | 1 | 1 | 1 | 1 | 1 | 1 | 1 | 1 | 1 | 1 | 1 | 1 | 1 | 1 | 1 | 1 | 1 | 1 | 1 | 1 | 1 | 1 | 1 | 1 | 1 | 1 | 1 | 1 | 1 | 1 | 1 | 1 | 1 | 1 | 1 | 1 | 1 | 1 | 1 | 1 | 1 | 1 | 1 | 1 | 1 | 1 | 1 | 1 | 1 | 1 | 1 | 1 | 1 | 1 | 1 | 1 | 1 | 1 | 1 | 1 | 1 | 1 | 1 | 1 | 1 | 1 | 1 | 1 | 1 | 1 | 1 | 1 | 1 | 1 | 1 | 1 | 1 | 1 | 1 | 1 | 1 | 1 | 1 | 1 | 1 | 1 | 1 | 1 | 1 | 1 | 1 | 1 | 1 | 1 | 1 | 1 | 1 | 1 | 1 | 1 | 1 | 1 | 1 | 1 | 1 | 1 | 1 | 1 | 1 | 1 | 1 | 1 | 1 | 1 | 1 | 1 | 1 | 1 | 1 | 1 | 1 | 1 | 1 | 1 | 1 | 1 | 1 | 1 | 1 | 1 | 1 | 1 | 1 | 1 | 1 | 1 | 1 | 1 | 1 | 1 | 1 | 1 | 1 | 1 | 1 | 1 | 1 | 1 | 1 | 1 | 1 | 1 | 1 | 1 | 1 | 1 | 1 | 1 | 1 | 1 | 1 | 1 | 1 | 1 | 1 | 1 | 1 | 1 | 1 | 1 | 1 | 1 | 1 | 1 | 1 | 1 | 1 | 1 | 1 | 1 | 1 | 1 | 1 | 1 | 1 | 1 | 1 | 1 | 1 | 1 | 1 | 1 | 1 | 1 | 1 | 1 | 1 | 1 | 1 | 1 | 1 | 1 | 1 | 1 | 1 | 1 | 1 | 1 | 1 | 1 | 1 | 1 | 1 | 1 | 1 | 1 | 1 | 1 | 1 | 1 | 1 | 1 | 1 | 1 | 1 | 1 | 1 | 1 | 1 | 1 | 1 | 1 | 1 | 1 | 1 | 1 | 1 | 1 | 1 | 1 | 1 | 1 | 1 | 1 | 1 | 1 | 1 | 1 | 1 | 1 | 1 | 1 | 1 | 1 | 1 | 1 | 1 | 1 | 1 | 1 | 1 | 1 |
| Dalcera abrasa\* | 1 | 1 | 1 | 1 | 1 | 1 | 1 | 1 | 1 | 1 | 1 | 1 | 1 | 1 | 1 | 1 | 1 | 1 | 1 | 1 | 1 | 1 | 1 | 1 | 1 | 1 | 1 | 1 | 1 | 1 | 1 | 1 | 1 | 1 | 1 | 1 | 1 | 1 | 1 | 1 | 1 | 1 | 1 | 1 | 1 | 1 | 1 | 1 | 1 | 1 | 1 | 1 | 1 | 1 | 1 | 1 | 1 | 1 | 1 | 1 | 1 | 1 | 1 | 1 | 1 | 1 | 1 | 1 | 1 | 1 | 1 | 1 | 1 | 1 | 1 | 1 | 1 | 1 | 1 | 1 | 1 | 1 | 1 | 1 | 1 | 1 | 1 | 1 | 1 | 1 | 1 | 1 | 1 | 1 | 1 | 1 | 1 | 1 | 1 | 1 | 1 | 1 | 1 | 1 | 1 | 1 | 1 | 1 | 1 | 1 | 1 | 1 | 1 | 1 | 1 | 1 | 1 | 1 | 1 | 1 | 1 | 1 | 1 | 1 | 1 | 1 | 1 | 1 | 1 | 1 | 1 | 1 | 1 | 1 | 1 | 1 | 1 | 1 | 1 | 1 | 1 | 1 | 1 | 1 | 1 | 1 | 1 | 1 | 1 | 1 | 1 | 1 | 1 | 1 | 1 | 1 | 1 | 1 | 1 | 1 | 1 | 1 | 1 | 1 | 1 | 1 | 1 | 1 | 1 | 1 | 1 | 1 | 1 | 1 | 1 | 1 | 1 | 1 | 1 | 1 | 1 | 1 | 1 | 1 | 1 | 1 | 1 | 1 | 1 | 1 | 1 | 1 | 1 | 1 | 1 | 1 | 1 | 1 | 1 | 1 | 1 | 1 | 1 | 1 | 1 | 1 | 1 | 1 | 1 | 1 | 1 | 1 | 1 | 1 | 1 | 1 | 1 | 1 | 1 | 1 | 1 | 1 | 1 | 1 | 1 | 1 | 1 | 1 | 1 | 1 | 1 | 1 | 1 | 1 | 1 | 1 | 1 | 1 | 1 | 1 | 1 | 1 | 1 | 1 | 1 | 1 | 1 | 1 | 1 | 1 | 1 | 1 | 1 | 1 | 1 | 1 | 1 | 1 | 1 | 1 | 1 | 1 | 1 | 1 | 1 | 1 | 1 | 1 | 1 | 1 | 1 | 1 | 1 | 1 | 1 | 1 | 1 | 1 | 1 | 1 | 1 | 1 | 1 | 1 | 1 | 1 | 1 | 1 | 1 | 1 | 1 | 1 | 1 | 1 | 1 | 1 | 1 | 1 | 1 | 1 | 1 | 1 | 1 | 1 | 1 | 1 | 1 | 1 | 1 | 1 | 1 | 1 | 1 | 1 | 1 | 1 | 1 | 1 | 1 | 1 | 1 | 1 | 1 | 1 | 1 | 1 | 1 | 1 | 1 | 1 | 1 | 1 | 1 | 1 | 1 | 1 | 1 | 1 | 1 | 1 | 1 | 1 | 1 | 1 | 1 | 1 | 1 | 1 | 1 | 1 | 1 | 1 | 1 | 1 | 1 | 1 | 1 | 1 | 1 | 1 | 1 | 1 | 1 | 1 | 1 | 1 | 1 | 1 | 1 | 1 | 1 | 1 | 1 | 1 | 1 | 1 | 1 | 1 | 1 | 1 | 1 | 1 | 1 | 1 | 1 | 1 | 1 | 1 | 1 | 1 | 1 | 1 | 1 | 1 | 1 | 1 | 1 | 1 | 1 | 1 | 1 | 1 | 1 | 1 | 1 | 1 | 1 | 1 | 1 | 1 | 1 | 1 | 1 | 1 | 1 | 1 | 1 | 1 | 1 | 1 | 1 | 1 | 1 | 1 | 1 | 1 | 1 | 1 | 1 | 1 | 1 | 1 | 1 | 1 | 1 | 1 | 1 | 1 | 1 | 1 | 1 | 1 | 1 | 1 | 1 | 1 | 1 | 1 | 1 | 1 | 1 | 1 | 1 | 1 | 1 | 1 | 1 | 1 | 1 | 1 | 1 | 1 | 1 | 1 | 1 |
| Danaus plexippus\* | 1 | 1 | 1 | 1 | 1 | 1 | 1 | 1 | 1 | 1 | 1 | 1 | 1 | 1 | 1 | 1 | 1 | 1 | 1 | 1 | 1 | 1 | 1 | 1 | 1 | 1 | 1 | 1 | 1 | 1 | 1 | 1 | 1 | 1 | 1 | 1 | 1 | 1 | 1 | 1 | 1 | 1 | 1 | 1 | 1 | 1 | 1 | 1 | 1 | 1 | 1 | 1 | 1 | 1 | 1 | 1 | 1 | 1 | 1 | 1 | 1 | 1 | 1 | 1 | 1 | 1 | 1 | 1 | 1 | 1 | 1 | 1 | 1 | 1 | 1 | 1 | 1 | 1 | 1 | 1 | 1 | 1 | 1 | 1 | 1 | 1 | 1 | 1 | 1 | 1 | 1 | 1 | 1 | 1 | 1 | 1 | 1 | 1 | 1 | 1 | 1 | 1 | 1 | 1 | 1 | 1 | 1 | 1 | 1 | 1 | 1 | 1 | 1 | 1 | 1 | 1 | 1 | 1 | 1 | 1 | 1 | 1 | 1 | 1 | 1 | 1 | 1 | 1 | 1 | 1 | 1 | 1 | 1 | 1 | 1 | 1 | 1 | 1 | 1 | 1 | 1 | 1 | 1 | 1 | 1 | 1 | 1 | 1 | 1 | 1 | 1 | 1 | 1 | 1 | 1 | 1 | 1 | 1 | 1 | 1 | 1 | 1 | 1 | 1 | 1 | 1 | 1 | 1 | 1 | 1 | 1 | 1 | 1 | 1 | 1 | 1 | 1 | 1 | 1 | 1 | 1 | 1 | 1 | 1 | 1 | 1 | 1 | 1 | 1 | 1 | 1 | 1 | 1 | 1 | 1 | 1 | 1 | 1 | 1 | 1 | 1 | 1 | 1 | 1 | 1 | 1 | 1 | 1 | 1 | 1 | 1 | 1 | 1 | 1 | 1 | 1 | 1 | 1 | 1 | 1 | 1 | 1 | 1 | 1 | 1 | 1 | 1 | 1 | 1 | 1 | 1 | 1 | 1 | 1 | 1 | 1 | 1 | 1 | 1 | 1 | 1 | 1 | 1 | 1 | 1 | 1 | 1 | 1 | 1 | 1 | 1 | 1 | 1 | 1 | 1 | 1 | 1 | 1 | 1 | 1 | 1 | 1 | 1 | 1 | 1 | 1 | 1 | 1 | 1 | 1 | 1 | 1 | 1 | 1 | 1 | 1 | 1 | 1 | 1 | 1 | 1 | 1 | 1 | 1 | 1 | 1 | 1 | 1 | 1 | 1 | 1 | 1 | 1 | 1 | 1 | 1 | 1 | 1 | 1 | 1 | 1 | 1 | 1 | 1 | 1 | 1 | 1 | 1 | 1 | 1 | 1 | 1 | 1 | 1 | 1 | 1 | 1 | 1 | 1 | 1 | 1 | 1 | 1 | 1 | 1 | 1 | 1 | 1 | 1 | 1 | 1 | 1 | 1 | 1 | 1 | 1 | 1 | 1 | 1 | 1 | 1 | 1 | 1 | 1 | 1 | 1 | 1 | 1 | 1 | 1 | 1 | 1 | 1 | 1 | 1 | 1 | 1 | 1 | 1 | 1 | 1 | 1 | 1 | 1 | 1 | 1 | 1 | 1 | 1 | 1 | 1 | 1 | 1 | 1 | 1 | 1 | 1 | 1 | 1 | 1 | 1 | 1 | 1 | 1 | 1 | 1 | 1 | 1 | 1 | 1 | 1 | 1 | 1 | 1 | 1 | 1 | 1 | 1 | 1 | 1 | 1 | 1 | 1 | 1 | 1 | 1 | 1 | 1 | 1 | 1 | 1 | 1 | 1 | 1 | 1 | 1 | 1 | 1 | 1 | 1 | 1 | 1 | 1 | 1 | 1 | 1 | 1 | 1 | 1 | 1 | 1 | 1 | 1 | 1 | 1 | 1 | 1 | 1 | 1 | 1 | 1 | 1 | 1 | 1 | 1 | 1 | 1 | 1 | 1 | 1 | 1 | 1 | 1 | 1 | 1 | 1 | 1 | 1 | 1 | 1 | 1 | 1 | 1 | 1 | 1 |
| Grapholita dimorpha\* | 1 | 1 | 1 | 1 | 1 | 1 | 1 | 1 | 1 | 1 | 1 | 1 | 1 | 1 | 1 | 1 | 1 | 1 | 1 | 1 | 1 | 1 | 1 | 1 | 1 | 1 | 1 | 1 | 1 | 1 | 1 | 1 | 1 | 1 | 1 | 1 | 1 | 1 | 1 | 1 | 1 | 1 | 1 | 1 | 1 | 1 | 1 | 1 | 1 | 1 | 1 | 1 | 1 | 1 | 1 | 1 | 1 | 1 | 1 | 1 | 1 | 1 | 1 | 1 | 1 | 1 | 1 | 1 | 1 | 1 | 1 | 1 | 1 | 1 | 1 | 1 | 1 | 1 | 1 | 1 | 1 | 1 | 1 | 1 | 1 | 1 | 1 | 1 | 1 | 1 | 1 | 1 | 1 | 1 | 1 | 1 | 1 | 1 | 1 | 1 | 1 | 1 | 1 | 1 | 1 | 1 | 1 | 1 | 1 | 1 | 1 | 1 | 1 | 1 | 1 | 1 | 1 | 1 | 1 | 1 | 1 | 1 | 1 | 1 | 1 | 1 | 1 | 1 | 1 | 1 | 1 | 1 | 1 | 1 | 1 | 1 | 1 | 1 | 1 | 1 | 1 | 1 | 1 | 1 | 1 | 1 | 1 | 1 | 1 | 1 | 1 | 1 | 1 | 1 | 1 | 1 | 1 | 1 | 1 | 1 | 1 | 1 | 1 | 1 | 1 | 1 | 1 | 1 | 1 | 1 | 1 | 1 | 1 | 1 | 1 | 1 | 1 | 1 | 1 | 1 | 1 | 1 | 1 | 1 | 1 | 1 | 1 | 1 | 1 | 1 | 1 | 1 | 1 | 1 | 1 | 1 | 1 | 1 | 1 | 1 | 1 | 1 | 1 | 1 | 1 | 1 | 1 | 1 | 1 | 1 | 1 | 1 | 1 | 1 | 1 | 1 | 1 | 1 | 1 | 1 | 1 | 1 | 1 | 1 | 1 | 1 | 1 | 1 | 1 | 1 | 1 | 1 | 1 | 1 | 1 | 1 | 1 | 1 | 1 | 1 | 1 | 1 | 1 | 1 | 1 | 1 | 1 | 1 | 1 | 1 | 1 | 1 | 1 | 1 | 1 | 1 | 1 | 1 | 1 | 1 | 1 | 1 | 1 | 1 | 1 | 1 | 1 | 1 | 1 | 1 | 1 | 1 | 1 | 1 | 1 | 1 | 1 | 1 | 1 | 1 | 1 | 1 | 1 | 1 | 1 | 1 | 1 | 1 | 1 | 1 | 1 | 1 | 1 | 1 | 1 | 1 | 1 | 1 | 1 | 1 | 1 | 1 | 1 | 1 | 1 | 1 | 1 | 1 | 1 | 1 | 1 | 1 | 1 | 1 | 1 | 1 | 1 | 1 | 1 | 1 | 1 | 1 | 1 | 1 | 1 | 1 | 1 | 1 | 1 | 1 | 1 | 1 | 1 | 1 | 1 | 1 | 1 | 1 | 1 | 1 | 1 | 1 | 1 | 1 | 1 | 1 | 1 | 1 | 1 | 1 | 1 | 1 | 1 | 1 | 1 | 1 | 1 | 1 | 1 | 1 | 1 | 1 | 1 | 1 | 1 | 1 | 1 | 1 | 1 | 1 | 1 | 1 | 1 | 1 | 1 | 1 | 1 | 1 | 1 | 1 | 1 | 1 | 1 | 1 | 1 | 1 | 1 | 1 | 1 | 1 | 1 | 1 | 1 | 1 | 1 | 1 | 1 | 1 | 1 | 1 | 1 | 1 | 1 | 1 | 1 | 1 | 1 | 1 | 1 | 1 | 1 | 1 | 1 | 1 | 1 | 1 | 1 | 1 | 1 | 1 | 1 | 1 | 1 | 1 | 1 | 1 | 1 | 1 | 1 | 1 | 1 | 1 | 1 | 1 | 1 | 1 | 1 | 1 | 1 | 1 | 1 | 1 | 1 | 1 | 1 | 1 | 1 | 1 | 1 | 1 | 1 | 1 | 1 | 1 | 1 | 1 | 1 | 1 | 1 | 1 | 1 | 1 | 1 | 1 | 1 |
| Lacosoma ludolpha\* | 1 | 1 | 1 | 1 | 1 | 1 | 1 | 1 | 1 | 1 | 1 | 1 | 1 | 1 | 1 | 1 | 1 | 1 | 1 | 1 | 1 | 1 | 1 | 1 | 1 | 1 | 1 | 1 | 1 | 1 | 1 | 1 | 1 | 1 | 1 | 1 | 1 | 1 | 1 | 1 | 1 | 1 | 1 | 1 | 1 | 1 | 1 | 1 | 1 | 1 | 1 | 1 | 1 | 1 | 1 | 1 | 1 | 1 | 1 | 1 | 1 | 1 | 1 | 1 | 1 | 1 | 1 | 1 | 1 | 1 | 1 | 1 | 1 | 1 | 1 | 1 | 1 | 1 | 1 | 1 | 1 | 1 | 1 | 1 | 1 | 1 | 1 | 1 | 1 | 1 | 1 | 1 | 1 | 1 | 1 | 1 | 1 | 1 | 1 | 1 | 1 | 1 | 1 | 1 | 1 | 1 | 1 | 1 | 1 | 1 | 1 | 1 | 1 | 1 | 1 | 1 | 1 | 1 | 1 | 1 | 1 | 1 | 1 | 1 | 1 | 1 | 1 | 1 | 1 | 1 | 1 | 1 | 1 | 1 | 1 | 1 | 1 | 1 | 1 | 1 | 1 | 1 | 1 | 1 | 1 | 1 | 1 | 1 | 1 | 1 | 1 | 1 | 1 | 1 | 1 | 1 | 1 | 1 | 1 | 1 | 1 | 1 | 1 | 1 | 1 | 1 | 1 | 1 | 1 | 1 | 1 | 1 | 1 | 1 | 1 | 1 | 1 | 1 | 1 | 1 | 1 | 1 | 1 | 1 | 1 | 1 | 1 | 1 | 1 | 1 | 1 | 1 | 1 | 1 | 1 | 1 | 1 | 1 | 1 | 1 | 1 | 1 | 1 | 1 | 1 | 1 | 1 | 1 | 1 | 1 | 1 | 1 | 1 | 1 | 1 | 1 | 1 | 1 | 1 | 1 | 1 | 1 | 1 | 1 | 1 | 1 | 1 | 1 | 1 | 1 | 1 | 1 | 1 | 1 | 1 | 1 | 1 | 1 | 1 | 1 | 1 | 1 | 1 | 1 | 1 | 1 | 1 | 1 | 1 | 1 | 1 | 1 | 1 | 1 | 1 | 1 | 1 | 1 | 1 | 1 | 1 | 1 | 1 | 1 | 1 | 1 | 1 | 1 | 1 | 1 | 1 | 1 | 1 | 1 | 1 | 1 | 1 | 1 | 1 | 1 | 1 | 1 | 1 | 1 | 1 | 1 | 1 | 1 | 1 | 1 | 1 | 1 | 1 | 1 | 1 | 1 | 1 | 1 | 1 | 1 | 1 | 1 | 1 | 1 | 1 | 1 | 1 | 1 | 1 | 1 | 1 | 1 | 1 | 1 | 1 | 1 | 1 | 1 | 1 | 1 | 1 | 1 | 1 | 1 | 1 | 1 | 1 | 1 | 1 | 1 | 1 | 1 | 1 | 1 | 1 | 1 | 1 | 1 | 1 | 1 | 1 | 1 | 1 | 1 | 1 | 1 | 1 | 1 | 1 | 1 | 1 | 1 | 1 | 1 | 1 | 1 | 1 | 1 | 1 | 1 | 1 | 1 | 1 | 1 | 1 | 1 | 1 | 1 | 1 | 1 | 1 | 1 | 1 | 1 | 1 | 1 | 1 | 1 | 1 | 1 | 1 | 1 | 1 | 1 | 1 | 1 | 1 | 1 | 1 | 1 | 1 | 1 | 1 | 1 | 1 | 1 | 1 | 1 | 1 | 1 | 1 | 1 | 1 | 1 | 1 | 1 | 1 | 1 | 1 | 1 | 1 | 1 | 1 | 1 | 1 | 1 | 1 | 1 | 1 | 1 | 1 | 1 | 1 | 1 | 1 | 1 | 1 | 1 | 1 | 1 | 1 | 1 | 1 | 1 | 1 | 1 | 1 | 1 | 1 | 1 | 1 | 1 | 1 | 1 | 1 | 1 | 1 | 1 | 1 | 1 | 1 | 1 | 1 | 1 | 1 | 1 | 1 | 1 | 1 | 1 | 1 | 1 | 1 | 1 | 1 |
| Lantanophaga pusillidactyla\* | 1 | 1 | 1 | 1 | 1 | 1 | 1 | 1 | 1 | 1 | 1 | 1 | 1 | 1 | 1 | 1 | 1 | 1 | 1 | 1 | 1 | 1 | 1 | 1 | 1 | 1 | 1 | 1 | 1 | 1 | 1 | 1 | 1 | 1 | 1 | 1 | 1 | 1 | 1 | 1 | 1 | 1 | 1 | 1 | 1 | 1 | 1 | 1 | 1 | 1 | 1 | 1 | 1 | 1 | 1 | 1 | 1 | 1 | 1 | 1 | 1 | 1 | 1 | 1 | 1 | 1 | 1 | 1 | 1 | 1 | 1 | 1 | 1 | 1 | 1 | 1 | 1 | 1 | 1 | 1 | 1 | 1 | 1 | 1 | 1 | 1 | 1 | 1 | 1 | 1 | 1 | 1 | 1 | 1 | 1 | 1 | 1 | 1 | 1 | 1 | 1 | 1 | 1 | 1 | 1 | 1 | 1 | 1 | 1 | 1 | 1 | 1 | 1 | 1 | 1 | 1 | 1 | 1 | 1 | 1 | 1 | 1 | 1 | 1 | 1 | 1 | 1 | 1 | 1 | 1 | 1 | 1 | 1 | 1 | 1 | 1 | 1 | 1 | 1 | 1 | 1 | 1 | 1 | 1 | 1 | 1 | 1 | 1 | 1 | 1 | 1 | 1 | 1 | 1 | 1 | 1 | 1 | 1 | 1 | 1 | 1 | 1 | 1 | 1 | 1 | 1 | 1 | 1 | 1 | 1 | 1 | 1 | 1 | 1 | 1 | 1 | 1 | 1 | 1 | 1 | 1 | 1 | 1 | 1 | 1 | 1 | 1 | 1 | 1 | 1 | 1 | 1 | 1 | 1 | 1 | 1 | 1 | 1 | 1 | 1 | 1 | 1 | 1 | 1 | 1 | 1 | 1 | 1 | 1 | 1 | 1 | 1 | 1 | 1 | 1 | 1 | 1 | 1 | 1 | 1 | 1 | 1 | 1 | 1 | 1 | 1 | 1 | 1 | 1 | 1 | 1 | 1 | 1 | 1 | 1 | 1 | 1 | 1 | 1 | 1 | 1 | 1 | 1 | 1 | 1 | 1 | 1 | 1 | 1 | 1 | 1 | 1 | 1 | 1 | 1 | 1 | 1 | 1 | 1 | 1 | 1 | 1 | 1 | 1 | 1 | 1 | 1 | 1 | 1 | 1 | 1 | 1 | 1 | 1 | 1 | 1 | 1 | 1 | 1 | 1 | 1 | 1 | 1 | 1 | 1 | 1 | 1 | 1 | 1 | 1 | 1 | 1 | 1 | 1 | 1 | 1 | 1 | 1 | 1 | 1 | 1 | 1 | 1 | 1 | 1 | 1 | 1 | 1 | 1 | 1 | 1 | 1 | 1 | 1 | 1 | 1 | 1 | 1 | 1 | 1 | 1 | 1 | 1 | 1 | 1 | 1 | 1 | 1 | 1 | 1 | 1 | 1 | 1 | 1 | 1 | 1 | 1 | 1 | 1 | 1 | 1 | 1 | 1 | 1 | 1 | 1 | 1 | 1 | 1 | 1 | 1 | 1 | 1 | 1 | 1 | 1 | 1 | 1 | 1 | 1 | 1 | 1 | 1 | 1 | 1 | 1 | 1 | 1 | 1 | 1 | 1 | 1 | 1 | 1 | 1 | 1 | 1 | 1 | 1 | 1 | 1 | 1 | 1 | 1 | 1 | 1 | 1 | 1 | 1 | 1 | 1 | 1 | 1 | 1 | 1 | 1 | 1 | 1 | 1 | 1 | 1 | 1 | 1 | 1 | 1 | 1 | 1 | 1 | 1 | 1 | 1 | 1 | 1 | 1 | 1 | 1 | 1 | 1 | 1 | 1 | 1 | 1 | 1 | 1 | 1 | 1 | 1 | 1 | 1 | 1 | 1 | 1 | 1 | 1 | 1 | 1 | 1 | 1 | 1 | 1 | 1 | 1 | 1 | 1 | 1 | 1 | 1 | 1 | 1 | 1 | 1 | 1 | 1 | 1 | 1 | 1 | 1 | 1 | 1 | 1 | 1 | 1 | 1 | 1 | 1 |
| Lyssa zampa\* | 1 | 1 | 1 | 1 | 1 | 1 | 1 | 1 | 1 | 1 | 1 | 1 | 1 | 1 | 1 | 1 | 1 | 1 | 1 | 1 | 1 | 1 | 1 | 1 | 1 | 1 | 1 | 1 | 1 | 1 | 1 | 1 | 1 | 1 | 1 | 1 | 1 | 1 | 1 | 1 | 1 | 1 | 1 | 1 | 1 | 1 | 1 | 1 | 1 | 1 | 1 | 1 | 1 | 1 | 1 | 1 | 1 | 1 | 1 | 1 | 1 | 1 | 1 | 1 | 1 | 1 | 1 | 1 | 1 | 1 | 1 | 1 | 1 | 1 | 1 | 1 | 1 | 1 | 1 | 1 | 1 | 1 | 1 | 1 | 1 | 1 | 1 | 1 | 1 | 1 | 1 | 1 | 1 | 1 | 1 | 1 | 1 | 1 | 1 | 1 | 1 | 1 | 1 | 1 | 1 | 1 | 1 | 1 | 1 | 1 | 1 | 1 | 1 | 1 | 1 | 1 | 1 | 1 | 1 | 1 | 1 | 1 | 1 | 1 | 1 | 1 | 1 | 1 | 1 | 1 | 1 | 1 | 1 | 1 | 1 | 1 | 1 | 1 | 1 | 1 | 1 | 1 | 1 | 1 | 1 | 1 | 1 | 1 | 1 | 1 | 1 | 1 | 1 | 1 | 1 | 1 | 1 | 1 | 1 | 1 | 1 | 1 | 1 | 1 | 1 | 1 | 1 | 1 | 1 | 1 | 1 | 1 | 1 | 1 | 1 | 1 | 1 | 1 | 1 | 1 | 1 | 1 | 1 | 1 | 1 | 1 | 1 | 1 | 1 | 1 | 1 | 1 | 1 | 1 | 1 | 1 | 1 | 1 | 1 | 1 | 1 | 1 | 1 | 1 | 1 | 1 | 1 | 1 | 1 | 1 | 1 | 1 | 1 | 1 | 1 | 1 | 1 | 1 | 1 | 1 | 1 | 1 | 1 | 1 | 1 | 1 | 1 | 1 | 1 | 1 | 1 | 1 | 1 | 1 | 1 | 1 | 1 | 1 | 1 | 1 | 1 | 1 | 1 | 1 | 1 | 1 | 1 | 1 | 1 | 1 | 1 | 1 | 1 | 1 | 1 | 1 | 1 | 1 | 1 | 1 | 1 | 1 | 1 | 1 | 1 | 1 | 1 | 1 | 1 | 1 | 1 | 1 | 1 | 1 | 1 | 1 | 1 | 1 | 1 | 1 | 1 | 1 | 1 | 1 | 1 | 1 | 1 | 1 | 1 | 1 | 1 | 1 | 1 | 1 | 1 | 1 | 1 | 1 | 1 | 1 | 1 | 1 | 1 | 1 | 1 | 1 | 1 | 1 | 1 | 1 | 1 | 1 | 1 | 1 | 1 | 1 | 1 | 1 | 1 | 1 | 1 | 1 | 1 | 1 | 1 | 1 | 1 | 1 | 1 | 1 | 1 | 1 | 1 | 1 | 1 | 1 | 1 | 1 | 1 | 1 | 1 | 1 | 1 | 1 | 1 | 1 | 1 | 1 | 1 | 1 | 1 | 1 | 1 | 1 | 1 | 1 | 1 | 1 | 1 | 1 | 1 | 1 | 1 | 1 | 1 | 1 | 1 | 1 | 1 | 1 | 1 | 1 | 1 | 1 | 1 | 1 | 1 | 1 | 1 | 1 | 1 | 1 | 1 | 1 | 1 | 1 | 1 | 1 | 1 | 1 | 1 | 1 | 1 | 1 | 1 | 1 | 1 | 1 | 1 | 1 | 1 | 1 | 1 | 1 | 1 | 1 | 1 | 1 | 1 | 1 | 1 | 1 | 1 | 1 | 1 | 1 | 1 | 1 | 1 | 1 | 1 | 1 | 1 | 1 | 1 | 1 | 1 | 1 | 1 | 1 | 1 | 1 | 1 | 1 | 1 | 1 | 1 | 1 | 1 | 1 | 1 | 1 | 1 | 1 | 1 | 1 | 1 | 1 | 1 | 1 | 1 | 1 | 1 | 1 | 1 | 1 | 1 | 1 | 1 | 1 | 1 | 1 | 1 | 1 | 1 |
| Macrosoma sp.\* | 1 | 1 | 1 | 1 | 1 | 1 | 1 | 1 | 1 | 1 | 1 | 1 | 1 | 1 | 1 | 1 | 1 | 1 | 1 | 1 | 1 | 1 | 1 | 1 | 1 | 1 | 1 | 1 | 1 | 1 | 1 | 1 | 1 | 1 | 1 | 1 | 1 | 1 | 1 | 1 | 1 | 1 | 1 | 1 | 1 | 1 | 1 | 1 | 1 | 1 | 1 | 1 | 1 | 1 | 1 | 1 | 1 | 1 | 1 | 1 | 1 | 1 | 1 | 1 | 1 | 1 | 1 | 1 | 1 | 1 | 1 | 1 | 1 | 1 | 1 | 1 | 1 | 1 | 1 | 1 | 1 | 1 | 1 | 1 | 1 | 1 | 1 | 1 | 1 | 1 | 1 | 1 | 1 | 1 | 1 | 1 | 1 | 1 | 1 | 1 | 1 | 1 | 1 | 1 | 1 | 1 | 1 | 1 | 1 | 1 | 1 | 1 | 1 | 1 | 1 | 1 | 1 | 1 | 1 | 1 | 1 | 1 | 1 | 1 | 1 | 1 | 1 | 1 | 1 | 1 | 1 | 1 | 1 | 1 | 1 | 1 | 1 | 1 | 1 | 1 | 1 | 1 | 1 | 1 | 1 | 1 | 1 | 1 | 1 | 1 | 1 | 1 | 1 | 1 | 1 | 1 | 1 | 1 | 1 | 1 | 1 | 1 | 1 | 1 | 1 | 1 | 1 | 1 | 1 | 1 | 1 | 1 | 1 | 1 | 1 | 1 | 1 | 1 | 1 | 1 | 1 | 1 | 1 | 1 | 1 | 1 | 1 | 1 | 1 | 1 | 1 | 1 | 1 | 1 | 1 | 1 | 1 | 1 | 1 | 1 | 1 | 1 | 1 | 1 | 1 | 1 | 1 | 1 | 1 | 1 | 1 | 1 | 1 | 1 | 1 | 1 | 1 | 1 | 1 | 1 | 1 | 1 | 1 | 1 | 1 | 1 | 1 | 1 | 1 | 1 | 1 | 1 | 1 | 1 | 1 | 1 | 1 | 1 | 1 | 1 | 1 | 1 | 1 | 1 | 1 | 1 | 1 | 1 | 1 | 1 | 1 | 1 | 1 | 1 | 1 | 1 | 1 | 1 | 1 | 1 | 1 | 1 | 1 | 1 | 1 | 1 | 1 | 1 | 1 | 1 | 1 | 1 | 1 | 1 | 1 | 1 | 1 | 1 | 1 | 1 | 1 | 1 | 1 | 1 | 1 | 1 | 1 | 1 | 1 | 1 | 1 | 1 | 1 | 1 | 1 | 1 | 1 | 1 | 1 | 1 | 1 | 1 | 1 | 1 | 1 | 1 | 1 | 1 | 1 | 1 | 1 | 1 | 1 | 1 | 1 | 1 | 1 | 1 | 1 | 1 | 1 | 1 | 1 | 1 | 1 | 1 | 1 | 1 | 1 | 1 | 1 | 1 | 1 | 1 | 1 | 1 | 1 | 1 | 1 | 1 | 1 | 1 | 1 | 1 | 1 | 1 | 1 | 1 | 1 | 1 | 1 | 1 | 1 | 1 | 1 | 1 | 1 | 1 | 1 | 1 | 1 | 1 | 1 | 1 | 1 | 1 | 1 | 1 | 1 | 1 | 1 | 1 | 1 | 1 | 1 | 1 | 1 | 1 | 1 | 1 | 1 | 1 | 1 | 1 | 1 | 1 | 1 | 1 | 1 | 1 | 1 | 1 | 1 | 1 | 1 | 1 | 1 | 1 | 1 | 1 | 1 | 1 | 1 | 1 | 1 | 1 | 1 | 1 | 1 | 1 | 1 | 1 | 1 | 1 | 1 | 1 | 1 | 1 | 1 | 1 | 1 | 1 | 1 | 1 | 1 | 1 | 1 | 1 | 1 | 1 | 1 | 1 | 1 | 1 | 1 | 1 | 1 | 1 | 1 | 1 | 1 | 1 | 1 | 1 | 1 | 1 | 1 | 1 | 1 | 1 | 1 | 1 | 1 | 1 | 1 | 1 | 1 | 1 | 1 | 1 | 1 | 1 | 1 | 1 | 1 |
| Manduca sexta\* | 1 | 1 | 1 | 1 | 1 | 1 | 1 | 1 | 1 | 1 | 1 | 1 | 1 | 1 | 1 | 1 | 1 | 1 | 1 | 1 | 1 | 1 | 1 | 1 | 1 | 1 | 1 | 1 | 1 | 1 | 1 | 1 | 1 | 1 | 1 | 1 | 1 | 1 | 1 | 1 | 1 | 1 | 1 | 1 | 1 | 1 | 1 | 1 | 1 | 1 | 1 | 1 | 1 | 1 | 1 | 1 | 1 | 1 | 1 | 1 | 1 | 1 | 1 | 1 | 1 | 1 | 1 | 1 | 1 | 1 | 1 | 1 | 1 | 1 | 1 | 1 | 1 | 1 | 1 | 1 | 1 | 1 | 1 | 1 | 1 | 1 | 1 | 1 | 1 | 1 | 1 | 1 | 1 | 1 | 1 | 1 | 1 | 1 | 1 | 1 | 1 | 1 | 1 | 1 | 1 | 1 | 1 | 1 | 1 | 1 | 1 | 1 | 1 | 1 | 1 | 1 | 1 | 1 | 1 | 1 | 1 | 1 | 1 | 1 | 1 | 1 | 1 | 1 | 1 | 1 | 1 | 1 | 1 | 1 | 1 | 1 | 1 | 1 | 1 | 1 | 1 | 1 | 1 | 1 | 1 | 1 | 1 | 1 | 1 | 1 | 1 | 1 | 1 | 1 | 1 | 1 | 1 | 1 | 1 | 1 | 1 | 1 | 1 | 1 | 1 | 1 | 1 | 1 | 1 | 1 | 1 | 1 | 1 | 1 | 1 | 1 | 1 | 1 | 1 | 1 | 1 | 1 | 1 | 1 | 1 | 1 | 1 | 1 | 1 | 1 | 1 | 1 | 1 | 1 | 1 | 1 | 1 | 1 | 1 | 1 | 1 | 1 | 1 | 1 | 1 | 1 | 1 | 1 | 1 | 1 | 1 | 1 | 1 | 1 | 1 | 1 | 1 | 1 | 1 | 1 | 1 | 1 | 1 | 1 | 1 | 1 | 1 | 1 | 1 | 1 | 1 | 1 | 1 | 1 | 1 | 1 | 1 | 1 | 1 | 1 | 1 | 1 | 1 | 1 | 1 | 1 | 1 | 1 | 1 | 1 | 1 | 1 | 1 | 1 | 1 | 1 | 1 | 1 | 1 | 1 | 1 | 1 | 1 | 1 | 1 | 1 | 1 | 1 | 1 | 1 | 1 | 1 | 1 | 1 | 1 | 1 | 1 | 1 | 1 | 1 | 1 | 1 | 1 | 1 | 1 | 1 | 1 | 1 | 1 | 1 | 1 | 1 | 1 | 1 | 1 | 1 | 1 | 1 | 1 | 1 | 1 | 1 | 1 | 1 | 1 | 1 | 1 | 1 | 1 | 1 | 1 | 1 | 1 | 1 | 1 | 1 | 1 | 1 | 1 | 1 | 1 | 1 | 1 | 1 | 1 | 1 | 1 | 1 | 1 | 1 | 1 | 1 | 1 | 1 | 1 | 1 | 1 | 1 | 1 | 1 | 1 | 1 | 1 | 1 | 1 | 1 | 1 | 1 | 1 | 1 | 1 | 1 | 1 | 1 | 1 | 1 | 1 | 1 | 1 | 1 | 1 | 1 | 1 | 1 | 1 | 1 | 1 | 1 | 1 | 1 | 1 | 1 | 1 | 1 | 1 | 1 | 1 | 1 | 1 | 1 | 1 | 1 | 1 | 1 | 1 | 1 | 1 | 1 | 1 | 1 | 1 | 1 | 1 | 1 | 1 | 1 | 1 | 1 | 1 | 1 | 1 | 1 | 1 | 1 | 1 | 1 | 1 | 1 | 1 | 1 | 1 | 1 | 1 | 1 | 1 | 1 | 1 | 1 | 1 | 1 | 1 | 1 | 1 | 1 | 1 | 1 | 1 | 1 | 1 | 1 | 1 | 1 | 1 | 1 | 1 | 1 | 1 | 1 | 1 | 1 | 1 | 1 | 1 | 1 | 1 | 1 | 1 | 1 | 1 | 1 | 1 | 1 | 1 | 1 | 1 | 1 | 1 | 1 | 1 | 1 | 1 | 1 | 1 | 1 | 1 |
| Megalopyge tharops\* | 1 | 1 | 1 | 1 | 1 | 1 | 1 | 1 | 1 | 1 | 1 | 1 | 1 | 1 | 1 | 1 | 1 | 1 | 1 | 1 | 1 | 1 | 1 | 1 | 1 | 1 | 1 | 1 | 1 | 1 | 1 | 1 | 1 | 1 | 1 | 1 | 1 | 1 | 1 | 1 | 1 | 1 | 1 | 1 | 1 | 1 | 1 | 1 | 1 | 1 | 1 | 1 | 1 | 1 | 1 | 1 | 1 | 1 | 1 | 1 | 1 | 1 | 1 | 1 | 1 | 1 | 1 | 1 | 1 | 1 | 1 | 1 | 1 | 1 | 1 | 1 | 1 | 1 | 1 | 1 | 1 | 1 | 1 | 1 | 1 | 1 | 1 | 1 | 1 | 1 | 1 | 1 | 1 | 1 | 1 | 1 | 1 | 1 | 1 | 1 | 1 | 1 | 1 | 1 | 1 | 1 | 1 | 1 | 1 | 1 | 1 | 1 | 1 | 1 | 1 | 1 | 1 | 1 | 1 | 1 | 1 | 1 | 1 | 1 | 1 | 1 | 1 | 1 | 1 | 1 | 1 | 1 | 1 | 1 | 1 | 1 | 1 | 1 | 1 | 1 | 1 | 1 | 1 | 1 | 1 | 1 | 1 | 1 | 1 | 1 | 1 | 1 | 1 | 1 | 1 | 1 | 1 | 1 | 1 | 1 | 1 | 1 | 1 | 1 | 1 | 1 | 1 | 1 | 1 | 1 | 1 | 1 | 1 | 1 | 1 | 1 | 1 | 1 | 1 | 1 | 1 | 1 | 1 | 1 | 1 | 1 | 1 | 1 | 1 | 1 | 1 | 1 | 1 | 1 | 1 | 1 | 1 | 1 | 1 | 1 | 1 | 1 | 1 | 1 | 1 | 1 | 1 | 1 | 1 | 1 | 1 | 1 | 1 | 1 | 1 | 1 | 1 | 1 | 1 | 1 | 1 | 1 | 1 | 1 | 1 | 1 | 1 | 1 | 1 | 1 | 1 | 1 | 1 | 1 | 1 | 1 | 1 | 1 | 1 | 1 | 1 | 1 | 1 | 1 | 1 | 1 | 1 | 1 | 1 | 1 | 1 | 1 | 1 | 1 | 1 | 1 | 1 | 1 | 1 | 1 | 1 | 1 | 1 | 1 | 1 | 1 | 1 | 1 | 1 | 1 | 1 | 1 | 1 | 1 | 1 | 1 | 1 | 1 | 1 | 1 | 1 | 1 | 1 | 1 | 1 | 1 | 1 | 1 | 1 | 1 | 1 | 1 | 1 | 1 | 1 | 1 | 1 | 1 | 1 | 1 | 1 | 1 | 1 | 1 | 1 | 1 | 1 | 1 | 1 | 1 | 1 | 1 | 1 | 1 | 1 | 1 | 1 | 1 | 1 | 1 | 1 | 1 | 1 | 1 | 1 | 1 | 1 | 1 | 1 | 1 | 1 | 1 | 1 | 1 | 1 | 1 | 1 | 1 | 1 | 1 | 1 | 1 | 1 | 1 | 1 | 1 | 1 | 1 | 1 | 1 | 1 | 1 | 1 | 1 | 1 | 1 | 1 | 1 | 1 | 1 | 1 | 1 | 1 | 1 | 1 | 1 | 1 | 1 | 1 | 1 | 1 | 1 | 1 | 1 | 1 | 1 | 1 | 1 | 1 | 1 | 1 | 1 | 1 | 1 | 1 | 1 | 1 | 1 | 1 | 1 | 1 | 1 | 1 | 1 | 1 | 1 | 1 | 1 | 1 | 1 | 1 | 1 | 1 | 1 | 1 | 1 | 1 | 1 | 1 | 1 | 1 | 1 | 1 | 1 | 1 | 1 | 1 | 1 | 1 | 1 | 1 | 1 | 1 | 1 | 1 | 1 | 1 | 1 | 1 | 1 | 1 | 1 | 1 | 1 | 1 | 1 | 1 | 1 | 1 | 1 | 1 | 1 | 1 | 1 | 1 | 1 | 1 | 1 | 1 | 1 | 1 | 1 | 1 | 1 | 1 | 1 | 1 | 1 | 1 | 1 | 1 | 1 | 1 | 1 | 1 |
| Megathymus yuccae\* | 1 | 1 | 1 | 1 | 1 | 1 | 1 | 1 | 1 | 1 | 1 | 1 | 1 | 1 | 1 | 1 | 1 | 1 | 1 | 1 | 1 | 1 | 1 | 1 | 1 | 1 | 1 | 1 | 1 | 1 | 1 | 1 | 1 | 1 | 1 | 1 | 1 | 1 | 1 | 1 | 1 | 1 | 1 | 1 | 1 | 1 | 1 | 1 | 1 | 1 | 1 | 1 | 1 | 1 | 1 | 1 | 1 | 1 | 1 | 1 | 1 | 1 | 1 | 1 | 1 | 1 | 1 | 1 | 1 | 1 | 1 | 1 | 1 | 1 | 1 | 1 | 1 | 1 | 1 | 1 | 1 | 1 | 1 | 1 | 1 | 1 | 1 | 1 | 1 | 1 | 1 | 1 | 1 | 1 | 1 | 1 | 1 | 1 | 1 | 1 | 1 | 1 | 1 | 1 | 1 | 1 | 1 | 1 | 1 | 1 | 1 | 1 | 1 | 1 | 1 | 1 | 1 | 1 | 1 | 1 | 1 | 1 | 1 | 1 | 1 | 1 | 1 | 1 | 1 | 1 | 1 | 1 | 1 | 1 | 1 | 1 | 1 | 1 | 1 | 1 | 1 | 1 | 1 | 1 | 1 | 1 | 1 | 1 | 1 | 1 | 1 | 1 | 1 | 1 | 1 | 1 | 1 | 1 | 1 | 1 | 1 | 1 | 1 | 1 | 1 | 1 | 1 | 1 | 1 | 1 | 1 | 1 | 1 | 1 | 1 | 1 | 1 | 1 | 1 | 1 | 1 | 1 | 1 | 1 | 1 | 1 | 1 | 1 | 1 | 1 | 1 | 1 | 1 | 1 | 1 | 1 | 1 | 1 | 1 | 1 | 1 | 1 | 1 | 1 | 1 | 1 | 1 | 1 | 1 | 1 | 1 | 1 | 1 | 1 | 1 | 1 | 1 | 1 | 1 | 1 | 1 | 1 | 1 | 1 | 1 | 1 | 1 | 1 | 1 | 1 | 1 | 1 | 1 | 1 | 1 | 1 | 1 | 1 | 1 | 1 | 1 | 1 | 1 | 1 | 1 | 1 | 1 | 1 | 1 | 1 | 1 | 1 | 1 | 1 | 1 | 1 | 1 | 1 | 1 | 1 | 1 | 1 | 1 | 1 | 1 | 1 | 1 | 1 | 1 | 1 | 1 | 1 | 1 | 1 | 1 | 1 | 1 | 1 | 1 | 1 | 1 | 1 | 1 | 1 | 1 | 1 | 1 | 1 | 1 | 1 | 1 | 1 | 1 | 1 | 1 | 1 | 1 | 1 | 1 | 1 | 1 | 1 | 1 | 1 | 1 | 1 | 1 | 1 | 1 | 1 | 1 | 1 | 1 | 1 | 1 | 1 | 1 | 1 | 1 | 1 | 1 | 1 | 1 | 1 | 1 | 1 | 1 | 1 | 1 | 1 | 1 | 1 | 1 | 1 | 1 | 1 | 1 | 1 | 1 | 1 | 1 | 1 | 1 | 1 | 1 | 1 | 1 | 1 | 1 | 1 | 1 | 1 | 1 | 1 | 1 | 1 | 1 | 1 | 1 | 1 | 1 | 1 | 1 | 1 | 1 | 1 | 1 | 1 | 1 | 1 | 1 | 1 | 1 | 1 | 1 | 1 | 1 | 1 | 1 | 1 | 1 | 1 | 1 | 1 | 1 | 1 | 1 | 1 | 1 | 1 | 1 | 1 | 1 | 1 | 1 | 1 | 1 | 1 | 1 | 1 | 1 | 1 | 1 | 1 | 1 | 1 | 1 | 1 | 1 | 1 | 1 | 1 | 1 | 1 | 1 | 1 | 1 | 1 | 1 | 1 | 1 | 1 | 1 | 1 | 1 | 1 | 1 | 1 | 1 | 1 | 1 | 1 | 1 | 1 | 1 | 1 | 1 | 1 | 1 | 1 | 1 | 1 | 1 | 1 | 1 | 1 | 1 | 1 | 1 | 1 | 1 | 1 | 1 | 1 | 1 | 1 | 1 | 1 | 1 | 1 | 1 | 1 | 1 | 1 | 1 |
| Morpheis mathani\* | 1 | 1 | 1 | 1 | 1 | 1 | 1 | 1 | 1 | 1 | 1 | 1 | 1 | 1 | 1 | 1 | 1 | 1 | 1 | 1 | 1 | 1 | 1 | 1 | 1 | 1 | 1 | 1 | 1 | 1 | 1 | 1 | 1 | 1 | 1 | 1 | 1 | 1 | 1 | 1 | 1 | 1 | 1 | 1 | 1 | 1 | 1 | 1 | 1 | 1 | 1 | 1 | 1 | 1 | 1 | 1 | 1 | 1 | 1 | 1 | 1 | 1 | 1 | 1 | 1 | 1 | 1 | 1 | 1 | 1 | 1 | 1 | 1 | 1 | 1 | 1 | 1 | 1 | 1 | 1 | 1 | 1 | 1 | 1 | 1 | 1 | 1 | 1 | 1 | 1 | 1 | 1 | 1 | 1 | 1 | 1 | 1 | 1 | 1 | 1 | 1 | 1 | 1 | 1 | 1 | 1 | 1 | 1 | 1 | 1 | 1 | 1 | 1 | 1 | 1 | 1 | 1 | 1 | 1 | 1 | 1 | 1 | 1 | 1 | 1 | 1 | 1 | 1 | 1 | 1 | 1 | 1 | 1 | 1 | 1 | 1 | 1 | 1 | 1 | 1 | 1 | 1 | 1 | 1 | 1 | 1 | 1 | 1 | 1 | 1 | 1 | 1 | 1 | 1 | 1 | 1 | 1 | 1 | 1 | 1 | 1 | 1 | 1 | 1 | 1 | 1 | 1 | 1 | 1 | 1 | 1 | 1 | 1 | 1 | 1 | 1 | 1 | 1 | 1 | 1 | 1 | 1 | 1 | 1 | 1 | 1 | 1 | 1 | 1 | 1 | 1 | 1 | 1 | 1 | 1 | 1 | 1 | 1 | 1 | 1 | 1 | 1 | 1 | 1 | 1 | 1 | 1 | 1 | 1 | 1 | 1 | 1 | 1 | 1 | 1 | 1 | 1 | 1 | 1 | 1 | 1 | 1 | 1 | 1 | 1 | 1 | 1 | 1 | 1 | 1 | 1 | 1 | 1 | 1 | 1 | 1 | 1 | 1 | 1 | 1 | 1 | 1 | 1 | 1 | 1 | 1 | 1 | 1 | 1 | 1 | 1 | 1 | 1 | 1 | 1 | 1 | 1 | 1 | 1 | 1 | 1 | 1 | 1 | 1 | 1 | 1 | 1 | 1 | 1 | 1 | 1 | 1 | 1 | 1 | 1 | 1 | 1 | 1 | 1 | 1 | 1 | 1 | 1 | 1 | 1 | 1 | 1 | 1 | 1 | 1 | 1 | 1 | 1 | 1 | 1 | 1 | 1 | 1 | 1 | 1 | 1 | 1 | 1 | 1 | 1 | 1 | 1 | 1 | 1 | 1 | 1 | 1 | 1 | 1 | 1 | 1 | 1 | 1 | 1 | 1 | 1 | 1 | 1 | 1 | 1 | 1 | 1 | 1 | 1 | 1 | 1 | 1 | 1 | 1 | 1 | 1 | 1 | 1 | 1 | 1 | 1 | 1 | 1 | 1 | 1 | 1 | 1 | 1 | 1 | 1 | 1 | 1 | 1 | 1 | 1 | 1 | 1 | 1 | 1 | 1 | 1 | 1 | 1 | 1 | 1 | 1 | 1 | 1 | 1 | 1 | 1 | 1 | 1 | 1 | 1 | 1 | 1 | 1 | 1 | 1 | 1 | 1 | 1 | 1 | 1 | 1 | 1 | 1 | 1 | 1 | 1 | 1 | 1 | 1 | 1 | 1 | 1 | 1 | 1 | 1 | 1 | 1 | 1 | 1 | 1 | 1 | 1 | 1 | 1 | 1 | 1 | 1 | 1 | 1 | 1 | 1 | 1 | 1 | 1 | 1 | 1 | 1 | 1 | 1 | 1 | 1 | 1 | 1 | 1 | 1 | 1 | 1 | 1 | 1 | 1 | 1 | 1 | 1 | 1 | 1 | 1 | 1 | 1 | 1 | 1 | 1 | 1 | 1 | 1 | 1 | 1 | 1 | 1 | 1 | 1 | 1 | 1 | 1 | 1 | 1 | 1 | 1 | 1 | 1 | 1 |
| Myelobia sp.\* | 1 | 1 | 1 | 1 | 1 | 1 | 1 | 1 | 1 | 1 | 1 | 1 | 1 | 1 | 1 | 1 | 1 | 1 | 1 | 1 | 1 | 1 | 1 | 1 | 1 | 1 | 1 | 1 | 1 | 1 | 1 | 1 | 1 | 1 | 1 | 1 | 1 | 1 | 1 | 1 | 1 | 1 | 1 | 1 | 1 | 1 | 1 | 1 | 1 | 1 | 1 | 1 | 1 | 1 | 1 | 1 | 1 | 1 | 1 | 1 | 1 | 1 | 1 | 1 | 1 | 1 | 1 | 1 | 1 | 1 | 1 | 1 | 1 | 1 | 1 | 1 | 1 | 1 | 1 | 1 | 1 | 1 | 1 | 1 | 1 | 1 | 1 | 1 | 1 | 1 | 1 | 1 | 1 | 1 | 1 | 1 | 1 | 1 | 1 | 1 | 1 | 1 | 1 | 1 | 1 | 1 | 1 | 1 | 1 | 1 | 1 | 1 | 1 | 1 | 1 | 1 | 1 | 1 | 1 | 1 | 1 | 1 | 1 | 1 | 1 | 1 | 1 | 1 | 1 | 1 | 1 | 1 | 1 | 1 | 1 | 1 | 1 | 1 | 1 | 1 | 1 | 1 | 1 | 1 | 1 | 1 | 1 | 1 | 1 | 1 | 1 | 1 | 1 | 1 | 1 | 1 | 1 | 1 | 1 | 1 | 1 | 1 | 1 | 1 | 1 | 1 | 1 | 1 | 1 | 1 | 1 | 1 | 1 | 1 | 1 | 1 | 1 | 1 | 1 | 1 | 1 | 1 | 1 | 1 | 1 | 1 | 1 | 1 | 1 | 1 | 1 | 1 | 1 | 1 | 1 | 1 | 1 | 1 | 1 | 1 | 1 | 1 | 1 | 1 | 1 | 1 | 1 | 1 | 1 | 1 | 1 | 1 | 1 | 1 | 1 | 1 | 1 | 1 | 1 | 1 | 1 | 1 | 1 | 1 | 1 | 1 | 1 | 1 | 1 | 1 | 1 | 1 | 1 | 1 | 1 | 1 | 1 | 1 | 1 | 1 | 1 | 1 | 1 | 1 | 1 | 1 | 1 | 1 | 1 | 1 | 1 | 1 | 1 | 1 | 1 | 1 | 1 | 1 | 1 | 1 | 1 | 1 | 1 | 1 | 1 | 1 | 1 | 1 | 1 | 1 | 1 | 1 | 1 | 1 | 1 | 1 | 1 | 1 | 1 | 1 | 1 | 1 | 1 | 1 | 1 | 1 | 1 | 1 | 1 | 1 | 1 | 1 | 1 | 1 | 1 | 1 | 1 | 1 | 1 | 1 | 1 | 1 | 1 | 1 | 1 | 1 | 1 | 1 | 1 | 1 | 1 | 1 | 1 | 1 | 1 | 1 | 1 | 1 | 1 | 1 | 1 | 1 | 1 | 1 | 1 | 1 | 1 | 1 | 1 | 1 | 1 | 1 | 1 | 1 | 1 | 1 | 1 | 1 | 1 | 1 | 1 | 1 | 1 | 1 | 1 | 1 | 1 | 1 | 1 | 1 | 1 | 1 | 1 | 1 | 1 | 1 | 1 | 1 | 1 | 1 | 1 | 1 | 1 | 1 | 1 | 1 | 1 | 1 | 1 | 1 | 1 | 1 | 1 | 1 | 1 | 1 | 1 | 1 | 1 | 1 | 1 | 1 | 1 | 1 | 1 | 1 | 1 | 1 | 1 | 1 | 1 | 1 | 1 | 1 | 1 | 1 | 1 | 1 | 1 | 1 | 1 | 1 | 1 | 1 | 1 | 1 | 1 | 1 | 1 | 1 | 1 | 1 | 1 | 1 | 1 | 1 | 1 | 1 | 1 | 1 | 1 | 1 | 1 | 1 | 1 | 1 | 1 | 1 | 1 | 1 | 1 | 1 | 1 | 1 | 1 | 1 | 1 | 1 | 1 | 1 | 1 | 1 | 1 | 1 | 1 | 1 | 1 | 1 | 1 | 1 | 1 | 1 | 1 | 1 | 1 | 1 | 1 | 1 | 1 | 1 | 1 | 1 | 1 | 1 | 1 |
| Nemoria lixaria\* | 1 | 1 | 1 | 1 | 1 | 1 | 1 | 1 | 1 | 1 | 1 | 1 | 1 | 1 | 1 | 1 | 1 | 1 | 1 | 1 | 1 | 1 | 1 | 1 | 1 | 1 | 1 | 1 | 1 | 1 | 1 | 1 | 1 | 1 | 1 | 1 | 1 | 1 | 1 | 1 | 1 | 1 | 1 | 1 | 1 | 1 | 1 | 1 | 1 | 1 | 1 | 1 | 1 | 1 | 1 | 1 | 1 | 1 | 1 | 1 | 1 | 1 | 1 | 1 | 1 | 1 | 1 | 1 | 1 | 1 | 1 | 1 | 1 | 1 | 1 | 1 | 1 | 1 | 1 | 1 | 1 | 1 | 1 | 1 | 1 | 1 | 1 | 1 | 1 | 1 | 1 | 1 | 1 | 1 | 1 | 1 | 1 | 1 | 1 | 1 | 1 | 1 | 1 | 1 | 1 | 1 | 1 | 1 | 1 | 1 | 1 | 1 | 1 | 1 | 1 | 1 | 1 | 1 | 1 | 1 | 1 | 1 | 1 | 1 | 1 | 1 | 1 | 1 | 1 | 1 | 1 | 1 | 1 | 1 | 1 | 1 | 1 | 1 | 1 | 1 | 1 | 1 | 1 | 1 | 1 | 1 | 1 | 1 | 1 | 1 | 1 | 1 | 1 | 1 | 1 | 1 | 1 | 1 | 1 | 1 | 1 | 1 | 1 | 1 | 1 | 1 | 1 | 1 | 1 | 1 | 1 | 1 | 1 | 1 | 1 | 1 | 1 | 1 | 1 | 1 | 1 | 1 | 1 | 1 | 1 | 1 | 1 | 1 | 1 | 1 | 1 | 1 | 1 | 1 | 1 | 1 | 1 | 1 | 1 | 1 | 1 | 1 | 1 | 1 | 1 | 1 | 1 | 1 | 1 | 1 | 1 | 1 | 1 | 1 | 1 | 1 | 1 | 1 | 1 | 1 | 1 | 1 | 1 | 1 | 1 | 1 | 1 | 1 | 1 | 1 | 1 | 1 | 1 | 1 | 1 | 1 | 1 | 1 | 1 | 1 | 1 | 1 | 1 | 1 | 1 | 1 | 1 | 1 | 1 | 1 | 1 | 1 | 1 | 1 | 1 | 1 | 1 | 1 | 1 | 1 | 1 | 1 | 1 | 1 | 1 | 1 | 1 | 1 | 1 | 1 | 1 | 1 | 1 | 1 | 1 | 1 | 1 | 1 | 1 | 1 | 1 | 1 | 1 | 1 | 1 | 1 | 1 | 1 | 1 | 1 | 1 | 1 | 1 | 1 | 1 | 1 | 1 | 1 | 1 | 1 | 1 | 1 | 1 | 1 | 1 | 1 | 1 | 1 | 1 | 1 | 1 | 1 | 1 | 1 | 1 | 1 | 1 | 1 | 1 | 1 | 1 | 1 | 1 | 1 | 1 | 1 | 1 | 1 | 1 | 1 | 1 | 1 | 1 | 1 | 1 | 1 | 1 | 1 | 1 | 1 | 1 | 1 | 1 | 1 | 1 | 1 | 1 | 1 | 1 | 1 | 1 | 1 | 1 | 1 | 1 | 1 | 1 | 1 | 1 | 1 | 1 | 1 | 1 | 1 | 1 | 1 | 1 | 1 | 1 | 1 | 1 | 1 | 1 | 1 | 1 | 1 | 1 | 1 | 1 | 1 | 1 | 1 | 1 | 1 | 1 | 1 | 1 | 1 | 1 | 1 | 1 | 1 | 1 | 1 | 1 | 1 | 1 | 1 | 1 | 1 | 1 | 1 | 1 | 1 | 1 | 1 | 1 | 1 | 1 | 1 | 1 | 1 | 1 | 1 | 1 | 1 | 1 | 1 | 1 | 1 | 1 | 1 | 1 | 1 | 1 | 1 | 1 | 1 | 1 | 1 | 1 | 1 | 1 | 1 | 1 | 1 | 1 | 1 | 1 | 1 | 1 | 1 | 1 | 1 | 1 | 1 | 1 | 1 | 1 | 1 | 1 | 1 | 1 | 1 | 1 | 1 | 1 | 1 | 1 | 1 | 1 | 1 | 1 | 1 | 1 |
| Nothus lunus\* | 1 | 1 | 1 | 1 | 1 | 1 | 1 | 1 | 1 | 1 | 1 | 1 | 1 | 1 | 1 | 1 | 1 | 1 | 1 | 1 | 1 | 1 | 1 | 1 | 1 | 1 | 1 | 1 | 1 | 1 | 1 | 1 | 1 | 1 | 1 | 1 | 1 | 1 | 1 | 1 | 1 | 1 | 1 | 1 | 1 | 1 | 1 | 1 | 1 | 1 | 1 | 1 | 1 | 1 | 1 | 1 | 1 | 1 | 1 | 1 | 1 | 1 | 1 | 1 | 1 | 1 | 1 | 1 | 1 | 1 | 1 | 1 | 1 | 1 | 1 | 1 | 1 | 1 | 1 | 1 | 1 | 1 | 1 | 1 | 1 | 1 | 1 | 1 | 1 | 1 | 1 | 1 | 1 | 1 | 1 | 1 | 1 | 1 | 1 | 1 | 1 | 1 | 1 | 1 | 1 | 1 | 1 | 1 | 1 | 1 | 1 | 1 | 1 | 1 | 1 | 1 | 1 | 1 | 1 | 1 | 1 | 1 | 1 | 1 | 1 | 1 | 1 | 1 | 1 | 1 | 1 | 1 | 1 | 1 | 1 | 1 | 1 | 1 | 1 | 1 | 1 | 1 | 1 | 1 | 1 | 1 | 1 | 1 | 1 | 1 | 1 | 1 | 1 | 1 | 1 | 1 | 1 | 1 | 1 | 1 | 1 | 1 | 1 | 1 | 1 | 1 | 1 | 1 | 1 | 1 | 1 | 1 | 1 | 1 | 1 | 1 | 1 | 1 | 1 | 1 | 1 | 1 | 1 | 1 | 1 | 1 | 1 | 1 | 1 | 1 | 1 | 1 | 1 | 1 | 1 | 1 | 1 | 1 | 1 | 1 | 1 | 1 | 1 | 1 | 1 | 1 | 1 | 1 | 1 | 1 | 1 | 1 | 1 | 1 | 1 | 1 | 1 | 1 | 1 | 1 | 1 | 1 | 1 | 1 | 1 | 1 | 1 | 1 | 1 | 1 | 1 | 1 | 1 | 1 | 1 | 1 | 1 | 1 | 1 | 1 | 1 | 1 | 1 | 1 | 1 | 1 | 1 | 1 | 1 | 1 | 1 | 1 | 1 | 1 | 1 | 1 | 1 | 1 | 1 | 1 | 1 | 1 | 1 | 1 | 1 | 1 | 1 | 1 | 1 | 1 | 1 | 1 | 1 | 1 | 1 | 1 | 1 | 1 | 1 | 1 | 1 | 1 | 1 | 1 | 1 | 1 | 1 | 1 | 1 | 1 | 1 | 1 | 1 | 1 | 1 | 1 | 1 | 1 | 1 | 1 | 1 | 1 | 1 | 1 | 1 | 1 | 1 | 1 | 1 | 1 | 1 | 1 | 1 | 1 | 1 | 1 | 1 | 1 | 1 | 1 | 1 | 1 | 1 | 1 | 1 | 1 | 1 | 1 | 1 | 1 | 1 | 1 | 1 | 1 | 1 | 1 | 1 | 1 | 1 | 1 | 1 | 1 | 1 | 1 | 1 | 1 | 1 | 1 | 1 | 1 | 1 | 1 | 1 | 1 | 1 | 1 | 1 | 1 | 1 | 1 | 1 | 1 | 1 | 1 | 1 | 1 | 1 | 1 | 1 | 1 | 1 | 1 | 1 | 1 | 1 | 1 | 1 | 1 | 1 | 1 | 1 | 1 | 1 | 1 | 1 | 1 | 1 | 1 | 1 | 1 | 1 | 1 | 1 | 1 | 1 | 1 | 1 | 1 | 1 | 1 | 1 | 1 | 1 | 1 | 1 | 1 | 1 | 1 | 1 | 1 | 1 | 1 | 1 | 1 | 1 | 1 | 1 | 1 | 1 | 1 | 1 | 1 | 1 | 1 | 1 | 1 | 1 | 1 | 1 | 1 | 1 | 1 | 1 | 1 | 1 | 1 | 1 | 1 | 1 | 1 | 1 | 1 | 1 | 1 | 1 | 1 | 1 | 1 | 1 | 1 | 1 | 1 | 1 | 1 | 1 | 1 | 1 | 1 | 1 | 1 | 1 | 1 | 1 | 1 | 1 |
| Papilio glaucus | 1 | 1 | 1 | 1 | 1 | 1 | 1 | 1 | 1 | 1 | 1 | 1 | 1 | 1 | 1 | 1 | 1 | 1 | 1 | 1 | 1 | 1 | 1 | 1 | 1 | 1 | 1 | 1 | 1 | 1 | 1 | 1 | 1 | 1 | 1 | 1 | 1 | 1 | 1 | 1 | 1 | 1 | 1 | 1 | 1 | 1 | 1 | 1 | 1 | 1 | 1 | 1 | 1 | 1 | 1 | 1 | 1 | 1 | 1 | 1 | 1 | 1 | 1 | 1 | 1 | 1 | 1 | 1 | 1 | 1 | 1 | 1 | 1 | 1 | 1 | 1 | 1 | 1 | 1 | 1 | 1 | 1 | 1 | 1 | 1 | 1 | 1 | 1 | 1 | 1 | 1 | 1 | 1 | 1 | 1 | 1 | 1 | 1 | 1 | 1 | 1 | 1 | 1 | 1 | 1 | 1 | 1 | 1 | 1 | 1 | 1 | 1 | 1 | 1 | 1 | 1 | 1 | 1 | 1 | 1 | 1 | 1 | 1 | 1 | 1 | 1 | 1 | 1 | 1 | 1 | 1 | 1 | 1 | 1 | 1 | 1 | 1 | 1 | 1 | 1 | 1 | 1 | 1 | 1 | 1 | 1 | 1 | 1 | 1 | 1 | 1 | 1 | 1 | 1 | 1 | 1 | 1 | 1 | 1 | 1 | 1 | 1 | 1 | 1 | 1 | 1 | 1 | 1 | 1 | 1 | 1 | 1 | 1 | 1 | 1 | 1 | 1 | 1 | 1 | 1 | 1 | 1 | 1 | 1 | 1 | 1 | 1 | 1 | 1 | 1 | 1 | 1 | 1 | 1 | 1 | 1 | 1 | 1 | 1 | 1 | 1 | 1 | 1 | 1 | 1 | 1 | 1 | 1 | 1 | 1 | 1 | 1 | 1 | 1 | 1 | 1 | 1 | 1 | 1 | 1 | 1 | 1 | 1 | 1 | 1 | 1 | 1 | 1 | 1 | 1 | 1 | 1 | 1 | 1 | 1 | 1 | 1 | 1 | 1 | 1 | 1 | 1 | 1 | 1 | 1 | 1 | 1 | 1 | 1 | 1 | 1 | 1 | 1 | 1 | 1 | 1 | 1 | 1 | 1 | 1 | 1 | 1 | 1 | 1 | 1 | 1 | 1 | 1 | 1 | 1 | 1 | 1 | 1 | 1 | 1 | 1 | 1 | 1 | 1 | 1 | 1 | 1 | 1 | 1 | 1 | 1 | 1 | 1 | 1 | 1 | 1 | 1 | 1 | 1 | 1 | 1 | 1 | 1 | 1 | 1 | 1 | 1 | 1 | 1 | 1 | 1 | 1 | 1 | 1 | 1 | 1 | 1 | 1 | 1 | 1 | 1 | 1 | 1 | 1 | 1 | 1 | 1 | 1 | 1 | 1 | 1 | 1 | 1 | 1 | 1 | 1 | 1 | 1 | 1 | 1 | 1 | 1 | 1 | 1 | 1 | 1 | 1 | 1 | 1 | 1 | 1 | 1 | 1 | 1 | 1 | 1 | 1 | 1 | 1 | 1 | 1 | 1 | 1 | 1 | 1 | 1 | 1 | 1 | 1 | 1 | 1 | 1 | 1 | 1 | 1 | 1 | 1 | 1 | 1 | 1 | 1 | 1 | 1 | 1 | 1 | 1 | 1 | 1 | 1 | 1 | 1 | 1 | 1 | 1 | 1 | 1 | 1 | 1 | 1 | 1 | 1 | 1 | 1 | 1 | 1 | 1 | 1 | 1 | 1 | 1 | 1 | 1 | 1 | 1 | 1 | 1 | 1 | 1 | 1 | 1 | 1 | 1 | 1 | 1 | 1 | 1 | 1 | 1 | 1 | 1 | 1 | 1 | 1 | 1 | 1 | 1 | 1 | 1 | 1 | 1 | 1 | 1 | 1 | 1 | 1 | 1 | 1 | 1 | 1 | 1 | 1 | 1 | 1 | 1 | 1 | 1 | 1 | 1 | 1 | 1 | 1 | 1 | 1 | 1 | 1 | 1 | 1 | 1 | 1 | 1 |
| Phyllocnistis citrella\* | 1 | 1 | 1 | 1 | 1 | 1 | 1 | 1 | 1 | 1 | 1 | 1 | 1 | 1 | 1 | 1 | 1 | 1 | 1 | 1 | 1 | 1 | 1 | 1 | 1 | 1 | 1 | 1 | 1 | 1 | 1 | 1 | 1 | 1 | 1 | 1 | 1 | 1 | 1 | 1 | 1 | 1 | 1 | 1 | 1 | 1 | 1 | 1 | 1 | 1 | 1 | 1 | 1 | 1 | 1 | 1 | 1 | 1 | 1 | 1 | 1 | 1 | 1 | 1 | 1 | 1 | 1 | 1 | 1 | 1 | 1 | 1 | 1 | 1 | 1 | 1 | 1 | 1 | 1 | 1 | 1 | 1 | 1 | 1 | 1 | 1 | 1 | 1 | 1 | 1 | 1 | 1 | 1 | 1 | 1 | 1 | 1 | 1 | 1 | 1 | 1 | 1 | 1 | 1 | 1 | 1 | 1 | 1 | 1 | 1 | 1 | 1 | 1 | 1 | 1 | 1 | 1 | 1 | 1 | 1 | 1 | 1 | 1 | 1 | 1 | 1 | 1 | 1 | 1 | 1 | 1 | 1 | 1 | 1 | 1 | 1 | 1 | 1 | 1 | 1 | 1 | 1 | 1 | 1 | 1 | 1 | 1 | 1 | 1 | 1 | 1 | 1 | 1 | 1 | 1 | 1 | 1 | 1 | 1 | 1 | 1 | 1 | 1 | 1 | 1 | 1 | 1 | 1 | 1 | 1 | 1 | 1 | 1 | 1 | 1 | 1 | 1 | 1 | 1 | 1 | 1 | 1 | 1 | 1 | 1 | 1 | 1 | 1 | 1 | 1 | 1 | 1 | 1 | 1 | 1 | 1 | 1 | 1 | 1 | 1 | 1 | 1 | 1 | 1 | 1 | 1 | 1 | 1 | 1 | 1 | 1 | 1 | 1 | 1 | 1 | 1 | 1 | 1 | 1 | 1 | 1 | 1 | 1 | 1 | 1 | 1 | 1 | 1 | 1 | 1 | 1 | 1 | 1 | 1 | 1 | 1 | 1 | 1 | 1 | 1 | 1 | 1 | 1 | 1 | 1 | 1 | 1 | 1 | 1 | 1 | 1 | 1 | 1 | 1 | 1 | 1 | 1 | 1 | 1 | 1 | 1 | 1 | 1 | 1 | 1 | 1 | 1 | 1 | 1 | 1 | 1 | 1 | 1 | 1 | 1 | 1 | 1 | 1 | 1 | 1 | 1 | 1 | 1 | 1 | 1 | 1 | 1 | 1 | 1 | 1 | 1 | 1 | 1 | 1 | 1 | 1 | 1 | 1 | 1 | 1 | 1 | 1 | 1 | 1 | 1 | 1 | 1 | 1 | 1 | 1 | 1 | 1 | 1 | 1 | 1 | 1 | 1 | 1 | 1 | 1 | 1 | 1 | 1 | 1 | 1 | 1 | 1 | 1 | 1 | 1 | 1 | 1 | 1 | 1 | 1 | 1 | 1 | 1 | 1 | 1 | 1 | 1 | 1 | 1 | 1 | 1 | 1 | 1 | 1 | 1 | 1 | 1 | 1 | 1 | 1 | 1 | 1 | 1 | 1 | 1 | 1 | 1 | 1 | 1 | 1 | 1 | 1 | 1 | 1 | 1 | 1 | 1 | 1 | 1 | 1 | 1 | 1 | 1 | 1 | 1 | 1 | 1 | 1 | 1 | 1 | 1 | 1 | 1 | 1 | 1 | 1 | 1 | 1 | 1 | 1 | 1 | 1 | 1 | 1 | 1 | 1 | 1 | 1 | 1 | 1 | 1 | 1 | 1 | 1 | 1 | 1 | 1 | 1 | 1 | 1 | 1 | 1 | 1 | 1 | 1 | 1 | 1 | 1 | 1 | 1 | 1 | 1 | 1 | 1 | 1 | 1 | 1 | 1 | 1 | 1 | 1 | 1 | 1 | 1 | 1 | 1 | 1 | 1 | 1 | 1 | 1 | 1 | 1 | 1 | 1 | 1 | 1 | 1 | 1 | 1 | 1 | 1 | 1 | 1 | 1 | 1 | 1 | 1 | 1 | 1 |
| Plutella xylostella\* | 1 | 1 | 1 | 1 | 1 | 1 | 1 | 1 | 1 | 1 | 1 | 1 | 1 | 1 | 1 | 1 | 1 | 1 | 1 | 1 | 1 | 1 | 1 | 1 | 1 | 1 | 1 | 1 | 1 | 1 | 1 | 1 | 1 | 1 | 1 | 1 | 1 | 1 | 1 | 1 | 1 | 1 | 1 | 1 | 1 | 1 | 1 | 1 | 1 | 1 | 1 | 1 | 1 | 1 | 1 | 1 | 1 | 1 | 1 | 1 | 1 | 1 | 1 | 1 | 1 | 1 | 1 | 1 | 1 | 1 | 1 | 1 | 1 | 1 | 1 | 1 | 1 | 1 | 1 | 1 | 1 | 1 | 1 | 1 | 1 | 1 | 1 | 1 | 1 | 1 | 1 | 1 | 1 | 1 | 1 | 1 | 1 | 1 | 1 | 1 | 1 | 1 | 1 | 1 | 1 | 1 | 1 | 1 | 1 | 1 | 1 | 1 | 1 | 1 | 1 | 1 | 1 | 1 | 1 | 1 | 1 | 1 | 1 | 1 | 1 | 1 | 1 | 1 | 1 | 1 | 1 | 1 | 1 | 1 | 1 | 1 | 1 | 1 | 1 | 1 | 1 | 1 | 1 | 1 | 1 | 1 | 1 | 1 | 1 | 1 | 1 | 1 | 1 | 1 | 1 | 1 | 1 | 1 | 1 | 1 | 1 | 1 | 1 | 1 | 1 | 1 | 1 | 1 | 1 | 1 | 1 | 1 | 1 | 1 | 1 | 1 | 1 | 1 | 1 | 1 | 1 | 1 | 1 | 1 | 1 | 1 | 1 | 1 | 1 | 1 | 1 | 1 | 1 | 1 | 1 | 1 | 1 | 1 | 1 | 1 | 1 | 1 | 1 | 1 | 1 | 1 | 1 | 1 | 1 | 1 | 1 | 1 | 1 | 1 | 1 | 1 | 1 | 1 | 1 | 1 | 1 | 1 | 1 | 1 | 1 | 1 | 1 | 1 | 1 | 1 | 1 | 1 | 1 | 1 | 1 | 1 | 1 | 1 | 1 | 1 | 1 | 1 | 1 | 1 | 1 | 1 | 1 | 1 | 1 | 1 | 1 | 1 | 1 | 1 | 1 | 1 | 1 | 1 | 1 | 1 | 1 | 1 | 1 | 1 | 1 | 1 | 1 | 1 | 1 | 1 | 1 | 1 | 1 | 1 | 1 | 1 | 1 | 1 | 1 | 1 | 1 | 1 | 1 | 1 | 1 | 1 | 1 | 1 | 1 | 1 | 1 | 1 | 1 | 1 | 1 | 1 | 1 | 1 | 1 | 1 | 1 | 1 | 1 | 1 | 1 | 1 | 1 | 1 | 1 | 1 | 1 | 1 | 1 | 1 | 1 | 1 | 1 | 1 | 1 | 1 | 1 | 1 | 1 | 1 | 1 | 1 | 1 | 1 | 1 | 1 | 1 | 1 | 1 | 1 | 1 | 1 | 1 | 1 | 1 | 1 | 1 | 1 | 1 | 1 | 1 | 1 | 1 | 1 | 1 | 1 | 1 | 1 | 1 | 1 | 1 | 1 | 1 | 1 | 1 | 1 | 1 | 1 | 1 | 1 | 1 | 1 | 1 | 1 | 1 | 1 | 1 | 1 | 1 | 1 | 1 | 1 | 1 | 1 | 1 | 1 | 1 | 1 | 1 | 1 | 1 | 1 | 1 | 1 | 1 | 1 | 1 | 1 | 1 | 1 | 1 | 1 | 1 | 1 | 1 | 1 | 1 | 1 | 1 | 1 | 1 | 1 | 1 | 1 | 1 | 1 | 1 | 1 | 1 | 1 | 1 | 1 | 1 | 1 | 1 | 1 | 1 | 1 | 1 | 1 | 1 | 1 | 1 | 1 | 1 | 1 | 1 | 1 | 1 | 1 | 1 | 1 | 1 | 1 | 1 | 1 | 1 | 1 | 1 | 1 | 1 | 1 | 1 | 1 | 1 | 1 | 1 | 1 | 1 | 1 | 1 | 1 | 1 | 1 | 1 | 1 | 1 | 1 | 1 | 1 | 1 |
| Pterodecta felderi\* | 1 | 1 | 1 | 1 | 1 | 1 | 1 | 1 | 1 | 1 | 1 | 1 | 1 | 1 | 1 | 1 | 1 | 1 | 1 | 1 | 1 | 1 | 1 | 1 | 1 | 1 | 1 | 1 | 1 | 1 | 1 | 1 | 1 | 1 | 1 | 1 | 1 | 1 | 1 | 1 | 1 | 1 | 1 | 1 | 1 | 1 | 1 | 1 | 1 | 1 | 1 | 1 | 1 | 1 | 1 | 1 | 1 | 1 | 1 | 1 | 1 | 1 | 1 | 1 | 1 | 1 | 1 | 1 | 1 | 1 | 1 | 1 | 1 | 1 | 1 | 1 | 1 | 1 | 1 | 1 | 1 | 1 | 1 | 1 | 1 | 1 | 1 | 1 | 1 | 1 | 1 | 1 | 1 | 1 | 1 | 1 | 1 | 1 | 1 | 1 | 1 | 1 | 1 | 1 | 1 | 1 | 1 | 1 | 1 | 1 | 1 | 1 | 1 | 1 | 1 | 1 | 1 | 1 | 1 | 1 | 1 | 1 | 1 | 1 | 1 | 1 | 1 | 1 | 1 | 1 | 1 | 1 | 1 | 1 | 1 | 1 | 1 | 1 | 1 | 1 | 1 | 1 | 1 | 1 | 1 | 1 | 1 | 1 | 1 | 1 | 1 | 1 | 1 | 1 | 1 | 1 | 1 | 1 | 1 | 1 | 1 | 1 | 1 | 1 | 1 | 1 | 1 | 1 | 1 | 1 | 1 | 1 | 1 | 1 | 1 | 1 | 1 | 1 | 1 | 1 | 1 | 1 | 1 | 1 | 1 | 1 | 1 | 1 | 1 | 1 | 1 | 1 | 1 | 1 | 1 | 1 | 1 | 1 | 1 | 1 | 1 | 1 | 1 | 1 | 1 | 1 | 1 | 1 | 1 | 1 | 1 | 1 | 1 | 1 | 1 | 1 | 1 | 1 | 1 | 1 | 1 | 1 | 1 | 1 | 1 | 1 | 1 | 1 | 1 | 1 | 1 | 1 | 1 | 1 | 1 | 1 | 1 | 1 | 1 | 1 | 1 | 1 | 1 | 1 | 1 | 1 | 1 | 1 | 1 | 1 | 1 | 1 | 1 | 1 | 1 | 1 | 1 | 1 | 1 | 1 | 1 | 1 | 1 | 1 | 1 | 1 | 1 | 1 | 1 | 1 | 1 | 1 | 1 | 1 | 1 | 1 | 1 | 1 | 1 | 1 | 1 | 1 | 1 | 1 | 1 | 1 | 1 | 1 | 1 | 1 | 1 | 1 | 1 | 1 | 1 | 1 | 1 | 1 | 1 | 1 | 1 | 1 | 1 | 1 | 1 | 1 | 1 | 1 | 1 | 1 | 1 | 1 | 1 | 1 | 1 | 1 | 1 | 1 | 1 | 1 | 1 | 1 | 1 | 1 | 1 | 1 | 1 | 1 | 1 | 1 | 1 | 1 | 1 | 1 | 1 | 1 | 1 | 1 | 1 | 1 | 1 | 1 | 1 | 1 | 1 | 1 | 1 | 1 | 1 | 1 | 1 | 1 | 1 | 1 | 1 | 1 | 1 | 1 | 1 | 1 | 1 | 1 | 1 | 1 | 1 | 1 | 1 | 1 | 1 | 1 | 1 | 1 | 1 | 1 | 1 | 1 | 1 | 1 | 1 | 1 | 1 | 1 | 1 | 1 | 1 | 1 | 1 | 1 | 1 | 1 | 1 | 1 | 1 | 1 | 1 | 1 | 1 | 1 | 1 | 1 | 1 | 1 | 1 | 1 | 1 | 1 | 1 | 1 | 1 | 1 | 1 | 1 | 1 | 1 | 1 | 1 | 1 | 1 | 1 | 1 | 1 | 1 | 1 | 1 | 1 | 1 | 1 | 1 | 1 | 1 | 1 | 1 | 1 | 1 | 1 | 1 | 1 | 1 | 1 | 1 | 1 | 1 | 1 | 1 | 1 | 1 | 1 | 1 | 1 | 1 | 1 | 1 | 1 | 1 | 1 | 1 | 1 | 1 | 1 | 1 | 1 | 1 | 1 | 1 | 1 |
| Semomesia campanea | 1 | 1 | 1 | 1 | 1 | 1 | 1 | 1 | 1 | 1 | 1 | 1 | 1 | 1 | 1 | 1 | 1 | 1 | 1 | 1 | 1 | 1 | 1 | 1 | 1 | 1 | 1 | 1 | 1 | 1 | 1 | 1 | 1 | 1 | 1 | 1 | 1 | 1 | 1 | 1 | 1 | 1 | 1 | 1 | 1 | 1 | 1 | 1 | 1 | 1 | 1 | 1 | 1 | 1 | 1 | 1 | 1 | 1 | 1 | 1 | 1 | 1 | 1 | 1 | 1 | 1 | 1 | 1 | 1 | 1 | 1 | 1 | 1 | 1 | 1 | 1 | 1 | 1 | 1 | 1 | 1 | 1 | 1 | 1 | 1 | 1 | 1 | 1 | 1 | 1 | 1 | 1 | 1 | 1 | 1 | 1 | 1 | 1 | 1 | 1 | 1 | 1 | 1 | 1 | 1 | 1 | 1 | 1 | 1 | 1 | 1 | 1 | 1 | 1 | 1 | 1 | 1 | 1 | 1 | 1 | 1 | 1 | 1 | 1 | 1 | 1 | 1 | 1 | 1 | 1 | 1 | 1 | 1 | 1 | 1 | 1 | 1 | 1 | 1 | 1 | 1 | 1 | 1 | 1 | 1 | 1 | 1 | 1 | 1 | 1 | 1 | 1 | 1 | 1 | 1 | 1 | 1 | 1 | 1 | 1 | 1 | 1 | 1 | 1 | 1 | 1 | 1 | 1 | 1 | 1 | 1 | 1 | 1 | 1 | 1 | 1 | 1 | 1 | 1 | 1 | 1 | 1 | 1 | 1 | 1 | 1 | 1 | 1 | 1 | 1 | 1 | 1 | 1 | 1 | 1 | 1 | 1 | 1 | 1 | 1 | 1 | 1 | 1 | 1 | 1 | 1 | 1 | 1 | 1 | 1 | 1 | 1 | 1 | 1 | 1 | 1 | 1 | 1 | 1 | 1 | 1 | 1 | 1 | 1 | 1 | 1 | 1 | 1 | 1 | 1 | 1 | 1 | 1 | 1 | 1 | 1 | 1 | 1 | 1 | 1 | 1 | 1 | 1 | 1 | 1 | 1 | 1 | 1 | 1 | 1 | 1 | 1 | 1 | 1 | 1 | 1 | 1 | 1 | 1 | 1 | 1 | 1 | 1 | 1 | 1 | 1 | 1 | 1 | 1 | 1 | 1 | 1 | 1 | 1 | 1 | 1 | 1 | 1 | 1 | 1 | 1 | 1 | 1 | 1 | 1 | 1 | 1 | 1 | 1 | 1 | 1 | 1 | 1 | 1 | 1 | 1 | 1 | 1 | 1 | 1 | 1 | 1 | 1 | 1 | 1 | 1 | 1 | 1 | 1 | 1 | 1 | 1 | 1 | 1 | 1 | 1 | 1 | 1 | 1 | 1 | 1 | 1 | 1 | 1 | 1 | 1 | 1 | 1 | 1 | 1 | 1 | 1 | 1 | 1 | 1 | 1 | 1 | 1 | 1 | 1 | 1 | 1 | 1 | 1 | 1 | 1 | 1 | 1 | 1 | 1 | 1 | 1 | 1 | 1 | 1 | 1 | 1 | 1 | 1 | 1 | 1 | 1 | 1 | 1 | 1 | 1 | 1 | 1 | 1 | 1 | 1 | 1 | 1 | 1 | 1 | 1 | 1 | 1 | 1 | 1 | 1 | 1 | 1 | 1 | 1 | 1 | 1 | 1 | 1 | 1 | 1 | 1 | 1 | 1 | 1 | 1 | 1 | 1 | 1 | 1 | 1 | 1 | 1 | 1 | 1 | 1 | 1 | 1 | 1 | 1 | 1 | 1 | 1 | 1 | 1 | 1 | 1 | 1 | 1 | 1 | 1 | 1 | 1 | 1 | 1 | 1 | 1 | 1 | 1 | 1 | 1 | 1 | 1 | 1 | 1 | 1 | 1 | 1 | 1 | 1 | 1 | 1 | 1 | 1 | 1 | 1 | 1 | 1 | 1 | 1 | 1 | 1 | 1 | 1 | 1 | 1 | 1 | 1 | 1 | 1 | 1 | 1 | 1 | 1 | 1 |
| Thubana sp.\* | 1 | 1 | 1 | 1 | 1 | 1 | 1 | 1 | 1 | 1 | 1 | 1 | 1 | 1 | 1 | 1 | 1 | 1 | 1 | 1 | 1 | 1 | 1 | 1 | 1 | 1 | 1 | 1 | 1 | 1 | 1 | 1 | 1 | 1 | 1 | 1 | 1 | 1 | 1 | 1 | 1 | 1 | 1 | 1 | 1 | 1 | 1 | 1 | 1 | 1 | 1 | 1 | 1 | 1 | 1 | 1 | 1 | 1 | 1 | 1 | 1 | 1 | 1 | 1 | 1 | 1 | 1 | 1 | 1 | 1 | 1 | 1 | 1 | 1 | 1 | 1 | 1 | 1 | 1 | 1 | 1 | 1 | 1 | 1 | 1 | 1 | 1 | 1 | 1 | 1 | 1 | 1 | 1 | 1 | 1 | 1 | 1 | 1 | 1 | 1 | 1 | 1 | 1 | 1 | 1 | 1 | 1 | 1 | 1 | 1 | 1 | 1 | 1 | 1 | 1 | 1 | 1 | 1 | 1 | 1 | 1 | 1 | 1 | 1 | 1 | 1 | 1 | 1 | 1 | 1 | 1 | 1 | 1 | 1 | 1 | 1 | 1 | 1 | 1 | 1 | 1 | 1 | 1 | 1 | 1 | 1 | 1 | 1 | 1 | 1 | 1 | 1 | 1 | 1 | 1 | 1 | 1 | 1 | 1 | 1 | 1 | 1 | 1 | 1 | 1 | 1 | 1 | 1 | 1 | 1 | 1 | 1 | 1 | 1 | 1 | 1 | 1 | 1 | 1 | 1 | 1 | 1 | 1 | 1 | 1 | 1 | 1 | 1 | 1 | 1 | 1 | 1 | 1 | 1 | 1 | 1 | 1 | 1 | 1 | 1 | 1 | 1 | 1 | 1 | 1 | 1 | 1 | 1 | 1 | 1 | 1 | 1 | 1 | 1 | 1 | 1 | 1 | 1 | 1 | 1 | 1 | 1 | 1 | 1 | 1 | 1 | 1 | 1 | 1 | 1 | 1 | 1 | 1 | 1 | 1 | 1 | 1 | 1 | 1 | 1 | 1 | 1 | 1 | 1 | 1 | 1 | 1 | 1 | 1 | 1 | 1 | 1 | 1 | 1 | 1 | 1 | 1 | 1 | 1 | 1 | 1 | 1 | 1 | 1 | 1 | 1 | 1 | 1 | 1 | 1 | 1 | 1 | 1 | 1 | 1 | 1 | 1 | 1 | 1 | 1 | 1 | 1 | 1 | 1 | 1 | 1 | 1 | 1 | 1 | 1 | 1 | 1 | 1 | 1 | 1 | 1 | 1 | 1 | 1 | 1 | 1 | 1 | 1 | 1 | 1 | 1 | 1 | 1 | 1 | 1 | 1 | 1 | 1 | 1 | 1 | 1 | 1 | 1 | 1 | 1 | 1 | 1 | 1 | 1 | 1 | 1 | 1 | 1 | 1 | 1 | 1 | 1 | 1 | 1 | 1 | 1 | 1 | 1 | 1 | 1 | 1 | 1 | 1 | 1 | 1 | 1 | 1 | 1 | 1 | 1 | 1 | 1 | 1 | 1 | 1 | 1 | 1 | 1 | 1 | 1 | 1 | 1 | 1 | 1 | 1 | 1 | 1 | 1 | 1 | 1 | 1 | 1 | 1 | 1 | 1 | 1 | 1 | 1 | 1 | 1 | 1 | 1 | 1 | 1 | 1 | 1 | 1 | 1 | 1 | 1 | 1 | 1 | 1 | 1 | 1 | 1 | 1 | 1 | 1 | 1 | 1 | 1 | 1 | 1 | 1 | 1 | 1 | 1 | 1 | 1 | 1 | 1 | 1 | 1 | 1 | 1 | 1 | 1 | 1 | 1 | 1 | 1 | 1 | 1 | 1 | 1 | 1 | 1 | 1 | 1 | 1 | 1 | 1 | 1 | 1 | 1 | 1 | 1 | 1 | 1 | 1 | 1 | 1 | 1 | 1 | 1 | 1 | 1 | 1 | 1 | 1 | 1 | 1 | 1 | 1 | 1 | 1 | 1 | 1 | 1 | 1 | 1 | 1 | 1 | 1 |
| Urodus parvula\* | 1 | 1 | 1 | 1 | 1 | 1 | 1 | 1 | 1 | 1 | 1 | 1 | 1 | 1 | 1 | 1 | 1 | 1 | 1 | 1 | 1 | 1 | 1 | 1 | 1 | 1 | 1 | 1 | 1 | 1 | 1 | 1 | 1 | 1 | 1 | 1 | 1 | 1 | 1 | 1 | 1 | 1 | 1 | 1 | 1 | 1 | 1 | 1 | 1 | 1 | 1 | 1 | 1 | 1 | 1 | 1 | 1 | 1 | 1 | 1 | 1 | 1 | 1 | 1 | 1 | 1 | 1 | 1 | 1 | 1 | 1 | 1 | 1 | 1 | 1 | 1 | 1 | 1 | 1 | 1 | 1 | 1 | 1 | 1 | 1 | 1 | 1 | 1 | 1 | 1 | 1 | 1 | 1 | 1 | 1 | 1 | 1 | 1 | 1 | 1 | 1 | 1 | 1 | 1 | 1 | 1 | 1 | 1 | 1 | 1 | 1 | 1 | 1 | 1 | 1 | 1 | 1 | 1 | 1 | 1 | 1 | 1 | 1 | 1 | 1 | 1 | 1 | 1 | 1 | 1 | 1 | 1 | 1 | 1 | 1 | 1 | 1 | 1 | 1 | 1 | 1 | 1 | 1 | 1 | 1 | 1 | 1 | 1 | 1 | 1 | 1 | 1 | 1 | 1 | 1 | 1 | 1 | 1 | 1 | 1 | 1 | 1 | 1 | 1 | 1 | 1 | 1 | 1 | 1 | 1 | 1 | 1 | 1 | 1 | 1 | 1 | 1 | 1 | 1 | 1 | 1 | 1 | 1 | 1 | 1 | 1 | 1 | 1 | 1 | 1 | 1 | 1 | 1 | 1 | 1 | 1 | 1 | 1 | 1 | 1 | 1 | 1 | 1 | 1 | 1 | 1 | 1 | 1 | 1 | 1 | 1 | 1 | 1 | 1 | 1 | 1 | 1 | 1 | 1 | 1 | 1 | 1 | 1 | 1 | 1 | 1 | 1 | 1 | 1 | 1 | 1 | 1 | 1 | 1 | 1 | 1 | 1 | 1 | 1 | 1 | 1 | 1 | 1 | 1 | 1 | 1 | 1 | 1 | 1 | 1 | 1 | 1 | 1 | 1 | 1 | 1 | 1 | 1 | 1 | 1 | 1 | 1 | 1 | 1 | 1 | 1 | 1 | 1 | 1 | 1 | 1 | 1 | 1 | 1 | 1 | 1 | 1 | 1 | 1 | 1 | 1 | 1 | 1 | 1 | 1 | 1 | 1 | 1 | 1 | 1 | 1 | 1 | 1 | 1 | 1 | 1 | 1 | 1 | 1 | 1 | 1 | 1 | 1 | 1 | 1 | 1 | 1 | 1 | 1 | 1 | 1 | 1 | 1 | 1 | 1 | 1 | 1 | 1 | 1 | 1 | 1 | 1 | 1 | 1 | 1 | 1 | 1 | 1 | 1 | 1 | 1 | 1 | 1 | 1 | 1 | 1 | 1 | 1 | 1 | 1 | 1 | 1 | 1 | 1 | 1 | 1 | 1 | 1 | 1 | 1 | 1 | 1 | 1 | 1 | 1 | 1 | 1 | 1 | 1 | 1 | 1 | 1 | 1 | 1 | 1 | 1 | 1 | 1 | 1 | 1 | 1 | 1 | 1 | 1 | 1 | 1 | 1 | 1 | 1 | 1 | 1 | 1 | 1 | 1 | 1 | 1 | 1 | 1 | 1 | 1 | 1 | 1 | 1 | 1 | 1 | 1 | 1 | 1 | 1 | 1 | 1 | 1 | 1 | 1 | 1 | 1 | 1 | 1 | 1 | 1 | 1 | 1 | 1 | 1 | 1 | 1 | 1 | 1 | 1 | 1 | 1 | 1 | 1 | 1 | 1 | 1 | 1 | 1 | 1 | 1 | 1 | 1 | 1 | 1 | 1 | 1 | 1 | 1 | 1 | 1 | 1 | 1 | 1 | 1 | 1 | 1 | 1 | 1 | 1 | 1 | 1 | 1 | 1 | 1 | 1 | 1 | 1 | 1 | 1 | 1 | 1 | 1 | 1 | 1 | 1 |
| Zeuzerodes maculata\* | 1 | 1 | 1 | 1 | 1 | 1 | 1 | 1 | 1 | 1 | 1 | 1 | 1 | 1 | 1 | 1 | 1 | 1 | 1 | 1 | 1 | 1 | 1 | 1 | 1 | 1 | 1 | 1 | 1 | 1 | 1 | 1 | 1 | 1 | 1 | 1 | 1 | 1 | 1 | 1 | 1 | 1 | 1 | 1 | 1 | 1 | 1 | 1 | 1 | 1 | 1 | 1 | 1 | 1 | 1 | 1 | 1 | 1 | 1 | 1 | 1 | 1 | 1 | 1 | 1 | 1 | 1 | 1 | 1 | 1 | 1 | 1 | 1 | 1 | 1 | 1 | 1 | 1 | 1 | 1 | 1 | 1 | 1 | 1 | 1 | 1 | 1 | 1 | 1 | 1 | 1 | 1 | 1 | 1 | 1 | 1 | 1 | 1 | 1 | 1 | 1 | 1 | 1 | 1 | 1 | 1 | 1 | 1 | 1 | 1 | 1 | 1 | 1 | 1 | 1 | 1 | 1 | 1 | 1 | 1 | 1 | 1 | 1 | 1 | 1 | 1 | 1 | 1 | 1 | 1 | 1 | 1 | 1 | 1 | 1 | 1 | 1 | 1 | 1 | 1 | 1 | 1 | 1 | 1 | 1 | 1 | 1 | 1 | 1 | 1 | 1 | 1 | 1 | 1 | 1 | 1 | 1 | 1 | 1 | 1 | 1 | 1 | 1 | 1 | 1 | 1 | 1 | 1 | 1 | 1 | 1 | 1 | 1 | 1 | 1 | 1 | 1 | 1 | 1 | 1 | 1 | 1 | 1 | 1 | 1 | 1 | 1 | 1 | 1 | 1 | 1 | 1 | 1 | 1 | 1 | 1 | 1 | 1 | 1 | 1 | 1 | 1 | 1 | 1 | 1 | 1 | 1 | 1 | 1 | 1 | 1 | 1 | 1 | 1 | 1 | 1 | 1 | 1 | 1 | 1 | 1 | 1 | 1 | 1 | 1 | 1 | 1 | 1 | 1 | 1 | 1 | 1 | 1 | 1 | 1 | 1 | 1 | 1 | 1 | 1 | 1 | 1 | 1 | 1 | 1 | 1 | 1 | 1 | 1 | 1 | 1 | 1 | 1 | 1 | 1 | 1 | 1 | 1 | 1 | 1 | 1 | 1 | 1 | 1 | 1 | 1 | 1 | 1 | 1 | 1 | 1 | 1 | 1 | 1 | 1 | 1 | 1 | 1 | 1 | 1 | 1 | 1 | 1 | 1 | 1 | 1 | 1 | 1 | 1 | 1 | 1 | 1 | 1 | 1 | 1 | 1 | 1 | 1 | 1 | 1 | 1 | 1 | 1 | 1 | 1 | 1 | 1 | 1 | 1 | 1 | 1 | 1 | 1 | 1 | 1 | 1 | 1 | 1 | 1 | 1 | 1 | 1 | 1 | 1 | 1 | 1 | 1 | 1 | 1 | 1 | 1 | 1 | 1 | 1 | 1 | 1 | 1 | 1 | 1 | 1 | 1 | 1 | 1 | 1 | 1 | 1 | 1 | 1 | 1 | 1 | 1 | 1 | 1 | 1 | 1 | 1 | 1 | 1 | 1 | 1 | 1 | 1 | 1 | 1 | 1 | 1 | 1 | 1 | 1 | 1 | 1 | 1 | 1 | 1 | 1 | 1 | 1 | 1 | 1 | 1 | 1 | 1 | 1 | 1 | 1 | 1 | 1 | 1 | 1 | 1 | 1 | 1 | 1 | 1 | 1 | 1 | 1 | 1 | 1 | 1 | 1 | 1 | 1 | 1 | 1 | 1 | 1 | 1 | 1 | 1 | 1 | 1 | 1 | 1 | 1 | 1 | 1 | 1 | 1 | 1 | 1 | 1 | 1 | 1 | 1 | 1 | 1 | 1 | 1 | 1 | 1 | 1 | 1 | 1 | 1 | 1 | 1 | 1 | 1 | 1 | 1 | 1 | 1 | 1 | 1 | 1 | 1 | 1 | 1 | 1 | 1 | 1 | 1 | 1 | 1 | 1 | 1 | 1 | 1 | 1 | 1 | 1 | 1 | 1 | 1 |

  

### Description Of Data Concatenations

  

#### Content Of Concatenation "Entropy\_1.14\_0.00\_Loci\_0\_To\_199"

  
Rules for "entropy\_1.14\_0.00\_loci\_0\_to\_199":  
OTUs must have the loci:   
OTUs must have at least one of the following loci:

```
'EOG6M65NR_1', 'EOG6SN1SH_1', 'EOG654933_1', 'EOG66147B_1', 'EOG680J15_1', 'EOG6S7JTK_1', 'EOG60ZR1Z_1', 'EOG6DBTJG_1', 'EOG69ZXTF_1', 'EOG64QT4T_1', 'EOG6K3M19_1', 'EOG60P4BJ_1', 'EOG6DBTK0_1', 'EOG64MXX7_1', 'EOG6GTKHJ_1', 'EOG6NVZQC_1', 'EOG6MPHV2_1', 'EOG68KRFS_1', 'EOG6GHZSR_1', 'EOG666VQ4_1', 'EOG651DV5_1', 'EOG6JDHBD_1', 'EOG63212K_1', 'EOG68D102_1', 'EOG6CJVMH_1', 'EOG60VVTB_1', 'EOG698V46_1', 'EOG6N8R81_1', 'EOG6FXRCK_1', 'EOG6R2400_1', 'EOG68KRG4_1', 'EOG61C70T_1', 'EOG6SJ5JS_1', 'EOG63212W_1', 'EOG6F7NPJ_1', 'EOG695ZW4_1', 'EOG61NTQM_1', 'EOG6NZTZ3_1', 'EOG6J3WM7_1', 'EOG68SGXM_1', 'EOG6RNBCT_1', 'EOG6M39DN_1', 'EOG6RFKXS_1', 'EOG6RXZ4G_1', 'EOG67SSJJ_1', 'EOG69GKKW_1', 'EOG6NZTXP_1', 'EOG6QFWHZ_1', 'EOG68PMPX_1', 'EOG6RFKXB_1', 'EOG62BXD6_1', 'EOG647FXX_1', 'EOG6B8JHP_1', 'EOG6S1TBX_1', 'EOG6DFPRT_1', 'EOG65HS25_1', 'EOG6FFD5C_1', 'EOG6R506D_1', 'EOG670TNG_1', 'EOG6F4SFZ_1', 'EOG6QVCGP_1', 'EOG65TCRS_1', 'EOG6QRH88_1', 'EOG6M65N6_1', 'EOG61G385_1', 'EOG6QRH81_1', 'EOG6RJG4W_1', 'EOG6K0QSG_1', 'EOG6KKZ6X_1', 'EOG60VVTG_1', 'EOG6D26TG_1', 'EOG6QZ7R6_1', 'EOG679FBS_1', 'EOG61RPZF_1', 'EOG6PC9WG_1', 'EOG60GCVD_1', 'EOG6R506Q_1', 'EOG605S4X_1', 'EOG65TCRN_1', 'EOG608ND4_1', 'EOG6640GX_1', 'EOG6SQX12_1', 'EOG69GKMS_1', 'EOG6D26TV_1', 'EOG605S4Z_1', 'EOG6N04JN_1', 'EOG67WNS9_1', 'EOG64TPCQ_1', 'EOG6NS3GS_1', 'EOG6NP76T_1', 'EOG63JC90_1', 'EOG67H5V2_1', 'EOG6NP77P_1', 'EOG6QFWJH_1', 'EOG6N04K4_1', 'EOG6CJVMQ_1', 'EOG6DZ204_1', 'EOG6C5CPH_1', 'EOG6FFD51_1', 'EOG6NP779_1', 'EOG6933N9_1', 'EOG6JQ427_1', 'EOG6C5CNP_1', 'EOG6FTW4C_1', 'EOG65759X_1', 'EOG6QNN0N_1', 'EOG66WZD1_1', 'EOG6FR0WR_1', 'EOG6P2Q64_1', 'EOG666VQC_1', 'EOG63R3R5_1', 'EOG641QGH_1', 'EOG69S6BG_1', 'EOG64XJM7_1', 'EOG65MN9B_1', 'EOG641QFW_1', 'EOG63R3R9_1', 'EOG6N04J6_1', 'EOG6GMV1P_1', 'EOG6S4PKX_1', 'EOG6NP773_1', 'EOG6DZ201_1', 'EOG6QFWJ6_1', 'EOG6CG0CT_1', 'EOG6HHP6B_1', 'EOG605S4Q_1', 'EOG6R7VF9_1', 'EOG6CZBM5_1', 'EOG6JT092_1', 'EOG6FJ8D6_1', 'EOG6QZ7QX_1', 'EOG6F1X6M_1', 'EOG6SBF2V_1', 'EOG6KSPQ0_1', 'EOG6GMV1H_1', 'EOG63R3RC_1', 'EOG68SGX8_1', 'EOG6BK57S_1', 'EOG60K83N_1', 'EOG6KD6Q9_1', 'EOG68GW6Q_1', 'EOG6Q852G_1', 'EOG676K44_1', 'EOG6FN4NZ_1', 'EOG6S1TC2_1', 'EOG6HT8WD_1', 'EOG63FH1F_1', 'EOG6PRSVP_1', 'EOG6R5069_1', 'EOG6CC453_1', 'EOG6001PC_1', 'EOG676K40_1', 'EOG6N30SN_1', 'EOG67D9M5_1', 'EOG6RBQP0_1', 'EOG6Q2DKD_1', 'EOG6255XM_1', 'EOG64BB5X_1', 'EOG6H72G0_1', 'EOG6PZJB1_1', 'EOG64MXWG_1', 'EOG65QHHP_1', 'EOG698V4R_1', 'EOG615GHV_1', 'EOG6FFD5G_1', 'EOG6NVZQ8_1', 'EOG60K833_1', 'EOG62BXCZ_1', 'EOG6CG0D0_1', 'EOG69KFVF_1', 'EOG615GHP_1', 'EOG683D8Q_1', 'EOG6PRSVM_1', 'EOG64J2NS_1', 'EOG66HGF5_1', 'EOG60P4BZ_1', 'EOG66MBPR_1', 'EOG6DFPSD_1', 'EOG64BB5N_1', 'EOG6DBTJX_1', 'EOG6FXRC9_1', 'EOG6STS87_1', 'EOG6SXNHG_1', 'EOG6255XS_1', 'EOG6GF3JF_1', 'EOG6KSPPS_1', 'EOG637RJP_1', 'EOG6J3WMR_1', 'EOG6B2T20_1', 'EOG6GB79H_1', 'EOG6JDHBG_1', 'EOG676K3Q_1', 'EOG6RV2WT_1', 'EOG6GTKHQ_1', 'EOG6FR0W5_1', 'EOG6CC452_1', 'EOG61G37F_1', 'EOG6GXFR5_1', 'EOG6DV5QK_1', 'EOG6KPTG0_1'
```

  

|  |  | EOG6M65NR\_1 | EOG6SN1SH\_1 | EOG654933\_1 | EOG66147B\_1 | EOG680J15\_1 | EOG6S7JTK\_1 | EOG60ZR1Z\_1 | EOG6DBTJG\_1 | EOG69ZXTF\_1 | EOG64QT4T\_1 | EOG6K3M19\_1 | EOG60P4BJ\_1 | EOG6DBTK0\_1 | EOG64MXX7\_1 | EOG6GTKHJ\_1 | EOG6NVZQC\_1 | EOG6MPHV2\_1 | EOG68KRFS\_1 | EOG6GHZSR\_1 | EOG666VQ4\_1 | EOG651DV5\_1 | EOG6JDHBD\_1 | EOG63212K\_1 | EOG68D102\_1 | EOG6CJVMH\_1 | EOG60VVTB\_1 | EOG698V46\_1 | EOG6N8R81\_1 | EOG6FXRCK\_1 | EOG6R2400\_1 | EOG68KRG4\_1 | EOG61C70T\_1 | EOG6SJ5JS\_1 | EOG63212W\_1 | EOG6F7NPJ\_1 | EOG695ZW4\_1 | EOG61NTQM\_1 | EOG6NZTZ3\_1 | EOG6J3WM7\_1 | EOG68SGXM\_1 | EOG6RNBCT\_1 | EOG6M39DN\_1 | EOG6RFKXS\_1 | EOG6RXZ4G\_1 | EOG67SSJJ\_1 | EOG69GKKW\_1 | EOG6NZTXP\_1 | EOG6QFWHZ\_1 | EOG68PMPX\_1 | EOG6RFKXB\_1 | EOG62BXD6\_1 | EOG647FXX\_1 | EOG6B8JHP\_1 | EOG6S1TBX\_1 | EOG6DFPRT\_1 | EOG65HS25\_1 | EOG6FFD5C\_1 | EOG6R506D\_1 | EOG670TNG\_1 | EOG6F4SFZ\_1 | EOG6QVCGP\_1 | EOG65TCRS\_1 | EOG6QRH88\_1 | EOG6M65N6\_1 | EOG61G385\_1 | EOG6QRH81\_1 | EOG6RJG4W\_1 | EOG6K0QSG\_1 | EOG6KKZ6X\_1 | EOG60VVTG\_1 | EOG6D26TG\_1 | EOG6QZ7R6\_1 | EOG679FBS\_1 | EOG61RPZF\_1 | EOG6PC9WG\_1 | EOG60GCVD\_1 | EOG6R506Q\_1 | EOG605S4X\_1 | EOG65TCRN\_1 | EOG608ND4\_1 | EOG6640GX\_1 | EOG6SQX12\_1 | EOG69GKMS\_1 | EOG6D26TV\_1 | EOG605S4Z\_1 | EOG6N04JN\_1 | EOG67WNS9\_1 | EOG64TPCQ\_1 | EOG6NS3GS\_1 | EOG6NP76T\_1 | EOG63JC90\_1 | EOG67H5V2\_1 | EOG6NP77P\_1 | EOG6QFWJH\_1 | EOG6N04K4\_1 | EOG6CJVMQ\_1 | EOG6DZ204\_1 | EOG6C5CPH\_1 | EOG6FFD51\_1 | EOG6NP779\_1 | EOG6933N9\_1 | EOG6JQ427\_1 | EOG6C5CNP\_1 | EOG6FTW4C\_1 | EOG65759X\_1 | EOG6QNN0N\_1 | EOG66WZD1\_1 | EOG6FR0WR\_1 | EOG6P2Q64\_1 | EOG666VQC\_1 | EOG63R3R5\_1 | EOG641QGH\_1 | EOG69S6BG\_1 | EOG64XJM7\_1 | EOG65MN9B\_1 | EOG641QFW\_1 | EOG63R3R9\_1 | EOG6N04J6\_1 | EOG6GMV1P\_1 | EOG6S4PKX\_1 | EOG6NP773\_1 | EOG6DZ201\_1 | EOG6QFWJ6\_1 | EOG6CG0CT\_1 | EOG6HHP6B\_1 | EOG605S4Q\_1 | EOG6R7VF9\_1 | EOG6CZBM5\_1 | EOG6JT092\_1 | EOG6FJ8D6\_1 | EOG6QZ7QX\_1 | EOG6F1X6M\_1 | EOG6SBF2V\_1 | EOG6KSPQ0\_1 | EOG6GMV1H\_1 | EOG63R3RC\_1 | EOG68SGX8\_1 | EOG6BK57S\_1 | EOG60K83N\_1 | EOG6KD6Q9\_1 | EOG68GW6Q\_1 | EOG6Q852G\_1 | EOG676K44\_1 | EOG6FN4NZ\_1 | EOG6S1TC2\_1 | EOG6HT8WD\_1 | EOG63FH1F\_1 | EOG6PRSVP\_1 | EOG6R5069\_1 | EOG6CC453\_1 | EOG6001PC\_1 | EOG676K40\_1 | EOG6N30SN\_1 | EOG67D9M5\_1 | EOG6RBQP0\_1 | EOG6Q2DKD\_1 | EOG6255XM\_1 | EOG64BB5X\_1 | EOG6H72G0\_1 | EOG6PZJB1\_1 | EOG64MXWG\_1 | EOG65QHHP\_1 | EOG698V4R\_1 | EOG615GHV\_1 | EOG6FFD5G\_1 | EOG6NVZQ8\_1 | EOG60K833\_1 | EOG62BXCZ\_1 | EOG6CG0D0\_1 | EOG69KFVF\_1 | EOG615GHP\_1 | EOG683D8Q\_1 | EOG6PRSVM\_1 | EOG64J2NS\_1 | EOG66HGF5\_1 | EOG60P4BZ\_1 | EOG66MBPR\_1 | EOG6DFPSD\_1 | EOG64BB5N\_1 | EOG6DBTJX\_1 | EOG6FXRC9\_1 | EOG6STS87\_1 | EOG6SXNHG\_1 | EOG6255XS\_1 | EOG6GF3JF\_1 | EOG6KSPPS\_1 | EOG637RJP\_1 | EOG6J3WMR\_1 | EOG6B2T20\_1 | EOG6GB79H\_1 | EOG6JDHBG\_1 | EOG676K3Q\_1 | EOG6RV2WT\_1 | EOG6GTKHQ\_1 | EOG6FR0W5\_1 | EOG6CC452\_1 | EOG61G37F\_1 | EOG6GXFR5\_1 | EOG6DV5QK\_1 | EOG6KPTG0\_1 |
| --- | --- | --- | --- | --- | --- | --- | --- | --- | --- | --- | --- | --- | --- | --- | --- | --- | --- | --- | --- | --- | --- | --- | --- | --- | --- | --- | --- | --- | --- | --- | --- | --- | --- | --- | --- | --- | --- | --- | --- | --- | --- | --- | --- | --- | --- | --- | --- | --- | --- | --- | --- | --- | --- | --- | --- | --- | --- | --- | --- | --- | --- | --- | --- | --- | --- | --- | --- | --- | --- | --- | --- | --- | --- | --- | --- | --- | --- | --- | --- | --- | --- | --- | --- | --- | --- | --- | --- | --- | --- | --- | --- | --- | --- | --- | --- | --- | --- | --- | --- | --- | --- | --- | --- | --- | --- | --- | --- | --- | --- | --- | --- | --- | --- | --- | --- | --- | --- | --- | --- | --- | --- | --- | --- | --- | --- | --- | --- | --- | --- | --- | --- | --- | --- | --- | --- | --- | --- | --- | --- | --- | --- | --- | --- | --- | --- | --- | --- | --- | --- | --- | --- | --- | --- | --- | --- | --- | --- | --- | --- | --- | --- | --- | --- | --- | --- | --- | --- | --- | --- | --- | --- | --- | --- | --- | --- | --- | --- | --- | --- | --- | --- | --- | --- | --- | --- | --- | --- | --- | --- | --- | --- | --- | --- | --- | --- | --- | --- | --- | --- | --- | --- |
| Dplexcds | Danaus plexippus\* | denovo28\_f0 | denovo80\_f0 | denovo106\_f0 | denovo132\_f0 | denovo210\_f0 | denovo236\_f0 | denovo262\_f0 | denovo288\_f0 | denovo340\_f0 | denovo418\_f0 | denovo522\_f0 | denovo574\_f0 | denovo600\_f0 | denovo730\_f0 | denovo782\_f0 | denovo938\_f0 | denovo964\_f0 | denovo1042\_f0 | denovo1068\_f0 | denovo1146\_f0 | denovo1302\_f0 | denovo1484\_f0 | denovo1510\_f0 | denovo1640\_f0 | denovo1744\_f0 | denovo1770\_f0 | denovo1796\_f0 | denovo1952\_f0 | denovo2134\_f0 | denovo2186\_f0 | denovo2238\_f0 | denovo2264\_f0 | denovo2342\_f0 | denovo2446\_f0 | denovo2472\_f0 | denovo2498\_f0 | denovo2524\_f0 | denovo2576\_f0 | denovo2602\_f0 | denovo2654\_f0 | denovo2758\_f0 | denovo2784\_f0 | denovo2836\_f0 | denovo2862\_f0 | denovo2914\_f0 | denovo2992\_f0 | denovo3044\_f0 | denovo3148\_f0 | denovo3226\_f0 | denovo3252\_f0 | denovo3278\_f0 | denovo3304\_f0 | denovo3330\_f0 | denovo3434\_f0 | denovo3486\_f0 | denovo3590\_f0 | denovo3616\_f0 | denovo3668\_f0 | denovo3694\_f0 | denovo3720\_f0 | denovo3772\_f0 | denovo3850\_f0 | denovo3954\_f0 | denovo3980\_f0 | denovo4032\_f0 | denovo4058\_f0 | denovo4188\_f0 | denovo4292\_f0 | denovo4318\_f0 | denovo4344\_f0 | denovo4396\_f0 | denovo4448\_f0 | denovo4552\_f0 | denovo4630\_f0 | denovo4734\_f0 | denovo4760\_f0 | denovo4786\_f0 | denovo4838\_f0 | denovo4890\_f0 | denovo4916\_f0 | denovo4942\_f0 | denovo4968\_f0 | denovo4994\_f0 | denovo5202\_f0 | denovo5228\_f0 | denovo5254\_f0 | denovo5384\_f0 | denovo5436\_f0 | denovo5514\_f0 | denovo5592\_f0 | denovo5618\_f0 | denovo5644\_f0 | denovo5930\_f0 | denovo6008\_f0 | denovo6060\_f0 | denovo6112\_f0 | denovo6216\_f0 | denovo6242\_f0 | denovo6268\_f0 | denovo6580\_f0 | denovo6606\_f0 | denovo6684\_f0 | denovo6710\_f0 | denovo6762\_f0 | denovo6814\_f0 | denovo6866\_f0 | denovo6996\_f0 | denovo7074\_f0 | denovo7126\_f0 | denovo7152\_f0 | denovo7256\_f0 | denovo7334\_f0 | denovo7386\_f0 | denovo7412\_f0 | denovo7516\_f0 | denovo7542\_f0 | denovo7568\_f0 | denovo7620\_f0 | denovo7672\_f0 | denovo7698\_f0 | denovo7750\_f0 | denovo7802\_f0 | denovo7984\_f0 | denovo8036\_f0 | denovo8062\_f0 | denovo8218\_f0 | denovo8244\_f0 | denovo8270\_f0 | denovo8296\_f0 | denovo8400\_f0 | denovo8426\_f0 | denovo8452\_f0 | denovo8582\_f0 | denovo8634\_f0 | denovo8660\_f0 | denovo8686\_f0 | denovo8764\_f0 | denovo8816\_f0 | denovo8842\_f0 | denovo8894\_f0 | denovo8920\_f0 | denovo8946\_f0 | denovo8972\_f0 | denovo8998\_f0 | denovo9128\_f0 | denovo9154\_f0 | denovo9180\_f0 | denovo9310\_f0 | denovo9336\_f0 | denovo9362\_f0 | denovo9388\_f0 | denovo9440\_f0 | denovo9492\_f0 | denovo9544\_f0 | denovo9596\_f0 | denovo9674\_f0 | denovo9700\_f0 | denovo9726\_f0 | denovo9804\_f0 | denovo9830\_f0 | denovo9856\_f0 | denovo9882\_f0 | denovo9934\_f0 | denovo9960\_f0 | denovo9986\_f0 | denovo10064\_f0 | denovo10168\_f0 | denovo10194\_f0 | denovo10220\_f0 | denovo10272\_f0 | denovo10298\_f0 | denovo10350\_f0 | denovo10376\_f0 | denovo10402\_f0 | denovo10428\_f0 | denovo10480\_f0 | denovo10506\_f0 | denovo10532\_f0 | denovo10610\_f0 | denovo10662\_f0 | denovo10688\_f0 | denovo10766\_f0 | denovo10844\_f0 | denovo10870\_f0 | denovo10922\_f0 | denovo10974\_f0 | denovo11026\_f0 | denovo11078\_f0 | denovo11104\_f0 | denovo11156\_f0 | denovo11182\_f0 | denovo11234\_f0 | denovo11260\_f0 | denovo11312\_f0 | denovo11338\_f0 | denovo11442\_f0 | denovo11546\_f0 | denovo11910\_f0 | denovo11962\_f0 | denovo12066\_f0 |
| FG120077 | Semomesia campanea | denovo36\_f0 | denovo88\_f0 | denovo114\_f0 | denovo140\_f0 | denovo218\_f0 | denovo244\_f0 | denovo270\_f0 | denovo296\_f0 | denovo348\_f0 | denovo426\_f0 | denovo530\_f0 | denovo582\_f0 | denovo608\_f0 | denovo738\_f0 | denovo790\_f0 | denovo946\_f0 | denovo972\_f0 | denovo1050\_f0 | denovo1076\_f0 | denovo1154\_f0 | denovo1310\_f0 | denovo1492\_f0 | denovo1518\_f0 | denovo1648\_f0 | denovo1752\_f0 | denovo1778\_f0 | denovo1804\_f0 | denovo1960\_f0 | denovo2142\_f0 | denovo2194\_f0 | denovo2246\_f0 | denovo2272\_f0 | denovo2350\_f0 | denovo2454\_f0 | denovo2480\_f0 | denovo2506\_f0 | denovo2532\_f0 | denovo2584\_f0 | denovo2610\_f0 | denovo2662\_f0 | denovo2766\_f0 | denovo2792\_f0 | denovo2844\_f0 | denovo2870\_f0 | denovo2922\_f0 | denovo3000\_f0 | denovo3052\_f0 | denovo3156\_f0 | denovo3234\_f0 | denovo3260\_f0 | denovo3286\_f0 | denovo3312\_f0 | denovo3338\_f0 | denovo3442\_f0 | denovo3494\_f0 | denovo3598\_f0 | denovo3624\_f0 | denovo3676\_f0 | denovo3702\_f0 | denovo3728\_f0 | denovo3780\_f0 | denovo3858\_f0 | denovo3962\_f0 | denovo3988\_f0 | denovo4040\_f0 | denovo4066\_f0 | denovo4196\_f0 | denovo4300\_f0 | denovo4326\_f0 | denovo4352\_f0 | denovo4404\_f0 | denovo4456\_f0 | denovo4560\_f0 | denovo4638\_f0 | denovo4742\_f0 | denovo4768\_f0 | denovo4794\_f0 | denovo4846\_f0 | denovo4898\_f0 | denovo4924\_f0 | denovo4950\_f0 | denovo4976\_f0 | denovo5002\_f0 | denovo5210\_f0 | denovo5236\_f0 | denovo5262\_f0 | denovo5392\_f0 | denovo5444\_f0 | denovo5522\_f0 | denovo5600\_f0 | denovo5626\_f0 | denovo5652\_f0 | denovo5938\_f0 | denovo6016\_f0 | denovo6068\_f0 | denovo6120\_f0 | denovo6224\_f0 | denovo6250\_f0 | denovo6276\_f0 | denovo6588\_f0 | denovo6614\_f0 | denovo6692\_f0 | denovo6718\_f0 | denovo6770\_f0 | denovo6822\_f0 | denovo6874\_f0 | denovo7004\_f0 | denovo7082\_f0 | denovo7134\_f0 | denovo7160\_f0 | denovo7264\_f0 | denovo7342\_f0 | denovo7394\_f0 | denovo7420\_f0 | denovo7524\_f0 | denovo7550\_f0 | denovo7576\_f0 | denovo7628\_f0 | denovo7680\_f0 | denovo7706\_f0 | denovo7758\_f0 | denovo7810\_f0 | denovo7992\_f0 | denovo8044\_f0 | denovo8070\_f0 | denovo8226\_f0 | denovo8252\_f0 | denovo8278\_f0 | denovo8304\_f0 | denovo8408\_f0 | denovo8434\_f0 | denovo8460\_f0 | denovo8590\_f0 | denovo8642\_f0 | denovo8668\_f0 | denovo8694\_f0 | denovo8772\_f0 | denovo8824\_f0 | denovo8850\_f0 | denovo8902\_f0 | denovo8928\_f0 | denovo8954\_f0 | denovo8980\_f0 | denovo9006\_f0 | denovo9136\_f0 | denovo9162\_f0 | denovo9188\_f0 | denovo9318\_f0 | denovo9344\_f0 | denovo9370\_f0 | denovo9396\_f0 | denovo9448\_f0 | denovo9500\_f0 | denovo9552\_f0 | denovo9604\_f0 | denovo9682\_f0 | denovo9708\_f0 | denovo9734\_f0 | denovo9812\_f0 | denovo9838\_f0 | denovo9864\_f0 | denovo9890\_f0 | denovo9942\_f0 | denovo9968\_f0 | denovo9994\_f0 | denovo10072\_f0 | denovo10176\_f0 | denovo10202\_f0 | denovo10228\_f0 | denovo10280\_f0 | denovo10306\_f0 | denovo10358\_f0 | denovo10384\_f0 | denovo10410\_f0 | denovo10436\_f0 | denovo10488\_f0 | denovo10514\_f0 | denovo10540\_f0 | denovo10618\_f0 | denovo10670\_f0 | denovo10696\_f0 | denovo10774\_f0 | denovo10852\_f0 | denovo10878\_f0 | denovo10930\_f0 | denovo10982\_f0 | denovo11034\_f0 | denovo11086\_f0 | denovo11112\_f0 | denovo11164\_f0 | denovo11190\_f0 | denovo11242\_f0 | denovo11268\_f0 | denovo11320\_f0 | denovo11346\_f0 | denovo11450\_f0 | denovo11554\_f0 | denovo11918\_f0 | denovo11970\_f0 | denovo12074\_f0 |
| SRR803483 | Grapholita dimorpha\* | denovo46\_f0 | denovo98\_f0 | denovo124\_f0 | denovo150\_f0 | denovo228\_f0 | denovo254\_f0 | denovo280\_f0 | denovo306\_f0 | denovo358\_f0 | denovo436\_f0 | denovo540\_f0 | denovo592\_f0 | denovo618\_f0 | denovo748\_f0 | denovo800\_f0 | denovo956\_f0 | denovo982\_f0 | denovo1060\_f0 | denovo1086\_f0 | denovo1164\_f0 | denovo1320\_f0 | denovo1502\_f0 | denovo1528\_f0 | denovo1658\_f0 | denovo1762\_f0 | denovo1788\_f0 | denovo1814\_f0 | denovo1970\_f0 | denovo2152\_f0 | denovo2204\_f0 | denovo2256\_f0 | denovo2282\_f0 | denovo2360\_f0 | denovo2464\_f0 | denovo2490\_f0 | denovo2516\_f0 | denovo2542\_f0 | denovo2594\_f0 | denovo2620\_f0 | denovo2672\_f0 | denovo2776\_f0 | denovo2802\_f0 | denovo2854\_f0 | denovo2880\_f0 | denovo2932\_f0 | denovo3010\_f0 | denovo3062\_f0 | denovo3166\_f0 | denovo3244\_f0 | denovo3270\_f0 | denovo3296\_f0 | denovo3322\_f0 | denovo3348\_f0 | denovo3452\_f0 | denovo3504\_f0 | denovo3608\_f0 | denovo3634\_f0 | denovo3686\_f0 | denovo3712\_f0 | denovo3738\_f0 | denovo3790\_f0 | denovo3868\_f0 | denovo3972\_f0 | denovo3998\_f0 | denovo4050\_f0 | denovo4076\_f0 | denovo4206\_f0 | denovo4310\_f0 | denovo4336\_f0 | denovo4362\_f0 | denovo4414\_f0 | denovo4466\_f0 | denovo4570\_f0 | denovo4648\_f0 | denovo4752\_f0 | denovo4778\_f0 | denovo4804\_f0 | denovo4856\_f0 | denovo4908\_f0 | denovo4934\_f0 | denovo4960\_f0 | denovo4986\_f0 | denovo5012\_f0 | denovo5220\_f0 | denovo5246\_f0 | denovo5272\_f0 | denovo5402\_f0 | denovo5454\_f0 | denovo5532\_f0 | denovo5610\_f0 | denovo5636\_f0 | denovo5662\_f0 | denovo5948\_f0 | denovo6026\_f0 | denovo6078\_f0 | denovo6130\_f0 | denovo6234\_f0 | denovo6260\_f0 | denovo6286\_f0 | denovo6598\_f0 | denovo6624\_f0 | denovo6702\_f0 | denovo6728\_f0 | denovo6780\_f0 | denovo6832\_f0 | denovo6884\_f0 | denovo7014\_f0 | denovo7092\_f0 | denovo7144\_f0 | denovo7170\_f0 | denovo7274\_f0 | denovo7352\_f0 | denovo7404\_f0 | denovo7430\_f0 | denovo7534\_f0 | denovo7560\_f0 | denovo7586\_f0 | denovo7638\_f0 | denovo7690\_f0 | denovo7716\_f0 | denovo7768\_f0 | denovo7820\_f0 | denovo8002\_f0 | denovo8054\_f0 | denovo8080\_f0 | denovo8236\_f0 | denovo8262\_f0 | denovo8288\_f0 | denovo8314\_f0 | denovo8418\_f0 | denovo8444\_f0 | denovo8470\_f0 | denovo8600\_f0 | denovo8652\_f0 | denovo8678\_f0 | denovo8704\_f0 | denovo8782\_f0 | denovo8834\_f0 | denovo8860\_f0 | denovo8912\_f0 | denovo8938\_f0 | denovo8964\_f0 | denovo8990\_f0 | denovo9016\_f0 | denovo9146\_f0 | denovo9172\_f0 | denovo9198\_f0 | denovo9328\_f0 | denovo9354\_f0 | denovo9380\_f0 | denovo9406\_f0 | denovo9458\_f0 | denovo9510\_f0 | denovo9562\_f0 | denovo9614\_f0 | denovo9692\_f0 | denovo9718\_f0 | denovo9744\_f0 | denovo9822\_f0 | denovo9848\_f0 | denovo9874\_f0 | denovo9900\_f0 | denovo9952\_f0 | denovo9978\_f0 | denovo10004\_f0 | denovo10082\_f0 | denovo10186\_f0 | denovo10212\_f0 | denovo10238\_f0 | denovo10290\_f0 | denovo10316\_f0 | denovo10368\_f0 | denovo10394\_f0 | denovo10420\_f0 | denovo10446\_f0 | denovo10498\_f0 | denovo10524\_f0 | denovo10550\_f0 | denovo10628\_f0 | denovo10680\_f0 | denovo10706\_f0 | denovo10784\_f0 | denovo10862\_f0 | denovo10888\_f0 | denovo10940\_f0 | denovo10992\_f0 | denovo11044\_f0 | denovo11096\_f0 | denovo11122\_f0 | denovo11174\_f0 | denovo11200\_f0 | denovo11252\_f0 | denovo11278\_f0 | denovo11330\_f0 | denovo11356\_f0 | denovo11460\_f0 | denovo11564\_f0 | denovo11928\_f0 | denovo11980\_f0 | denovo12084\_f0 |
| FG120035 | Dalcera abrasa\* | denovo31\_f0 | denovo83\_f0 | denovo109\_f0 | denovo135\_f0 | denovo213\_f0 | denovo239\_f0 | denovo265\_f0 | denovo291\_f0 | denovo343\_f0 | denovo421\_f0 | denovo525\_f0 | denovo577\_f0 | denovo603\_f0 | denovo733\_f0 | denovo785\_f0 | denovo941\_f0 | denovo967\_f0 | denovo1045\_f0 | denovo1071\_f0 | denovo1149\_f0 | denovo1305\_f0 | denovo1487\_f0 | denovo1513\_f0 | denovo1643\_f0 | denovo1747\_f0 | denovo1773\_f0 | denovo1799\_f0 | denovo1955\_f0 | denovo2137\_f0 | denovo2189\_f0 | denovo2241\_f0 | denovo2267\_f0 | denovo2345\_f0 | denovo2449\_f0 | denovo2475\_f0 | denovo2501\_f0 | denovo2527\_f0 | denovo2579\_f0 | denovo2605\_f0 | denovo2657\_f0 | denovo2761\_f0 | denovo2787\_f0 | denovo2839\_f0 | denovo2865\_f0 | denovo2917\_f0 | denovo2995\_f0 | denovo3047\_f0 | denovo3151\_f0 | denovo3229\_f0 | denovo3255\_f0 | denovo3281\_f0 | denovo3307\_f0 | denovo3333\_f0 | denovo3437\_f0 | denovo3489\_f0 | denovo3593\_f0 | denovo3619\_f0 | denovo3671\_f0 | denovo3697\_f0 | denovo3723\_f0 | denovo3775\_f0 | denovo3853\_f0 | denovo3957\_f0 | denovo3983\_f0 | denovo4035\_f0 | denovo4061\_f0 | denovo4191\_f0 | denovo4295\_f0 | denovo4321\_f0 | denovo4347\_f0 | denovo4399\_f0 | denovo4451\_f0 | denovo4555\_f0 | denovo4633\_f0 | denovo4737\_f0 | denovo4763\_f0 | denovo4789\_f0 | denovo4841\_f0 | denovo4893\_f0 | denovo4919\_f0 | denovo4945\_f0 | denovo4971\_f0 | denovo4997\_f0 | denovo5205\_f0 | denovo5231\_f0 | denovo5257\_f0 | denovo5387\_f0 | denovo5439\_f0 | denovo5517\_f0 | denovo5595\_f0 | denovo5621\_f0 | denovo5647\_f0 | denovo5933\_f0 | denovo6011\_f0 | denovo6063\_f0 | denovo6115\_f0 | denovo6219\_f0 | denovo6245\_f0 | denovo6271\_f0 | denovo6583\_f0 | denovo6609\_f0 | denovo6687\_f0 | denovo6713\_f0 | denovo6765\_f0 | denovo6817\_f0 | denovo6869\_f0 | denovo6999\_f0 | denovo7077\_f0 | denovo7129\_f0 | denovo7155\_f0 | denovo7259\_f0 | denovo7337\_f0 | denovo7389\_f0 | denovo7415\_f0 | denovo7519\_f0 | denovo7545\_f0 | denovo7571\_f0 | denovo7623\_f0 | denovo7675\_f0 | denovo7701\_f0 | denovo7753\_f0 | denovo7805\_f0 | denovo7987\_f0 | denovo8039\_f0 | denovo8065\_f0 | denovo8221\_f0 | denovo8247\_f0 | denovo8273\_f0 | denovo8299\_f0 | denovo8403\_f0 | denovo8429\_f0 | denovo8455\_f0 | denovo8585\_f0 | denovo8637\_f0 | denovo8663\_f0 | denovo8689\_f0 | denovo8767\_f0 | denovo8819\_f0 | denovo8845\_f0 | denovo8897\_f0 | denovo8923\_f0 | denovo8949\_f0 | denovo8975\_f0 | denovo9001\_f0 | denovo9131\_f0 | denovo9157\_f0 | denovo9183\_f0 | denovo9313\_f0 | denovo9339\_f0 | denovo9365\_f0 | denovo9391\_f0 | denovo9443\_f0 | denovo9495\_f0 | denovo9547\_f0 | denovo9599\_f0 | denovo9677\_f0 | denovo9703\_f0 | denovo9729\_f0 | denovo9807\_f0 | denovo9833\_f0 | denovo9859\_f0 | denovo9885\_f0 | denovo9937\_f0 | denovo9963\_f0 | denovo9989\_f0 | denovo10067\_f0 | denovo10171\_f0 | denovo10197\_f0 | denovo10223\_f0 | denovo10275\_f0 | denovo10301\_f0 | denovo10353\_f0 | denovo10379\_f0 | denovo10405\_f0 | denovo10431\_f0 | denovo10483\_f0 | denovo10509\_f0 | denovo10535\_f0 | denovo10613\_f0 | denovo10665\_f0 | denovo10691\_f0 | denovo10769\_f0 | denovo10847\_f0 | denovo10873\_f0 | denovo10925\_f0 | denovo10977\_f0 | denovo11029\_f0 | denovo11081\_f0 | denovo11107\_f0 | denovo11159\_f0 | denovo11185\_f0 | denovo11237\_f0 | denovo11263\_f0 | denovo11315\_f0 | denovo11341\_f0 | denovo11445\_f0 | denovo11549\_f0 | denovo11913\_f0 | denovo11965\_f0 | denovo12069\_f0 |
| FG120046B | Lacosoma ludolpha\* | denovo32\_f0 | denovo84\_f0 | denovo110\_f0 | denovo136\_f0 | denovo214\_f0 | denovo240\_f0 | denovo266\_f0 | denovo292\_f0 | denovo344\_f0 | denovo422\_f0 | denovo526\_f0 | denovo578\_f0 | denovo604\_f0 | denovo734\_f0 | denovo786\_f0 | denovo942\_f0 | denovo968\_f0 | denovo1046\_f0 | denovo1072\_f0 | denovo1150\_f0 | denovo1306\_f0 | denovo1488\_f0 | denovo1514\_f0 | denovo1644\_f0 | denovo1748\_f0 | denovo1774\_f0 | denovo1800\_f0 | denovo1956\_f0 | denovo2138\_f0 | denovo2190\_f0 | denovo2242\_f0 | denovo2268\_f0 | denovo2346\_f0 | denovo2450\_f0 | denovo2476\_f0 | denovo2502\_f0 | denovo2528\_f0 | denovo2580\_f0 | denovo2606\_f0 | denovo2658\_f0 | denovo2762\_f0 | denovo2788\_f0 | denovo2840\_f0 | denovo2866\_f0 | denovo2918\_f0 | denovo2996\_f0 | denovo3048\_f0 | denovo3152\_f0 | denovo3230\_f0 | denovo3256\_f0 | denovo3282\_f0 | denovo3308\_f0 | denovo3334\_f0 | denovo3438\_f0 | denovo3490\_f0 | denovo3594\_f0 | denovo3620\_f0 | denovo3672\_f0 | denovo3698\_f0 | denovo3724\_f0 | denovo3776\_f0 | denovo3854\_f0 | denovo3958\_f0 | denovo3984\_f0 | denovo4036\_f0 | denovo4062\_f0 | denovo4192\_f0 | denovo4296\_f0 | denovo4322\_f0 | denovo4348\_f0 | denovo4400\_f0 | denovo4452\_f0 | denovo4556\_f0 | denovo4634\_f0 | denovo4738\_f0 | denovo4764\_f0 | denovo4790\_f0 | denovo4842\_f0 | denovo4894\_f0 | denovo4920\_f0 | denovo4946\_f0 | denovo4972\_f0 | denovo4998\_f0 | denovo5206\_f0 | denovo5232\_f0 | denovo5258\_f0 | denovo5388\_f0 | denovo5440\_f0 | denovo5518\_f0 | denovo5596\_f0 | denovo5622\_f0 | denovo5648\_f0 | denovo5934\_f0 | denovo6012\_f0 | denovo6064\_f0 | denovo6116\_f0 | denovo6220\_f0 | denovo6246\_f0 | denovo6272\_f0 | denovo6584\_f0 | denovo6610\_f0 | denovo6688\_f0 | denovo6714\_f0 | denovo6766\_f0 | denovo6818\_f0 | denovo6870\_f0 | denovo7000\_f0 | denovo7078\_f0 | denovo7130\_f0 | denovo7156\_f0 | denovo7260\_f0 | denovo7338\_f0 | denovo7390\_f0 | denovo7416\_f0 | denovo7520\_f0 | denovo7546\_f0 | denovo7572\_f0 | denovo7624\_f0 | denovo7676\_f0 | denovo7702\_f0 | denovo7754\_f0 | denovo7806\_f0 | denovo7988\_f0 | denovo8040\_f0 | denovo8066\_f0 | denovo8222\_f0 | denovo8248\_f0 | denovo8274\_f0 | denovo8300\_f0 | denovo8404\_f0 | denovo8430\_f0 | denovo8456\_f0 | denovo8586\_f0 | denovo8638\_f0 | denovo8664\_f0 | denovo8690\_f0 | denovo8768\_f0 | denovo8820\_f0 | denovo8846\_f0 | denovo8898\_f0 | denovo8924\_f0 | denovo8950\_f0 | denovo8976\_f0 | denovo9002\_f0 | denovo9132\_f0 | denovo9158\_f0 | denovo9184\_f0 | denovo9314\_f0 | denovo9340\_f0 | denovo9366\_f0 | denovo9392\_f0 | denovo9444\_f0 | denovo9496\_f0 | denovo9548\_f0 | denovo9600\_f0 | denovo9678\_f0 | denovo9704\_f0 | denovo9730\_f0 | denovo9808\_f0 | denovo9834\_f0 | denovo9860\_f0 | denovo9886\_f0 | denovo9938\_f0 | denovo9964\_f0 | denovo9990\_f0 | denovo10068\_f0 | denovo10172\_f0 | denovo10198\_f0 | denovo10224\_f0 | denovo10276\_f0 | denovo10302\_f0 | denovo10354\_f0 | denovo10380\_f0 | denovo10406\_f0 | denovo10432\_f0 | denovo10484\_f0 | denovo10510\_f0 | denovo10536\_f0 | denovo10614\_f0 | denovo10666\_f0 | denovo10692\_f0 | denovo10770\_f0 | denovo10848\_f0 | denovo10874\_f0 | denovo10926\_f0 | denovo10978\_f0 | denovo11030\_f0 | denovo11082\_f0 | denovo11108\_f0 | denovo11160\_f0 | denovo11186\_f0 | denovo11238\_f0 | denovo11264\_f0 | denovo11316\_f0 | denovo11342\_f0 | denovo11446\_f0 | denovo11550\_f0 | denovo11914\_f0 | denovo11966\_f0 | denovo12070\_f0 |
| GNV129007 | Urodus parvula\* | denovo41\_f0 | denovo93\_f0 | denovo119\_f0 | denovo145\_f0 | denovo223\_f0 | denovo249\_f0 | denovo275\_f0 | denovo301\_f0 | denovo353\_f0 | denovo431\_f0 | denovo535\_f0 | denovo587\_f0 | denovo613\_f0 | denovo743\_f0 | denovo795\_f0 | denovo951\_f0 | denovo977\_f0 | denovo1055\_f0 | denovo1081\_f0 | denovo1159\_f0 | denovo1315\_f0 | denovo1497\_f0 | denovo1523\_f0 | denovo1653\_f0 | denovo1757\_f0 | denovo1783\_f0 | denovo1809\_f0 | denovo1965\_f0 | denovo2147\_f0 | denovo2199\_f0 | denovo2251\_f0 | denovo2277\_f0 | denovo2355\_f0 | denovo2459\_f0 | denovo2485\_f0 | denovo2511\_f0 | denovo2537\_f0 | denovo2589\_f0 | denovo2615\_f0 | denovo2667\_f0 | denovo2771\_f0 | denovo2797\_f0 | denovo2849\_f0 | denovo2875\_f0 | denovo2927\_f0 | denovo3005\_f0 | denovo3057\_f0 | denovo3161\_f0 | denovo3239\_f0 | denovo3265\_f0 | denovo3291\_f0 | denovo3317\_f0 | denovo3343\_f0 | denovo3447\_f0 | denovo3499\_f0 | denovo3603\_f0 | denovo3629\_f0 | denovo3681\_f0 | denovo3707\_f0 | denovo3733\_f0 | denovo3785\_f0 | denovo3863\_f0 | denovo3967\_f0 | denovo3993\_f0 | denovo4045\_f0 | denovo4071\_f0 | denovo4201\_f0 | denovo4305\_f0 | denovo4331\_f0 | denovo4357\_f0 | denovo4409\_f0 | denovo4461\_f0 | denovo4565\_f0 | denovo4643\_f0 | denovo4747\_f0 | denovo4773\_f0 | denovo4799\_f0 | denovo4851\_f0 | denovo4903\_f0 | denovo4929\_f0 | denovo4955\_f0 | denovo4981\_f0 | denovo5007\_f0 | denovo5215\_f0 | denovo5241\_f0 | denovo5267\_f0 | denovo5397\_f0 | denovo5449\_f0 | denovo5527\_f0 | denovo5605\_f0 | denovo5631\_f0 | denovo5657\_f0 | denovo5943\_f0 | denovo6021\_f0 | denovo6073\_f0 | denovo6125\_f0 | denovo6229\_f0 | denovo6255\_f0 | denovo6281\_f0 | denovo6593\_f0 | denovo6619\_f0 | denovo6697\_f0 | denovo6723\_f0 | denovo6775\_f0 | denovo6827\_f0 | denovo6879\_f0 | denovo7009\_f0 | denovo7087\_f0 | denovo7139\_f0 | denovo7165\_f0 | denovo7269\_f0 | denovo7347\_f0 | denovo7399\_f0 | denovo7425\_f0 | denovo7529\_f0 | denovo7555\_f0 | denovo7581\_f0 | denovo7633\_f0 | denovo7685\_f0 | denovo7711\_f0 | denovo7763\_f0 | denovo7815\_f0 | denovo7997\_f0 | denovo8049\_f0 | denovo8075\_f0 | denovo8231\_f0 | denovo8257\_f0 | denovo8283\_f0 | denovo8309\_f0 | denovo8413\_f0 | denovo8439\_f0 | denovo8465\_f0 | denovo8595\_f0 | denovo8647\_f0 | denovo8673\_f0 | denovo8699\_f0 | denovo8777\_f0 | denovo8829\_f0 | denovo8855\_f0 | denovo8907\_f0 | denovo8933\_f0 | denovo8959\_f0 | denovo8985\_f0 | denovo9011\_f0 | denovo9141\_f0 | denovo9167\_f0 | denovo9193\_f0 | denovo9323\_f0 | denovo9349\_f0 | denovo9375\_f0 | denovo9401\_f0 | denovo9453\_f0 | denovo9505\_f0 | denovo9557\_f0 | denovo9609\_f0 | denovo9687\_f0 | denovo9713\_f0 | denovo9739\_f0 | denovo9817\_f0 | denovo9843\_f0 | denovo9869\_f0 | denovo9895\_f0 | denovo9947\_f0 | denovo9973\_f0 | denovo9999\_f0 | denovo10077\_f0 | denovo10181\_f0 | denovo10207\_f0 | denovo10233\_f0 | denovo10285\_f0 | denovo10311\_f0 | denovo10363\_f0 | denovo10389\_f0 | denovo10415\_f0 | denovo10441\_f0 | denovo10493\_f0 | denovo10519\_f0 | denovo10545\_f0 | denovo10623\_f0 | denovo10675\_f0 | denovo10701\_f0 | denovo10779\_f0 | denovo10857\_f0 | denovo10883\_f0 | denovo10935\_f0 | denovo10987\_f0 | denovo11039\_f0 | denovo11091\_f0 | denovo11117\_f0 | denovo11169\_f0 | denovo11195\_f0 | denovo11247\_f0 | denovo11273\_f0 | denovo11325\_f0 | denovo11351\_f0 | denovo11455\_f0 | denovo11559\_f0 | denovo11923\_f0 | denovo11975\_f0 | denovo12079\_f0 |
| SW130126 | Lyssa zampa\* | denovo50\_f0 | denovo102\_f0 | denovo128\_f0 | denovo154\_f0 | denovo232\_f0 | denovo258\_f0 | denovo284\_f0 | denovo310\_f0 | denovo362\_f0 | denovo440\_f0 | denovo544\_f0 | denovo596\_f0 | denovo622\_f0 | denovo752\_f0 | denovo804\_f0 | denovo960\_f0 | denovo986\_f0 | denovo1064\_f0 | denovo1090\_f0 | denovo1168\_f0 | denovo1324\_f0 | denovo1506\_f0 | denovo1532\_f0 | denovo1662\_f0 | denovo1766\_f0 | denovo1792\_f0 | denovo1818\_f0 | denovo1974\_f0 | denovo2156\_f0 | denovo2208\_f0 | denovo2260\_f0 | denovo2286\_f0 | denovo2364\_f0 | denovo2468\_f0 | denovo2494\_f0 | denovo2520\_f0 | denovo2546\_f0 | denovo2598\_f0 | denovo2624\_f0 | denovo2676\_f0 | denovo2780\_f0 | denovo2806\_f0 | denovo2858\_f0 | denovo2884\_f0 | denovo2936\_f0 | denovo3014\_f0 | denovo3066\_f0 | denovo3170\_f0 | denovo3248\_f0 | denovo3274\_f0 | denovo3300\_f0 | denovo3326\_f0 | denovo3352\_f0 | denovo3456\_f0 | denovo3508\_f0 | denovo3612\_f0 | denovo3638\_f0 | denovo3690\_f0 | denovo3716\_f0 | denovo3742\_f0 | denovo3794\_f0 | denovo3872\_f0 | denovo3976\_f0 | denovo4002\_f0 | denovo4054\_f0 | denovo4080\_f0 | denovo4210\_f0 | denovo4314\_f0 | denovo4340\_f0 | denovo4366\_f0 | denovo4418\_f0 | denovo4470\_f0 | denovo4574\_f0 | denovo4652\_f0 | denovo4756\_f0 | denovo4782\_f0 | denovo4808\_f0 | denovo4860\_f0 | denovo4912\_f0 | denovo4938\_f0 | denovo4964\_f0 | denovo4990\_f0 | denovo5016\_f0 | denovo5224\_f0 | denovo5250\_f0 | denovo5276\_f0 | denovo5406\_f0 | denovo5458\_f0 | denovo5536\_f0 | denovo5614\_f0 | denovo5640\_f0 | denovo5666\_f0 | denovo5952\_f0 | denovo6030\_f0 | denovo6082\_f0 | denovo6134\_f0 | denovo6238\_f0 | denovo6264\_f0 | denovo6290\_f0 | denovo6602\_f0 | denovo6628\_f0 | denovo6706\_f0 | denovo6732\_f0 | denovo6784\_f0 | denovo6836\_f0 | denovo6888\_f0 | denovo7018\_f0 | denovo7096\_f0 | denovo7148\_f0 | denovo7174\_f0 | denovo7278\_f0 | denovo7356\_f0 | denovo7408\_f0 | denovo7434\_f0 | denovo7538\_f0 | denovo7564\_f0 | denovo7590\_f0 | denovo7642\_f0 | denovo7694\_f0 | denovo7720\_f0 | denovo7772\_f0 | denovo7824\_f0 | denovo8006\_f0 | denovo8058\_f0 | denovo8084\_f0 | denovo8240\_f0 | denovo8266\_f0 | denovo8292\_f0 | denovo8318\_f0 | denovo8422\_f0 | denovo8448\_f0 | denovo8474\_f0 | denovo8604\_f0 | denovo8656\_f0 | denovo8682\_f0 | denovo8708\_f0 | denovo8786\_f0 | denovo8838\_f0 | denovo8864\_f0 | denovo8916\_f0 | denovo8942\_f0 | denovo8968\_f0 | denovo8994\_f0 | denovo9020\_f0 | denovo9150\_f0 | denovo9176\_f0 | denovo9202\_f0 | denovo9332\_f0 | denovo9358\_f0 | denovo9384\_f0 | denovo9410\_f0 | denovo9462\_f0 | denovo9514\_f0 | denovo9566\_f0 | denovo9618\_f0 | denovo9696\_f0 | denovo9722\_f0 | denovo9748\_f0 | denovo9826\_f0 | denovo9852\_f0 | denovo9878\_f0 | denovo9904\_f0 | denovo9956\_f0 | denovo9982\_f0 | denovo10008\_f0 | denovo10086\_f0 | denovo10190\_f0 | denovo10216\_f0 | denovo10242\_f0 | denovo10294\_f0 | denovo10320\_f0 | denovo10372\_f0 | denovo10398\_f0 | denovo10424\_f0 | denovo10450\_f0 | denovo10502\_f0 | denovo10528\_f0 | denovo10554\_f0 | denovo10632\_f0 | denovo10684\_f0 | denovo10710\_f0 | denovo10788\_f0 | denovo10866\_f0 | denovo10892\_f0 | denovo10944\_f0 | denovo10996\_f0 | denovo11048\_f0 | denovo11100\_f0 | denovo11126\_f0 | denovo11178\_f0 | denovo11204\_f0 | denovo11256\_f0 | denovo11282\_f0 | denovo11334\_f0 | denovo11360\_f0 | denovo11464\_f0 | denovo11568\_f0 | denovo11932\_f0 | denovo11984\_f0 | denovo12088\_f0 |
| SW130103 | Anigraea sp. | denovo49\_f0 | denovo101\_f0 | denovo127\_f0 | denovo153\_f0 | denovo231\_f0 | denovo257\_f0 | denovo283\_f0 | denovo309\_f0 | denovo361\_f0 | denovo439\_f0 | denovo543\_f0 | denovo595\_f0 | denovo621\_f0 | denovo751\_f0 | denovo803\_f0 | denovo959\_f0 | denovo985\_f0 | denovo1063\_f0 | denovo1089\_f0 | denovo1167\_f0 | denovo1323\_f0 | denovo1505\_f0 | denovo1531\_f0 | denovo1661\_f0 | denovo1765\_f0 | denovo1791\_f0 | denovo1817\_f0 | denovo1973\_f0 | denovo2155\_f0 | denovo2207\_f0 | denovo2259\_f0 | denovo2285\_f0 | denovo2363\_f0 | denovo2467\_f0 | denovo2493\_f0 | denovo2519\_f0 | denovo2545\_f0 | denovo2597\_f0 | denovo2623\_f0 | denovo2675\_f0 | denovo2779\_f0 | denovo2805\_f0 | denovo2857\_f0 | denovo2883\_f0 | denovo2935\_f0 | denovo3013\_f0 | denovo3065\_f0 | denovo3169\_f0 | denovo3247\_f0 | denovo3273\_f0 | denovo3299\_f0 | denovo3325\_f0 | denovo3351\_f0 | denovo3455\_f0 | denovo3507\_f0 | denovo3611\_f0 | denovo3637\_f0 | denovo3689\_f0 | denovo3715\_f0 | denovo3741\_f0 | denovo3793\_f0 | denovo3871\_f0 | denovo3975\_f0 | denovo4001\_f0 | denovo4053\_f0 | denovo4079\_f0 | denovo4209\_f0 | denovo4313\_f0 | denovo4339\_f0 | denovo4365\_f0 | denovo4417\_f0 | denovo4469\_f0 | denovo4573\_f0 | denovo4651\_f0 | denovo4755\_f0 | denovo4781\_f0 | denovo4807\_f0 | denovo4859\_f0 | denovo4911\_f0 | denovo4937\_f0 | denovo4963\_f0 | denovo4989\_f0 | denovo5015\_f0 | denovo5223\_f0 | denovo5249\_f0 | denovo5275\_f0 | denovo5405\_f0 | denovo5457\_f0 | denovo5535\_f0 | denovo5613\_f0 | denovo5639\_f0 | denovo5665\_f0 | denovo5951\_f0 | denovo6029\_f0 | denovo6081\_f0 | denovo6133\_f0 | denovo6237\_f0 | denovo6263\_f0 | denovo6289\_f0 | denovo6601\_f0 | denovo6627\_f0 | denovo6705\_f0 | denovo6731\_f0 | denovo6783\_f0 | denovo6835\_f0 | denovo6887\_f0 | denovo7017\_f0 | denovo7095\_f0 | denovo7147\_f0 | denovo7173\_f0 | denovo7277\_f0 | denovo7355\_f0 | denovo7407\_f0 | denovo7433\_f0 | denovo7537\_f0 | denovo7563\_f0 | denovo7589\_f0 | denovo7641\_f0 | denovo7693\_f0 | denovo7719\_f0 | denovo7771\_f0 | denovo7823\_f0 | denovo8005\_f0 | denovo8057\_f0 | denovo8083\_f0 | denovo8239\_f0 | denovo8265\_f0 | denovo8291\_f0 | denovo8317\_f0 | denovo8421\_f0 | denovo8447\_f0 | denovo8473\_f0 | denovo8603\_f0 | denovo8655\_f0 | denovo8681\_f0 | denovo8707\_f0 | denovo8785\_f0 | denovo8837\_f0 | denovo8863\_f0 | denovo8915\_f0 | denovo8941\_f0 | denovo8967\_f0 | denovo8993\_f0 | denovo9019\_f0 | denovo9149\_f0 | denovo9175\_f0 | denovo9201\_f0 | denovo9331\_f0 | denovo9357\_f0 | denovo9383\_f0 | denovo9409\_f0 | denovo9461\_f0 | denovo9513\_f0 | denovo9565\_f0 | denovo9617\_f0 | denovo9695\_f0 | denovo9721\_f0 | denovo9747\_f0 | denovo9825\_f0 | denovo9851\_f0 | denovo9877\_f0 | denovo9903\_f0 | denovo9955\_f0 | denovo9981\_f0 | denovo10007\_f0 | denovo10085\_f0 | denovo10189\_f0 | denovo10215\_f0 | denovo10241\_f0 | denovo10293\_f0 | denovo10319\_f0 | denovo10371\_f0 | denovo10397\_f0 | denovo10423\_f0 | denovo10449\_f0 | denovo10501\_f0 | denovo10527\_f0 | denovo10553\_f0 | denovo10631\_f0 | denovo10683\_f0 | denovo10709\_f0 | denovo10787\_f0 | denovo10865\_f0 | denovo10891\_f0 | denovo10943\_f0 | denovo10995\_f0 | denovo11047\_f0 | denovo11099\_f0 | denovo11125\_f0 | denovo11177\_f0 | denovo11203\_f0 | denovo11255\_f0 | denovo11281\_f0 | denovo11333\_f0 | denovo11359\_f0 | denovo11463\_f0 | denovo11567\_f0 | denovo11931\_f0 | denovo11983\_f0 | denovo12087\_f0 |
| Callid | Pterodecta felderi\* | denovo27\_f0 | denovo79\_f0 | denovo105\_f0 | denovo131\_f0 | denovo209\_f0 | denovo235\_f0 | denovo261\_f0 | denovo287\_f0 | denovo339\_f0 | denovo417\_f0 | denovo521\_f0 | denovo573\_f0 | denovo599\_f0 | denovo729\_f0 | denovo781\_f0 | denovo937\_f0 | denovo963\_f0 | denovo1041\_f0 | denovo1067\_f0 | denovo1145\_f0 | denovo1301\_f0 | denovo1483\_f0 | denovo1509\_f0 | denovo1639\_f0 | denovo1743\_f0 | denovo1769\_f0 | denovo1795\_f0 | denovo1951\_f0 | denovo2133\_f0 | denovo2185\_f0 | denovo2237\_f0 | denovo2263\_f0 | denovo2341\_f0 | denovo2445\_f0 | denovo2471\_f0 | denovo2497\_f0 | denovo2523\_f0 | denovo2575\_f0 | denovo2601\_f0 | denovo2653\_f0 | denovo2757\_f0 | denovo2783\_f0 | denovo2835\_f0 | denovo2861\_f0 | denovo2913\_f0 | denovo2991\_f0 | denovo3043\_f0 | denovo3147\_f0 | denovo3225\_f0 | denovo3251\_f0 | denovo3277\_f0 | denovo3303\_f0 | denovo3329\_f0 | denovo3433\_f0 | denovo3485\_f0 | denovo3589\_f0 | denovo3615\_f0 | denovo3667\_f0 | denovo3693\_f0 | denovo3719\_f0 | denovo3771\_f0 | denovo3849\_f0 | denovo3953\_f0 | denovo3979\_f0 | denovo4031\_f0 | denovo4057\_f0 | denovo4187\_f0 | denovo4291\_f0 | denovo4317\_f0 | denovo4343\_f0 | denovo4395\_f0 | denovo4447\_f0 | denovo4551\_f0 | denovo4629\_f0 | denovo4733\_f0 | denovo4759\_f0 | denovo4785\_f0 | denovo4837\_f0 | denovo4889\_f0 | denovo4915\_f0 | denovo4941\_f0 | denovo4967\_f0 | denovo4993\_f0 | denovo5201\_f0 | denovo5227\_f0 | denovo5253\_f0 | denovo5383\_f0 | denovo5435\_f0 | denovo5513\_f0 | denovo5591\_f0 | denovo5617\_f0 | denovo5643\_f0 | denovo5929\_f0 | denovo6007\_f0 | denovo6059\_f0 | denovo6111\_f0 | denovo6215\_f0 | denovo6241\_f0 | denovo6267\_f0 | denovo6579\_f0 | denovo6605\_f0 | denovo6683\_f0 | denovo6709\_f0 | denovo6761\_f0 | denovo6813\_f0 | denovo6865\_f0 | denovo6995\_f0 | denovo7073\_f0 | denovo7125\_f0 | denovo7151\_f0 | denovo7255\_f0 | denovo7333\_f0 | denovo7385\_f0 | denovo7411\_f0 | denovo7515\_f0 | denovo7541\_f0 | denovo7567\_f0 | denovo7619\_f0 | denovo7671\_f0 | denovo7697\_f0 | denovo7749\_f0 | denovo7801\_f0 | denovo7983\_f0 | denovo8035\_f0 | denovo8061\_f0 | denovo8217\_f0 | denovo8243\_f0 | denovo8269\_f0 | denovo8295\_f0 | denovo8399\_f0 | denovo8425\_f0 | denovo8451\_f0 | denovo8581\_f0 | denovo8633\_f0 | denovo8659\_f0 | denovo8685\_f0 | denovo8763\_f0 | denovo8815\_f0 | denovo8841\_f0 | denovo8893\_f0 | denovo8919\_f0 | denovo8945\_f0 | denovo8971\_f0 | denovo8997\_f0 | denovo9127\_f0 | denovo9153\_f0 | denovo9179\_f0 | denovo9309\_f0 | denovo9335\_f0 | denovo9361\_f0 | denovo9387\_f0 | denovo9439\_f0 | denovo9491\_f0 | denovo9543\_f0 | denovo9595\_f0 | denovo9673\_f0 | denovo9699\_f0 | denovo9725\_f0 | denovo9803\_f0 | denovo9829\_f0 | denovo9855\_f0 | denovo9881\_f0 | denovo9933\_f0 | denovo9959\_f0 | denovo9985\_f0 | denovo10063\_f0 | denovo10167\_f0 | denovo10193\_f0 | denovo10219\_f0 | denovo10271\_f0 | denovo10297\_f0 | denovo10349\_f0 | denovo10375\_f0 | denovo10401\_f0 | denovo10427\_f0 | denovo10479\_f0 | denovo10505\_f0 | denovo10531\_f0 | denovo10609\_f0 | denovo10661\_f0 | denovo10687\_f0 | denovo10765\_f0 | denovo10843\_f0 | denovo10869\_f0 | denovo10921\_f0 | denovo10973\_f0 | denovo11025\_f0 | denovo11077\_f0 | denovo11103\_f0 | denovo11155\_f0 | denovo11181\_f0 | denovo11233\_f0 | denovo11259\_f0 | denovo11311\_f0 | denovo11337\_f0 | denovo11441\_f0 | denovo11545\_f0 | denovo11909\_f0 | denovo11961\_f0 | denovo12065\_f0 |
| FG120070B | Artace sp.\* | denovo34\_f0 | denovo86\_f0 | denovo112\_f0 | denovo138\_f0 | denovo216\_f0 | denovo242\_f0 | denovo268\_f0 | denovo294\_f0 | denovo346\_f0 | denovo424\_f0 | denovo528\_f0 | denovo580\_f0 | denovo606\_f0 | denovo736\_f0 | denovo788\_f0 | denovo944\_f0 | denovo970\_f0 | denovo1048\_f0 | denovo1074\_f0 | denovo1152\_f0 | denovo1308\_f0 | denovo1490\_f0 | denovo1516\_f0 | denovo1646\_f0 | denovo1750\_f0 | denovo1776\_f0 | denovo1802\_f0 | denovo1958\_f0 | denovo2140\_f0 | denovo2192\_f0 | denovo2244\_f0 | denovo2270\_f0 | denovo2348\_f0 | denovo2452\_f0 | denovo2478\_f0 | denovo2504\_f0 | denovo2530\_f0 | denovo2582\_f0 | denovo2608\_f0 | denovo2660\_f0 | denovo2764\_f0 | denovo2790\_f0 | denovo2842\_f0 | denovo2868\_f0 | denovo2920\_f0 | denovo2998\_f0 | denovo3050\_f0 | denovo3154\_f0 | denovo3232\_f0 | denovo3258\_f0 | denovo3284\_f0 | denovo3310\_f0 | denovo3336\_f0 | denovo3440\_f0 | denovo3492\_f0 | denovo3596\_f0 | denovo3622\_f0 | denovo3674\_f0 | denovo3700\_f0 | denovo3726\_f0 | denovo3778\_f0 | denovo3856\_f0 | denovo3960\_f0 | denovo3986\_f0 | denovo4038\_f0 | denovo4064\_f0 | denovo4194\_f0 | denovo4298\_f0 | denovo4324\_f0 | denovo4350\_f0 | denovo4402\_f0 | denovo4454\_f0 | denovo4558\_f0 | denovo4636\_f0 | denovo4740\_f0 | denovo4766\_f0 | denovo4792\_f0 | denovo4844\_f0 | denovo4896\_f0 | denovo4922\_f0 | denovo4948\_f0 | denovo4974\_f0 | denovo5000\_f0 | denovo5208\_f0 | denovo5234\_f0 | denovo5260\_f0 | denovo5390\_f0 | denovo5442\_f0 | denovo5520\_f0 | denovo5598\_f0 | denovo5624\_f0 | denovo5650\_f0 | denovo5936\_f0 | denovo6014\_f0 | denovo6066\_f0 | denovo6118\_f0 | denovo6222\_f0 | denovo6248\_f0 | denovo6274\_f0 | denovo6586\_f0 | denovo6612\_f0 | denovo6690\_f0 | denovo6716\_f0 | denovo6768\_f0 | denovo6820\_f0 | denovo6872\_f0 | denovo7002\_f0 | denovo7080\_f0 | denovo7132\_f0 | denovo7158\_f0 | denovo7262\_f0 | denovo7340\_f0 | denovo7392\_f0 | denovo7418\_f0 | denovo7522\_f0 | denovo7548\_f0 | denovo7574\_f0 | denovo7626\_f0 | denovo7678\_f0 | denovo7704\_f0 | denovo7756\_f0 | denovo7808\_f0 | denovo7990\_f0 | denovo8042\_f0 | denovo8068\_f0 | denovo8224\_f0 | denovo8250\_f0 | denovo8276\_f0 | denovo8302\_f0 | denovo8406\_f0 | denovo8432\_f0 | denovo8458\_f0 | denovo8588\_f0 | denovo8640\_f0 | denovo8666\_f0 | denovo8692\_f0 | denovo8770\_f0 | denovo8822\_f0 | denovo8848\_f0 | denovo8900\_f0 | denovo8926\_f0 | denovo8952\_f0 | denovo8978\_f0 | denovo9004\_f0 | denovo9134\_f0 | denovo9160\_f0 | denovo9186\_f0 | denovo9316\_f0 | denovo9342\_f0 | denovo9368\_f0 | denovo9394\_f0 | denovo9446\_f0 | denovo9498\_f0 | denovo9550\_f0 | denovo9602\_f0 | denovo9680\_f0 | denovo9706\_f0 | denovo9732\_f0 | denovo9810\_f0 | denovo9836\_f0 | denovo9862\_f0 | denovo9888\_f0 | denovo9940\_f0 | denovo9966\_f0 | denovo9992\_f0 | denovo10070\_f0 | denovo10174\_f0 | denovo10200\_f0 | denovo10226\_f0 | denovo10278\_f0 | denovo10304\_f0 | denovo10356\_f0 | denovo10382\_f0 | denovo10408\_f0 | denovo10434\_f0 | denovo10486\_f0 | denovo10512\_f0 | denovo10538\_f0 | denovo10616\_f0 | denovo10668\_f0 | denovo10694\_f0 | denovo10772\_f0 | denovo10850\_f0 | denovo10876\_f0 | denovo10928\_f0 | denovo10980\_f0 | denovo11032\_f0 | denovo11084\_f0 | denovo11110\_f0 | denovo11162\_f0 | denovo11188\_f0 | denovo11240\_f0 | denovo11266\_f0 | denovo11318\_f0 | denovo11344\_f0 | denovo11448\_f0 | denovo11552\_f0 | denovo11916\_f0 | denovo11968\_f0 | denovo12072\_f0 |
| SW130007 | Thubana sp.\* | denovo48\_f0 | denovo100\_f0 | denovo126\_f0 | denovo152\_f0 | denovo230\_f0 | denovo256\_f0 | denovo282\_f0 | denovo308\_f0 | denovo360\_f0 | denovo438\_f0 | denovo542\_f0 | denovo594\_f0 | denovo620\_f0 | denovo750\_f0 | denovo802\_f0 | denovo958\_f0 | denovo984\_f0 | denovo1062\_f0 | denovo1088\_f0 | denovo1166\_f0 | denovo1322\_f0 | denovo1504\_f0 | denovo1530\_f0 | denovo1660\_f0 | denovo1764\_f0 | denovo1790\_f0 | denovo1816\_f0 | denovo1972\_f0 | denovo2154\_f0 | denovo2206\_f0 | denovo2258\_f0 | denovo2284\_f0 | denovo2362\_f0 | denovo2466\_f0 | denovo2492\_f0 | denovo2518\_f0 | denovo2544\_f0 | denovo2596\_f0 | denovo2622\_f0 | denovo2674\_f0 | denovo2778\_f0 | denovo2804\_f0 | denovo2856\_f0 | denovo2882\_f0 | denovo2934\_f0 | denovo3012\_f0 | denovo3064\_f0 | denovo3168\_f0 | denovo3246\_f0 | denovo3272\_f0 | denovo3298\_f0 | denovo3324\_f0 | denovo3350\_f0 | denovo3454\_f0 | denovo3506\_f0 | denovo3610\_f0 | denovo3636\_f0 | denovo3688\_f0 | denovo3714\_f0 | denovo3740\_f0 | denovo3792\_f0 | denovo3870\_f0 | denovo3974\_f0 | denovo4000\_f0 | denovo4052\_f0 | denovo4078\_f0 | denovo4208\_f0 | denovo4312\_f0 | denovo4338\_f0 | denovo4364\_f0 | denovo4416\_f0 | denovo4468\_f0 | denovo4572\_f0 | denovo4650\_f0 | denovo4754\_f0 | denovo4780\_f0 | denovo4806\_f0 | denovo4858\_f0 | denovo4910\_f0 | denovo4936\_f0 | denovo4962\_f0 | denovo4988\_f0 | denovo5014\_f0 | denovo5222\_f0 | denovo5248\_f0 | denovo5274\_f0 | denovo5404\_f0 | denovo5456\_f0 | denovo5534\_f0 | denovo5612\_f0 | denovo5638\_f0 | denovo5664\_f0 | denovo5950\_f0 | denovo6028\_f0 | denovo6080\_f0 | denovo6132\_f0 | denovo6236\_f0 | denovo6262\_f0 | denovo6288\_f0 | denovo6600\_f0 | denovo6626\_f0 | denovo6704\_f0 | denovo6730\_f0 | denovo6782\_f0 | denovo6834\_f0 | denovo6886\_f0 | denovo7016\_f0 | denovo7094\_f0 | denovo7146\_f0 | denovo7172\_f0 | denovo7276\_f0 | denovo7354\_f0 | denovo7406\_f0 | denovo7432\_f0 | denovo7536\_f0 | denovo7562\_f0 | denovo7588\_f0 | denovo7640\_f0 | denovo7692\_f0 | denovo7718\_f0 | denovo7770\_f0 | denovo7822\_f0 | denovo8004\_f0 | denovo8056\_f0 | denovo8082\_f0 | denovo8238\_f0 | denovo8264\_f0 | denovo8290\_f0 | denovo8316\_f0 | denovo8420\_f0 | denovo8446\_f0 | denovo8472\_f0 | denovo8602\_f0 | denovo8654\_f0 | denovo8680\_f0 | denovo8706\_f0 | denovo8784\_f0 | denovo8836\_f0 | denovo8862\_f0 | denovo8914\_f0 | denovo8940\_f0 | denovo8966\_f0 | denovo8992\_f0 | denovo9018\_f0 | denovo9148\_f0 | denovo9174\_f0 | denovo9200\_f0 | denovo9330\_f0 | denovo9356\_f0 | denovo9382\_f0 | denovo9408\_f0 | denovo9460\_f0 | denovo9512\_f0 | denovo9564\_f0 | denovo9616\_f0 | denovo9694\_f0 | denovo9720\_f0 | denovo9746\_f0 | denovo9824\_f0 | denovo9850\_f0 | denovo9876\_f0 | denovo9902\_f0 | denovo9954\_f0 | denovo9980\_f0 | denovo10006\_f0 | denovo10084\_f0 | denovo10188\_f0 | denovo10214\_f0 | denovo10240\_f0 | denovo10292\_f0 | denovo10318\_f0 | denovo10370\_f0 | denovo10396\_f0 | denovo10422\_f0 | denovo10448\_f0 | denovo10500\_f0 | denovo10526\_f0 | denovo10552\_f0 | denovo10630\_f0 | denovo10682\_f0 | denovo10708\_f0 | denovo10786\_f0 | denovo10864\_f0 | denovo10890\_f0 | denovo10942\_f0 | denovo10994\_f0 | denovo11046\_f0 | denovo11098\_f0 | denovo11124\_f0 | denovo11176\_f0 | denovo11202\_f0 | denovo11254\_f0 | denovo11280\_f0 | denovo11332\_f0 | denovo11358\_f0 | denovo11462\_f0 | denovo11566\_f0 | denovo11930\_f0 | denovo11982\_f0 | denovo12086\_f0 |
| GNV120032 | Nemoria lixaria\* | denovo40\_f0 | denovo92\_f0 | denovo118\_f0 | denovo144\_f0 | denovo222\_f0 | denovo248\_f0 | denovo274\_f0 | denovo300\_f0 | denovo352\_f0 | denovo430\_f0 | denovo534\_f0 | denovo586\_f0 | denovo612\_f0 | denovo742\_f0 | denovo794\_f0 | denovo950\_f0 | denovo976\_f0 | denovo1054\_f0 | denovo1080\_f0 | denovo1158\_f0 | denovo1314\_f0 | denovo1496\_f0 | denovo1522\_f0 | denovo1652\_f0 | denovo1756\_f0 | denovo1782\_f0 | denovo1808\_f0 | denovo1964\_f0 | denovo2146\_f0 | denovo2198\_f0 | denovo2250\_f0 | denovo2276\_f0 | denovo2354\_f0 | denovo2458\_f0 | denovo2484\_f0 | denovo2510\_f0 | denovo2536\_f0 | denovo2588\_f0 | denovo2614\_f0 | denovo2666\_f0 | denovo2770\_f0 | denovo2796\_f0 | denovo2848\_f0 | denovo2874\_f0 | denovo2926\_f0 | denovo3004\_f0 | denovo3056\_f0 | denovo3160\_f0 | denovo3238\_f0 | denovo3264\_f0 | denovo3290\_f0 | denovo3316\_f0 | denovo3342\_f0 | denovo3446\_f0 | denovo3498\_f0 | denovo3602\_f0 | denovo3628\_f0 | denovo3680\_f0 | denovo3706\_f0 | denovo3732\_f0 | denovo3784\_f0 | denovo3862\_f0 | denovo3966\_f0 | denovo3992\_f0 | denovo4044\_f0 | denovo4070\_f0 | denovo4200\_f0 | denovo4304\_f0 | denovo4330\_f0 | denovo4356\_f0 | denovo4408\_f0 | denovo4460\_f0 | denovo4564\_f0 | denovo4642\_f0 | denovo4746\_f0 | denovo4772\_f0 | denovo4798\_f0 | denovo4850\_f0 | denovo4902\_f0 | denovo4928\_f0 | denovo4954\_f0 | denovo4980\_f0 | denovo5006\_f0 | denovo5214\_f0 | denovo5240\_f0 | denovo5266\_f0 | denovo5396\_f0 | denovo5448\_f0 | denovo5526\_f0 | denovo5604\_f0 | denovo5630\_f0 | denovo5656\_f0 | denovo5942\_f0 | denovo6020\_f0 | denovo6072\_f0 | denovo6124\_f0 | denovo6228\_f0 | denovo6254\_f0 | denovo6280\_f0 | denovo6592\_f0 | denovo6618\_f0 | denovo6696\_f0 | denovo6722\_f0 | denovo6774\_f0 | denovo6826\_f0 | denovo6878\_f0 | denovo7008\_f0 | denovo7086\_f0 | denovo7138\_f0 | denovo7164\_f0 | denovo7268\_f0 | denovo7346\_f0 | denovo7398\_f0 | denovo7424\_f0 | denovo7528\_f0 | denovo7554\_f0 | denovo7580\_f0 | denovo7632\_f0 | denovo7684\_f0 | denovo7710\_f0 | denovo7762\_f0 | denovo7814\_f0 | denovo7996\_f0 | denovo8048\_f0 | denovo8074\_f0 | denovo8230\_f0 | denovo8256\_f0 | denovo8282\_f0 | denovo8308\_f0 | denovo8412\_f0 | denovo8438\_f0 | denovo8464\_f0 | denovo8594\_f0 | denovo8646\_f0 | denovo8672\_f0 | denovo8698\_f0 | denovo8776\_f0 | denovo8828\_f0 | denovo8854\_f0 | denovo8906\_f0 | denovo8932\_f0 | denovo8958\_f0 | denovo8984\_f0 | denovo9010\_f0 | denovo9140\_f0 | denovo9166\_f0 | denovo9192\_f0 | denovo9322\_f0 | denovo9348\_f0 | denovo9374\_f0 | denovo9400\_f0 | denovo9452\_f0 | denovo9504\_f0 | denovo9556\_f0 | denovo9608\_f0 | denovo9686\_f0 | denovo9712\_f0 | denovo9738\_f0 | denovo9816\_f0 | denovo9842\_f0 | denovo9868\_f0 | denovo9894\_f0 | denovo9946\_f0 | denovo9972\_f0 | denovo9998\_f0 | denovo10076\_f0 | denovo10180\_f0 | denovo10206\_f0 | denovo10232\_f0 | denovo10284\_f0 | denovo10310\_f0 | denovo10362\_f0 | denovo10388\_f0 | denovo10414\_f0 | denovo10440\_f0 | denovo10492\_f0 | denovo10518\_f0 | denovo10544\_f0 | denovo10622\_f0 | denovo10674\_f0 | denovo10700\_f0 | denovo10778\_f0 | denovo10856\_f0 | denovo10882\_f0 | denovo10934\_f0 | denovo10986\_f0 | denovo11038\_f0 | denovo11090\_f0 | denovo11116\_f0 | denovo11168\_f0 | denovo11194\_f0 | denovo11246\_f0 | denovo11272\_f0 | denovo11324\_f0 | denovo11350\_f0 | denovo11454\_f0 | denovo11558\_f0 | denovo11922\_f0 | denovo11974\_f0 | denovo12078\_f0 |
| PXYLO | Plutella xylostella\* | denovo44\_f0 | denovo96\_f0 | denovo122\_f0 | denovo148\_f0 | denovo226\_f0 | denovo252\_f0 | denovo278\_f0 | denovo304\_f0 | denovo356\_f0 | denovo434\_f0 | denovo538\_f0 | denovo590\_f0 | denovo616\_f0 | denovo746\_f0 | denovo798\_f0 | denovo954\_f0 | denovo980\_f0 | denovo1058\_f0 | denovo1084\_f0 | denovo1162\_f0 | denovo1318\_f0 | denovo1500\_f0 | denovo1526\_f0 | denovo1656\_f0 | denovo1760\_f0 | denovo1786\_f0 | denovo1812\_f0 | denovo1968\_f0 | denovo2150\_f0 | denovo2202\_f0 | denovo2254\_f0 | denovo2280\_f0 | denovo2358\_f0 | denovo2462\_f0 | denovo2488\_f0 | denovo2514\_f0 | denovo2540\_f0 | denovo2592\_f0 | denovo2618\_f0 | denovo2670\_f0 | denovo2774\_f0 | denovo2800\_f0 | denovo2852\_f0 | denovo2878\_f0 | denovo2930\_f0 | denovo3008\_f0 | denovo3060\_f0 | denovo3164\_f0 | denovo3242\_f0 | denovo3268\_f0 | denovo3294\_f0 | denovo3320\_f0 | denovo3346\_f0 | denovo3450\_f0 | denovo3502\_f0 | denovo3606\_f0 | denovo3632\_f0 | denovo3684\_f0 | denovo3710\_f0 | denovo3736\_f0 | denovo3788\_f0 | denovo3866\_f0 | denovo3970\_f0 | denovo3996\_f0 | denovo4048\_f0 | denovo4074\_f0 | denovo4204\_f0 | denovo4308\_f0 | denovo4334\_f0 | denovo4360\_f0 | denovo4412\_f0 | denovo4464\_f0 | denovo4568\_f0 | denovo4646\_f0 | denovo4750\_f0 | denovo4776\_f0 | denovo4802\_f0 | denovo4854\_f0 | denovo4906\_f0 | denovo4932\_f0 | denovo4958\_f0 | denovo4984\_f0 | denovo5010\_f0 | denovo5218\_f0 | denovo5244\_f0 | denovo5270\_f0 | denovo5400\_f0 | denovo5452\_f0 | denovo5530\_f0 | denovo5608\_f0 | denovo5634\_f0 | denovo5660\_f0 | denovo5946\_f0 | denovo6024\_f0 | denovo6076\_f0 | denovo6128\_f0 | denovo6232\_f0 | denovo6258\_f0 | denovo6284\_f0 | denovo6596\_f0 | denovo6622\_f0 | denovo6700\_f0 | denovo6726\_f0 | denovo6778\_f0 | denovo6830\_f0 | denovo6882\_f0 | denovo7012\_f0 | denovo7090\_f0 | denovo7142\_f0 | denovo7168\_f0 | denovo7272\_f0 | denovo7350\_f0 | denovo7402\_f0 | denovo7428\_f0 | denovo7532\_f0 | denovo7558\_f0 | denovo7584\_f0 | denovo7636\_f0 | denovo7688\_f0 | denovo7714\_f0 | denovo7766\_f0 | denovo7818\_f0 | denovo8000\_f0 | denovo8052\_f0 | denovo8078\_f0 | denovo8234\_f0 | denovo8260\_f0 | denovo8286\_f0 | denovo8312\_f0 | denovo8416\_f0 | denovo8442\_f0 | denovo8468\_f0 | denovo8598\_f0 | denovo8650\_f0 | denovo8676\_f0 | denovo8702\_f0 | denovo8780\_f0 | denovo8832\_f0 | denovo8858\_f0 | denovo8910\_f0 | denovo8936\_f0 | denovo8962\_f0 | denovo8988\_f0 | denovo9014\_f0 | denovo9144\_f0 | denovo9170\_f0 | denovo9196\_f0 | denovo9326\_f0 | denovo9352\_f0 | denovo9378\_f0 | denovo9404\_f0 | denovo9456\_f0 | denovo9508\_f0 | denovo9560\_f0 | denovo9612\_f0 | denovo9690\_f0 | denovo9716\_f0 | denovo9742\_f0 | denovo9820\_f0 | denovo9846\_f0 | denovo9872\_f0 | denovo9898\_f0 | denovo9950\_f0 | denovo9976\_f0 | denovo10002\_f0 | denovo10080\_f0 | denovo10184\_f0 | denovo10210\_f0 | denovo10236\_f0 | denovo10288\_f0 | denovo10314\_f0 | denovo10366\_f0 | denovo10392\_f0 | denovo10418\_f0 | denovo10444\_f0 | denovo10496\_f0 | denovo10522\_f0 | denovo10548\_f0 | denovo10626\_f0 | denovo10678\_f0 | denovo10704\_f0 | denovo10782\_f0 | denovo10860\_f0 | denovo10886\_f0 | denovo10938\_f0 | denovo10990\_f0 | denovo11042\_f0 | denovo11094\_f0 | denovo11120\_f0 | denovo11172\_f0 | denovo11198\_f0 | denovo11250\_f0 | denovo11276\_f0 | denovo11328\_f0 | denovo11354\_f0 | denovo11458\_f0 | denovo11562\_f0 | denovo11926\_f0 | denovo11978\_f0 | denovo12082\_f0 |
| FG120079 | Zeuzerodes maculata\* | denovo37\_f0 | denovo89\_f0 | denovo115\_f0 | denovo141\_f0 | denovo219\_f0 | denovo245\_f0 | denovo271\_f0 | denovo297\_f0 | denovo349\_f0 | denovo427\_f0 | denovo531\_f0 | denovo583\_f0 | denovo609\_f0 | denovo739\_f0 | denovo791\_f0 | denovo947\_f0 | denovo973\_f0 | denovo1051\_f0 | denovo1077\_f0 | denovo1155\_f0 | denovo1311\_f0 | denovo1493\_f0 | denovo1519\_f0 | denovo1649\_f0 | denovo1753\_f0 | denovo1779\_f0 | denovo1805\_f0 | denovo1961\_f0 | denovo2143\_f0 | denovo2195\_f0 | denovo2247\_f0 | denovo2273\_f0 | denovo2351\_f0 | denovo2455\_f0 | denovo2481\_f0 | denovo2507\_f0 | denovo2533\_f0 | denovo2585\_f0 | denovo2611\_f0 | denovo2663\_f0 | denovo2767\_f0 | denovo2793\_f0 | denovo2845\_f0 | denovo2871\_f0 | denovo2923\_f0 | denovo3001\_f0 | denovo3053\_f0 | denovo3157\_f0 | denovo3235\_f0 | denovo3261\_f0 | denovo3287\_f0 | denovo3313\_f0 | denovo3339\_f0 | denovo3443\_f0 | denovo3495\_f0 | denovo3599\_f0 | denovo3625\_f0 | denovo3677\_f0 | denovo3703\_f0 | denovo3729\_f0 | denovo3781\_f0 | denovo3859\_f0 | denovo3963\_f0 | denovo3989\_f0 | denovo4041\_f0 | denovo4067\_f0 | denovo4197\_f0 | denovo4301\_f0 | denovo4327\_f0 | denovo4353\_f0 | denovo4405\_f0 | denovo4457\_f0 | denovo4561\_f0 | denovo4639\_f0 | denovo4743\_f0 | denovo4769\_f0 | denovo4795\_f0 | denovo4847\_f0 | denovo4899\_f0 | denovo4925\_f0 | denovo4951\_f0 | denovo4977\_f0 | denovo5003\_f0 | denovo5211\_f0 | denovo5237\_f0 | denovo5263\_f0 | denovo5393\_f0 | denovo5445\_f0 | denovo5523\_f0 | denovo5601\_f0 | denovo5627\_f0 | denovo5653\_f0 | denovo5939\_f0 | denovo6017\_f0 | denovo6069\_f0 | denovo6121\_f0 | denovo6225\_f0 | denovo6251\_f0 | denovo6277\_f0 | denovo6589\_f0 | denovo6615\_f0 | denovo6693\_f0 | denovo6719\_f0 | denovo6771\_f0 | denovo6823\_f0 | denovo6875\_f0 | denovo7005\_f0 | denovo7083\_f0 | denovo7135\_f0 | denovo7161\_f0 | denovo7265\_f0 | denovo7343\_f0 | denovo7395\_f0 | denovo7421\_f0 | denovo7525\_f0 | denovo7551\_f0 | denovo7577\_f0 | denovo7629\_f0 | denovo7681\_f0 | denovo7707\_f0 | denovo7759\_f0 | denovo7811\_f0 | denovo7993\_f0 | denovo8045\_f0 | denovo8071\_f0 | denovo8227\_f0 | denovo8253\_f0 | denovo8279\_f0 | denovo8305\_f0 | denovo8409\_f0 | denovo8435\_f0 | denovo8461\_f0 | denovo8591\_f0 | denovo8643\_f0 | denovo8669\_f0 | denovo8695\_f0 | denovo8773\_f0 | denovo8825\_f0 | denovo8851\_f0 | denovo8903\_f0 | denovo8929\_f0 | denovo8955\_f0 | denovo8981\_f0 | denovo9007\_f0 | denovo9137\_f0 | denovo9163\_f0 | denovo9189\_f0 | denovo9319\_f0 | denovo9345\_f0 | denovo9371\_f0 | denovo9397\_f0 | denovo9449\_f0 | denovo9501\_f0 | denovo9553\_f0 | denovo9605\_f0 | denovo9683\_f0 | denovo9709\_f0 | denovo9735\_f0 | denovo9813\_f0 | denovo9839\_f0 | denovo9865\_f0 | denovo9891\_f0 | denovo9943\_f0 | denovo9969\_f0 | denovo9995\_f0 | denovo10073\_f0 | denovo10177\_f0 | denovo10203\_f0 | denovo10229\_f0 | denovo10281\_f0 | denovo10307\_f0 | denovo10359\_f0 | denovo10385\_f0 | denovo10411\_f0 | denovo10437\_f0 | denovo10489\_f0 | denovo10515\_f0 | denovo10541\_f0 | denovo10619\_f0 | denovo10671\_f0 | denovo10697\_f0 | denovo10775\_f0 | denovo10853\_f0 | denovo10879\_f0 | denovo10931\_f0 | denovo10983\_f0 | denovo11035\_f0 | denovo11087\_f0 | denovo11113\_f0 | denovo11165\_f0 | denovo11191\_f0 | denovo11243\_f0 | denovo11269\_f0 | denovo11321\_f0 | denovo11347\_f0 | denovo11451\_f0 | denovo11555\_f0 | denovo11919\_f0 | denovo11971\_f0 | denovo12075\_f0 |
| SRR850324 | Papilio glaucus | denovo47\_f0 | denovo99\_f0 | denovo125\_f0 | denovo151\_f0 | denovo229\_f0 | denovo255\_f0 | denovo281\_f0 | denovo307\_f0 | denovo359\_f0 | denovo437\_f0 | denovo541\_f0 | denovo593\_f0 | denovo619\_f0 | denovo749\_f0 | denovo801\_f0 | denovo957\_f0 | denovo983\_f0 | denovo1061\_f0 | denovo1087\_f0 | denovo1165\_f0 | denovo1321\_f0 | denovo1503\_f0 | denovo1529\_f0 | denovo1659\_f0 | denovo1763\_f0 | denovo1789\_f0 | denovo1815\_f0 | denovo1971\_f0 | denovo2153\_f0 | denovo2205\_f0 | denovo2257\_f0 | denovo2283\_f0 | denovo2361\_f0 | denovo2465\_f0 | denovo2491\_f0 | denovo2517\_f0 | denovo2543\_f0 | denovo2595\_f0 | denovo2621\_f0 | denovo2673\_f0 | denovo2777\_f0 | denovo2803\_f0 | denovo2855\_f0 | denovo2881\_f0 | denovo2933\_f0 | denovo3011\_f0 | denovo3063\_f0 | denovo3167\_f0 | denovo3245\_f0 | denovo3271\_f0 | denovo3297\_f0 | denovo3323\_f0 | denovo3349\_f0 | denovo3453\_f0 | denovo3505\_f0 | denovo3609\_f0 | denovo3635\_f0 | denovo3687\_f0 | denovo3713\_f0 | denovo3739\_f0 | denovo3791\_f0 | denovo3869\_f0 | denovo3973\_f0 | denovo3999\_f0 | denovo4051\_f0 | denovo4077\_f0 | denovo4207\_f0 | denovo4311\_f0 | denovo4337\_f0 | denovo4363\_f0 | denovo4415\_f0 | denovo4467\_f0 | denovo4571\_f0 | denovo4649\_f0 | denovo4753\_f0 | denovo4779\_f0 | denovo4805\_f0 | denovo4857\_f0 | denovo4909\_f0 | denovo4935\_f0 | denovo4961\_f0 | denovo4987\_f0 | denovo5013\_f0 | denovo5221\_f0 | denovo5247\_f0 | denovo5273\_f0 | denovo5403\_f0 | denovo5455\_f0 | denovo5533\_f0 | denovo5611\_f0 | denovo5637\_f0 | denovo5663\_f0 | denovo5949\_f0 | denovo6027\_f0 | denovo6079\_f0 | denovo6131\_f0 | denovo6235\_f0 | denovo6261\_f0 | denovo6287\_f0 | denovo6599\_f0 | denovo6625\_f0 | denovo6703\_f0 | denovo6729\_f0 | denovo6781\_f0 | denovo6833\_f0 | denovo6885\_f0 | denovo7015\_f0 | denovo7093\_f0 | denovo7145\_f0 | denovo7171\_f0 | denovo7275\_f0 | denovo7353\_f0 | denovo7405\_f0 | denovo7431\_f0 | denovo7535\_f0 | denovo7561\_f0 | denovo7587\_f0 | denovo7639\_f0 | denovo7691\_f0 | denovo7717\_f0 | denovo7769\_f0 | denovo7821\_f0 | denovo8003\_f0 | denovo8055\_f0 | denovo8081\_f0 | denovo8237\_f0 | denovo8263\_f0 | denovo8289\_f0 | denovo8315\_f0 | denovo8419\_f0 | denovo8445\_f0 | denovo8471\_f0 | denovo8601\_f0 | denovo8653\_f0 | denovo8679\_f0 | denovo8705\_f0 | denovo8783\_f0 | denovo8835\_f0 | denovo8861\_f0 | denovo8913\_f0 | denovo8939\_f0 | denovo8965\_f0 | denovo8991\_f0 | denovo9017\_f0 | denovo9147\_f0 | denovo9173\_f0 | denovo9199\_f0 | denovo9329\_f0 | denovo9355\_f0 | denovo9381\_f0 | denovo9407\_f0 | denovo9459\_f0 | denovo9511\_f0 | denovo9563\_f0 | denovo9615\_f0 | denovo9693\_f0 | denovo9719\_f0 | denovo9745\_f0 | denovo9823\_f0 | denovo9849\_f0 | denovo9875\_f0 | denovo9901\_f0 | denovo9953\_f0 | denovo9979\_f0 | denovo10005\_f0 | denovo10083\_f0 | denovo10187\_f0 | denovo10213\_f0 | denovo10239\_f0 | denovo10291\_f0 | denovo10317\_f0 | denovo10369\_f0 | denovo10395\_f0 | denovo10421\_f0 | denovo10447\_f0 | denovo10499\_f0 | denovo10525\_f0 | denovo10551\_f0 | denovo10629\_f0 | denovo10681\_f0 | denovo10707\_f0 | denovo10785\_f0 | denovo10863\_f0 | denovo10889\_f0 | denovo10941\_f0 | denovo10993\_f0 | denovo11045\_f0 | denovo11097\_f0 | denovo11123\_f0 | denovo11175\_f0 | denovo11201\_f0 | denovo11253\_f0 | denovo11279\_f0 | denovo11331\_f0 | denovo11357\_f0 | denovo11461\_f0 | denovo11565\_f0 | denovo11929\_f0 | denovo11981\_f0 | denovo12085\_f0 |
| FG120055B | Nothus lunus\* | denovo33\_f0 | denovo85\_f0 | denovo111\_f0 | denovo137\_f0 | denovo215\_f0 | denovo241\_f0 | denovo267\_f0 | denovo293\_f0 | denovo345\_f0 | denovo423\_f0 | denovo527\_f0 | denovo579\_f0 | denovo605\_f0 | denovo735\_f0 | denovo787\_f0 | denovo943\_f0 | denovo969\_f0 | denovo1047\_f0 | denovo1073\_f0 | denovo1151\_f0 | denovo1307\_f0 | denovo1489\_f0 | denovo1515\_f0 | denovo1645\_f0 | denovo1749\_f0 | denovo1775\_f0 | denovo1801\_f0 | denovo1957\_f0 | denovo2139\_f0 | denovo2191\_f0 | denovo2243\_f0 | denovo2269\_f0 | denovo2347\_f0 | denovo2451\_f0 | denovo2477\_f0 | denovo2503\_f0 | denovo2529\_f0 | denovo2581\_f0 | denovo2607\_f0 | denovo2659\_f0 | denovo2763\_f0 | denovo2789\_f0 | denovo2841\_f0 | denovo2867\_f0 | denovo2919\_f0 | denovo2997\_f0 | denovo3049\_f0 | denovo3153\_f0 | denovo3231\_f0 | denovo3257\_f0 | denovo3283\_f0 | denovo3309\_f0 | denovo3335\_f0 | denovo3439\_f0 | denovo3491\_f0 | denovo3595\_f0 | denovo3621\_f0 | denovo3673\_f0 | denovo3699\_f0 | denovo3725\_f0 | denovo3777\_f0 | denovo3855\_f0 | denovo3959\_f0 | denovo3985\_f0 | denovo4037\_f0 | denovo4063\_f0 | denovo4193\_f0 | denovo4297\_f0 | denovo4323\_f0 | denovo4349\_f0 | denovo4401\_f0 | denovo4453\_f0 | denovo4557\_f0 | denovo4635\_f0 | denovo4739\_f0 | denovo4765\_f0 | denovo4791\_f0 | denovo4843\_f0 | denovo4895\_f0 | denovo4921\_f0 | denovo4947\_f0 | denovo4973\_f0 | denovo4999\_f0 | denovo5207\_f0 | denovo5233\_f0 | denovo5259\_f0 | denovo5389\_f0 | denovo5441\_f0 | denovo5519\_f0 | denovo5597\_f0 | denovo5623\_f0 | denovo5649\_f0 | denovo5935\_f0 | denovo6013\_f0 | denovo6065\_f0 | denovo6117\_f0 | denovo6221\_f0 | denovo6247\_f0 | denovo6273\_f0 | denovo6585\_f0 | denovo6611\_f0 | denovo6689\_f0 | denovo6715\_f0 | denovo6767\_f0 | denovo6819\_f0 | denovo6871\_f0 | denovo7001\_f0 | denovo7079\_f0 | denovo7131\_f0 | denovo7157\_f0 | denovo7261\_f0 | denovo7339\_f0 | denovo7391\_f0 | denovo7417\_f0 | denovo7521\_f0 | denovo7547\_f0 | denovo7573\_f0 | denovo7625\_f0 | denovo7677\_f0 | denovo7703\_f0 | denovo7755\_f0 | denovo7807\_f0 | denovo7989\_f0 | denovo8041\_f0 | denovo8067\_f0 | denovo8223\_f0 | denovo8249\_f0 | denovo8275\_f0 | denovo8301\_f0 | denovo8405\_f0 | denovo8431\_f0 | denovo8457\_f0 | denovo8587\_f0 | denovo8639\_f0 | denovo8665\_f0 | denovo8691\_f0 | denovo8769\_f0 | denovo8821\_f0 | denovo8847\_f0 | denovo8899\_f0 | denovo8925\_f0 | denovo8951\_f0 | denovo8977\_f0 | denovo9003\_f0 | denovo9133\_f0 | denovo9159\_f0 | denovo9185\_f0 | denovo9315\_f0 | denovo9341\_f0 | denovo9367\_f0 | denovo9393\_f0 | denovo9445\_f0 | denovo9497\_f0 | denovo9549\_f0 | denovo9601\_f0 | denovo9679\_f0 | denovo9705\_f0 | denovo9731\_f0 | denovo9809\_f0 | denovo9835\_f0 | denovo9861\_f0 | denovo9887\_f0 | denovo9939\_f0 | denovo9965\_f0 | denovo9991\_f0 | denovo10069\_f0 | denovo10173\_f0 | denovo10199\_f0 | denovo10225\_f0 | denovo10277\_f0 | denovo10303\_f0 | denovo10355\_f0 | denovo10381\_f0 | denovo10407\_f0 | denovo10433\_f0 | denovo10485\_f0 | denovo10511\_f0 | denovo10537\_f0 | denovo10615\_f0 | denovo10667\_f0 | denovo10693\_f0 | denovo10771\_f0 | denovo10849\_f0 | denovo10875\_f0 | denovo10927\_f0 | denovo10979\_f0 | denovo11031\_f0 | denovo11083\_f0 | denovo11109\_f0 | denovo11161\_f0 | denovo11187\_f0 | denovo11239\_f0 | denovo11265\_f0 | denovo11317\_f0 | denovo11343\_f0 | denovo11447\_f0 | denovo11551\_f0 | denovo11915\_f0 | denovo11967\_f0 | denovo12071\_f0 |
| FG120122 | Macrosoma sp.\* | denovo38\_f0 | denovo90\_f0 | denovo116\_f0 | denovo142\_f0 | denovo220\_f0 | denovo246\_f0 | denovo272\_f0 | denovo298\_f0 | denovo350\_f0 | denovo428\_f0 | denovo532\_f0 | denovo584\_f0 | denovo610\_f0 | denovo740\_f0 | denovo792\_f0 | denovo948\_f0 | denovo974\_f0 | denovo1052\_f0 | denovo1078\_f0 | denovo1156\_f0 | denovo1312\_f0 | denovo1494\_f0 | denovo1520\_f0 | denovo1650\_f0 | denovo1754\_f0 | denovo1780\_f0 | denovo1806\_f0 | denovo1962\_f0 | denovo2144\_f0 | denovo2196\_f0 | denovo2248\_f0 | denovo2274\_f0 | denovo2352\_f0 | denovo2456\_f0 | denovo2482\_f0 | denovo2508\_f0 | denovo2534\_f0 | denovo2586\_f0 | denovo2612\_f0 | denovo2664\_f0 | denovo2768\_f0 | denovo2794\_f0 | denovo2846\_f0 | denovo2872\_f0 | denovo2924\_f0 | denovo3002\_f0 | denovo3054\_f0 | denovo3158\_f0 | denovo3236\_f0 | denovo3262\_f0 | denovo3288\_f0 | denovo3314\_f0 | denovo3340\_f0 | denovo3444\_f0 | denovo3496\_f0 | denovo3600\_f0 | denovo3626\_f0 | denovo3678\_f0 | denovo3704\_f0 | denovo3730\_f0 | denovo3782\_f0 | denovo3860\_f0 | denovo3964\_f0 | denovo3990\_f0 | denovo4042\_f0 | denovo4068\_f0 | denovo4198\_f0 | denovo4302\_f0 | denovo4328\_f0 | denovo4354\_f0 | denovo4406\_f0 | denovo4458\_f0 | denovo4562\_f0 | denovo4640\_f0 | denovo4744\_f0 | denovo4770\_f0 | denovo4796\_f0 | denovo4848\_f0 | denovo4900\_f0 | denovo4926\_f0 | denovo4952\_f0 | denovo4978\_f0 | denovo5004\_f0 | denovo5212\_f0 | denovo5238\_f0 | denovo5264\_f0 | denovo5394\_f0 | denovo5446\_f0 | denovo5524\_f0 | denovo5602\_f0 | denovo5628\_f0 | denovo5654\_f0 | denovo5940\_f0 | denovo6018\_f0 | denovo6070\_f0 | denovo6122\_f0 | denovo6226\_f0 | denovo6252\_f0 | denovo6278\_f0 | denovo6590\_f0 | denovo6616\_f0 | denovo6694\_f0 | denovo6720\_f0 | denovo6772\_f0 | denovo6824\_f0 | denovo6876\_f0 | denovo7006\_f0 | denovo7084\_f0 | denovo7136\_f0 | denovo7162\_f0 | denovo7266\_f0 | denovo7344\_f0 | denovo7396\_f0 | denovo7422\_f0 | denovo7526\_f0 | denovo7552\_f0 | denovo7578\_f0 | denovo7630\_f0 | denovo7682\_f0 | denovo7708\_f0 | denovo7760\_f0 | denovo7812\_f0 | denovo7994\_f0 | denovo8046\_f0 | denovo8072\_f0 | denovo8228\_f0 | denovo8254\_f0 | denovo8280\_f0 | denovo8306\_f0 | denovo8410\_f0 | denovo8436\_f0 | denovo8462\_f0 | denovo8592\_f0 | denovo8644\_f0 | denovo8670\_f0 | denovo8696\_f0 | denovo8774\_f0 | denovo8826\_f0 | denovo8852\_f0 | denovo8904\_f0 | denovo8930\_f0 | denovo8956\_f0 | denovo8982\_f0 | denovo9008\_f0 | denovo9138\_f0 | denovo9164\_f0 | denovo9190\_f0 | denovo9320\_f0 | denovo9346\_f0 | denovo9372\_f0 | denovo9398\_f0 | denovo9450\_f0 | denovo9502\_f0 | denovo9554\_f0 | denovo9606\_f0 | denovo9684\_f0 | denovo9710\_f0 | denovo9736\_f0 | denovo9814\_f0 | denovo9840\_f0 | denovo9866\_f0 | denovo9892\_f0 | denovo9944\_f0 | denovo9970\_f0 | denovo9996\_f0 | denovo10074\_f0 | denovo10178\_f0 | denovo10204\_f0 | denovo10230\_f0 | denovo10282\_f0 | denovo10308\_f0 | denovo10360\_f0 | denovo10386\_f0 | denovo10412\_f0 | denovo10438\_f0 | denovo10490\_f0 | denovo10516\_f0 | denovo10542\_f0 | denovo10620\_f0 | denovo10672\_f0 | denovo10698\_f0 | denovo10776\_f0 | denovo10854\_f0 | denovo10880\_f0 | denovo10932\_f0 | denovo10984\_f0 | denovo11036\_f0 | denovo11088\_f0 | denovo11114\_f0 | denovo11166\_f0 | denovo11192\_f0 | denovo11244\_f0 | denovo11270\_f0 | denovo11322\_f0 | denovo11348\_f0 | denovo11452\_f0 | denovo11556\_f0 | denovo11920\_f0 | denovo11972\_f0 | denovo12076\_f0 |
| FG120024 | Megalopyge tharops\* | denovo30\_f0 | denovo82\_f0 | denovo108\_f0 | denovo134\_f0 | denovo212\_f0 | denovo238\_f0 | denovo264\_f0 | denovo290\_f0 | denovo342\_f0 | denovo420\_f0 | denovo524\_f0 | denovo576\_f0 | denovo602\_f0 | denovo732\_f0 | denovo784\_f0 | denovo940\_f0 | denovo966\_f0 | denovo1044\_f0 | denovo1070\_f0 | denovo1148\_f0 | denovo1304\_f0 | denovo1486\_f0 | denovo1512\_f0 | denovo1642\_f0 | denovo1746\_f0 | denovo1772\_f0 | denovo1798\_f0 | denovo1954\_f0 | denovo2136\_f0 | denovo2188\_f0 | denovo2240\_f0 | denovo2266\_f0 | denovo2344\_f0 | denovo2448\_f0 | denovo2474\_f0 | denovo2500\_f0 | denovo2526\_f0 | denovo2578\_f0 | denovo2604\_f0 | denovo2656\_f0 | denovo2760\_f0 | denovo2786\_f0 | denovo2838\_f0 | denovo2864\_f0 | denovo2916\_f0 | denovo2994\_f0 | denovo3046\_f0 | denovo3150\_f0 | denovo3228\_f0 | denovo3254\_f0 | denovo3280\_f0 | denovo3306\_f0 | denovo3332\_f0 | denovo3436\_f0 | denovo3488\_f0 | denovo3592\_f0 | denovo3618\_f0 | denovo3670\_f0 | denovo3696\_f0 | denovo3722\_f0 | denovo3774\_f0 | denovo3852\_f0 | denovo3956\_f0 | denovo3982\_f0 | denovo4034\_f0 | denovo4060\_f0 | denovo4190\_f0 | denovo4294\_f0 | denovo4320\_f0 | denovo4346\_f0 | denovo4398\_f0 | denovo4450\_f0 | denovo4554\_f0 | denovo4632\_f0 | denovo4736\_f0 | denovo4762\_f0 | denovo4788\_f0 | denovo4840\_f0 | denovo4892\_f0 | denovo4918\_f0 | denovo4944\_f0 | denovo4970\_f0 | denovo4996\_f0 | denovo5204\_f0 | denovo5230\_f0 | denovo5256\_f0 | denovo5386\_f0 | denovo5438\_f0 | denovo5516\_f0 | denovo5594\_f0 | denovo5620\_f0 | denovo5646\_f0 | denovo5932\_f0 | denovo6010\_f0 | denovo6062\_f0 | denovo6114\_f0 | denovo6218\_f0 | denovo6244\_f0 | denovo6270\_f0 | denovo6582\_f0 | denovo6608\_f0 | denovo6686\_f0 | denovo6712\_f0 | denovo6764\_f0 | denovo6816\_f0 | denovo6868\_f0 | denovo6998\_f0 | denovo7076\_f0 | denovo7128\_f0 | denovo7154\_f0 | denovo7258\_f0 | denovo7336\_f0 | denovo7388\_f0 | denovo7414\_f0 | denovo7518\_f0 | denovo7544\_f0 | denovo7570\_f0 | denovo7622\_f0 | denovo7674\_f0 | denovo7700\_f0 | denovo7752\_f0 | denovo7804\_f0 | denovo7986\_f0 | denovo8038\_f0 | denovo8064\_f0 | denovo8220\_f0 | denovo8246\_f0 | denovo8272\_f0 | denovo8298\_f0 | denovo8402\_f0 | denovo8428\_f0 | denovo8454\_f0 | denovo8584\_f0 | denovo8636\_f0 | denovo8662\_f0 | denovo8688\_f0 | denovo8766\_f0 | denovo8818\_f0 | denovo8844\_f0 | denovo8896\_f0 | denovo8922\_f0 | denovo8948\_f0 | denovo8974\_f0 | denovo9000\_f0 | denovo9130\_f0 | denovo9156\_f0 | denovo9182\_f0 | denovo9312\_f0 | denovo9338\_f0 | denovo9364\_f0 | denovo9390\_f0 | denovo9442\_f0 | denovo9494\_f0 | denovo9546\_f0 | denovo9598\_f0 | denovo9676\_f0 | denovo9702\_f0 | denovo9728\_f0 | denovo9806\_f0 | denovo9832\_f0 | denovo9858\_f0 | denovo9884\_f0 | denovo9936\_f0 | denovo9962\_f0 | denovo9988\_f0 | denovo10066\_f0 | denovo10170\_f0 | denovo10196\_f0 | denovo10222\_f0 | denovo10274\_f0 | denovo10300\_f0 | denovo10352\_f0 | denovo10378\_f0 | denovo10404\_f0 | denovo10430\_f0 | denovo10482\_f0 | denovo10508\_f0 | denovo10534\_f0 | denovo10612\_f0 | denovo10664\_f0 | denovo10690\_f0 | denovo10768\_f0 | denovo10846\_f0 | denovo10872\_f0 | denovo10924\_f0 | denovo10976\_f0 | denovo11028\_f0 | denovo11080\_f0 | denovo11106\_f0 | denovo11158\_f0 | denovo11184\_f0 | denovo11236\_f0 | denovo11262\_f0 | denovo11314\_f0 | denovo11340\_f0 | denovo11444\_f0 | denovo11548\_f0 | denovo11912\_f0 | denovo11964\_f0 | denovo12068\_f0 |
| FG120022 | Morpheis mathani\* | denovo29\_f0 | denovo81\_f0 | denovo107\_f0 | denovo133\_f0 | denovo211\_f0 | denovo237\_f0 | denovo263\_f0 | denovo289\_f0 | denovo341\_f0 | denovo419\_f0 | denovo523\_f0 | denovo575\_f0 | denovo601\_f0 | denovo731\_f0 | denovo783\_f0 | denovo939\_f0 | denovo965\_f0 | denovo1043\_f0 | denovo1069\_f0 | denovo1147\_f0 | denovo1303\_f0 | denovo1485\_f0 | denovo1511\_f0 | denovo1641\_f0 | denovo1745\_f0 | denovo1771\_f0 | denovo1797\_f0 | denovo1953\_f0 | denovo2135\_f0 | denovo2187\_f0 | denovo2239\_f0 | denovo2265\_f0 | denovo2343\_f0 | denovo2447\_f0 | denovo2473\_f0 | denovo2499\_f0 | denovo2525\_f0 | denovo2577\_f0 | denovo2603\_f0 | denovo2655\_f0 | denovo2759\_f0 | denovo2785\_f0 | denovo2837\_f0 | denovo2863\_f0 | denovo2915\_f0 | denovo2993\_f0 | denovo3045\_f0 | denovo3149\_f0 | denovo3227\_f0 | denovo3253\_f0 | denovo3279\_f0 | denovo3305\_f0 | denovo3331\_f0 | denovo3435\_f0 | denovo3487\_f0 | denovo3591\_f0 | denovo3617\_f0 | denovo3669\_f0 | denovo3695\_f0 | denovo3721\_f0 | denovo3773\_f0 | denovo3851\_f0 | denovo3955\_f0 | denovo3981\_f0 | denovo4033\_f0 | denovo4059\_f0 | denovo4189\_f0 | denovo4293\_f0 | denovo4319\_f0 | denovo4345\_f0 | denovo4397\_f0 | denovo4449\_f0 | denovo4553\_f0 | denovo4631\_f0 | denovo4735\_f0 | denovo4761\_f0 | denovo4787\_f0 | denovo4839\_f0 | denovo4891\_f0 | denovo4917\_f0 | denovo4943\_f0 | denovo4969\_f0 | denovo4995\_f0 | denovo5203\_f0 | denovo5229\_f0 | denovo5255\_f0 | denovo5385\_f0 | denovo5437\_f0 | denovo5515\_f0 | denovo5593\_f0 | denovo5619\_f0 | denovo5645\_f0 | denovo5931\_f0 | denovo6009\_f0 | denovo6061\_f0 | denovo6113\_f0 | denovo6217\_f0 | denovo6243\_f0 | denovo6269\_f0 | denovo6581\_f0 | denovo6607\_f0 | denovo6685\_f0 | denovo6711\_f0 | denovo6763\_f0 | denovo6815\_f0 | denovo6867\_f0 | denovo6997\_f0 | denovo7075\_f0 | denovo7127\_f0 | denovo7153\_f0 | denovo7257\_f0 | denovo7335\_f0 | denovo7387\_f0 | denovo7413\_f0 | denovo7517\_f0 | denovo7543\_f0 | denovo7569\_f0 | denovo7621\_f0 | denovo7673\_f0 | denovo7699\_f0 | denovo7751\_f0 | denovo7803\_f0 | denovo7985\_f0 | denovo8037\_f0 | denovo8063\_f0 | denovo8219\_f0 | denovo8245\_f0 | denovo8271\_f0 | denovo8297\_f0 | denovo8401\_f0 | denovo8427\_f0 | denovo8453\_f0 | denovo8583\_f0 | denovo8635\_f0 | denovo8661\_f0 | denovo8687\_f0 | denovo8765\_f0 | denovo8817\_f0 | denovo8843\_f0 | denovo8895\_f0 | denovo8921\_f0 | denovo8947\_f0 | denovo8973\_f0 | denovo8999\_f0 | denovo9129\_f0 | denovo9155\_f0 | denovo9181\_f0 | denovo9311\_f0 | denovo9337\_f0 | denovo9363\_f0 | denovo9389\_f0 | denovo9441\_f0 | denovo9493\_f0 | denovo9545\_f0 | denovo9597\_f0 | denovo9675\_f0 | denovo9701\_f0 | denovo9727\_f0 | denovo9805\_f0 | denovo9831\_f0 | denovo9857\_f0 | denovo9883\_f0 | denovo9935\_f0 | denovo9961\_f0 | denovo9987\_f0 | denovo10065\_f0 | denovo10169\_f0 | denovo10195\_f0 | denovo10221\_f0 | denovo10273\_f0 | denovo10299\_f0 | denovo10351\_f0 | denovo10377\_f0 | denovo10403\_f0 | denovo10429\_f0 | denovo10481\_f0 | denovo10507\_f0 | denovo10533\_f0 | denovo10611\_f0 | denovo10663\_f0 | denovo10689\_f0 | denovo10767\_f0 | denovo10845\_f0 | denovo10871\_f0 | denovo10923\_f0 | denovo10975\_f0 | denovo11027\_f0 | denovo11079\_f0 | denovo11105\_f0 | denovo11157\_f0 | denovo11183\_f0 | denovo11235\_f0 | denovo11261\_f0 | denovo11313\_f0 | denovo11339\_f0 | denovo11443\_f0 | denovo11547\_f0 | denovo11911\_f0 | denovo11963\_f0 | denovo12067\_f0 |
| GNV139000 | Megathymus yuccae\* | denovo42\_f0 | denovo94\_f0 | denovo120\_f0 | denovo146\_f0 | denovo224\_f0 | denovo250\_f0 | denovo276\_f0 | denovo302\_f0 | denovo354\_f0 | denovo432\_f0 | denovo536\_f0 | denovo588\_f0 | denovo614\_f0 | denovo744\_f0 | denovo796\_f0 | denovo952\_f0 | denovo978\_f0 | denovo1056\_f0 | denovo1082\_f0 | denovo1160\_f0 | denovo1316\_f0 | denovo1498\_f0 | denovo1524\_f0 | denovo1654\_f0 | denovo1758\_f0 | denovo1784\_f0 | denovo1810\_f0 | denovo1966\_f0 | denovo2148\_f0 | denovo2200\_f0 | denovo2252\_f0 | denovo2278\_f0 | denovo2356\_f0 | denovo2460\_f0 | denovo2486\_f0 | denovo2512\_f0 | denovo2538\_f0 | denovo2590\_f0 | denovo2616\_f0 | denovo2668\_f0 | denovo2772\_f0 | denovo2798\_f0 | denovo2850\_f0 | denovo2876\_f0 | denovo2928\_f0 | denovo3006\_f0 | denovo3058\_f0 | denovo3162\_f0 | denovo3240\_f0 | denovo3266\_f0 | denovo3292\_f0 | denovo3318\_f0 | denovo3344\_f0 | denovo3448\_f0 | denovo3500\_f0 | denovo3604\_f0 | denovo3630\_f0 | denovo3682\_f0 | denovo3708\_f0 | denovo3734\_f0 | denovo3786\_f0 | denovo3864\_f0 | denovo3968\_f0 | denovo3994\_f0 | denovo4046\_f0 | denovo4072\_f0 | denovo4202\_f0 | denovo4306\_f0 | denovo4332\_f0 | denovo4358\_f0 | denovo4410\_f0 | denovo4462\_f0 | denovo4566\_f0 | denovo4644\_f0 | denovo4748\_f0 | denovo4774\_f0 | denovo4800\_f0 | denovo4852\_f0 | denovo4904\_f0 | denovo4930\_f0 | denovo4956\_f0 | denovo4982\_f0 | denovo5008\_f0 | denovo5216\_f0 | denovo5242\_f0 | denovo5268\_f0 | denovo5398\_f0 | denovo5450\_f0 | denovo5528\_f0 | denovo5606\_f0 | denovo5632\_f0 | denovo5658\_f0 | denovo5944\_f0 | denovo6022\_f0 | denovo6074\_f0 | denovo6126\_f0 | denovo6230\_f0 | denovo6256\_f0 | denovo6282\_f0 | denovo6594\_f0 | denovo6620\_f0 | denovo6698\_f0 | denovo6724\_f0 | denovo6776\_f0 | denovo6828\_f0 | denovo6880\_f0 | denovo7010\_f0 | denovo7088\_f0 | denovo7140\_f0 | denovo7166\_f0 | denovo7270\_f0 | denovo7348\_f0 | denovo7400\_f0 | denovo7426\_f0 | denovo7530\_f0 | denovo7556\_f0 | denovo7582\_f0 | denovo7634\_f0 | denovo7686\_f0 | denovo7712\_f0 | denovo7764\_f0 | denovo7816\_f0 | denovo7998\_f0 | denovo8050\_f0 | denovo8076\_f0 | denovo8232\_f0 | denovo8258\_f0 | denovo8284\_f0 | denovo8310\_f0 | denovo8414\_f0 | denovo8440\_f0 | denovo8466\_f0 | denovo8596\_f0 | denovo8648\_f0 | denovo8674\_f0 | denovo8700\_f0 | denovo8778\_f0 | denovo8830\_f0 | denovo8856\_f0 | denovo8908\_f0 | denovo8934\_f0 | denovo8960\_f0 | denovo8986\_f0 | denovo9012\_f0 | denovo9142\_f0 | denovo9168\_f0 | denovo9194\_f0 | denovo9324\_f0 | denovo9350\_f0 | denovo9376\_f0 | denovo9402\_f0 | denovo9454\_f0 | denovo9506\_f0 | denovo9558\_f0 | denovo9610\_f0 | denovo9688\_f0 | denovo9714\_f0 | denovo9740\_f0 | denovo9818\_f0 | denovo9844\_f0 | denovo9870\_f0 | denovo9896\_f0 | denovo9948\_f0 | denovo9974\_f0 | denovo10000\_f0 | denovo10078\_f0 | denovo10182\_f0 | denovo10208\_f0 | denovo10234\_f0 | denovo10286\_f0 | denovo10312\_f0 | denovo10364\_f0 | denovo10390\_f0 | denovo10416\_f0 | denovo10442\_f0 | denovo10494\_f0 | denovo10520\_f0 | denovo10546\_f0 | denovo10624\_f0 | denovo10676\_f0 | denovo10702\_f0 | denovo10780\_f0 | denovo10858\_f0 | denovo10884\_f0 | denovo10936\_f0 | denovo10988\_f0 | denovo11040\_f0 | denovo11092\_f0 | denovo11118\_f0 | denovo11170\_f0 | denovo11196\_f0 | denovo11248\_f0 | denovo11274\_f0 | denovo11326\_f0 | denovo11352\_f0 | denovo11456\_f0 | denovo11560\_f0 | denovo11924\_f0 | denovo11976\_f0 | denovo12080\_f0 |
| Msexta | Manduca sexta\* | denovo43\_f0 | denovo95\_f0 | denovo121\_f0 | denovo147\_f0 | denovo225\_f0 | denovo251\_f0 | denovo277\_f0 | denovo303\_f0 | denovo355\_f0 | denovo433\_f0 | denovo537\_f0 | denovo589\_f0 | denovo615\_f0 | denovo745\_f0 | denovo797\_f0 | denovo953\_f0 | denovo979\_f0 | denovo1057\_f0 | denovo1083\_f0 | denovo1161\_f0 | denovo1317\_f0 | denovo1499\_f0 | denovo1525\_f0 | denovo1655\_f0 | denovo1759\_f0 | denovo1785\_f0 | denovo1811\_f0 | denovo1967\_f0 | denovo2149\_f0 | denovo2201\_f0 | denovo2253\_f0 | denovo2279\_f0 | denovo2357\_f0 | denovo2461\_f0 | denovo2487\_f0 | denovo2513\_f0 | denovo2539\_f0 | denovo2591\_f0 | denovo2617\_f0 | denovo2669\_f0 | denovo2773\_f0 | denovo2799\_f0 | denovo2851\_f0 | denovo2877\_f0 | denovo2929\_f0 | denovo3007\_f0 | denovo3059\_f0 | denovo3163\_f0 | denovo3241\_f0 | denovo3267\_f0 | denovo3293\_f0 | denovo3319\_f0 | denovo3345\_f0 | denovo3449\_f0 | denovo3501\_f0 | denovo3605\_f0 | denovo3631\_f0 | denovo3683\_f0 | denovo3709\_f0 | denovo3735\_f0 | denovo3787\_f0 | denovo3865\_f0 | denovo3969\_f0 | denovo3995\_f0 | denovo4047\_f0 | denovo4073\_f0 | denovo4203\_f0 | denovo4307\_f0 | denovo4333\_f0 | denovo4359\_f0 | denovo4411\_f0 | denovo4463\_f0 | denovo4567\_f0 | denovo4645\_f0 | denovo4749\_f0 | denovo4775\_f0 | denovo4801\_f0 | denovo4853\_f0 | denovo4905\_f0 | denovo4931\_f0 | denovo4957\_f0 | denovo4983\_f0 | denovo5009\_f0 | denovo5217\_f0 | denovo5243\_f0 | denovo5269\_f0 | denovo5399\_f0 | denovo5451\_f0 | denovo5529\_f0 | denovo5607\_f0 | denovo5633\_f0 | denovo5659\_f0 | denovo5945\_f0 | denovo6023\_f0 | denovo6075\_f0 | denovo6127\_f0 | denovo6231\_f0 | denovo6257\_f0 | denovo6283\_f0 | denovo6595\_f0 | denovo6621\_f0 | denovo6699\_f0 | denovo6725\_f0 | denovo6777\_f0 | denovo6829\_f0 | denovo6881\_f0 | denovo7011\_f0 | denovo7089\_f0 | denovo7141\_f0 | denovo7167\_f0 | denovo7271\_f0 | denovo7349\_f0 | denovo7401\_f0 | denovo7427\_f0 | denovo7531\_f0 | denovo7557\_f0 | denovo7583\_f0 | denovo7635\_f0 | denovo7687\_f0 | denovo7713\_f0 | denovo7765\_f0 | denovo7817\_f0 | denovo7999\_f0 | denovo8051\_f0 | denovo8077\_f0 | denovo8233\_f0 | denovo8259\_f0 | denovo8285\_f0 | denovo8311\_f0 | denovo8415\_f0 | denovo8441\_f0 | denovo8467\_f0 | denovo8597\_f0 | denovo8649\_f0 | denovo8675\_f0 | denovo8701\_f0 | denovo8779\_f0 | denovo8831\_f0 | denovo8857\_f0 | denovo8909\_f0 | denovo8935\_f0 | denovo8961\_f0 | denovo8987\_f0 | denovo9013\_f0 | denovo9143\_f0 | denovo9169\_f0 | denovo9195\_f0 | denovo9325\_f0 | denovo9351\_f0 | denovo9377\_f0 | denovo9403\_f0 | denovo9455\_f0 | denovo9507\_f0 | denovo9559\_f0 | denovo9611\_f0 | denovo9689\_f0 | denovo9715\_f0 | denovo9741\_f0 | denovo9819\_f0 | denovo9845\_f0 | denovo9871\_f0 | denovo9897\_f0 | denovo9949\_f0 | denovo9975\_f0 | denovo10001\_f0 | denovo10079\_f0 | denovo10183\_f0 | denovo10209\_f0 | denovo10235\_f0 | denovo10287\_f0 | denovo10313\_f0 | denovo10365\_f0 | denovo10391\_f0 | denovo10417\_f0 | denovo10443\_f0 | denovo10495\_f0 | denovo10521\_f0 | denovo10547\_f0 | denovo10625\_f0 | denovo10677\_f0 | denovo10703\_f0 | denovo10781\_f0 | denovo10859\_f0 | denovo10885\_f0 | denovo10937\_f0 | denovo10989\_f0 | denovo11041\_f0 | denovo11093\_f0 | denovo11119\_f0 | denovo11171\_f0 | denovo11197\_f0 | denovo11249\_f0 | denovo11275\_f0 | denovo11327\_f0 | denovo11353\_f0 | denovo11457\_f0 | denovo11561\_f0 | denovo11925\_f0 | denovo11977\_f0 | denovo12081\_f0 |
| acti2 | Actias luna\* | denovo51\_f0 | denovo103\_f0 | denovo129\_f0 | denovo155\_f0 | denovo233\_f0 | denovo259\_f0 | denovo285\_f0 | denovo311\_f0 | denovo363\_f0 | denovo441\_f0 | denovo545\_f0 | denovo597\_f0 | denovo623\_f0 | denovo753\_f0 | denovo805\_f0 | denovo961\_f0 | denovo987\_f0 | denovo1065\_f0 | denovo1091\_f0 | denovo1169\_f0 | denovo1325\_f0 | denovo1507\_f0 | denovo1533\_f0 | denovo1663\_f0 | denovo1767\_f0 | denovo1793\_f0 | denovo1819\_f0 | denovo1975\_f0 | denovo2157\_f0 | denovo2209\_f0 | denovo2261\_f0 | denovo2287\_f0 | denovo2365\_f0 | denovo2469\_f0 | denovo2495\_f0 | denovo2521\_f0 | denovo2547\_f0 | denovo2599\_f0 | denovo2625\_f0 | denovo2677\_f0 | denovo2781\_f0 | denovo2807\_f0 | denovo2859\_f0 | denovo2885\_f0 | denovo2937\_f0 | denovo3015\_f0 | denovo3067\_f0 | denovo3171\_f0 | denovo3249\_f0 | denovo3275\_f0 | denovo3301\_f0 | denovo3327\_f0 | denovo3353\_f0 | denovo3457\_f0 | denovo3509\_f0 | denovo3613\_f0 | denovo3639\_f0 | denovo3691\_f0 | denovo3717\_f0 | denovo3743\_f0 | denovo3795\_f0 | denovo3873\_f0 | denovo3977\_f0 | denovo4003\_f0 | denovo4055\_f0 | denovo4081\_f0 | denovo4211\_f0 | denovo4315\_f0 | denovo4341\_f0 | denovo4367\_f0 | denovo4419\_f0 | denovo4471\_f0 | denovo4575\_f0 | denovo4653\_f0 | denovo4757\_f0 | denovo4783\_f0 | denovo4809\_f0 | denovo4861\_f0 | denovo4913\_f0 | denovo4939\_f0 | denovo4965\_f0 | denovo4991\_f0 | denovo5017\_f0 | denovo5225\_f0 | denovo5251\_f0 | denovo5277\_f0 | denovo5407\_f0 | denovo5459\_f0 | denovo5537\_f0 | denovo5615\_f0 | denovo5641\_f0 | denovo5667\_f0 | denovo5953\_f0 | denovo6031\_f0 | denovo6083\_f0 | denovo6135\_f0 | denovo6239\_f0 | denovo6265\_f0 | denovo6291\_f0 | denovo6603\_f0 | denovo6629\_f0 | denovo6707\_f0 | denovo6733\_f0 | denovo6785\_f0 | denovo6837\_f0 | denovo6889\_f0 | denovo7019\_f0 | denovo7097\_f0 | denovo7149\_f0 | denovo7175\_f0 | denovo7279\_f0 | denovo7357\_f0 | denovo7409\_f0 | denovo7435\_f0 | denovo7539\_f0 | denovo7565\_f0 | denovo7591\_f0 | denovo7643\_f0 | denovo7695\_f0 | denovo7721\_f0 | denovo7773\_f0 | denovo7825\_f0 | denovo8007\_f0 | denovo8059\_f0 | denovo8085\_f0 | denovo8241\_f0 | denovo8267\_f0 | denovo8293\_f0 | denovo8319\_f0 | denovo8423\_f0 | denovo8449\_f0 | denovo8475\_f0 | denovo8605\_f0 | denovo8657\_f0 | denovo8683\_f0 | denovo8709\_f0 | denovo8787\_f0 | denovo8839\_f0 | denovo8865\_f0 | denovo8917\_f0 | denovo8943\_f0 | denovo8969\_f0 | denovo8995\_f0 | denovo9021\_f0 | denovo9151\_f0 | denovo9177\_f0 | denovo9203\_f0 | denovo9333\_f0 | denovo9359\_f0 | denovo9385\_f0 | denovo9411\_f0 | denovo9463\_f0 | denovo9515\_f0 | denovo9567\_f0 | denovo9619\_f0 | denovo9697\_f0 | denovo9723\_f0 | denovo9749\_f0 | denovo9827\_f0 | denovo9853\_f0 | denovo9879\_f0 | denovo9905\_f0 | denovo9957\_f0 | denovo9983\_f0 | denovo10009\_f0 | denovo10087\_f0 | denovo10191\_f0 | denovo10217\_f0 | denovo10243\_f0 | denovo10295\_f0 | denovo10321\_f0 | denovo10373\_f0 | denovo10399\_f0 | denovo10425\_f0 | denovo10451\_f0 | denovo10503\_f0 | denovo10529\_f0 | denovo10555\_f0 | denovo10633\_f0 | denovo10685\_f0 | denovo10711\_f0 | denovo10789\_f0 | denovo10867\_f0 | denovo10893\_f0 | denovo10945\_f0 | denovo10997\_f0 | denovo11049\_f0 | denovo11101\_f0 | denovo11127\_f0 | denovo11179\_f0 | denovo11205\_f0 | denovo11257\_f0 | denovo11283\_f0 | denovo11335\_f0 | denovo11361\_f0 | denovo11465\_f0 | denovo11569\_f0 | denovo11933\_f0 | denovo11985\_f0 | denovo12089\_f0 |
| FG120071B | Myelobia sp.\* | denovo35\_f0 | denovo87\_f0 | denovo113\_f0 | denovo139\_f0 | denovo217\_f0 | denovo243\_f0 | denovo269\_f0 | denovo295\_f0 | denovo347\_f0 | denovo425\_f0 | denovo529\_f0 | denovo581\_f0 | denovo607\_f0 | denovo737\_f0 | denovo789\_f0 | denovo945\_f0 | denovo971\_f0 | denovo1049\_f0 | denovo1075\_f0 | denovo1153\_f0 | denovo1309\_f0 | denovo1491\_f0 | denovo1517\_f0 | denovo1647\_f0 | denovo1751\_f0 | denovo1777\_f0 | denovo1803\_f0 | denovo1959\_f0 | denovo2141\_f0 | denovo2193\_f0 | denovo2245\_f0 | denovo2271\_f0 | denovo2349\_f0 | denovo2453\_f0 | denovo2479\_f0 | denovo2505\_f0 | denovo2531\_f0 | denovo2583\_f0 | denovo2609\_f0 | denovo2661\_f0 | denovo2765\_f0 | denovo2791\_f0 | denovo2843\_f0 | denovo2869\_f0 | denovo2921\_f0 | denovo2999\_f0 | denovo3051\_f0 | denovo3155\_f0 | denovo3233\_f0 | denovo3259\_f0 | denovo3285\_f0 | denovo3311\_f0 | denovo3337\_f0 | denovo3441\_f0 | denovo3493\_f0 | denovo3597\_f0 | denovo3623\_f0 | denovo3675\_f0 | denovo3701\_f0 | denovo3727\_f0 | denovo3779\_f0 | denovo3857\_f0 | denovo3961\_f0 | denovo3987\_f0 | denovo4039\_f0 | denovo4065\_f0 | denovo4195\_f0 | denovo4299\_f0 | denovo4325\_f0 | denovo4351\_f0 | denovo4403\_f0 | denovo4455\_f0 | denovo4559\_f0 | denovo4637\_f0 | denovo4741\_f0 | denovo4767\_f0 | denovo4793\_f0 | denovo4845\_f0 | denovo4897\_f0 | denovo4923\_f0 | denovo4949\_f0 | denovo4975\_f0 | denovo5001\_f0 | denovo5209\_f0 | denovo5235\_f0 | denovo5261\_f0 | denovo5391\_f0 | denovo5443\_f0 | denovo5521\_f0 | denovo5599\_f0 | denovo5625\_f0 | denovo5651\_f0 | denovo5937\_f0 | denovo6015\_f0 | denovo6067\_f0 | denovo6119\_f0 | denovo6223\_f0 | denovo6249\_f0 | denovo6275\_f0 | denovo6587\_f0 | denovo6613\_f0 | denovo6691\_f0 | denovo6717\_f0 | denovo6769\_f0 | denovo6821\_f0 | denovo6873\_f0 | denovo7003\_f0 | denovo7081\_f0 | denovo7133\_f0 | denovo7159\_f0 | denovo7263\_f0 | denovo7341\_f0 | denovo7393\_f0 | denovo7419\_f0 | denovo7523\_f0 | denovo7549\_f0 | denovo7575\_f0 | denovo7627\_f0 | denovo7679\_f0 | denovo7705\_f0 | denovo7757\_f0 | denovo7809\_f0 | denovo7991\_f0 | denovo8043\_f0 | denovo8069\_f0 | denovo8225\_f0 | denovo8251\_f0 | denovo8277\_f0 | denovo8303\_f0 | denovo8407\_f0 | denovo8433\_f0 | denovo8459\_f0 | denovo8589\_f0 | denovo8641\_f0 | denovo8667\_f0 | denovo8693\_f0 | denovo8771\_f0 | denovo8823\_f0 | denovo8849\_f0 | denovo8901\_f0 | denovo8927\_f0 | denovo8953\_f0 | denovo8979\_f0 | denovo9005\_f0 | denovo9135\_f0 | denovo9161\_f0 | denovo9187\_f0 | denovo9317\_f0 | denovo9343\_f0 | denovo9369\_f0 | denovo9395\_f0 | denovo9447\_f0 | denovo9499\_f0 | denovo9551\_f0 | denovo9603\_f0 | denovo9681\_f0 | denovo9707\_f0 | denovo9733\_f0 | denovo9811\_f0 | denovo9837\_f0 | denovo9863\_f0 | denovo9889\_f0 | denovo9941\_f0 | denovo9967\_f0 | denovo9993\_f0 | denovo10071\_f0 | denovo10175\_f0 | denovo10201\_f0 | denovo10227\_f0 | denovo10279\_f0 | denovo10305\_f0 | denovo10357\_f0 | denovo10383\_f0 | denovo10409\_f0 | denovo10435\_f0 | denovo10487\_f0 | denovo10513\_f0 | denovo10539\_f0 | denovo10617\_f0 | denovo10669\_f0 | denovo10695\_f0 | denovo10773\_f0 | denovo10851\_f0 | denovo10877\_f0 | denovo10929\_f0 | denovo10981\_f0 | denovo11033\_f0 | denovo11085\_f0 | denovo11111\_f0 | denovo11163\_f0 | denovo11189\_f0 | denovo11241\_f0 | denovo11267\_f0 | denovo11319\_f0 | denovo11345\_f0 | denovo11449\_f0 | denovo11553\_f0 | denovo11917\_f0 | denovo11969\_f0 | denovo12073\_f0 |
| GNV120027 | Lantanophaga pusillidactyla\* | denovo39\_f0 | denovo91\_f0 | denovo117\_f0 | denovo143\_f0 | denovo221\_f0 | denovo247\_f0 | denovo273\_f0 | denovo299\_f0 | denovo351\_f0 | denovo429\_f0 | denovo533\_f0 | denovo585\_f0 | denovo611\_f0 | denovo741\_f0 | denovo793\_f0 | denovo949\_f0 | denovo975\_f0 | denovo1053\_f0 | denovo1079\_f0 | denovo1157\_f0 | denovo1313\_f0 | denovo1495\_f0 | denovo1521\_f0 | denovo1651\_f0 | denovo1755\_f0 | denovo1781\_f0 | denovo1807\_f0 | denovo1963\_f0 | denovo2145\_f0 | denovo2197\_f0 | denovo2249\_f0 | denovo2275\_f0 | denovo2353\_f0 | denovo2457\_f0 | denovo2483\_f0 | denovo2509\_f0 | denovo2535\_f0 | denovo2587\_f0 | denovo2613\_f0 | denovo2665\_f0 | denovo2769\_f0 | denovo2795\_f0 | denovo2847\_f0 | denovo2873\_f0 | denovo2925\_f0 | denovo3003\_f0 | denovo3055\_f0 | denovo3159\_f0 | denovo3237\_f0 | denovo3263\_f0 | denovo3289\_f0 | denovo3315\_f0 | denovo3341\_f0 | denovo3445\_f0 | denovo3497\_f0 | denovo3601\_f0 | denovo3627\_f0 | denovo3679\_f0 | denovo3705\_f0 | denovo3731\_f0 | denovo3783\_f0 | denovo3861\_f0 | denovo3965\_f0 | denovo3991\_f0 | denovo4043\_f0 | denovo4069\_f0 | denovo4199\_f0 | denovo4303\_f0 | denovo4329\_f0 | denovo4355\_f0 | denovo4407\_f0 | denovo4459\_f0 | denovo4563\_f0 | denovo4641\_f0 | denovo4745\_f0 | denovo4771\_f0 | denovo4797\_f0 | denovo4849\_f0 | denovo4901\_f0 | denovo4927\_f0 | denovo4953\_f0 | denovo4979\_f0 | denovo5005\_f0 | denovo5213\_f0 | denovo5239\_f0 | denovo5265\_f0 | denovo5395\_f0 | denovo5447\_f0 | denovo5525\_f0 | denovo5603\_f0 | denovo5629\_f0 | denovo5655\_f0 | denovo5941\_f0 | denovo6019\_f0 | denovo6071\_f0 | denovo6123\_f0 | denovo6227\_f0 | denovo6253\_f0 | denovo6279\_f0 | denovo6591\_f0 | denovo6617\_f0 | denovo6695\_f0 | denovo6721\_f0 | denovo6773\_f0 | denovo6825\_f0 | denovo6877\_f0 | denovo7007\_f0 | denovo7085\_f0 | denovo7137\_f0 | denovo7163\_f0 | denovo7267\_f0 | denovo7345\_f0 | denovo7397\_f0 | denovo7423\_f0 | denovo7527\_f0 | denovo7553\_f0 | denovo7579\_f0 | denovo7631\_f0 | denovo7683\_f0 | denovo7709\_f0 | denovo7761\_f0 | denovo7813\_f0 | denovo7995\_f0 | denovo8047\_f0 | denovo8073\_f0 | denovo8229\_f0 | denovo8255\_f0 | denovo8281\_f0 | denovo8307\_f0 | denovo8411\_f0 | denovo8437\_f0 | denovo8463\_f0 | denovo8593\_f0 | denovo8645\_f0 | denovo8671\_f0 | denovo8697\_f0 | denovo8775\_f0 | denovo8827\_f0 | denovo8853\_f0 | denovo8905\_f0 | denovo8931\_f0 | denovo8957\_f0 | denovo8983\_f0 | denovo9009\_f0 | denovo9139\_f0 | denovo9165\_f0 | denovo9191\_f0 | denovo9321\_f0 | denovo9347\_f0 | denovo9373\_f0 | denovo9399\_f0 | denovo9451\_f0 | denovo9503\_f0 | denovo9555\_f0 | denovo9607\_f0 | denovo9685\_f0 | denovo9711\_f0 | denovo9737\_f0 | denovo9815\_f0 | denovo9841\_f0 | denovo9867\_f0 | denovo9893\_f0 | denovo9945\_f0 | denovo9971\_f0 | denovo9997\_f0 | denovo10075\_f0 | denovo10179\_f0 | denovo10205\_f0 | denovo10231\_f0 | denovo10283\_f0 | denovo10309\_f0 | denovo10361\_f0 | denovo10387\_f0 | denovo10413\_f0 | denovo10439\_f0 | denovo10491\_f0 | denovo10517\_f0 | denovo10543\_f0 | denovo10621\_f0 | denovo10673\_f0 | denovo10699\_f0 | denovo10777\_f0 | denovo10855\_f0 | denovo10881\_f0 | denovo10933\_f0 | denovo10985\_f0 | denovo11037\_f0 | denovo11089\_f0 | denovo11115\_f0 | denovo11167\_f0 | denovo11193\_f0 | denovo11245\_f0 | denovo11271\_f0 | denovo11323\_f0 | denovo11349\_f0 | denovo11453\_f0 | denovo11557\_f0 | denovo11921\_f0 | denovo11973\_f0 | denovo12077\_f0 |
| Bmoricds | Bombyx mori\* | denovo26\_f0 | denovo78\_f0 | denovo104\_f0 | denovo130\_f0 | denovo208\_f0 | denovo234\_f0 | denovo260\_f0 | denovo286\_f0 | denovo338\_f0 | denovo416\_f0 | denovo520\_f0 | denovo572\_f0 | denovo598\_f0 | denovo728\_f0 | denovo780\_f0 | denovo936\_f0 | denovo962\_f0 | denovo1040\_f0 | denovo1066\_f0 | denovo1144\_f0 | denovo1300\_f0 | denovo1482\_f0 | denovo1508\_f0 | denovo1638\_f0 | denovo1742\_f0 | denovo1768\_f0 | denovo1794\_f0 | denovo1950\_f0 | denovo2132\_f0 | denovo2184\_f0 | denovo2236\_f0 | denovo2262\_f0 | denovo2340\_f0 | denovo2444\_f0 | denovo2470\_f0 | denovo2496\_f0 | denovo2522\_f0 | denovo2574\_f0 | denovo2600\_f0 | denovo2652\_f0 | denovo2756\_f0 | denovo2782\_f0 | denovo2834\_f0 | denovo2860\_f0 | denovo2912\_f0 | denovo2990\_f0 | denovo3042\_f0 | denovo3146\_f0 | denovo3224\_f0 | denovo3250\_f0 | denovo3276\_f0 | denovo3302\_f0 | denovo3328\_f0 | denovo3432\_f0 | denovo3484\_f0 | denovo3588\_f0 | denovo3614\_f0 | denovo3666\_f0 | denovo3692\_f0 | denovo3718\_f0 | denovo3770\_f0 | denovo3848\_f0 | denovo3952\_f0 | denovo3978\_f0 | denovo4030\_f0 | denovo4056\_f0 | denovo4186\_f0 | denovo4290\_f0 | denovo4316\_f0 | denovo4342\_f0 | denovo4394\_f0 | denovo4446\_f0 | denovo4550\_f0 | denovo4628\_f0 | denovo4732\_f0 | denovo4758\_f0 | denovo4784\_f0 | denovo4836\_f0 | denovo4888\_f0 | denovo4914\_f0 | denovo4940\_f0 | denovo4966\_f0 | denovo4992\_f0 | denovo5200\_f0 | denovo5226\_f0 | denovo5252\_f0 | denovo5382\_f0 | denovo5434\_f0 | denovo5512\_f0 | denovo5590\_f0 | denovo5616\_f0 | denovo5642\_f0 | denovo5928\_f0 | denovo6006\_f0 | denovo6058\_f0 | denovo6110\_f0 | denovo6214\_f0 | denovo6240\_f0 | denovo6266\_f0 | denovo6578\_f0 | denovo6604\_f0 | denovo6682\_f0 | denovo6708\_f0 | denovo6760\_f0 | denovo6812\_f0 | denovo6864\_f0 | denovo6994\_f0 | denovo7072\_f0 | denovo7124\_f0 | denovo7150\_f0 | denovo7254\_f0 | denovo7332\_f0 | denovo7384\_f0 | denovo7410\_f0 | denovo7514\_f0 | denovo7540\_f0 | denovo7566\_f0 | denovo7618\_f0 | denovo7670\_f0 | denovo7696\_f0 | denovo7748\_f0 | denovo7800\_f0 | denovo7982\_f0 | denovo8034\_f0 | denovo8060\_f0 | denovo8216\_f0 | denovo8242\_f0 | denovo8268\_f0 | denovo8294\_f0 | denovo8398\_f0 | denovo8424\_f0 | denovo8450\_f0 | denovo8580\_f0 | denovo8632\_f0 | denovo8658\_f0 | denovo8684\_f0 | denovo8762\_f0 | denovo8814\_f0 | denovo8840\_f0 | denovo8892\_f0 | denovo8918\_f0 | denovo8944\_f0 | denovo8970\_f0 | denovo8996\_f0 | denovo9126\_f0 | denovo9152\_f0 | denovo9178\_f0 | denovo9308\_f0 | denovo9334\_f0 | denovo9360\_f0 | denovo9386\_f0 | denovo9438\_f0 | denovo9490\_f0 | denovo9542\_f0 | denovo9594\_f0 | denovo9672\_f0 | denovo9698\_f0 | denovo9724\_f0 | denovo9802\_f0 | denovo9828\_f0 | denovo9854\_f0 | denovo9880\_f0 | denovo9932\_f0 | denovo9958\_f0 | denovo9984\_f0 | denovo10062\_f0 | denovo10166\_f0 | denovo10192\_f0 | denovo10218\_f0 | denovo10270\_f0 | denovo10296\_f0 | denovo10348\_f0 | denovo10374\_f0 | denovo10400\_f0 | denovo10426\_f0 | denovo10478\_f0 | denovo10504\_f0 | denovo10530\_f0 | denovo10608\_f0 | denovo10660\_f0 | denovo10686\_f0 | denovo10764\_f0 | denovo10842\_f0 | denovo10868\_f0 | denovo10920\_f0 | denovo10972\_f0 | denovo11024\_f0 | denovo11076\_f0 | denovo11102\_f0 | denovo11154\_f0 | denovo11180\_f0 | denovo11232\_f0 | denovo11258\_f0 | denovo11310\_f0 | denovo11336\_f0 | denovo11440\_f0 | denovo11544\_f0 | denovo11908\_f0 | denovo11960\_f0 | denovo12064\_f0 |
| Pcit2 | Phyllocnistis citrella\* | denovo45\_f0 | denovo97\_f0 | denovo123\_f0 | denovo149\_f0 | denovo227\_f0 | denovo253\_f0 | denovo279\_f0 | denovo305\_f0 | denovo357\_f0 | denovo435\_f0 | denovo539\_f0 | denovo591\_f0 | denovo617\_f0 | denovo747\_f0 | denovo799\_f0 | denovo955\_f0 | denovo981\_f0 | denovo1059\_f0 | denovo1085\_f0 | denovo1163\_f0 | denovo1319\_f0 | denovo1501\_f0 | denovo1527\_f0 | denovo1657\_f0 | denovo1761\_f0 | denovo1787\_f0 | denovo1813\_f0 | denovo1969\_f0 | denovo2151\_f0 | denovo2203\_f0 | denovo2255\_f0 | denovo2281\_f0 | denovo2359\_f0 | denovo2463\_f0 | denovo2489\_f0 | denovo2515\_f0 | denovo2541\_f0 | denovo2593\_f0 | denovo2619\_f0 | denovo2671\_f0 | denovo2775\_f0 | denovo2801\_f0 | denovo2853\_f0 | denovo2879\_f0 | denovo2931\_f0 | denovo3009\_f0 | denovo3061\_f0 | denovo3165\_f0 | denovo3243\_f0 | denovo3269\_f0 | denovo3295\_f0 | denovo3321\_f0 | denovo3347\_f0 | denovo3451\_f0 | denovo3503\_f0 | denovo3607\_f0 | denovo3633\_f0 | denovo3685\_f0 | denovo3711\_f0 | denovo3737\_f0 | denovo3789\_f0 | denovo3867\_f0 | denovo3971\_f0 | denovo3997\_f0 | denovo4049\_f0 | denovo4075\_f0 | denovo4205\_f0 | denovo4309\_f0 | denovo4335\_f0 | denovo4361\_f0 | denovo4413\_f0 | denovo4465\_f0 | denovo4569\_f0 | denovo4647\_f0 | denovo4751\_f0 | denovo4777\_f0 | denovo4803\_f0 | denovo4855\_f0 | denovo4907\_f0 | denovo4933\_f0 | denovo4959\_f0 | denovo4985\_f0 | denovo5011\_f0 | denovo5219\_f0 | denovo5245\_f0 | denovo5271\_f0 | denovo5401\_f0 | denovo5453\_f0 | denovo5531\_f0 | denovo5609\_f0 | denovo5635\_f0 | denovo5661\_f0 | denovo5947\_f0 | denovo6025\_f0 | denovo6077\_f0 | denovo6129\_f0 | denovo6233\_f0 | denovo6259\_f0 | denovo6285\_f0 | denovo6597\_f0 | denovo6623\_f0 | denovo6701\_f0 | denovo6727\_f0 | denovo6779\_f0 | denovo6831\_f0 | denovo6883\_f0 | denovo7013\_f0 | denovo7091\_f0 | denovo7143\_f0 | denovo7169\_f0 | denovo7273\_f0 | denovo7351\_f0 | denovo7403\_f0 | denovo7429\_f0 | denovo7533\_f0 | denovo7559\_f0 | denovo7585\_f0 | denovo7637\_f0 | denovo7689\_f0 | denovo7715\_f0 | denovo7767\_f0 | denovo7819\_f0 | denovo8001\_f0 | denovo8053\_f0 | denovo8079\_f0 | denovo8235\_f0 | denovo8261\_f0 | denovo8287\_f0 | denovo8313\_f0 | denovo8417\_f0 | denovo8443\_f0 | denovo8469\_f0 | denovo8599\_f0 | denovo8651\_f0 | denovo8677\_f0 | denovo8703\_f0 | denovo8781\_f0 | denovo8833\_f0 | denovo8859\_f0 | denovo8911\_f0 | denovo8937\_f0 | denovo8963\_f0 | denovo8989\_f0 | denovo9015\_f0 | denovo9145\_f0 | denovo9171\_f0 | denovo9197\_f0 | denovo9327\_f0 | denovo9353\_f0 | denovo9379\_f0 | denovo9405\_f0 | denovo9457\_f0 | denovo9509\_f0 | denovo9561\_f0 | denovo9613\_f0 | denovo9691\_f0 | denovo9717\_f0 | denovo9743\_f0 | denovo9821\_f0 | denovo9847\_f0 | denovo9873\_f0 | denovo9899\_f0 | denovo9951\_f0 | denovo9977\_f0 | denovo10003\_f0 | denovo10081\_f0 | denovo10185\_f0 | denovo10211\_f0 | denovo10237\_f0 | denovo10289\_f0 | denovo10315\_f0 | denovo10367\_f0 | denovo10393\_f0 | denovo10419\_f0 | denovo10445\_f0 | denovo10497\_f0 | denovo10523\_f0 | denovo10549\_f0 | denovo10627\_f0 | denovo10679\_f0 | denovo10705\_f0 | denovo10783\_f0 | denovo10861\_f0 | denovo10887\_f0 | denovo10939\_f0 | denovo10991\_f0 | denovo11043\_f0 | denovo11095\_f0 | denovo11121\_f0 | denovo11173\_f0 | denovo11199\_f0 | denovo11251\_f0 | denovo11277\_f0 | denovo11329\_f0 | denovo11355\_f0 | denovo11459\_f0 | denovo11563\_f0 | denovo11927\_f0 | denovo11979\_f0 | denovo12083\_f0 |

#### Content Of Concatenation "Entropy\_0.48\_0.00\_Loci\_50\_To\_249"

  
Rules for "entropy\_0.48\_0.00\_loci\_50\_to\_249":  
OTUs must have the loci:   
OTUs must have at least one of the following loci:

```
'EOG69CQC1_1', 'EOG6SN1SH_1', 'EOG6PRSVJ_1', 'EOG680J15_1', 'EOG60ZR1Z_1', 'EOG60P4BD_1', 'EOG64QT4T_1', 'EOG6NCMGZ_1', 'EOG6K3M19_1', 'EOG60P4BJ_1', 'EOG6DBTK0_1', 'EOG6GTKHJ_1', 'EOG68KRFS_1', 'EOG6GHZSR_1', 'EOG666VQ4_1', 'EOG698V44_1', 'EOG6894QQ_1', 'EOG6868GW_1', 'EOG69PB31_1', 'EOG6JDHBD_1', 'EOG63212K_1', 'EOG68D102_1', 'EOG60ZR1T_1', 'EOG6CJVMH_1', 'EOG698V46_1', 'EOG68KRG8_1', 'EOG6N8R81_1', 'EOG63BMSV_1', 'EOG69KFV1_1', 'EOG6CC44S_1', 'EOG6FXRCK_1', 'EOG6R2400_1', 'EOG68KRG4_1', 'EOG61C70T_1', 'EOG6BK575_1', 'EOG63212W_1', 'EOG6F7NPJ_1', 'EOG695ZW4_1', 'EOG61NTQM_1', 'EOG6NZTZ3_1', 'EOG6J3WM7_1', 'EOG680J16_1', 'EOG68SGXM_1', 'EOG6RNBCT_1', 'EOG6RFKXS_1', 'EOG67SSJJ_1', 'EOG69GKKW_1', 'EOG6QFWHZ_1', 'EOG6RFKXB_1', 'EOG647FXX_1', 'EOG6B8JHP_1', 'EOG6S1TBX_1', 'EOG6DFPRT_1', 'EOG65HS25_1', 'EOG6FFD5C_1', 'EOG6R506D_1', 'EOG6F4SFZ_1', 'EOG6Q8524_1', 'EOG6QVCGP_1', 'EOG669QZR_1', 'EOG6JT09B_1', 'EOG65TCRS_1', 'EOG6QRH88_1', 'EOG6M65N6_1', 'EOG6QRH81_1', 'EOG6RJG4W_1', 'EOG6KKZ6X_1', 'EOG60VVTG_1', 'EOG6QC198_1', 'EOG6D26TG_1', 'EOG679FBS_1', 'EOG61RPZF_1', 'EOG6PC9WG_1', 'EOG60GCVD_1', 'EOG6R506Q_1', 'EOG605S4X_1', 'EOG65TCRN_1', 'EOG608ND4_1', 'EOG6640GX_1', 'EOG6SQX12_1', 'EOG69GKMS_1', 'EOG6D26TV_1', 'EOG605S4Z_1', 'EOG6N04JN_1', 'EOG64TPCQ_1', 'EOG6NP76T_1', 'EOG67H5V2_1', 'EOG66DM62_1', 'EOG6QFWJH_1', 'EOG6N04K4_1', 'EOG69KFTV_1', 'EOG6CJVMQ_1', 'EOG62V8KS_1', 'EOG6DZ204_1', 'EOG6C5CPH_1', 'EOG6FFD51_1', 'EOG6QNN0J_1', 'EOG6KSPP5_1', 'EOG6N5W22_1', 'EOG6PK2CG_1', 'EOG6NP779_1', 'EOG6933N9_1', 'EOG66Q6XP_1', 'EOG6JQ427_1', 'EOG6C5CNP_1', 'EOG6R5066_1', 'EOG6FTW4C_1', 'EOG65B1K2_1', 'EOG66WZD1_1', 'EOG6FR0WR_1', 'EOG6P2Q64_1', 'EOG666VQC_1', 'EOG63R3R5_1', 'EOG6PC9WF_1', 'EOG6Q58T0_1', 'EOG641QGH_1', 'EOG6933N7_1', 'EOG69S6BG_1', 'EOG6NS3FV_1', 'EOG65MN9B_1', 'EOG641QFW_1', 'EOG6N04J6_1', 'EOG6GMV1P_1', 'EOG6S4PKX_1', 'EOG6BZN62_1', 'EOG6SXNGX_1', 'EOG6CG0CT_1', 'EOG6HHP6B_1', 'EOG605S4Q_1', 'EOG6R7VF9_1', 'EOG6CZBM5_1', 'EOG6JT092_1', 'EOG64F6DK_1', 'EOG6FJ8D6_1', 'EOG6QZ7QX_1', 'EOG6F1X6M_1', 'EOG67PX9B_1', 'EOG6SBF2V_1', 'EOG6KSPQ0_1', 'EOG6GMV1H_1', 'EOG63R3RC_1', 'EOG68SGX8_1', 'EOG60K83N_1', 'EOG6KD6Q9_1', 'EOG68GW6Q_1', 'EOG6Q852G_1', 'EOG676K44_1', 'EOG6FN4NZ_1', 'EOG6S1TC2_1', 'EOG6HT8WD_1', 'EOG63FH1F_1', 'EOG6CRM3H_1', 'EOG6PRSVP_1', 'EOG6R5069_1', 'EOG6CC453_1', 'EOG6001PC_1', 'EOG676K40_1', 'EOG6N30SN_1', 'EOG6255XM_1', 'EOG64BB5X_1', 'EOG62FSN7_1', 'EOG6H72G0_1', 'EOG6PZJB1_1', 'EOG65QHHP_1', 'EOG615GHV_1', 'EOG62BXCZ_1', 'EOG6CG0D0_1', 'EOG69KFVF_1', 'EOG615GHP_1', 'EOG683D8Q_1', 'EOG64J2NS_1', 'EOG66HGF5_1', 'EOG6DFPSF_1', 'EOG66MBPR_1', 'EOG6DFPSD_1', 'EOG64BB5N_1', 'EOG6DBTJX_1', 'EOG6DBTHZ_1', 'EOG6KH2ZD_1', 'EOG6STS87_1', 'EOG67D9KV_1', 'EOG6SXNHG_1', 'EOG6GF3JF_1', 'EOG61RPZ9_1', 'EOG637RJP_1', 'EOG6J3WMR_1', 'EOG6GB79H_1', 'EOG6JDHBG_1', 'EOG6DV5QM_1', 'EOG6GTKHQ_1', 'EOG6FR0W5_1', 'EOG6K0QRT_1', 'EOG65DWSM_1', 'EOG6CC452_1', 'EOG6G4GTX_1', 'EOG61G37F_1', 'EOG6FFD5V_1', 'EOG6G4GTT_1', 'EOG6GXFR5_1', 'EOG6KPTG0_1'
```

  

|  |  | EOG69CQC1\_1 | EOG6SN1SH\_1 | EOG6PRSVJ\_1 | EOG680J15\_1 | EOG60ZR1Z\_1 | EOG60P4BD\_1 | EOG64QT4T\_1 | EOG6NCMGZ\_1 | EOG6K3M19\_1 | EOG60P4BJ\_1 | EOG6DBTK0\_1 | EOG6GTKHJ\_1 | EOG68KRFS\_1 | EOG6GHZSR\_1 | EOG666VQ4\_1 | EOG698V44\_1 | EOG6894QQ\_1 | EOG6868GW\_1 | EOG69PB31\_1 | EOG6JDHBD\_1 | EOG63212K\_1 | EOG68D102\_1 | EOG60ZR1T\_1 | EOG6CJVMH\_1 | EOG698V46\_1 | EOG68KRG8\_1 | EOG6N8R81\_1 | EOG63BMSV\_1 | EOG69KFV1\_1 | EOG6CC44S\_1 | EOG6FXRCK\_1 | EOG6R2400\_1 | EOG68KRG4\_1 | EOG61C70T\_1 | EOG6BK575\_1 | EOG63212W\_1 | EOG6F7NPJ\_1 | EOG695ZW4\_1 | EOG61NTQM\_1 | EOG6NZTZ3\_1 | EOG6J3WM7\_1 | EOG680J16\_1 | EOG68SGXM\_1 | EOG6RNBCT\_1 | EOG6RFKXS\_1 | EOG67SSJJ\_1 | EOG69GKKW\_1 | EOG6QFWHZ\_1 | EOG6RFKXB\_1 | EOG647FXX\_1 | EOG6B8JHP\_1 | EOG6S1TBX\_1 | EOG6DFPRT\_1 | EOG65HS25\_1 | EOG6FFD5C\_1 | EOG6R506D\_1 | EOG6F4SFZ\_1 | EOG6Q8524\_1 | EOG6QVCGP\_1 | EOG669QZR\_1 | EOG6JT09B\_1 | EOG65TCRS\_1 | EOG6QRH88\_1 | EOG6M65N6\_1 | EOG6QRH81\_1 | EOG6RJG4W\_1 | EOG6KKZ6X\_1 | EOG60VVTG\_1 | EOG6QC198\_1 | EOG6D26TG\_1 | EOG679FBS\_1 | EOG61RPZF\_1 | EOG6PC9WG\_1 | EOG60GCVD\_1 | EOG6R506Q\_1 | EOG605S4X\_1 | EOG65TCRN\_1 | EOG608ND4\_1 | EOG6640GX\_1 | EOG6SQX12\_1 | EOG69GKMS\_1 | EOG6D26TV\_1 | EOG605S4Z\_1 | EOG6N04JN\_1 | EOG64TPCQ\_1 | EOG6NP76T\_1 | EOG67H5V2\_1 | EOG66DM62\_1 | EOG6QFWJH\_1 | EOG6N04K4\_1 | EOG69KFTV\_1 | EOG6CJVMQ\_1 | EOG62V8KS\_1 | EOG6DZ204\_1 | EOG6C5CPH\_1 | EOG6FFD51\_1 | EOG6QNN0J\_1 | EOG6KSPP5\_1 | EOG6N5W22\_1 | EOG6PK2CG\_1 | EOG6NP779\_1 | EOG6933N9\_1 | EOG66Q6XP\_1 | EOG6JQ427\_1 | EOG6C5CNP\_1 | EOG6R5066\_1 | EOG6FTW4C\_1 | EOG65B1K2\_1 | EOG66WZD1\_1 | EOG6FR0WR\_1 | EOG6P2Q64\_1 | EOG666VQC\_1 | EOG63R3R5\_1 | EOG6PC9WF\_1 | EOG6Q58T0\_1 | EOG641QGH\_1 | EOG6933N7\_1 | EOG69S6BG\_1 | EOG6NS3FV\_1 | EOG65MN9B\_1 | EOG641QFW\_1 | EOG6N04J6\_1 | EOG6GMV1P\_1 | EOG6S4PKX\_1 | EOG6BZN62\_1 | EOG6SXNGX\_1 | EOG6CG0CT\_1 | EOG6HHP6B\_1 | EOG605S4Q\_1 | EOG6R7VF9\_1 | EOG6CZBM5\_1 | EOG6JT092\_1 | EOG64F6DK\_1 | EOG6FJ8D6\_1 | EOG6QZ7QX\_1 | EOG6F1X6M\_1 | EOG67PX9B\_1 | EOG6SBF2V\_1 | EOG6KSPQ0\_1 | EOG6GMV1H\_1 | EOG63R3RC\_1 | EOG68SGX8\_1 | EOG60K83N\_1 | EOG6KD6Q9\_1 | EOG68GW6Q\_1 | EOG6Q852G\_1 | EOG676K44\_1 | EOG6FN4NZ\_1 | EOG6S1TC2\_1 | EOG6HT8WD\_1 | EOG63FH1F\_1 | EOG6CRM3H\_1 | EOG6PRSVP\_1 | EOG6R5069\_1 | EOG6CC453\_1 | EOG6001PC\_1 | EOG676K40\_1 | EOG6N30SN\_1 | EOG6255XM\_1 | EOG64BB5X\_1 | EOG62FSN7\_1 | EOG6H72G0\_1 | EOG6PZJB1\_1 | EOG65QHHP\_1 | EOG615GHV\_1 | EOG62BXCZ\_1 | EOG6CG0D0\_1 | EOG69KFVF\_1 | EOG615GHP\_1 | EOG683D8Q\_1 | EOG64J2NS\_1 | EOG66HGF5\_1 | EOG6DFPSF\_1 | EOG66MBPR\_1 | EOG6DFPSD\_1 | EOG64BB5N\_1 | EOG6DBTJX\_1 | EOG6DBTHZ\_1 | EOG6KH2ZD\_1 | EOG6STS87\_1 | EOG67D9KV\_1 | EOG6SXNHG\_1 | EOG6GF3JF\_1 | EOG61RPZ9\_1 | EOG637RJP\_1 | EOG6J3WMR\_1 | EOG6GB79H\_1 | EOG6JDHBG\_1 | EOG6DV5QM\_1 | EOG6GTKHQ\_1 | EOG6FR0W5\_1 | EOG6K0QRT\_1 | EOG65DWSM\_1 | EOG6CC452\_1 | EOG6G4GTX\_1 | EOG61G37F\_1 | EOG6FFD5V\_1 | EOG6G4GTT\_1 | EOG6GXFR5\_1 | EOG6KPTG0\_1 |
| --- | --- | --- | --- | --- | --- | --- | --- | --- | --- | --- | --- | --- | --- | --- | --- | --- | --- | --- | --- | --- | --- | --- | --- | --- | --- | --- | --- | --- | --- | --- | --- | --- | --- | --- | --- | --- | --- | --- | --- | --- | --- | --- | --- | --- | --- | --- | --- | --- | --- | --- | --- | --- | --- | --- | --- | --- | --- | --- | --- | --- | --- | --- | --- | --- | --- | --- | --- | --- | --- | --- | --- | --- | --- | --- | --- | --- | --- | --- | --- | --- | --- | --- | --- | --- | --- | --- | --- | --- | --- | --- | --- | --- | --- | --- | --- | --- | --- | --- | --- | --- | --- | --- | --- | --- | --- | --- | --- | --- | --- | --- | --- | --- | --- | --- | --- | --- | --- | --- | --- | --- | --- | --- | --- | --- | --- | --- | --- | --- | --- | --- | --- | --- | --- | --- | --- | --- | --- | --- | --- | --- | --- | --- | --- | --- | --- | --- | --- | --- | --- | --- | --- | --- | --- | --- | --- | --- | --- | --- | --- | --- | --- | --- | --- | --- | --- | --- | --- | --- | --- | --- | --- | --- | --- | --- | --- | --- | --- | --- | --- | --- | --- | --- | --- | --- | --- | --- | --- | --- | --- | --- | --- | --- | --- | --- | --- | --- | --- | --- | --- | --- | --- |
| Dplexcds | Danaus plexippus\* | denovo2\_f0 | denovo80\_f0 | denovo158\_f0 | denovo210\_f0 | denovo262\_f0 | denovo366\_f0 | denovo418\_f0 | denovo444\_f0 | denovo522\_f0 | denovo574\_f0 | denovo600\_f0 | denovo782\_f0 | denovo1042\_f0 | denovo1068\_f0 | denovo1146\_f0 | denovo1224\_f0 | denovo1354\_f0 | denovo1380\_f0 | denovo1406\_f0 | denovo1484\_f0 | denovo1510\_f0 | denovo1640\_f0 | denovo1666\_f0 | denovo1744\_f0 | denovo1796\_f0 | denovo1926\_f0 | denovo1952\_f0 | denovo1978\_f0 | denovo2004\_f0 | denovo2108\_f0 | denovo2134\_f0 | denovo2186\_f0 | denovo2238\_f0 | denovo2264\_f0 | denovo2290\_f0 | denovo2446\_f0 | denovo2472\_f0 | denovo2498\_f0 | denovo2524\_f0 | denovo2576\_f0 | denovo2602\_f0 | denovo2628\_f0 | denovo2654\_f0 | denovo2758\_f0 | denovo2836\_f0 | denovo2914\_f0 | denovo2992\_f0 | denovo3148\_f0 | denovo3252\_f0 | denovo3304\_f0 | denovo3330\_f0 | denovo3434\_f0 | denovo3486\_f0 | denovo3590\_f0 | denovo3616\_f0 | denovo3668\_f0 | denovo3720\_f0 | denovo3746\_f0 | denovo3772\_f0 | denovo3798\_f0 | denovo3824\_f0 | denovo3850\_f0 | denovo3954\_f0 | denovo3980\_f0 | denovo4058\_f0 | denovo4188\_f0 | denovo4318\_f0 | denovo4344\_f0 | denovo4370\_f0 | denovo4396\_f0 | denovo4552\_f0 | denovo4630\_f0 | denovo4734\_f0 | denovo4760\_f0 | denovo4786\_f0 | denovo4838\_f0 | denovo4890\_f0 | denovo4916\_f0 | denovo4942\_f0 | denovo4968\_f0 | denovo4994\_f0 | denovo5202\_f0 | denovo5228\_f0 | denovo5254\_f0 | denovo5436\_f0 | denovo5592\_f0 | denovo5644\_f0 | denovo5956\_f0 | denovo6008\_f0 | denovo6060\_f0 | denovo6086\_f0 | denovo6112\_f0 | denovo6190\_f0 | denovo6216\_f0 | denovo6242\_f0 | denovo6268\_f0 | denovo6294\_f0 | denovo6372\_f0 | denovo6502\_f0 | denovo6554\_f0 | denovo6580\_f0 | denovo6606\_f0 | denovo6658\_f0 | denovo6684\_f0 | denovo6710\_f0 | denovo6736\_f0 | denovo6762\_f0 | denovo6970\_f0 | denovo6996\_f0 | denovo7074\_f0 | denovo7126\_f0 | denovo7152\_f0 | denovo7256\_f0 | denovo7282\_f0 | denovo7308\_f0 | denovo7334\_f0 | denovo7360\_f0 | denovo7386\_f0 | denovo7490\_f0 | denovo7516\_f0 | denovo7542\_f0 | denovo7620\_f0 | denovo7672\_f0 | denovo7698\_f0 | denovo7828\_f0 | denovo7906\_f0 | denovo8036\_f0 | denovo8062\_f0 | denovo8218\_f0 | denovo8244\_f0 | denovo8270\_f0 | denovo8296\_f0 | denovo8374\_f0 | denovo8400\_f0 | denovo8426\_f0 | denovo8452\_f0 | denovo8556\_f0 | denovo8582\_f0 | denovo8634\_f0 | denovo8660\_f0 | denovo8686\_f0 | denovo8764\_f0 | denovo8842\_f0 | denovo8894\_f0 | denovo8920\_f0 | denovo8946\_f0 | denovo8972\_f0 | denovo8998\_f0 | denovo9128\_f0 | denovo9154\_f0 | denovo9180\_f0 | denovo9284\_f0 | denovo9310\_f0 | denovo9336\_f0 | denovo9362\_f0 | denovo9388\_f0 | denovo9440\_f0 | denovo9492\_f0 | denovo9700\_f0 | denovo9726\_f0 | denovo9778\_f0 | denovo9804\_f0 | denovo9830\_f0 | denovo9882\_f0 | denovo9960\_f0 | denovo10194\_f0 | denovo10220\_f0 | denovo10272\_f0 | denovo10298\_f0 | denovo10350\_f0 | denovo10402\_f0 | denovo10428\_f0 | denovo10454\_f0 | denovo10506\_f0 | denovo10532\_f0 | denovo10610\_f0 | denovo10662\_f0 | denovo10714\_f0 | denovo10740\_f0 | denovo10766\_f0 | denovo10818\_f0 | denovo10844\_f0 | denovo10922\_f0 | denovo11000\_f0 | denovo11026\_f0 | denovo11078\_f0 | denovo11156\_f0 | denovo11182\_f0 | denovo11286\_f0 | denovo11312\_f0 | denovo11338\_f0 | denovo11390\_f0 | denovo11416\_f0 | denovo11442\_f0 | denovo11468\_f0 | denovo11546\_f0 | denovo11702\_f0 | denovo11728\_f0 | denovo11910\_f0 | denovo12066\_f0 |
| FG120077 | Semomesia campanea | denovo10\_f0 | denovo88\_f0 | denovo166\_f0 | denovo218\_f0 | denovo270\_f0 | denovo374\_f0 | denovo426\_f0 | denovo452\_f0 | denovo530\_f0 | denovo582\_f0 | denovo608\_f0 | denovo790\_f0 | denovo1050\_f0 | denovo1076\_f0 | denovo1154\_f0 | denovo1232\_f0 | denovo1362\_f0 | denovo1388\_f0 | denovo1414\_f0 | denovo1492\_f0 | denovo1518\_f0 | denovo1648\_f0 | denovo1674\_f0 | denovo1752\_f0 | denovo1804\_f0 | denovo1934\_f0 | denovo1960\_f0 | denovo1986\_f0 | denovo2012\_f0 | denovo2116\_f0 | denovo2142\_f0 | denovo2194\_f0 | denovo2246\_f0 | denovo2272\_f0 | denovo2298\_f0 | denovo2454\_f0 | denovo2480\_f0 | denovo2506\_f0 | denovo2532\_f0 | denovo2584\_f0 | denovo2610\_f0 | denovo2636\_f0 | denovo2662\_f0 | denovo2766\_f0 | denovo2844\_f0 | denovo2922\_f0 | denovo3000\_f0 | denovo3156\_f0 | denovo3260\_f0 | denovo3312\_f0 | denovo3338\_f0 | denovo3442\_f0 | denovo3494\_f0 | denovo3598\_f0 | denovo3624\_f0 | denovo3676\_f0 | denovo3728\_f0 | denovo3754\_f0 | denovo3780\_f0 | denovo3806\_f0 | denovo3832\_f0 | denovo3858\_f0 | denovo3962\_f0 | denovo3988\_f0 | denovo4066\_f0 | denovo4196\_f0 | denovo4326\_f0 | denovo4352\_f0 | denovo4378\_f0 | denovo4404\_f0 | denovo4560\_f0 | denovo4638\_f0 | denovo4742\_f0 | denovo4768\_f0 | denovo4794\_f0 | denovo4846\_f0 | denovo4898\_f0 | denovo4924\_f0 | denovo4950\_f0 | denovo4976\_f0 | denovo5002\_f0 | denovo5210\_f0 | denovo5236\_f0 | denovo5262\_f0 | denovo5444\_f0 | denovo5600\_f0 | denovo5652\_f0 | denovo5964\_f0 | denovo6016\_f0 | denovo6068\_f0 | denovo6094\_f0 | denovo6120\_f0 | denovo6198\_f0 | denovo6224\_f0 | denovo6250\_f0 | denovo6276\_f0 | denovo6302\_f0 | denovo6380\_f0 | denovo6510\_f0 | denovo6562\_f0 | denovo6588\_f0 | denovo6614\_f0 | denovo6666\_f0 | denovo6692\_f0 | denovo6718\_f0 | denovo6744\_f0 | denovo6770\_f0 | denovo6978\_f0 | denovo7004\_f0 | denovo7082\_f0 | denovo7134\_f0 | denovo7160\_f0 | denovo7264\_f0 | denovo7290\_f0 | denovo7316\_f0 | denovo7342\_f0 | denovo7368\_f0 | denovo7394\_f0 | denovo7498\_f0 | denovo7524\_f0 | denovo7550\_f0 | denovo7628\_f0 | denovo7680\_f0 | denovo7706\_f0 | denovo7836\_f0 | denovo7914\_f0 | denovo8044\_f0 | denovo8070\_f0 | denovo8226\_f0 | denovo8252\_f0 | denovo8278\_f0 | denovo8304\_f0 | denovo8382\_f0 | denovo8408\_f0 | denovo8434\_f0 | denovo8460\_f0 | denovo8564\_f0 | denovo8590\_f0 | denovo8642\_f0 | denovo8668\_f0 | denovo8694\_f0 | denovo8772\_f0 | denovo8850\_f0 | denovo8902\_f0 | denovo8928\_f0 | denovo8954\_f0 | denovo8980\_f0 | denovo9006\_f0 | denovo9136\_f0 | denovo9162\_f0 | denovo9188\_f0 | denovo9292\_f0 | denovo9318\_f0 | denovo9344\_f0 | denovo9370\_f0 | denovo9396\_f0 | denovo9448\_f0 | denovo9500\_f0 | denovo9708\_f0 | denovo9734\_f0 | denovo9786\_f0 | denovo9812\_f0 | denovo9838\_f0 | denovo9890\_f0 | denovo9968\_f0 | denovo10202\_f0 | denovo10228\_f0 | denovo10280\_f0 | denovo10306\_f0 | denovo10358\_f0 | denovo10410\_f0 | denovo10436\_f0 | denovo10462\_f0 | denovo10514\_f0 | denovo10540\_f0 | denovo10618\_f0 | denovo10670\_f0 | denovo10722\_f0 | denovo10748\_f0 | denovo10774\_f0 | denovo10826\_f0 | denovo10852\_f0 | denovo10930\_f0 | denovo11008\_f0 | denovo11034\_f0 | denovo11086\_f0 | denovo11164\_f0 | denovo11190\_f0 | denovo11294\_f0 | denovo11320\_f0 | denovo11346\_f0 | denovo11398\_f0 | denovo11424\_f0 | denovo11450\_f0 | denovo11476\_f0 | denovo11554\_f0 | denovo11710\_f0 | denovo11736\_f0 | denovo11918\_f0 | denovo12074\_f0 |
| SRR803483 | Grapholita dimorpha\* | denovo20\_f0 | denovo98\_f0 | denovo176\_f0 | denovo228\_f0 | denovo280\_f0 | denovo384\_f0 | denovo436\_f0 | denovo462\_f0 | denovo540\_f0 | denovo592\_f0 | denovo618\_f0 | denovo800\_f0 | denovo1060\_f0 | denovo1086\_f0 | denovo1164\_f0 | denovo1242\_f0 | denovo1372\_f0 | denovo1398\_f0 | denovo1424\_f0 | denovo1502\_f0 | denovo1528\_f0 | denovo1658\_f0 | denovo1684\_f0 | denovo1762\_f0 | denovo1814\_f0 | denovo1944\_f0 | denovo1970\_f0 | denovo1996\_f0 | denovo2022\_f0 | denovo2126\_f0 | denovo2152\_f0 | denovo2204\_f0 | denovo2256\_f0 | denovo2282\_f0 | denovo2308\_f0 | denovo2464\_f0 | denovo2490\_f0 | denovo2516\_f0 | denovo2542\_f0 | denovo2594\_f0 | denovo2620\_f0 | denovo2646\_f0 | denovo2672\_f0 | denovo2776\_f0 | denovo2854\_f0 | denovo2932\_f0 | denovo3010\_f0 | denovo3166\_f0 | denovo3270\_f0 | denovo3322\_f0 | denovo3348\_f0 | denovo3452\_f0 | denovo3504\_f0 | denovo3608\_f0 | denovo3634\_f0 | denovo3686\_f0 | denovo3738\_f0 | denovo3764\_f0 | denovo3790\_f0 | denovo3816\_f0 | denovo3842\_f0 | denovo3868\_f0 | denovo3972\_f0 | denovo3998\_f0 | denovo4076\_f0 | denovo4206\_f0 | denovo4336\_f0 | denovo4362\_f0 | denovo4388\_f0 | denovo4414\_f0 | denovo4570\_f0 | denovo4648\_f0 | denovo4752\_f0 | denovo4778\_f0 | denovo4804\_f0 | denovo4856\_f0 | denovo4908\_f0 | denovo4934\_f0 | denovo4960\_f0 | denovo4986\_f0 | denovo5012\_f0 | denovo5220\_f0 | denovo5246\_f0 | denovo5272\_f0 | denovo5454\_f0 | denovo5610\_f0 | denovo5662\_f0 | denovo5974\_f0 | denovo6026\_f0 | denovo6078\_f0 | denovo6104\_f0 | denovo6130\_f0 | denovo6208\_f0 | denovo6234\_f0 | denovo6260\_f0 | denovo6286\_f0 | denovo6312\_f0 | denovo6390\_f0 | denovo6520\_f0 | denovo6572\_f0 | denovo6598\_f0 | denovo6624\_f0 | denovo6676\_f0 | denovo6702\_f0 | denovo6728\_f0 | denovo6754\_f0 | denovo6780\_f0 | denovo6988\_f0 | denovo7014\_f0 | denovo7092\_f0 | denovo7144\_f0 | denovo7170\_f0 | denovo7274\_f0 | denovo7300\_f0 | denovo7326\_f0 | denovo7352\_f0 | denovo7378\_f0 | denovo7404\_f0 | denovo7508\_f0 | denovo7534\_f0 | denovo7560\_f0 | denovo7638\_f0 | denovo7690\_f0 | denovo7716\_f0 | denovo7846\_f0 | denovo7924\_f0 | denovo8054\_f0 | denovo8080\_f0 | denovo8236\_f0 | denovo8262\_f0 | denovo8288\_f0 | denovo8314\_f0 | denovo8392\_f0 | denovo8418\_f0 | denovo8444\_f0 | denovo8470\_f0 | denovo8574\_f0 | denovo8600\_f0 | denovo8652\_f0 | denovo8678\_f0 | denovo8704\_f0 | denovo8782\_f0 | denovo8860\_f0 | denovo8912\_f0 | denovo8938\_f0 | denovo8964\_f0 | denovo8990\_f0 | denovo9016\_f0 | denovo9146\_f0 | denovo9172\_f0 | denovo9198\_f0 | denovo9302\_f0 | denovo9328\_f0 | denovo9354\_f0 | denovo9380\_f0 | denovo9406\_f0 | denovo9458\_f0 | denovo9510\_f0 | denovo9718\_f0 | denovo9744\_f0 | denovo9796\_f0 | denovo9822\_f0 | denovo9848\_f0 | denovo9900\_f0 | denovo9978\_f0 | denovo10212\_f0 | denovo10238\_f0 | denovo10290\_f0 | denovo10316\_f0 | denovo10368\_f0 | denovo10420\_f0 | denovo10446\_f0 | denovo10472\_f0 | denovo10524\_f0 | denovo10550\_f0 | denovo10628\_f0 | denovo10680\_f0 | denovo10732\_f0 | denovo10758\_f0 | denovo10784\_f0 | denovo10836\_f0 | denovo10862\_f0 | denovo10940\_f0 | denovo11018\_f0 | denovo11044\_f0 | denovo11096\_f0 | denovo11174\_f0 | denovo11200\_f0 | denovo11304\_f0 | denovo11330\_f0 | denovo11356\_f0 | denovo11408\_f0 | denovo11434\_f0 | denovo11460\_f0 | denovo11486\_f0 | denovo11564\_f0 | denovo11720\_f0 | denovo11746\_f0 | denovo11928\_f0 | denovo12084\_f0 |
| FG120035 | Dalcera abrasa\* | denovo5\_f0 | denovo83\_f0 | denovo161\_f0 | denovo213\_f0 | denovo265\_f0 | denovo369\_f0 | denovo421\_f0 | denovo447\_f0 | denovo525\_f0 | denovo577\_f0 | denovo603\_f0 | denovo785\_f0 | denovo1045\_f0 | denovo1071\_f0 | denovo1149\_f0 | denovo1227\_f0 | denovo1357\_f0 | denovo1383\_f0 | denovo1409\_f0 | denovo1487\_f0 | denovo1513\_f0 | denovo1643\_f0 | denovo1669\_f0 | denovo1747\_f0 | denovo1799\_f0 | denovo1929\_f0 | denovo1955\_f0 | denovo1981\_f0 | denovo2007\_f0 | denovo2111\_f0 | denovo2137\_f0 | denovo2189\_f0 | denovo2241\_f0 | denovo2267\_f0 | denovo2293\_f0 | denovo2449\_f0 | denovo2475\_f0 | denovo2501\_f0 | denovo2527\_f0 | denovo2579\_f0 | denovo2605\_f0 | denovo2631\_f0 | denovo2657\_f0 | denovo2761\_f0 | denovo2839\_f0 | denovo2917\_f0 | denovo2995\_f0 | denovo3151\_f0 | denovo3255\_f0 | denovo3307\_f0 | denovo3333\_f0 | denovo3437\_f0 | denovo3489\_f0 | denovo3593\_f0 | denovo3619\_f0 | denovo3671\_f0 | denovo3723\_f0 | denovo3749\_f0 | denovo3775\_f0 | denovo3801\_f0 | denovo3827\_f0 | denovo3853\_f0 | denovo3957\_f0 | denovo3983\_f0 | denovo4061\_f0 | denovo4191\_f0 | denovo4321\_f0 | denovo4347\_f0 | denovo4373\_f0 | denovo4399\_f0 | denovo4555\_f0 | denovo4633\_f0 | denovo4737\_f0 | denovo4763\_f0 | denovo4789\_f0 | denovo4841\_f0 | denovo4893\_f0 | denovo4919\_f0 | denovo4945\_f0 | denovo4971\_f0 | denovo4997\_f0 | denovo5205\_f0 | denovo5231\_f0 | denovo5257\_f0 | denovo5439\_f0 | denovo5595\_f0 | denovo5647\_f0 | denovo5959\_f0 | denovo6011\_f0 | denovo6063\_f0 | denovo6089\_f0 | denovo6115\_f0 | denovo6193\_f0 | denovo6219\_f0 | denovo6245\_f0 | denovo6271\_f0 | denovo6297\_f0 | denovo6375\_f0 | denovo6505\_f0 | denovo6557\_f0 | denovo6583\_f0 | denovo6609\_f0 | denovo6661\_f0 | denovo6687\_f0 | denovo6713\_f0 | denovo6739\_f0 | denovo6765\_f0 | denovo6973\_f0 | denovo6999\_f0 | denovo7077\_f0 | denovo7129\_f0 | denovo7155\_f0 | denovo7259\_f0 | denovo7285\_f0 | denovo7311\_f0 | denovo7337\_f0 | denovo7363\_f0 | denovo7389\_f0 | denovo7493\_f0 | denovo7519\_f0 | denovo7545\_f0 | denovo7623\_f0 | denovo7675\_f0 | denovo7701\_f0 | denovo7831\_f0 | denovo7909\_f0 | denovo8039\_f0 | denovo8065\_f0 | denovo8221\_f0 | denovo8247\_f0 | denovo8273\_f0 | denovo8299\_f0 | denovo8377\_f0 | denovo8403\_f0 | denovo8429\_f0 | denovo8455\_f0 | denovo8559\_f0 | denovo8585\_f0 | denovo8637\_f0 | denovo8663\_f0 | denovo8689\_f0 | denovo8767\_f0 | denovo8845\_f0 | denovo8897\_f0 | denovo8923\_f0 | denovo8949\_f0 | denovo8975\_f0 | denovo9001\_f0 | denovo9131\_f0 | denovo9157\_f0 | denovo9183\_f0 | denovo9287\_f0 | denovo9313\_f0 | denovo9339\_f0 | denovo9365\_f0 | denovo9391\_f0 | denovo9443\_f0 | denovo9495\_f0 | denovo9703\_f0 | denovo9729\_f0 | denovo9781\_f0 | denovo9807\_f0 | denovo9833\_f0 | denovo9885\_f0 | denovo9963\_f0 | denovo10197\_f0 | denovo10223\_f0 | denovo10275\_f0 | denovo10301\_f0 | denovo10353\_f0 | denovo10405\_f0 | denovo10431\_f0 | denovo10457\_f0 | denovo10509\_f0 | denovo10535\_f0 | denovo10613\_f0 | denovo10665\_f0 | denovo10717\_f0 | denovo10743\_f0 | denovo10769\_f0 | denovo10821\_f0 | denovo10847\_f0 | denovo10925\_f0 | denovo11003\_f0 | denovo11029\_f0 | denovo11081\_f0 | denovo11159\_f0 | denovo11185\_f0 | denovo11289\_f0 | denovo11315\_f0 | denovo11341\_f0 | denovo11393\_f0 | denovo11419\_f0 | denovo11445\_f0 | denovo11471\_f0 | denovo11549\_f0 | denovo11705\_f0 | denovo11731\_f0 | denovo11913\_f0 | denovo12069\_f0 |
| FG120046B | Lacosoma ludolpha\* | denovo6\_f0 | denovo84\_f0 | denovo162\_f0 | denovo214\_f0 | denovo266\_f0 | denovo370\_f0 | denovo422\_f0 | denovo448\_f0 | denovo526\_f0 | denovo578\_f0 | denovo604\_f0 | denovo786\_f0 | denovo1046\_f0 | denovo1072\_f0 | denovo1150\_f0 | denovo1228\_f0 | denovo1358\_f0 | denovo1384\_f0 | denovo1410\_f0 | denovo1488\_f0 | denovo1514\_f0 | denovo1644\_f0 | denovo1670\_f0 | denovo1748\_f0 | denovo1800\_f0 | denovo1930\_f0 | denovo1956\_f0 | denovo1982\_f0 | denovo2008\_f0 | denovo2112\_f0 | denovo2138\_f0 | denovo2190\_f0 | denovo2242\_f0 | denovo2268\_f0 | denovo2294\_f0 | denovo2450\_f0 | denovo2476\_f0 | denovo2502\_f0 | denovo2528\_f0 | denovo2580\_f0 | denovo2606\_f0 | denovo2632\_f0 | denovo2658\_f0 | denovo2762\_f0 | denovo2840\_f0 | denovo2918\_f0 | denovo2996\_f0 | denovo3152\_f0 | denovo3256\_f0 | denovo3308\_f0 | denovo3334\_f0 | denovo3438\_f0 | denovo3490\_f0 | denovo3594\_f0 | denovo3620\_f0 | denovo3672\_f0 | denovo3724\_f0 | denovo3750\_f0 | denovo3776\_f0 | denovo3802\_f0 | denovo3828\_f0 | denovo3854\_f0 | denovo3958\_f0 | denovo3984\_f0 | denovo4062\_f0 | denovo4192\_f0 | denovo4322\_f0 | denovo4348\_f0 | denovo4374\_f0 | denovo4400\_f0 | denovo4556\_f0 | denovo4634\_f0 | denovo4738\_f0 | denovo4764\_f0 | denovo4790\_f0 | denovo4842\_f0 | denovo4894\_f0 | denovo4920\_f0 | denovo4946\_f0 | denovo4972\_f0 | denovo4998\_f0 | denovo5206\_f0 | denovo5232\_f0 | denovo5258\_f0 | denovo5440\_f0 | denovo5596\_f0 | denovo5648\_f0 | denovo5960\_f0 | denovo6012\_f0 | denovo6064\_f0 | denovo6090\_f0 | denovo6116\_f0 | denovo6194\_f0 | denovo6220\_f0 | denovo6246\_f0 | denovo6272\_f0 | denovo6298\_f0 | denovo6376\_f0 | denovo6506\_f0 | denovo6558\_f0 | denovo6584\_f0 | denovo6610\_f0 | denovo6662\_f0 | denovo6688\_f0 | denovo6714\_f0 | denovo6740\_f0 | denovo6766\_f0 | denovo6974\_f0 | denovo7000\_f0 | denovo7078\_f0 | denovo7130\_f0 | denovo7156\_f0 | denovo7260\_f0 | denovo7286\_f0 | denovo7312\_f0 | denovo7338\_f0 | denovo7364\_f0 | denovo7390\_f0 | denovo7494\_f0 | denovo7520\_f0 | denovo7546\_f0 | denovo7624\_f0 | denovo7676\_f0 | denovo7702\_f0 | denovo7832\_f0 | denovo7910\_f0 | denovo8040\_f0 | denovo8066\_f0 | denovo8222\_f0 | denovo8248\_f0 | denovo8274\_f0 | denovo8300\_f0 | denovo8378\_f0 | denovo8404\_f0 | denovo8430\_f0 | denovo8456\_f0 | denovo8560\_f0 | denovo8586\_f0 | denovo8638\_f0 | denovo8664\_f0 | denovo8690\_f0 | denovo8768\_f0 | denovo8846\_f0 | denovo8898\_f0 | denovo8924\_f0 | denovo8950\_f0 | denovo8976\_f0 | denovo9002\_f0 | denovo9132\_f0 | denovo9158\_f0 | denovo9184\_f0 | denovo9288\_f0 | denovo9314\_f0 | denovo9340\_f0 | denovo9366\_f0 | denovo9392\_f0 | denovo9444\_f0 | denovo9496\_f0 | denovo9704\_f0 | denovo9730\_f0 | denovo9782\_f0 | denovo9808\_f0 | denovo9834\_f0 | denovo9886\_f0 | denovo9964\_f0 | denovo10198\_f0 | denovo10224\_f0 | denovo10276\_f0 | denovo10302\_f0 | denovo10354\_f0 | denovo10406\_f0 | denovo10432\_f0 | denovo10458\_f0 | denovo10510\_f0 | denovo10536\_f0 | denovo10614\_f0 | denovo10666\_f0 | denovo10718\_f0 | denovo10744\_f0 | denovo10770\_f0 | denovo10822\_f0 | denovo10848\_f0 | denovo10926\_f0 | denovo11004\_f0 | denovo11030\_f0 | denovo11082\_f0 | denovo11160\_f0 | denovo11186\_f0 | denovo11290\_f0 | denovo11316\_f0 | denovo11342\_f0 | denovo11394\_f0 | denovo11420\_f0 | denovo11446\_f0 | denovo11472\_f0 | denovo11550\_f0 | denovo11706\_f0 | denovo11732\_f0 | denovo11914\_f0 | denovo12070\_f0 |
| GNV129007 | Urodus parvula\* | denovo15\_f0 | denovo93\_f0 | denovo171\_f0 | denovo223\_f0 | denovo275\_f0 | denovo379\_f0 | denovo431\_f0 | denovo457\_f0 | denovo535\_f0 | denovo587\_f0 | denovo613\_f0 | denovo795\_f0 | denovo1055\_f0 | denovo1081\_f0 | denovo1159\_f0 | denovo1237\_f0 | denovo1367\_f0 | denovo1393\_f0 | denovo1419\_f0 | denovo1497\_f0 | denovo1523\_f0 | denovo1653\_f0 | denovo1679\_f0 | denovo1757\_f0 | denovo1809\_f0 | denovo1939\_f0 | denovo1965\_f0 | denovo1991\_f0 | denovo2017\_f0 | denovo2121\_f0 | denovo2147\_f0 | denovo2199\_f0 | denovo2251\_f0 | denovo2277\_f0 | denovo2303\_f0 | denovo2459\_f0 | denovo2485\_f0 | denovo2511\_f0 | denovo2537\_f0 | denovo2589\_f0 | denovo2615\_f0 | denovo2641\_f0 | denovo2667\_f0 | denovo2771\_f0 | denovo2849\_f0 | denovo2927\_f0 | denovo3005\_f0 | denovo3161\_f0 | denovo3265\_f0 | denovo3317\_f0 | denovo3343\_f0 | denovo3447\_f0 | denovo3499\_f0 | denovo3603\_f0 | denovo3629\_f0 | denovo3681\_f0 | denovo3733\_f0 | denovo3759\_f0 | denovo3785\_f0 | denovo3811\_f0 | denovo3837\_f0 | denovo3863\_f0 | denovo3967\_f0 | denovo3993\_f0 | denovo4071\_f0 | denovo4201\_f0 | denovo4331\_f0 | denovo4357\_f0 | denovo4383\_f0 | denovo4409\_f0 | denovo4565\_f0 | denovo4643\_f0 | denovo4747\_f0 | denovo4773\_f0 | denovo4799\_f0 | denovo4851\_f0 | denovo4903\_f0 | denovo4929\_f0 | denovo4955\_f0 | denovo4981\_f0 | denovo5007\_f0 | denovo5215\_f0 | denovo5241\_f0 | denovo5267\_f0 | denovo5449\_f0 | denovo5605\_f0 | denovo5657\_f0 | denovo5969\_f0 | denovo6021\_f0 | denovo6073\_f0 | denovo6099\_f0 | denovo6125\_f0 | denovo6203\_f0 | denovo6229\_f0 | denovo6255\_f0 | denovo6281\_f0 | denovo6307\_f0 | denovo6385\_f0 | denovo6515\_f0 | denovo6567\_f0 | denovo6593\_f0 | denovo6619\_f0 | denovo6671\_f0 | denovo6697\_f0 | denovo6723\_f0 | denovo6749\_f0 | denovo6775\_f0 | denovo6983\_f0 | denovo7009\_f0 | denovo7087\_f0 | denovo7139\_f0 | denovo7165\_f0 | denovo7269\_f0 | denovo7295\_f0 | denovo7321\_f0 | denovo7347\_f0 | denovo7373\_f0 | denovo7399\_f0 | denovo7503\_f0 | denovo7529\_f0 | denovo7555\_f0 | denovo7633\_f0 | denovo7685\_f0 | denovo7711\_f0 | denovo7841\_f0 | denovo7919\_f0 | denovo8049\_f0 | denovo8075\_f0 | denovo8231\_f0 | denovo8257\_f0 | denovo8283\_f0 | denovo8309\_f0 | denovo8387\_f0 | denovo8413\_f0 | denovo8439\_f0 | denovo8465\_f0 | denovo8569\_f0 | denovo8595\_f0 | denovo8647\_f0 | denovo8673\_f0 | denovo8699\_f0 | denovo8777\_f0 | denovo8855\_f0 | denovo8907\_f0 | denovo8933\_f0 | denovo8959\_f0 | denovo8985\_f0 | denovo9011\_f0 | denovo9141\_f0 | denovo9167\_f0 | denovo9193\_f0 | denovo9297\_f0 | denovo9323\_f0 | denovo9349\_f0 | denovo9375\_f0 | denovo9401\_f0 | denovo9453\_f0 | denovo9505\_f0 | denovo9713\_f0 | denovo9739\_f0 | denovo9791\_f0 | denovo9817\_f0 | denovo9843\_f0 | denovo9895\_f0 | denovo9973\_f0 | denovo10207\_f0 | denovo10233\_f0 | denovo10285\_f0 | denovo10311\_f0 | denovo10363\_f0 | denovo10415\_f0 | denovo10441\_f0 | denovo10467\_f0 | denovo10519\_f0 | denovo10545\_f0 | denovo10623\_f0 | denovo10675\_f0 | denovo10727\_f0 | denovo10753\_f0 | denovo10779\_f0 | denovo10831\_f0 | denovo10857\_f0 | denovo10935\_f0 | denovo11013\_f0 | denovo11039\_f0 | denovo11091\_f0 | denovo11169\_f0 | denovo11195\_f0 | denovo11299\_f0 | denovo11325\_f0 | denovo11351\_f0 | denovo11403\_f0 | denovo11429\_f0 | denovo11455\_f0 | denovo11481\_f0 | denovo11559\_f0 | denovo11715\_f0 | denovo11741\_f0 | denovo11923\_f0 | denovo12079\_f0 |
| SW130126 | Lyssa zampa\* | denovo24\_f0 | denovo102\_f0 | denovo180\_f0 | denovo232\_f0 | denovo284\_f0 | denovo388\_f0 | denovo440\_f0 | denovo466\_f0 | denovo544\_f0 | denovo596\_f0 | denovo622\_f0 | denovo804\_f0 | denovo1064\_f0 | denovo1090\_f0 | denovo1168\_f0 | denovo1246\_f0 | denovo1376\_f0 | denovo1402\_f0 | denovo1428\_f0 | denovo1506\_f0 | denovo1532\_f0 | denovo1662\_f0 | denovo1688\_f0 | denovo1766\_f0 | denovo1818\_f0 | denovo1948\_f0 | denovo1974\_f0 | denovo2000\_f0 | denovo2026\_f0 | denovo2130\_f0 | denovo2156\_f0 | denovo2208\_f0 | denovo2260\_f0 | denovo2286\_f0 | denovo2312\_f0 | denovo2468\_f0 | denovo2494\_f0 | denovo2520\_f0 | denovo2546\_f0 | denovo2598\_f0 | denovo2624\_f0 | denovo2650\_f0 | denovo2676\_f0 | denovo2780\_f0 | denovo2858\_f0 | denovo2936\_f0 | denovo3014\_f0 | denovo3170\_f0 | denovo3274\_f0 | denovo3326\_f0 | denovo3352\_f0 | denovo3456\_f0 | denovo3508\_f0 | denovo3612\_f0 | denovo3638\_f0 | denovo3690\_f0 | denovo3742\_f0 | denovo3768\_f0 | denovo3794\_f0 | denovo3820\_f0 | denovo3846\_f0 | denovo3872\_f0 | denovo3976\_f0 | denovo4002\_f0 | denovo4080\_f0 | denovo4210\_f0 | denovo4340\_f0 | denovo4366\_f0 | denovo4392\_f0 | denovo4418\_f0 | denovo4574\_f0 | denovo4652\_f0 | denovo4756\_f0 | denovo4782\_f0 | denovo4808\_f0 | denovo4860\_f0 | denovo4912\_f0 | denovo4938\_f0 | denovo4964\_f0 | denovo4990\_f0 | denovo5016\_f0 | denovo5224\_f0 | denovo5250\_f0 | denovo5276\_f0 | denovo5458\_f0 | denovo5614\_f0 | denovo5666\_f0 | denovo5978\_f0 | denovo6030\_f0 | denovo6082\_f0 | denovo6108\_f0 | denovo6134\_f0 | denovo6212\_f0 | denovo6238\_f0 | denovo6264\_f0 | denovo6290\_f0 | denovo6316\_f0 | denovo6394\_f0 | denovo6524\_f0 | denovo6576\_f0 | denovo6602\_f0 | denovo6628\_f0 | denovo6680\_f0 | denovo6706\_f0 | denovo6732\_f0 | denovo6758\_f0 | denovo6784\_f0 | denovo6992\_f0 | denovo7018\_f0 | denovo7096\_f0 | denovo7148\_f0 | denovo7174\_f0 | denovo7278\_f0 | denovo7304\_f0 | denovo7330\_f0 | denovo7356\_f0 | denovo7382\_f0 | denovo7408\_f0 | denovo7512\_f0 | denovo7538\_f0 | denovo7564\_f0 | denovo7642\_f0 | denovo7694\_f0 | denovo7720\_f0 | denovo7850\_f0 | denovo7928\_f0 | denovo8058\_f0 | denovo8084\_f0 | denovo8240\_f0 | denovo8266\_f0 | denovo8292\_f0 | denovo8318\_f0 | denovo8396\_f0 | denovo8422\_f0 | denovo8448\_f0 | denovo8474\_f0 | denovo8578\_f0 | denovo8604\_f0 | denovo8656\_f0 | denovo8682\_f0 | denovo8708\_f0 | denovo8786\_f0 | denovo8864\_f0 | denovo8916\_f0 | denovo8942\_f0 | denovo8968\_f0 | denovo8994\_f0 | denovo9020\_f0 | denovo9150\_f0 | denovo9176\_f0 | denovo9202\_f0 | denovo9306\_f0 | denovo9332\_f0 | denovo9358\_f0 | denovo9384\_f0 | denovo9410\_f0 | denovo9462\_f0 | denovo9514\_f0 | denovo9722\_f0 | denovo9748\_f0 | denovo9800\_f0 | denovo9826\_f0 | denovo9852\_f0 | denovo9904\_f0 | denovo9982\_f0 | denovo10216\_f0 | denovo10242\_f0 | denovo10294\_f0 | denovo10320\_f0 | denovo10372\_f0 | denovo10424\_f0 | denovo10450\_f0 | denovo10476\_f0 | denovo10528\_f0 | denovo10554\_f0 | denovo10632\_f0 | denovo10684\_f0 | denovo10736\_f0 | denovo10762\_f0 | denovo10788\_f0 | denovo10840\_f0 | denovo10866\_f0 | denovo10944\_f0 | denovo11022\_f0 | denovo11048\_f0 | denovo11100\_f0 | denovo11178\_f0 | denovo11204\_f0 | denovo11308\_f0 | denovo11334\_f0 | denovo11360\_f0 | denovo11412\_f0 | denovo11438\_f0 | denovo11464\_f0 | denovo11490\_f0 | denovo11568\_f0 | denovo11724\_f0 | denovo11750\_f0 | denovo11932\_f0 | denovo12088\_f0 |
| SW130103 | Anigraea sp. | denovo23\_f0 | denovo101\_f0 | denovo179\_f0 | denovo231\_f0 | denovo283\_f0 | denovo387\_f0 | denovo439\_f0 | denovo465\_f0 | denovo543\_f0 | denovo595\_f0 | denovo621\_f0 | denovo803\_f0 | denovo1063\_f0 | denovo1089\_f0 | denovo1167\_f0 | denovo1245\_f0 | denovo1375\_f0 | denovo1401\_f0 | denovo1427\_f0 | denovo1505\_f0 | denovo1531\_f0 | denovo1661\_f0 | denovo1687\_f0 | denovo1765\_f0 | denovo1817\_f0 | denovo1947\_f0 | denovo1973\_f0 | denovo1999\_f0 | denovo2025\_f0 | denovo2129\_f0 | denovo2155\_f0 | denovo2207\_f0 | denovo2259\_f0 | denovo2285\_f0 | denovo2311\_f0 | denovo2467\_f0 | denovo2493\_f0 | denovo2519\_f0 | denovo2545\_f0 | denovo2597\_f0 | denovo2623\_f0 | denovo2649\_f0 | denovo2675\_f0 | denovo2779\_f0 | denovo2857\_f0 | denovo2935\_f0 | denovo3013\_f0 | denovo3169\_f0 | denovo3273\_f0 | denovo3325\_f0 | denovo3351\_f0 | denovo3455\_f0 | denovo3507\_f0 | denovo3611\_f0 | denovo3637\_f0 | denovo3689\_f0 | denovo3741\_f0 | denovo3767\_f0 | denovo3793\_f0 | denovo3819\_f0 | denovo3845\_f0 | denovo3871\_f0 | denovo3975\_f0 | denovo4001\_f0 | denovo4079\_f0 | denovo4209\_f0 | denovo4339\_f0 | denovo4365\_f0 | denovo4391\_f0 | denovo4417\_f0 | denovo4573\_f0 | denovo4651\_f0 | denovo4755\_f0 | denovo4781\_f0 | denovo4807\_f0 | denovo4859\_f0 | denovo4911\_f0 | denovo4937\_f0 | denovo4963\_f0 | denovo4989\_f0 | denovo5015\_f0 | denovo5223\_f0 | denovo5249\_f0 | denovo5275\_f0 | denovo5457\_f0 | denovo5613\_f0 | denovo5665\_f0 | denovo5977\_f0 | denovo6029\_f0 | denovo6081\_f0 | denovo6107\_f0 | denovo6133\_f0 | denovo6211\_f0 | denovo6237\_f0 | denovo6263\_f0 | denovo6289\_f0 | denovo6315\_f0 | denovo6393\_f0 | denovo6523\_f0 | denovo6575\_f0 | denovo6601\_f0 | denovo6627\_f0 | denovo6679\_f0 | denovo6705\_f0 | denovo6731\_f0 | denovo6757\_f0 | denovo6783\_f0 | denovo6991\_f0 | denovo7017\_f0 | denovo7095\_f0 | denovo7147\_f0 | denovo7173\_f0 | denovo7277\_f0 | denovo7303\_f0 | denovo7329\_f0 | denovo7355\_f0 | denovo7381\_f0 | denovo7407\_f0 | denovo7511\_f0 | denovo7537\_f0 | denovo7563\_f0 | denovo7641\_f0 | denovo7693\_f0 | denovo7719\_f0 | denovo7849\_f0 | denovo7927\_f0 | denovo8057\_f0 | denovo8083\_f0 | denovo8239\_f0 | denovo8265\_f0 | denovo8291\_f0 | denovo8317\_f0 | denovo8395\_f0 | denovo8421\_f0 | denovo8447\_f0 | denovo8473\_f0 | denovo8577\_f0 | denovo8603\_f0 | denovo8655\_f0 | denovo8681\_f0 | denovo8707\_f0 | denovo8785\_f0 | denovo8863\_f0 | denovo8915\_f0 | denovo8941\_f0 | denovo8967\_f0 | denovo8993\_f0 | denovo9019\_f0 | denovo9149\_f0 | denovo9175\_f0 | denovo9201\_f0 | denovo9305\_f0 | denovo9331\_f0 | denovo9357\_f0 | denovo9383\_f0 | denovo9409\_f0 | denovo9461\_f0 | denovo9513\_f0 | denovo9721\_f0 | denovo9747\_f0 | denovo9799\_f0 | denovo9825\_f0 | denovo9851\_f0 | denovo9903\_f0 | denovo9981\_f0 | denovo10215\_f0 | denovo10241\_f0 | denovo10293\_f0 | denovo10319\_f0 | denovo10371\_f0 | denovo10423\_f0 | denovo10449\_f0 | denovo10475\_f0 | denovo10527\_f0 | denovo10553\_f0 | denovo10631\_f0 | denovo10683\_f0 | denovo10735\_f0 | denovo10761\_f0 | denovo10787\_f0 | denovo10839\_f0 | denovo10865\_f0 | denovo10943\_f0 | denovo11021\_f0 | denovo11047\_f0 | denovo11099\_f0 | denovo11177\_f0 | denovo11203\_f0 | denovo11307\_f0 | denovo11333\_f0 | denovo11359\_f0 | denovo11411\_f0 | denovo11437\_f0 | denovo11463\_f0 | denovo11489\_f0 | denovo11567\_f0 | denovo11723\_f0 | denovo11749\_f0 | denovo11931\_f0 | denovo12087\_f0 |
| Callid | Pterodecta felderi\* | denovo1\_f0 | denovo79\_f0 | denovo157\_f0 | denovo209\_f0 | denovo261\_f0 | denovo365\_f0 | denovo417\_f0 | denovo443\_f0 | denovo521\_f0 | denovo573\_f0 | denovo599\_f0 | denovo781\_f0 | denovo1041\_f0 | denovo1067\_f0 | denovo1145\_f0 | denovo1223\_f0 | denovo1353\_f0 | denovo1379\_f0 | denovo1405\_f0 | denovo1483\_f0 | denovo1509\_f0 | denovo1639\_f0 | denovo1665\_f0 | denovo1743\_f0 | denovo1795\_f0 | denovo1925\_f0 | denovo1951\_f0 | denovo1977\_f0 | denovo2003\_f0 | denovo2107\_f0 | denovo2133\_f0 | denovo2185\_f0 | denovo2237\_f0 | denovo2263\_f0 | denovo2289\_f0 | denovo2445\_f0 | denovo2471\_f0 | denovo2497\_f0 | denovo2523\_f0 | denovo2575\_f0 | denovo2601\_f0 | denovo2627\_f0 | denovo2653\_f0 | denovo2757\_f0 | denovo2835\_f0 | denovo2913\_f0 | denovo2991\_f0 | denovo3147\_f0 | denovo3251\_f0 | denovo3303\_f0 | denovo3329\_f0 | denovo3433\_f0 | denovo3485\_f0 | denovo3589\_f0 | denovo3615\_f0 | denovo3667\_f0 | denovo3719\_f0 | denovo3745\_f0 | denovo3771\_f0 | denovo3797\_f0 | denovo3823\_f0 | denovo3849\_f0 | denovo3953\_f0 | denovo3979\_f0 | denovo4057\_f0 | denovo4187\_f0 | denovo4317\_f0 | denovo4343\_f0 | denovo4369\_f0 | denovo4395\_f0 | denovo4551\_f0 | denovo4629\_f0 | denovo4733\_f0 | denovo4759\_f0 | denovo4785\_f0 | denovo4837\_f0 | denovo4889\_f0 | denovo4915\_f0 | denovo4941\_f0 | denovo4967\_f0 | denovo4993\_f0 | denovo5201\_f0 | denovo5227\_f0 | denovo5253\_f0 | denovo5435\_f0 | denovo5591\_f0 | denovo5643\_f0 | denovo5955\_f0 | denovo6007\_f0 | denovo6059\_f0 | denovo6085\_f0 | denovo6111\_f0 | denovo6189\_f0 | denovo6215\_f0 | denovo6241\_f0 | denovo6267\_f0 | denovo6293\_f0 | denovo6371\_f0 | denovo6501\_f0 | denovo6553\_f0 | denovo6579\_f0 | denovo6605\_f0 | denovo6657\_f0 | denovo6683\_f0 | denovo6709\_f0 | denovo6735\_f0 | denovo6761\_f0 | denovo6969\_f0 | denovo6995\_f0 | denovo7073\_f0 | denovo7125\_f0 | denovo7151\_f0 | denovo7255\_f0 | denovo7281\_f0 | denovo7307\_f0 | denovo7333\_f0 | denovo7359\_f0 | denovo7385\_f0 | denovo7489\_f0 | denovo7515\_f0 | denovo7541\_f0 | denovo7619\_f0 | denovo7671\_f0 | denovo7697\_f0 | denovo7827\_f0 | denovo7905\_f0 | denovo8035\_f0 | denovo8061\_f0 | denovo8217\_f0 | denovo8243\_f0 | denovo8269\_f0 | denovo8295\_f0 | denovo8373\_f0 | denovo8399\_f0 | denovo8425\_f0 | denovo8451\_f0 | denovo8555\_f0 | denovo8581\_f0 | denovo8633\_f0 | denovo8659\_f0 | denovo8685\_f0 | denovo8763\_f0 | denovo8841\_f0 | denovo8893\_f0 | denovo8919\_f0 | denovo8945\_f0 | denovo8971\_f0 | denovo8997\_f0 | denovo9127\_f0 | denovo9153\_f0 | denovo9179\_f0 | denovo9283\_f0 | denovo9309\_f0 | denovo9335\_f0 | denovo9361\_f0 | denovo9387\_f0 | denovo9439\_f0 | denovo9491\_f0 | denovo9699\_f0 | denovo9725\_f0 | denovo9777\_f0 | denovo9803\_f0 | denovo9829\_f0 | denovo9881\_f0 | denovo9959\_f0 | denovo10193\_f0 | denovo10219\_f0 | denovo10271\_f0 | denovo10297\_f0 | denovo10349\_f0 | denovo10401\_f0 | denovo10427\_f0 | denovo10453\_f0 | denovo10505\_f0 | denovo10531\_f0 | denovo10609\_f0 | denovo10661\_f0 | denovo10713\_f0 | denovo10739\_f0 | denovo10765\_f0 | denovo10817\_f0 | denovo10843\_f0 | denovo10921\_f0 | denovo10999\_f0 | denovo11025\_f0 | denovo11077\_f0 | denovo11155\_f0 | denovo11181\_f0 | denovo11285\_f0 | denovo11311\_f0 | denovo11337\_f0 | denovo11389\_f0 | denovo11415\_f0 | denovo11441\_f0 | denovo11467\_f0 | denovo11545\_f0 | denovo11701\_f0 | denovo11727\_f0 | denovo11909\_f0 | denovo12065\_f0 |
| FG120070B | Artace sp.\* | denovo8\_f0 | denovo86\_f0 | denovo164\_f0 | denovo216\_f0 | denovo268\_f0 | denovo372\_f0 | denovo424\_f0 | denovo450\_f0 | denovo528\_f0 | denovo580\_f0 | denovo606\_f0 | denovo788\_f0 | denovo1048\_f0 | denovo1074\_f0 | denovo1152\_f0 | denovo1230\_f0 | denovo1360\_f0 | denovo1386\_f0 | denovo1412\_f0 | denovo1490\_f0 | denovo1516\_f0 | denovo1646\_f0 | denovo1672\_f0 | denovo1750\_f0 | denovo1802\_f0 | denovo1932\_f0 | denovo1958\_f0 | denovo1984\_f0 | denovo2010\_f0 | denovo2114\_f0 | denovo2140\_f0 | denovo2192\_f0 | denovo2244\_f0 | denovo2270\_f0 | denovo2296\_f0 | denovo2452\_f0 | denovo2478\_f0 | denovo2504\_f0 | denovo2530\_f0 | denovo2582\_f0 | denovo2608\_f0 | denovo2634\_f0 | denovo2660\_f0 | denovo2764\_f0 | denovo2842\_f0 | denovo2920\_f0 | denovo2998\_f0 | denovo3154\_f0 | denovo3258\_f0 | denovo3310\_f0 | denovo3336\_f0 | denovo3440\_f0 | denovo3492\_f0 | denovo3596\_f0 | denovo3622\_f0 | denovo3674\_f0 | denovo3726\_f0 | denovo3752\_f0 | denovo3778\_f0 | denovo3804\_f0 | denovo3830\_f0 | denovo3856\_f0 | denovo3960\_f0 | denovo3986\_f0 | denovo4064\_f0 | denovo4194\_f0 | denovo4324\_f0 | denovo4350\_f0 | denovo4376\_f0 | denovo4402\_f0 | denovo4558\_f0 | denovo4636\_f0 | denovo4740\_f0 | denovo4766\_f0 | denovo4792\_f0 | denovo4844\_f0 | denovo4896\_f0 | denovo4922\_f0 | denovo4948\_f0 | denovo4974\_f0 | denovo5000\_f0 | denovo5208\_f0 | denovo5234\_f0 | denovo5260\_f0 | denovo5442\_f0 | denovo5598\_f0 | denovo5650\_f0 | denovo5962\_f0 | denovo6014\_f0 | denovo6066\_f0 | denovo6092\_f0 | denovo6118\_f0 | denovo6196\_f0 | denovo6222\_f0 | denovo6248\_f0 | denovo6274\_f0 | denovo6300\_f0 | denovo6378\_f0 | denovo6508\_f0 | denovo6560\_f0 | denovo6586\_f0 | denovo6612\_f0 | denovo6664\_f0 | denovo6690\_f0 | denovo6716\_f0 | denovo6742\_f0 | denovo6768\_f0 | denovo6976\_f0 | denovo7002\_f0 | denovo7080\_f0 | denovo7132\_f0 | denovo7158\_f0 | denovo7262\_f0 | denovo7288\_f0 | denovo7314\_f0 | denovo7340\_f0 | denovo7366\_f0 | denovo7392\_f0 | denovo7496\_f0 | denovo7522\_f0 | denovo7548\_f0 | denovo7626\_f0 | denovo7678\_f0 | denovo7704\_f0 | denovo7834\_f0 | denovo7912\_f0 | denovo8042\_f0 | denovo8068\_f0 | denovo8224\_f0 | denovo8250\_f0 | denovo8276\_f0 | denovo8302\_f0 | denovo8380\_f0 | denovo8406\_f0 | denovo8432\_f0 | denovo8458\_f0 | denovo8562\_f0 | denovo8588\_f0 | denovo8640\_f0 | denovo8666\_f0 | denovo8692\_f0 | denovo8770\_f0 | denovo8848\_f0 | denovo8900\_f0 | denovo8926\_f0 | denovo8952\_f0 | denovo8978\_f0 | denovo9004\_f0 | denovo9134\_f0 | denovo9160\_f0 | denovo9186\_f0 | denovo9290\_f0 | denovo9316\_f0 | denovo9342\_f0 | denovo9368\_f0 | denovo9394\_f0 | denovo9446\_f0 | denovo9498\_f0 | denovo9706\_f0 | denovo9732\_f0 | denovo9784\_f0 | denovo9810\_f0 | denovo9836\_f0 | denovo9888\_f0 | denovo9966\_f0 | denovo10200\_f0 | denovo10226\_f0 | denovo10278\_f0 | denovo10304\_f0 | denovo10356\_f0 | denovo10408\_f0 | denovo10434\_f0 | denovo10460\_f0 | denovo10512\_f0 | denovo10538\_f0 | denovo10616\_f0 | denovo10668\_f0 | denovo10720\_f0 | denovo10746\_f0 | denovo10772\_f0 | denovo10824\_f0 | denovo10850\_f0 | denovo10928\_f0 | denovo11006\_f0 | denovo11032\_f0 | denovo11084\_f0 | denovo11162\_f0 | denovo11188\_f0 | denovo11292\_f0 | denovo11318\_f0 | denovo11344\_f0 | denovo11396\_f0 | denovo11422\_f0 | denovo11448\_f0 | denovo11474\_f0 | denovo11552\_f0 | denovo11708\_f0 | denovo11734\_f0 | denovo11916\_f0 | denovo12072\_f0 |
| SW130007 | Thubana sp.\* | denovo22\_f0 | denovo100\_f0 | denovo178\_f0 | denovo230\_f0 | denovo282\_f0 | denovo386\_f0 | denovo438\_f0 | denovo464\_f0 | denovo542\_f0 | denovo594\_f0 | denovo620\_f0 | denovo802\_f0 | denovo1062\_f0 | denovo1088\_f0 | denovo1166\_f0 | denovo1244\_f0 | denovo1374\_f0 | denovo1400\_f0 | denovo1426\_f0 | denovo1504\_f0 | denovo1530\_f0 | denovo1660\_f0 | denovo1686\_f0 | denovo1764\_f0 | denovo1816\_f0 | denovo1946\_f0 | denovo1972\_f0 | denovo1998\_f0 | denovo2024\_f0 | denovo2128\_f0 | denovo2154\_f0 | denovo2206\_f0 | denovo2258\_f0 | denovo2284\_f0 | denovo2310\_f0 | denovo2466\_f0 | denovo2492\_f0 | denovo2518\_f0 | denovo2544\_f0 | denovo2596\_f0 | denovo2622\_f0 | denovo2648\_f0 | denovo2674\_f0 | denovo2778\_f0 | denovo2856\_f0 | denovo2934\_f0 | denovo3012\_f0 | denovo3168\_f0 | denovo3272\_f0 | denovo3324\_f0 | denovo3350\_f0 | denovo3454\_f0 | denovo3506\_f0 | denovo3610\_f0 | denovo3636\_f0 | denovo3688\_f0 | denovo3740\_f0 | denovo3766\_f0 | denovo3792\_f0 | denovo3818\_f0 | denovo3844\_f0 | denovo3870\_f0 | denovo3974\_f0 | denovo4000\_f0 | denovo4078\_f0 | denovo4208\_f0 | denovo4338\_f0 | denovo4364\_f0 | denovo4390\_f0 | denovo4416\_f0 | denovo4572\_f0 | denovo4650\_f0 | denovo4754\_f0 | denovo4780\_f0 | denovo4806\_f0 | denovo4858\_f0 | denovo4910\_f0 | denovo4936\_f0 | denovo4962\_f0 | denovo4988\_f0 | denovo5014\_f0 | denovo5222\_f0 | denovo5248\_f0 | denovo5274\_f0 | denovo5456\_f0 | denovo5612\_f0 | denovo5664\_f0 | denovo5976\_f0 | denovo6028\_f0 | denovo6080\_f0 | denovo6106\_f0 | denovo6132\_f0 | denovo6210\_f0 | denovo6236\_f0 | denovo6262\_f0 | denovo6288\_f0 | denovo6314\_f0 | denovo6392\_f0 | denovo6522\_f0 | denovo6574\_f0 | denovo6600\_f0 | denovo6626\_f0 | denovo6678\_f0 | denovo6704\_f0 | denovo6730\_f0 | denovo6756\_f0 | denovo6782\_f0 | denovo6990\_f0 | denovo7016\_f0 | denovo7094\_f0 | denovo7146\_f0 | denovo7172\_f0 | denovo7276\_f0 | denovo7302\_f0 | denovo7328\_f0 | denovo7354\_f0 | denovo7380\_f0 | denovo7406\_f0 | denovo7510\_f0 | denovo7536\_f0 | denovo7562\_f0 | denovo7640\_f0 | denovo7692\_f0 | denovo7718\_f0 | denovo7848\_f0 | denovo7926\_f0 | denovo8056\_f0 | denovo8082\_f0 | denovo8238\_f0 | denovo8264\_f0 | denovo8290\_f0 | denovo8316\_f0 | denovo8394\_f0 | denovo8420\_f0 | denovo8446\_f0 | denovo8472\_f0 | denovo8576\_f0 | denovo8602\_f0 | denovo8654\_f0 | denovo8680\_f0 | denovo8706\_f0 | denovo8784\_f0 | denovo8862\_f0 | denovo8914\_f0 | denovo8940\_f0 | denovo8966\_f0 | denovo8992\_f0 | denovo9018\_f0 | denovo9148\_f0 | denovo9174\_f0 | denovo9200\_f0 | denovo9304\_f0 | denovo9330\_f0 | denovo9356\_f0 | denovo9382\_f0 | denovo9408\_f0 | denovo9460\_f0 | denovo9512\_f0 | denovo9720\_f0 | denovo9746\_f0 | denovo9798\_f0 | denovo9824\_f0 | denovo9850\_f0 | denovo9902\_f0 | denovo9980\_f0 | denovo10214\_f0 | denovo10240\_f0 | denovo10292\_f0 | denovo10318\_f0 | denovo10370\_f0 | denovo10422\_f0 | denovo10448\_f0 | denovo10474\_f0 | denovo10526\_f0 | denovo10552\_f0 | denovo10630\_f0 | denovo10682\_f0 | denovo10734\_f0 | denovo10760\_f0 | denovo10786\_f0 | denovo10838\_f0 | denovo10864\_f0 | denovo10942\_f0 | denovo11020\_f0 | denovo11046\_f0 | denovo11098\_f0 | denovo11176\_f0 | denovo11202\_f0 | denovo11306\_f0 | denovo11332\_f0 | denovo11358\_f0 | denovo11410\_f0 | denovo11436\_f0 | denovo11462\_f0 | denovo11488\_f0 | denovo11566\_f0 | denovo11722\_f0 | denovo11748\_f0 | denovo11930\_f0 | denovo12086\_f0 |
| GNV120032 | Nemoria lixaria\* | denovo14\_f0 | denovo92\_f0 | denovo170\_f0 | denovo222\_f0 | denovo274\_f0 | denovo378\_f0 | denovo430\_f0 | denovo456\_f0 | denovo534\_f0 | denovo586\_f0 | denovo612\_f0 | denovo794\_f0 | denovo1054\_f0 | denovo1080\_f0 | denovo1158\_f0 | denovo1236\_f0 | denovo1366\_f0 | denovo1392\_f0 | denovo1418\_f0 | denovo1496\_f0 | denovo1522\_f0 | denovo1652\_f0 | denovo1678\_f0 | denovo1756\_f0 | denovo1808\_f0 | denovo1938\_f0 | denovo1964\_f0 | denovo1990\_f0 | denovo2016\_f0 | denovo2120\_f0 | denovo2146\_f0 | denovo2198\_f0 | denovo2250\_f0 | denovo2276\_f0 | denovo2302\_f0 | denovo2458\_f0 | denovo2484\_f0 | denovo2510\_f0 | denovo2536\_f0 | denovo2588\_f0 | denovo2614\_f0 | denovo2640\_f0 | denovo2666\_f0 | denovo2770\_f0 | denovo2848\_f0 | denovo2926\_f0 | denovo3004\_f0 | denovo3160\_f0 | denovo3264\_f0 | denovo3316\_f0 | denovo3342\_f0 | denovo3446\_f0 | denovo3498\_f0 | denovo3602\_f0 | denovo3628\_f0 | denovo3680\_f0 | denovo3732\_f0 | denovo3758\_f0 | denovo3784\_f0 | denovo3810\_f0 | denovo3836\_f0 | denovo3862\_f0 | denovo3966\_f0 | denovo3992\_f0 | denovo4070\_f0 | denovo4200\_f0 | denovo4330\_f0 | denovo4356\_f0 | denovo4382\_f0 | denovo4408\_f0 | denovo4564\_f0 | denovo4642\_f0 | denovo4746\_f0 | denovo4772\_f0 | denovo4798\_f0 | denovo4850\_f0 | denovo4902\_f0 | denovo4928\_f0 | denovo4954\_f0 | denovo4980\_f0 | denovo5006\_f0 | denovo5214\_f0 | denovo5240\_f0 | denovo5266\_f0 | denovo5448\_f0 | denovo5604\_f0 | denovo5656\_f0 | denovo5968\_f0 | denovo6020\_f0 | denovo6072\_f0 | denovo6098\_f0 | denovo6124\_f0 | denovo6202\_f0 | denovo6228\_f0 | denovo6254\_f0 | denovo6280\_f0 | denovo6306\_f0 | denovo6384\_f0 | denovo6514\_f0 | denovo6566\_f0 | denovo6592\_f0 | denovo6618\_f0 | denovo6670\_f0 | denovo6696\_f0 | denovo6722\_f0 | denovo6748\_f0 | denovo6774\_f0 | denovo6982\_f0 | denovo7008\_f0 | denovo7086\_f0 | denovo7138\_f0 | denovo7164\_f0 | denovo7268\_f0 | denovo7294\_f0 | denovo7320\_f0 | denovo7346\_f0 | denovo7372\_f0 | denovo7398\_f0 | denovo7502\_f0 | denovo7528\_f0 | denovo7554\_f0 | denovo7632\_f0 | denovo7684\_f0 | denovo7710\_f0 | denovo7840\_f0 | denovo7918\_f0 | denovo8048\_f0 | denovo8074\_f0 | denovo8230\_f0 | denovo8256\_f0 | denovo8282\_f0 | denovo8308\_f0 | denovo8386\_f0 | denovo8412\_f0 | denovo8438\_f0 | denovo8464\_f0 | denovo8568\_f0 | denovo8594\_f0 | denovo8646\_f0 | denovo8672\_f0 | denovo8698\_f0 | denovo8776\_f0 | denovo8854\_f0 | denovo8906\_f0 | denovo8932\_f0 | denovo8958\_f0 | denovo8984\_f0 | denovo9010\_f0 | denovo9140\_f0 | denovo9166\_f0 | denovo9192\_f0 | denovo9296\_f0 | denovo9322\_f0 | denovo9348\_f0 | denovo9374\_f0 | denovo9400\_f0 | denovo9452\_f0 | denovo9504\_f0 | denovo9712\_f0 | denovo9738\_f0 | denovo9790\_f0 | denovo9816\_f0 | denovo9842\_f0 | denovo9894\_f0 | denovo9972\_f0 | denovo10206\_f0 | denovo10232\_f0 | denovo10284\_f0 | denovo10310\_f0 | denovo10362\_f0 | denovo10414\_f0 | denovo10440\_f0 | denovo10466\_f0 | denovo10518\_f0 | denovo10544\_f0 | denovo10622\_f0 | denovo10674\_f0 | denovo10726\_f0 | denovo10752\_f0 | denovo10778\_f0 | denovo10830\_f0 | denovo10856\_f0 | denovo10934\_f0 | denovo11012\_f0 | denovo11038\_f0 | denovo11090\_f0 | denovo11168\_f0 | denovo11194\_f0 | denovo11298\_f0 | denovo11324\_f0 | denovo11350\_f0 | denovo11402\_f0 | denovo11428\_f0 | denovo11454\_f0 | denovo11480\_f0 | denovo11558\_f0 | denovo11714\_f0 | denovo11740\_f0 | denovo11922\_f0 | denovo12078\_f0 |
| PXYLO | Plutella xylostella\* | denovo18\_f0 | denovo96\_f0 | denovo174\_f0 | denovo226\_f0 | denovo278\_f0 | denovo382\_f0 | denovo434\_f0 | denovo460\_f0 | denovo538\_f0 | denovo590\_f0 | denovo616\_f0 | denovo798\_f0 | denovo1058\_f0 | denovo1084\_f0 | denovo1162\_f0 | denovo1240\_f0 | denovo1370\_f0 | denovo1396\_f0 | denovo1422\_f0 | denovo1500\_f0 | denovo1526\_f0 | denovo1656\_f0 | denovo1682\_f0 | denovo1760\_f0 | denovo1812\_f0 | denovo1942\_f0 | denovo1968\_f0 | denovo1994\_f0 | denovo2020\_f0 | denovo2124\_f0 | denovo2150\_f0 | denovo2202\_f0 | denovo2254\_f0 | denovo2280\_f0 | denovo2306\_f0 | denovo2462\_f0 | denovo2488\_f0 | denovo2514\_f0 | denovo2540\_f0 | denovo2592\_f0 | denovo2618\_f0 | denovo2644\_f0 | denovo2670\_f0 | denovo2774\_f0 | denovo2852\_f0 | denovo2930\_f0 | denovo3008\_f0 | denovo3164\_f0 | denovo3268\_f0 | denovo3320\_f0 | denovo3346\_f0 | denovo3450\_f0 | denovo3502\_f0 | denovo3606\_f0 | denovo3632\_f0 | denovo3684\_f0 | denovo3736\_f0 | denovo3762\_f0 | denovo3788\_f0 | denovo3814\_f0 | denovo3840\_f0 | denovo3866\_f0 | denovo3970\_f0 | denovo3996\_f0 | denovo4074\_f0 | denovo4204\_f0 | denovo4334\_f0 | denovo4360\_f0 | denovo4386\_f0 | denovo4412\_f0 | denovo4568\_f0 | denovo4646\_f0 | denovo4750\_f0 | denovo4776\_f0 | denovo4802\_f0 | denovo4854\_f0 | denovo4906\_f0 | denovo4932\_f0 | denovo4958\_f0 | denovo4984\_f0 | denovo5010\_f0 | denovo5218\_f0 | denovo5244\_f0 | denovo5270\_f0 | denovo5452\_f0 | denovo5608\_f0 | denovo5660\_f0 | denovo5972\_f0 | denovo6024\_f0 | denovo6076\_f0 | denovo6102\_f0 | denovo6128\_f0 | denovo6206\_f0 | denovo6232\_f0 | denovo6258\_f0 | denovo6284\_f0 | denovo6310\_f0 | denovo6388\_f0 | denovo6518\_f0 | denovo6570\_f0 | denovo6596\_f0 | denovo6622\_f0 | denovo6674\_f0 | denovo6700\_f0 | denovo6726\_f0 | denovo6752\_f0 | denovo6778\_f0 | denovo6986\_f0 | denovo7012\_f0 | denovo7090\_f0 | denovo7142\_f0 | denovo7168\_f0 | denovo7272\_f0 | denovo7298\_f0 | denovo7324\_f0 | denovo7350\_f0 | denovo7376\_f0 | denovo7402\_f0 | denovo7506\_f0 | denovo7532\_f0 | denovo7558\_f0 | denovo7636\_f0 | denovo7688\_f0 | denovo7714\_f0 | denovo7844\_f0 | denovo7922\_f0 | denovo8052\_f0 | denovo8078\_f0 | denovo8234\_f0 | denovo8260\_f0 | denovo8286\_f0 | denovo8312\_f0 | denovo8390\_f0 | denovo8416\_f0 | denovo8442\_f0 | denovo8468\_f0 | denovo8572\_f0 | denovo8598\_f0 | denovo8650\_f0 | denovo8676\_f0 | denovo8702\_f0 | denovo8780\_f0 | denovo8858\_f0 | denovo8910\_f0 | denovo8936\_f0 | denovo8962\_f0 | denovo8988\_f0 | denovo9014\_f0 | denovo9144\_f0 | denovo9170\_f0 | denovo9196\_f0 | denovo9300\_f0 | denovo9326\_f0 | denovo9352\_f0 | denovo9378\_f0 | denovo9404\_f0 | denovo9456\_f0 | denovo9508\_f0 | denovo9716\_f0 | denovo9742\_f0 | denovo9794\_f0 | denovo9820\_f0 | denovo9846\_f0 | denovo9898\_f0 | denovo9976\_f0 | denovo10210\_f0 | denovo10236\_f0 | denovo10288\_f0 | denovo10314\_f0 | denovo10366\_f0 | denovo10418\_f0 | denovo10444\_f0 | denovo10470\_f0 | denovo10522\_f0 | denovo10548\_f0 | denovo10626\_f0 | denovo10678\_f0 | denovo10730\_f0 | denovo10756\_f0 | denovo10782\_f0 | denovo10834\_f0 | denovo10860\_f0 | denovo10938\_f0 | denovo11016\_f0 | denovo11042\_f0 | denovo11094\_f0 | denovo11172\_f0 | denovo11198\_f0 | denovo11302\_f0 | denovo11328\_f0 | denovo11354\_f0 | denovo11406\_f0 | denovo11432\_f0 | denovo11458\_f0 | denovo11484\_f0 | denovo11562\_f0 | denovo11718\_f0 | denovo11744\_f0 | denovo11926\_f0 | denovo12082\_f0 |
| FG120079 | Zeuzerodes maculata\* | denovo11\_f0 | denovo89\_f0 | denovo167\_f0 | denovo219\_f0 | denovo271\_f0 | denovo375\_f0 | denovo427\_f0 | denovo453\_f0 | denovo531\_f0 | denovo583\_f0 | denovo609\_f0 | denovo791\_f0 | denovo1051\_f0 | denovo1077\_f0 | denovo1155\_f0 | denovo1233\_f0 | denovo1363\_f0 | denovo1389\_f0 | denovo1415\_f0 | denovo1493\_f0 | denovo1519\_f0 | denovo1649\_f0 | denovo1675\_f0 | denovo1753\_f0 | denovo1805\_f0 | denovo1935\_f0 | denovo1961\_f0 | denovo1987\_f0 | denovo2013\_f0 | denovo2117\_f0 | denovo2143\_f0 | denovo2195\_f0 | denovo2247\_f0 | denovo2273\_f0 | denovo2299\_f0 | denovo2455\_f0 | denovo2481\_f0 | denovo2507\_f0 | denovo2533\_f0 | denovo2585\_f0 | denovo2611\_f0 | denovo2637\_f0 | denovo2663\_f0 | denovo2767\_f0 | denovo2845\_f0 | denovo2923\_f0 | denovo3001\_f0 | denovo3157\_f0 | denovo3261\_f0 | denovo3313\_f0 | denovo3339\_f0 | denovo3443\_f0 | denovo3495\_f0 | denovo3599\_f0 | denovo3625\_f0 | denovo3677\_f0 | denovo3729\_f0 | denovo3755\_f0 | denovo3781\_f0 | denovo3807\_f0 | denovo3833\_f0 | denovo3859\_f0 | denovo3963\_f0 | denovo3989\_f0 | denovo4067\_f0 | denovo4197\_f0 | denovo4327\_f0 | denovo4353\_f0 | denovo4379\_f0 | denovo4405\_f0 | denovo4561\_f0 | denovo4639\_f0 | denovo4743\_f0 | denovo4769\_f0 | denovo4795\_f0 | denovo4847\_f0 | denovo4899\_f0 | denovo4925\_f0 | denovo4951\_f0 | denovo4977\_f0 | denovo5003\_f0 | denovo5211\_f0 | denovo5237\_f0 | denovo5263\_f0 | denovo5445\_f0 | denovo5601\_f0 | denovo5653\_f0 | denovo5965\_f0 | denovo6017\_f0 | denovo6069\_f0 | denovo6095\_f0 | denovo6121\_f0 | denovo6199\_f0 | denovo6225\_f0 | denovo6251\_f0 | denovo6277\_f0 | denovo6303\_f0 | denovo6381\_f0 | denovo6511\_f0 | denovo6563\_f0 | denovo6589\_f0 | denovo6615\_f0 | denovo6667\_f0 | denovo6693\_f0 | denovo6719\_f0 | denovo6745\_f0 | denovo6771\_f0 | denovo6979\_f0 | denovo7005\_f0 | denovo7083\_f0 | denovo7135\_f0 | denovo7161\_f0 | denovo7265\_f0 | denovo7291\_f0 | denovo7317\_f0 | denovo7343\_f0 | denovo7369\_f0 | denovo7395\_f0 | denovo7499\_f0 | denovo7525\_f0 | denovo7551\_f0 | denovo7629\_f0 | denovo7681\_f0 | denovo7707\_f0 | denovo7837\_f0 | denovo7915\_f0 | denovo8045\_f0 | denovo8071\_f0 | denovo8227\_f0 | denovo8253\_f0 | denovo8279\_f0 | denovo8305\_f0 | denovo8383\_f0 | denovo8409\_f0 | denovo8435\_f0 | denovo8461\_f0 | denovo8565\_f0 | denovo8591\_f0 | denovo8643\_f0 | denovo8669\_f0 | denovo8695\_f0 | denovo8773\_f0 | denovo8851\_f0 | denovo8903\_f0 | denovo8929\_f0 | denovo8955\_f0 | denovo8981\_f0 | denovo9007\_f0 | denovo9137\_f0 | denovo9163\_f0 | denovo9189\_f0 | denovo9293\_f0 | denovo9319\_f0 | denovo9345\_f0 | denovo9371\_f0 | denovo9397\_f0 | denovo9449\_f0 | denovo9501\_f0 | denovo9709\_f0 | denovo9735\_f0 | denovo9787\_f0 | denovo9813\_f0 | denovo9839\_f0 | denovo9891\_f0 | denovo9969\_f0 | denovo10203\_f0 | denovo10229\_f0 | denovo10281\_f0 | denovo10307\_f0 | denovo10359\_f0 | denovo10411\_f0 | denovo10437\_f0 | denovo10463\_f0 | denovo10515\_f0 | denovo10541\_f0 | denovo10619\_f0 | denovo10671\_f0 | denovo10723\_f0 | denovo10749\_f0 | denovo10775\_f0 | denovo10827\_f0 | denovo10853\_f0 | denovo10931\_f0 | denovo11009\_f0 | denovo11035\_f0 | denovo11087\_f0 | denovo11165\_f0 | denovo11191\_f0 | denovo11295\_f0 | denovo11321\_f0 | denovo11347\_f0 | denovo11399\_f0 | denovo11425\_f0 | denovo11451\_f0 | denovo11477\_f0 | denovo11555\_f0 | denovo11711\_f0 | denovo11737\_f0 | denovo11919\_f0 | denovo12075\_f0 |
| SRR850324 | Papilio glaucus | denovo21\_f0 | denovo99\_f0 | denovo177\_f0 | denovo229\_f0 | denovo281\_f0 | denovo385\_f0 | denovo437\_f0 | denovo463\_f0 | denovo541\_f0 | denovo593\_f0 | denovo619\_f0 | denovo801\_f0 | denovo1061\_f0 | denovo1087\_f0 | denovo1165\_f0 | denovo1243\_f0 | denovo1373\_f0 | denovo1399\_f0 | denovo1425\_f0 | denovo1503\_f0 | denovo1529\_f0 | denovo1659\_f0 | denovo1685\_f0 | denovo1763\_f0 | denovo1815\_f0 | denovo1945\_f0 | denovo1971\_f0 | denovo1997\_f0 | denovo2023\_f0 | denovo2127\_f0 | denovo2153\_f0 | denovo2205\_f0 | denovo2257\_f0 | denovo2283\_f0 | denovo2309\_f0 | denovo2465\_f0 | denovo2491\_f0 | denovo2517\_f0 | denovo2543\_f0 | denovo2595\_f0 | denovo2621\_f0 | denovo2647\_f0 | denovo2673\_f0 | denovo2777\_f0 | denovo2855\_f0 | denovo2933\_f0 | denovo3011\_f0 | denovo3167\_f0 | denovo3271\_f0 | denovo3323\_f0 | denovo3349\_f0 | denovo3453\_f0 | denovo3505\_f0 | denovo3609\_f0 | denovo3635\_f0 | denovo3687\_f0 | denovo3739\_f0 | denovo3765\_f0 | denovo3791\_f0 | denovo3817\_f0 | denovo3843\_f0 | denovo3869\_f0 | denovo3973\_f0 | denovo3999\_f0 | denovo4077\_f0 | denovo4207\_f0 | denovo4337\_f0 | denovo4363\_f0 | denovo4389\_f0 | denovo4415\_f0 | denovo4571\_f0 | denovo4649\_f0 | denovo4753\_f0 | denovo4779\_f0 | denovo4805\_f0 | denovo4857\_f0 | denovo4909\_f0 | denovo4935\_f0 | denovo4961\_f0 | denovo4987\_f0 | denovo5013\_f0 | denovo5221\_f0 | denovo5247\_f0 | denovo5273\_f0 | denovo5455\_f0 | denovo5611\_f0 | denovo5663\_f0 | denovo5975\_f0 | denovo6027\_f0 | denovo6079\_f0 | denovo6105\_f0 | denovo6131\_f0 | denovo6209\_f0 | denovo6235\_f0 | denovo6261\_f0 | denovo6287\_f0 | denovo6313\_f0 | denovo6391\_f0 | denovo6521\_f0 | denovo6573\_f0 | denovo6599\_f0 | denovo6625\_f0 | denovo6677\_f0 | denovo6703\_f0 | denovo6729\_f0 | denovo6755\_f0 | denovo6781\_f0 | denovo6989\_f0 | denovo7015\_f0 | denovo7093\_f0 | denovo7145\_f0 | denovo7171\_f0 | denovo7275\_f0 | denovo7301\_f0 | denovo7327\_f0 | denovo7353\_f0 | denovo7379\_f0 | denovo7405\_f0 | denovo7509\_f0 | denovo7535\_f0 | denovo7561\_f0 | denovo7639\_f0 | denovo7691\_f0 | denovo7717\_f0 | denovo7847\_f0 | denovo7925\_f0 | denovo8055\_f0 | denovo8081\_f0 | denovo8237\_f0 | denovo8263\_f0 | denovo8289\_f0 | denovo8315\_f0 | denovo8393\_f0 | denovo8419\_f0 | denovo8445\_f0 | denovo8471\_f0 | denovo8575\_f0 | denovo8601\_f0 | denovo8653\_f0 | denovo8679\_f0 | denovo8705\_f0 | denovo8783\_f0 | denovo8861\_f0 | denovo8913\_f0 | denovo8939\_f0 | denovo8965\_f0 | denovo8991\_f0 | denovo9017\_f0 | denovo9147\_f0 | denovo9173\_f0 | denovo9199\_f0 | denovo9303\_f0 | denovo9329\_f0 | denovo9355\_f0 | denovo9381\_f0 | denovo9407\_f0 | denovo9459\_f0 | denovo9511\_f0 | denovo9719\_f0 | denovo9745\_f0 | denovo9797\_f0 | denovo9823\_f0 | denovo9849\_f0 | denovo9901\_f0 | denovo9979\_f0 | denovo10213\_f0 | denovo10239\_f0 | denovo10291\_f0 | denovo10317\_f0 | denovo10369\_f0 | denovo10421\_f0 | denovo10447\_f0 | denovo10473\_f0 | denovo10525\_f0 | denovo10551\_f0 | denovo10629\_f0 | denovo10681\_f0 | denovo10733\_f0 | denovo10759\_f0 | denovo10785\_f0 | denovo10837\_f0 | denovo10863\_f0 | denovo10941\_f0 | denovo11019\_f0 | denovo11045\_f0 | denovo11097\_f0 | denovo11175\_f0 | denovo11201\_f0 | denovo11305\_f0 | denovo11331\_f0 | denovo11357\_f0 | denovo11409\_f0 | denovo11435\_f0 | denovo11461\_f0 | denovo11487\_f0 | denovo11565\_f0 | denovo11721\_f0 | denovo11747\_f0 | denovo11929\_f0 | denovo12085\_f0 |
| FG120055B | Nothus lunus\* | denovo7\_f0 | denovo85\_f0 | denovo163\_f0 | denovo215\_f0 | denovo267\_f0 | denovo371\_f0 | denovo423\_f0 | denovo449\_f0 | denovo527\_f0 | denovo579\_f0 | denovo605\_f0 | denovo787\_f0 | denovo1047\_f0 | denovo1073\_f0 | denovo1151\_f0 | denovo1229\_f0 | denovo1359\_f0 | denovo1385\_f0 | denovo1411\_f0 | denovo1489\_f0 | denovo1515\_f0 | denovo1645\_f0 | denovo1671\_f0 | denovo1749\_f0 | denovo1801\_f0 | denovo1931\_f0 | denovo1957\_f0 | denovo1983\_f0 | denovo2009\_f0 | denovo2113\_f0 | denovo2139\_f0 | denovo2191\_f0 | denovo2243\_f0 | denovo2269\_f0 | denovo2295\_f0 | denovo2451\_f0 | denovo2477\_f0 | denovo2503\_f0 | denovo2529\_f0 | denovo2581\_f0 | denovo2607\_f0 | denovo2633\_f0 | denovo2659\_f0 | denovo2763\_f0 | denovo2841\_f0 | denovo2919\_f0 | denovo2997\_f0 | denovo3153\_f0 | denovo3257\_f0 | denovo3309\_f0 | denovo3335\_f0 | denovo3439\_f0 | denovo3491\_f0 | denovo3595\_f0 | denovo3621\_f0 | denovo3673\_f0 | denovo3725\_f0 | denovo3751\_f0 | denovo3777\_f0 | denovo3803\_f0 | denovo3829\_f0 | denovo3855\_f0 | denovo3959\_f0 | denovo3985\_f0 | denovo4063\_f0 | denovo4193\_f0 | denovo4323\_f0 | denovo4349\_f0 | denovo4375\_f0 | denovo4401\_f0 | denovo4557\_f0 | denovo4635\_f0 | denovo4739\_f0 | denovo4765\_f0 | denovo4791\_f0 | denovo4843\_f0 | denovo4895\_f0 | denovo4921\_f0 | denovo4947\_f0 | denovo4973\_f0 | denovo4999\_f0 | denovo5207\_f0 | denovo5233\_f0 | denovo5259\_f0 | denovo5441\_f0 | denovo5597\_f0 | denovo5649\_f0 | denovo5961\_f0 | denovo6013\_f0 | denovo6065\_f0 | denovo6091\_f0 | denovo6117\_f0 | denovo6195\_f0 | denovo6221\_f0 | denovo6247\_f0 | denovo6273\_f0 | denovo6299\_f0 | denovo6377\_f0 | denovo6507\_f0 | denovo6559\_f0 | denovo6585\_f0 | denovo6611\_f0 | denovo6663\_f0 | denovo6689\_f0 | denovo6715\_f0 | denovo6741\_f0 | denovo6767\_f0 | denovo6975\_f0 | denovo7001\_f0 | denovo7079\_f0 | denovo7131\_f0 | denovo7157\_f0 | denovo7261\_f0 | denovo7287\_f0 | denovo7313\_f0 | denovo7339\_f0 | denovo7365\_f0 | denovo7391\_f0 | denovo7495\_f0 | denovo7521\_f0 | denovo7547\_f0 | denovo7625\_f0 | denovo7677\_f0 | denovo7703\_f0 | denovo7833\_f0 | denovo7911\_f0 | denovo8041\_f0 | denovo8067\_f0 | denovo8223\_f0 | denovo8249\_f0 | denovo8275\_f0 | denovo8301\_f0 | denovo8379\_f0 | denovo8405\_f0 | denovo8431\_f0 | denovo8457\_f0 | denovo8561\_f0 | denovo8587\_f0 | denovo8639\_f0 | denovo8665\_f0 | denovo8691\_f0 | denovo8769\_f0 | denovo8847\_f0 | denovo8899\_f0 | denovo8925\_f0 | denovo8951\_f0 | denovo8977\_f0 | denovo9003\_f0 | denovo9133\_f0 | denovo9159\_f0 | denovo9185\_f0 | denovo9289\_f0 | denovo9315\_f0 | denovo9341\_f0 | denovo9367\_f0 | denovo9393\_f0 | denovo9445\_f0 | denovo9497\_f0 | denovo9705\_f0 | denovo9731\_f0 | denovo9783\_f0 | denovo9809\_f0 | denovo9835\_f0 | denovo9887\_f0 | denovo9965\_f0 | denovo10199\_f0 | denovo10225\_f0 | denovo10277\_f0 | denovo10303\_f0 | denovo10355\_f0 | denovo10407\_f0 | denovo10433\_f0 | denovo10459\_f0 | denovo10511\_f0 | denovo10537\_f0 | denovo10615\_f0 | denovo10667\_f0 | denovo10719\_f0 | denovo10745\_f0 | denovo10771\_f0 | denovo10823\_f0 | denovo10849\_f0 | denovo10927\_f0 | denovo11005\_f0 | denovo11031\_f0 | denovo11083\_f0 | denovo11161\_f0 | denovo11187\_f0 | denovo11291\_f0 | denovo11317\_f0 | denovo11343\_f0 | denovo11395\_f0 | denovo11421\_f0 | denovo11447\_f0 | denovo11473\_f0 | denovo11551\_f0 | denovo11707\_f0 | denovo11733\_f0 | denovo11915\_f0 | denovo12071\_f0 |
| FG120122 | Macrosoma sp.\* | denovo12\_f0 | denovo90\_f0 | denovo168\_f0 | denovo220\_f0 | denovo272\_f0 | denovo376\_f0 | denovo428\_f0 | denovo454\_f0 | denovo532\_f0 | denovo584\_f0 | denovo610\_f0 | denovo792\_f0 | denovo1052\_f0 | denovo1078\_f0 | denovo1156\_f0 | denovo1234\_f0 | denovo1364\_f0 | denovo1390\_f0 | denovo1416\_f0 | denovo1494\_f0 | denovo1520\_f0 | denovo1650\_f0 | denovo1676\_f0 | denovo1754\_f0 | denovo1806\_f0 | denovo1936\_f0 | denovo1962\_f0 | denovo1988\_f0 | denovo2014\_f0 | denovo2118\_f0 | denovo2144\_f0 | denovo2196\_f0 | denovo2248\_f0 | denovo2274\_f0 | denovo2300\_f0 | denovo2456\_f0 | denovo2482\_f0 | denovo2508\_f0 | denovo2534\_f0 | denovo2586\_f0 | denovo2612\_f0 | denovo2638\_f0 | denovo2664\_f0 | denovo2768\_f0 | denovo2846\_f0 | denovo2924\_f0 | denovo3002\_f0 | denovo3158\_f0 | denovo3262\_f0 | denovo3314\_f0 | denovo3340\_f0 | denovo3444\_f0 | denovo3496\_f0 | denovo3600\_f0 | denovo3626\_f0 | denovo3678\_f0 | denovo3730\_f0 | denovo3756\_f0 | denovo3782\_f0 | denovo3808\_f0 | denovo3834\_f0 | denovo3860\_f0 | denovo3964\_f0 | denovo3990\_f0 | denovo4068\_f0 | denovo4198\_f0 | denovo4328\_f0 | denovo4354\_f0 | denovo4380\_f0 | denovo4406\_f0 | denovo4562\_f0 | denovo4640\_f0 | denovo4744\_f0 | denovo4770\_f0 | denovo4796\_f0 | denovo4848\_f0 | denovo4900\_f0 | denovo4926\_f0 | denovo4952\_f0 | denovo4978\_f0 | denovo5004\_f0 | denovo5212\_f0 | denovo5238\_f0 | denovo5264\_f0 | denovo5446\_f0 | denovo5602\_f0 | denovo5654\_f0 | denovo5966\_f0 | denovo6018\_f0 | denovo6070\_f0 | denovo6096\_f0 | denovo6122\_f0 | denovo6200\_f0 | denovo6226\_f0 | denovo6252\_f0 | denovo6278\_f0 | denovo6304\_f0 | denovo6382\_f0 | denovo6512\_f0 | denovo6564\_f0 | denovo6590\_f0 | denovo6616\_f0 | denovo6668\_f0 | denovo6694\_f0 | denovo6720\_f0 | denovo6746\_f0 | denovo6772\_f0 | denovo6980\_f0 | denovo7006\_f0 | denovo7084\_f0 | denovo7136\_f0 | denovo7162\_f0 | denovo7266\_f0 | denovo7292\_f0 | denovo7318\_f0 | denovo7344\_f0 | denovo7370\_f0 | denovo7396\_f0 | denovo7500\_f0 | denovo7526\_f0 | denovo7552\_f0 | denovo7630\_f0 | denovo7682\_f0 | denovo7708\_f0 | denovo7838\_f0 | denovo7916\_f0 | denovo8046\_f0 | denovo8072\_f0 | denovo8228\_f0 | denovo8254\_f0 | denovo8280\_f0 | denovo8306\_f0 | denovo8384\_f0 | denovo8410\_f0 | denovo8436\_f0 | denovo8462\_f0 | denovo8566\_f0 | denovo8592\_f0 | denovo8644\_f0 | denovo8670\_f0 | denovo8696\_f0 | denovo8774\_f0 | denovo8852\_f0 | denovo8904\_f0 | denovo8930\_f0 | denovo8956\_f0 | denovo8982\_f0 | denovo9008\_f0 | denovo9138\_f0 | denovo9164\_f0 | denovo9190\_f0 | denovo9294\_f0 | denovo9320\_f0 | denovo9346\_f0 | denovo9372\_f0 | denovo9398\_f0 | denovo9450\_f0 | denovo9502\_f0 | denovo9710\_f0 | denovo9736\_f0 | denovo9788\_f0 | denovo9814\_f0 | denovo9840\_f0 | denovo9892\_f0 | denovo9970\_f0 | denovo10204\_f0 | denovo10230\_f0 | denovo10282\_f0 | denovo10308\_f0 | denovo10360\_f0 | denovo10412\_f0 | denovo10438\_f0 | denovo10464\_f0 | denovo10516\_f0 | denovo10542\_f0 | denovo10620\_f0 | denovo10672\_f0 | denovo10724\_f0 | denovo10750\_f0 | denovo10776\_f0 | denovo10828\_f0 | denovo10854\_f0 | denovo10932\_f0 | denovo11010\_f0 | denovo11036\_f0 | denovo11088\_f0 | denovo11166\_f0 | denovo11192\_f0 | denovo11296\_f0 | denovo11322\_f0 | denovo11348\_f0 | denovo11400\_f0 | denovo11426\_f0 | denovo11452\_f0 | denovo11478\_f0 | denovo11556\_f0 | denovo11712\_f0 | denovo11738\_f0 | denovo11920\_f0 | denovo12076\_f0 |
| FG120024 | Megalopyge tharops\* | denovo4\_f0 | denovo82\_f0 | denovo160\_f0 | denovo212\_f0 | denovo264\_f0 | denovo368\_f0 | denovo420\_f0 | denovo446\_f0 | denovo524\_f0 | denovo576\_f0 | denovo602\_f0 | denovo784\_f0 | denovo1044\_f0 | denovo1070\_f0 | denovo1148\_f0 | denovo1226\_f0 | denovo1356\_f0 | denovo1382\_f0 | denovo1408\_f0 | denovo1486\_f0 | denovo1512\_f0 | denovo1642\_f0 | denovo1668\_f0 | denovo1746\_f0 | denovo1798\_f0 | denovo1928\_f0 | denovo1954\_f0 | denovo1980\_f0 | denovo2006\_f0 | denovo2110\_f0 | denovo2136\_f0 | denovo2188\_f0 | denovo2240\_f0 | denovo2266\_f0 | denovo2292\_f0 | denovo2448\_f0 | denovo2474\_f0 | denovo2500\_f0 | denovo2526\_f0 | denovo2578\_f0 | denovo2604\_f0 | denovo2630\_f0 | denovo2656\_f0 | denovo2760\_f0 | denovo2838\_f0 | denovo2916\_f0 | denovo2994\_f0 | denovo3150\_f0 | denovo3254\_f0 | denovo3306\_f0 | denovo3332\_f0 | denovo3436\_f0 | denovo3488\_f0 | denovo3592\_f0 | denovo3618\_f0 | denovo3670\_f0 | denovo3722\_f0 | denovo3748\_f0 | denovo3774\_f0 | denovo3800\_f0 | denovo3826\_f0 | denovo3852\_f0 | denovo3956\_f0 | denovo3982\_f0 | denovo4060\_f0 | denovo4190\_f0 | denovo4320\_f0 | denovo4346\_f0 | denovo4372\_f0 | denovo4398\_f0 | denovo4554\_f0 | denovo4632\_f0 | denovo4736\_f0 | denovo4762\_f0 | denovo4788\_f0 | denovo4840\_f0 | denovo4892\_f0 | denovo4918\_f0 | denovo4944\_f0 | denovo4970\_f0 | denovo4996\_f0 | denovo5204\_f0 | denovo5230\_f0 | denovo5256\_f0 | denovo5438\_f0 | denovo5594\_f0 | denovo5646\_f0 | denovo5958\_f0 | denovo6010\_f0 | denovo6062\_f0 | denovo6088\_f0 | denovo6114\_f0 | denovo6192\_f0 | denovo6218\_f0 | denovo6244\_f0 | denovo6270\_f0 | denovo6296\_f0 | denovo6374\_f0 | denovo6504\_f0 | denovo6556\_f0 | denovo6582\_f0 | denovo6608\_f0 | denovo6660\_f0 | denovo6686\_f0 | denovo6712\_f0 | denovo6738\_f0 | denovo6764\_f0 | denovo6972\_f0 | denovo6998\_f0 | denovo7076\_f0 | denovo7128\_f0 | denovo7154\_f0 | denovo7258\_f0 | denovo7284\_f0 | denovo7310\_f0 | denovo7336\_f0 | denovo7362\_f0 | denovo7388\_f0 | denovo7492\_f0 | denovo7518\_f0 | denovo7544\_f0 | denovo7622\_f0 | denovo7674\_f0 | denovo7700\_f0 | denovo7830\_f0 | denovo7908\_f0 | denovo8038\_f0 | denovo8064\_f0 | denovo8220\_f0 | denovo8246\_f0 | denovo8272\_f0 | denovo8298\_f0 | denovo8376\_f0 | denovo8402\_f0 | denovo8428\_f0 | denovo8454\_f0 | denovo8558\_f0 | denovo8584\_f0 | denovo8636\_f0 | denovo8662\_f0 | denovo8688\_f0 | denovo8766\_f0 | denovo8844\_f0 | denovo8896\_f0 | denovo8922\_f0 | denovo8948\_f0 | denovo8974\_f0 | denovo9000\_f0 | denovo9130\_f0 | denovo9156\_f0 | denovo9182\_f0 | denovo9286\_f0 | denovo9312\_f0 | denovo9338\_f0 | denovo9364\_f0 | denovo9390\_f0 | denovo9442\_f0 | denovo9494\_f0 | denovo9702\_f0 | denovo9728\_f0 | denovo9780\_f0 | denovo9806\_f0 | denovo9832\_f0 | denovo9884\_f0 | denovo9962\_f0 | denovo10196\_f0 | denovo10222\_f0 | denovo10274\_f0 | denovo10300\_f0 | denovo10352\_f0 | denovo10404\_f0 | denovo10430\_f0 | denovo10456\_f0 | denovo10508\_f0 | denovo10534\_f0 | denovo10612\_f0 | denovo10664\_f0 | denovo10716\_f0 | denovo10742\_f0 | denovo10768\_f0 | denovo10820\_f0 | denovo10846\_f0 | denovo10924\_f0 | denovo11002\_f0 | denovo11028\_f0 | denovo11080\_f0 | denovo11158\_f0 | denovo11184\_f0 | denovo11288\_f0 | denovo11314\_f0 | denovo11340\_f0 | denovo11392\_f0 | denovo11418\_f0 | denovo11444\_f0 | denovo11470\_f0 | denovo11548\_f0 | denovo11704\_f0 | denovo11730\_f0 | denovo11912\_f0 | denovo12068\_f0 |
| FG120022 | Morpheis mathani\* | denovo3\_f0 | denovo81\_f0 | denovo159\_f0 | denovo211\_f0 | denovo263\_f0 | denovo367\_f0 | denovo419\_f0 | denovo445\_f0 | denovo523\_f0 | denovo575\_f0 | denovo601\_f0 | denovo783\_f0 | denovo1043\_f0 | denovo1069\_f0 | denovo1147\_f0 | denovo1225\_f0 | denovo1355\_f0 | denovo1381\_f0 | denovo1407\_f0 | denovo1485\_f0 | denovo1511\_f0 | denovo1641\_f0 | denovo1667\_f0 | denovo1745\_f0 | denovo1797\_f0 | denovo1927\_f0 | denovo1953\_f0 | denovo1979\_f0 | denovo2005\_f0 | denovo2109\_f0 | denovo2135\_f0 | denovo2187\_f0 | denovo2239\_f0 | denovo2265\_f0 | denovo2291\_f0 | denovo2447\_f0 | denovo2473\_f0 | denovo2499\_f0 | denovo2525\_f0 | denovo2577\_f0 | denovo2603\_f0 | denovo2629\_f0 | denovo2655\_f0 | denovo2759\_f0 | denovo2837\_f0 | denovo2915\_f0 | denovo2993\_f0 | denovo3149\_f0 | denovo3253\_f0 | denovo3305\_f0 | denovo3331\_f0 | denovo3435\_f0 | denovo3487\_f0 | denovo3591\_f0 | denovo3617\_f0 | denovo3669\_f0 | denovo3721\_f0 | denovo3747\_f0 | denovo3773\_f0 | denovo3799\_f0 | denovo3825\_f0 | denovo3851\_f0 | denovo3955\_f0 | denovo3981\_f0 | denovo4059\_f0 | denovo4189\_f0 | denovo4319\_f0 | denovo4345\_f0 | denovo4371\_f0 | denovo4397\_f0 | denovo4553\_f0 | denovo4631\_f0 | denovo4735\_f0 | denovo4761\_f0 | denovo4787\_f0 | denovo4839\_f0 | denovo4891\_f0 | denovo4917\_f0 | denovo4943\_f0 | denovo4969\_f0 | denovo4995\_f0 | denovo5203\_f0 | denovo5229\_f0 | denovo5255\_f0 | denovo5437\_f0 | denovo5593\_f0 | denovo5645\_f0 | denovo5957\_f0 | denovo6009\_f0 | denovo6061\_f0 | denovo6087\_f0 | denovo6113\_f0 | denovo6191\_f0 | denovo6217\_f0 | denovo6243\_f0 | denovo6269\_f0 | denovo6295\_f0 | denovo6373\_f0 | denovo6503\_f0 | denovo6555\_f0 | denovo6581\_f0 | denovo6607\_f0 | denovo6659\_f0 | denovo6685\_f0 | denovo6711\_f0 | denovo6737\_f0 | denovo6763\_f0 | denovo6971\_f0 | denovo6997\_f0 | denovo7075\_f0 | denovo7127\_f0 | denovo7153\_f0 | denovo7257\_f0 | denovo7283\_f0 | denovo7309\_f0 | denovo7335\_f0 | denovo7361\_f0 | denovo7387\_f0 | denovo7491\_f0 | denovo7517\_f0 | denovo7543\_f0 | denovo7621\_f0 | denovo7673\_f0 | denovo7699\_f0 | denovo7829\_f0 | denovo7907\_f0 | denovo8037\_f0 | denovo8063\_f0 | denovo8219\_f0 | denovo8245\_f0 | denovo8271\_f0 | denovo8297\_f0 | denovo8375\_f0 | denovo8401\_f0 | denovo8427\_f0 | denovo8453\_f0 | denovo8557\_f0 | denovo8583\_f0 | denovo8635\_f0 | denovo8661\_f0 | denovo8687\_f0 | denovo8765\_f0 | denovo8843\_f0 | denovo8895\_f0 | denovo8921\_f0 | denovo8947\_f0 | denovo8973\_f0 | denovo8999\_f0 | denovo9129\_f0 | denovo9155\_f0 | denovo9181\_f0 | denovo9285\_f0 | denovo9311\_f0 | denovo9337\_f0 | denovo9363\_f0 | denovo9389\_f0 | denovo9441\_f0 | denovo9493\_f0 | denovo9701\_f0 | denovo9727\_f0 | denovo9779\_f0 | denovo9805\_f0 | denovo9831\_f0 | denovo9883\_f0 | denovo9961\_f0 | denovo10195\_f0 | denovo10221\_f0 | denovo10273\_f0 | denovo10299\_f0 | denovo10351\_f0 | denovo10403\_f0 | denovo10429\_f0 | denovo10455\_f0 | denovo10507\_f0 | denovo10533\_f0 | denovo10611\_f0 | denovo10663\_f0 | denovo10715\_f0 | denovo10741\_f0 | denovo10767\_f0 | denovo10819\_f0 | denovo10845\_f0 | denovo10923\_f0 | denovo11001\_f0 | denovo11027\_f0 | denovo11079\_f0 | denovo11157\_f0 | denovo11183\_f0 | denovo11287\_f0 | denovo11313\_f0 | denovo11339\_f0 | denovo11391\_f0 | denovo11417\_f0 | denovo11443\_f0 | denovo11469\_f0 | denovo11547\_f0 | denovo11703\_f0 | denovo11729\_f0 | denovo11911\_f0 | denovo12067\_f0 |
| GNV139000 | Megathymus yuccae\* | denovo16\_f0 | denovo94\_f0 | denovo172\_f0 | denovo224\_f0 | denovo276\_f0 | denovo380\_f0 | denovo432\_f0 | denovo458\_f0 | denovo536\_f0 | denovo588\_f0 | denovo614\_f0 | denovo796\_f0 | denovo1056\_f0 | denovo1082\_f0 | denovo1160\_f0 | denovo1238\_f0 | denovo1368\_f0 | denovo1394\_f0 | denovo1420\_f0 | denovo1498\_f0 | denovo1524\_f0 | denovo1654\_f0 | denovo1680\_f0 | denovo1758\_f0 | denovo1810\_f0 | denovo1940\_f0 | denovo1966\_f0 | denovo1992\_f0 | denovo2018\_f0 | denovo2122\_f0 | denovo2148\_f0 | denovo2200\_f0 | denovo2252\_f0 | denovo2278\_f0 | denovo2304\_f0 | denovo2460\_f0 | denovo2486\_f0 | denovo2512\_f0 | denovo2538\_f0 | denovo2590\_f0 | denovo2616\_f0 | denovo2642\_f0 | denovo2668\_f0 | denovo2772\_f0 | denovo2850\_f0 | denovo2928\_f0 | denovo3006\_f0 | denovo3162\_f0 | denovo3266\_f0 | denovo3318\_f0 | denovo3344\_f0 | denovo3448\_f0 | denovo3500\_f0 | denovo3604\_f0 | denovo3630\_f0 | denovo3682\_f0 | denovo3734\_f0 | denovo3760\_f0 | denovo3786\_f0 | denovo3812\_f0 | denovo3838\_f0 | denovo3864\_f0 | denovo3968\_f0 | denovo3994\_f0 | denovo4072\_f0 | denovo4202\_f0 | denovo4332\_f0 | denovo4358\_f0 | denovo4384\_f0 | denovo4410\_f0 | denovo4566\_f0 | denovo4644\_f0 | denovo4748\_f0 | denovo4774\_f0 | denovo4800\_f0 | denovo4852\_f0 | denovo4904\_f0 | denovo4930\_f0 | denovo4956\_f0 | denovo4982\_f0 | denovo5008\_f0 | denovo5216\_f0 | denovo5242\_f0 | denovo5268\_f0 | denovo5450\_f0 | denovo5606\_f0 | denovo5658\_f0 | denovo5970\_f0 | denovo6022\_f0 | denovo6074\_f0 | denovo6100\_f0 | denovo6126\_f0 | denovo6204\_f0 | denovo6230\_f0 | denovo6256\_f0 | denovo6282\_f0 | denovo6308\_f0 | denovo6386\_f0 | denovo6516\_f0 | denovo6568\_f0 | denovo6594\_f0 | denovo6620\_f0 | denovo6672\_f0 | denovo6698\_f0 | denovo6724\_f0 | denovo6750\_f0 | denovo6776\_f0 | denovo6984\_f0 | denovo7010\_f0 | denovo7088\_f0 | denovo7140\_f0 | denovo7166\_f0 | denovo7270\_f0 | denovo7296\_f0 | denovo7322\_f0 | denovo7348\_f0 | denovo7374\_f0 | denovo7400\_f0 | denovo7504\_f0 | denovo7530\_f0 | denovo7556\_f0 | denovo7634\_f0 | denovo7686\_f0 | denovo7712\_f0 | denovo7842\_f0 | denovo7920\_f0 | denovo8050\_f0 | denovo8076\_f0 | denovo8232\_f0 | denovo8258\_f0 | denovo8284\_f0 | denovo8310\_f0 | denovo8388\_f0 | denovo8414\_f0 | denovo8440\_f0 | denovo8466\_f0 | denovo8570\_f0 | denovo8596\_f0 | denovo8648\_f0 | denovo8674\_f0 | denovo8700\_f0 | denovo8778\_f0 | denovo8856\_f0 | denovo8908\_f0 | denovo8934\_f0 | denovo8960\_f0 | denovo8986\_f0 | denovo9012\_f0 | denovo9142\_f0 | denovo9168\_f0 | denovo9194\_f0 | denovo9298\_f0 | denovo9324\_f0 | denovo9350\_f0 | denovo9376\_f0 | denovo9402\_f0 | denovo9454\_f0 | denovo9506\_f0 | denovo9714\_f0 | denovo9740\_f0 | denovo9792\_f0 | denovo9818\_f0 | denovo9844\_f0 | denovo9896\_f0 | denovo9974\_f0 | denovo10208\_f0 | denovo10234\_f0 | denovo10286\_f0 | denovo10312\_f0 | denovo10364\_f0 | denovo10416\_f0 | denovo10442\_f0 | denovo10468\_f0 | denovo10520\_f0 | denovo10546\_f0 | denovo10624\_f0 | denovo10676\_f0 | denovo10728\_f0 | denovo10754\_f0 | denovo10780\_f0 | denovo10832\_f0 | denovo10858\_f0 | denovo10936\_f0 | denovo11014\_f0 | denovo11040\_f0 | denovo11092\_f0 | denovo11170\_f0 | denovo11196\_f0 | denovo11300\_f0 | denovo11326\_f0 | denovo11352\_f0 | denovo11404\_f0 | denovo11430\_f0 | denovo11456\_f0 | denovo11482\_f0 | denovo11560\_f0 | denovo11716\_f0 | denovo11742\_f0 | denovo11924\_f0 | denovo12080\_f0 |
| Msexta | Manduca sexta\* | denovo17\_f0 | denovo95\_f0 | denovo173\_f0 | denovo225\_f0 | denovo277\_f0 | denovo381\_f0 | denovo433\_f0 | denovo459\_f0 | denovo537\_f0 | denovo589\_f0 | denovo615\_f0 | denovo797\_f0 | denovo1057\_f0 | denovo1083\_f0 | denovo1161\_f0 | denovo1239\_f0 | denovo1369\_f0 | denovo1395\_f0 | denovo1421\_f0 | denovo1499\_f0 | denovo1525\_f0 | denovo1655\_f0 | denovo1681\_f0 | denovo1759\_f0 | denovo1811\_f0 | denovo1941\_f0 | denovo1967\_f0 | denovo1993\_f0 | denovo2019\_f0 | denovo2123\_f0 | denovo2149\_f0 | denovo2201\_f0 | denovo2253\_f0 | denovo2279\_f0 | denovo2305\_f0 | denovo2461\_f0 | denovo2487\_f0 | denovo2513\_f0 | denovo2539\_f0 | denovo2591\_f0 | denovo2617\_f0 | denovo2643\_f0 | denovo2669\_f0 | denovo2773\_f0 | denovo2851\_f0 | denovo2929\_f0 | denovo3007\_f0 | denovo3163\_f0 | denovo3267\_f0 | denovo3319\_f0 | denovo3345\_f0 | denovo3449\_f0 | denovo3501\_f0 | denovo3605\_f0 | denovo3631\_f0 | denovo3683\_f0 | denovo3735\_f0 | denovo3761\_f0 | denovo3787\_f0 | denovo3813\_f0 | denovo3839\_f0 | denovo3865\_f0 | denovo3969\_f0 | denovo3995\_f0 | denovo4073\_f0 | denovo4203\_f0 | denovo4333\_f0 | denovo4359\_f0 | denovo4385\_f0 | denovo4411\_f0 | denovo4567\_f0 | denovo4645\_f0 | denovo4749\_f0 | denovo4775\_f0 | denovo4801\_f0 | denovo4853\_f0 | denovo4905\_f0 | denovo4931\_f0 | denovo4957\_f0 | denovo4983\_f0 | denovo5009\_f0 | denovo5217\_f0 | denovo5243\_f0 | denovo5269\_f0 | denovo5451\_f0 | denovo5607\_f0 | denovo5659\_f0 | denovo5971\_f0 | denovo6023\_f0 | denovo6075\_f0 | denovo6101\_f0 | denovo6127\_f0 | denovo6205\_f0 | denovo6231\_f0 | denovo6257\_f0 | denovo6283\_f0 | denovo6309\_f0 | denovo6387\_f0 | denovo6517\_f0 | denovo6569\_f0 | denovo6595\_f0 | denovo6621\_f0 | denovo6673\_f0 | denovo6699\_f0 | denovo6725\_f0 | denovo6751\_f0 | denovo6777\_f0 | denovo6985\_f0 | denovo7011\_f0 | denovo7089\_f0 | denovo7141\_f0 | denovo7167\_f0 | denovo7271\_f0 | denovo7297\_f0 | denovo7323\_f0 | denovo7349\_f0 | denovo7375\_f0 | denovo7401\_f0 | denovo7505\_f0 | denovo7531\_f0 | denovo7557\_f0 | denovo7635\_f0 | denovo7687\_f0 | denovo7713\_f0 | denovo7843\_f0 | denovo7921\_f0 | denovo8051\_f0 | denovo8077\_f0 | denovo8233\_f0 | denovo8259\_f0 | denovo8285\_f0 | denovo8311\_f0 | denovo8389\_f0 | denovo8415\_f0 | denovo8441\_f0 | denovo8467\_f0 | denovo8571\_f0 | denovo8597\_f0 | denovo8649\_f0 | denovo8675\_f0 | denovo8701\_f0 | denovo8779\_f0 | denovo8857\_f0 | denovo8909\_f0 | denovo8935\_f0 | denovo8961\_f0 | denovo8987\_f0 | denovo9013\_f0 | denovo9143\_f0 | denovo9169\_f0 | denovo9195\_f0 | denovo9299\_f0 | denovo9325\_f0 | denovo9351\_f0 | denovo9377\_f0 | denovo9403\_f0 | denovo9455\_f0 | denovo9507\_f0 | denovo9715\_f0 | denovo9741\_f0 | denovo9793\_f0 | denovo9819\_f0 | denovo9845\_f0 | denovo9897\_f0 | denovo9975\_f0 | denovo10209\_f0 | denovo10235\_f0 | denovo10287\_f0 | denovo10313\_f0 | denovo10365\_f0 | denovo10417\_f0 | denovo10443\_f0 | denovo10469\_f0 | denovo10521\_f0 | denovo10547\_f0 | denovo10625\_f0 | denovo10677\_f0 | denovo10729\_f0 | denovo10755\_f0 | denovo10781\_f0 | denovo10833\_f0 | denovo10859\_f0 | denovo10937\_f0 | denovo11015\_f0 | denovo11041\_f0 | denovo11093\_f0 | denovo11171\_f0 | denovo11197\_f0 | denovo11301\_f0 | denovo11327\_f0 | denovo11353\_f0 | denovo11405\_f0 | denovo11431\_f0 | denovo11457\_f0 | denovo11483\_f0 | denovo11561\_f0 | denovo11717\_f0 | denovo11743\_f0 | denovo11925\_f0 | denovo12081\_f0 |
| acti2 | Actias luna\* | denovo25\_f0 | denovo103\_f0 | denovo181\_f0 | denovo233\_f0 | denovo285\_f0 | denovo389\_f0 | denovo441\_f0 | denovo467\_f0 | denovo545\_f0 | denovo597\_f0 | denovo623\_f0 | denovo805\_f0 | denovo1065\_f0 | denovo1091\_f0 | denovo1169\_f0 | denovo1247\_f0 | denovo1377\_f0 | denovo1403\_f0 | denovo1429\_f0 | denovo1507\_f0 | denovo1533\_f0 | denovo1663\_f0 | denovo1689\_f0 | denovo1767\_f0 | denovo1819\_f0 | denovo1949\_f0 | denovo1975\_f0 | denovo2001\_f0 | denovo2027\_f0 | denovo2131\_f0 | denovo2157\_f0 | denovo2209\_f0 | denovo2261\_f0 | denovo2287\_f0 | denovo2313\_f0 | denovo2469\_f0 | denovo2495\_f0 | denovo2521\_f0 | denovo2547\_f0 | denovo2599\_f0 | denovo2625\_f0 | denovo2651\_f0 | denovo2677\_f0 | denovo2781\_f0 | denovo2859\_f0 | denovo2937\_f0 | denovo3015\_f0 | denovo3171\_f0 | denovo3275\_f0 | denovo3327\_f0 | denovo3353\_f0 | denovo3457\_f0 | denovo3509\_f0 | denovo3613\_f0 | denovo3639\_f0 | denovo3691\_f0 | denovo3743\_f0 | denovo3769\_f0 | denovo3795\_f0 | denovo3821\_f0 | denovo3847\_f0 | denovo3873\_f0 | denovo3977\_f0 | denovo4003\_f0 | denovo4081\_f0 | denovo4211\_f0 | denovo4341\_f0 | denovo4367\_f0 | denovo4393\_f0 | denovo4419\_f0 | denovo4575\_f0 | denovo4653\_f0 | denovo4757\_f0 | denovo4783\_f0 | denovo4809\_f0 | denovo4861\_f0 | denovo4913\_f0 | denovo4939\_f0 | denovo4965\_f0 | denovo4991\_f0 | denovo5017\_f0 | denovo5225\_f0 | denovo5251\_f0 | denovo5277\_f0 | denovo5459\_f0 | denovo5615\_f0 | denovo5667\_f0 | denovo5979\_f0 | denovo6031\_f0 | denovo6083\_f0 | denovo6109\_f0 | denovo6135\_f0 | denovo6213\_f0 | denovo6239\_f0 | denovo6265\_f0 | denovo6291\_f0 | denovo6317\_f0 | denovo6395\_f0 | denovo6525\_f0 | denovo6577\_f0 | denovo6603\_f0 | denovo6629\_f0 | denovo6681\_f0 | denovo6707\_f0 | denovo6733\_f0 | denovo6759\_f0 | denovo6785\_f0 | denovo6993\_f0 | denovo7019\_f0 | denovo7097\_f0 | denovo7149\_f0 | denovo7175\_f0 | denovo7279\_f0 | denovo7305\_f0 | denovo7331\_f0 | denovo7357\_f0 | denovo7383\_f0 | denovo7409\_f0 | denovo7513\_f0 | denovo7539\_f0 | denovo7565\_f0 | denovo7643\_f0 | denovo7695\_f0 | denovo7721\_f0 | denovo7851\_f0 | denovo7929\_f0 | denovo8059\_f0 | denovo8085\_f0 | denovo8241\_f0 | denovo8267\_f0 | denovo8293\_f0 | denovo8319\_f0 | denovo8397\_f0 | denovo8423\_f0 | denovo8449\_f0 | denovo8475\_f0 | denovo8579\_f0 | denovo8605\_f0 | denovo8657\_f0 | denovo8683\_f0 | denovo8709\_f0 | denovo8787\_f0 | denovo8865\_f0 | denovo8917\_f0 | denovo8943\_f0 | denovo8969\_f0 | denovo8995\_f0 | denovo9021\_f0 | denovo9151\_f0 | denovo9177\_f0 | denovo9203\_f0 | denovo9307\_f0 | denovo9333\_f0 | denovo9359\_f0 | denovo9385\_f0 | denovo9411\_f0 | denovo9463\_f0 | denovo9515\_f0 | denovo9723\_f0 | denovo9749\_f0 | denovo9801\_f0 | denovo9827\_f0 | denovo9853\_f0 | denovo9905\_f0 | denovo9983\_f0 | denovo10217\_f0 | denovo10243\_f0 | denovo10295\_f0 | denovo10321\_f0 | denovo10373\_f0 | denovo10425\_f0 | denovo10451\_f0 | denovo10477\_f0 | denovo10529\_f0 | denovo10555\_f0 | denovo10633\_f0 | denovo10685\_f0 | denovo10737\_f0 | denovo10763\_f0 | denovo10789\_f0 | denovo10841\_f0 | denovo10867\_f0 | denovo10945\_f0 | denovo11023\_f0 | denovo11049\_f0 | denovo11101\_f0 | denovo11179\_f0 | denovo11205\_f0 | denovo11309\_f0 | denovo11335\_f0 | denovo11361\_f0 | denovo11413\_f0 | denovo11439\_f0 | denovo11465\_f0 | denovo11491\_f0 | denovo11569\_f0 | denovo11725\_f0 | denovo11751\_f0 | denovo11933\_f0 | denovo12089\_f0 |
| FG120071B | Myelobia sp.\* | denovo9\_f0 | denovo87\_f0 | denovo165\_f0 | denovo217\_f0 | denovo269\_f0 | denovo373\_f0 | denovo425\_f0 | denovo451\_f0 | denovo529\_f0 | denovo581\_f0 | denovo607\_f0 | denovo789\_f0 | denovo1049\_f0 | denovo1075\_f0 | denovo1153\_f0 | denovo1231\_f0 | denovo1361\_f0 | denovo1387\_f0 | denovo1413\_f0 | denovo1491\_f0 | denovo1517\_f0 | denovo1647\_f0 | denovo1673\_f0 | denovo1751\_f0 | denovo1803\_f0 | denovo1933\_f0 | denovo1959\_f0 | denovo1985\_f0 | denovo2011\_f0 | denovo2115\_f0 | denovo2141\_f0 | denovo2193\_f0 | denovo2245\_f0 | denovo2271\_f0 | denovo2297\_f0 | denovo2453\_f0 | denovo2479\_f0 | denovo2505\_f0 | denovo2531\_f0 | denovo2583\_f0 | denovo2609\_f0 | denovo2635\_f0 | denovo2661\_f0 | denovo2765\_f0 | denovo2843\_f0 | denovo2921\_f0 | denovo2999\_f0 | denovo3155\_f0 | denovo3259\_f0 | denovo3311\_f0 | denovo3337\_f0 | denovo3441\_f0 | denovo3493\_f0 | denovo3597\_f0 | denovo3623\_f0 | denovo3675\_f0 | denovo3727\_f0 | denovo3753\_f0 | denovo3779\_f0 | denovo3805\_f0 | denovo3831\_f0 | denovo3857\_f0 | denovo3961\_f0 | denovo3987\_f0 | denovo4065\_f0 | denovo4195\_f0 | denovo4325\_f0 | denovo4351\_f0 | denovo4377\_f0 | denovo4403\_f0 | denovo4559\_f0 | denovo4637\_f0 | denovo4741\_f0 | denovo4767\_f0 | denovo4793\_f0 | denovo4845\_f0 | denovo4897\_f0 | denovo4923\_f0 | denovo4949\_f0 | denovo4975\_f0 | denovo5001\_f0 | denovo5209\_f0 | denovo5235\_f0 | denovo5261\_f0 | denovo5443\_f0 | denovo5599\_f0 | denovo5651\_f0 | denovo5963\_f0 | denovo6015\_f0 | denovo6067\_f0 | denovo6093\_f0 | denovo6119\_f0 | denovo6197\_f0 | denovo6223\_f0 | denovo6249\_f0 | denovo6275\_f0 | denovo6301\_f0 | denovo6379\_f0 | denovo6509\_f0 | denovo6561\_f0 | denovo6587\_f0 | denovo6613\_f0 | denovo6665\_f0 | denovo6691\_f0 | denovo6717\_f0 | denovo6743\_f0 | denovo6769\_f0 | denovo6977\_f0 | denovo7003\_f0 | denovo7081\_f0 | denovo7133\_f0 | denovo7159\_f0 | denovo7263\_f0 | denovo7289\_f0 | denovo7315\_f0 | denovo7341\_f0 | denovo7367\_f0 | denovo7393\_f0 | denovo7497\_f0 | denovo7523\_f0 | denovo7549\_f0 | denovo7627\_f0 | denovo7679\_f0 | denovo7705\_f0 | denovo7835\_f0 | denovo7913\_f0 | denovo8043\_f0 | denovo8069\_f0 | denovo8225\_f0 | denovo8251\_f0 | denovo8277\_f0 | denovo8303\_f0 | denovo8381\_f0 | denovo8407\_f0 | denovo8433\_f0 | denovo8459\_f0 | denovo8563\_f0 | denovo8589\_f0 | denovo8641\_f0 | denovo8667\_f0 | denovo8693\_f0 | denovo8771\_f0 | denovo8849\_f0 | denovo8901\_f0 | denovo8927\_f0 | denovo8953\_f0 | denovo8979\_f0 | denovo9005\_f0 | denovo9135\_f0 | denovo9161\_f0 | denovo9187\_f0 | denovo9291\_f0 | denovo9317\_f0 | denovo9343\_f0 | denovo9369\_f0 | denovo9395\_f0 | denovo9447\_f0 | denovo9499\_f0 | denovo9707\_f0 | denovo9733\_f0 | denovo9785\_f0 | denovo9811\_f0 | denovo9837\_f0 | denovo9889\_f0 | denovo9967\_f0 | denovo10201\_f0 | denovo10227\_f0 | denovo10279\_f0 | denovo10305\_f0 | denovo10357\_f0 | denovo10409\_f0 | denovo10435\_f0 | denovo10461\_f0 | denovo10513\_f0 | denovo10539\_f0 | denovo10617\_f0 | denovo10669\_f0 | denovo10721\_f0 | denovo10747\_f0 | denovo10773\_f0 | denovo10825\_f0 | denovo10851\_f0 | denovo10929\_f0 | denovo11007\_f0 | denovo11033\_f0 | denovo11085\_f0 | denovo11163\_f0 | denovo11189\_f0 | denovo11293\_f0 | denovo11319\_f0 | denovo11345\_f0 | denovo11397\_f0 | denovo11423\_f0 | denovo11449\_f0 | denovo11475\_f0 | denovo11553\_f0 | denovo11709\_f0 | denovo11735\_f0 | denovo11917\_f0 | denovo12073\_f0 |
| GNV120027 | Lantanophaga pusillidactyla\* | denovo13\_f0 | denovo91\_f0 | denovo169\_f0 | denovo221\_f0 | denovo273\_f0 | denovo377\_f0 | denovo429\_f0 | denovo455\_f0 | denovo533\_f0 | denovo585\_f0 | denovo611\_f0 | denovo793\_f0 | denovo1053\_f0 | denovo1079\_f0 | denovo1157\_f0 | denovo1235\_f0 | denovo1365\_f0 | denovo1391\_f0 | denovo1417\_f0 | denovo1495\_f0 | denovo1521\_f0 | denovo1651\_f0 | denovo1677\_f0 | denovo1755\_f0 | denovo1807\_f0 | denovo1937\_f0 | denovo1963\_f0 | denovo1989\_f0 | denovo2015\_f0 | denovo2119\_f0 | denovo2145\_f0 | denovo2197\_f0 | denovo2249\_f0 | denovo2275\_f0 | denovo2301\_f0 | denovo2457\_f0 | denovo2483\_f0 | denovo2509\_f0 | denovo2535\_f0 | denovo2587\_f0 | denovo2613\_f0 | denovo2639\_f0 | denovo2665\_f0 | denovo2769\_f0 | denovo2847\_f0 | denovo2925\_f0 | denovo3003\_f0 | denovo3159\_f0 | denovo3263\_f0 | denovo3315\_f0 | denovo3341\_f0 | denovo3445\_f0 | denovo3497\_f0 | denovo3601\_f0 | denovo3627\_f0 | denovo3679\_f0 | denovo3731\_f0 | denovo3757\_f0 | denovo3783\_f0 | denovo3809\_f0 | denovo3835\_f0 | denovo3861\_f0 | denovo3965\_f0 | denovo3991\_f0 | denovo4069\_f0 | denovo4199\_f0 | denovo4329\_f0 | denovo4355\_f0 | denovo4381\_f0 | denovo4407\_f0 | denovo4563\_f0 | denovo4641\_f0 | denovo4745\_f0 | denovo4771\_f0 | denovo4797\_f0 | denovo4849\_f0 | denovo4901\_f0 | denovo4927\_f0 | denovo4953\_f0 | denovo4979\_f0 | denovo5005\_f0 | denovo5213\_f0 | denovo5239\_f0 | denovo5265\_f0 | denovo5447\_f0 | denovo5603\_f0 | denovo5655\_f0 | denovo5967\_f0 | denovo6019\_f0 | denovo6071\_f0 | denovo6097\_f0 | denovo6123\_f0 | denovo6201\_f0 | denovo6227\_f0 | denovo6253\_f0 | denovo6279\_f0 | denovo6305\_f0 | denovo6383\_f0 | denovo6513\_f0 | denovo6565\_f0 | denovo6591\_f0 | denovo6617\_f0 | denovo6669\_f0 | denovo6695\_f0 | denovo6721\_f0 | denovo6747\_f0 | denovo6773\_f0 | denovo6981\_f0 | denovo7007\_f0 | denovo7085\_f0 | denovo7137\_f0 | denovo7163\_f0 | denovo7267\_f0 | denovo7293\_f0 | denovo7319\_f0 | denovo7345\_f0 | denovo7371\_f0 | denovo7397\_f0 | denovo7501\_f0 | denovo7527\_f0 | denovo7553\_f0 | denovo7631\_f0 | denovo7683\_f0 | denovo7709\_f0 | denovo7839\_f0 | denovo7917\_f0 | denovo8047\_f0 | denovo8073\_f0 | denovo8229\_f0 | denovo8255\_f0 | denovo8281\_f0 | denovo8307\_f0 | denovo8385\_f0 | denovo8411\_f0 | denovo8437\_f0 | denovo8463\_f0 | denovo8567\_f0 | denovo8593\_f0 | denovo8645\_f0 | denovo8671\_f0 | denovo8697\_f0 | denovo8775\_f0 | denovo8853\_f0 | denovo8905\_f0 | denovo8931\_f0 | denovo8957\_f0 | denovo8983\_f0 | denovo9009\_f0 | denovo9139\_f0 | denovo9165\_f0 | denovo9191\_f0 | denovo9295\_f0 | denovo9321\_f0 | denovo9347\_f0 | denovo9373\_f0 | denovo9399\_f0 | denovo9451\_f0 | denovo9503\_f0 | denovo9711\_f0 | denovo9737\_f0 | denovo9789\_f0 | denovo9815\_f0 | denovo9841\_f0 | denovo9893\_f0 | denovo9971\_f0 | denovo10205\_f0 | denovo10231\_f0 | denovo10283\_f0 | denovo10309\_f0 | denovo10361\_f0 | denovo10413\_f0 | denovo10439\_f0 | denovo10465\_f0 | denovo10517\_f0 | denovo10543\_f0 | denovo10621\_f0 | denovo10673\_f0 | denovo10725\_f0 | denovo10751\_f0 | denovo10777\_f0 | denovo10829\_f0 | denovo10855\_f0 | denovo10933\_f0 | denovo11011\_f0 | denovo11037\_f0 | denovo11089\_f0 | denovo11167\_f0 | denovo11193\_f0 | denovo11297\_f0 | denovo11323\_f0 | denovo11349\_f0 | denovo11401\_f0 | denovo11427\_f0 | denovo11453\_f0 | denovo11479\_f0 | denovo11557\_f0 | denovo11713\_f0 | denovo11739\_f0 | denovo11921\_f0 | denovo12077\_f0 |
| Bmoricds | Bombyx mori\* | denovo0\_f0 | denovo78\_f0 | denovo156\_f0 | denovo208\_f0 | denovo260\_f0 | denovo364\_f0 | denovo416\_f0 | denovo442\_f0 | denovo520\_f0 | denovo572\_f0 | denovo598\_f0 | denovo780\_f0 | denovo1040\_f0 | denovo1066\_f0 | denovo1144\_f0 | denovo1222\_f0 | denovo1352\_f0 | denovo1378\_f0 | denovo1404\_f0 | denovo1482\_f0 | denovo1508\_f0 | denovo1638\_f0 | denovo1664\_f0 | denovo1742\_f0 | denovo1794\_f0 | denovo1924\_f0 | denovo1950\_f0 | denovo1976\_f0 | denovo2002\_f0 | denovo2106\_f0 | denovo2132\_f0 | denovo2184\_f0 | denovo2236\_f0 | denovo2262\_f0 | denovo2288\_f0 | denovo2444\_f0 | denovo2470\_f0 | denovo2496\_f0 | denovo2522\_f0 | denovo2574\_f0 | denovo2600\_f0 | denovo2626\_f0 | denovo2652\_f0 | denovo2756\_f0 | denovo2834\_f0 | denovo2912\_f0 | denovo2990\_f0 | denovo3146\_f0 | denovo3250\_f0 | denovo3302\_f0 | denovo3328\_f0 | denovo3432\_f0 | denovo3484\_f0 | denovo3588\_f0 | denovo3614\_f0 | denovo3666\_f0 | denovo3718\_f0 | denovo3744\_f0 | denovo3770\_f0 | denovo3796\_f0 | denovo3822\_f0 | denovo3848\_f0 | denovo3952\_f0 | denovo3978\_f0 | denovo4056\_f0 | denovo4186\_f0 | denovo4316\_f0 | denovo4342\_f0 | denovo4368\_f0 | denovo4394\_f0 | denovo4550\_f0 | denovo4628\_f0 | denovo4732\_f0 | denovo4758\_f0 | denovo4784\_f0 | denovo4836\_f0 | denovo4888\_f0 | denovo4914\_f0 | denovo4940\_f0 | denovo4966\_f0 | denovo4992\_f0 | denovo5200\_f0 | denovo5226\_f0 | denovo5252\_f0 | denovo5434\_f0 | denovo5590\_f0 | denovo5642\_f0 | denovo5954\_f0 | denovo6006\_f0 | denovo6058\_f0 | denovo6084\_f0 | denovo6110\_f0 | denovo6188\_f0 | denovo6214\_f0 | denovo6240\_f0 | denovo6266\_f0 | denovo6292\_f0 | denovo6370\_f0 | denovo6500\_f0 | denovo6552\_f0 | denovo6578\_f0 | denovo6604\_f0 | denovo6656\_f0 | denovo6682\_f0 | denovo6708\_f0 | denovo6734\_f0 | denovo6760\_f0 | denovo6968\_f0 | denovo6994\_f0 | denovo7072\_f0 | denovo7124\_f0 | denovo7150\_f0 | denovo7254\_f0 | denovo7280\_f0 | denovo7306\_f0 | denovo7332\_f0 | denovo7358\_f0 | denovo7384\_f0 | denovo7488\_f0 | denovo7514\_f0 | denovo7540\_f0 | denovo7618\_f0 | denovo7670\_f0 | denovo7696\_f0 | denovo7826\_f0 | denovo7904\_f0 | denovo8034\_f0 | denovo8060\_f0 | denovo8216\_f0 | denovo8242\_f0 | denovo8268\_f0 | denovo8294\_f0 | denovo8372\_f0 | denovo8398\_f0 | denovo8424\_f0 | denovo8450\_f0 | denovo8554\_f0 | denovo8580\_f0 | denovo8632\_f0 | denovo8658\_f0 | denovo8684\_f0 | denovo8762\_f0 | denovo8840\_f0 | denovo8892\_f0 | denovo8918\_f0 | denovo8944\_f0 | denovo8970\_f0 | denovo8996\_f0 | denovo9126\_f0 | denovo9152\_f0 | denovo9178\_f0 | denovo9282\_f0 | denovo9308\_f0 | denovo9334\_f0 | denovo9360\_f0 | denovo9386\_f0 | denovo9438\_f0 | denovo9490\_f0 | denovo9698\_f0 | denovo9724\_f0 | denovo9776\_f0 | denovo9802\_f0 | denovo9828\_f0 | denovo9880\_f0 | denovo9958\_f0 | denovo10192\_f0 | denovo10218\_f0 | denovo10270\_f0 | denovo10296\_f0 | denovo10348\_f0 | denovo10400\_f0 | denovo10426\_f0 | denovo10452\_f0 | denovo10504\_f0 | denovo10530\_f0 | denovo10608\_f0 | denovo10660\_f0 | denovo10712\_f0 | denovo10738\_f0 | denovo10764\_f0 | denovo10816\_f0 | denovo10842\_f0 | denovo10920\_f0 | denovo10998\_f0 | denovo11024\_f0 | denovo11076\_f0 | denovo11154\_f0 | denovo11180\_f0 | denovo11284\_f0 | denovo11310\_f0 | denovo11336\_f0 | denovo11388\_f0 | denovo11414\_f0 | denovo11440\_f0 | denovo11466\_f0 | denovo11544\_f0 | denovo11700\_f0 | denovo11726\_f0 | denovo11908\_f0 | denovo12064\_f0 |
| Pcit2 | Phyllocnistis citrella\* | denovo19\_f0 | denovo97\_f0 | denovo175\_f0 | denovo227\_f0 | denovo279\_f0 | denovo383\_f0 | denovo435\_f0 | denovo461\_f0 | denovo539\_f0 | denovo591\_f0 | denovo617\_f0 | denovo799\_f0 | denovo1059\_f0 | denovo1085\_f0 | denovo1163\_f0 | denovo1241\_f0 | denovo1371\_f0 | denovo1397\_f0 | denovo1423\_f0 | denovo1501\_f0 | denovo1527\_f0 | denovo1657\_f0 | denovo1683\_f0 | denovo1761\_f0 | denovo1813\_f0 | denovo1943\_f0 | denovo1969\_f0 | denovo1995\_f0 | denovo2021\_f0 | denovo2125\_f0 | denovo2151\_f0 | denovo2203\_f0 | denovo2255\_f0 | denovo2281\_f0 | denovo2307\_f0 | denovo2463\_f0 | denovo2489\_f0 | denovo2515\_f0 | denovo2541\_f0 | denovo2593\_f0 | denovo2619\_f0 | denovo2645\_f0 | denovo2671\_f0 | denovo2775\_f0 | denovo2853\_f0 | denovo2931\_f0 | denovo3009\_f0 | denovo3165\_f0 | denovo3269\_f0 | denovo3321\_f0 | denovo3347\_f0 | denovo3451\_f0 | denovo3503\_f0 | denovo3607\_f0 | denovo3633\_f0 | denovo3685\_f0 | denovo3737\_f0 | denovo3763\_f0 | denovo3789\_f0 | denovo3815\_f0 | denovo3841\_f0 | denovo3867\_f0 | denovo3971\_f0 | denovo3997\_f0 | denovo4075\_f0 | denovo4205\_f0 | denovo4335\_f0 | denovo4361\_f0 | denovo4387\_f0 | denovo4413\_f0 | denovo4569\_f0 | denovo4647\_f0 | denovo4751\_f0 | denovo4777\_f0 | denovo4803\_f0 | denovo4855\_f0 | denovo4907\_f0 | denovo4933\_f0 | denovo4959\_f0 | denovo4985\_f0 | denovo5011\_f0 | denovo5219\_f0 | denovo5245\_f0 | denovo5271\_f0 | denovo5453\_f0 | denovo5609\_f0 | denovo5661\_f0 | denovo5973\_f0 | denovo6025\_f0 | denovo6077\_f0 | denovo6103\_f0 | denovo6129\_f0 | denovo6207\_f0 | denovo6233\_f0 | denovo6259\_f0 | denovo6285\_f0 | denovo6311\_f0 | denovo6389\_f0 | denovo6519\_f0 | denovo6571\_f0 | denovo6597\_f0 | denovo6623\_f0 | denovo6675\_f0 | denovo6701\_f0 | denovo6727\_f0 | denovo6753\_f0 | denovo6779\_f0 | denovo6987\_f0 | denovo7013\_f0 | denovo7091\_f0 | denovo7143\_f0 | denovo7169\_f0 | denovo7273\_f0 | denovo7299\_f0 | denovo7325\_f0 | denovo7351\_f0 | denovo7377\_f0 | denovo7403\_f0 | denovo7507\_f0 | denovo7533\_f0 | denovo7559\_f0 | denovo7637\_f0 | denovo7689\_f0 | denovo7715\_f0 | denovo7845\_f0 | denovo7923\_f0 | denovo8053\_f0 | denovo8079\_f0 | denovo8235\_f0 | denovo8261\_f0 | denovo8287\_f0 | denovo8313\_f0 | denovo8391\_f0 | denovo8417\_f0 | denovo8443\_f0 | denovo8469\_f0 | denovo8573\_f0 | denovo8599\_f0 | denovo8651\_f0 | denovo8677\_f0 | denovo8703\_f0 | denovo8781\_f0 | denovo8859\_f0 | denovo8911\_f0 | denovo8937\_f0 | denovo8963\_f0 | denovo8989\_f0 | denovo9015\_f0 | denovo9145\_f0 | denovo9171\_f0 | denovo9197\_f0 | denovo9301\_f0 | denovo9327\_f0 | denovo9353\_f0 | denovo9379\_f0 | denovo9405\_f0 | denovo9457\_f0 | denovo9509\_f0 | denovo9717\_f0 | denovo9743\_f0 | denovo9795\_f0 | denovo9821\_f0 | denovo9847\_f0 | denovo9899\_f0 | denovo9977\_f0 | denovo10211\_f0 | denovo10237\_f0 | denovo10289\_f0 | denovo10315\_f0 | denovo10367\_f0 | denovo10419\_f0 | denovo10445\_f0 | denovo10471\_f0 | denovo10523\_f0 | denovo10549\_f0 | denovo10627\_f0 | denovo10679\_f0 | denovo10731\_f0 | denovo10757\_f0 | denovo10783\_f0 | denovo10835\_f0 | denovo10861\_f0 | denovo10939\_f0 | denovo11017\_f0 | denovo11043\_f0 | denovo11095\_f0 | denovo11173\_f0 | denovo11199\_f0 | denovo11303\_f0 | denovo11329\_f0 | denovo11355\_f0 | denovo11407\_f0 | denovo11433\_f0 | denovo11459\_f0 | denovo11485\_f0 | denovo11563\_f0 | denovo11719\_f0 | denovo11745\_f0 | denovo11927\_f0 | denovo12083\_f0 |

#### Content Of Concatenation "Entropy\_0.30\_0.00\_Loci\_100\_To\_299"

  
Rules for "entropy\_0.30\_0.00\_loci\_100\_to\_299":  
OTUs must have the loci:   
OTUs must have at least one of the following loci:

```
'EOG69CQC1_1', 'EOG6SN1SH_1', 'EOG6PRSVJ_1', 'EOG6PRSVH_1', 'EOG60ZR1Z_1', 'EOG6FXRCN_1', 'EOG60P4BD_1', 'EOG6NCMGZ_1', 'EOG6K3M19_1', 'EOG63TZZV_1', 'EOG6G7C30_1', 'EOG68KRFS_1', 'EOG666VQ4_1', 'EOG698V44_1', 'EOG6F7NPM_1', 'EOG6894QQ_1', 'EOG6868GW_1', 'EOG69PB31_1', 'EOG6JDHBD_1', 'EOG68D102_1', 'EOG60ZR1T_1', 'EOG63JC89_1', 'EOG698V46_1', 'EOG6J6RV9_1', 'EOG68KRG8_1', 'EOG6N8R81_1', 'EOG63BMSV_1', 'EOG69KFV1_1', 'EOG6C2HF4_1', 'EOG6CC44S_1', 'EOG6FXRCK_1', 'EOG68KRG4_1', 'EOG61C70T_1', 'EOG6BK575_1', 'EOG6R506X_1', 'EOG6JT09G_1', 'EOG6F7NPJ_1', 'EOG61NTQM_1', 'EOG6SJ5J3_1', 'EOG6NZTZ3_1', 'EOG6J3WM7_1', 'EOG680J16_1', 'EOG68SGXM_1', 'EOG6RNBCT_1', 'EOG6RFKXS_1', 'EOG6B8JHB_1', 'EOG6QFWHZ_1', 'EOG6RFKXB_1', 'EOG647FXX_1', 'EOG6B8JHP_1', 'EOG6S1TBX_1', 'EOG6DFPRT_1', 'EOG65HS25_1', 'EOG6FFD5C_1', 'EOG6PNXM9_1', 'EOG6F4SFZ_1', 'EOG6Q8524_1', 'EOG669QZR_1', 'EOG6JT09B_1', 'EOG6M65N6_1', 'EOG6QRH81_1', 'EOG65HS1N_1', 'EOG6FR0VW_1', 'EOG6KKZ6X_1', 'EOG60VVTG_1', 'EOG6QC198_1', 'EOG6D26TG_1', 'EOG6FFD52_1', 'EOG634W9K_1', 'EOG6DNF7N_1', 'EOG61RPZF_1', 'EOG60GCVD_1', 'EOG605S4X_1', 'EOG65TCRN_1', 'EOG608ND4_1', 'EOG6640GX_1', 'EOG6SQX12_1', 'EOG69GKMS_1', 'EOG6B8JHC_1', 'EOG6Q2DJZ_1', 'EOG6D26TV_1', 'EOG6NS3G8_1', 'EOG64TPCQ_1', 'EOG6229Q2_1', 'EOG63TZZW_1', 'EOG6BG90S_1', 'EOG66DM62_1', 'EOG6QFWJH_1', 'EOG69CQBW_1', 'EOG6N04K4_1', 'EOG69KFTV_1', 'EOG62V8KS_1', 'EOG6C5CPH_1', 'EOG6FFD51_1', 'EOG6QNN0J_1', 'EOG6KSPP5_1', 'EOG6N5W22_1', 'EOG6PK2CG_1', 'EOG6NP779_1', 'EOG6933N9_1', 'EOG66Q6XP_1', 'EOG6JQ427_1', 'EOG6C5CNP_1', 'EOG6R5066_1', 'EOG6FTW4C_1', 'EOG641QFN_1', 'EOG65B1K2_1', 'EOG66WZD1_1', 'EOG65TCRK_1', 'EOG6FR0WR_1', 'EOG6P2Q64_1', 'EOG666VQC_1', 'EOG6PC9WF_1', 'EOG6Q58T0_1', 'EOG641QGH_1', 'EOG6933N7_1', 'EOG69S6BG_1', 'EOG6PK2CM_1', 'EOG6NS3FV_1', 'EOG65MN9B_1', 'EOG641QFW_1', 'EOG6N04J6_1', 'EOG6GMV1P_1', 'EOG6S4PKX_1', 'EOG6K6G7S_1', 'EOG6BZN62_1', 'EOG6SXNGX_1', 'EOG6QC18P_1', 'EOG6CG0CT_1', 'EOG605S4Q_1', 'EOG6R7VF9_1', 'EOG6CZBM5_1', 'EOG63R3RK_1', 'EOG64F6DK_1', 'EOG6FJ8D6_1', 'EOG6001NN_1', 'EOG695ZWC_1', 'EOG67PX9B_1', 'EOG6P8FNH_1', 'EOG6KSPQ0_1', 'EOG63R3RC_1', 'EOG6907D7_1', 'EOG6JWVHT_1', 'EOG68SGX8_1', 'EOG6KD6Q9_1', 'EOG68GW6Q_1', 'EOG6Q852G_1', 'EOG676K44_1', 'EOG64MXWZ_1', 'EOG6S1TC2_1', 'EOG6Q58SW_1', 'EOG6CRM3H_1', 'EOG6PRSVP_1', 'EOG6R5069_1', 'EOG676K40_1', 'EOG64MXWK_1', 'EOG6N30SN_1', 'EOG6DR9GZ_1', 'EOG6255XM_1', 'EOG62FSN7_1', 'EOG6H72G0_1', 'EOG6PZJB1_1', 'EOG65QHHP_1', 'EOG615GHV_1', 'EOG66WZCQ_1', 'EOG698V4H_1', 'EOG62BXCZ_1', 'EOG6CG0D0_1', 'EOG69KFVF_1', 'EOG615GHP_1', 'EOG66HGF5_1', 'EOG6DFPSF_1', 'EOG66MBPR_1', 'EOG6DFPSD_1', 'EOG67D9KC_1', 'EOG6HHP5S_1', 'EOG6DBTJX_1', 'EOG6DBTHZ_1', 'EOG6KH2ZD_1', 'EOG6STS87_1', 'EOG67D9KV_1', 'EOG61RPZ9_1', 'EOG637RJP_1', 'EOG66MBP7_1', 'EOG6JDHBG_1', 'EOG6DV5QM_1', 'EOG6FR0W5_1', 'EOG6K0QRT_1', 'EOG65DWSM_1', 'EOG6CC452_1', 'EOG6G4GTX_1', 'EOG64BB5H_1', 'EOG64J2NC_1', 'EOG6H72G3_1', 'EOG6SBF2B_1', 'EOG6FFD5V_1', 'EOG6G4GTT_1', 'EOG6GXFR5_1', 'EOG61C70W_1', 'EOG6KPTG0_1'
```

  

|  |  | EOG69CQC1\_1 | EOG6SN1SH\_1 | EOG6PRSVJ\_1 | EOG6PRSVH\_1 | EOG60ZR1Z\_1 | EOG6FXRCN\_1 | EOG60P4BD\_1 | EOG6NCMGZ\_1 | EOG6K3M19\_1 | EOG63TZZV\_1 | EOG6G7C30\_1 | EOG68KRFS\_1 | EOG666VQ4\_1 | EOG698V44\_1 | EOG6F7NPM\_1 | EOG6894QQ\_1 | EOG6868GW\_1 | EOG69PB31\_1 | EOG6JDHBD\_1 | EOG68D102\_1 | EOG60ZR1T\_1 | EOG63JC89\_1 | EOG698V46\_1 | EOG6J6RV9\_1 | EOG68KRG8\_1 | EOG6N8R81\_1 | EOG63BMSV\_1 | EOG69KFV1\_1 | EOG6C2HF4\_1 | EOG6CC44S\_1 | EOG6FXRCK\_1 | EOG68KRG4\_1 | EOG61C70T\_1 | EOG6BK575\_1 | EOG6R506X\_1 | EOG6JT09G\_1 | EOG6F7NPJ\_1 | EOG61NTQM\_1 | EOG6SJ5J3\_1 | EOG6NZTZ3\_1 | EOG6J3WM7\_1 | EOG680J16\_1 | EOG68SGXM\_1 | EOG6RNBCT\_1 | EOG6RFKXS\_1 | EOG6B8JHB\_1 | EOG6QFWHZ\_1 | EOG6RFKXB\_1 | EOG647FXX\_1 | EOG6B8JHP\_1 | EOG6S1TBX\_1 | EOG6DFPRT\_1 | EOG65HS25\_1 | EOG6FFD5C\_1 | EOG6PNXM9\_1 | EOG6F4SFZ\_1 | EOG6Q8524\_1 | EOG669QZR\_1 | EOG6JT09B\_1 | EOG6M65N6\_1 | EOG6QRH81\_1 | EOG65HS1N\_1 | EOG6FR0VW\_1 | EOG6KKZ6X\_1 | EOG60VVTG\_1 | EOG6QC198\_1 | EOG6D26TG\_1 | EOG6FFD52\_1 | EOG634W9K\_1 | EOG6DNF7N\_1 | EOG61RPZF\_1 | EOG60GCVD\_1 | EOG605S4X\_1 | EOG65TCRN\_1 | EOG608ND4\_1 | EOG6640GX\_1 | EOG6SQX12\_1 | EOG69GKMS\_1 | EOG6B8JHC\_1 | EOG6Q2DJZ\_1 | EOG6D26TV\_1 | EOG6NS3G8\_1 | EOG64TPCQ\_1 | EOG6229Q2\_1 | EOG63TZZW\_1 | EOG6BG90S\_1 | EOG66DM62\_1 | EOG6QFWJH\_1 | EOG69CQBW\_1 | EOG6N04K4\_1 | EOG69KFTV\_1 | EOG62V8KS\_1 | EOG6C5CPH\_1 | EOG6FFD51\_1 | EOG6QNN0J\_1 | EOG6KSPP5\_1 | EOG6N5W22\_1 | EOG6PK2CG\_1 | EOG6NP779\_1 | EOG6933N9\_1 | EOG66Q6XP\_1 | EOG6JQ427\_1 | EOG6C5CNP\_1 | EOG6R5066\_1 | EOG6FTW4C\_1 | EOG641QFN\_1 | EOG65B1K2\_1 | EOG66WZD1\_1 | EOG65TCRK\_1 | EOG6FR0WR\_1 | EOG6P2Q64\_1 | EOG666VQC\_1 | EOG6PC9WF\_1 | EOG6Q58T0\_1 | EOG641QGH\_1 | EOG6933N7\_1 | EOG69S6BG\_1 | EOG6PK2CM\_1 | EOG6NS3FV\_1 | EOG65MN9B\_1 | EOG641QFW\_1 | EOG6N04J6\_1 | EOG6GMV1P\_1 | EOG6S4PKX\_1 | EOG6K6G7S\_1 | EOG6BZN62\_1 | EOG6SXNGX\_1 | EOG6QC18P\_1 | EOG6CG0CT\_1 | EOG605S4Q\_1 | EOG6R7VF9\_1 | EOG6CZBM5\_1 | EOG63R3RK\_1 | EOG64F6DK\_1 | EOG6FJ8D6\_1 | EOG6001NN\_1 | EOG695ZWC\_1 | EOG67PX9B\_1 | EOG6P8FNH\_1 | EOG6KSPQ0\_1 | EOG63R3RC\_1 | EOG6907D7\_1 | EOG6JWVHT\_1 | EOG68SGX8\_1 | EOG6KD6Q9\_1 | EOG68GW6Q\_1 | EOG6Q852G\_1 | EOG676K44\_1 | EOG64MXWZ\_1 | EOG6S1TC2\_1 | EOG6Q58SW\_1 | EOG6CRM3H\_1 | EOG6PRSVP\_1 | EOG6R5069\_1 | EOG676K40\_1 | EOG64MXWK\_1 | EOG6N30SN\_1 | EOG6DR9GZ\_1 | EOG6255XM\_1 | EOG62FSN7\_1 | EOG6H72G0\_1 | EOG6PZJB1\_1 | EOG65QHHP\_1 | EOG615GHV\_1 | EOG66WZCQ\_1 | EOG698V4H\_1 | EOG62BXCZ\_1 | EOG6CG0D0\_1 | EOG69KFVF\_1 | EOG615GHP\_1 | EOG66HGF5\_1 | EOG6DFPSF\_1 | EOG66MBPR\_1 | EOG6DFPSD\_1 | EOG67D9KC\_1 | EOG6HHP5S\_1 | EOG6DBTJX\_1 | EOG6DBTHZ\_1 | EOG6KH2ZD\_1 | EOG6STS87\_1 | EOG67D9KV\_1 | EOG61RPZ9\_1 | EOG637RJP\_1 | EOG66MBP7\_1 | EOG6JDHBG\_1 | EOG6DV5QM\_1 | EOG6FR0W5\_1 | EOG6K0QRT\_1 | EOG65DWSM\_1 | EOG6CC452\_1 | EOG6G4GTX\_1 | EOG64BB5H\_1 | EOG64J2NC\_1 | EOG6H72G3\_1 | EOG6SBF2B\_1 | EOG6FFD5V\_1 | EOG6G4GTT\_1 | EOG6GXFR5\_1 | EOG61C70W\_1 | EOG6KPTG0\_1 |
| --- | --- | --- | --- | --- | --- | --- | --- | --- | --- | --- | --- | --- | --- | --- | --- | --- | --- | --- | --- | --- | --- | --- | --- | --- | --- | --- | --- | --- | --- | --- | --- | --- | --- | --- | --- | --- | --- | --- | --- | --- | --- | --- | --- | --- | --- | --- | --- | --- | --- | --- | --- | --- | --- | --- | --- | --- | --- | --- | --- | --- | --- | --- | --- | --- | --- | --- | --- | --- | --- | --- | --- | --- | --- | --- | --- | --- | --- | --- | --- | --- | --- | --- | --- | --- | --- | --- | --- | --- | --- | --- | --- | --- | --- | --- | --- | --- | --- | --- | --- | --- | --- | --- | --- | --- | --- | --- | --- | --- | --- | --- | --- | --- | --- | --- | --- | --- | --- | --- | --- | --- | --- | --- | --- | --- | --- | --- | --- | --- | --- | --- | --- | --- | --- | --- | --- | --- | --- | --- | --- | --- | --- | --- | --- | --- | --- | --- | --- | --- | --- | --- | --- | --- | --- | --- | --- | --- | --- | --- | --- | --- | --- | --- | --- | --- | --- | --- | --- | --- | --- | --- | --- | --- | --- | --- | --- | --- | --- | --- | --- | --- | --- | --- | --- | --- | --- | --- | --- | --- | --- | --- | --- | --- | --- | --- | --- | --- | --- | --- | --- | --- | --- |
| Dplexcds | Danaus plexippus\* | denovo2\_f0 | denovo80\_f0 | denovo158\_f0 | denovo184\_f0 | denovo262\_f0 | denovo314\_f0 | denovo366\_f0 | denovo444\_f0 | denovo522\_f0 | denovo678\_f0 | denovo834\_f0 | denovo1042\_f0 | denovo1146\_f0 | denovo1224\_f0 | denovo1250\_f0 | denovo1354\_f0 | denovo1380\_f0 | denovo1406\_f0 | denovo1484\_f0 | denovo1640\_f0 | denovo1666\_f0 | denovo1718\_f0 | denovo1796\_f0 | denovo1848\_f0 | denovo1926\_f0 | denovo1952\_f0 | denovo1978\_f0 | denovo2004\_f0 | denovo2082\_f0 | denovo2108\_f0 | denovo2134\_f0 | denovo2238\_f0 | denovo2264\_f0 | denovo2290\_f0 | denovo2316\_f0 | denovo2368\_f0 | denovo2472\_f0 | denovo2524\_f0 | denovo2550\_f0 | denovo2576\_f0 | denovo2602\_f0 | denovo2628\_f0 | denovo2654\_f0 | denovo2758\_f0 | denovo2836\_f0 | denovo2888\_f0 | denovo3148\_f0 | denovo3252\_f0 | denovo3304\_f0 | denovo3330\_f0 | denovo3434\_f0 | denovo3486\_f0 | denovo3590\_f0 | denovo3616\_f0 | denovo3642\_f0 | denovo3720\_f0 | denovo3746\_f0 | denovo3798\_f0 | denovo3824\_f0 | denovo3980\_f0 | denovo4058\_f0 | denovo4136\_f0 | denovo4240\_f0 | denovo4318\_f0 | denovo4344\_f0 | denovo4370\_f0 | denovo4396\_f0 | denovo4422\_f0 | denovo4500\_f0 | denovo4526\_f0 | denovo4630\_f0 | denovo4760\_f0 | denovo4838\_f0 | denovo4890\_f0 | denovo4916\_f0 | denovo4942\_f0 | denovo4968\_f0 | denovo4994\_f0 | denovo5098\_f0 | denovo5150\_f0 | denovo5202\_f0 | denovo5410\_f0 | denovo5436\_f0 | denovo5540\_f0 | denovo5566\_f0 | denovo5904\_f0 | denovo5956\_f0 | denovo6008\_f0 | denovo6034\_f0 | denovo6060\_f0 | denovo6086\_f0 | denovo6190\_f0 | denovo6242\_f0 | denovo6268\_f0 | denovo6294\_f0 | denovo6372\_f0 | denovo6502\_f0 | denovo6554\_f0 | denovo6580\_f0 | denovo6606\_f0 | denovo6658\_f0 | denovo6684\_f0 | denovo6710\_f0 | denovo6736\_f0 | denovo6762\_f0 | denovo6892\_f0 | denovo6970\_f0 | denovo6996\_f0 | denovo7022\_f0 | denovo7074\_f0 | denovo7126\_f0 | denovo7152\_f0 | denovo7282\_f0 | denovo7308\_f0 | denovo7334\_f0 | denovo7360\_f0 | denovo7386\_f0 | denovo7464\_f0 | denovo7490\_f0 | denovo7516\_f0 | denovo7542\_f0 | denovo7620\_f0 | denovo7672\_f0 | denovo7698\_f0 | denovo7776\_f0 | denovo7828\_f0 | denovo7906\_f0 | denovo8010\_f0 | denovo8036\_f0 | denovo8218\_f0 | denovo8244\_f0 | denovo8270\_f0 | denovo8348\_f0 | denovo8374\_f0 | denovo8400\_f0 | denovo8478\_f0 | denovo8530\_f0 | denovo8556\_f0 | denovo8608\_f0 | denovo8634\_f0 | denovo8686\_f0 | denovo8712\_f0 | denovo8738\_f0 | denovo8764\_f0 | denovo8894\_f0 | denovo8920\_f0 | denovo8946\_f0 | denovo8972\_f0 | denovo9102\_f0 | denovo9128\_f0 | denovo9232\_f0 | denovo9284\_f0 | denovo9310\_f0 | denovo9336\_f0 | denovo9440\_f0 | denovo9466\_f0 | denovo9492\_f0 | denovo9648\_f0 | denovo9700\_f0 | denovo9778\_f0 | denovo9804\_f0 | denovo9830\_f0 | denovo9882\_f0 | denovo9960\_f0 | denovo10012\_f0 | denovo10038\_f0 | denovo10194\_f0 | denovo10220\_f0 | denovo10272\_f0 | denovo10298\_f0 | denovo10428\_f0 | denovo10454\_f0 | denovo10506\_f0 | denovo10532\_f0 | denovo10558\_f0 | denovo10584\_f0 | denovo10662\_f0 | denovo10714\_f0 | denovo10740\_f0 | denovo10766\_f0 | denovo10818\_f0 | denovo11000\_f0 | denovo11026\_f0 | denovo11052\_f0 | denovo11182\_f0 | denovo11286\_f0 | denovo11338\_f0 | denovo11390\_f0 | denovo11416\_f0 | denovo11442\_f0 | denovo11468\_f0 | denovo11494\_f0 | denovo11520\_f0 | denovo11572\_f0 | denovo11598\_f0 | denovo11702\_f0 | denovo11728\_f0 | denovo11910\_f0 | denovo11936\_f0 | denovo12066\_f0 |
| FG120077 | Semomesia campanea | denovo10\_f0 | denovo88\_f0 | denovo166\_f0 | denovo192\_f0 | denovo270\_f0 | denovo322\_f0 | denovo374\_f0 | denovo452\_f0 | denovo530\_f0 | denovo686\_f0 | denovo842\_f0 | denovo1050\_f0 | denovo1154\_f0 | denovo1232\_f0 | denovo1258\_f0 | denovo1362\_f0 | denovo1388\_f0 | denovo1414\_f0 | denovo1492\_f0 | denovo1648\_f0 | denovo1674\_f0 | denovo1726\_f0 | denovo1804\_f0 | denovo1856\_f0 | denovo1934\_f0 | denovo1960\_f0 | denovo1986\_f0 | denovo2012\_f0 | denovo2090\_f0 | denovo2116\_f0 | denovo2142\_f0 | denovo2246\_f0 | denovo2272\_f0 | denovo2298\_f0 | denovo2324\_f0 | denovo2376\_f0 | denovo2480\_f0 | denovo2532\_f0 | denovo2558\_f0 | denovo2584\_f0 | denovo2610\_f0 | denovo2636\_f0 | denovo2662\_f0 | denovo2766\_f0 | denovo2844\_f0 | denovo2896\_f0 | denovo3156\_f0 | denovo3260\_f0 | denovo3312\_f0 | denovo3338\_f0 | denovo3442\_f0 | denovo3494\_f0 | denovo3598\_f0 | denovo3624\_f0 | denovo3650\_f0 | denovo3728\_f0 | denovo3754\_f0 | denovo3806\_f0 | denovo3832\_f0 | denovo3988\_f0 | denovo4066\_f0 | denovo4144\_f0 | denovo4248\_f0 | denovo4326\_f0 | denovo4352\_f0 | denovo4378\_f0 | denovo4404\_f0 | denovo4430\_f0 | denovo4508\_f0 | denovo4534\_f0 | denovo4638\_f0 | denovo4768\_f0 | denovo4846\_f0 | denovo4898\_f0 | denovo4924\_f0 | denovo4950\_f0 | denovo4976\_f0 | denovo5002\_f0 | denovo5106\_f0 | denovo5158\_f0 | denovo5210\_f0 | denovo5418\_f0 | denovo5444\_f0 | denovo5548\_f0 | denovo5574\_f0 | denovo5912\_f0 | denovo5964\_f0 | denovo6016\_f0 | denovo6042\_f0 | denovo6068\_f0 | denovo6094\_f0 | denovo6198\_f0 | denovo6250\_f0 | denovo6276\_f0 | denovo6302\_f0 | denovo6380\_f0 | denovo6510\_f0 | denovo6562\_f0 | denovo6588\_f0 | denovo6614\_f0 | denovo6666\_f0 | denovo6692\_f0 | denovo6718\_f0 | denovo6744\_f0 | denovo6770\_f0 | denovo6900\_f0 | denovo6978\_f0 | denovo7004\_f0 | denovo7030\_f0 | denovo7082\_f0 | denovo7134\_f0 | denovo7160\_f0 | denovo7290\_f0 | denovo7316\_f0 | denovo7342\_f0 | denovo7368\_f0 | denovo7394\_f0 | denovo7472\_f0 | denovo7498\_f0 | denovo7524\_f0 | denovo7550\_f0 | denovo7628\_f0 | denovo7680\_f0 | denovo7706\_f0 | denovo7784\_f0 | denovo7836\_f0 | denovo7914\_f0 | denovo8018\_f0 | denovo8044\_f0 | denovo8226\_f0 | denovo8252\_f0 | denovo8278\_f0 | denovo8356\_f0 | denovo8382\_f0 | denovo8408\_f0 | denovo8486\_f0 | denovo8538\_f0 | denovo8564\_f0 | denovo8616\_f0 | denovo8642\_f0 | denovo8694\_f0 | denovo8720\_f0 | denovo8746\_f0 | denovo8772\_f0 | denovo8902\_f0 | denovo8928\_f0 | denovo8954\_f0 | denovo8980\_f0 | denovo9110\_f0 | denovo9136\_f0 | denovo9240\_f0 | denovo9292\_f0 | denovo9318\_f0 | denovo9344\_f0 | denovo9448\_f0 | denovo9474\_f0 | denovo9500\_f0 | denovo9656\_f0 | denovo9708\_f0 | denovo9786\_f0 | denovo9812\_f0 | denovo9838\_f0 | denovo9890\_f0 | denovo9968\_f0 | denovo10020\_f0 | denovo10046\_f0 | denovo10202\_f0 | denovo10228\_f0 | denovo10280\_f0 | denovo10306\_f0 | denovo10436\_f0 | denovo10462\_f0 | denovo10514\_f0 | denovo10540\_f0 | denovo10566\_f0 | denovo10592\_f0 | denovo10670\_f0 | denovo10722\_f0 | denovo10748\_f0 | denovo10774\_f0 | denovo10826\_f0 | denovo11008\_f0 | denovo11034\_f0 | denovo11060\_f0 | denovo11190\_f0 | denovo11294\_f0 | denovo11346\_f0 | denovo11398\_f0 | denovo11424\_f0 | denovo11450\_f0 | denovo11476\_f0 | denovo11502\_f0 | denovo11528\_f0 | denovo11580\_f0 | denovo11606\_f0 | denovo11710\_f0 | denovo11736\_f0 | denovo11918\_f0 | denovo11944\_f0 | denovo12074\_f0 |
| SRR803483 | Grapholita dimorpha\* | denovo20\_f0 | denovo98\_f0 | denovo176\_f0 | denovo202\_f0 | denovo280\_f0 | denovo332\_f0 | denovo384\_f0 | denovo462\_f0 | denovo540\_f0 | denovo696\_f0 | denovo852\_f0 | denovo1060\_f0 | denovo1164\_f0 | denovo1242\_f0 | denovo1268\_f0 | denovo1372\_f0 | denovo1398\_f0 | denovo1424\_f0 | denovo1502\_f0 | denovo1658\_f0 | denovo1684\_f0 | denovo1736\_f0 | denovo1814\_f0 | denovo1866\_f0 | denovo1944\_f0 | denovo1970\_f0 | denovo1996\_f0 | denovo2022\_f0 | denovo2100\_f0 | denovo2126\_f0 | denovo2152\_f0 | denovo2256\_f0 | denovo2282\_f0 | denovo2308\_f0 | denovo2334\_f0 | denovo2386\_f0 | denovo2490\_f0 | denovo2542\_f0 | denovo2568\_f0 | denovo2594\_f0 | denovo2620\_f0 | denovo2646\_f0 | denovo2672\_f0 | denovo2776\_f0 | denovo2854\_f0 | denovo2906\_f0 | denovo3166\_f0 | denovo3270\_f0 | denovo3322\_f0 | denovo3348\_f0 | denovo3452\_f0 | denovo3504\_f0 | denovo3608\_f0 | denovo3634\_f0 | denovo3660\_f0 | denovo3738\_f0 | denovo3764\_f0 | denovo3816\_f0 | denovo3842\_f0 | denovo3998\_f0 | denovo4076\_f0 | denovo4154\_f0 | denovo4258\_f0 | denovo4336\_f0 | denovo4362\_f0 | denovo4388\_f0 | denovo4414\_f0 | denovo4440\_f0 | denovo4518\_f0 | denovo4544\_f0 | denovo4648\_f0 | denovo4778\_f0 | denovo4856\_f0 | denovo4908\_f0 | denovo4934\_f0 | denovo4960\_f0 | denovo4986\_f0 | denovo5012\_f0 | denovo5116\_f0 | denovo5168\_f0 | denovo5220\_f0 | denovo5428\_f0 | denovo5454\_f0 | denovo5558\_f0 | denovo5584\_f0 | denovo5922\_f0 | denovo5974\_f0 | denovo6026\_f0 | denovo6052\_f0 | denovo6078\_f0 | denovo6104\_f0 | denovo6208\_f0 | denovo6260\_f0 | denovo6286\_f0 | denovo6312\_f0 | denovo6390\_f0 | denovo6520\_f0 | denovo6572\_f0 | denovo6598\_f0 | denovo6624\_f0 | denovo6676\_f0 | denovo6702\_f0 | denovo6728\_f0 | denovo6754\_f0 | denovo6780\_f0 | denovo6910\_f0 | denovo6988\_f0 | denovo7014\_f0 | denovo7040\_f0 | denovo7092\_f0 | denovo7144\_f0 | denovo7170\_f0 | denovo7300\_f0 | denovo7326\_f0 | denovo7352\_f0 | denovo7378\_f0 | denovo7404\_f0 | denovo7482\_f0 | denovo7508\_f0 | denovo7534\_f0 | denovo7560\_f0 | denovo7638\_f0 | denovo7690\_f0 | denovo7716\_f0 | denovo7794\_f0 | denovo7846\_f0 | denovo7924\_f0 | denovo8028\_f0 | denovo8054\_f0 | denovo8236\_f0 | denovo8262\_f0 | denovo8288\_f0 | denovo8366\_f0 | denovo8392\_f0 | denovo8418\_f0 | denovo8496\_f0 | denovo8548\_f0 | denovo8574\_f0 | denovo8626\_f0 | denovo8652\_f0 | denovo8704\_f0 | denovo8730\_f0 | denovo8756\_f0 | denovo8782\_f0 | denovo8912\_f0 | denovo8938\_f0 | denovo8964\_f0 | denovo8990\_f0 | denovo9120\_f0 | denovo9146\_f0 | denovo9250\_f0 | denovo9302\_f0 | denovo9328\_f0 | denovo9354\_f0 | denovo9458\_f0 | denovo9484\_f0 | denovo9510\_f0 | denovo9666\_f0 | denovo9718\_f0 | denovo9796\_f0 | denovo9822\_f0 | denovo9848\_f0 | denovo9900\_f0 | denovo9978\_f0 | denovo10030\_f0 | denovo10056\_f0 | denovo10212\_f0 | denovo10238\_f0 | denovo10290\_f0 | denovo10316\_f0 | denovo10446\_f0 | denovo10472\_f0 | denovo10524\_f0 | denovo10550\_f0 | denovo10576\_f0 | denovo10602\_f0 | denovo10680\_f0 | denovo10732\_f0 | denovo10758\_f0 | denovo10784\_f0 | denovo10836\_f0 | denovo11018\_f0 | denovo11044\_f0 | denovo11070\_f0 | denovo11200\_f0 | denovo11304\_f0 | denovo11356\_f0 | denovo11408\_f0 | denovo11434\_f0 | denovo11460\_f0 | denovo11486\_f0 | denovo11512\_f0 | denovo11538\_f0 | denovo11590\_f0 | denovo11616\_f0 | denovo11720\_f0 | denovo11746\_f0 | denovo11928\_f0 | denovo11954\_f0 | denovo12084\_f0 |
| FG120035 | Dalcera abrasa\* | denovo5\_f0 | denovo83\_f0 | denovo161\_f0 | denovo187\_f0 | denovo265\_f0 | denovo317\_f0 | denovo369\_f0 | denovo447\_f0 | denovo525\_f0 | denovo681\_f0 | denovo837\_f0 | denovo1045\_f0 | denovo1149\_f0 | denovo1227\_f0 | denovo1253\_f0 | denovo1357\_f0 | denovo1383\_f0 | denovo1409\_f0 | denovo1487\_f0 | denovo1643\_f0 | denovo1669\_f0 | denovo1721\_f0 | denovo1799\_f0 | denovo1851\_f0 | denovo1929\_f0 | denovo1955\_f0 | denovo1981\_f0 | denovo2007\_f0 | denovo2085\_f0 | denovo2111\_f0 | denovo2137\_f0 | denovo2241\_f0 | denovo2267\_f0 | denovo2293\_f0 | denovo2319\_f0 | denovo2371\_f0 | denovo2475\_f0 | denovo2527\_f0 | denovo2553\_f0 | denovo2579\_f0 | denovo2605\_f0 | denovo2631\_f0 | denovo2657\_f0 | denovo2761\_f0 | denovo2839\_f0 | denovo2891\_f0 | denovo3151\_f0 | denovo3255\_f0 | denovo3307\_f0 | denovo3333\_f0 | denovo3437\_f0 | denovo3489\_f0 | denovo3593\_f0 | denovo3619\_f0 | denovo3645\_f0 | denovo3723\_f0 | denovo3749\_f0 | denovo3801\_f0 | denovo3827\_f0 | denovo3983\_f0 | denovo4061\_f0 | denovo4139\_f0 | denovo4243\_f0 | denovo4321\_f0 | denovo4347\_f0 | denovo4373\_f0 | denovo4399\_f0 | denovo4425\_f0 | denovo4503\_f0 | denovo4529\_f0 | denovo4633\_f0 | denovo4763\_f0 | denovo4841\_f0 | denovo4893\_f0 | denovo4919\_f0 | denovo4945\_f0 | denovo4971\_f0 | denovo4997\_f0 | denovo5101\_f0 | denovo5153\_f0 | denovo5205\_f0 | denovo5413\_f0 | denovo5439\_f0 | denovo5543\_f0 | denovo5569\_f0 | denovo5907\_f0 | denovo5959\_f0 | denovo6011\_f0 | denovo6037\_f0 | denovo6063\_f0 | denovo6089\_f0 | denovo6193\_f0 | denovo6245\_f0 | denovo6271\_f0 | denovo6297\_f0 | denovo6375\_f0 | denovo6505\_f0 | denovo6557\_f0 | denovo6583\_f0 | denovo6609\_f0 | denovo6661\_f0 | denovo6687\_f0 | denovo6713\_f0 | denovo6739\_f0 | denovo6765\_f0 | denovo6895\_f0 | denovo6973\_f0 | denovo6999\_f0 | denovo7025\_f0 | denovo7077\_f0 | denovo7129\_f0 | denovo7155\_f0 | denovo7285\_f0 | denovo7311\_f0 | denovo7337\_f0 | denovo7363\_f0 | denovo7389\_f0 | denovo7467\_f0 | denovo7493\_f0 | denovo7519\_f0 | denovo7545\_f0 | denovo7623\_f0 | denovo7675\_f0 | denovo7701\_f0 | denovo7779\_f0 | denovo7831\_f0 | denovo7909\_f0 | denovo8013\_f0 | denovo8039\_f0 | denovo8221\_f0 | denovo8247\_f0 | denovo8273\_f0 | denovo8351\_f0 | denovo8377\_f0 | denovo8403\_f0 | denovo8481\_f0 | denovo8533\_f0 | denovo8559\_f0 | denovo8611\_f0 | denovo8637\_f0 | denovo8689\_f0 | denovo8715\_f0 | denovo8741\_f0 | denovo8767\_f0 | denovo8897\_f0 | denovo8923\_f0 | denovo8949\_f0 | denovo8975\_f0 | denovo9105\_f0 | denovo9131\_f0 | denovo9235\_f0 | denovo9287\_f0 | denovo9313\_f0 | denovo9339\_f0 | denovo9443\_f0 | denovo9469\_f0 | denovo9495\_f0 | denovo9651\_f0 | denovo9703\_f0 | denovo9781\_f0 | denovo9807\_f0 | denovo9833\_f0 | denovo9885\_f0 | denovo9963\_f0 | denovo10015\_f0 | denovo10041\_f0 | denovo10197\_f0 | denovo10223\_f0 | denovo10275\_f0 | denovo10301\_f0 | denovo10431\_f0 | denovo10457\_f0 | denovo10509\_f0 | denovo10535\_f0 | denovo10561\_f0 | denovo10587\_f0 | denovo10665\_f0 | denovo10717\_f0 | denovo10743\_f0 | denovo10769\_f0 | denovo10821\_f0 | denovo11003\_f0 | denovo11029\_f0 | denovo11055\_f0 | denovo11185\_f0 | denovo11289\_f0 | denovo11341\_f0 | denovo11393\_f0 | denovo11419\_f0 | denovo11445\_f0 | denovo11471\_f0 | denovo11497\_f0 | denovo11523\_f0 | denovo11575\_f0 | denovo11601\_f0 | denovo11705\_f0 | denovo11731\_f0 | denovo11913\_f0 | denovo11939\_f0 | denovo12069\_f0 |
| FG120046B | Lacosoma ludolpha\* | denovo6\_f0 | denovo84\_f0 | denovo162\_f0 | denovo188\_f0 | denovo266\_f0 | denovo318\_f0 | denovo370\_f0 | denovo448\_f0 | denovo526\_f0 | denovo682\_f0 | denovo838\_f0 | denovo1046\_f0 | denovo1150\_f0 | denovo1228\_f0 | denovo1254\_f0 | denovo1358\_f0 | denovo1384\_f0 | denovo1410\_f0 | denovo1488\_f0 | denovo1644\_f0 | denovo1670\_f0 | denovo1722\_f0 | denovo1800\_f0 | denovo1852\_f0 | denovo1930\_f0 | denovo1956\_f0 | denovo1982\_f0 | denovo2008\_f0 | denovo2086\_f0 | denovo2112\_f0 | denovo2138\_f0 | denovo2242\_f0 | denovo2268\_f0 | denovo2294\_f0 | denovo2320\_f0 | denovo2372\_f0 | denovo2476\_f0 | denovo2528\_f0 | denovo2554\_f0 | denovo2580\_f0 | denovo2606\_f0 | denovo2632\_f0 | denovo2658\_f0 | denovo2762\_f0 | denovo2840\_f0 | denovo2892\_f0 | denovo3152\_f0 | denovo3256\_f0 | denovo3308\_f0 | denovo3334\_f0 | denovo3438\_f0 | denovo3490\_f0 | denovo3594\_f0 | denovo3620\_f0 | denovo3646\_f0 | denovo3724\_f0 | denovo3750\_f0 | denovo3802\_f0 | denovo3828\_f0 | denovo3984\_f0 | denovo4062\_f0 | denovo4140\_f0 | denovo4244\_f0 | denovo4322\_f0 | denovo4348\_f0 | denovo4374\_f0 | denovo4400\_f0 | denovo4426\_f0 | denovo4504\_f0 | denovo4530\_f0 | denovo4634\_f0 | denovo4764\_f0 | denovo4842\_f0 | denovo4894\_f0 | denovo4920\_f0 | denovo4946\_f0 | denovo4972\_f0 | denovo4998\_f0 | denovo5102\_f0 | denovo5154\_f0 | denovo5206\_f0 | denovo5414\_f0 | denovo5440\_f0 | denovo5544\_f0 | denovo5570\_f0 | denovo5908\_f0 | denovo5960\_f0 | denovo6012\_f0 | denovo6038\_f0 | denovo6064\_f0 | denovo6090\_f0 | denovo6194\_f0 | denovo6246\_f0 | denovo6272\_f0 | denovo6298\_f0 | denovo6376\_f0 | denovo6506\_f0 | denovo6558\_f0 | denovo6584\_f0 | denovo6610\_f0 | denovo6662\_f0 | denovo6688\_f0 | denovo6714\_f0 | denovo6740\_f0 | denovo6766\_f0 | denovo6896\_f0 | denovo6974\_f0 | denovo7000\_f0 | denovo7026\_f0 | denovo7078\_f0 | denovo7130\_f0 | denovo7156\_f0 | denovo7286\_f0 | denovo7312\_f0 | denovo7338\_f0 | denovo7364\_f0 | denovo7390\_f0 | denovo7468\_f0 | denovo7494\_f0 | denovo7520\_f0 | denovo7546\_f0 | denovo7624\_f0 | denovo7676\_f0 | denovo7702\_f0 | denovo7780\_f0 | denovo7832\_f0 | denovo7910\_f0 | denovo8014\_f0 | denovo8040\_f0 | denovo8222\_f0 | denovo8248\_f0 | denovo8274\_f0 | denovo8352\_f0 | denovo8378\_f0 | denovo8404\_f0 | denovo8482\_f0 | denovo8534\_f0 | denovo8560\_f0 | denovo8612\_f0 | denovo8638\_f0 | denovo8690\_f0 | denovo8716\_f0 | denovo8742\_f0 | denovo8768\_f0 | denovo8898\_f0 | denovo8924\_f0 | denovo8950\_f0 | denovo8976\_f0 | denovo9106\_f0 | denovo9132\_f0 | denovo9236\_f0 | denovo9288\_f0 | denovo9314\_f0 | denovo9340\_f0 | denovo9444\_f0 | denovo9470\_f0 | denovo9496\_f0 | denovo9652\_f0 | denovo9704\_f0 | denovo9782\_f0 | denovo9808\_f0 | denovo9834\_f0 | denovo9886\_f0 | denovo9964\_f0 | denovo10016\_f0 | denovo10042\_f0 | denovo10198\_f0 | denovo10224\_f0 | denovo10276\_f0 | denovo10302\_f0 | denovo10432\_f0 | denovo10458\_f0 | denovo10510\_f0 | denovo10536\_f0 | denovo10562\_f0 | denovo10588\_f0 | denovo10666\_f0 | denovo10718\_f0 | denovo10744\_f0 | denovo10770\_f0 | denovo10822\_f0 | denovo11004\_f0 | denovo11030\_f0 | denovo11056\_f0 | denovo11186\_f0 | denovo11290\_f0 | denovo11342\_f0 | denovo11394\_f0 | denovo11420\_f0 | denovo11446\_f0 | denovo11472\_f0 | denovo11498\_f0 | denovo11524\_f0 | denovo11576\_f0 | denovo11602\_f0 | denovo11706\_f0 | denovo11732\_f0 | denovo11914\_f0 | denovo11940\_f0 | denovo12070\_f0 |
| GNV129007 | Urodus parvula\* | denovo15\_f0 | denovo93\_f0 | denovo171\_f0 | denovo197\_f0 | denovo275\_f0 | denovo327\_f0 | denovo379\_f0 | denovo457\_f0 | denovo535\_f0 | denovo691\_f0 | denovo847\_f0 | denovo1055\_f0 | denovo1159\_f0 | denovo1237\_f0 | denovo1263\_f0 | denovo1367\_f0 | denovo1393\_f0 | denovo1419\_f0 | denovo1497\_f0 | denovo1653\_f0 | denovo1679\_f0 | denovo1731\_f0 | denovo1809\_f0 | denovo1861\_f0 | denovo1939\_f0 | denovo1965\_f0 | denovo1991\_f0 | denovo2017\_f0 | denovo2095\_f0 | denovo2121\_f0 | denovo2147\_f0 | denovo2251\_f0 | denovo2277\_f0 | denovo2303\_f0 | denovo2329\_f0 | denovo2381\_f0 | denovo2485\_f0 | denovo2537\_f0 | denovo2563\_f0 | denovo2589\_f0 | denovo2615\_f0 | denovo2641\_f0 | denovo2667\_f0 | denovo2771\_f0 | denovo2849\_f0 | denovo2901\_f0 | denovo3161\_f0 | denovo3265\_f0 | denovo3317\_f0 | denovo3343\_f0 | denovo3447\_f0 | denovo3499\_f0 | denovo3603\_f0 | denovo3629\_f0 | denovo3655\_f0 | denovo3733\_f0 | denovo3759\_f0 | denovo3811\_f0 | denovo3837\_f0 | denovo3993\_f0 | denovo4071\_f0 | denovo4149\_f0 | denovo4253\_f0 | denovo4331\_f0 | denovo4357\_f0 | denovo4383\_f0 | denovo4409\_f0 | denovo4435\_f0 | denovo4513\_f0 | denovo4539\_f0 | denovo4643\_f0 | denovo4773\_f0 | denovo4851\_f0 | denovo4903\_f0 | denovo4929\_f0 | denovo4955\_f0 | denovo4981\_f0 | denovo5007\_f0 | denovo5111\_f0 | denovo5163\_f0 | denovo5215\_f0 | denovo5423\_f0 | denovo5449\_f0 | denovo5553\_f0 | denovo5579\_f0 | denovo5917\_f0 | denovo5969\_f0 | denovo6021\_f0 | denovo6047\_f0 | denovo6073\_f0 | denovo6099\_f0 | denovo6203\_f0 | denovo6255\_f0 | denovo6281\_f0 | denovo6307\_f0 | denovo6385\_f0 | denovo6515\_f0 | denovo6567\_f0 | denovo6593\_f0 | denovo6619\_f0 | denovo6671\_f0 | denovo6697\_f0 | denovo6723\_f0 | denovo6749\_f0 | denovo6775\_f0 | denovo6905\_f0 | denovo6983\_f0 | denovo7009\_f0 | denovo7035\_f0 | denovo7087\_f0 | denovo7139\_f0 | denovo7165\_f0 | denovo7295\_f0 | denovo7321\_f0 | denovo7347\_f0 | denovo7373\_f0 | denovo7399\_f0 | denovo7477\_f0 | denovo7503\_f0 | denovo7529\_f0 | denovo7555\_f0 | denovo7633\_f0 | denovo7685\_f0 | denovo7711\_f0 | denovo7789\_f0 | denovo7841\_f0 | denovo7919\_f0 | denovo8023\_f0 | denovo8049\_f0 | denovo8231\_f0 | denovo8257\_f0 | denovo8283\_f0 | denovo8361\_f0 | denovo8387\_f0 | denovo8413\_f0 | denovo8491\_f0 | denovo8543\_f0 | denovo8569\_f0 | denovo8621\_f0 | denovo8647\_f0 | denovo8699\_f0 | denovo8725\_f0 | denovo8751\_f0 | denovo8777\_f0 | denovo8907\_f0 | denovo8933\_f0 | denovo8959\_f0 | denovo8985\_f0 | denovo9115\_f0 | denovo9141\_f0 | denovo9245\_f0 | denovo9297\_f0 | denovo9323\_f0 | denovo9349\_f0 | denovo9453\_f0 | denovo9479\_f0 | denovo9505\_f0 | denovo9661\_f0 | denovo9713\_f0 | denovo9791\_f0 | denovo9817\_f0 | denovo9843\_f0 | denovo9895\_f0 | denovo9973\_f0 | denovo10025\_f0 | denovo10051\_f0 | denovo10207\_f0 | denovo10233\_f0 | denovo10285\_f0 | denovo10311\_f0 | denovo10441\_f0 | denovo10467\_f0 | denovo10519\_f0 | denovo10545\_f0 | denovo10571\_f0 | denovo10597\_f0 | denovo10675\_f0 | denovo10727\_f0 | denovo10753\_f0 | denovo10779\_f0 | denovo10831\_f0 | denovo11013\_f0 | denovo11039\_f0 | denovo11065\_f0 | denovo11195\_f0 | denovo11299\_f0 | denovo11351\_f0 | denovo11403\_f0 | denovo11429\_f0 | denovo11455\_f0 | denovo11481\_f0 | denovo11507\_f0 | denovo11533\_f0 | denovo11585\_f0 | denovo11611\_f0 | denovo11715\_f0 | denovo11741\_f0 | denovo11923\_f0 | denovo11949\_f0 | denovo12079\_f0 |
| SW130126 | Lyssa zampa\* | denovo24\_f0 | denovo102\_f0 | denovo180\_f0 | denovo206\_f0 | denovo284\_f0 | denovo336\_f0 | denovo388\_f0 | denovo466\_f0 | denovo544\_f0 | denovo700\_f0 | denovo856\_f0 | denovo1064\_f0 | denovo1168\_f0 | denovo1246\_f0 | denovo1272\_f0 | denovo1376\_f0 | denovo1402\_f0 | denovo1428\_f0 | denovo1506\_f0 | denovo1662\_f0 | denovo1688\_f0 | denovo1740\_f0 | denovo1818\_f0 | denovo1870\_f0 | denovo1948\_f0 | denovo1974\_f0 | denovo2000\_f0 | denovo2026\_f0 | denovo2104\_f0 | denovo2130\_f0 | denovo2156\_f0 | denovo2260\_f0 | denovo2286\_f0 | denovo2312\_f0 | denovo2338\_f0 | denovo2390\_f0 | denovo2494\_f0 | denovo2546\_f0 | denovo2572\_f0 | denovo2598\_f0 | denovo2624\_f0 | denovo2650\_f0 | denovo2676\_f0 | denovo2780\_f0 | denovo2858\_f0 | denovo2910\_f0 | denovo3170\_f0 | denovo3274\_f0 | denovo3326\_f0 | denovo3352\_f0 | denovo3456\_f0 | denovo3508\_f0 | denovo3612\_f0 | denovo3638\_f0 | denovo3664\_f0 | denovo3742\_f0 | denovo3768\_f0 | denovo3820\_f0 | denovo3846\_f0 | denovo4002\_f0 | denovo4080\_f0 | denovo4158\_f0 | denovo4262\_f0 | denovo4340\_f0 | denovo4366\_f0 | denovo4392\_f0 | denovo4418\_f0 | denovo4444\_f0 | denovo4522\_f0 | denovo4548\_f0 | denovo4652\_f0 | denovo4782\_f0 | denovo4860\_f0 | denovo4912\_f0 | denovo4938\_f0 | denovo4964\_f0 | denovo4990\_f0 | denovo5016\_f0 | denovo5120\_f0 | denovo5172\_f0 | denovo5224\_f0 | denovo5432\_f0 | denovo5458\_f0 | denovo5562\_f0 | denovo5588\_f0 | denovo5926\_f0 | denovo5978\_f0 | denovo6030\_f0 | denovo6056\_f0 | denovo6082\_f0 | denovo6108\_f0 | denovo6212\_f0 | denovo6264\_f0 | denovo6290\_f0 | denovo6316\_f0 | denovo6394\_f0 | denovo6524\_f0 | denovo6576\_f0 | denovo6602\_f0 | denovo6628\_f0 | denovo6680\_f0 | denovo6706\_f0 | denovo6732\_f0 | denovo6758\_f0 | denovo6784\_f0 | denovo6914\_f0 | denovo6992\_f0 | denovo7018\_f0 | denovo7044\_f0 | denovo7096\_f0 | denovo7148\_f0 | denovo7174\_f0 | denovo7304\_f0 | denovo7330\_f0 | denovo7356\_f0 | denovo7382\_f0 | denovo7408\_f0 | denovo7486\_f0 | denovo7512\_f0 | denovo7538\_f0 | denovo7564\_f0 | denovo7642\_f0 | denovo7694\_f0 | denovo7720\_f0 | denovo7798\_f0 | denovo7850\_f0 | denovo7928\_f0 | denovo8032\_f0 | denovo8058\_f0 | denovo8240\_f0 | denovo8266\_f0 | denovo8292\_f0 | denovo8370\_f0 | denovo8396\_f0 | denovo8422\_f0 | denovo8500\_f0 | denovo8552\_f0 | denovo8578\_f0 | denovo8630\_f0 | denovo8656\_f0 | denovo8708\_f0 | denovo8734\_f0 | denovo8760\_f0 | denovo8786\_f0 | denovo8916\_f0 | denovo8942\_f0 | denovo8968\_f0 | denovo8994\_f0 | denovo9124\_f0 | denovo9150\_f0 | denovo9254\_f0 | denovo9306\_f0 | denovo9332\_f0 | denovo9358\_f0 | denovo9462\_f0 | denovo9488\_f0 | denovo9514\_f0 | denovo9670\_f0 | denovo9722\_f0 | denovo9800\_f0 | denovo9826\_f0 | denovo9852\_f0 | denovo9904\_f0 | denovo9982\_f0 | denovo10034\_f0 | denovo10060\_f0 | denovo10216\_f0 | denovo10242\_f0 | denovo10294\_f0 | denovo10320\_f0 | denovo10450\_f0 | denovo10476\_f0 | denovo10528\_f0 | denovo10554\_f0 | denovo10580\_f0 | denovo10606\_f0 | denovo10684\_f0 | denovo10736\_f0 | denovo10762\_f0 | denovo10788\_f0 | denovo10840\_f0 | denovo11022\_f0 | denovo11048\_f0 | denovo11074\_f0 | denovo11204\_f0 | denovo11308\_f0 | denovo11360\_f0 | denovo11412\_f0 | denovo11438\_f0 | denovo11464\_f0 | denovo11490\_f0 | denovo11516\_f0 | denovo11542\_f0 | denovo11594\_f0 | denovo11620\_f0 | denovo11724\_f0 | denovo11750\_f0 | denovo11932\_f0 | denovo11958\_f0 | denovo12088\_f0 |
| SW130103 | Anigraea sp. | denovo23\_f0 | denovo101\_f0 | denovo179\_f0 | denovo205\_f0 | denovo283\_f0 | denovo335\_f0 | denovo387\_f0 | denovo465\_f0 | denovo543\_f0 | denovo699\_f0 | denovo855\_f0 | denovo1063\_f0 | denovo1167\_f0 | denovo1245\_f0 | denovo1271\_f0 | denovo1375\_f0 | denovo1401\_f0 | denovo1427\_f0 | denovo1505\_f0 | denovo1661\_f0 | denovo1687\_f0 | denovo1739\_f0 | denovo1817\_f0 | denovo1869\_f0 | denovo1947\_f0 | denovo1973\_f0 | denovo1999\_f0 | denovo2025\_f0 | denovo2103\_f0 | denovo2129\_f0 | denovo2155\_f0 | denovo2259\_f0 | denovo2285\_f0 | denovo2311\_f0 | denovo2337\_f0 | denovo2389\_f0 | denovo2493\_f0 | denovo2545\_f0 | denovo2571\_f0 | denovo2597\_f0 | denovo2623\_f0 | denovo2649\_f0 | denovo2675\_f0 | denovo2779\_f0 | denovo2857\_f0 | denovo2909\_f0 | denovo3169\_f0 | denovo3273\_f0 | denovo3325\_f0 | denovo3351\_f0 | denovo3455\_f0 | denovo3507\_f0 | denovo3611\_f0 | denovo3637\_f0 | denovo3663\_f0 | denovo3741\_f0 | denovo3767\_f0 | denovo3819\_f0 | denovo3845\_f0 | denovo4001\_f0 | denovo4079\_f0 | denovo4157\_f0 | denovo4261\_f0 | denovo4339\_f0 | denovo4365\_f0 | denovo4391\_f0 | denovo4417\_f0 | denovo4443\_f0 | denovo4521\_f0 | denovo4547\_f0 | denovo4651\_f0 | denovo4781\_f0 | denovo4859\_f0 | denovo4911\_f0 | denovo4937\_f0 | denovo4963\_f0 | denovo4989\_f0 | denovo5015\_f0 | denovo5119\_f0 | denovo5171\_f0 | denovo5223\_f0 | denovo5431\_f0 | denovo5457\_f0 | denovo5561\_f0 | denovo5587\_f0 | denovo5925\_f0 | denovo5977\_f0 | denovo6029\_f0 | denovo6055\_f0 | denovo6081\_f0 | denovo6107\_f0 | denovo6211\_f0 | denovo6263\_f0 | denovo6289\_f0 | denovo6315\_f0 | denovo6393\_f0 | denovo6523\_f0 | denovo6575\_f0 | denovo6601\_f0 | denovo6627\_f0 | denovo6679\_f0 | denovo6705\_f0 | denovo6731\_f0 | denovo6757\_f0 | denovo6783\_f0 | denovo6913\_f0 | denovo6991\_f0 | denovo7017\_f0 | denovo7043\_f0 | denovo7095\_f0 | denovo7147\_f0 | denovo7173\_f0 | denovo7303\_f0 | denovo7329\_f0 | denovo7355\_f0 | denovo7381\_f0 | denovo7407\_f0 | denovo7485\_f0 | denovo7511\_f0 | denovo7537\_f0 | denovo7563\_f0 | denovo7641\_f0 | denovo7693\_f0 | denovo7719\_f0 | denovo7797\_f0 | denovo7849\_f0 | denovo7927\_f0 | denovo8031\_f0 | denovo8057\_f0 | denovo8239\_f0 | denovo8265\_f0 | denovo8291\_f0 | denovo8369\_f0 | denovo8395\_f0 | denovo8421\_f0 | denovo8499\_f0 | denovo8551\_f0 | denovo8577\_f0 | denovo8629\_f0 | denovo8655\_f0 | denovo8707\_f0 | denovo8733\_f0 | denovo8759\_f0 | denovo8785\_f0 | denovo8915\_f0 | denovo8941\_f0 | denovo8967\_f0 | denovo8993\_f0 | denovo9123\_f0 | denovo9149\_f0 | denovo9253\_f0 | denovo9305\_f0 | denovo9331\_f0 | denovo9357\_f0 | denovo9461\_f0 | denovo9487\_f0 | denovo9513\_f0 | denovo9669\_f0 | denovo9721\_f0 | denovo9799\_f0 | denovo9825\_f0 | denovo9851\_f0 | denovo9903\_f0 | denovo9981\_f0 | denovo10033\_f0 | denovo10059\_f0 | denovo10215\_f0 | denovo10241\_f0 | denovo10293\_f0 | denovo10319\_f0 | denovo10449\_f0 | denovo10475\_f0 | denovo10527\_f0 | denovo10553\_f0 | denovo10579\_f0 | denovo10605\_f0 | denovo10683\_f0 | denovo10735\_f0 | denovo10761\_f0 | denovo10787\_f0 | denovo10839\_f0 | denovo11021\_f0 | denovo11047\_f0 | denovo11073\_f0 | denovo11203\_f0 | denovo11307\_f0 | denovo11359\_f0 | denovo11411\_f0 | denovo11437\_f0 | denovo11463\_f0 | denovo11489\_f0 | denovo11515\_f0 | denovo11541\_f0 | denovo11593\_f0 | denovo11619\_f0 | denovo11723\_f0 | denovo11749\_f0 | denovo11931\_f0 | denovo11957\_f0 | denovo12087\_f0 |
| Callid | Pterodecta felderi\* | denovo1\_f0 | denovo79\_f0 | denovo157\_f0 | denovo183\_f0 | denovo261\_f0 | denovo313\_f0 | denovo365\_f0 | denovo443\_f0 | denovo521\_f0 | denovo677\_f0 | denovo833\_f0 | denovo1041\_f0 | denovo1145\_f0 | denovo1223\_f0 | denovo1249\_f0 | denovo1353\_f0 | denovo1379\_f0 | denovo1405\_f0 | denovo1483\_f0 | denovo1639\_f0 | denovo1665\_f0 | denovo1717\_f0 | denovo1795\_f0 | denovo1847\_f0 | denovo1925\_f0 | denovo1951\_f0 | denovo1977\_f0 | denovo2003\_f0 | denovo2081\_f0 | denovo2107\_f0 | denovo2133\_f0 | denovo2237\_f0 | denovo2263\_f0 | denovo2289\_f0 | denovo2315\_f0 | denovo2367\_f0 | denovo2471\_f0 | denovo2523\_f0 | denovo2549\_f0 | denovo2575\_f0 | denovo2601\_f0 | denovo2627\_f0 | denovo2653\_f0 | denovo2757\_f0 | denovo2835\_f0 | denovo2887\_f0 | denovo3147\_f0 | denovo3251\_f0 | denovo3303\_f0 | denovo3329\_f0 | denovo3433\_f0 | denovo3485\_f0 | denovo3589\_f0 | denovo3615\_f0 | denovo3641\_f0 | denovo3719\_f0 | denovo3745\_f0 | denovo3797\_f0 | denovo3823\_f0 | denovo3979\_f0 | denovo4057\_f0 | denovo4135\_f0 | denovo4239\_f0 | denovo4317\_f0 | denovo4343\_f0 | denovo4369\_f0 | denovo4395\_f0 | denovo4421\_f0 | denovo4499\_f0 | denovo4525\_f0 | denovo4629\_f0 | denovo4759\_f0 | denovo4837\_f0 | denovo4889\_f0 | denovo4915\_f0 | denovo4941\_f0 | denovo4967\_f0 | denovo4993\_f0 | denovo5097\_f0 | denovo5149\_f0 | denovo5201\_f0 | denovo5409\_f0 | denovo5435\_f0 | denovo5539\_f0 | denovo5565\_f0 | denovo5903\_f0 | denovo5955\_f0 | denovo6007\_f0 | denovo6033\_f0 | denovo6059\_f0 | denovo6085\_f0 | denovo6189\_f0 | denovo6241\_f0 | denovo6267\_f0 | denovo6293\_f0 | denovo6371\_f0 | denovo6501\_f0 | denovo6553\_f0 | denovo6579\_f0 | denovo6605\_f0 | denovo6657\_f0 | denovo6683\_f0 | denovo6709\_f0 | denovo6735\_f0 | denovo6761\_f0 | denovo6891\_f0 | denovo6969\_f0 | denovo6995\_f0 | denovo7021\_f0 | denovo7073\_f0 | denovo7125\_f0 | denovo7151\_f0 | denovo7281\_f0 | denovo7307\_f0 | denovo7333\_f0 | denovo7359\_f0 | denovo7385\_f0 | denovo7463\_f0 | denovo7489\_f0 | denovo7515\_f0 | denovo7541\_f0 | denovo7619\_f0 | denovo7671\_f0 | denovo7697\_f0 | denovo7775\_f0 | denovo7827\_f0 | denovo7905\_f0 | denovo8009\_f0 | denovo8035\_f0 | denovo8217\_f0 | denovo8243\_f0 | denovo8269\_f0 | denovo8347\_f0 | denovo8373\_f0 | denovo8399\_f0 | denovo8477\_f0 | denovo8529\_f0 | denovo8555\_f0 | denovo8607\_f0 | denovo8633\_f0 | denovo8685\_f0 | denovo8711\_f0 | denovo8737\_f0 | denovo8763\_f0 | denovo8893\_f0 | denovo8919\_f0 | denovo8945\_f0 | denovo8971\_f0 | denovo9101\_f0 | denovo9127\_f0 | denovo9231\_f0 | denovo9283\_f0 | denovo9309\_f0 | denovo9335\_f0 | denovo9439\_f0 | denovo9465\_f0 | denovo9491\_f0 | denovo9647\_f0 | denovo9699\_f0 | denovo9777\_f0 | denovo9803\_f0 | denovo9829\_f0 | denovo9881\_f0 | denovo9959\_f0 | denovo10011\_f0 | denovo10037\_f0 | denovo10193\_f0 | denovo10219\_f0 | denovo10271\_f0 | denovo10297\_f0 | denovo10427\_f0 | denovo10453\_f0 | denovo10505\_f0 | denovo10531\_f0 | denovo10557\_f0 | denovo10583\_f0 | denovo10661\_f0 | denovo10713\_f0 | denovo10739\_f0 | denovo10765\_f0 | denovo10817\_f0 | denovo10999\_f0 | denovo11025\_f0 | denovo11051\_f0 | denovo11181\_f0 | denovo11285\_f0 | denovo11337\_f0 | denovo11389\_f0 | denovo11415\_f0 | denovo11441\_f0 | denovo11467\_f0 | denovo11493\_f0 | denovo11519\_f0 | denovo11571\_f0 | denovo11597\_f0 | denovo11701\_f0 | denovo11727\_f0 | denovo11909\_f0 | denovo11935\_f0 | denovo12065\_f0 |
| FG120070B | Artace sp.\* | denovo8\_f0 | denovo86\_f0 | denovo164\_f0 | denovo190\_f0 | denovo268\_f0 | denovo320\_f0 | denovo372\_f0 | denovo450\_f0 | denovo528\_f0 | denovo684\_f0 | denovo840\_f0 | denovo1048\_f0 | denovo1152\_f0 | denovo1230\_f0 | denovo1256\_f0 | denovo1360\_f0 | denovo1386\_f0 | denovo1412\_f0 | denovo1490\_f0 | denovo1646\_f0 | denovo1672\_f0 | denovo1724\_f0 | denovo1802\_f0 | denovo1854\_f0 | denovo1932\_f0 | denovo1958\_f0 | denovo1984\_f0 | denovo2010\_f0 | denovo2088\_f0 | denovo2114\_f0 | denovo2140\_f0 | denovo2244\_f0 | denovo2270\_f0 | denovo2296\_f0 | denovo2322\_f0 | denovo2374\_f0 | denovo2478\_f0 | denovo2530\_f0 | denovo2556\_f0 | denovo2582\_f0 | denovo2608\_f0 | denovo2634\_f0 | denovo2660\_f0 | denovo2764\_f0 | denovo2842\_f0 | denovo2894\_f0 | denovo3154\_f0 | denovo3258\_f0 | denovo3310\_f0 | denovo3336\_f0 | denovo3440\_f0 | denovo3492\_f0 | denovo3596\_f0 | denovo3622\_f0 | denovo3648\_f0 | denovo3726\_f0 | denovo3752\_f0 | denovo3804\_f0 | denovo3830\_f0 | denovo3986\_f0 | denovo4064\_f0 | denovo4142\_f0 | denovo4246\_f0 | denovo4324\_f0 | denovo4350\_f0 | denovo4376\_f0 | denovo4402\_f0 | denovo4428\_f0 | denovo4506\_f0 | denovo4532\_f0 | denovo4636\_f0 | denovo4766\_f0 | denovo4844\_f0 | denovo4896\_f0 | denovo4922\_f0 | denovo4948\_f0 | denovo4974\_f0 | denovo5000\_f0 | denovo5104\_f0 | denovo5156\_f0 | denovo5208\_f0 | denovo5416\_f0 | denovo5442\_f0 | denovo5546\_f0 | denovo5572\_f0 | denovo5910\_f0 | denovo5962\_f0 | denovo6014\_f0 | denovo6040\_f0 | denovo6066\_f0 | denovo6092\_f0 | denovo6196\_f0 | denovo6248\_f0 | denovo6274\_f0 | denovo6300\_f0 | denovo6378\_f0 | denovo6508\_f0 | denovo6560\_f0 | denovo6586\_f0 | denovo6612\_f0 | denovo6664\_f0 | denovo6690\_f0 | denovo6716\_f0 | denovo6742\_f0 | denovo6768\_f0 | denovo6898\_f0 | denovo6976\_f0 | denovo7002\_f0 | denovo7028\_f0 | denovo7080\_f0 | denovo7132\_f0 | denovo7158\_f0 | denovo7288\_f0 | denovo7314\_f0 | denovo7340\_f0 | denovo7366\_f0 | denovo7392\_f0 | denovo7470\_f0 | denovo7496\_f0 | denovo7522\_f0 | denovo7548\_f0 | denovo7626\_f0 | denovo7678\_f0 | denovo7704\_f0 | denovo7782\_f0 | denovo7834\_f0 | denovo7912\_f0 | denovo8016\_f0 | denovo8042\_f0 | denovo8224\_f0 | denovo8250\_f0 | denovo8276\_f0 | denovo8354\_f0 | denovo8380\_f0 | denovo8406\_f0 | denovo8484\_f0 | denovo8536\_f0 | denovo8562\_f0 | denovo8614\_f0 | denovo8640\_f0 | denovo8692\_f0 | denovo8718\_f0 | denovo8744\_f0 | denovo8770\_f0 | denovo8900\_f0 | denovo8926\_f0 | denovo8952\_f0 | denovo8978\_f0 | denovo9108\_f0 | denovo9134\_f0 | denovo9238\_f0 | denovo9290\_f0 | denovo9316\_f0 | denovo9342\_f0 | denovo9446\_f0 | denovo9472\_f0 | denovo9498\_f0 | denovo9654\_f0 | denovo9706\_f0 | denovo9784\_f0 | denovo9810\_f0 | denovo9836\_f0 | denovo9888\_f0 | denovo9966\_f0 | denovo10018\_f0 | denovo10044\_f0 | denovo10200\_f0 | denovo10226\_f0 | denovo10278\_f0 | denovo10304\_f0 | denovo10434\_f0 | denovo10460\_f0 | denovo10512\_f0 | denovo10538\_f0 | denovo10564\_f0 | denovo10590\_f0 | denovo10668\_f0 | denovo10720\_f0 | denovo10746\_f0 | denovo10772\_f0 | denovo10824\_f0 | denovo11006\_f0 | denovo11032\_f0 | denovo11058\_f0 | denovo11188\_f0 | denovo11292\_f0 | denovo11344\_f0 | denovo11396\_f0 | denovo11422\_f0 | denovo11448\_f0 | denovo11474\_f0 | denovo11500\_f0 | denovo11526\_f0 | denovo11578\_f0 | denovo11604\_f0 | denovo11708\_f0 | denovo11734\_f0 | denovo11916\_f0 | denovo11942\_f0 | denovo12072\_f0 |
| SW130007 | Thubana sp.\* | denovo22\_f0 | denovo100\_f0 | denovo178\_f0 | denovo204\_f0 | denovo282\_f0 | denovo334\_f0 | denovo386\_f0 | denovo464\_f0 | denovo542\_f0 | denovo698\_f0 | denovo854\_f0 | denovo1062\_f0 | denovo1166\_f0 | denovo1244\_f0 | denovo1270\_f0 | denovo1374\_f0 | denovo1400\_f0 | denovo1426\_f0 | denovo1504\_f0 | denovo1660\_f0 | denovo1686\_f0 | denovo1738\_f0 | denovo1816\_f0 | denovo1868\_f0 | denovo1946\_f0 | denovo1972\_f0 | denovo1998\_f0 | denovo2024\_f0 | denovo2102\_f0 | denovo2128\_f0 | denovo2154\_f0 | denovo2258\_f0 | denovo2284\_f0 | denovo2310\_f0 | denovo2336\_f0 | denovo2388\_f0 | denovo2492\_f0 | denovo2544\_f0 | denovo2570\_f0 | denovo2596\_f0 | denovo2622\_f0 | denovo2648\_f0 | denovo2674\_f0 | denovo2778\_f0 | denovo2856\_f0 | denovo2908\_f0 | denovo3168\_f0 | denovo3272\_f0 | denovo3324\_f0 | denovo3350\_f0 | denovo3454\_f0 | denovo3506\_f0 | denovo3610\_f0 | denovo3636\_f0 | denovo3662\_f0 | denovo3740\_f0 | denovo3766\_f0 | denovo3818\_f0 | denovo3844\_f0 | denovo4000\_f0 | denovo4078\_f0 | denovo4156\_f0 | denovo4260\_f0 | denovo4338\_f0 | denovo4364\_f0 | denovo4390\_f0 | denovo4416\_f0 | denovo4442\_f0 | denovo4520\_f0 | denovo4546\_f0 | denovo4650\_f0 | denovo4780\_f0 | denovo4858\_f0 | denovo4910\_f0 | denovo4936\_f0 | denovo4962\_f0 | denovo4988\_f0 | denovo5014\_f0 | denovo5118\_f0 | denovo5170\_f0 | denovo5222\_f0 | denovo5430\_f0 | denovo5456\_f0 | denovo5560\_f0 | denovo5586\_f0 | denovo5924\_f0 | denovo5976\_f0 | denovo6028\_f0 | denovo6054\_f0 | denovo6080\_f0 | denovo6106\_f0 | denovo6210\_f0 | denovo6262\_f0 | denovo6288\_f0 | denovo6314\_f0 | denovo6392\_f0 | denovo6522\_f0 | denovo6574\_f0 | denovo6600\_f0 | denovo6626\_f0 | denovo6678\_f0 | denovo6704\_f0 | denovo6730\_f0 | denovo6756\_f0 | denovo6782\_f0 | denovo6912\_f0 | denovo6990\_f0 | denovo7016\_f0 | denovo7042\_f0 | denovo7094\_f0 | denovo7146\_f0 | denovo7172\_f0 | denovo7302\_f0 | denovo7328\_f0 | denovo7354\_f0 | denovo7380\_f0 | denovo7406\_f0 | denovo7484\_f0 | denovo7510\_f0 | denovo7536\_f0 | denovo7562\_f0 | denovo7640\_f0 | denovo7692\_f0 | denovo7718\_f0 | denovo7796\_f0 | denovo7848\_f0 | denovo7926\_f0 | denovo8030\_f0 | denovo8056\_f0 | denovo8238\_f0 | denovo8264\_f0 | denovo8290\_f0 | denovo8368\_f0 | denovo8394\_f0 | denovo8420\_f0 | denovo8498\_f0 | denovo8550\_f0 | denovo8576\_f0 | denovo8628\_f0 | denovo8654\_f0 | denovo8706\_f0 | denovo8732\_f0 | denovo8758\_f0 | denovo8784\_f0 | denovo8914\_f0 | denovo8940\_f0 | denovo8966\_f0 | denovo8992\_f0 | denovo9122\_f0 | denovo9148\_f0 | denovo9252\_f0 | denovo9304\_f0 | denovo9330\_f0 | denovo9356\_f0 | denovo9460\_f0 | denovo9486\_f0 | denovo9512\_f0 | denovo9668\_f0 | denovo9720\_f0 | denovo9798\_f0 | denovo9824\_f0 | denovo9850\_f0 | denovo9902\_f0 | denovo9980\_f0 | denovo10032\_f0 | denovo10058\_f0 | denovo10214\_f0 | denovo10240\_f0 | denovo10292\_f0 | denovo10318\_f0 | denovo10448\_f0 | denovo10474\_f0 | denovo10526\_f0 | denovo10552\_f0 | denovo10578\_f0 | denovo10604\_f0 | denovo10682\_f0 | denovo10734\_f0 | denovo10760\_f0 | denovo10786\_f0 | denovo10838\_f0 | denovo11020\_f0 | denovo11046\_f0 | denovo11072\_f0 | denovo11202\_f0 | denovo11306\_f0 | denovo11358\_f0 | denovo11410\_f0 | denovo11436\_f0 | denovo11462\_f0 | denovo11488\_f0 | denovo11514\_f0 | denovo11540\_f0 | denovo11592\_f0 | denovo11618\_f0 | denovo11722\_f0 | denovo11748\_f0 | denovo11930\_f0 | denovo11956\_f0 | denovo12086\_f0 |
| GNV120032 | Nemoria lixaria\* | denovo14\_f0 | denovo92\_f0 | denovo170\_f0 | denovo196\_f0 | denovo274\_f0 | denovo326\_f0 | denovo378\_f0 | denovo456\_f0 | denovo534\_f0 | denovo690\_f0 | denovo846\_f0 | denovo1054\_f0 | denovo1158\_f0 | denovo1236\_f0 | denovo1262\_f0 | denovo1366\_f0 | denovo1392\_f0 | denovo1418\_f0 | denovo1496\_f0 | denovo1652\_f0 | denovo1678\_f0 | denovo1730\_f0 | denovo1808\_f0 | denovo1860\_f0 | denovo1938\_f0 | denovo1964\_f0 | denovo1990\_f0 | denovo2016\_f0 | denovo2094\_f0 | denovo2120\_f0 | denovo2146\_f0 | denovo2250\_f0 | denovo2276\_f0 | denovo2302\_f0 | denovo2328\_f0 | denovo2380\_f0 | denovo2484\_f0 | denovo2536\_f0 | denovo2562\_f0 | denovo2588\_f0 | denovo2614\_f0 | denovo2640\_f0 | denovo2666\_f0 | denovo2770\_f0 | denovo2848\_f0 | denovo2900\_f0 | denovo3160\_f0 | denovo3264\_f0 | denovo3316\_f0 | denovo3342\_f0 | denovo3446\_f0 | denovo3498\_f0 | denovo3602\_f0 | denovo3628\_f0 | denovo3654\_f0 | denovo3732\_f0 | denovo3758\_f0 | denovo3810\_f0 | denovo3836\_f0 | denovo3992\_f0 | denovo4070\_f0 | denovo4148\_f0 | denovo4252\_f0 | denovo4330\_f0 | denovo4356\_f0 | denovo4382\_f0 | denovo4408\_f0 | denovo4434\_f0 | denovo4512\_f0 | denovo4538\_f0 | denovo4642\_f0 | denovo4772\_f0 | denovo4850\_f0 | denovo4902\_f0 | denovo4928\_f0 | denovo4954\_f0 | denovo4980\_f0 | denovo5006\_f0 | denovo5110\_f0 | denovo5162\_f0 | denovo5214\_f0 | denovo5422\_f0 | denovo5448\_f0 | denovo5552\_f0 | denovo5578\_f0 | denovo5916\_f0 | denovo5968\_f0 | denovo6020\_f0 | denovo6046\_f0 | denovo6072\_f0 | denovo6098\_f0 | denovo6202\_f0 | denovo6254\_f0 | denovo6280\_f0 | denovo6306\_f0 | denovo6384\_f0 | denovo6514\_f0 | denovo6566\_f0 | denovo6592\_f0 | denovo6618\_f0 | denovo6670\_f0 | denovo6696\_f0 | denovo6722\_f0 | denovo6748\_f0 | denovo6774\_f0 | denovo6904\_f0 | denovo6982\_f0 | denovo7008\_f0 | denovo7034\_f0 | denovo7086\_f0 | denovo7138\_f0 | denovo7164\_f0 | denovo7294\_f0 | denovo7320\_f0 | denovo7346\_f0 | denovo7372\_f0 | denovo7398\_f0 | denovo7476\_f0 | denovo7502\_f0 | denovo7528\_f0 | denovo7554\_f0 | denovo7632\_f0 | denovo7684\_f0 | denovo7710\_f0 | denovo7788\_f0 | denovo7840\_f0 | denovo7918\_f0 | denovo8022\_f0 | denovo8048\_f0 | denovo8230\_f0 | denovo8256\_f0 | denovo8282\_f0 | denovo8360\_f0 | denovo8386\_f0 | denovo8412\_f0 | denovo8490\_f0 | denovo8542\_f0 | denovo8568\_f0 | denovo8620\_f0 | denovo8646\_f0 | denovo8698\_f0 | denovo8724\_f0 | denovo8750\_f0 | denovo8776\_f0 | denovo8906\_f0 | denovo8932\_f0 | denovo8958\_f0 | denovo8984\_f0 | denovo9114\_f0 | denovo9140\_f0 | denovo9244\_f0 | denovo9296\_f0 | denovo9322\_f0 | denovo9348\_f0 | denovo9452\_f0 | denovo9478\_f0 | denovo9504\_f0 | denovo9660\_f0 | denovo9712\_f0 | denovo9790\_f0 | denovo9816\_f0 | denovo9842\_f0 | denovo9894\_f0 | denovo9972\_f0 | denovo10024\_f0 | denovo10050\_f0 | denovo10206\_f0 | denovo10232\_f0 | denovo10284\_f0 | denovo10310\_f0 | denovo10440\_f0 | denovo10466\_f0 | denovo10518\_f0 | denovo10544\_f0 | denovo10570\_f0 | denovo10596\_f0 | denovo10674\_f0 | denovo10726\_f0 | denovo10752\_f0 | denovo10778\_f0 | denovo10830\_f0 | denovo11012\_f0 | denovo11038\_f0 | denovo11064\_f0 | denovo11194\_f0 | denovo11298\_f0 | denovo11350\_f0 | denovo11402\_f0 | denovo11428\_f0 | denovo11454\_f0 | denovo11480\_f0 | denovo11506\_f0 | denovo11532\_f0 | denovo11584\_f0 | denovo11610\_f0 | denovo11714\_f0 | denovo11740\_f0 | denovo11922\_f0 | denovo11948\_f0 | denovo12078\_f0 |
| PXYLO | Plutella xylostella\* | denovo18\_f0 | denovo96\_f0 | denovo174\_f0 | denovo200\_f0 | denovo278\_f0 | denovo330\_f0 | denovo382\_f0 | denovo460\_f0 | denovo538\_f0 | denovo694\_f0 | denovo850\_f0 | denovo1058\_f0 | denovo1162\_f0 | denovo1240\_f0 | denovo1266\_f0 | denovo1370\_f0 | denovo1396\_f0 | denovo1422\_f0 | denovo1500\_f0 | denovo1656\_f0 | denovo1682\_f0 | denovo1734\_f0 | denovo1812\_f0 | denovo1864\_f0 | denovo1942\_f0 | denovo1968\_f0 | denovo1994\_f0 | denovo2020\_f0 | denovo2098\_f0 | denovo2124\_f0 | denovo2150\_f0 | denovo2254\_f0 | denovo2280\_f0 | denovo2306\_f0 | denovo2332\_f0 | denovo2384\_f0 | denovo2488\_f0 | denovo2540\_f0 | denovo2566\_f0 | denovo2592\_f0 | denovo2618\_f0 | denovo2644\_f0 | denovo2670\_f0 | denovo2774\_f0 | denovo2852\_f0 | denovo2904\_f0 | denovo3164\_f0 | denovo3268\_f0 | denovo3320\_f0 | denovo3346\_f0 | denovo3450\_f0 | denovo3502\_f0 | denovo3606\_f0 | denovo3632\_f0 | denovo3658\_f0 | denovo3736\_f0 | denovo3762\_f0 | denovo3814\_f0 | denovo3840\_f0 | denovo3996\_f0 | denovo4074\_f0 | denovo4152\_f0 | denovo4256\_f0 | denovo4334\_f0 | denovo4360\_f0 | denovo4386\_f0 | denovo4412\_f0 | denovo4438\_f0 | denovo4516\_f0 | denovo4542\_f0 | denovo4646\_f0 | denovo4776\_f0 | denovo4854\_f0 | denovo4906\_f0 | denovo4932\_f0 | denovo4958\_f0 | denovo4984\_f0 | denovo5010\_f0 | denovo5114\_f0 | denovo5166\_f0 | denovo5218\_f0 | denovo5426\_f0 | denovo5452\_f0 | denovo5556\_f0 | denovo5582\_f0 | denovo5920\_f0 | denovo5972\_f0 | denovo6024\_f0 | denovo6050\_f0 | denovo6076\_f0 | denovo6102\_f0 | denovo6206\_f0 | denovo6258\_f0 | denovo6284\_f0 | denovo6310\_f0 | denovo6388\_f0 | denovo6518\_f0 | denovo6570\_f0 | denovo6596\_f0 | denovo6622\_f0 | denovo6674\_f0 | denovo6700\_f0 | denovo6726\_f0 | denovo6752\_f0 | denovo6778\_f0 | denovo6908\_f0 | denovo6986\_f0 | denovo7012\_f0 | denovo7038\_f0 | denovo7090\_f0 | denovo7142\_f0 | denovo7168\_f0 | denovo7298\_f0 | denovo7324\_f0 | denovo7350\_f0 | denovo7376\_f0 | denovo7402\_f0 | denovo7480\_f0 | denovo7506\_f0 | denovo7532\_f0 | denovo7558\_f0 | denovo7636\_f0 | denovo7688\_f0 | denovo7714\_f0 | denovo7792\_f0 | denovo7844\_f0 | denovo7922\_f0 | denovo8026\_f0 | denovo8052\_f0 | denovo8234\_f0 | denovo8260\_f0 | denovo8286\_f0 | denovo8364\_f0 | denovo8390\_f0 | denovo8416\_f0 | denovo8494\_f0 | denovo8546\_f0 | denovo8572\_f0 | denovo8624\_f0 | denovo8650\_f0 | denovo8702\_f0 | denovo8728\_f0 | denovo8754\_f0 | denovo8780\_f0 | denovo8910\_f0 | denovo8936\_f0 | denovo8962\_f0 | denovo8988\_f0 | denovo9118\_f0 | denovo9144\_f0 | denovo9248\_f0 | denovo9300\_f0 | denovo9326\_f0 | denovo9352\_f0 | denovo9456\_f0 | denovo9482\_f0 | denovo9508\_f0 | denovo9664\_f0 | denovo9716\_f0 | denovo9794\_f0 | denovo9820\_f0 | denovo9846\_f0 | denovo9898\_f0 | denovo9976\_f0 | denovo10028\_f0 | denovo10054\_f0 | denovo10210\_f0 | denovo10236\_f0 | denovo10288\_f0 | denovo10314\_f0 | denovo10444\_f0 | denovo10470\_f0 | denovo10522\_f0 | denovo10548\_f0 | denovo10574\_f0 | denovo10600\_f0 | denovo10678\_f0 | denovo10730\_f0 | denovo10756\_f0 | denovo10782\_f0 | denovo10834\_f0 | denovo11016\_f0 | denovo11042\_f0 | denovo11068\_f0 | denovo11198\_f0 | denovo11302\_f0 | denovo11354\_f0 | denovo11406\_f0 | denovo11432\_f0 | denovo11458\_f0 | denovo11484\_f0 | denovo11510\_f0 | denovo11536\_f0 | denovo11588\_f0 | denovo11614\_f0 | denovo11718\_f0 | denovo11744\_f0 | denovo11926\_f0 | denovo11952\_f0 | denovo12082\_f0 |
| FG120079 | Zeuzerodes maculata\* | denovo11\_f0 | denovo89\_f0 | denovo167\_f0 | denovo193\_f0 | denovo271\_f0 | denovo323\_f0 | denovo375\_f0 | denovo453\_f0 | denovo531\_f0 | denovo687\_f0 | denovo843\_f0 | denovo1051\_f0 | denovo1155\_f0 | denovo1233\_f0 | denovo1259\_f0 | denovo1363\_f0 | denovo1389\_f0 | denovo1415\_f0 | denovo1493\_f0 | denovo1649\_f0 | denovo1675\_f0 | denovo1727\_f0 | denovo1805\_f0 | denovo1857\_f0 | denovo1935\_f0 | denovo1961\_f0 | denovo1987\_f0 | denovo2013\_f0 | denovo2091\_f0 | denovo2117\_f0 | denovo2143\_f0 | denovo2247\_f0 | denovo2273\_f0 | denovo2299\_f0 | denovo2325\_f0 | denovo2377\_f0 | denovo2481\_f0 | denovo2533\_f0 | denovo2559\_f0 | denovo2585\_f0 | denovo2611\_f0 | denovo2637\_f0 | denovo2663\_f0 | denovo2767\_f0 | denovo2845\_f0 | denovo2897\_f0 | denovo3157\_f0 | denovo3261\_f0 | denovo3313\_f0 | denovo3339\_f0 | denovo3443\_f0 | denovo3495\_f0 | denovo3599\_f0 | denovo3625\_f0 | denovo3651\_f0 | denovo3729\_f0 | denovo3755\_f0 | denovo3807\_f0 | denovo3833\_f0 | denovo3989\_f0 | denovo4067\_f0 | denovo4145\_f0 | denovo4249\_f0 | denovo4327\_f0 | denovo4353\_f0 | denovo4379\_f0 | denovo4405\_f0 | denovo4431\_f0 | denovo4509\_f0 | denovo4535\_f0 | denovo4639\_f0 | denovo4769\_f0 | denovo4847\_f0 | denovo4899\_f0 | denovo4925\_f0 | denovo4951\_f0 | denovo4977\_f0 | denovo5003\_f0 | denovo5107\_f0 | denovo5159\_f0 | denovo5211\_f0 | denovo5419\_f0 | denovo5445\_f0 | denovo5549\_f0 | denovo5575\_f0 | denovo5913\_f0 | denovo5965\_f0 | denovo6017\_f0 | denovo6043\_f0 | denovo6069\_f0 | denovo6095\_f0 | denovo6199\_f0 | denovo6251\_f0 | denovo6277\_f0 | denovo6303\_f0 | denovo6381\_f0 | denovo6511\_f0 | denovo6563\_f0 | denovo6589\_f0 | denovo6615\_f0 | denovo6667\_f0 | denovo6693\_f0 | denovo6719\_f0 | denovo6745\_f0 | denovo6771\_f0 | denovo6901\_f0 | denovo6979\_f0 | denovo7005\_f0 | denovo7031\_f0 | denovo7083\_f0 | denovo7135\_f0 | denovo7161\_f0 | denovo7291\_f0 | denovo7317\_f0 | denovo7343\_f0 | denovo7369\_f0 | denovo7395\_f0 | denovo7473\_f0 | denovo7499\_f0 | denovo7525\_f0 | denovo7551\_f0 | denovo7629\_f0 | denovo7681\_f0 | denovo7707\_f0 | denovo7785\_f0 | denovo7837\_f0 | denovo7915\_f0 | denovo8019\_f0 | denovo8045\_f0 | denovo8227\_f0 | denovo8253\_f0 | denovo8279\_f0 | denovo8357\_f0 | denovo8383\_f0 | denovo8409\_f0 | denovo8487\_f0 | denovo8539\_f0 | denovo8565\_f0 | denovo8617\_f0 | denovo8643\_f0 | denovo8695\_f0 | denovo8721\_f0 | denovo8747\_f0 | denovo8773\_f0 | denovo8903\_f0 | denovo8929\_f0 | denovo8955\_f0 | denovo8981\_f0 | denovo9111\_f0 | denovo9137\_f0 | denovo9241\_f0 | denovo9293\_f0 | denovo9319\_f0 | denovo9345\_f0 | denovo9449\_f0 | denovo9475\_f0 | denovo9501\_f0 | denovo9657\_f0 | denovo9709\_f0 | denovo9787\_f0 | denovo9813\_f0 | denovo9839\_f0 | denovo9891\_f0 | denovo9969\_f0 | denovo10021\_f0 | denovo10047\_f0 | denovo10203\_f0 | denovo10229\_f0 | denovo10281\_f0 | denovo10307\_f0 | denovo10437\_f0 | denovo10463\_f0 | denovo10515\_f0 | denovo10541\_f0 | denovo10567\_f0 | denovo10593\_f0 | denovo10671\_f0 | denovo10723\_f0 | denovo10749\_f0 | denovo10775\_f0 | denovo10827\_f0 | denovo11009\_f0 | denovo11035\_f0 | denovo11061\_f0 | denovo11191\_f0 | denovo11295\_f0 | denovo11347\_f0 | denovo11399\_f0 | denovo11425\_f0 | denovo11451\_f0 | denovo11477\_f0 | denovo11503\_f0 | denovo11529\_f0 | denovo11581\_f0 | denovo11607\_f0 | denovo11711\_f0 | denovo11737\_f0 | denovo11919\_f0 | denovo11945\_f0 | denovo12075\_f0 |
| SRR850324 | Papilio glaucus | denovo21\_f0 | denovo99\_f0 | denovo177\_f0 | denovo203\_f0 | denovo281\_f0 | denovo333\_f0 | denovo385\_f0 | denovo463\_f0 | denovo541\_f0 | denovo697\_f0 | denovo853\_f0 | denovo1061\_f0 | denovo1165\_f0 | denovo1243\_f0 | denovo1269\_f0 | denovo1373\_f0 | denovo1399\_f0 | denovo1425\_f0 | denovo1503\_f0 | denovo1659\_f0 | denovo1685\_f0 | denovo1737\_f0 | denovo1815\_f0 | denovo1867\_f0 | denovo1945\_f0 | denovo1971\_f0 | denovo1997\_f0 | denovo2023\_f0 | denovo2101\_f0 | denovo2127\_f0 | denovo2153\_f0 | denovo2257\_f0 | denovo2283\_f0 | denovo2309\_f0 | denovo2335\_f0 | denovo2387\_f0 | denovo2491\_f0 | denovo2543\_f0 | denovo2569\_f0 | denovo2595\_f0 | denovo2621\_f0 | denovo2647\_f0 | denovo2673\_f0 | denovo2777\_f0 | denovo2855\_f0 | denovo2907\_f0 | denovo3167\_f0 | denovo3271\_f0 | denovo3323\_f0 | denovo3349\_f0 | denovo3453\_f0 | denovo3505\_f0 | denovo3609\_f0 | denovo3635\_f0 | denovo3661\_f0 | denovo3739\_f0 | denovo3765\_f0 | denovo3817\_f0 | denovo3843\_f0 | denovo3999\_f0 | denovo4077\_f0 | denovo4155\_f0 | denovo4259\_f0 | denovo4337\_f0 | denovo4363\_f0 | denovo4389\_f0 | denovo4415\_f0 | denovo4441\_f0 | denovo4519\_f0 | denovo4545\_f0 | denovo4649\_f0 | denovo4779\_f0 | denovo4857\_f0 | denovo4909\_f0 | denovo4935\_f0 | denovo4961\_f0 | denovo4987\_f0 | denovo5013\_f0 | denovo5117\_f0 | denovo5169\_f0 | denovo5221\_f0 | denovo5429\_f0 | denovo5455\_f0 | denovo5559\_f0 | denovo5585\_f0 | denovo5923\_f0 | denovo5975\_f0 | denovo6027\_f0 | denovo6053\_f0 | denovo6079\_f0 | denovo6105\_f0 | denovo6209\_f0 | denovo6261\_f0 | denovo6287\_f0 | denovo6313\_f0 | denovo6391\_f0 | denovo6521\_f0 | denovo6573\_f0 | denovo6599\_f0 | denovo6625\_f0 | denovo6677\_f0 | denovo6703\_f0 | denovo6729\_f0 | denovo6755\_f0 | denovo6781\_f0 | denovo6911\_f0 | denovo6989\_f0 | denovo7015\_f0 | denovo7041\_f0 | denovo7093\_f0 | denovo7145\_f0 | denovo7171\_f0 | denovo7301\_f0 | denovo7327\_f0 | denovo7353\_f0 | denovo7379\_f0 | denovo7405\_f0 | denovo7483\_f0 | denovo7509\_f0 | denovo7535\_f0 | denovo7561\_f0 | denovo7639\_f0 | denovo7691\_f0 | denovo7717\_f0 | denovo7795\_f0 | denovo7847\_f0 | denovo7925\_f0 | denovo8029\_f0 | denovo8055\_f0 | denovo8237\_f0 | denovo8263\_f0 | denovo8289\_f0 | denovo8367\_f0 | denovo8393\_f0 | denovo8419\_f0 | denovo8497\_f0 | denovo8549\_f0 | denovo8575\_f0 | denovo8627\_f0 | denovo8653\_f0 | denovo8705\_f0 | denovo8731\_f0 | denovo8757\_f0 | denovo8783\_f0 | denovo8913\_f0 | denovo8939\_f0 | denovo8965\_f0 | denovo8991\_f0 | denovo9121\_f0 | denovo9147\_f0 | denovo9251\_f0 | denovo9303\_f0 | denovo9329\_f0 | denovo9355\_f0 | denovo9459\_f0 | denovo9485\_f0 | denovo9511\_f0 | denovo9667\_f0 | denovo9719\_f0 | denovo9797\_f0 | denovo9823\_f0 | denovo9849\_f0 | denovo9901\_f0 | denovo9979\_f0 | denovo10031\_f0 | denovo10057\_f0 | denovo10213\_f0 | denovo10239\_f0 | denovo10291\_f0 | denovo10317\_f0 | denovo10447\_f0 | denovo10473\_f0 | denovo10525\_f0 | denovo10551\_f0 | denovo10577\_f0 | denovo10603\_f0 | denovo10681\_f0 | denovo10733\_f0 | denovo10759\_f0 | denovo10785\_f0 | denovo10837\_f0 | denovo11019\_f0 | denovo11045\_f0 | denovo11071\_f0 | denovo11201\_f0 | denovo11305\_f0 | denovo11357\_f0 | denovo11409\_f0 | denovo11435\_f0 | denovo11461\_f0 | denovo11487\_f0 | denovo11513\_f0 | denovo11539\_f0 | denovo11591\_f0 | denovo11617\_f0 | denovo11721\_f0 | denovo11747\_f0 | denovo11929\_f0 | denovo11955\_f0 | denovo12085\_f0 |
| FG120055B | Nothus lunus\* | denovo7\_f0 | denovo85\_f0 | denovo163\_f0 | denovo189\_f0 | denovo267\_f0 | denovo319\_f0 | denovo371\_f0 | denovo449\_f0 | denovo527\_f0 | denovo683\_f0 | denovo839\_f0 | denovo1047\_f0 | denovo1151\_f0 | denovo1229\_f0 | denovo1255\_f0 | denovo1359\_f0 | denovo1385\_f0 | denovo1411\_f0 | denovo1489\_f0 | denovo1645\_f0 | denovo1671\_f0 | denovo1723\_f0 | denovo1801\_f0 | denovo1853\_f0 | denovo1931\_f0 | denovo1957\_f0 | denovo1983\_f0 | denovo2009\_f0 | denovo2087\_f0 | denovo2113\_f0 | denovo2139\_f0 | denovo2243\_f0 | denovo2269\_f0 | denovo2295\_f0 | denovo2321\_f0 | denovo2373\_f0 | denovo2477\_f0 | denovo2529\_f0 | denovo2555\_f0 | denovo2581\_f0 | denovo2607\_f0 | denovo2633\_f0 | denovo2659\_f0 | denovo2763\_f0 | denovo2841\_f0 | denovo2893\_f0 | denovo3153\_f0 | denovo3257\_f0 | denovo3309\_f0 | denovo3335\_f0 | denovo3439\_f0 | denovo3491\_f0 | denovo3595\_f0 | denovo3621\_f0 | denovo3647\_f0 | denovo3725\_f0 | denovo3751\_f0 | denovo3803\_f0 | denovo3829\_f0 | denovo3985\_f0 | denovo4063\_f0 | denovo4141\_f0 | denovo4245\_f0 | denovo4323\_f0 | denovo4349\_f0 | denovo4375\_f0 | denovo4401\_f0 | denovo4427\_f0 | denovo4505\_f0 | denovo4531\_f0 | denovo4635\_f0 | denovo4765\_f0 | denovo4843\_f0 | denovo4895\_f0 | denovo4921\_f0 | denovo4947\_f0 | denovo4973\_f0 | denovo4999\_f0 | denovo5103\_f0 | denovo5155\_f0 | denovo5207\_f0 | denovo5415\_f0 | denovo5441\_f0 | denovo5545\_f0 | denovo5571\_f0 | denovo5909\_f0 | denovo5961\_f0 | denovo6013\_f0 | denovo6039\_f0 | denovo6065\_f0 | denovo6091\_f0 | denovo6195\_f0 | denovo6247\_f0 | denovo6273\_f0 | denovo6299\_f0 | denovo6377\_f0 | denovo6507\_f0 | denovo6559\_f0 | denovo6585\_f0 | denovo6611\_f0 | denovo6663\_f0 | denovo6689\_f0 | denovo6715\_f0 | denovo6741\_f0 | denovo6767\_f0 | denovo6897\_f0 | denovo6975\_f0 | denovo7001\_f0 | denovo7027\_f0 | denovo7079\_f0 | denovo7131\_f0 | denovo7157\_f0 | denovo7287\_f0 | denovo7313\_f0 | denovo7339\_f0 | denovo7365\_f0 | denovo7391\_f0 | denovo7469\_f0 | denovo7495\_f0 | denovo7521\_f0 | denovo7547\_f0 | denovo7625\_f0 | denovo7677\_f0 | denovo7703\_f0 | denovo7781\_f0 | denovo7833\_f0 | denovo7911\_f0 | denovo8015\_f0 | denovo8041\_f0 | denovo8223\_f0 | denovo8249\_f0 | denovo8275\_f0 | denovo8353\_f0 | denovo8379\_f0 | denovo8405\_f0 | denovo8483\_f0 | denovo8535\_f0 | denovo8561\_f0 | denovo8613\_f0 | denovo8639\_f0 | denovo8691\_f0 | denovo8717\_f0 | denovo8743\_f0 | denovo8769\_f0 | denovo8899\_f0 | denovo8925\_f0 | denovo8951\_f0 | denovo8977\_f0 | denovo9107\_f0 | denovo9133\_f0 | denovo9237\_f0 | denovo9289\_f0 | denovo9315\_f0 | denovo9341\_f0 | denovo9445\_f0 | denovo9471\_f0 | denovo9497\_f0 | denovo9653\_f0 | denovo9705\_f0 | denovo9783\_f0 | denovo9809\_f0 | denovo9835\_f0 | denovo9887\_f0 | denovo9965\_f0 | denovo10017\_f0 | denovo10043\_f0 | denovo10199\_f0 | denovo10225\_f0 | denovo10277\_f0 | denovo10303\_f0 | denovo10433\_f0 | denovo10459\_f0 | denovo10511\_f0 | denovo10537\_f0 | denovo10563\_f0 | denovo10589\_f0 | denovo10667\_f0 | denovo10719\_f0 | denovo10745\_f0 | denovo10771\_f0 | denovo10823\_f0 | denovo11005\_f0 | denovo11031\_f0 | denovo11057\_f0 | denovo11187\_f0 | denovo11291\_f0 | denovo11343\_f0 | denovo11395\_f0 | denovo11421\_f0 | denovo11447\_f0 | denovo11473\_f0 | denovo11499\_f0 | denovo11525\_f0 | denovo11577\_f0 | denovo11603\_f0 | denovo11707\_f0 | denovo11733\_f0 | denovo11915\_f0 | denovo11941\_f0 | denovo12071\_f0 |
| FG120122 | Macrosoma sp.\* | denovo12\_f0 | denovo90\_f0 | denovo168\_f0 | denovo194\_f0 | denovo272\_f0 | denovo324\_f0 | denovo376\_f0 | denovo454\_f0 | denovo532\_f0 | denovo688\_f0 | denovo844\_f0 | denovo1052\_f0 | denovo1156\_f0 | denovo1234\_f0 | denovo1260\_f0 | denovo1364\_f0 | denovo1390\_f0 | denovo1416\_f0 | denovo1494\_f0 | denovo1650\_f0 | denovo1676\_f0 | denovo1728\_f0 | denovo1806\_f0 | denovo1858\_f0 | denovo1936\_f0 | denovo1962\_f0 | denovo1988\_f0 | denovo2014\_f0 | denovo2092\_f0 | denovo2118\_f0 | denovo2144\_f0 | denovo2248\_f0 | denovo2274\_f0 | denovo2300\_f0 | denovo2326\_f0 | denovo2378\_f0 | denovo2482\_f0 | denovo2534\_f0 | denovo2560\_f0 | denovo2586\_f0 | denovo2612\_f0 | denovo2638\_f0 | denovo2664\_f0 | denovo2768\_f0 | denovo2846\_f0 | denovo2898\_f0 | denovo3158\_f0 | denovo3262\_f0 | denovo3314\_f0 | denovo3340\_f0 | denovo3444\_f0 | denovo3496\_f0 | denovo3600\_f0 | denovo3626\_f0 | denovo3652\_f0 | denovo3730\_f0 | denovo3756\_f0 | denovo3808\_f0 | denovo3834\_f0 | denovo3990\_f0 | denovo4068\_f0 | denovo4146\_f0 | denovo4250\_f0 | denovo4328\_f0 | denovo4354\_f0 | denovo4380\_f0 | denovo4406\_f0 | denovo4432\_f0 | denovo4510\_f0 | denovo4536\_f0 | denovo4640\_f0 | denovo4770\_f0 | denovo4848\_f0 | denovo4900\_f0 | denovo4926\_f0 | denovo4952\_f0 | denovo4978\_f0 | denovo5004\_f0 | denovo5108\_f0 | denovo5160\_f0 | denovo5212\_f0 | denovo5420\_f0 | denovo5446\_f0 | denovo5550\_f0 | denovo5576\_f0 | denovo5914\_f0 | denovo5966\_f0 | denovo6018\_f0 | denovo6044\_f0 | denovo6070\_f0 | denovo6096\_f0 | denovo6200\_f0 | denovo6252\_f0 | denovo6278\_f0 | denovo6304\_f0 | denovo6382\_f0 | denovo6512\_f0 | denovo6564\_f0 | denovo6590\_f0 | denovo6616\_f0 | denovo6668\_f0 | denovo6694\_f0 | denovo6720\_f0 | denovo6746\_f0 | denovo6772\_f0 | denovo6902\_f0 | denovo6980\_f0 | denovo7006\_f0 | denovo7032\_f0 | denovo7084\_f0 | denovo7136\_f0 | denovo7162\_f0 | denovo7292\_f0 | denovo7318\_f0 | denovo7344\_f0 | denovo7370\_f0 | denovo7396\_f0 | denovo7474\_f0 | denovo7500\_f0 | denovo7526\_f0 | denovo7552\_f0 | denovo7630\_f0 | denovo7682\_f0 | denovo7708\_f0 | denovo7786\_f0 | denovo7838\_f0 | denovo7916\_f0 | denovo8020\_f0 | denovo8046\_f0 | denovo8228\_f0 | denovo8254\_f0 | denovo8280\_f0 | denovo8358\_f0 | denovo8384\_f0 | denovo8410\_f0 | denovo8488\_f0 | denovo8540\_f0 | denovo8566\_f0 | denovo8618\_f0 | denovo8644\_f0 | denovo8696\_f0 | denovo8722\_f0 | denovo8748\_f0 | denovo8774\_f0 | denovo8904\_f0 | denovo8930\_f0 | denovo8956\_f0 | denovo8982\_f0 | denovo9112\_f0 | denovo9138\_f0 | denovo9242\_f0 | denovo9294\_f0 | denovo9320\_f0 | denovo9346\_f0 | denovo9450\_f0 | denovo9476\_f0 | denovo9502\_f0 | denovo9658\_f0 | denovo9710\_f0 | denovo9788\_f0 | denovo9814\_f0 | denovo9840\_f0 | denovo9892\_f0 | denovo9970\_f0 | denovo10022\_f0 | denovo10048\_f0 | denovo10204\_f0 | denovo10230\_f0 | denovo10282\_f0 | denovo10308\_f0 | denovo10438\_f0 | denovo10464\_f0 | denovo10516\_f0 | denovo10542\_f0 | denovo10568\_f0 | denovo10594\_f0 | denovo10672\_f0 | denovo10724\_f0 | denovo10750\_f0 | denovo10776\_f0 | denovo10828\_f0 | denovo11010\_f0 | denovo11036\_f0 | denovo11062\_f0 | denovo11192\_f0 | denovo11296\_f0 | denovo11348\_f0 | denovo11400\_f0 | denovo11426\_f0 | denovo11452\_f0 | denovo11478\_f0 | denovo11504\_f0 | denovo11530\_f0 | denovo11582\_f0 | denovo11608\_f0 | denovo11712\_f0 | denovo11738\_f0 | denovo11920\_f0 | denovo11946\_f0 | denovo12076\_f0 |
| FG120024 | Megalopyge tharops\* | denovo4\_f0 | denovo82\_f0 | denovo160\_f0 | denovo186\_f0 | denovo264\_f0 | denovo316\_f0 | denovo368\_f0 | denovo446\_f0 | denovo524\_f0 | denovo680\_f0 | denovo836\_f0 | denovo1044\_f0 | denovo1148\_f0 | denovo1226\_f0 | denovo1252\_f0 | denovo1356\_f0 | denovo1382\_f0 | denovo1408\_f0 | denovo1486\_f0 | denovo1642\_f0 | denovo1668\_f0 | denovo1720\_f0 | denovo1798\_f0 | denovo1850\_f0 | denovo1928\_f0 | denovo1954\_f0 | denovo1980\_f0 | denovo2006\_f0 | denovo2084\_f0 | denovo2110\_f0 | denovo2136\_f0 | denovo2240\_f0 | denovo2266\_f0 | denovo2292\_f0 | denovo2318\_f0 | denovo2370\_f0 | denovo2474\_f0 | denovo2526\_f0 | denovo2552\_f0 | denovo2578\_f0 | denovo2604\_f0 | denovo2630\_f0 | denovo2656\_f0 | denovo2760\_f0 | denovo2838\_f0 | denovo2890\_f0 | denovo3150\_f0 | denovo3254\_f0 | denovo3306\_f0 | denovo3332\_f0 | denovo3436\_f0 | denovo3488\_f0 | denovo3592\_f0 | denovo3618\_f0 | denovo3644\_f0 | denovo3722\_f0 | denovo3748\_f0 | denovo3800\_f0 | denovo3826\_f0 | denovo3982\_f0 | denovo4060\_f0 | denovo4138\_f0 | denovo4242\_f0 | denovo4320\_f0 | denovo4346\_f0 | denovo4372\_f0 | denovo4398\_f0 | denovo4424\_f0 | denovo4502\_f0 | denovo4528\_f0 | denovo4632\_f0 | denovo4762\_f0 | denovo4840\_f0 | denovo4892\_f0 | denovo4918\_f0 | denovo4944\_f0 | denovo4970\_f0 | denovo4996\_f0 | denovo5100\_f0 | denovo5152\_f0 | denovo5204\_f0 | denovo5412\_f0 | denovo5438\_f0 | denovo5542\_f0 | denovo5568\_f0 | denovo5906\_f0 | denovo5958\_f0 | denovo6010\_f0 | denovo6036\_f0 | denovo6062\_f0 | denovo6088\_f0 | denovo6192\_f0 | denovo6244\_f0 | denovo6270\_f0 | denovo6296\_f0 | denovo6374\_f0 | denovo6504\_f0 | denovo6556\_f0 | denovo6582\_f0 | denovo6608\_f0 | denovo6660\_f0 | denovo6686\_f0 | denovo6712\_f0 | denovo6738\_f0 | denovo6764\_f0 | denovo6894\_f0 | denovo6972\_f0 | denovo6998\_f0 | denovo7024\_f0 | denovo7076\_f0 | denovo7128\_f0 | denovo7154\_f0 | denovo7284\_f0 | denovo7310\_f0 | denovo7336\_f0 | denovo7362\_f0 | denovo7388\_f0 | denovo7466\_f0 | denovo7492\_f0 | denovo7518\_f0 | denovo7544\_f0 | denovo7622\_f0 | denovo7674\_f0 | denovo7700\_f0 | denovo7778\_f0 | denovo7830\_f0 | denovo7908\_f0 | denovo8012\_f0 | denovo8038\_f0 | denovo8220\_f0 | denovo8246\_f0 | denovo8272\_f0 | denovo8350\_f0 | denovo8376\_f0 | denovo8402\_f0 | denovo8480\_f0 | denovo8532\_f0 | denovo8558\_f0 | denovo8610\_f0 | denovo8636\_f0 | denovo8688\_f0 | denovo8714\_f0 | denovo8740\_f0 | denovo8766\_f0 | denovo8896\_f0 | denovo8922\_f0 | denovo8948\_f0 | denovo8974\_f0 | denovo9104\_f0 | denovo9130\_f0 | denovo9234\_f0 | denovo9286\_f0 | denovo9312\_f0 | denovo9338\_f0 | denovo9442\_f0 | denovo9468\_f0 | denovo9494\_f0 | denovo9650\_f0 | denovo9702\_f0 | denovo9780\_f0 | denovo9806\_f0 | denovo9832\_f0 | denovo9884\_f0 | denovo9962\_f0 | denovo10014\_f0 | denovo10040\_f0 | denovo10196\_f0 | denovo10222\_f0 | denovo10274\_f0 | denovo10300\_f0 | denovo10430\_f0 | denovo10456\_f0 | denovo10508\_f0 | denovo10534\_f0 | denovo10560\_f0 | denovo10586\_f0 | denovo10664\_f0 | denovo10716\_f0 | denovo10742\_f0 | denovo10768\_f0 | denovo10820\_f0 | denovo11002\_f0 | denovo11028\_f0 | denovo11054\_f0 | denovo11184\_f0 | denovo11288\_f0 | denovo11340\_f0 | denovo11392\_f0 | denovo11418\_f0 | denovo11444\_f0 | denovo11470\_f0 | denovo11496\_f0 | denovo11522\_f0 | denovo11574\_f0 | denovo11600\_f0 | denovo11704\_f0 | denovo11730\_f0 | denovo11912\_f0 | denovo11938\_f0 | denovo12068\_f0 |
| FG120022 | Morpheis mathani\* | denovo3\_f0 | denovo81\_f0 | denovo159\_f0 | denovo185\_f0 | denovo263\_f0 | denovo315\_f0 | denovo367\_f0 | denovo445\_f0 | denovo523\_f0 | denovo679\_f0 | denovo835\_f0 | denovo1043\_f0 | denovo1147\_f0 | denovo1225\_f0 | denovo1251\_f0 | denovo1355\_f0 | denovo1381\_f0 | denovo1407\_f0 | denovo1485\_f0 | denovo1641\_f0 | denovo1667\_f0 | denovo1719\_f0 | denovo1797\_f0 | denovo1849\_f0 | denovo1927\_f0 | denovo1953\_f0 | denovo1979\_f0 | denovo2005\_f0 | denovo2083\_f0 | denovo2109\_f0 | denovo2135\_f0 | denovo2239\_f0 | denovo2265\_f0 | denovo2291\_f0 | denovo2317\_f0 | denovo2369\_f0 | denovo2473\_f0 | denovo2525\_f0 | denovo2551\_f0 | denovo2577\_f0 | denovo2603\_f0 | denovo2629\_f0 | denovo2655\_f0 | denovo2759\_f0 | denovo2837\_f0 | denovo2889\_f0 | denovo3149\_f0 | denovo3253\_f0 | denovo3305\_f0 | denovo3331\_f0 | denovo3435\_f0 | denovo3487\_f0 | denovo3591\_f0 | denovo3617\_f0 | denovo3643\_f0 | denovo3721\_f0 | denovo3747\_f0 | denovo3799\_f0 | denovo3825\_f0 | denovo3981\_f0 | denovo4059\_f0 | denovo4137\_f0 | denovo4241\_f0 | denovo4319\_f0 | denovo4345\_f0 | denovo4371\_f0 | denovo4397\_f0 | denovo4423\_f0 | denovo4501\_f0 | denovo4527\_f0 | denovo4631\_f0 | denovo4761\_f0 | denovo4839\_f0 | denovo4891\_f0 | denovo4917\_f0 | denovo4943\_f0 | denovo4969\_f0 | denovo4995\_f0 | denovo5099\_f0 | denovo5151\_f0 | denovo5203\_f0 | denovo5411\_f0 | denovo5437\_f0 | denovo5541\_f0 | denovo5567\_f0 | denovo5905\_f0 | denovo5957\_f0 | denovo6009\_f0 | denovo6035\_f0 | denovo6061\_f0 | denovo6087\_f0 | denovo6191\_f0 | denovo6243\_f0 | denovo6269\_f0 | denovo6295\_f0 | denovo6373\_f0 | denovo6503\_f0 | denovo6555\_f0 | denovo6581\_f0 | denovo6607\_f0 | denovo6659\_f0 | denovo6685\_f0 | denovo6711\_f0 | denovo6737\_f0 | denovo6763\_f0 | denovo6893\_f0 | denovo6971\_f0 | denovo6997\_f0 | denovo7023\_f0 | denovo7075\_f0 | denovo7127\_f0 | denovo7153\_f0 | denovo7283\_f0 | denovo7309\_f0 | denovo7335\_f0 | denovo7361\_f0 | denovo7387\_f0 | denovo7465\_f0 | denovo7491\_f0 | denovo7517\_f0 | denovo7543\_f0 | denovo7621\_f0 | denovo7673\_f0 | denovo7699\_f0 | denovo7777\_f0 | denovo7829\_f0 | denovo7907\_f0 | denovo8011\_f0 | denovo8037\_f0 | denovo8219\_f0 | denovo8245\_f0 | denovo8271\_f0 | denovo8349\_f0 | denovo8375\_f0 | denovo8401\_f0 | denovo8479\_f0 | denovo8531\_f0 | denovo8557\_f0 | denovo8609\_f0 | denovo8635\_f0 | denovo8687\_f0 | denovo8713\_f0 | denovo8739\_f0 | denovo8765\_f0 | denovo8895\_f0 | denovo8921\_f0 | denovo8947\_f0 | denovo8973\_f0 | denovo9103\_f0 | denovo9129\_f0 | denovo9233\_f0 | denovo9285\_f0 | denovo9311\_f0 | denovo9337\_f0 | denovo9441\_f0 | denovo9467\_f0 | denovo9493\_f0 | denovo9649\_f0 | denovo9701\_f0 | denovo9779\_f0 | denovo9805\_f0 | denovo9831\_f0 | denovo9883\_f0 | denovo9961\_f0 | denovo10013\_f0 | denovo10039\_f0 | denovo10195\_f0 | denovo10221\_f0 | denovo10273\_f0 | denovo10299\_f0 | denovo10429\_f0 | denovo10455\_f0 | denovo10507\_f0 | denovo10533\_f0 | denovo10559\_f0 | denovo10585\_f0 | denovo10663\_f0 | denovo10715\_f0 | denovo10741\_f0 | denovo10767\_f0 | denovo10819\_f0 | denovo11001\_f0 | denovo11027\_f0 | denovo11053\_f0 | denovo11183\_f0 | denovo11287\_f0 | denovo11339\_f0 | denovo11391\_f0 | denovo11417\_f0 | denovo11443\_f0 | denovo11469\_f0 | denovo11495\_f0 | denovo11521\_f0 | denovo11573\_f0 | denovo11599\_f0 | denovo11703\_f0 | denovo11729\_f0 | denovo11911\_f0 | denovo11937\_f0 | denovo12067\_f0 |
| GNV139000 | Megathymus yuccae\* | denovo16\_f0 | denovo94\_f0 | denovo172\_f0 | denovo198\_f0 | denovo276\_f0 | denovo328\_f0 | denovo380\_f0 | denovo458\_f0 | denovo536\_f0 | denovo692\_f0 | denovo848\_f0 | denovo1056\_f0 | denovo1160\_f0 | denovo1238\_f0 | denovo1264\_f0 | denovo1368\_f0 | denovo1394\_f0 | denovo1420\_f0 | denovo1498\_f0 | denovo1654\_f0 | denovo1680\_f0 | denovo1732\_f0 | denovo1810\_f0 | denovo1862\_f0 | denovo1940\_f0 | denovo1966\_f0 | denovo1992\_f0 | denovo2018\_f0 | denovo2096\_f0 | denovo2122\_f0 | denovo2148\_f0 | denovo2252\_f0 | denovo2278\_f0 | denovo2304\_f0 | denovo2330\_f0 | denovo2382\_f0 | denovo2486\_f0 | denovo2538\_f0 | denovo2564\_f0 | denovo2590\_f0 | denovo2616\_f0 | denovo2642\_f0 | denovo2668\_f0 | denovo2772\_f0 | denovo2850\_f0 | denovo2902\_f0 | denovo3162\_f0 | denovo3266\_f0 | denovo3318\_f0 | denovo3344\_f0 | denovo3448\_f0 | denovo3500\_f0 | denovo3604\_f0 | denovo3630\_f0 | denovo3656\_f0 | denovo3734\_f0 | denovo3760\_f0 | denovo3812\_f0 | denovo3838\_f0 | denovo3994\_f0 | denovo4072\_f0 | denovo4150\_f0 | denovo4254\_f0 | denovo4332\_f0 | denovo4358\_f0 | denovo4384\_f0 | denovo4410\_f0 | denovo4436\_f0 | denovo4514\_f0 | denovo4540\_f0 | denovo4644\_f0 | denovo4774\_f0 | denovo4852\_f0 | denovo4904\_f0 | denovo4930\_f0 | denovo4956\_f0 | denovo4982\_f0 | denovo5008\_f0 | denovo5112\_f0 | denovo5164\_f0 | denovo5216\_f0 | denovo5424\_f0 | denovo5450\_f0 | denovo5554\_f0 | denovo5580\_f0 | denovo5918\_f0 | denovo5970\_f0 | denovo6022\_f0 | denovo6048\_f0 | denovo6074\_f0 | denovo6100\_f0 | denovo6204\_f0 | denovo6256\_f0 | denovo6282\_f0 | denovo6308\_f0 | denovo6386\_f0 | denovo6516\_f0 | denovo6568\_f0 | denovo6594\_f0 | denovo6620\_f0 | denovo6672\_f0 | denovo6698\_f0 | denovo6724\_f0 | denovo6750\_f0 | denovo6776\_f0 | denovo6906\_f0 | denovo6984\_f0 | denovo7010\_f0 | denovo7036\_f0 | denovo7088\_f0 | denovo7140\_f0 | denovo7166\_f0 | denovo7296\_f0 | denovo7322\_f0 | denovo7348\_f0 | denovo7374\_f0 | denovo7400\_f0 | denovo7478\_f0 | denovo7504\_f0 | denovo7530\_f0 | denovo7556\_f0 | denovo7634\_f0 | denovo7686\_f0 | denovo7712\_f0 | denovo7790\_f0 | denovo7842\_f0 | denovo7920\_f0 | denovo8024\_f0 | denovo8050\_f0 | denovo8232\_f0 | denovo8258\_f0 | denovo8284\_f0 | denovo8362\_f0 | denovo8388\_f0 | denovo8414\_f0 | denovo8492\_f0 | denovo8544\_f0 | denovo8570\_f0 | denovo8622\_f0 | denovo8648\_f0 | denovo8700\_f0 | denovo8726\_f0 | denovo8752\_f0 | denovo8778\_f0 | denovo8908\_f0 | denovo8934\_f0 | denovo8960\_f0 | denovo8986\_f0 | denovo9116\_f0 | denovo9142\_f0 | denovo9246\_f0 | denovo9298\_f0 | denovo9324\_f0 | denovo9350\_f0 | denovo9454\_f0 | denovo9480\_f0 | denovo9506\_f0 | denovo9662\_f0 | denovo9714\_f0 | denovo9792\_f0 | denovo9818\_f0 | denovo9844\_f0 | denovo9896\_f0 | denovo9974\_f0 | denovo10026\_f0 | denovo10052\_f0 | denovo10208\_f0 | denovo10234\_f0 | denovo10286\_f0 | denovo10312\_f0 | denovo10442\_f0 | denovo10468\_f0 | denovo10520\_f0 | denovo10546\_f0 | denovo10572\_f0 | denovo10598\_f0 | denovo10676\_f0 | denovo10728\_f0 | denovo10754\_f0 | denovo10780\_f0 | denovo10832\_f0 | denovo11014\_f0 | denovo11040\_f0 | denovo11066\_f0 | denovo11196\_f0 | denovo11300\_f0 | denovo11352\_f0 | denovo11404\_f0 | denovo11430\_f0 | denovo11456\_f0 | denovo11482\_f0 | denovo11508\_f0 | denovo11534\_f0 | denovo11586\_f0 | denovo11612\_f0 | denovo11716\_f0 | denovo11742\_f0 | denovo11924\_f0 | denovo11950\_f0 | denovo12080\_f0 |
| Msexta | Manduca sexta\* | denovo17\_f0 | denovo95\_f0 | denovo173\_f0 | denovo199\_f0 | denovo277\_f0 | denovo329\_f0 | denovo381\_f0 | denovo459\_f0 | denovo537\_f0 | denovo693\_f0 | denovo849\_f0 | denovo1057\_f0 | denovo1161\_f0 | denovo1239\_f0 | denovo1265\_f0 | denovo1369\_f0 | denovo1395\_f0 | denovo1421\_f0 | denovo1499\_f0 | denovo1655\_f0 | denovo1681\_f0 | denovo1733\_f0 | denovo1811\_f0 | denovo1863\_f0 | denovo1941\_f0 | denovo1967\_f0 | denovo1993\_f0 | denovo2019\_f0 | denovo2097\_f0 | denovo2123\_f0 | denovo2149\_f0 | denovo2253\_f0 | denovo2279\_f0 | denovo2305\_f0 | denovo2331\_f0 | denovo2383\_f0 | denovo2487\_f0 | denovo2539\_f0 | denovo2565\_f0 | denovo2591\_f0 | denovo2617\_f0 | denovo2643\_f0 | denovo2669\_f0 | denovo2773\_f0 | denovo2851\_f0 | denovo2903\_f0 | denovo3163\_f0 | denovo3267\_f0 | denovo3319\_f0 | denovo3345\_f0 | denovo3449\_f0 | denovo3501\_f0 | denovo3605\_f0 | denovo3631\_f0 | denovo3657\_f0 | denovo3735\_f0 | denovo3761\_f0 | denovo3813\_f0 | denovo3839\_f0 | denovo3995\_f0 | denovo4073\_f0 | denovo4151\_f0 | denovo4255\_f0 | denovo4333\_f0 | denovo4359\_f0 | denovo4385\_f0 | denovo4411\_f0 | denovo4437\_f0 | denovo4515\_f0 | denovo4541\_f0 | denovo4645\_f0 | denovo4775\_f0 | denovo4853\_f0 | denovo4905\_f0 | denovo4931\_f0 | denovo4957\_f0 | denovo4983\_f0 | denovo5009\_f0 | denovo5113\_f0 | denovo5165\_f0 | denovo5217\_f0 | denovo5425\_f0 | denovo5451\_f0 | denovo5555\_f0 | denovo5581\_f0 | denovo5919\_f0 | denovo5971\_f0 | denovo6023\_f0 | denovo6049\_f0 | denovo6075\_f0 | denovo6101\_f0 | denovo6205\_f0 | denovo6257\_f0 | denovo6283\_f0 | denovo6309\_f0 | denovo6387\_f0 | denovo6517\_f0 | denovo6569\_f0 | denovo6595\_f0 | denovo6621\_f0 | denovo6673\_f0 | denovo6699\_f0 | denovo6725\_f0 | denovo6751\_f0 | denovo6777\_f0 | denovo6907\_f0 | denovo6985\_f0 | denovo7011\_f0 | denovo7037\_f0 | denovo7089\_f0 | denovo7141\_f0 | denovo7167\_f0 | denovo7297\_f0 | denovo7323\_f0 | denovo7349\_f0 | denovo7375\_f0 | denovo7401\_f0 | denovo7479\_f0 | denovo7505\_f0 | denovo7531\_f0 | denovo7557\_f0 | denovo7635\_f0 | denovo7687\_f0 | denovo7713\_f0 | denovo7791\_f0 | denovo7843\_f0 | denovo7921\_f0 | denovo8025\_f0 | denovo8051\_f0 | denovo8233\_f0 | denovo8259\_f0 | denovo8285\_f0 | denovo8363\_f0 | denovo8389\_f0 | denovo8415\_f0 | denovo8493\_f0 | denovo8545\_f0 | denovo8571\_f0 | denovo8623\_f0 | denovo8649\_f0 | denovo8701\_f0 | denovo8727\_f0 | denovo8753\_f0 | denovo8779\_f0 | denovo8909\_f0 | denovo8935\_f0 | denovo8961\_f0 | denovo8987\_f0 | denovo9117\_f0 | denovo9143\_f0 | denovo9247\_f0 | denovo9299\_f0 | denovo9325\_f0 | denovo9351\_f0 | denovo9455\_f0 | denovo9481\_f0 | denovo9507\_f0 | denovo9663\_f0 | denovo9715\_f0 | denovo9793\_f0 | denovo9819\_f0 | denovo9845\_f0 | denovo9897\_f0 | denovo9975\_f0 | denovo10027\_f0 | denovo10053\_f0 | denovo10209\_f0 | denovo10235\_f0 | denovo10287\_f0 | denovo10313\_f0 | denovo10443\_f0 | denovo10469\_f0 | denovo10521\_f0 | denovo10547\_f0 | denovo10573\_f0 | denovo10599\_f0 | denovo10677\_f0 | denovo10729\_f0 | denovo10755\_f0 | denovo10781\_f0 | denovo10833\_f0 | denovo11015\_f0 | denovo11041\_f0 | denovo11067\_f0 | denovo11197\_f0 | denovo11301\_f0 | denovo11353\_f0 | denovo11405\_f0 | denovo11431\_f0 | denovo11457\_f0 | denovo11483\_f0 | denovo11509\_f0 | denovo11535\_f0 | denovo11587\_f0 | denovo11613\_f0 | denovo11717\_f0 | denovo11743\_f0 | denovo11925\_f0 | denovo11951\_f0 | denovo12081\_f0 |
| acti2 | Actias luna\* | denovo25\_f0 | denovo103\_f0 | denovo181\_f0 | denovo207\_f0 | denovo285\_f0 | denovo337\_f0 | denovo389\_f0 | denovo467\_f0 | denovo545\_f0 | denovo701\_f0 | denovo857\_f0 | denovo1065\_f0 | denovo1169\_f0 | denovo1247\_f0 | denovo1273\_f0 | denovo1377\_f0 | denovo1403\_f0 | denovo1429\_f0 | denovo1507\_f0 | denovo1663\_f0 | denovo1689\_f0 | denovo1741\_f0 | denovo1819\_f0 | denovo1871\_f0 | denovo1949\_f0 | denovo1975\_f0 | denovo2001\_f0 | denovo2027\_f0 | denovo2105\_f0 | denovo2131\_f0 | denovo2157\_f0 | denovo2261\_f0 | denovo2287\_f0 | denovo2313\_f0 | denovo2339\_f0 | denovo2391\_f0 | denovo2495\_f0 | denovo2547\_f0 | denovo2573\_f0 | denovo2599\_f0 | denovo2625\_f0 | denovo2651\_f0 | denovo2677\_f0 | denovo2781\_f0 | denovo2859\_f0 | denovo2911\_f0 | denovo3171\_f0 | denovo3275\_f0 | denovo3327\_f0 | denovo3353\_f0 | denovo3457\_f0 | denovo3509\_f0 | denovo3613\_f0 | denovo3639\_f0 | denovo3665\_f0 | denovo3743\_f0 | denovo3769\_f0 | denovo3821\_f0 | denovo3847\_f0 | denovo4003\_f0 | denovo4081\_f0 | denovo4159\_f0 | denovo4263\_f0 | denovo4341\_f0 | denovo4367\_f0 | denovo4393\_f0 | denovo4419\_f0 | denovo4445\_f0 | denovo4523\_f0 | denovo4549\_f0 | denovo4653\_f0 | denovo4783\_f0 | denovo4861\_f0 | denovo4913\_f0 | denovo4939\_f0 | denovo4965\_f0 | denovo4991\_f0 | denovo5017\_f0 | denovo5121\_f0 | denovo5173\_f0 | denovo5225\_f0 | denovo5433\_f0 | denovo5459\_f0 | denovo5563\_f0 | denovo5589\_f0 | denovo5927\_f0 | denovo5979\_f0 | denovo6031\_f0 | denovo6057\_f0 | denovo6083\_f0 | denovo6109\_f0 | denovo6213\_f0 | denovo6265\_f0 | denovo6291\_f0 | denovo6317\_f0 | denovo6395\_f0 | denovo6525\_f0 | denovo6577\_f0 | denovo6603\_f0 | denovo6629\_f0 | denovo6681\_f0 | denovo6707\_f0 | denovo6733\_f0 | denovo6759\_f0 | denovo6785\_f0 | denovo6915\_f0 | denovo6993\_f0 | denovo7019\_f0 | denovo7045\_f0 | denovo7097\_f0 | denovo7149\_f0 | denovo7175\_f0 | denovo7305\_f0 | denovo7331\_f0 | denovo7357\_f0 | denovo7383\_f0 | denovo7409\_f0 | denovo7487\_f0 | denovo7513\_f0 | denovo7539\_f0 | denovo7565\_f0 | denovo7643\_f0 | denovo7695\_f0 | denovo7721\_f0 | denovo7799\_f0 | denovo7851\_f0 | denovo7929\_f0 | denovo8033\_f0 | denovo8059\_f0 | denovo8241\_f0 | denovo8267\_f0 | denovo8293\_f0 | denovo8371\_f0 | denovo8397\_f0 | denovo8423\_f0 | denovo8501\_f0 | denovo8553\_f0 | denovo8579\_f0 | denovo8631\_f0 | denovo8657\_f0 | denovo8709\_f0 | denovo8735\_f0 | denovo8761\_f0 | denovo8787\_f0 | denovo8917\_f0 | denovo8943\_f0 | denovo8969\_f0 | denovo8995\_f0 | denovo9125\_f0 | denovo9151\_f0 | denovo9255\_f0 | denovo9307\_f0 | denovo9333\_f0 | denovo9359\_f0 | denovo9463\_f0 | denovo9489\_f0 | denovo9515\_f0 | denovo9671\_f0 | denovo9723\_f0 | denovo9801\_f0 | denovo9827\_f0 | denovo9853\_f0 | denovo9905\_f0 | denovo9983\_f0 | denovo10035\_f0 | denovo10061\_f0 | denovo10217\_f0 | denovo10243\_f0 | denovo10295\_f0 | denovo10321\_f0 | denovo10451\_f0 | denovo10477\_f0 | denovo10529\_f0 | denovo10555\_f0 | denovo10581\_f0 | denovo10607\_f0 | denovo10685\_f0 | denovo10737\_f0 | denovo10763\_f0 | denovo10789\_f0 | denovo10841\_f0 | denovo11023\_f0 | denovo11049\_f0 | denovo11075\_f0 | denovo11205\_f0 | denovo11309\_f0 | denovo11361\_f0 | denovo11413\_f0 | denovo11439\_f0 | denovo11465\_f0 | denovo11491\_f0 | denovo11517\_f0 | denovo11543\_f0 | denovo11595\_f0 | denovo11621\_f0 | denovo11725\_f0 | denovo11751\_f0 | denovo11933\_f0 | denovo11959\_f0 | denovo12089\_f0 |
| FG120071B | Myelobia sp.\* | denovo9\_f0 | denovo87\_f0 | denovo165\_f0 | denovo191\_f0 | denovo269\_f0 | denovo321\_f0 | denovo373\_f0 | denovo451\_f0 | denovo529\_f0 | denovo685\_f0 | denovo841\_f0 | denovo1049\_f0 | denovo1153\_f0 | denovo1231\_f0 | denovo1257\_f0 | denovo1361\_f0 | denovo1387\_f0 | denovo1413\_f0 | denovo1491\_f0 | denovo1647\_f0 | denovo1673\_f0 | denovo1725\_f0 | denovo1803\_f0 | denovo1855\_f0 | denovo1933\_f0 | denovo1959\_f0 | denovo1985\_f0 | denovo2011\_f0 | denovo2089\_f0 | denovo2115\_f0 | denovo2141\_f0 | denovo2245\_f0 | denovo2271\_f0 | denovo2297\_f0 | denovo2323\_f0 | denovo2375\_f0 | denovo2479\_f0 | denovo2531\_f0 | denovo2557\_f0 | denovo2583\_f0 | denovo2609\_f0 | denovo2635\_f0 | denovo2661\_f0 | denovo2765\_f0 | denovo2843\_f0 | denovo2895\_f0 | denovo3155\_f0 | denovo3259\_f0 | denovo3311\_f0 | denovo3337\_f0 | denovo3441\_f0 | denovo3493\_f0 | denovo3597\_f0 | denovo3623\_f0 | denovo3649\_f0 | denovo3727\_f0 | denovo3753\_f0 | denovo3805\_f0 | denovo3831\_f0 | denovo3987\_f0 | denovo4065\_f0 | denovo4143\_f0 | denovo4247\_f0 | denovo4325\_f0 | denovo4351\_f0 | denovo4377\_f0 | denovo4403\_f0 | denovo4429\_f0 | denovo4507\_f0 | denovo4533\_f0 | denovo4637\_f0 | denovo4767\_f0 | denovo4845\_f0 | denovo4897\_f0 | denovo4923\_f0 | denovo4949\_f0 | denovo4975\_f0 | denovo5001\_f0 | denovo5105\_f0 | denovo5157\_f0 | denovo5209\_f0 | denovo5417\_f0 | denovo5443\_f0 | denovo5547\_f0 | denovo5573\_f0 | denovo5911\_f0 | denovo5963\_f0 | denovo6015\_f0 | denovo6041\_f0 | denovo6067\_f0 | denovo6093\_f0 | denovo6197\_f0 | denovo6249\_f0 | denovo6275\_f0 | denovo6301\_f0 | denovo6379\_f0 | denovo6509\_f0 | denovo6561\_f0 | denovo6587\_f0 | denovo6613\_f0 | denovo6665\_f0 | denovo6691\_f0 | denovo6717\_f0 | denovo6743\_f0 | denovo6769\_f0 | denovo6899\_f0 | denovo6977\_f0 | denovo7003\_f0 | denovo7029\_f0 | denovo7081\_f0 | denovo7133\_f0 | denovo7159\_f0 | denovo7289\_f0 | denovo7315\_f0 | denovo7341\_f0 | denovo7367\_f0 | denovo7393\_f0 | denovo7471\_f0 | denovo7497\_f0 | denovo7523\_f0 | denovo7549\_f0 | denovo7627\_f0 | denovo7679\_f0 | denovo7705\_f0 | denovo7783\_f0 | denovo7835\_f0 | denovo7913\_f0 | denovo8017\_f0 | denovo8043\_f0 | denovo8225\_f0 | denovo8251\_f0 | denovo8277\_f0 | denovo8355\_f0 | denovo8381\_f0 | denovo8407\_f0 | denovo8485\_f0 | denovo8537\_f0 | denovo8563\_f0 | denovo8615\_f0 | denovo8641\_f0 | denovo8693\_f0 | denovo8719\_f0 | denovo8745\_f0 | denovo8771\_f0 | denovo8901\_f0 | denovo8927\_f0 | denovo8953\_f0 | denovo8979\_f0 | denovo9109\_f0 | denovo9135\_f0 | denovo9239\_f0 | denovo9291\_f0 | denovo9317\_f0 | denovo9343\_f0 | denovo9447\_f0 | denovo9473\_f0 | denovo9499\_f0 | denovo9655\_f0 | denovo9707\_f0 | denovo9785\_f0 | denovo9811\_f0 | denovo9837\_f0 | denovo9889\_f0 | denovo9967\_f0 | denovo10019\_f0 | denovo10045\_f0 | denovo10201\_f0 | denovo10227\_f0 | denovo10279\_f0 | denovo10305\_f0 | denovo10435\_f0 | denovo10461\_f0 | denovo10513\_f0 | denovo10539\_f0 | denovo10565\_f0 | denovo10591\_f0 | denovo10669\_f0 | denovo10721\_f0 | denovo10747\_f0 | denovo10773\_f0 | denovo10825\_f0 | denovo11007\_f0 | denovo11033\_f0 | denovo11059\_f0 | denovo11189\_f0 | denovo11293\_f0 | denovo11345\_f0 | denovo11397\_f0 | denovo11423\_f0 | denovo11449\_f0 | denovo11475\_f0 | denovo11501\_f0 | denovo11527\_f0 | denovo11579\_f0 | denovo11605\_f0 | denovo11709\_f0 | denovo11735\_f0 | denovo11917\_f0 | denovo11943\_f0 | denovo12073\_f0 |
| GNV120027 | Lantanophaga pusillidactyla\* | denovo13\_f0 | denovo91\_f0 | denovo169\_f0 | denovo195\_f0 | denovo273\_f0 | denovo325\_f0 | denovo377\_f0 | denovo455\_f0 | denovo533\_f0 | denovo689\_f0 | denovo845\_f0 | denovo1053\_f0 | denovo1157\_f0 | denovo1235\_f0 | denovo1261\_f0 | denovo1365\_f0 | denovo1391\_f0 | denovo1417\_f0 | denovo1495\_f0 | denovo1651\_f0 | denovo1677\_f0 | denovo1729\_f0 | denovo1807\_f0 | denovo1859\_f0 | denovo1937\_f0 | denovo1963\_f0 | denovo1989\_f0 | denovo2015\_f0 | denovo2093\_f0 | denovo2119\_f0 | denovo2145\_f0 | denovo2249\_f0 | denovo2275\_f0 | denovo2301\_f0 | denovo2327\_f0 | denovo2379\_f0 | denovo2483\_f0 | denovo2535\_f0 | denovo2561\_f0 | denovo2587\_f0 | denovo2613\_f0 | denovo2639\_f0 | denovo2665\_f0 | denovo2769\_f0 | denovo2847\_f0 | denovo2899\_f0 | denovo3159\_f0 | denovo3263\_f0 | denovo3315\_f0 | denovo3341\_f0 | denovo3445\_f0 | denovo3497\_f0 | denovo3601\_f0 | denovo3627\_f0 | denovo3653\_f0 | denovo3731\_f0 | denovo3757\_f0 | denovo3809\_f0 | denovo3835\_f0 | denovo3991\_f0 | denovo4069\_f0 | denovo4147\_f0 | denovo4251\_f0 | denovo4329\_f0 | denovo4355\_f0 | denovo4381\_f0 | denovo4407\_f0 | denovo4433\_f0 | denovo4511\_f0 | denovo4537\_f0 | denovo4641\_f0 | denovo4771\_f0 | denovo4849\_f0 | denovo4901\_f0 | denovo4927\_f0 | denovo4953\_f0 | denovo4979\_f0 | denovo5005\_f0 | denovo5109\_f0 | denovo5161\_f0 | denovo5213\_f0 | denovo5421\_f0 | denovo5447\_f0 | denovo5551\_f0 | denovo5577\_f0 | denovo5915\_f0 | denovo5967\_f0 | denovo6019\_f0 | denovo6045\_f0 | denovo6071\_f0 | denovo6097\_f0 | denovo6201\_f0 | denovo6253\_f0 | denovo6279\_f0 | denovo6305\_f0 | denovo6383\_f0 | denovo6513\_f0 | denovo6565\_f0 | denovo6591\_f0 | denovo6617\_f0 | denovo6669\_f0 | denovo6695\_f0 | denovo6721\_f0 | denovo6747\_f0 | denovo6773\_f0 | denovo6903\_f0 | denovo6981\_f0 | denovo7007\_f0 | denovo7033\_f0 | denovo7085\_f0 | denovo7137\_f0 | denovo7163\_f0 | denovo7293\_f0 | denovo7319\_f0 | denovo7345\_f0 | denovo7371\_f0 | denovo7397\_f0 | denovo7475\_f0 | denovo7501\_f0 | denovo7527\_f0 | denovo7553\_f0 | denovo7631\_f0 | denovo7683\_f0 | denovo7709\_f0 | denovo7787\_f0 | denovo7839\_f0 | denovo7917\_f0 | denovo8021\_f0 | denovo8047\_f0 | denovo8229\_f0 | denovo8255\_f0 | denovo8281\_f0 | denovo8359\_f0 | denovo8385\_f0 | denovo8411\_f0 | denovo8489\_f0 | denovo8541\_f0 | denovo8567\_f0 | denovo8619\_f0 | denovo8645\_f0 | denovo8697\_f0 | denovo8723\_f0 | denovo8749\_f0 | denovo8775\_f0 | denovo8905\_f0 | denovo8931\_f0 | denovo8957\_f0 | denovo8983\_f0 | denovo9113\_f0 | denovo9139\_f0 | denovo9243\_f0 | denovo9295\_f0 | denovo9321\_f0 | denovo9347\_f0 | denovo9451\_f0 | denovo9477\_f0 | denovo9503\_f0 | denovo9659\_f0 | denovo9711\_f0 | denovo9789\_f0 | denovo9815\_f0 | denovo9841\_f0 | denovo9893\_f0 | denovo9971\_f0 | denovo10023\_f0 | denovo10049\_f0 | denovo10205\_f0 | denovo10231\_f0 | denovo10283\_f0 | denovo10309\_f0 | denovo10439\_f0 | denovo10465\_f0 | denovo10517\_f0 | denovo10543\_f0 | denovo10569\_f0 | denovo10595\_f0 | denovo10673\_f0 | denovo10725\_f0 | denovo10751\_f0 | denovo10777\_f0 | denovo10829\_f0 | denovo11011\_f0 | denovo11037\_f0 | denovo11063\_f0 | denovo11193\_f0 | denovo11297\_f0 | denovo11349\_f0 | denovo11401\_f0 | denovo11427\_f0 | denovo11453\_f0 | denovo11479\_f0 | denovo11505\_f0 | denovo11531\_f0 | denovo11583\_f0 | denovo11609\_f0 | denovo11713\_f0 | denovo11739\_f0 | denovo11921\_f0 | denovo11947\_f0 | denovo12077\_f0 |
| Bmoricds | Bombyx mori\* | denovo0\_f0 | denovo78\_f0 | denovo156\_f0 | denovo182\_f0 | denovo260\_f0 | denovo312\_f0 | denovo364\_f0 | denovo442\_f0 | denovo520\_f0 | denovo676\_f0 | denovo832\_f0 | denovo1040\_f0 | denovo1144\_f0 | denovo1222\_f0 | denovo1248\_f0 | denovo1352\_f0 | denovo1378\_f0 | denovo1404\_f0 | denovo1482\_f0 | denovo1638\_f0 | denovo1664\_f0 | denovo1716\_f0 | denovo1794\_f0 | denovo1846\_f0 | denovo1924\_f0 | denovo1950\_f0 | denovo1976\_f0 | denovo2002\_f0 | denovo2080\_f0 | denovo2106\_f0 | denovo2132\_f0 | denovo2236\_f0 | denovo2262\_f0 | denovo2288\_f0 | denovo2314\_f0 | denovo2366\_f0 | denovo2470\_f0 | denovo2522\_f0 | denovo2548\_f0 | denovo2574\_f0 | denovo2600\_f0 | denovo2626\_f0 | denovo2652\_f0 | denovo2756\_f0 | denovo2834\_f0 | denovo2886\_f0 | denovo3146\_f0 | denovo3250\_f0 | denovo3302\_f0 | denovo3328\_f0 | denovo3432\_f0 | denovo3484\_f0 | denovo3588\_f0 | denovo3614\_f0 | denovo3640\_f0 | denovo3718\_f0 | denovo3744\_f0 | denovo3796\_f0 | denovo3822\_f0 | denovo3978\_f0 | denovo4056\_f0 | denovo4134\_f0 | denovo4238\_f0 | denovo4316\_f0 | denovo4342\_f0 | denovo4368\_f0 | denovo4394\_f0 | denovo4420\_f0 | denovo4498\_f0 | denovo4524\_f0 | denovo4628\_f0 | denovo4758\_f0 | denovo4836\_f0 | denovo4888\_f0 | denovo4914\_f0 | denovo4940\_f0 | denovo4966\_f0 | denovo4992\_f0 | denovo5096\_f0 | denovo5148\_f0 | denovo5200\_f0 | denovo5408\_f0 | denovo5434\_f0 | denovo5538\_f0 | denovo5564\_f0 | denovo5902\_f0 | denovo5954\_f0 | denovo6006\_f0 | denovo6032\_f0 | denovo6058\_f0 | denovo6084\_f0 | denovo6188\_f0 | denovo6240\_f0 | denovo6266\_f0 | denovo6292\_f0 | denovo6370\_f0 | denovo6500\_f0 | denovo6552\_f0 | denovo6578\_f0 | denovo6604\_f0 | denovo6656\_f0 | denovo6682\_f0 | denovo6708\_f0 | denovo6734\_f0 | denovo6760\_f0 | denovo6890\_f0 | denovo6968\_f0 | denovo6994\_f0 | denovo7020\_f0 | denovo7072\_f0 | denovo7124\_f0 | denovo7150\_f0 | denovo7280\_f0 | denovo7306\_f0 | denovo7332\_f0 | denovo7358\_f0 | denovo7384\_f0 | denovo7462\_f0 | denovo7488\_f0 | denovo7514\_f0 | denovo7540\_f0 | denovo7618\_f0 | denovo7670\_f0 | denovo7696\_f0 | denovo7774\_f0 | denovo7826\_f0 | denovo7904\_f0 | denovo8008\_f0 | denovo8034\_f0 | denovo8216\_f0 | denovo8242\_f0 | denovo8268\_f0 | denovo8346\_f0 | denovo8372\_f0 | denovo8398\_f0 | denovo8476\_f0 | denovo8528\_f0 | denovo8554\_f0 | denovo8606\_f0 | denovo8632\_f0 | denovo8684\_f0 | denovo8710\_f0 | denovo8736\_f0 | denovo8762\_f0 | denovo8892\_f0 | denovo8918\_f0 | denovo8944\_f0 | denovo8970\_f0 | denovo9100\_f0 | denovo9126\_f0 | denovo9230\_f0 | denovo9282\_f0 | denovo9308\_f0 | denovo9334\_f0 | denovo9438\_f0 | denovo9464\_f0 | denovo9490\_f0 | denovo9646\_f0 | denovo9698\_f0 | denovo9776\_f0 | denovo9802\_f0 | denovo9828\_f0 | denovo9880\_f0 | denovo9958\_f0 | denovo10010\_f0 | denovo10036\_f0 | denovo10192\_f0 | denovo10218\_f0 | denovo10270\_f0 | denovo10296\_f0 | denovo10426\_f0 | denovo10452\_f0 | denovo10504\_f0 | denovo10530\_f0 | denovo10556\_f0 | denovo10582\_f0 | denovo10660\_f0 | denovo10712\_f0 | denovo10738\_f0 | denovo10764\_f0 | denovo10816\_f0 | denovo10998\_f0 | denovo11024\_f0 | denovo11050\_f0 | denovo11180\_f0 | denovo11284\_f0 | denovo11336\_f0 | denovo11388\_f0 | denovo11414\_f0 | denovo11440\_f0 | denovo11466\_f0 | denovo11492\_f0 | denovo11518\_f0 | denovo11570\_f0 | denovo11596\_f0 | denovo11700\_f0 | denovo11726\_f0 | denovo11908\_f0 | denovo11934\_f0 | denovo12064\_f0 |
| Pcit2 | Phyllocnistis citrella\* | denovo19\_f0 | denovo97\_f0 | denovo175\_f0 | denovo201\_f0 | denovo279\_f0 | denovo331\_f0 | denovo383\_f0 | denovo461\_f0 | denovo539\_f0 | denovo695\_f0 | denovo851\_f0 | denovo1059\_f0 | denovo1163\_f0 | denovo1241\_f0 | denovo1267\_f0 | denovo1371\_f0 | denovo1397\_f0 | denovo1423\_f0 | denovo1501\_f0 | denovo1657\_f0 | denovo1683\_f0 | denovo1735\_f0 | denovo1813\_f0 | denovo1865\_f0 | denovo1943\_f0 | denovo1969\_f0 | denovo1995\_f0 | denovo2021\_f0 | denovo2099\_f0 | denovo2125\_f0 | denovo2151\_f0 | denovo2255\_f0 | denovo2281\_f0 | denovo2307\_f0 | denovo2333\_f0 | denovo2385\_f0 | denovo2489\_f0 | denovo2541\_f0 | denovo2567\_f0 | denovo2593\_f0 | denovo2619\_f0 | denovo2645\_f0 | denovo2671\_f0 | denovo2775\_f0 | denovo2853\_f0 | denovo2905\_f0 | denovo3165\_f0 | denovo3269\_f0 | denovo3321\_f0 | denovo3347\_f0 | denovo3451\_f0 | denovo3503\_f0 | denovo3607\_f0 | denovo3633\_f0 | denovo3659\_f0 | denovo3737\_f0 | denovo3763\_f0 | denovo3815\_f0 | denovo3841\_f0 | denovo3997\_f0 | denovo4075\_f0 | denovo4153\_f0 | denovo4257\_f0 | denovo4335\_f0 | denovo4361\_f0 | denovo4387\_f0 | denovo4413\_f0 | denovo4439\_f0 | denovo4517\_f0 | denovo4543\_f0 | denovo4647\_f0 | denovo4777\_f0 | denovo4855\_f0 | denovo4907\_f0 | denovo4933\_f0 | denovo4959\_f0 | denovo4985\_f0 | denovo5011\_f0 | denovo5115\_f0 | denovo5167\_f0 | denovo5219\_f0 | denovo5427\_f0 | denovo5453\_f0 | denovo5557\_f0 | denovo5583\_f0 | denovo5921\_f0 | denovo5973\_f0 | denovo6025\_f0 | denovo6051\_f0 | denovo6077\_f0 | denovo6103\_f0 | denovo6207\_f0 | denovo6259\_f0 | denovo6285\_f0 | denovo6311\_f0 | denovo6389\_f0 | denovo6519\_f0 | denovo6571\_f0 | denovo6597\_f0 | denovo6623\_f0 | denovo6675\_f0 | denovo6701\_f0 | denovo6727\_f0 | denovo6753\_f0 | denovo6779\_f0 | denovo6909\_f0 | denovo6987\_f0 | denovo7013\_f0 | denovo7039\_f0 | denovo7091\_f0 | denovo7143\_f0 | denovo7169\_f0 | denovo7299\_f0 | denovo7325\_f0 | denovo7351\_f0 | denovo7377\_f0 | denovo7403\_f0 | denovo7481\_f0 | denovo7507\_f0 | denovo7533\_f0 | denovo7559\_f0 | denovo7637\_f0 | denovo7689\_f0 | denovo7715\_f0 | denovo7793\_f0 | denovo7845\_f0 | denovo7923\_f0 | denovo8027\_f0 | denovo8053\_f0 | denovo8235\_f0 | denovo8261\_f0 | denovo8287\_f0 | denovo8365\_f0 | denovo8391\_f0 | denovo8417\_f0 | denovo8495\_f0 | denovo8547\_f0 | denovo8573\_f0 | denovo8625\_f0 | denovo8651\_f0 | denovo8703\_f0 | denovo8729\_f0 | denovo8755\_f0 | denovo8781\_f0 | denovo8911\_f0 | denovo8937\_f0 | denovo8963\_f0 | denovo8989\_f0 | denovo9119\_f0 | denovo9145\_f0 | denovo9249\_f0 | denovo9301\_f0 | denovo9327\_f0 | denovo9353\_f0 | denovo9457\_f0 | denovo9483\_f0 | denovo9509\_f0 | denovo9665\_f0 | denovo9717\_f0 | denovo9795\_f0 | denovo9821\_f0 | denovo9847\_f0 | denovo9899\_f0 | denovo9977\_f0 | denovo10029\_f0 | denovo10055\_f0 | denovo10211\_f0 | denovo10237\_f0 | denovo10289\_f0 | denovo10315\_f0 | denovo10445\_f0 | denovo10471\_f0 | denovo10523\_f0 | denovo10549\_f0 | denovo10575\_f0 | denovo10601\_f0 | denovo10679\_f0 | denovo10731\_f0 | denovo10757\_f0 | denovo10783\_f0 | denovo10835\_f0 | denovo11017\_f0 | denovo11043\_f0 | denovo11069\_f0 | denovo11199\_f0 | denovo11303\_f0 | denovo11355\_f0 | denovo11407\_f0 | denovo11433\_f0 | denovo11459\_f0 | denovo11485\_f0 | denovo11511\_f0 | denovo11537\_f0 | denovo11589\_f0 | denovo11615\_f0 | denovo11719\_f0 | denovo11745\_f0 | denovo11927\_f0 | denovo11953\_f0 | denovo12083\_f0 |

#### Content Of Concatenation "Entropy\_0.00\_0.00\_Loci\_150\_To\_349"

  
Rules for "entropy\_0.00\_0.00\_loci\_150\_to\_349":  
OTUs must have the loci:   
OTUs must have at least one of the following loci:

```
'EOG69CQC1_1', 'EOG69KFV8_1', 'EOG6SN1SH_1', 'EOG6PRSVJ_1', 'EOG6PRSVH_1', 'EOG60ZR1Z_1', 'EOG6FXRCN_1', 'EOG60P4BD_1', 'EOG6NCMGZ_1', 'EOG670TN2_1', 'EOG63TZZV_1', 'EOG6G7C30_1', 'EOG6K6G7Z_1', 'EOG6SXNH7_1', 'EOG65X80D_1', 'EOG68KRFS_1', 'EOG6MSD2X_1', 'EOG698V44_1', 'EOG6F7NPM_1', 'EOG676K3N_1', 'EOG6894QQ_1', 'EOG6868GW_1', 'EOG69PB31_1', 'EOG6JDHBD_1', 'EOG6C87X4_1', 'EOG68D102_1', 'EOG60ZR1T_1', 'EOG63JC89_1', 'EOG6J6RV9_1', 'EOG68D104_1', 'EOG68KRG8_1', 'EOG6N8R81_1', 'EOG63BMSV_1', 'EOG69KFV1_1', 'EOG6CJVMF_1', 'EOG6C2HF4_1', 'EOG6CC44S_1', 'EOG6FXRCK_1', 'EOG68KRG4_1', 'EOG61C70T_1', 'EOG6BK575_1', 'EOG6R506X_1', 'EOG6JT09G_1', 'EOG6HQDPG_1', 'EOG6H4677_1', 'EOG6SJ5J3_1', 'EOG6NZTZ3_1', 'EOG6J3WM7_1', 'EOG680J16_1', 'EOG602WWN_1', 'EOG6RNBCT_1', 'EOG63BMSB_1', 'EOG6B8JHB_1', 'EOG647FXX_1', 'EOG6B8JHP_1', 'EOG6S1TBX_1', 'EOG65HS25_1', 'EOG6FFD5C_1', 'EOG6PNXM9_1', 'EOG6F4SFZ_1', 'EOG6Q8524_1', 'EOG669QZR_1', 'EOG6JT09B_1', 'EOG6CJVMM_1', 'EOG6QRH81_1', 'EOG6NCMH9_1', 'EOG65HS1N_1', 'EOG6CC44T_1', 'EOG6FR0VW_1', 'EOG6KKZ6X_1', 'EOG60VVTG_1', 'EOG6QC198_1', 'EOG6FFD52_1', 'EOG634W9K_1', 'EOG6DNF7N_1', 'EOG61RPZF_1', 'EOG605S4X_1', 'EOG69GKMS_1', 'EOG6FR0VX_1', 'EOG6B8JHC_1', 'EOG6QVCGS_1', 'EOG6Q2DJZ_1', 'EOG6CC44M_1', 'EOG6NS3G8_1', 'EOG68GW77_1', 'EOG6229Q2_1', 'EOG63TZZW_1', 'EOG6MW89D_1', 'EOG6SJ5K0_1', 'EOG6MKNM3_1', 'EOG63V00H_1', 'EOG6BG90S_1', 'EOG66DM62_1', 'EOG69CQBW_1', 'EOG69KFTV_1', 'EOG62V8KS_1', 'EOG6FFD51_1', 'EOG6QNN0J_1', 'EOG6PC9WN_1', 'EOG6Q8529_1', 'EOG6KSPP5_1', 'EOG61ZFF6_1', 'EOG6N5W22_1', 'EOG63V009_1', 'EOG6PK2CG_1', 'EOG68GW6V_1', 'EOG66Q6XP_1', 'EOG6C5CNP_1', 'EOG6R5066_1', 'EOG641QFN_1', 'EOG65B1K2_1', 'EOG65TCRK_1', 'EOG6PNXN7_1', 'EOG6P2Q64_1', 'EOG666VQC_1', 'EOG6PC9WF_1', 'EOG6Q58T0_1', 'EOG641QGH_1', 'EOG6933N7_1', 'EOG69S6BG_1', 'EOG66Q6XR_1', 'EOG6PK2CM_1', 'EOG6NS3FV_1', 'EOG641QFW_1', 'EOG6N04J6_1', 'EOG6CRM3G_1', 'EOG6S4PKX_1', 'EOG6K6G7S_1', 'EOG6BZN62_1', 'EOG68SGZ1_1', 'EOG6SXNGX_1', 'EOG6QZ7Q8_1', 'EOG6QC18P_1', 'EOG6CG0CT_1', 'EOG6PK2CQ_1', 'EOG6JWVJQ_1', 'EOG6PG64S_1', 'EOG63R3RK_1', 'EOG64F6DK_1', 'EOG6FJ8D6_1', 'EOG6001NN_1', 'EOG695ZWC_1', 'EOG67PX9B_1', 'EOG6P8FNH_1', 'EOG6KSPQ0_1', 'EOG63R3RC_1', 'EOG6907D7_1', 'EOG6JWVHT_1', 'EOG68SGX8_1', 'EOG6933NR_1', 'EOG62JNVR_1', 'EOG64MXWZ_1', 'EOG6S1TC2_1', 'EOG6PC9X3_1', 'EOG6Q58SW_1', 'EOG6CRM3H_1', 'EOG6R5069_1', 'EOG64MXWK_1', 'EOG6N30SN_1', 'EOG68KRFQ_1', 'EOG6DR9GZ_1', 'EOG62FSN7_1', 'EOG6PZJB1_1', 'EOG65QHHP_1', 'EOG615GHV_1', 'EOG66WZCQ_1', 'EOG698V4H_1', 'EOG6JDHB2_1', 'EOG62BXCX_1', 'EOG69KFVF_1', 'EOG615GHP_1', 'EOG66HGF5_1', 'EOG6DFPSF_1', 'EOG66MBPR_1', 'EOG6DFPSD_1', 'EOG67D9KC_1', 'EOG6HHP5S_1', 'EOG6DBTHZ_1', 'EOG6KH2ZD_1', 'EOG6STS87_1', 'EOG67D9KV_1', 'EOG65MN8T_1', 'EOG61RPZ9_1', 'EOG66MBP7_1', 'EOG6QJRRQ_1', 'EOG6Q852B_1', 'EOG6DV5QM_1', 'EOG6K0QRT_1', 'EOG65DWSM_1', 'EOG6G4GTX_1', 'EOG64BB5H_1', 'EOG64J2NC_1', 'EOG6H72G3_1', 'EOG6SBF2B_1', 'EOG6FFD5V_1', 'EOG6G4GTT_1', 'EOG6K3M11_1', 'EOG66147J_1', 'EOG63FH1C_1', 'EOG61C70W_1'
```

  

|  |  | EOG69CQC1\_1 | EOG69KFV8\_1 | EOG6SN1SH\_1 | EOG6PRSVJ\_1 | EOG6PRSVH\_1 | EOG60ZR1Z\_1 | EOG6FXRCN\_1 | EOG60P4BD\_1 | EOG6NCMGZ\_1 | EOG670TN2\_1 | EOG63TZZV\_1 | EOG6G7C30\_1 | EOG6K6G7Z\_1 | EOG6SXNH7\_1 | EOG65X80D\_1 | EOG68KRFS\_1 | EOG6MSD2X\_1 | EOG698V44\_1 | EOG6F7NPM\_1 | EOG676K3N\_1 | EOG6894QQ\_1 | EOG6868GW\_1 | EOG69PB31\_1 | EOG6JDHBD\_1 | EOG6C87X4\_1 | EOG68D102\_1 | EOG60ZR1T\_1 | EOG63JC89\_1 | EOG6J6RV9\_1 | EOG68D104\_1 | EOG68KRG8\_1 | EOG6N8R81\_1 | EOG63BMSV\_1 | EOG69KFV1\_1 | EOG6CJVMF\_1 | EOG6C2HF4\_1 | EOG6CC44S\_1 | EOG6FXRCK\_1 | EOG68KRG4\_1 | EOG61C70T\_1 | EOG6BK575\_1 | EOG6R506X\_1 | EOG6JT09G\_1 | EOG6HQDPG\_1 | EOG6H4677\_1 | EOG6SJ5J3\_1 | EOG6NZTZ3\_1 | EOG6J3WM7\_1 | EOG680J16\_1 | EOG602WWN\_1 | EOG6RNBCT\_1 | EOG63BMSB\_1 | EOG6B8JHB\_1 | EOG647FXX\_1 | EOG6B8JHP\_1 | EOG6S1TBX\_1 | EOG65HS25\_1 | EOG6FFD5C\_1 | EOG6PNXM9\_1 | EOG6F4SFZ\_1 | EOG6Q8524\_1 | EOG669QZR\_1 | EOG6JT09B\_1 | EOG6CJVMM\_1 | EOG6QRH81\_1 | EOG6NCMH9\_1 | EOG65HS1N\_1 | EOG6CC44T\_1 | EOG6FR0VW\_1 | EOG6KKZ6X\_1 | EOG60VVTG\_1 | EOG6QC198\_1 | EOG6FFD52\_1 | EOG634W9K\_1 | EOG6DNF7N\_1 | EOG61RPZF\_1 | EOG605S4X\_1 | EOG69GKMS\_1 | EOG6FR0VX\_1 | EOG6B8JHC\_1 | EOG6QVCGS\_1 | EOG6Q2DJZ\_1 | EOG6CC44M\_1 | EOG6NS3G8\_1 | EOG68GW77\_1 | EOG6229Q2\_1 | EOG63TZZW\_1 | EOG6MW89D\_1 | EOG6SJ5K0\_1 | EOG6MKNM3\_1 | EOG63V00H\_1 | EOG6BG90S\_1 | EOG66DM62\_1 | EOG69CQBW\_1 | EOG69KFTV\_1 | EOG62V8KS\_1 | EOG6FFD51\_1 | EOG6QNN0J\_1 | EOG6PC9WN\_1 | EOG6Q8529\_1 | EOG6KSPP5\_1 | EOG61ZFF6\_1 | EOG6N5W22\_1 | EOG63V009\_1 | EOG6PK2CG\_1 | EOG68GW6V\_1 | EOG66Q6XP\_1 | EOG6C5CNP\_1 | EOG6R5066\_1 | EOG641QFN\_1 | EOG65B1K2\_1 | EOG65TCRK\_1 | EOG6PNXN7\_1 | EOG6P2Q64\_1 | EOG666VQC\_1 | EOG6PC9WF\_1 | EOG6Q58T0\_1 | EOG641QGH\_1 | EOG6933N7\_1 | EOG69S6BG\_1 | EOG66Q6XR\_1 | EOG6PK2CM\_1 | EOG6NS3FV\_1 | EOG641QFW\_1 | EOG6N04J6\_1 | EOG6CRM3G\_1 | EOG6S4PKX\_1 | EOG6K6G7S\_1 | EOG6BZN62\_1 | EOG68SGZ1\_1 | EOG6SXNGX\_1 | EOG6QZ7Q8\_1 | EOG6QC18P\_1 | EOG6CG0CT\_1 | EOG6PK2CQ\_1 | EOG6JWVJQ\_1 | EOG6PG64S\_1 | EOG63R3RK\_1 | EOG64F6DK\_1 | EOG6FJ8D6\_1 | EOG6001NN\_1 | EOG695ZWC\_1 | EOG67PX9B\_1 | EOG6P8FNH\_1 | EOG6KSPQ0\_1 | EOG63R3RC\_1 | EOG6907D7\_1 | EOG6JWVHT\_1 | EOG68SGX8\_1 | EOG6933NR\_1 | EOG62JNVR\_1 | EOG64MXWZ\_1 | EOG6S1TC2\_1 | EOG6PC9X3\_1 | EOG6Q58SW\_1 | EOG6CRM3H\_1 | EOG6R5069\_1 | EOG64MXWK\_1 | EOG6N30SN\_1 | EOG68KRFQ\_1 | EOG6DR9GZ\_1 | EOG62FSN7\_1 | EOG6PZJB1\_1 | EOG65QHHP\_1 | EOG615GHV\_1 | EOG66WZCQ\_1 | EOG698V4H\_1 | EOG6JDHB2\_1 | EOG62BXCX\_1 | EOG69KFVF\_1 | EOG615GHP\_1 | EOG66HGF5\_1 | EOG6DFPSF\_1 | EOG66MBPR\_1 | EOG6DFPSD\_1 | EOG67D9KC\_1 | EOG6HHP5S\_1 | EOG6DBTHZ\_1 | EOG6KH2ZD\_1 | EOG6STS87\_1 | EOG67D9KV\_1 | EOG65MN8T\_1 | EOG61RPZ9\_1 | EOG66MBP7\_1 | EOG6QJRRQ\_1 | EOG6Q852B\_1 | EOG6DV5QM\_1 | EOG6K0QRT\_1 | EOG65DWSM\_1 | EOG6G4GTX\_1 | EOG64BB5H\_1 | EOG64J2NC\_1 | EOG6H72G3\_1 | EOG6SBF2B\_1 | EOG6FFD5V\_1 | EOG6G4GTT\_1 | EOG6K3M11\_1 | EOG66147J\_1 | EOG63FH1C\_1 | EOG61C70W\_1 |
| --- | --- | --- | --- | --- | --- | --- | --- | --- | --- | --- | --- | --- | --- | --- | --- | --- | --- | --- | --- | --- | --- | --- | --- | --- | --- | --- | --- | --- | --- | --- | --- | --- | --- | --- | --- | --- | --- | --- | --- | --- | --- | --- | --- | --- | --- | --- | --- | --- | --- | --- | --- | --- | --- | --- | --- | --- | --- | --- | --- | --- | --- | --- | --- | --- | --- | --- | --- | --- | --- | --- | --- | --- | --- | --- | --- | --- | --- | --- | --- | --- | --- | --- | --- | --- | --- | --- | --- | --- | --- | --- | --- | --- | --- | --- | --- | --- | --- | --- | --- | --- | --- | --- | --- | --- | --- | --- | --- | --- | --- | --- | --- | --- | --- | --- | --- | --- | --- | --- | --- | --- | --- | --- | --- | --- | --- | --- | --- | --- | --- | --- | --- | --- | --- | --- | --- | --- | --- | --- | --- | --- | --- | --- | --- | --- | --- | --- | --- | --- | --- | --- | --- | --- | --- | --- | --- | --- | --- | --- | --- | --- | --- | --- | --- | --- | --- | --- | --- | --- | --- | --- | --- | --- | --- | --- | --- | --- | --- | --- | --- | --- | --- | --- | --- | --- | --- | --- | --- | --- | --- | --- | --- | --- | --- | --- | --- | --- | --- | --- | --- | --- | --- |
| Dplexcds | Danaus plexippus\* | denovo2\_f0 | denovo54\_f0 | denovo80\_f0 | denovo158\_f0 | denovo184\_f0 | denovo262\_f0 | denovo314\_f0 | denovo366\_f0 | denovo444\_f0 | denovo548\_f0 | denovo678\_f0 | denovo834\_f0 | denovo860\_f0 | denovo886\_f0 | denovo912\_f0 | denovo1042\_f0 | denovo1172\_f0 | denovo1224\_f0 | denovo1250\_f0 | denovo1276\_f0 | denovo1354\_f0 | denovo1380\_f0 | denovo1406\_f0 | denovo1484\_f0 | denovo1562\_f0 | denovo1640\_f0 | denovo1666\_f0 | denovo1718\_f0 | denovo1848\_f0 | denovo1900\_f0 | denovo1926\_f0 | denovo1952\_f0 | denovo1978\_f0 | denovo2004\_f0 | denovo2056\_f0 | denovo2082\_f0 | denovo2108\_f0 | denovo2134\_f0 | denovo2238\_f0 | denovo2264\_f0 | denovo2290\_f0 | denovo2316\_f0 | denovo2368\_f0 | denovo2394\_f0 | denovo2420\_f0 | denovo2550\_f0 | denovo2576\_f0 | denovo2602\_f0 | denovo2628\_f0 | denovo2732\_f0 | denovo2758\_f0 | denovo2810\_f0 | denovo2888\_f0 | denovo3304\_f0 | denovo3330\_f0 | denovo3434\_f0 | denovo3590\_f0 | denovo3616\_f0 | denovo3642\_f0 | denovo3720\_f0 | denovo3746\_f0 | denovo3798\_f0 | denovo3824\_f0 | denovo3876\_f0 | denovo4058\_f0 | denovo4084\_f0 | denovo4136\_f0 | denovo4162\_f0 | denovo4240\_f0 | denovo4318\_f0 | denovo4344\_f0 | denovo4370\_f0 | denovo4422\_f0 | denovo4500\_f0 | denovo4526\_f0 | denovo4630\_f0 | denovo4838\_f0 | denovo4994\_f0 | denovo5020\_f0 | denovo5098\_f0 | denovo5124\_f0 | denovo5150\_f0 | denovo5332\_f0 | denovo5410\_f0 | denovo5488\_f0 | denovo5540\_f0 | denovo5566\_f0 | denovo5722\_f0 | denovo5774\_f0 | denovo5852\_f0 | denovo5878\_f0 | denovo5904\_f0 | denovo5956\_f0 | denovo6034\_f0 | denovo6086\_f0 | denovo6190\_f0 | denovo6268\_f0 | denovo6294\_f0 | denovo6320\_f0 | denovo6346\_f0 | denovo6372\_f0 | denovo6476\_f0 | denovo6502\_f0 | denovo6528\_f0 | denovo6554\_f0 | denovo6632\_f0 | denovo6658\_f0 | denovo6710\_f0 | denovo6736\_f0 | denovo6892\_f0 | denovo6970\_f0 | denovo7022\_f0 | denovo7048\_f0 | denovo7126\_f0 | denovo7152\_f0 | denovo7282\_f0 | denovo7308\_f0 | denovo7334\_f0 | denovo7360\_f0 | denovo7386\_f0 | denovo7438\_f0 | denovo7464\_f0 | denovo7490\_f0 | denovo7542\_f0 | denovo7620\_f0 | denovo7646\_f0 | denovo7698\_f0 | denovo7776\_f0 | denovo7828\_f0 | denovo7854\_f0 | denovo7906\_f0 | denovo7932\_f0 | denovo8010\_f0 | denovo8036\_f0 | denovo8088\_f0 | denovo8140\_f0 | denovo8192\_f0 | denovo8348\_f0 | denovo8374\_f0 | denovo8400\_f0 | denovo8478\_f0 | denovo8530\_f0 | denovo8556\_f0 | denovo8608\_f0 | denovo8634\_f0 | denovo8686\_f0 | denovo8712\_f0 | denovo8738\_f0 | denovo8764\_f0 | denovo9050\_f0 | denovo9076\_f0 | denovo9102\_f0 | denovo9128\_f0 | denovo9206\_f0 | denovo9232\_f0 | denovo9284\_f0 | denovo9336\_f0 | denovo9466\_f0 | denovo9492\_f0 | denovo9622\_f0 | denovo9648\_f0 | denovo9778\_f0 | denovo9830\_f0 | denovo9882\_f0 | denovo9960\_f0 | denovo10012\_f0 | denovo10038\_f0 | denovo10116\_f0 | denovo10142\_f0 | denovo10272\_f0 | denovo10298\_f0 | denovo10428\_f0 | denovo10454\_f0 | denovo10506\_f0 | denovo10532\_f0 | denovo10558\_f0 | denovo10584\_f0 | denovo10714\_f0 | denovo10740\_f0 | denovo10766\_f0 | denovo10818\_f0 | denovo10896\_f0 | denovo11000\_f0 | denovo11052\_f0 | denovo11130\_f0 | denovo11208\_f0 | denovo11286\_f0 | denovo11390\_f0 | denovo11416\_f0 | denovo11468\_f0 | denovo11494\_f0 | denovo11520\_f0 | denovo11572\_f0 | denovo11598\_f0 | denovo11702\_f0 | denovo11728\_f0 | denovo11754\_f0 | denovo11858\_f0 | denovo11884\_f0 | denovo11936\_f0 |
| FG120077 | Semomesia campanea | denovo10\_f0 | denovo62\_f0 | denovo88\_f0 | denovo166\_f0 | denovo192\_f0 | denovo270\_f0 | denovo322\_f0 | denovo374\_f0 | denovo452\_f0 | denovo556\_f0 | denovo686\_f0 | denovo842\_f0 | denovo868\_f0 | denovo894\_f0 | denovo920\_f0 | denovo1050\_f0 | denovo1180\_f0 | denovo1232\_f0 | denovo1258\_f0 | denovo1284\_f0 | denovo1362\_f0 | denovo1388\_f0 | denovo1414\_f0 | denovo1492\_f0 | denovo1570\_f0 | denovo1648\_f0 | denovo1674\_f0 | denovo1726\_f0 | denovo1856\_f0 | denovo1908\_f0 | denovo1934\_f0 | denovo1960\_f0 | denovo1986\_f0 | denovo2012\_f0 | denovo2064\_f0 | denovo2090\_f0 | denovo2116\_f0 | denovo2142\_f0 | denovo2246\_f0 | denovo2272\_f0 | denovo2298\_f0 | denovo2324\_f0 | denovo2376\_f0 | denovo2402\_f0 | denovo2428\_f0 | denovo2558\_f0 | denovo2584\_f0 | denovo2610\_f0 | denovo2636\_f0 | denovo2740\_f0 | denovo2766\_f0 | denovo2818\_f0 | denovo2896\_f0 | denovo3312\_f0 | denovo3338\_f0 | denovo3442\_f0 | denovo3598\_f0 | denovo3624\_f0 | denovo3650\_f0 | denovo3728\_f0 | denovo3754\_f0 | denovo3806\_f0 | denovo3832\_f0 | denovo3884\_f0 | denovo4066\_f0 | denovo4092\_f0 | denovo4144\_f0 | denovo4170\_f0 | denovo4248\_f0 | denovo4326\_f0 | denovo4352\_f0 | denovo4378\_f0 | denovo4430\_f0 | denovo4508\_f0 | denovo4534\_f0 | denovo4638\_f0 | denovo4846\_f0 | denovo5002\_f0 | denovo5028\_f0 | denovo5106\_f0 | denovo5132\_f0 | denovo5158\_f0 | denovo5340\_f0 | denovo5418\_f0 | denovo5496\_f0 | denovo5548\_f0 | denovo5574\_f0 | denovo5730\_f0 | denovo5782\_f0 | denovo5860\_f0 | denovo5886\_f0 | denovo5912\_f0 | denovo5964\_f0 | denovo6042\_f0 | denovo6094\_f0 | denovo6198\_f0 | denovo6276\_f0 | denovo6302\_f0 | denovo6328\_f0 | denovo6354\_f0 | denovo6380\_f0 | denovo6484\_f0 | denovo6510\_f0 | denovo6536\_f0 | denovo6562\_f0 | denovo6640\_f0 | denovo6666\_f0 | denovo6718\_f0 | denovo6744\_f0 | denovo6900\_f0 | denovo6978\_f0 | denovo7030\_f0 | denovo7056\_f0 | denovo7134\_f0 | denovo7160\_f0 | denovo7290\_f0 | denovo7316\_f0 | denovo7342\_f0 | denovo7368\_f0 | denovo7394\_f0 | denovo7446\_f0 | denovo7472\_f0 | denovo7498\_f0 | denovo7550\_f0 | denovo7628\_f0 | denovo7654\_f0 | denovo7706\_f0 | denovo7784\_f0 | denovo7836\_f0 | denovo7862\_f0 | denovo7914\_f0 | denovo7940\_f0 | denovo8018\_f0 | denovo8044\_f0 | denovo8096\_f0 | denovo8148\_f0 | denovo8200\_f0 | denovo8356\_f0 | denovo8382\_f0 | denovo8408\_f0 | denovo8486\_f0 | denovo8538\_f0 | denovo8564\_f0 | denovo8616\_f0 | denovo8642\_f0 | denovo8694\_f0 | denovo8720\_f0 | denovo8746\_f0 | denovo8772\_f0 | denovo9058\_f0 | denovo9084\_f0 | denovo9110\_f0 | denovo9136\_f0 | denovo9214\_f0 | denovo9240\_f0 | denovo9292\_f0 | denovo9344\_f0 | denovo9474\_f0 | denovo9500\_f0 | denovo9630\_f0 | denovo9656\_f0 | denovo9786\_f0 | denovo9838\_f0 | denovo9890\_f0 | denovo9968\_f0 | denovo10020\_f0 | denovo10046\_f0 | denovo10124\_f0 | denovo10150\_f0 | denovo10280\_f0 | denovo10306\_f0 | denovo10436\_f0 | denovo10462\_f0 | denovo10514\_f0 | denovo10540\_f0 | denovo10566\_f0 | denovo10592\_f0 | denovo10722\_f0 | denovo10748\_f0 | denovo10774\_f0 | denovo10826\_f0 | denovo10904\_f0 | denovo11008\_f0 | denovo11060\_f0 | denovo11138\_f0 | denovo11216\_f0 | denovo11294\_f0 | denovo11398\_f0 | denovo11424\_f0 | denovo11476\_f0 | denovo11502\_f0 | denovo11528\_f0 | denovo11580\_f0 | denovo11606\_f0 | denovo11710\_f0 | denovo11736\_f0 | denovo11762\_f0 | denovo11866\_f0 | denovo11892\_f0 | denovo11944\_f0 |
| SRR803483 | Grapholita dimorpha\* | denovo20\_f0 | denovo72\_f0 | denovo98\_f0 | denovo176\_f0 | denovo202\_f0 | denovo280\_f0 | denovo332\_f0 | denovo384\_f0 | denovo462\_f0 | denovo566\_f0 | denovo696\_f0 | denovo852\_f0 | denovo878\_f0 | denovo904\_f0 | denovo930\_f0 | denovo1060\_f0 | denovo1190\_f0 | denovo1242\_f0 | denovo1268\_f0 | denovo1294\_f0 | denovo1372\_f0 | denovo1398\_f0 | denovo1424\_f0 | denovo1502\_f0 | denovo1580\_f0 | denovo1658\_f0 | denovo1684\_f0 | denovo1736\_f0 | denovo1866\_f0 | denovo1918\_f0 | denovo1944\_f0 | denovo1970\_f0 | denovo1996\_f0 | denovo2022\_f0 | denovo2074\_f0 | denovo2100\_f0 | denovo2126\_f0 | denovo2152\_f0 | denovo2256\_f0 | denovo2282\_f0 | denovo2308\_f0 | denovo2334\_f0 | denovo2386\_f0 | denovo2412\_f0 | denovo2438\_f0 | denovo2568\_f0 | denovo2594\_f0 | denovo2620\_f0 | denovo2646\_f0 | denovo2750\_f0 | denovo2776\_f0 | denovo2828\_f0 | denovo2906\_f0 | denovo3322\_f0 | denovo3348\_f0 | denovo3452\_f0 | denovo3608\_f0 | denovo3634\_f0 | denovo3660\_f0 | denovo3738\_f0 | denovo3764\_f0 | denovo3816\_f0 | denovo3842\_f0 | denovo3894\_f0 | denovo4076\_f0 | denovo4102\_f0 | denovo4154\_f0 | denovo4180\_f0 | denovo4258\_f0 | denovo4336\_f0 | denovo4362\_f0 | denovo4388\_f0 | denovo4440\_f0 | denovo4518\_f0 | denovo4544\_f0 | denovo4648\_f0 | denovo4856\_f0 | denovo5012\_f0 | denovo5038\_f0 | denovo5116\_f0 | denovo5142\_f0 | denovo5168\_f0 | denovo5350\_f0 | denovo5428\_f0 | denovo5506\_f0 | denovo5558\_f0 | denovo5584\_f0 | denovo5740\_f0 | denovo5792\_f0 | denovo5870\_f0 | denovo5896\_f0 | denovo5922\_f0 | denovo5974\_f0 | denovo6052\_f0 | denovo6104\_f0 | denovo6208\_f0 | denovo6286\_f0 | denovo6312\_f0 | denovo6338\_f0 | denovo6364\_f0 | denovo6390\_f0 | denovo6494\_f0 | denovo6520\_f0 | denovo6546\_f0 | denovo6572\_f0 | denovo6650\_f0 | denovo6676\_f0 | denovo6728\_f0 | denovo6754\_f0 | denovo6910\_f0 | denovo6988\_f0 | denovo7040\_f0 | denovo7066\_f0 | denovo7144\_f0 | denovo7170\_f0 | denovo7300\_f0 | denovo7326\_f0 | denovo7352\_f0 | denovo7378\_f0 | denovo7404\_f0 | denovo7456\_f0 | denovo7482\_f0 | denovo7508\_f0 | denovo7560\_f0 | denovo7638\_f0 | denovo7664\_f0 | denovo7716\_f0 | denovo7794\_f0 | denovo7846\_f0 | denovo7872\_f0 | denovo7924\_f0 | denovo7950\_f0 | denovo8028\_f0 | denovo8054\_f0 | denovo8106\_f0 | denovo8158\_f0 | denovo8210\_f0 | denovo8366\_f0 | denovo8392\_f0 | denovo8418\_f0 | denovo8496\_f0 | denovo8548\_f0 | denovo8574\_f0 | denovo8626\_f0 | denovo8652\_f0 | denovo8704\_f0 | denovo8730\_f0 | denovo8756\_f0 | denovo8782\_f0 | denovo9068\_f0 | denovo9094\_f0 | denovo9120\_f0 | denovo9146\_f0 | denovo9224\_f0 | denovo9250\_f0 | denovo9302\_f0 | denovo9354\_f0 | denovo9484\_f0 | denovo9510\_f0 | denovo9640\_f0 | denovo9666\_f0 | denovo9796\_f0 | denovo9848\_f0 | denovo9900\_f0 | denovo9978\_f0 | denovo10030\_f0 | denovo10056\_f0 | denovo10134\_f0 | denovo10160\_f0 | denovo10290\_f0 | denovo10316\_f0 | denovo10446\_f0 | denovo10472\_f0 | denovo10524\_f0 | denovo10550\_f0 | denovo10576\_f0 | denovo10602\_f0 | denovo10732\_f0 | denovo10758\_f0 | denovo10784\_f0 | denovo10836\_f0 | denovo10914\_f0 | denovo11018\_f0 | denovo11070\_f0 | denovo11148\_f0 | denovo11226\_f0 | denovo11304\_f0 | denovo11408\_f0 | denovo11434\_f0 | denovo11486\_f0 | denovo11512\_f0 | denovo11538\_f0 | denovo11590\_f0 | denovo11616\_f0 | denovo11720\_f0 | denovo11746\_f0 | denovo11772\_f0 | denovo11876\_f0 | denovo11902\_f0 | denovo11954\_f0 |
| FG120035 | Dalcera abrasa\* | denovo5\_f0 | denovo57\_f0 | denovo83\_f0 | denovo161\_f0 | denovo187\_f0 | denovo265\_f0 | denovo317\_f0 | denovo369\_f0 | denovo447\_f0 | denovo551\_f0 | denovo681\_f0 | denovo837\_f0 | denovo863\_f0 | denovo889\_f0 | denovo915\_f0 | denovo1045\_f0 | denovo1175\_f0 | denovo1227\_f0 | denovo1253\_f0 | denovo1279\_f0 | denovo1357\_f0 | denovo1383\_f0 | denovo1409\_f0 | denovo1487\_f0 | denovo1565\_f0 | denovo1643\_f0 | denovo1669\_f0 | denovo1721\_f0 | denovo1851\_f0 | denovo1903\_f0 | denovo1929\_f0 | denovo1955\_f0 | denovo1981\_f0 | denovo2007\_f0 | denovo2059\_f0 | denovo2085\_f0 | denovo2111\_f0 | denovo2137\_f0 | denovo2241\_f0 | denovo2267\_f0 | denovo2293\_f0 | denovo2319\_f0 | denovo2371\_f0 | denovo2397\_f0 | denovo2423\_f0 | denovo2553\_f0 | denovo2579\_f0 | denovo2605\_f0 | denovo2631\_f0 | denovo2735\_f0 | denovo2761\_f0 | denovo2813\_f0 | denovo2891\_f0 | denovo3307\_f0 | denovo3333\_f0 | denovo3437\_f0 | denovo3593\_f0 | denovo3619\_f0 | denovo3645\_f0 | denovo3723\_f0 | denovo3749\_f0 | denovo3801\_f0 | denovo3827\_f0 | denovo3879\_f0 | denovo4061\_f0 | denovo4087\_f0 | denovo4139\_f0 | denovo4165\_f0 | denovo4243\_f0 | denovo4321\_f0 | denovo4347\_f0 | denovo4373\_f0 | denovo4425\_f0 | denovo4503\_f0 | denovo4529\_f0 | denovo4633\_f0 | denovo4841\_f0 | denovo4997\_f0 | denovo5023\_f0 | denovo5101\_f0 | denovo5127\_f0 | denovo5153\_f0 | denovo5335\_f0 | denovo5413\_f0 | denovo5491\_f0 | denovo5543\_f0 | denovo5569\_f0 | denovo5725\_f0 | denovo5777\_f0 | denovo5855\_f0 | denovo5881\_f0 | denovo5907\_f0 | denovo5959\_f0 | denovo6037\_f0 | denovo6089\_f0 | denovo6193\_f0 | denovo6271\_f0 | denovo6297\_f0 | denovo6323\_f0 | denovo6349\_f0 | denovo6375\_f0 | denovo6479\_f0 | denovo6505\_f0 | denovo6531\_f0 | denovo6557\_f0 | denovo6635\_f0 | denovo6661\_f0 | denovo6713\_f0 | denovo6739\_f0 | denovo6895\_f0 | denovo6973\_f0 | denovo7025\_f0 | denovo7051\_f0 | denovo7129\_f0 | denovo7155\_f0 | denovo7285\_f0 | denovo7311\_f0 | denovo7337\_f0 | denovo7363\_f0 | denovo7389\_f0 | denovo7441\_f0 | denovo7467\_f0 | denovo7493\_f0 | denovo7545\_f0 | denovo7623\_f0 | denovo7649\_f0 | denovo7701\_f0 | denovo7779\_f0 | denovo7831\_f0 | denovo7857\_f0 | denovo7909\_f0 | denovo7935\_f0 | denovo8013\_f0 | denovo8039\_f0 | denovo8091\_f0 | denovo8143\_f0 | denovo8195\_f0 | denovo8351\_f0 | denovo8377\_f0 | denovo8403\_f0 | denovo8481\_f0 | denovo8533\_f0 | denovo8559\_f0 | denovo8611\_f0 | denovo8637\_f0 | denovo8689\_f0 | denovo8715\_f0 | denovo8741\_f0 | denovo8767\_f0 | denovo9053\_f0 | denovo9079\_f0 | denovo9105\_f0 | denovo9131\_f0 | denovo9209\_f0 | denovo9235\_f0 | denovo9287\_f0 | denovo9339\_f0 | denovo9469\_f0 | denovo9495\_f0 | denovo9625\_f0 | denovo9651\_f0 | denovo9781\_f0 | denovo9833\_f0 | denovo9885\_f0 | denovo9963\_f0 | denovo10015\_f0 | denovo10041\_f0 | denovo10119\_f0 | denovo10145\_f0 | denovo10275\_f0 | denovo10301\_f0 | denovo10431\_f0 | denovo10457\_f0 | denovo10509\_f0 | denovo10535\_f0 | denovo10561\_f0 | denovo10587\_f0 | denovo10717\_f0 | denovo10743\_f0 | denovo10769\_f0 | denovo10821\_f0 | denovo10899\_f0 | denovo11003\_f0 | denovo11055\_f0 | denovo11133\_f0 | denovo11211\_f0 | denovo11289\_f0 | denovo11393\_f0 | denovo11419\_f0 | denovo11471\_f0 | denovo11497\_f0 | denovo11523\_f0 | denovo11575\_f0 | denovo11601\_f0 | denovo11705\_f0 | denovo11731\_f0 | denovo11757\_f0 | denovo11861\_f0 | denovo11887\_f0 | denovo11939\_f0 |
| FG120046B | Lacosoma ludolpha\* | denovo6\_f0 | denovo58\_f0 | denovo84\_f0 | denovo162\_f0 | denovo188\_f0 | denovo266\_f0 | denovo318\_f0 | denovo370\_f0 | denovo448\_f0 | denovo552\_f0 | denovo682\_f0 | denovo838\_f0 | denovo864\_f0 | denovo890\_f0 | denovo916\_f0 | denovo1046\_f0 | denovo1176\_f0 | denovo1228\_f0 | denovo1254\_f0 | denovo1280\_f0 | denovo1358\_f0 | denovo1384\_f0 | denovo1410\_f0 | denovo1488\_f0 | denovo1566\_f0 | denovo1644\_f0 | denovo1670\_f0 | denovo1722\_f0 | denovo1852\_f0 | denovo1904\_f0 | denovo1930\_f0 | denovo1956\_f0 | denovo1982\_f0 | denovo2008\_f0 | denovo2060\_f0 | denovo2086\_f0 | denovo2112\_f0 | denovo2138\_f0 | denovo2242\_f0 | denovo2268\_f0 | denovo2294\_f0 | denovo2320\_f0 | denovo2372\_f0 | denovo2398\_f0 | denovo2424\_f0 | denovo2554\_f0 | denovo2580\_f0 | denovo2606\_f0 | denovo2632\_f0 | denovo2736\_f0 | denovo2762\_f0 | denovo2814\_f0 | denovo2892\_f0 | denovo3308\_f0 | denovo3334\_f0 | denovo3438\_f0 | denovo3594\_f0 | denovo3620\_f0 | denovo3646\_f0 | denovo3724\_f0 | denovo3750\_f0 | denovo3802\_f0 | denovo3828\_f0 | denovo3880\_f0 | denovo4062\_f0 | denovo4088\_f0 | denovo4140\_f0 | denovo4166\_f0 | denovo4244\_f0 | denovo4322\_f0 | denovo4348\_f0 | denovo4374\_f0 | denovo4426\_f0 | denovo4504\_f0 | denovo4530\_f0 | denovo4634\_f0 | denovo4842\_f0 | denovo4998\_f0 | denovo5024\_f0 | denovo5102\_f0 | denovo5128\_f0 | denovo5154\_f0 | denovo5336\_f0 | denovo5414\_f0 | denovo5492\_f0 | denovo5544\_f0 | denovo5570\_f0 | denovo5726\_f0 | denovo5778\_f0 | denovo5856\_f0 | denovo5882\_f0 | denovo5908\_f0 | denovo5960\_f0 | denovo6038\_f0 | denovo6090\_f0 | denovo6194\_f0 | denovo6272\_f0 | denovo6298\_f0 | denovo6324\_f0 | denovo6350\_f0 | denovo6376\_f0 | denovo6480\_f0 | denovo6506\_f0 | denovo6532\_f0 | denovo6558\_f0 | denovo6636\_f0 | denovo6662\_f0 | denovo6714\_f0 | denovo6740\_f0 | denovo6896\_f0 | denovo6974\_f0 | denovo7026\_f0 | denovo7052\_f0 | denovo7130\_f0 | denovo7156\_f0 | denovo7286\_f0 | denovo7312\_f0 | denovo7338\_f0 | denovo7364\_f0 | denovo7390\_f0 | denovo7442\_f0 | denovo7468\_f0 | denovo7494\_f0 | denovo7546\_f0 | denovo7624\_f0 | denovo7650\_f0 | denovo7702\_f0 | denovo7780\_f0 | denovo7832\_f0 | denovo7858\_f0 | denovo7910\_f0 | denovo7936\_f0 | denovo8014\_f0 | denovo8040\_f0 | denovo8092\_f0 | denovo8144\_f0 | denovo8196\_f0 | denovo8352\_f0 | denovo8378\_f0 | denovo8404\_f0 | denovo8482\_f0 | denovo8534\_f0 | denovo8560\_f0 | denovo8612\_f0 | denovo8638\_f0 | denovo8690\_f0 | denovo8716\_f0 | denovo8742\_f0 | denovo8768\_f0 | denovo9054\_f0 | denovo9080\_f0 | denovo9106\_f0 | denovo9132\_f0 | denovo9210\_f0 | denovo9236\_f0 | denovo9288\_f0 | denovo9340\_f0 | denovo9470\_f0 | denovo9496\_f0 | denovo9626\_f0 | denovo9652\_f0 | denovo9782\_f0 | denovo9834\_f0 | denovo9886\_f0 | denovo9964\_f0 | denovo10016\_f0 | denovo10042\_f0 | denovo10120\_f0 | denovo10146\_f0 | denovo10276\_f0 | denovo10302\_f0 | denovo10432\_f0 | denovo10458\_f0 | denovo10510\_f0 | denovo10536\_f0 | denovo10562\_f0 | denovo10588\_f0 | denovo10718\_f0 | denovo10744\_f0 | denovo10770\_f0 | denovo10822\_f0 | denovo10900\_f0 | denovo11004\_f0 | denovo11056\_f0 | denovo11134\_f0 | denovo11212\_f0 | denovo11290\_f0 | denovo11394\_f0 | denovo11420\_f0 | denovo11472\_f0 | denovo11498\_f0 | denovo11524\_f0 | denovo11576\_f0 | denovo11602\_f0 | denovo11706\_f0 | denovo11732\_f0 | denovo11758\_f0 | denovo11862\_f0 | denovo11888\_f0 | denovo11940\_f0 |
| GNV129007 | Urodus parvula\* | denovo15\_f0 | denovo67\_f0 | denovo93\_f0 | denovo171\_f0 | denovo197\_f0 | denovo275\_f0 | denovo327\_f0 | denovo379\_f0 | denovo457\_f0 | denovo561\_f0 | denovo691\_f0 | denovo847\_f0 | denovo873\_f0 | denovo899\_f0 | denovo925\_f0 | denovo1055\_f0 | denovo1185\_f0 | denovo1237\_f0 | denovo1263\_f0 | denovo1289\_f0 | denovo1367\_f0 | denovo1393\_f0 | denovo1419\_f0 | denovo1497\_f0 | denovo1575\_f0 | denovo1653\_f0 | denovo1679\_f0 | denovo1731\_f0 | denovo1861\_f0 | denovo1913\_f0 | denovo1939\_f0 | denovo1965\_f0 | denovo1991\_f0 | denovo2017\_f0 | denovo2069\_f0 | denovo2095\_f0 | denovo2121\_f0 | denovo2147\_f0 | denovo2251\_f0 | denovo2277\_f0 | denovo2303\_f0 | denovo2329\_f0 | denovo2381\_f0 | denovo2407\_f0 | denovo2433\_f0 | denovo2563\_f0 | denovo2589\_f0 | denovo2615\_f0 | denovo2641\_f0 | denovo2745\_f0 | denovo2771\_f0 | denovo2823\_f0 | denovo2901\_f0 | denovo3317\_f0 | denovo3343\_f0 | denovo3447\_f0 | denovo3603\_f0 | denovo3629\_f0 | denovo3655\_f0 | denovo3733\_f0 | denovo3759\_f0 | denovo3811\_f0 | denovo3837\_f0 | denovo3889\_f0 | denovo4071\_f0 | denovo4097\_f0 | denovo4149\_f0 | denovo4175\_f0 | denovo4253\_f0 | denovo4331\_f0 | denovo4357\_f0 | denovo4383\_f0 | denovo4435\_f0 | denovo4513\_f0 | denovo4539\_f0 | denovo4643\_f0 | denovo4851\_f0 | denovo5007\_f0 | denovo5033\_f0 | denovo5111\_f0 | denovo5137\_f0 | denovo5163\_f0 | denovo5345\_f0 | denovo5423\_f0 | denovo5501\_f0 | denovo5553\_f0 | denovo5579\_f0 | denovo5735\_f0 | denovo5787\_f0 | denovo5865\_f0 | denovo5891\_f0 | denovo5917\_f0 | denovo5969\_f0 | denovo6047\_f0 | denovo6099\_f0 | denovo6203\_f0 | denovo6281\_f0 | denovo6307\_f0 | denovo6333\_f0 | denovo6359\_f0 | denovo6385\_f0 | denovo6489\_f0 | denovo6515\_f0 | denovo6541\_f0 | denovo6567\_f0 | denovo6645\_f0 | denovo6671\_f0 | denovo6723\_f0 | denovo6749\_f0 | denovo6905\_f0 | denovo6983\_f0 | denovo7035\_f0 | denovo7061\_f0 | denovo7139\_f0 | denovo7165\_f0 | denovo7295\_f0 | denovo7321\_f0 | denovo7347\_f0 | denovo7373\_f0 | denovo7399\_f0 | denovo7451\_f0 | denovo7477\_f0 | denovo7503\_f0 | denovo7555\_f0 | denovo7633\_f0 | denovo7659\_f0 | denovo7711\_f0 | denovo7789\_f0 | denovo7841\_f0 | denovo7867\_f0 | denovo7919\_f0 | denovo7945\_f0 | denovo8023\_f0 | denovo8049\_f0 | denovo8101\_f0 | denovo8153\_f0 | denovo8205\_f0 | denovo8361\_f0 | denovo8387\_f0 | denovo8413\_f0 | denovo8491\_f0 | denovo8543\_f0 | denovo8569\_f0 | denovo8621\_f0 | denovo8647\_f0 | denovo8699\_f0 | denovo8725\_f0 | denovo8751\_f0 | denovo8777\_f0 | denovo9063\_f0 | denovo9089\_f0 | denovo9115\_f0 | denovo9141\_f0 | denovo9219\_f0 | denovo9245\_f0 | denovo9297\_f0 | denovo9349\_f0 | denovo9479\_f0 | denovo9505\_f0 | denovo9635\_f0 | denovo9661\_f0 | denovo9791\_f0 | denovo9843\_f0 | denovo9895\_f0 | denovo9973\_f0 | denovo10025\_f0 | denovo10051\_f0 | denovo10129\_f0 | denovo10155\_f0 | denovo10285\_f0 | denovo10311\_f0 | denovo10441\_f0 | denovo10467\_f0 | denovo10519\_f0 | denovo10545\_f0 | denovo10571\_f0 | denovo10597\_f0 | denovo10727\_f0 | denovo10753\_f0 | denovo10779\_f0 | denovo10831\_f0 | denovo10909\_f0 | denovo11013\_f0 | denovo11065\_f0 | denovo11143\_f0 | denovo11221\_f0 | denovo11299\_f0 | denovo11403\_f0 | denovo11429\_f0 | denovo11481\_f0 | denovo11507\_f0 | denovo11533\_f0 | denovo11585\_f0 | denovo11611\_f0 | denovo11715\_f0 | denovo11741\_f0 | denovo11767\_f0 | denovo11871\_f0 | denovo11897\_f0 | denovo11949\_f0 |
| SW130126 | Lyssa zampa\* | denovo24\_f0 | denovo76\_f0 | denovo102\_f0 | denovo180\_f0 | denovo206\_f0 | denovo284\_f0 | denovo336\_f0 | denovo388\_f0 | denovo466\_f0 | denovo570\_f0 | denovo700\_f0 | denovo856\_f0 | denovo882\_f0 | denovo908\_f0 | denovo934\_f0 | denovo1064\_f0 | denovo1194\_f0 | denovo1246\_f0 | denovo1272\_f0 | denovo1298\_f0 | denovo1376\_f0 | denovo1402\_f0 | denovo1428\_f0 | denovo1506\_f0 | denovo1584\_f0 | denovo1662\_f0 | denovo1688\_f0 | denovo1740\_f0 | denovo1870\_f0 | denovo1922\_f0 | denovo1948\_f0 | denovo1974\_f0 | denovo2000\_f0 | denovo2026\_f0 | denovo2078\_f0 | denovo2104\_f0 | denovo2130\_f0 | denovo2156\_f0 | denovo2260\_f0 | denovo2286\_f0 | denovo2312\_f0 | denovo2338\_f0 | denovo2390\_f0 | denovo2416\_f0 | denovo2442\_f0 | denovo2572\_f0 | denovo2598\_f0 | denovo2624\_f0 | denovo2650\_f0 | denovo2754\_f0 | denovo2780\_f0 | denovo2832\_f0 | denovo2910\_f0 | denovo3326\_f0 | denovo3352\_f0 | denovo3456\_f0 | denovo3612\_f0 | denovo3638\_f0 | denovo3664\_f0 | denovo3742\_f0 | denovo3768\_f0 | denovo3820\_f0 | denovo3846\_f0 | denovo3898\_f0 | denovo4080\_f0 | denovo4106\_f0 | denovo4158\_f0 | denovo4184\_f0 | denovo4262\_f0 | denovo4340\_f0 | denovo4366\_f0 | denovo4392\_f0 | denovo4444\_f0 | denovo4522\_f0 | denovo4548\_f0 | denovo4652\_f0 | denovo4860\_f0 | denovo5016\_f0 | denovo5042\_f0 | denovo5120\_f0 | denovo5146\_f0 | denovo5172\_f0 | denovo5354\_f0 | denovo5432\_f0 | denovo5510\_f0 | denovo5562\_f0 | denovo5588\_f0 | denovo5744\_f0 | denovo5796\_f0 | denovo5874\_f0 | denovo5900\_f0 | denovo5926\_f0 | denovo5978\_f0 | denovo6056\_f0 | denovo6108\_f0 | denovo6212\_f0 | denovo6290\_f0 | denovo6316\_f0 | denovo6342\_f0 | denovo6368\_f0 | denovo6394\_f0 | denovo6498\_f0 | denovo6524\_f0 | denovo6550\_f0 | denovo6576\_f0 | denovo6654\_f0 | denovo6680\_f0 | denovo6732\_f0 | denovo6758\_f0 | denovo6914\_f0 | denovo6992\_f0 | denovo7044\_f0 | denovo7070\_f0 | denovo7148\_f0 | denovo7174\_f0 | denovo7304\_f0 | denovo7330\_f0 | denovo7356\_f0 | denovo7382\_f0 | denovo7408\_f0 | denovo7460\_f0 | denovo7486\_f0 | denovo7512\_f0 | denovo7564\_f0 | denovo7642\_f0 | denovo7668\_f0 | denovo7720\_f0 | denovo7798\_f0 | denovo7850\_f0 | denovo7876\_f0 | denovo7928\_f0 | denovo7954\_f0 | denovo8032\_f0 | denovo8058\_f0 | denovo8110\_f0 | denovo8162\_f0 | denovo8214\_f0 | denovo8370\_f0 | denovo8396\_f0 | denovo8422\_f0 | denovo8500\_f0 | denovo8552\_f0 | denovo8578\_f0 | denovo8630\_f0 | denovo8656\_f0 | denovo8708\_f0 | denovo8734\_f0 | denovo8760\_f0 | denovo8786\_f0 | denovo9072\_f0 | denovo9098\_f0 | denovo9124\_f0 | denovo9150\_f0 | denovo9228\_f0 | denovo9254\_f0 | denovo9306\_f0 | denovo9358\_f0 | denovo9488\_f0 | denovo9514\_f0 | denovo9644\_f0 | denovo9670\_f0 | denovo9800\_f0 | denovo9852\_f0 | denovo9904\_f0 | denovo9982\_f0 | denovo10034\_f0 | denovo10060\_f0 | denovo10138\_f0 | denovo10164\_f0 | denovo10294\_f0 | denovo10320\_f0 | denovo10450\_f0 | denovo10476\_f0 | denovo10528\_f0 | denovo10554\_f0 | denovo10580\_f0 | denovo10606\_f0 | denovo10736\_f0 | denovo10762\_f0 | denovo10788\_f0 | denovo10840\_f0 | denovo10918\_f0 | denovo11022\_f0 | denovo11074\_f0 | denovo11152\_f0 | denovo11230\_f0 | denovo11308\_f0 | denovo11412\_f0 | denovo11438\_f0 | denovo11490\_f0 | denovo11516\_f0 | denovo11542\_f0 | denovo11594\_f0 | denovo11620\_f0 | denovo11724\_f0 | denovo11750\_f0 | denovo11776\_f0 | denovo11880\_f0 | denovo11906\_f0 | denovo11958\_f0 |
| SW130103 | Anigraea sp. | denovo23\_f0 | denovo75\_f0 | denovo101\_f0 | denovo179\_f0 | denovo205\_f0 | denovo283\_f0 | denovo335\_f0 | denovo387\_f0 | denovo465\_f0 | denovo569\_f0 | denovo699\_f0 | denovo855\_f0 | denovo881\_f0 | denovo907\_f0 | denovo933\_f0 | denovo1063\_f0 | denovo1193\_f0 | denovo1245\_f0 | denovo1271\_f0 | denovo1297\_f0 | denovo1375\_f0 | denovo1401\_f0 | denovo1427\_f0 | denovo1505\_f0 | denovo1583\_f0 | denovo1661\_f0 | denovo1687\_f0 | denovo1739\_f0 | denovo1869\_f0 | denovo1921\_f0 | denovo1947\_f0 | denovo1973\_f0 | denovo1999\_f0 | denovo2025\_f0 | denovo2077\_f0 | denovo2103\_f0 | denovo2129\_f0 | denovo2155\_f0 | denovo2259\_f0 | denovo2285\_f0 | denovo2311\_f0 | denovo2337\_f0 | denovo2389\_f0 | denovo2415\_f0 | denovo2441\_f0 | denovo2571\_f0 | denovo2597\_f0 | denovo2623\_f0 | denovo2649\_f0 | denovo2753\_f0 | denovo2779\_f0 | denovo2831\_f0 | denovo2909\_f0 | denovo3325\_f0 | denovo3351\_f0 | denovo3455\_f0 | denovo3611\_f0 | denovo3637\_f0 | denovo3663\_f0 | denovo3741\_f0 | denovo3767\_f0 | denovo3819\_f0 | denovo3845\_f0 | denovo3897\_f0 | denovo4079\_f0 | denovo4105\_f0 | denovo4157\_f0 | denovo4183\_f0 | denovo4261\_f0 | denovo4339\_f0 | denovo4365\_f0 | denovo4391\_f0 | denovo4443\_f0 | denovo4521\_f0 | denovo4547\_f0 | denovo4651\_f0 | denovo4859\_f0 | denovo5015\_f0 | denovo5041\_f0 | denovo5119\_f0 | denovo5145\_f0 | denovo5171\_f0 | denovo5353\_f0 | denovo5431\_f0 | denovo5509\_f0 | denovo5561\_f0 | denovo5587\_f0 | denovo5743\_f0 | denovo5795\_f0 | denovo5873\_f0 | denovo5899\_f0 | denovo5925\_f0 | denovo5977\_f0 | denovo6055\_f0 | denovo6107\_f0 | denovo6211\_f0 | denovo6289\_f0 | denovo6315\_f0 | denovo6341\_f0 | denovo6367\_f0 | denovo6393\_f0 | denovo6497\_f0 | denovo6523\_f0 | denovo6549\_f0 | denovo6575\_f0 | denovo6653\_f0 | denovo6679\_f0 | denovo6731\_f0 | denovo6757\_f0 | denovo6913\_f0 | denovo6991\_f0 | denovo7043\_f0 | denovo7069\_f0 | denovo7147\_f0 | denovo7173\_f0 | denovo7303\_f0 | denovo7329\_f0 | denovo7355\_f0 | denovo7381\_f0 | denovo7407\_f0 | denovo7459\_f0 | denovo7485\_f0 | denovo7511\_f0 | denovo7563\_f0 | denovo7641\_f0 | denovo7667\_f0 | denovo7719\_f0 | denovo7797\_f0 | denovo7849\_f0 | denovo7875\_f0 | denovo7927\_f0 | denovo7953\_f0 | denovo8031\_f0 | denovo8057\_f0 | denovo8109\_f0 | denovo8161\_f0 | denovo8213\_f0 | denovo8369\_f0 | denovo8395\_f0 | denovo8421\_f0 | denovo8499\_f0 | denovo8551\_f0 | denovo8577\_f0 | denovo8629\_f0 | denovo8655\_f0 | denovo8707\_f0 | denovo8733\_f0 | denovo8759\_f0 | denovo8785\_f0 | denovo9071\_f0 | denovo9097\_f0 | denovo9123\_f0 | denovo9149\_f0 | denovo9227\_f0 | denovo9253\_f0 | denovo9305\_f0 | denovo9357\_f0 | denovo9487\_f0 | denovo9513\_f0 | denovo9643\_f0 | denovo9669\_f0 | denovo9799\_f0 | denovo9851\_f0 | denovo9903\_f0 | denovo9981\_f0 | denovo10033\_f0 | denovo10059\_f0 | denovo10137\_f0 | denovo10163\_f0 | denovo10293\_f0 | denovo10319\_f0 | denovo10449\_f0 | denovo10475\_f0 | denovo10527\_f0 | denovo10553\_f0 | denovo10579\_f0 | denovo10605\_f0 | denovo10735\_f0 | denovo10761\_f0 | denovo10787\_f0 | denovo10839\_f0 | denovo10917\_f0 | denovo11021\_f0 | denovo11073\_f0 | denovo11151\_f0 | denovo11229\_f0 | denovo11307\_f0 | denovo11411\_f0 | denovo11437\_f0 | denovo11489\_f0 | denovo11515\_f0 | denovo11541\_f0 | denovo11593\_f0 | denovo11619\_f0 | denovo11723\_f0 | denovo11749\_f0 | denovo11775\_f0 | denovo11879\_f0 | denovo11905\_f0 | denovo11957\_f0 |
| Callid | Pterodecta felderi\* | denovo1\_f0 | denovo53\_f0 | denovo79\_f0 | denovo157\_f0 | denovo183\_f0 | denovo261\_f0 | denovo313\_f0 | denovo365\_f0 | denovo443\_f0 | denovo547\_f0 | denovo677\_f0 | denovo833\_f0 | denovo859\_f0 | denovo885\_f0 | denovo911\_f0 | denovo1041\_f0 | denovo1171\_f0 | denovo1223\_f0 | denovo1249\_f0 | denovo1275\_f0 | denovo1353\_f0 | denovo1379\_f0 | denovo1405\_f0 | denovo1483\_f0 | denovo1561\_f0 | denovo1639\_f0 | denovo1665\_f0 | denovo1717\_f0 | denovo1847\_f0 | denovo1899\_f0 | denovo1925\_f0 | denovo1951\_f0 | denovo1977\_f0 | denovo2003\_f0 | denovo2055\_f0 | denovo2081\_f0 | denovo2107\_f0 | denovo2133\_f0 | denovo2237\_f0 | denovo2263\_f0 | denovo2289\_f0 | denovo2315\_f0 | denovo2367\_f0 | denovo2393\_f0 | denovo2419\_f0 | denovo2549\_f0 | denovo2575\_f0 | denovo2601\_f0 | denovo2627\_f0 | denovo2731\_f0 | denovo2757\_f0 | denovo2809\_f0 | denovo2887\_f0 | denovo3303\_f0 | denovo3329\_f0 | denovo3433\_f0 | denovo3589\_f0 | denovo3615\_f0 | denovo3641\_f0 | denovo3719\_f0 | denovo3745\_f0 | denovo3797\_f0 | denovo3823\_f0 | denovo3875\_f0 | denovo4057\_f0 | denovo4083\_f0 | denovo4135\_f0 | denovo4161\_f0 | denovo4239\_f0 | denovo4317\_f0 | denovo4343\_f0 | denovo4369\_f0 | denovo4421\_f0 | denovo4499\_f0 | denovo4525\_f0 | denovo4629\_f0 | denovo4837\_f0 | denovo4993\_f0 | denovo5019\_f0 | denovo5097\_f0 | denovo5123\_f0 | denovo5149\_f0 | denovo5331\_f0 | denovo5409\_f0 | denovo5487\_f0 | denovo5539\_f0 | denovo5565\_f0 | denovo5721\_f0 | denovo5773\_f0 | denovo5851\_f0 | denovo5877\_f0 | denovo5903\_f0 | denovo5955\_f0 | denovo6033\_f0 | denovo6085\_f0 | denovo6189\_f0 | denovo6267\_f0 | denovo6293\_f0 | denovo6319\_f0 | denovo6345\_f0 | denovo6371\_f0 | denovo6475\_f0 | denovo6501\_f0 | denovo6527\_f0 | denovo6553\_f0 | denovo6631\_f0 | denovo6657\_f0 | denovo6709\_f0 | denovo6735\_f0 | denovo6891\_f0 | denovo6969\_f0 | denovo7021\_f0 | denovo7047\_f0 | denovo7125\_f0 | denovo7151\_f0 | denovo7281\_f0 | denovo7307\_f0 | denovo7333\_f0 | denovo7359\_f0 | denovo7385\_f0 | denovo7437\_f0 | denovo7463\_f0 | denovo7489\_f0 | denovo7541\_f0 | denovo7619\_f0 | denovo7645\_f0 | denovo7697\_f0 | denovo7775\_f0 | denovo7827\_f0 | denovo7853\_f0 | denovo7905\_f0 | denovo7931\_f0 | denovo8009\_f0 | denovo8035\_f0 | denovo8087\_f0 | denovo8139\_f0 | denovo8191\_f0 | denovo8347\_f0 | denovo8373\_f0 | denovo8399\_f0 | denovo8477\_f0 | denovo8529\_f0 | denovo8555\_f0 | denovo8607\_f0 | denovo8633\_f0 | denovo8685\_f0 | denovo8711\_f0 | denovo8737\_f0 | denovo8763\_f0 | denovo9049\_f0 | denovo9075\_f0 | denovo9101\_f0 | denovo9127\_f0 | denovo9205\_f0 | denovo9231\_f0 | denovo9283\_f0 | denovo9335\_f0 | denovo9465\_f0 | denovo9491\_f0 | denovo9621\_f0 | denovo9647\_f0 | denovo9777\_f0 | denovo9829\_f0 | denovo9881\_f0 | denovo9959\_f0 | denovo10011\_f0 | denovo10037\_f0 | denovo10115\_f0 | denovo10141\_f0 | denovo10271\_f0 | denovo10297\_f0 | denovo10427\_f0 | denovo10453\_f0 | denovo10505\_f0 | denovo10531\_f0 | denovo10557\_f0 | denovo10583\_f0 | denovo10713\_f0 | denovo10739\_f0 | denovo10765\_f0 | denovo10817\_f0 | denovo10895\_f0 | denovo10999\_f0 | denovo11051\_f0 | denovo11129\_f0 | denovo11207\_f0 | denovo11285\_f0 | denovo11389\_f0 | denovo11415\_f0 | denovo11467\_f0 | denovo11493\_f0 | denovo11519\_f0 | denovo11571\_f0 | denovo11597\_f0 | denovo11701\_f0 | denovo11727\_f0 | denovo11753\_f0 | denovo11857\_f0 | denovo11883\_f0 | denovo11935\_f0 |
| FG120070B | Artace sp.\* | denovo8\_f0 | denovo60\_f0 | denovo86\_f0 | denovo164\_f0 | denovo190\_f0 | denovo268\_f0 | denovo320\_f0 | denovo372\_f0 | denovo450\_f0 | denovo554\_f0 | denovo684\_f0 | denovo840\_f0 | denovo866\_f0 | denovo892\_f0 | denovo918\_f0 | denovo1048\_f0 | denovo1178\_f0 | denovo1230\_f0 | denovo1256\_f0 | denovo1282\_f0 | denovo1360\_f0 | denovo1386\_f0 | denovo1412\_f0 | denovo1490\_f0 | denovo1568\_f0 | denovo1646\_f0 | denovo1672\_f0 | denovo1724\_f0 | denovo1854\_f0 | denovo1906\_f0 | denovo1932\_f0 | denovo1958\_f0 | denovo1984\_f0 | denovo2010\_f0 | denovo2062\_f0 | denovo2088\_f0 | denovo2114\_f0 | denovo2140\_f0 | denovo2244\_f0 | denovo2270\_f0 | denovo2296\_f0 | denovo2322\_f0 | denovo2374\_f0 | denovo2400\_f0 | denovo2426\_f0 | denovo2556\_f0 | denovo2582\_f0 | denovo2608\_f0 | denovo2634\_f0 | denovo2738\_f0 | denovo2764\_f0 | denovo2816\_f0 | denovo2894\_f0 | denovo3310\_f0 | denovo3336\_f0 | denovo3440\_f0 | denovo3596\_f0 | denovo3622\_f0 | denovo3648\_f0 | denovo3726\_f0 | denovo3752\_f0 | denovo3804\_f0 | denovo3830\_f0 | denovo3882\_f0 | denovo4064\_f0 | denovo4090\_f0 | denovo4142\_f0 | denovo4168\_f0 | denovo4246\_f0 | denovo4324\_f0 | denovo4350\_f0 | denovo4376\_f0 | denovo4428\_f0 | denovo4506\_f0 | denovo4532\_f0 | denovo4636\_f0 | denovo4844\_f0 | denovo5000\_f0 | denovo5026\_f0 | denovo5104\_f0 | denovo5130\_f0 | denovo5156\_f0 | denovo5338\_f0 | denovo5416\_f0 | denovo5494\_f0 | denovo5546\_f0 | denovo5572\_f0 | denovo5728\_f0 | denovo5780\_f0 | denovo5858\_f0 | denovo5884\_f0 | denovo5910\_f0 | denovo5962\_f0 | denovo6040\_f0 | denovo6092\_f0 | denovo6196\_f0 | denovo6274\_f0 | denovo6300\_f0 | denovo6326\_f0 | denovo6352\_f0 | denovo6378\_f0 | denovo6482\_f0 | denovo6508\_f0 | denovo6534\_f0 | denovo6560\_f0 | denovo6638\_f0 | denovo6664\_f0 | denovo6716\_f0 | denovo6742\_f0 | denovo6898\_f0 | denovo6976\_f0 | denovo7028\_f0 | denovo7054\_f0 | denovo7132\_f0 | denovo7158\_f0 | denovo7288\_f0 | denovo7314\_f0 | denovo7340\_f0 | denovo7366\_f0 | denovo7392\_f0 | denovo7444\_f0 | denovo7470\_f0 | denovo7496\_f0 | denovo7548\_f0 | denovo7626\_f0 | denovo7652\_f0 | denovo7704\_f0 | denovo7782\_f0 | denovo7834\_f0 | denovo7860\_f0 | denovo7912\_f0 | denovo7938\_f0 | denovo8016\_f0 | denovo8042\_f0 | denovo8094\_f0 | denovo8146\_f0 | denovo8198\_f0 | denovo8354\_f0 | denovo8380\_f0 | denovo8406\_f0 | denovo8484\_f0 | denovo8536\_f0 | denovo8562\_f0 | denovo8614\_f0 | denovo8640\_f0 | denovo8692\_f0 | denovo8718\_f0 | denovo8744\_f0 | denovo8770\_f0 | denovo9056\_f0 | denovo9082\_f0 | denovo9108\_f0 | denovo9134\_f0 | denovo9212\_f0 | denovo9238\_f0 | denovo9290\_f0 | denovo9342\_f0 | denovo9472\_f0 | denovo9498\_f0 | denovo9628\_f0 | denovo9654\_f0 | denovo9784\_f0 | denovo9836\_f0 | denovo9888\_f0 | denovo9966\_f0 | denovo10018\_f0 | denovo10044\_f0 | denovo10122\_f0 | denovo10148\_f0 | denovo10278\_f0 | denovo10304\_f0 | denovo10434\_f0 | denovo10460\_f0 | denovo10512\_f0 | denovo10538\_f0 | denovo10564\_f0 | denovo10590\_f0 | denovo10720\_f0 | denovo10746\_f0 | denovo10772\_f0 | denovo10824\_f0 | denovo10902\_f0 | denovo11006\_f0 | denovo11058\_f0 | denovo11136\_f0 | denovo11214\_f0 | denovo11292\_f0 | denovo11396\_f0 | denovo11422\_f0 | denovo11474\_f0 | denovo11500\_f0 | denovo11526\_f0 | denovo11578\_f0 | denovo11604\_f0 | denovo11708\_f0 | denovo11734\_f0 | denovo11760\_f0 | denovo11864\_f0 | denovo11890\_f0 | denovo11942\_f0 |
| SW130007 | Thubana sp.\* | denovo22\_f0 | denovo74\_f0 | denovo100\_f0 | denovo178\_f0 | denovo204\_f0 | denovo282\_f0 | denovo334\_f0 | denovo386\_f0 | denovo464\_f0 | denovo568\_f0 | denovo698\_f0 | denovo854\_f0 | denovo880\_f0 | denovo906\_f0 | denovo932\_f0 | denovo1062\_f0 | denovo1192\_f0 | denovo1244\_f0 | denovo1270\_f0 | denovo1296\_f0 | denovo1374\_f0 | denovo1400\_f0 | denovo1426\_f0 | denovo1504\_f0 | denovo1582\_f0 | denovo1660\_f0 | denovo1686\_f0 | denovo1738\_f0 | denovo1868\_f0 | denovo1920\_f0 | denovo1946\_f0 | denovo1972\_f0 | denovo1998\_f0 | denovo2024\_f0 | denovo2076\_f0 | denovo2102\_f0 | denovo2128\_f0 | denovo2154\_f0 | denovo2258\_f0 | denovo2284\_f0 | denovo2310\_f0 | denovo2336\_f0 | denovo2388\_f0 | denovo2414\_f0 | denovo2440\_f0 | denovo2570\_f0 | denovo2596\_f0 | denovo2622\_f0 | denovo2648\_f0 | denovo2752\_f0 | denovo2778\_f0 | denovo2830\_f0 | denovo2908\_f0 | denovo3324\_f0 | denovo3350\_f0 | denovo3454\_f0 | denovo3610\_f0 | denovo3636\_f0 | denovo3662\_f0 | denovo3740\_f0 | denovo3766\_f0 | denovo3818\_f0 | denovo3844\_f0 | denovo3896\_f0 | denovo4078\_f0 | denovo4104\_f0 | denovo4156\_f0 | denovo4182\_f0 | denovo4260\_f0 | denovo4338\_f0 | denovo4364\_f0 | denovo4390\_f0 | denovo4442\_f0 | denovo4520\_f0 | denovo4546\_f0 | denovo4650\_f0 | denovo4858\_f0 | denovo5014\_f0 | denovo5040\_f0 | denovo5118\_f0 | denovo5144\_f0 | denovo5170\_f0 | denovo5352\_f0 | denovo5430\_f0 | denovo5508\_f0 | denovo5560\_f0 | denovo5586\_f0 | denovo5742\_f0 | denovo5794\_f0 | denovo5872\_f0 | denovo5898\_f0 | denovo5924\_f0 | denovo5976\_f0 | denovo6054\_f0 | denovo6106\_f0 | denovo6210\_f0 | denovo6288\_f0 | denovo6314\_f0 | denovo6340\_f0 | denovo6366\_f0 | denovo6392\_f0 | denovo6496\_f0 | denovo6522\_f0 | denovo6548\_f0 | denovo6574\_f0 | denovo6652\_f0 | denovo6678\_f0 | denovo6730\_f0 | denovo6756\_f0 | denovo6912\_f0 | denovo6990\_f0 | denovo7042\_f0 | denovo7068\_f0 | denovo7146\_f0 | denovo7172\_f0 | denovo7302\_f0 | denovo7328\_f0 | denovo7354\_f0 | denovo7380\_f0 | denovo7406\_f0 | denovo7458\_f0 | denovo7484\_f0 | denovo7510\_f0 | denovo7562\_f0 | denovo7640\_f0 | denovo7666\_f0 | denovo7718\_f0 | denovo7796\_f0 | denovo7848\_f0 | denovo7874\_f0 | denovo7926\_f0 | denovo7952\_f0 | denovo8030\_f0 | denovo8056\_f0 | denovo8108\_f0 | denovo8160\_f0 | denovo8212\_f0 | denovo8368\_f0 | denovo8394\_f0 | denovo8420\_f0 | denovo8498\_f0 | denovo8550\_f0 | denovo8576\_f0 | denovo8628\_f0 | denovo8654\_f0 | denovo8706\_f0 | denovo8732\_f0 | denovo8758\_f0 | denovo8784\_f0 | denovo9070\_f0 | denovo9096\_f0 | denovo9122\_f0 | denovo9148\_f0 | denovo9226\_f0 | denovo9252\_f0 | denovo9304\_f0 | denovo9356\_f0 | denovo9486\_f0 | denovo9512\_f0 | denovo9642\_f0 | denovo9668\_f0 | denovo9798\_f0 | denovo9850\_f0 | denovo9902\_f0 | denovo9980\_f0 | denovo10032\_f0 | denovo10058\_f0 | denovo10136\_f0 | denovo10162\_f0 | denovo10292\_f0 | denovo10318\_f0 | denovo10448\_f0 | denovo10474\_f0 | denovo10526\_f0 | denovo10552\_f0 | denovo10578\_f0 | denovo10604\_f0 | denovo10734\_f0 | denovo10760\_f0 | denovo10786\_f0 | denovo10838\_f0 | denovo10916\_f0 | denovo11020\_f0 | denovo11072\_f0 | denovo11150\_f0 | denovo11228\_f0 | denovo11306\_f0 | denovo11410\_f0 | denovo11436\_f0 | denovo11488\_f0 | denovo11514\_f0 | denovo11540\_f0 | denovo11592\_f0 | denovo11618\_f0 | denovo11722\_f0 | denovo11748\_f0 | denovo11774\_f0 | denovo11878\_f0 | denovo11904\_f0 | denovo11956\_f0 |
| GNV120032 | Nemoria lixaria\* | denovo14\_f0 | denovo66\_f0 | denovo92\_f0 | denovo170\_f0 | denovo196\_f0 | denovo274\_f0 | denovo326\_f0 | denovo378\_f0 | denovo456\_f0 | denovo560\_f0 | denovo690\_f0 | denovo846\_f0 | denovo872\_f0 | denovo898\_f0 | denovo924\_f0 | denovo1054\_f0 | denovo1184\_f0 | denovo1236\_f0 | denovo1262\_f0 | denovo1288\_f0 | denovo1366\_f0 | denovo1392\_f0 | denovo1418\_f0 | denovo1496\_f0 | denovo1574\_f0 | denovo1652\_f0 | denovo1678\_f0 | denovo1730\_f0 | denovo1860\_f0 | denovo1912\_f0 | denovo1938\_f0 | denovo1964\_f0 | denovo1990\_f0 | denovo2016\_f0 | denovo2068\_f0 | denovo2094\_f0 | denovo2120\_f0 | denovo2146\_f0 | denovo2250\_f0 | denovo2276\_f0 | denovo2302\_f0 | denovo2328\_f0 | denovo2380\_f0 | denovo2406\_f0 | denovo2432\_f0 | denovo2562\_f0 | denovo2588\_f0 | denovo2614\_f0 | denovo2640\_f0 | denovo2744\_f0 | denovo2770\_f0 | denovo2822\_f0 | denovo2900\_f0 | denovo3316\_f0 | denovo3342\_f0 | denovo3446\_f0 | denovo3602\_f0 | denovo3628\_f0 | denovo3654\_f0 | denovo3732\_f0 | denovo3758\_f0 | denovo3810\_f0 | denovo3836\_f0 | denovo3888\_f0 | denovo4070\_f0 | denovo4096\_f0 | denovo4148\_f0 | denovo4174\_f0 | denovo4252\_f0 | denovo4330\_f0 | denovo4356\_f0 | denovo4382\_f0 | denovo4434\_f0 | denovo4512\_f0 | denovo4538\_f0 | denovo4642\_f0 | denovo4850\_f0 | denovo5006\_f0 | denovo5032\_f0 | denovo5110\_f0 | denovo5136\_f0 | denovo5162\_f0 | denovo5344\_f0 | denovo5422\_f0 | denovo5500\_f0 | denovo5552\_f0 | denovo5578\_f0 | denovo5734\_f0 | denovo5786\_f0 | denovo5864\_f0 | denovo5890\_f0 | denovo5916\_f0 | denovo5968\_f0 | denovo6046\_f0 | denovo6098\_f0 | denovo6202\_f0 | denovo6280\_f0 | denovo6306\_f0 | denovo6332\_f0 | denovo6358\_f0 | denovo6384\_f0 | denovo6488\_f0 | denovo6514\_f0 | denovo6540\_f0 | denovo6566\_f0 | denovo6644\_f0 | denovo6670\_f0 | denovo6722\_f0 | denovo6748\_f0 | denovo6904\_f0 | denovo6982\_f0 | denovo7034\_f0 | denovo7060\_f0 | denovo7138\_f0 | denovo7164\_f0 | denovo7294\_f0 | denovo7320\_f0 | denovo7346\_f0 | denovo7372\_f0 | denovo7398\_f0 | denovo7450\_f0 | denovo7476\_f0 | denovo7502\_f0 | denovo7554\_f0 | denovo7632\_f0 | denovo7658\_f0 | denovo7710\_f0 | denovo7788\_f0 | denovo7840\_f0 | denovo7866\_f0 | denovo7918\_f0 | denovo7944\_f0 | denovo8022\_f0 | denovo8048\_f0 | denovo8100\_f0 | denovo8152\_f0 | denovo8204\_f0 | denovo8360\_f0 | denovo8386\_f0 | denovo8412\_f0 | denovo8490\_f0 | denovo8542\_f0 | denovo8568\_f0 | denovo8620\_f0 | denovo8646\_f0 | denovo8698\_f0 | denovo8724\_f0 | denovo8750\_f0 | denovo8776\_f0 | denovo9062\_f0 | denovo9088\_f0 | denovo9114\_f0 | denovo9140\_f0 | denovo9218\_f0 | denovo9244\_f0 | denovo9296\_f0 | denovo9348\_f0 | denovo9478\_f0 | denovo9504\_f0 | denovo9634\_f0 | denovo9660\_f0 | denovo9790\_f0 | denovo9842\_f0 | denovo9894\_f0 | denovo9972\_f0 | denovo10024\_f0 | denovo10050\_f0 | denovo10128\_f0 | denovo10154\_f0 | denovo10284\_f0 | denovo10310\_f0 | denovo10440\_f0 | denovo10466\_f0 | denovo10518\_f0 | denovo10544\_f0 | denovo10570\_f0 | denovo10596\_f0 | denovo10726\_f0 | denovo10752\_f0 | denovo10778\_f0 | denovo10830\_f0 | denovo10908\_f0 | denovo11012\_f0 | denovo11064\_f0 | denovo11142\_f0 | denovo11220\_f0 | denovo11298\_f0 | denovo11402\_f0 | denovo11428\_f0 | denovo11480\_f0 | denovo11506\_f0 | denovo11532\_f0 | denovo11584\_f0 | denovo11610\_f0 | denovo11714\_f0 | denovo11740\_f0 | denovo11766\_f0 | denovo11870\_f0 | denovo11896\_f0 | denovo11948\_f0 |
[truncated: 531,760 more chars]
