# Supplementary material for: ReproPhylo: An Environment for Reproducible Phylogenomics
Source: PLoS Comput Biol. 2015 Sep 3;11(9):e1004447. doi: 10.1371/journal.pcbi.1004447 (PMC4559436; doi:10.1371/journal.pcbi.1004447)
Supplement: S1 Example — (HTML) [file pcbi.1004447.s005.html]

Git\_Demo


# Git and Pickle integration in ReproPhylo¶

This notebook demonstrates the interaction of ReproPhylo and of pickled ReproPhylo `Project` files with Git.

## The short version¶

### Start a Project, read data, do alignment, show Git log¶

**start a `Project`**

In [ ]:

```
pj = Project('git_demo_files/loci_edited.csv', pickle='git_demo_files/git_demo')
```

**Read data**

In [ ]:

```
pj.read_embl_genbank([genbank])
```

**Do alignment**

In [ ]:

```
pj.extract_by_locus()
mafft = AlnConf(pj)
pj.align([mafft])
```

**Show last Git action (which was to commit the pickle with the alignment)**

In [ ]:

```
pj.last_git_log()
```

### Revert to older `Project` version¶

**Show Git commits**

In [ ]:

```
pj.show_commits()
```

**Revert the `Project` pickle to undo last or more actions**  
Using a hash (commit id) taken from the output of `pj.show_commits()`

In [ ]:

```
pj = revert_pickle(pj, '22c27d5a25710ec78')
```

The newer version is not lost, you can toggle back to it the same way

### Unifying older and newer version¶

**Do another alignment without changing its name from the default, by misake**

In [ ]:

```
AlnConf(pj, cline_args=dict(localpair=True, maxiterate=1000))
pj.align([AlnConf])
```

**Realize your mistake, back up the new alignment and fix it's name**

In [ ]:

```
new_aln_ob = pj.alignments['mafftDefault']
new_used_method = pj.used_methods['mafftDefault']
new_used_method.method_name = 'SomeNewName'
```

**Revert to get a `Project` with the original alignment, named `'mafftDefault'`**

In [ ]:

```
pj = revert_pickle(pj, 'some hash')
```

**Add the new alignmnet alongside the old one, in the reverted `Project`**

In [ ]:

```
pj.alignments['SomeNewName'] = new_aln_ob
pj.used_methods['SomeNewName'] = new_used_method
```

Now you have a `Project` with both `'mafftDefault'` and `'SomeNewName'`, in both `pj.alignments` and in `pj.used_methods`

## The long version¶

The first step here is loading ReproPhylo:

In [2]:

```
from reprophylo import *
```

This demo uses Tetillidae GenBank records stored in `Tetillidae.gb` and the MT-CO1 locus described in the `loci_edited.csv`. To find out more about them, see the Tetillidae use case.

In [2]:

```
genbank = 'git_demo_files/Tetillidae.gb'
loci_file = 'git_demo_files/loci_edited.csv'
```

### The interaction between Git and ReproPhylo¶

The first step initiates a `Project` instance configured for a CO1 partition, as instructed by the `loci_edited.csv` file. In addition, the `Project` will be saved as a pickle in the binary `git_demo_files/git_demo`.

In [3]:

```
pj = Project('git_demo_files/loci_edited.csv', pickle='git_demo_files/git_demo')
```

```
/home/amir/Dropbox/python_modules/rpgit.py:74: UserWarning: Thanks to Stack-Overflow users Shane Geiger and Billy Jin for the git wrappers code
  warnings.warn('Thanks to Stack-Overflow users Shane Geiger and Billy Jin for the git wrappers code')
/home/amir/Dropbox/python_modules/rpgit.py:90: UserWarning: A git repository was created in /home/amir/Dropbox/ReproPhylo/Tutorial_files/ms.
  warnings.warn('A git repository was created in %s.'%repoDir)
/home/amir/Dropbox/python_modules/reprophylo.py:245: UserWarning: The new repository is called git_demo_files/git_demo.
  warnings.warn('The new repository is called %s.'%open(cwd + '/.git/description', 'r').read().rstrip())
DEBUG:Cloud:Log file (/home/amir/.picloud/cloud.log) opened
```

The massages include a credit to the programmers upon which code the Git code in RerpoPhylo is based, the path to the `.git` directory containing the newly created Git repository, and the name of the new repository. ReproPhylo uses the name to confirm it matches the pickle file, to prevent mistakes if files are moved around. However, this test will *not* break if the pickle file is renamed.

Since the `Project` is set up, we can read the data from the `Tetillidae.gb` file. The file contains several genes but we read only CO1 CDSs. More on this step in the Tetillidae use case.

In [4]:

```
pj.read_embl_genbank([genbank])
```

When data is read, the file is commited to the Git repository. To confirm this, it is possible to print the Git log file with `print(pj.git_log)` or to print the last log entry:

In [5]:

```
pj.last_git_log()
```

```
Sun Apr 19 17:08:41 2015
STDOUT:
[master b54e47d] 1 genbank/embl data file(s) from Sun Apr 19 17:08:41 2015
 1 file changed, 14973 insertions(+)
 create mode 100644 git_demo_files/Tetillidae.gb

STDERR:None
>>>>
```

To keep going, we need to break the sequence pile according to loci. Again, this is explained in detail in other use cases:

In [6]:

```
pj.extract_by_locus()
```

To illustrate version control, this demo focuses on the sequence alignment step, but the complete pipeline is also described in other use cases.

In [7]:

```
mafft = AlnConf(pj)
```

```
mafft 700311429459726.66_CDS_proteins_MT-CO1.fasta
```

The command above created an `AlnConf` object. We can print it as a string to see the details of the analysis:

In [8]:

```
print str(mafft)
```

```
AlnConf named mafftDefault with ID 700311429459726.66
Loci: MT-CO1 
Created on: Sun Apr 19 17:08:46 2015
Commands:
MT-CO1: mafft 700311429459726.66_CDS_proteins_MT-CO1.fasta

Environment:
[This was not executed yet]
execution time:
[This was not executed yet]
```

In [9]:

```
pj.used_methods
```

Out[9]:

```
{}
```

Since this `AlnConf` object was not executed yet, its string representation does not include some of the info, such as the environment it was run in. Also, the used\_methods dictionary is empty, because this `AlnConf` was not used yet. The next command will make use of this `AlnConf` object by passing it to the `align` method:

In [10]:

```
pj.align([mafft])
```

Several things happened here. First, an aligned CO1 dataset was placed in the `alignments` dictionary:

In [11]:

```
pj.alignments
```

Out[11]:

```
{'MT-CO1@mafftDefault': <<class 'Bio.Align.MultipleSeqAlignment'> instance (92 records of length 1566, IUPACAmbiguousDNA()) at 7f18d6d763d0>}
```

Second, the pickle of the `Project` was updated with the change and commited to the repository:

In [12]:

```
print pj.last_git_log()
```

```
Sun Apr 19 17:08:52 2015
STDOUT:
[master 22c27d5] AlnConf named mafftDefault with ID 700311429459726.66 Loci: MT-CO1 Created on: Sun Apr 19 17:08:46 2015 Commands: MT-CO1: mafft 700311429459726.66_CDS_proteins_MT-CO1.fasta
 1 file changed, 0 insertions(+), 0 deletions(-)

STDERR:None
>>>>

None
```

Third, the `AlnConf` object was placed in the `used_methods` dictionary,

In [13]:

```
pj.used_methods.keys()
```

Out[13]:

```
['mafftDefault']
```

and the string representation of this `AlnConf` object now includes execution, program and reference information:

In [14]:

```
print pj.used_methods['mafftDefault']
```

```
AlnConf named mafftDefault with ID 700311429459726.66
Loci: MT-CO1 
Created on: Sun Apr 19 17:08:46 2015
Commands:
MT-CO1: mafft 700311429459726.66_CDS_proteins_MT-CO1.fasta

Environment:
Platform: Linux-3.13.0-40-generic-x86_64-with-Ubuntu-14.04-trusty
 Processor: x86_64
 Python build: defaultMar 22 2014 22:59:56
 Python compiler: GCC 4.8.2
 Python implementation: CPython
 Python version: 2.7.6
 ete2 version: 2.2rev1056
 biopython version: 1.64
 dendropy version: 3.12.0
 cloud version: 2.8.5
 User: amir-TECRA-W50-A
 Program and version: MAFFT v7.123b\nPal2Nal v14
 Program reference:Katoh
 Standley 2013 (Molecular Biology and Evolution 30:772-780) MAFFT multiple sequence alignment software version 7: improvements in performance and usability.\nMikita Suyama
 David Torrents
 and Peer Bork (2006) PAL2NAL: robust conversion of protein sequence alignments into the corresponding codon alignments.Nucleic Acids Res. 34
 W609-W612.
execution time:
1.52710604668


==============================
Core Methods section sentence:
==============================
The dataset(s) MT-CO1 were first aligned at the protein level using the program MAFFT v7.123b [1].
The resulting alignments served as guides to codon-align the DNA sequences using Pal2Nal v14 [2].

Reference:
[1]Katoh, Standley 2013 (Molecular Biology and Evolution 30:772-780) MAFFT multiple sequence alignment software version 7: improvements in performance and usability.
[2]Mikita Suyama, David Torrents, and Peer Bork (2006) PAL2NAL: robust conversion of protein sequence alignments into the corresponding codon alignments.Nucleic Acids Res. 34, W609-W612.
```

The string representation of the `AlnConf` object now also includes a skeleton of a Methods section sentence which can be copied into a manuscript and edited. This complete string representation will also appear in the final HTML report that ReproPhylo will produce.

### Recovering from unintentional changes¶

Now lets do something stupid: We will make a new `AlnConf` object, with different run parameters, but without changing the name of the `AlnConf` object, thus overwriting the previous one. For this alignment step, this is not the end of the world, since it is very quick. However, this will work the same for long analyses, such as tree reconstruction or when there is a lot of data.

In [15]:

```
new_mafft = AlnConf(pj, cline_args=dict(localpair=True, maxiterate=1000))
pj.align([new_mafft])
```

```
mafft --localpair --maxiterate 1000 957341429459740.31_CDS_proteins_MT-CO1.fasta
```

Now, checking the `used_methods` dictionary, we realize the gravity of our mistake, as the new `AlnConf` is stored under the same key as the old one, which is now gone from both the `used_methods` and the `alignmnet` dictionaries:

In [16]:

```
print 'Alignments:'
print pj.alignments
print
print 'Used Methods:'
print pj.used_methods
```

```
Alignments:
{'MT-CO1@mafftDefault': <<class 'Bio.Align.MultipleSeqAlignment'> instance (92 records of length 1566, IUPACAmbiguousDNA()) at 7f18d6b60690>}

Used Methods:
{'mafftDefault': <reprophylo.AlnConf instance at 0x7f18d6d72950>}
```

Checking the string representation of the `AlnConf` object, which has the same name as the old one, will confirm it shows the new command line, rather than the old one:

In [17]:

```
print pj.used_methods['mafftDefault']
```

```
AlnConf named mafftDefault with ID 957341429459740.31
Loci: MT-CO1 
Created on: Sun Apr 19 17:09:00 2015
Commands:
MT-CO1: mafft --localpair --maxiterate 1000 957341429459740.31_CDS_proteins_MT-CO1.fasta

Environment:
Platform: Linux-3.13.0-40-generic-x86_64-with-Ubuntu-14.04-trusty
 Processor: x86_64
 Python build: defaultMar 22 2014 22:59:56
 Python compiler: GCC 4.8.2
 Python implementation: CPython
 Python version: 2.7.6
 ete2 version: 2.2rev1056
 biopython version: 1.64
 dendropy version: 3.12.0
 cloud version: 2.8.5
 User: amir-TECRA-W50-A
 Program and version: MAFFT v7.123b\nPal2Nal v14
 Program reference:Katoh
 Standley 2013 (Molecular Biology and Evolution 30:772-780) MAFFT multiple sequence alignment software version 7: improvements in performance and usability.\nMikita Suyama
 David Torrents
 and Peer Bork (2006) PAL2NAL: robust conversion of protein sequence alignments into the corresponding codon alignments.Nucleic Acids Res. 34
 W609-W612.
execution time:
3.85839509964


==============================
Core Methods section sentence:
==============================
The dataset(s) MT-CO1 were first aligned at the protein level using the program MAFFT v7.123b [1].
The resulting alignments served as guides to codon-align the DNA sequences using Pal2Nal v14 [2].

Reference:
[1]Katoh, Standley 2013 (Molecular Biology and Evolution 30:772-780) MAFFT multiple sequence alignment software version 7: improvements in performance and usability.
[2]Mikita Suyama, David Torrents, and Peer Bork (2006) PAL2NAL: robust conversion of protein sequence alignments into the corresponding codon alignments.Nucleic Acids Res. 34, W609-W612.
```

Since ReproPhylo maintains a Git repository, it is possible to recover from this blunder. We can spot an old version that contains the original alignment step and revert to it. The older versions can be listed with `pj.show_commits()`, as below, and they are listed with the newest at the top. The versions, termed 'commits', has hash identifiers, listed at the top of each version's record. The top version is the current one, and the one to revert to is just below, as indicated by the `AlnConf` descriptions in each of them:

In [18]:

```
pj.show_commits()
```

```
commit dfff4d62b1b4e4d9101b1bbb737d8d7a48a6dd70
Author: Amir Szitenberg <szitenberg@gmail.com>
Date:   Sun Apr 19 17:09:04 2015 +0100

    AlnConf named mafftDefault with ID 957341429459740.31
    Loci: MT-CO1
    Created on: Sun Apr 19 17:09:00 2015
    Commands:
    MT-CO1: mafft --localpair --maxiterate 1000 957341429459740.31_CDS_proteins_MT-CO1.fasta
    
    Environment:
    Platform: Linux-3.13.0-40-generic-x86_64-with-Ubuntu-14.04-trusty
     Processor: x86_64
     Python build: defaultMar 22 2014 22:59:56
     Python compiler: GCC 4.8.2
     Python implementation: CPython
     Python version: 2.7.6
     ete2 version: 2.2rev1056
     biopython version: 1.64
     dendropy version: 3.12.0
     cloud version: 2.8.5
     User: amir-TECRA-W50-A
     Program and version: MAFFT v7.123b\nPal2Nal v14
     Program reference:Katoh
     Standley 2013 (Molecular Biology and Evolution 30:772-780) MAFFT multiple sequence alignment software version 7: improvements in performance and usability.\nMikita Suyama
     David Torrents
     and Peer Bork (2006) PAL2NAL: robust conversion of protein sequence alignments into the corresponding codon alignments.Nucleic Acids Res. 34
     W609-W612.
    execution time:
    3.85839509964
    
    ==============================
    Core Methods section sentence:
    ==============================
    The dataset(s) MT-CO1 were first aligned at the protein level using the program MAFFT v7.123b [1].
    The resulting alignments served as guides to codon-align the DNA sequences using Pal2Nal v14 [2].
    
    Reference:
    [1]Katoh, Standley 2013 (Molecular Biology and Evolution 30:772-780) MAFFT multiple sequence alignment software version 7: improvements in performance and usability.
    [2]Mikita Suyama, David Torrents, and Peer Bork (2006) PAL2NAL: robust conversion of protein sequence alignments into the corresponding codon alignments.Nucleic Acids Res. 34, W609-W612.

commit 22c27d5a25710ec78cdd333d8ef3f593e61d7031
Author: Amir Szitenberg <szitenberg@gmail.com>
Date:   Sun Apr 19 17:08:52 2015 +0100

    AlnConf named mafftDefault with ID 700311429459726.66
    Loci: MT-CO1
    Created on: Sun Apr 19 17:08:46 2015
    Commands:
    MT-CO1: mafft 700311429459726.66_CDS_proteins_MT-CO1.fasta
    
    Environment:
    Platform: Linux-3.13.0-40-generic-x86_64-with-Ubuntu-14.04-trusty
     Processor: x86_64
     Python build: defaultMar 22 2014 22:59:56
     Python compiler: GCC 4.8.2
     Python implementation: CPython
     Python version: 2.7.6
     ete2 version: 2.2rev1056
     biopython version: 1.64
     dendropy version: 3.12.0
     cloud version: 2.8.5
     User: amir-TECRA-W50-A
     Program and version: MAFFT v7.123b\nPal2Nal v14
     Program reference:Katoh
     Standley 2013 (Molecular Biology and Evolution 30:772-780) MAFFT multiple sequence alignment software version 7: improvements in performance and usability.\nMikita Suyama
     David Torrents
     and Peer Bork (2006) PAL2NAL: robust conversion of protein sequence alignments into the corresponding codon alignments.Nucleic Acids Res. 34
     W609-W612.
    execution time:
    1.52710604668
    
    ==============================
    Core Methods section sentence:
    ==============================
    The dataset(s) MT-CO1 were first aligned at the protein level using the program MAFFT v7.123b [1].
    The resulting alignments served as guides to codon-align the DNA sequences using Pal2Nal v14 [2].
    
    Reference:
    [1]Katoh, Standley 2013 (Molecular Biology and Evolution 30:772-780) MAFFT multiple sequence alignment software version 7: improvements in performance and usability.
    [2]Mikita Suyama, David Torrents, and Peer Bork (2006) PAL2NAL: robust conversion of protein sequence alignments into the corresponding codon alignments.Nucleic Acids Res. 34, W609-W612.

commit b54e47d9619aa5f4db64b78253ec6e4c6171dd88
Author: Amir Szitenberg <szitenberg@gmail.com>
Date:   Sun Apr 19 17:08:41 2015 +0100

    1 genbank/embl data file(s) from Sun Apr 19 17:08:41 2015

commit 66f6ec8386d4cf439cfe66db994ea5a708389619
Author: Amir Szitenberg <szitenberg@gmail.com>
Date:   Sun Apr 19 17:08:39 2015 +0100

    Project object with the loci MT-CO1, from Sun Apr 19 17:08:39 2015

commit ca6444d5d9f9e7c87c2045ec3bee2c851aa8ecb7
Author: Amir Szitenberg <szitenberg@gmail.com>
Date:   Sun Apr 19 17:08:39 2015 +0100

    2 script file(s) from Sun Apr 19 17:08:39 2015
```

The hash identifier of a commit is required in order to revert to it. We will revert to the second newest version, with the hash that begins with `9649b5312d09`

This is done as follows:

In [19]:

```
pj = revert_pickle(pj, '22c27d5a25710ec78')
```

```
Git STDOUT: 
Git STDERR:
```

```
/home/amir/Dropbox/python_modules/reprophylo.py:232: UserWarning: Git repository exists for this Project
  warnings.warn('Git repository exists for this Project')
```

Git has raised no massages, which is a good thing. The Git repository is recognized and will be further maintain. Note that only the pickle reverted, the rest of the files, such as scripts, notebooks and sequence files has not. Also note that if instead of the line above, we run `revert_pickle(pj, '9649b5312d09')`, the pickle file is still reverted, but not loaded as a `Project`. It is still possible to load it with `pj=unpickle_pj('git_demo_files/git_demo')`

Now we can confirm the state of our reverted sequence alignment by printing the string representation of the used `AlnConf` object again, and see that the command line has changed back to its original form, of MAFFT defaults. The 'Short version' in the top of this page, also shows how to produce a `Project` with both alignments coexisting.

In [20]:

```
print pj.used_methods.keys()
```

```
['mafftDefault']
```

In [21]:

```
print pj.used_methods['mafftDefault']
```

```
AlnConf named mafftDefault with ID 700311429459726.66
Loci: MT-CO1 
Created on: Sun Apr 19 17:08:46 2015
Commands:
MT-CO1: mafft 700311429459726.66_CDS_proteins_MT-CO1.fasta

Environment:
Platform: Linux-3.13.0-40-generic-x86_64-with-Ubuntu-14.04-trusty
 Processor: x86_64
 Python build: defaultMar 22 2014 22:59:56
 Python compiler: GCC 4.8.2
 Python implementation: CPython
 Python version: 2.7.6
 ete2 version: 2.2rev1056
 biopython version: 1.64
 dendropy version: 3.12.0
 cloud version: 2.8.5
 User: amir-TECRA-W50-A
 Program and version: MAFFT v7.123b\nPal2Nal v14
 Program reference:Katoh
 Standley 2013 (Molecular Biology and Evolution 30:772-780) MAFFT multiple sequence alignment software version 7: improvements in performance and usability.\nMikita Suyama
 David Torrents
 and Peer Bork (2006) PAL2NAL: robust conversion of protein sequence alignments into the corresponding codon alignments.Nucleic Acids Res. 34
 W609-W612.
execution time:
1.52710604668


==============================
Core Methods section sentence:
==============================
The dataset(s) MT-CO1 were first aligned at the protein level using the program MAFFT v7.123b [1].
The resulting alignments served as guides to codon-align the DNA sequences using Pal2Nal v14 [2].

Reference:
[1]Katoh, Standley 2013 (Molecular Biology and Evolution 30:772-780) MAFFT multiple sequence alignment software version 7: improvements in performance and usability.
[2]Mikita Suyama, David Torrents, and Peer Bork (2006) PAL2NAL: robust conversion of protein sequence alignments into the corresponding codon alignments.Nucleic Acids Res. 34, W609-W612.
```

### Possible error messages¶

If you are not using the Docker ReproPhylo distribution, and you are new to Git, you might get the following error when you start a new `Project` with `pj=Project('loci_file',pickle='pikle_filename')`:

**`RuntimeError: Git: set your email with '!git config --global user.email "your_email@example.com"' or disable git (the ! is needed in IPython Notebook. In a terminal, ommit it)`**

This is because git expects your email to be configured. To configure it, run the following in a terminal:

`git config --global user.email "your_email@example.com"`

Another possible error when you start a new `Project` with `pj=Project('loci_file',pickle='pikle_filename')`, as opposed to loading one with `unpickle_pj` or with `revert_pickle`, can arise because `Project` expects `pickle` to be a file name that does not yet exist. Otherwise, the following error will be raised,

**`IOError: Pickle git_demo_files/git_demo exists. If you want to keep using it do pj=unpickle_pj('git_demo_files/git_demo') instead.`**

to protect you from unintentionally deleting existing projects.

ReproPhylo also tries to make sure that an unpickled, reverted or new `Project` can identify its unique Git repository. This connection can be broken if a Git reporsitory already existed in the working directory, which does not belong to the current `Project` or if the pickle file was moved independently from the directory in which it is found. The Git repository is found in a directory called `.git`, which is a hidden directory. To view hidden files and folders in your file browser, click `ctrt+H`. If you want to move the `Project` to another location, the folder containing both the `.git` directory and the pickle file must be moved as one unit. Should the connection between a `Project` and its Git repository be broken, the following error wil be show:

**`RuntimeError: The Git repository in the CWD does not belong to this project. Either the pickle moved, or this is a preexsisting repo. Try one of the following: Delete the local .Git dir if you don't need it, move the pickle and the notebook to a new work dir, or if possible, move them back to their original location. You may also disable Git by with stop_git().`**

Note that even if the link between a repository and a `project` was broken, the pickle file still contains the full `Project` and is totally usable, by passing `git=False`.

In [ ]:

```

```
